# Supplementary material for: Modular and stereoselective synthesis of tetrasubstituted vinyl sulfides leading to a library of AIEgens
Source: Nat Commun. 2021 Dec 15;12:7298. doi: 10.1038/s41467-021-27167-x (PMC8674301; doi:10.1038/s41467-021-27167-x)
Supplement: Supplementary file 1 — Supplementary Information [file 41467_2021_27167_MOESM1_ESM.pdf]

Supplementary Information

Modular and Seteroselective Synthesis of Tetrasubstituted Vinyl Sulfides Leading to a Library of AIEgens

Xun-shen Liu, Zhiqiong Tang, Zhiming Li, Mingjia Li, Lin Xu, and Lu Liu**

Contents

Supplementary Methods **2**

1. General information 2
2. Optimization of reaction conditions 3
3. General procedure for the synthesis of **3** and **11** 4
4. General procedure for the synthesis of **1** and **10** 27
5. General procedure for the synthesis of **2** 36
6. Gram scale preparation of **3** and synthetic application 43
7. Crossover experiment 47
8. DFT Calculation 48
9. X-ray crystal data for **3aa**, **3aw**, **11c**, **11d** 54
10. Photophysical properties of products 62
11. NMR Spectra 78

Supplementary References **186**

Supplementary Methods

1. General information

Unless otherwise noted, all reactions were carried out in standard Schlenk techniques with magnetic stirring bar under air. Materials obtained from commercial suppliers were used directly without further purification. ^{1}H NMR spectra were recorded on a BRUKER 500 (500 MHz) or BRUKER 600 (600 MHz) spectrometer in $CDCl_3$. Chemical shifts are reported in ppm with tetramethylsilane (TMS: 0 ppm) with the solvent resonance as the internal standard. Data are reported as follows: chemical shift, multiplicity (s = singlet, d = doublet, t = triplet, q = quartet, quint = quintus, sext = sextus, sept = septimum, dd = doublet of doublet, m = multiplet), coupling constants (Hz), and integration. ^{13}C NMR spectra were recorded on a BRUKER 500 (125 MHz) or BRUKER 600 (150 MHz) spectrometer in $CDCl_3$ with complete proton decoupling. Chemical shifts are reported in ppm with the deuterium solvent as the internal standard (e.g. $CDCl_3$: 77.0 ppm).

For the photochemical activity test data of compounds prepared in this article, we use the following equipment. UV-vis absorption spectra were measured by Agilent Cary 60 UV-Vis spectrophotometer at room temperature. Photoluminescence spectra (PL) were collected by Hitachi F-7000 spectrophotometer at room temperature. The solid-state emission spectrum is tested using Simadzu RF-6000 at room temperature. Fluorescence lifetime was obtained with Edinburgh FLS980 fluorescence spectrometer (U.K.).

Anhydrous tetrahydrofuran (THF), toluene, 1,4-Dioxane and diethyl ether (Et_2O) were distilled from sodium and benzophenone to use; Anhydrous dichloromethane (DCM) were distilled from CaH_2; $In(OTf)_3$, $Zn(OTf)_2$, $Fe(OTf)_3$, AgOTf, $Cu(OTf)_2$, $Ni(OTf)_2$, $Rh_2(Piv)_4$, $Rh_2(TFA)_4$ and $Rh_2(OAc)_4$ were purchased from Energy Chemical Company and used directly.

Reactions were monitored by thin layer chromatography (TLC) using silicycle pre-coated silica gel plates. Flash column chromatography was performed on silica gel 60 (particle size 200-400 mesh ASTM, purchased from Yantai, China) and eluted with petroleum ether/ethyl acetate (PE/EtOAc).

2. Optimization of reaction conditions

Supplementary Table 1. Optimization of reaction conditions

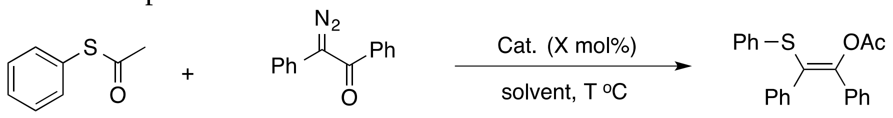

1a **2a** **3aa**

Entry	Cat. (X mol%)	Solvent	**1a** : **2a**	Time (min)[b]	Yield (%)[c]
1	$In(OTf)_3$ (5)	DCM	2:1	60	0
2	$Zn(OTf)_2$ (5)	DCM	2:1	60	0
3	$Fe(OTf)_3$ (5)	DCM	2:1	60	0
4	AgOTf (5)	DCM	2:1	30	16
5	$Cu(OTf)_2$ (5)	DCM	2:1	60	17
6	$Ni(OTf)_2$ (5)	DCM	2:1	480	NR.
7	$Rh_2(Piv)_4$ (5)	DCM	2:1	5	37
8	$Rh_2(TFA)_4$ (5)	DCM	2:1	5	52
9	$Rh_2(OAc)_4$ (5)	DCM	2:1	5	76
11	$Rh_2(OAc)_4$ (5)	DCM	1.2:1	5	70
12	$Rh_2(OAc)_4$ (5)	toluene	2:1	5	50
13	$Rh_2(OAc)_4$ (5)	THF	2:1	5	0
14[d]	$Rh_2(OAc)_4$ (5)	n-pentane	2:1	30	72
15	$Rh_2(OAc)_4$ (5)	DCM	1:1	5	75
16	$Rh_2(OAc)_4$ (5)	DCM	1:1.2	5	80
17	$Rh_2(OAc)_4$ (5)	DCM	1:1.5	5	86
18	$Rh_2(OAc)_4$ (5)	DCM	1:2	5	82
19	$Rh_2(OAc)_4$ (2)	DCM	1:1.5	5	90
20	$Rh_2(OAc)_4$ (1)	DCM	1:1.5	5	82
21[e]	$Rh_2(OAc)_4$ (2)	DCM	1:2	10	86
22[f]	$Rh_2(OAc)_4$ (2)	DCM	1:1.5	5	91(90)

[a] The reaction scale is 0.2 mmol and using solvent (1.0 mL); [b] consumed **2a** completely decomposition by TLC analysis as reaction time; [c] Yields were determined by ^{1}H-NMR using CH_2Br_2 as internal standard and the number in parenthesis is isolated yield; [d] Add 0.1 ml DCM to dissolve **2a**; [e] the reaction at 0 ℃; [f] **1a** (0.5 mmol), **2a** (0.75 mmol), $Rh_2(OAc)_4$ (2.0 mol %) and solvent (2 mL).

3. General procedure for the synthesis of 3 and 11

General synthesis method A to obtain 3 and 11

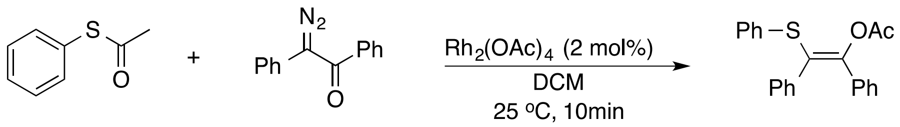

Method A: Thioester **1a** (76.1 mg, 0.5 mmol, 1.0 equiv), and $Rh_2(OAc)_4$ (4.4 mg, 0.01 mmol, 2.0 mol%) were introduced into a dried glass tube under N_2 protection, and add 1mL dry DCM as solvent, then the diazoketone **2a** (166.7 mg, 0.75 mmol, 1.5 equiv) was dissolved in 1 ml of DCM and add dropwise in 5 min at room temperature. After the addition, continue to react for 1 minute consumed diazo completely determined by TLC analysis. The mixture was purified by column chromatography on silica gel using PE/EtOAc (30:1) as the eluent and concentrated to obtain the product **3aa** (155.2 mg, 90%). Synthesize **3** and **11** according to this method.

1) (*Z*)-1,2-diphenyl-2-(phenylthio)vinyl acetate (3aa)

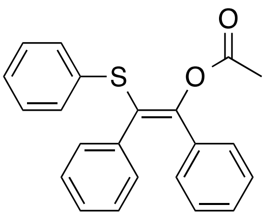

3aa

The general procedure was followed using **1a** (76.1 mg, 0.5 mmol, 1.0 equiv), $Rh_2(OAc)_4$ (4.4 mg, 0.01 mmol, 2.0 mol%) and **2a** (166.7 mg, 0.75 mmol, 1.5 equiv) by method A (R_f = 0.46, PE/EtOAc = 20:1). After purification by column chromatography (PE/EtOAc 30:1), **3aa** (155.2 mg, 90%) was obtained as white solid; m.p. = 145 – 146 ℃ ; ^{1}H NMR (600 MHz, $CDCl_3$) δ 7.27 – 7.24 (m, 2H), 7.23 – 7.19 (m, 2H), 7.17 – 7.11 (m, 5H), 7.10 – 7.01 (m, 6H), 2.26 (s, 3H); ^{13}C NMR (150 MHz, $CDCl_3$) δ 169.1, 146.3, 136.0, 135.1, 133.0, 131.7, 130.9, 128.8, 128.5, 128.2, 127.9, 127.8, 127.6, 127.3, 126.9, 20.9; HRMS (ESI-TOF) m/z: $[M+Na]^+$ calculated for $C_{22}H_{18}NaO_2S$ 369.0925, found 369.0920.

2) (*Z*)-2-((4-fluorophenyl)thio)-1,2-diphenylvinyl acetate (3ba)

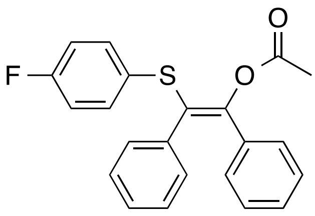

3ba

The general procedure was followed using **1b** (85.1 mg, 0.5 mmol, 1.0 equiv), $Rh_2(OAc)_4$ (4.4 mg, 0.01 mmol, 2.0 mol%) and **2a** (166.7 mg, 0.75 mmol, 1.5 equiv) by method A (R_f = 0.49, PE/EtOAc = 20:1). After purification by column chromatography (PE/EtOAc 30:1), **3ba** (152.1 mg, 83%) was obtained as white solid; m.p. = 86 – 88 °C ; ^{1}H NMR (500 MHz, $CDCl_3$) δ 7.25 – 7.21 (m, 2H), 7.17 – 7.10 (m, 7H), 7.07 – 7.02 (m, 3H), 6.78 (t, *J* = 8.7 Hz, 2H), 2.30 (s, 3H); ^{13}C NMR (125 MHz, $CDCl_3$) δ 169.2, 162.2 (d, *J* = 247.2 Hz), 145.5, 135.6, 134.9, 134.5 (d, *J* = 8.2 Hz), 130.8, 128.7, 128.2, 128.0, 127.9, 127.8, 127.75 (d, *J* = 3.3 Hz), 127.68, 115.6 (d, *J* = 22.1 Hz), 20.98; HRMS (ESI-TOF) m/z: $[M+Na]^+$ calculated for $C_{22}H_{17}FNaO_2S$ 387.0831, found 387.0832.

3) (*Z*)-2-((4-chlorophenyl)thio)-1,2-diphenylvinyl acetate (3ca)

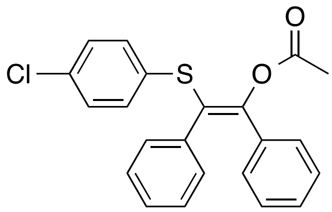

3ca

The general procedure was followed using **1c** (93.3 mg, 0.5 mmol, 1.0 equiv), $Rh_2(OAc)_4$ (4.4 mg, 0.01 mmol, 2.0 mol%) and **2a** (166.7 mg, 0.75 mmol, 1.5 equiv) by method A (R_f = 0.49, PE/EtOAc = 20:1). After purification by column chromatography (PE/EtOAc 30:1), **3ca** (170.9 mg, 90%) was obtained as white solid; m.p. = 137 – 139 °C ; ^{1}H NMR (500 MHz, $CDCl_3$) δ 7.23 – 7.18 (m, 3H), 7.17 – 7.11 (m, 6H), 7.08 – 7.04 (m, 5H), 2.29 (s, 3H); ^{13}C NMR (125 MHz, $CDCl_3$) δ 169.1, 146.7, 135.6, 134.9, 132.9, 132.8, 131.6, 130.8, 128.8, 128.7, 128.4, 128.0, 127.9, 127.8, 126.8, 20.9; HRMS (ESI-TOF) m/z: $[M+Na]^+$ calculated for $C_{22}H_{17}ClNaO_2S$ 403.0535, found 403.0525.

4) (*Z*)-2-((4-bromophenyl)thio)-1,2-diphenylvinyl acetate (3da)

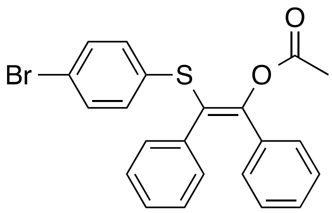

3da

The general procedure was followed using **1d** (115.6 mg, 0.5 mmol, 1.0 equiv), $Rh_2(OAc)_4$ (4.4 mg, 0.01 mmol, 2.0 mol%,) and **2a** (166.7 mg, 0.75 mmol, 1.5 equiv) by method A (R_f = 0.49, PE/EtOAc = 20:1). After purification by column chromatography (PE/EtOAc 30:1), **3da** (193.5 mg, 91%) was obtained as white solid; m.p. = 149 – 151 ℃ ; ^{1}H NMR (500 MHz, $CDCl_3$) δ 7.24 – 7.18 (m, 4H), 7.17 – 7.12 (m, 5H), 7.11 – 7.08 (m, 2H), 7.07 – 7.04 (m, 3H), 2.28 (s, 3H); ^{13}C NMR (125 MHz, $CDCl_3$) δ 169.1, 147.0, 135.6, 134.9, 132.9, 132.4, 131.6, 130.8, 128.8, 128.4, 128.0, 128.0, 127.8, 126.5, 120.9, 20.9; HRMS (ESI-TOF) m/z: $[M+Na]^+$ calculated for $C_{22}H_{17}BrNaO_2S$ 447.0030, found 447.0032.

5) (*Z*)-2-((4-iodophenyl)thio)-1,2-diphenylvinyl acetate (3ea)

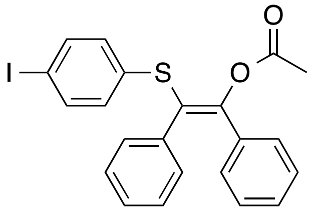

3ea

The general procedure was followed using **1e** (139.1 mg, 0.5 mmol, 1.0 equiv), $Rh_2(OAc)_4$ (4.4 mg, 0.01 mmol, 2.0 mol%,) and **2a** (166.7 mg, 0.75 mmol, 1.5 equiv) by method A (R_f = 0.49, PE/EtOAc = 20:1). After purification by column chromatography (PE/EtOAc 30:1), **3ea** (223.5 mg, 95%) was obtained as light brown solid; m.p. = 154 – 155 °C ; ^{1}H NMR (600 MHz, $CDCl_3$) δ 7.40 (d, J = 8.2 Hz, 2H), 7.24 – 7.19 (m, 2H), 7.19 – 7.11 (m, 5H), 7.09 – 7.02 (m, 3H), 6.97 (d, J = 8.2 Hz, 2H), 2.27 (s, 3H); ^{13}C NMR (150 MHz, $CDCl_3$) δ 169.1, 147.3, 137.5, 135.6, 134.9, 133.4, 132.8, 130.8, 128.8, 128.4, 128.0, 128.0, 127.8, 126.2, 92.1, 20.9; HRMS (ESI-TOF) m/z: $[M+Na]^+$ calculated for $C_{22}H_{17}INaO_2S$ 494.9892, found 494.9894.

6) (*Z*)-2-((4-nitrophenyl)thio)-1,2-diphenylvinyl acetate (3fa)

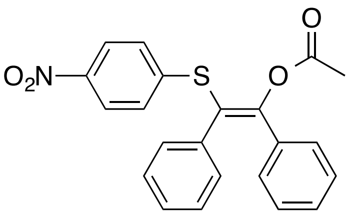

3fa

The general procedure was followed using **1f** (98.6 mg, 0.5 mmol, 1.0 equiv), $Rh_2(OAc)_4$ (4.4 mg, 0.01 mmol, 2.0 mol%,) and **2a** (166.7 mg, 0.75 mmol, 1.5 equiv) by method A (R_f = 0.44, PE/EtOAc = 10:1). After purification by column chromatography (PE/EtOAc 10:1 to 5:1), **3fa** (178.2 mg, 91%) was obtained as yellow solid; m.p. = 145 – 147 °C ; ^{1}H NMR (500 MHz, $CDCl_3$) δ 7.96 (d, *J* = 8.9 Hz, 2H), 7.35 (d, *J* = 8.9 Hz, 2H), 7.33 – 7.27 (m, 2H), 7.24 – 7.15 (m, 5H), 7.12 – 7.05 (m, 3H), 2.27 (s, 3H); ^{13}C NMR (125 MHz, $CDCl_3$) δ 169.0, 150.1, 145.8, 143.8, 135.4, 134.5, 130.7, 129.2, 128.99, 128.97, 128.3, 128.2, 128.1, 123.9, 123.6, 20.9; HRMS (ESI-TOF) m/z: $[M+Na]^+$ calculated for $C_{22}H_{17}NNaO_4S$ 414.0776, found 414.0766.

7) (*Z*)-1,2-diphenyl-2-(*p*-tolylthio)vinyl acetate (3ga)

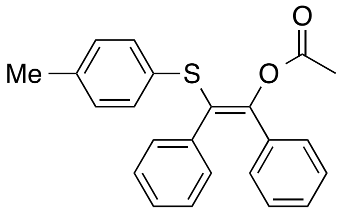

3ga

The general procedure was followed using **1g** (83.1 mg, 0.5 mmol, 1.0 equiv), $Rh_2(OAc)_4$ (4.4 mg, 0.01 mmol, 2.0 mol%,) and **2a** (166.7 mg, 0.75 mmol, 1.5 equiv) by method A (R_f = 0.44, PE/EtOAc = 20:1). After purification by column chromatography (PE/EtOAc 30:1), **3ga** (148.1 mg, 82%) was obtained as white solid; m.p. = 122 – 123 °C ; ^{1}H NMR (500 MHz, $CDCl_3$) δ 7.22 – 7.17 (m, 2H), 7.16 – 7.09 (m, 7H), 7.06 – 7.01 (m, 3H), 6.89 (d, *J* = 7.3 Hz, 2H), 2,27 (s, 3H), 2.19 (s, 3H); ^{13}C NMR (125 MHz, $CDCl_3$) δ 169.1, 145.6, 136.9, 136.0, 135.2, 132.0, 130.8, 129.3, 129.1, 128.7, 128.1, 127.9, 127.8, 127.5, 21.0, 20.9; HRMS (ESI-TOF) m/z: $[M+Na]^+$ calculated for $C_{23}H_{20}NaO_2S$ 383.1082, found 383.1080.

8) (*Z*)-2-((4-methoxyphenyl)thio)-1,2-diphenylvinyl acetate (3ha)

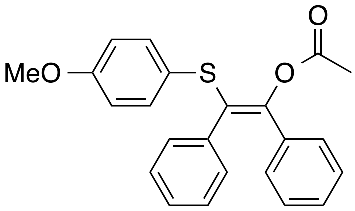

3ha

The general procedure was followed using **1h** (91.1 mg, 0.5 mmol, 1.0 equiv), $Rh_2(OAc)_4$ (4.4 mg, 0.01 mmol, 2.0 mol%,) and **2a** (166.7 mg, 0.75 mmol, 1.5 equiv) by method A (R_f = 0.43, PE/EtOAc = 10:1). After purification by column chromatography (PE/EtOAc 10:1), **3ha** (166.2 mg, 88%) was obtained as white solid; m.p. = 89 – 91 °C ; ^{1}H NMR (500 MHz, $CDCl_3$) δ 7.18 (d, *J* = 8.8 Hz, 2H), 7.15 – 7.08 (m, 7H), 7.06 – 7.00 (m, 3H), 6.62 (d, *J* = 8.8 Hz, 2H), 3.69 (s, 3H), 2.31 (s, 3H); ^{13}C NMR (125 MHz, $CDCl_3$) δ 169.2, 159.2, 144.3, 135.8, 135.1, 134.8, 130.8, 128.8, 128.6, 127.9, 127.8, 127.8, 127.5, 122.9, 114.1, 55.2, 21.0; HRMS (ESI-TOF) m/z: $[M+Na]^+$ calculated for $C_{23}H_{20}NaO_3S$ 399.1031, found 399.1026.

9) (*Z*)-2-((3-chlorophenyl)thio)-1,2-diphenylvinyl acetate (3ia)

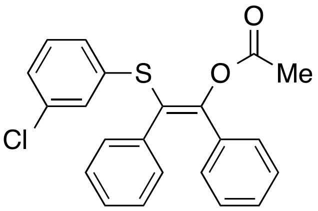

3ia

The general procedure was followed using **1i** (93.3 mg, 0.5 mmol, 1.0 equiv), $Rh_2(OAc)_4$ (4.4 mg, 0.01 mmol, 2.0 mol%,) and **2a** (166.7 mg, 0.75 mmol, 1.5 equiv) by method A (R_f = 0.49, PE/EtOAc = 20:1). After purification by column chromatography (PE/EtOAc 30:1), **3ia** (173.2 mg, 91%) was obtained as white solid; m.p. = 95 – 96 °C ; ^{1}H NMR (500 MHz, $CDCl_3$) δ 7.25 – 7.21 (m, 3H), 7.18 – 7.11 (m, 6H), 7.09 – 7.05 (m, 3H), 7.03 – 7.00 (m, 2H), 2.27 (s, 3H); ^{13}C NMR (125 MHz, $CDCl_3$) δ 169.0, 147.3, 135.6, 135.3, 134.9, 134.1, 130.8, 130.8, 129.5, 129.2, 128.8, 128.4, 128.0, 128.0, 127.8, 126.9, 126.2, 20.9; HRMS (ESI-TOF) m/z: $[M+Na]^+$ calculated for $C_{22}H_{17}ClNaO_2S$ 403.0535, found 403.0535.

10) (*Z*)-2-((2-bromophenyl)thio)-1,2-diphenylvinyl acetate (3ja)

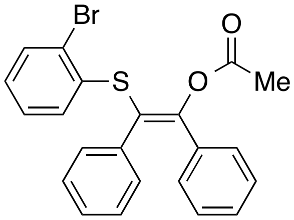

3ja

The general procedure was followed using **1j** (115.6 mg, 0.5 mmol, 1.0 equiv), $Rh_2(OAc)_4$ (4.4 mg, 0.01 mmol, 2.0 mol%,) and **2a** (166.7 mg, 0.75 mmol, 1.5 equiv) by method A (R_f = 0.46, PE/EtOAc = 20:1). After purification by column chromatography (PE/EtOAc 30:1), **3ja** (177.8 mg, 84%) was obtained as white solid; m.p. = 184 – 186 °C ; ^{1}H NMR (500 MHz, $CDCl_3$) δ 7.39 (dd, *J* = 8.0, 1.4 Hz, 1H), 7.32 – 7.27 (m, 3H), 7.21 – 7.12 (m, 5H), 7.08 – 7.04 (m, 3H), 7.02 (td, *J* = 7.7, 1.3 Hz, 1H), 6.89 (td, *J* = 7.6, 1.6 Hz, 1H), 2.26 (s, 3H); ^{13}C NMR (125 MHz, $CDCl_3$) δ 169.1, 148.1, 135.7, 135.0, 134.9, 132.7, 132.3, 130.8, 128.9, 128.5, 128.0, 127.9, 127.8, 127.7, 127.3, 126.0, 124.8, 20.9; HRMS (ESI-TOF) m/z: $[M+Na]^+$ calculated for $C_{22}H_{17}BrNaO_2S$ 447.0030, found 447.0023.

11) (*Z*)-2-((3,5-bis(trifluoromethyl)phenyl)thio)-1,2-diphenylvinyl acetate (3ka)

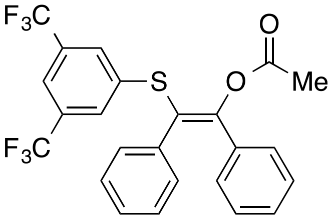

3ka

The general procedure was followed using **1k** (144.1 mg, 0.5 mmol, 1.0 equiv), $Rh_2(OAc)_4$ (4.4 mg, 0.01 mmol, 2.0 mol%,) and **2a** (166.7 mg, 0.75 mmol, 1.5 equiv) by method A (R_f = 0.59, PE/EtOAc = 20:1). After purification by column chromatography (PE/EtOAc 30:1), **3ka** (221.1 mg, 92%) was obtained as white solid; m.p. = 112 – 114 °C ; ^{1}H NMR (500 MHz, $CDCl_3$) δ 7.63 (s, 2H), 7.50(s, 1H), 7.27 – 7.23 (m, 2H), 7.22 – 7.14 (m, 5H), 7.10 – 7.05 (m, 3H), 2.30 (s, 3H); ^{13}C NMR (125 MHz, $CDCl_3$) δ 168.9, 148.9, 137.2, 134.9, 134.4, 131.6 (q, *J* = 33.5 Hz), 130.8, 130.1 (q, *J* = 3.8 Hz), 128.90, 128.86, 128.3, 128.2, 128.1, 124.5, 122.9 (q, *J* = 273.1 Hz), 120.0 (quint, *J* = 3.8 Hz), 20.9; HRMS (ESI-TOF) m/z: $[M+Na]^+$ calculated for $C_{24}H_{16}F_6NaO_2S$ 505.0673, found 505.0659.

12) (*Z*)-2-(naphthalen-2-ylthio)-1,2-diphenylvinyl acetate (3la)

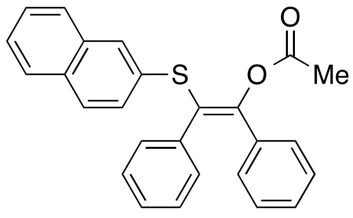

3la

The general procedure was followed using **1l** (101.1 mg, 0.5 mmol, 1.0 equiv), $Rh_2(OAc)_4$ (4.4 mg, 0.01 mmol, 2.0 mol%,) and **2a** (166.7 mg, 0.75 mmol, 1.5 equiv) by method A (R_f = 0.41, PE/EtOAc = 20:1). After purification by column chromatography (PE/EtOAc 30:1), **3la** (185.6 mg, 94%) was obtained as white solid; m.p. = 160 – 162 °C ; ^{1}H NMR (500 MHz, $CDCl_3$) δ 7.74 (s, 1H), 7.69 – 7.60 (m, 2H), 7.56 (d, *J* = 8.6 Hz, 1H), 7.41 – 7.30 (m, 3H), 7.30 – 7.25 (m, 2H), 7.22 – 7.10 (m, 5H), 7.04 – 6.93 (m, 3H), 2.27 (s, 3H); ^{13}C NMR (125 MHz, $CDCl_3$) δ 169.1, 146.7, 136.0, 135.1, 133.3, 132.0, 130.8, 130.7, 130.1, 128.8, 128.8, 128.3, 128.0, 127.9, 127.9, 127.7, 127.6, 127.2, 127.0, 126.2, 125.9, 20.9; HRMS (ESI-TOF) m/z: $[M+Na]^+$ calculated for $C_{26}H_{20}NaO_2S$ 419.1082, found 419.1069.

13) (*Z*)-2-(benzylthio)-1,2-diphenylvinyl acetate (3ma)

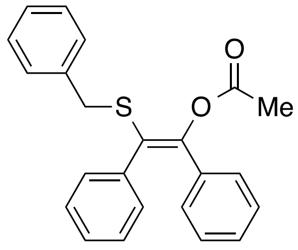

3ma

The general procedure was followed using **1m** (83.1 mg, 0.5 mmol, 1.0 equiv), $Rh_2(OAc)_4$ (4.4 mg, 0.01 mmol, 2.0 mol%,) and **2a** (166.7 mg, 0.75 mmol, 1.5 equiv) by method A (R_f = 0.44, PE/EtOAc = 20:1). After purification by column chromatography (PE/EtOAc 30:1), **3ma** (125.3 mg, 70%) was obtained as white solid; m.p. = 116 – 118 ℃ ; ^{1}H NMR (500 MHz, $CDCl_3$) δ 7.28 – 7.19 (m, 8H), 7.17 – 7.12 (m, 2H), 7.11 – 7.04 (m, 5H), 2.53 (s, 2H), 2.26 (s, 3H); ^{13}C NMR (125 MHz, $CDCl_3$) δ 168.8, 144.6, 137.4, 135.8, 135.2, 130.7, 128.9, 128.5, 128.4, 128.3, 127.9, 127.8, 127.8, 127.0, 36.3, 20.9; HRMS (ESI-TOF) m/z: $[M+Na]^+$ calculated for $C_{23}H_{20}NaO_2S$ 383.1082, found 383.1077.

14) (*Z*)-2-(methylthio)-1,2-diphenylvinyl acetate (3na)

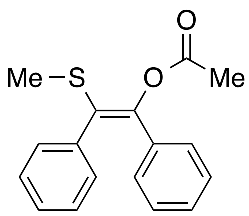

3na

The general procedure was followed using **1n** (45.1 mg, 0.5 mmol, 1.0 equiv), $Rh_2(OAc)_4$ (4.4 mg, 0.01 mmol, 2.0 mol%,) and **2a** (166.7 mg, 0.75 mmol, 1.5 equiv) by method A (R_f = 0.54, PE/EtOAc = 20:1). After purification by column chromatography (PE/EtOAc 30:1), **3na** (61.2 mg, 43%) was obtained as colorless oil. ^{1}H NMR (500 MHz, $CDCl_3$) δ 7.31 – 7.23 (m, 5H), 7.13 – 7.06 (m, 5H), 2.29 (s, 3H), 1.86 (s, 3H); ^{13}C NMR (125 MHz, $CDCl_3$) δ 168.9, 143.2, 135.3, 135.2, 130.6, 128.8, 128.5, 128.3, 127.9, 127.8, 127.7, 20.9, 14.8; HRMS (ESI-TOF) m/z: $[M+Na]^+$ calculated for $C_{17}H_{16}NaO_2S$ 307.0769, found 307.0758.

15) methyl (*Z*)-4-(2-acetoxy-1-(cyclohexylthio)-2-phenylvinyl)benzoate (3of)

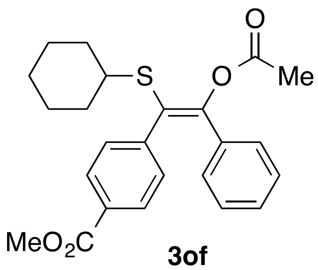

3of

The general procedure was followed using **1o** (79.1 mg, 0.5 mmol, 1.0 equiv), $Rh_2(OAc)_4$ (4.4 mg, 0.01 mmol, 2.0 mol%,) and **2f** (210.2 mg, 0.75 mmol, 1.5 equiv) by method A (R_f = 0.32, PE/EtOAc = 20:1). After purification by column chromatography (PE/EtOAc 30:1), **3of** (124.1 mg, 60%) was obtained as colorless oil. ^{1}H NMR (500 MHz, $CDCl_3$) δ 7.89 (d, *J* = 8.3 Hz, 2H), 7.41 (d, *J* = 8.4 Hz, 2H), 7.16 – 7.05 (m, 5H), 3.90 (s, 3H), 2.35 (tt, *J* = 10.6, 3.7 Hz, 1H), 2.28 (s, 3H), 1.79 – 1.71 (m, 2H), 1.69 – 1.64 (m, 2H), 1.52 – 1.44 (m, 1H), 1.35 – 1.27 (m, 2H), 1.22 – 1.12 (m, 1H), 1.09 – 0.99 (m, 2H); ^{13}C NMR (125 MHz, $CDCl_3$) δ 168.8, 166.8, 146.8, 141.9, 135.1, 130.6, 129.5, 129.1, 128.8, 128.2, 127.9, 126.7, 52.1, 43.7, 33.5, 25.7, 25.5, 20.9; HRMS (ESI-TOF) m/z: $[M+Na]^+$ calculated for $C_{24}H_{26}NaO_4S$ 433.1449, found 433.1446.

16) methyl (*Z*)-3-((2-acetoxy-1,2-diphenylvinyl)thio)propanoate (3pa)

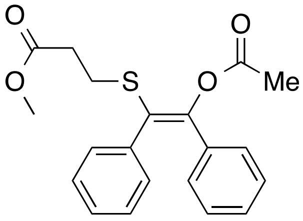

3pa

The general procedure was followed using **1p** (81.1 mg, 0.5 mmol, 1.0 equiv), $Rh_2(OAc)_4$ (4.4 mg, 0.01 mmol, 2.0 mol%,) and **2a** (166.7 mg, 0.75 mmol, 1.5 equiv) by method A (R_f = 0.25, PE/EtOAc = 20:1). After purification by column chromatography (PE/EtOAc 15:1), **3pa** (90.0 mg, 51%) was obtained as colorless oil. ^{1}H NMR (500 MHz, $CDCl_3$) δ 7.34 – 7.30 (m, 2H), 7.27 – 7.23 (m, 2H), 7.13 – 7.06 (m, 5H), 3.64 (s, 3H), 2,56 (t, *J* = 7.3 Hz, 2H), 2.43 (t, *J* = 7.2 Hz, 2H), 2.27 (s, 3H); ^{13}C NMR (125 MHz, $CDCl_3$) δ 172.0, 168.9, 145.6, 135.4, 135.1, 130.6, 128.6, 128.5, 128.1, 128.0, 127.8, 126.9, 51.7, 34.7, 26.2, 20.9; HRMS (ESI-TOF) m/z: $[M+Na]^+$ calculated for $C_{20}H_{20}NaO_4S$ 379.0980, found 379.0984.

17) (*Z*)-2-((furan-2-ylmethyl)thio)-1,2-diphenylvinyl propionate (3qa)

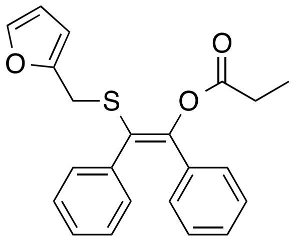

3qa

The general procedure was followed using **1q** (85.1 mg, 0.5 mmol, 1.0 equiv), $Rh_2(OAc)_4$ (4.4 mg, 0.01 mmol, 2.0 mol%,) and **2a** (166.7 mg, 0.6 mmol, 1.5 equiv) by method A (R_f = 0.54, PE/EtOAc = 10:1). After purification by column chromatography (PE/EtOAc 30:1), **3qa** (90.3 mg, 50%) was obtained as light yellow oil. ^{1}H NMR (600 MHz, $CDCl_3$) δ 7.31 – 7.21 (m, 6H), 7.13 – 7.05 (m, 5H), 6.25 (dd, *J* = 3.2, 1.9 Hz, 1H), 6.00 (d, *J* = 3.2 Hz, 1H), 3.53 (s, 2H), 2.57 (q, *J* = 7.5 Hz, 2H), 1.24 (t, *J* = 7.5 Hz, 3H); ^{13}C NMR (150 MHz, $CDCl_3$) δ 172.3, 151.0, 145.4, 141.9, 135.6, 135.2, 130.7, 128.5, 128.4, 127.9, 127.9, 127.8, 126.8, 110.4, 107.6, 28.5, 27.5, 9.0. HRMS (ESI-TOF) m/z: $[M+Na]^+$ calculated for $C_{22}H_{20}NaO_3S$ 387.1031, found 387.1015.

18) methyl (*Z*)-4-(2-(butyryloxy)-1-(methylthio)-2-phenylvinyl)benzoate (3rf)

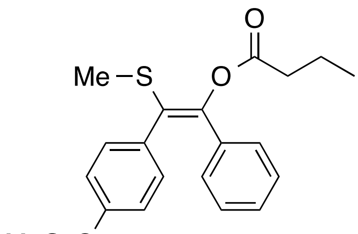

3rf

The general procedure was followed using **1r** (59.1 mg, 0.5 mmol, 1.0 equiv), $Rh_2(OAc)_4$ (4.4 mg, 0.01 mmol, 2.0 mol%,) and **2f** (210.2 mg, 0.75 mmol, 1.5 equiv) by method A (R_f = 0.41, PE/EtOAc = 10:1). After purification by column chromatography (PE/EtOAc 20:1), **3rf** (111.4 mg, 60%) was obtained as white solid; m.p. = 76 – 78 ℃; ^{1}H NMR (500 MHz, $CDCl_3$) δ 7.93 (d, *J* = 8.4 Hz, 2H), 7.37 (d, *J* = 8.5 Hz, 2H), 7.15 – 7.04 (m, 5H), 3.90 (s, 3H), 2.55 (t, *J* = 7.4 Hz, 2H), 1.86 (s, 3H), 1.79 (sext, *J* = 7.4 Hz, 2H), 1.03 (t, *J* = 7.4 Hz, 3H); ^{13}C NMR (125 MHz, $CDCl_3$) δ 171.3, 166.6, 144.6, 140.6, 134.9, 130.7, 129.7, 129.3, 128.5, 128.1, 127.9, 127.5, 52.1, 36.0, 18.3, 14.9, 13.7; HRMS (ESI-TOF) m/z: $[M+Na]^+$ calculated for $C_{21}H_{22}NaO_4S$ 393.1136, found 393.1121.

19) (*Z*)-2-((4-chlorophenyl)thio)-1,2-diphenylvinyl pivalate (3sa)

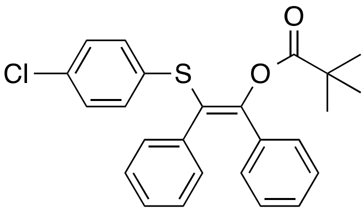

3sa

The general procedure was followed using **1s** (144.4 mg, 0.5 mmol, 1.0 equiv), $Rh_2(OAc)_4$ (4.4 mg, 0.01 mmol, 2.0 mol%,) and **2a** (166.7 mg, 0.75 mmol, 1.5 equiv) by method A (R_f = 0.66, PE/EtOAc = 20:1). After purification by column chromatography (PE/EtOAc 30:1), **3sa** (176.2 mg, 83%) was obtained as white solid; m.p. = 132 – 134 ℃; ^{1}H NMR (500 MHz, $CDCl_3$) δ 7.21 – 7.10 (m, 9H), 7.08 – 7.02 (m, 5H), 1.36 (s, 9H); ^{13}C NMR (125 MHz, $CDCl_3$) δ 176.4, 146.9, 135.9, 135.1, 132.8, 132.8, 131.8, 130.7, 128.7, 128.6, 128.2, 128.0, 127.9, 127.7, 126.5, 39.2, 27.1; HRMS (ESI-TOF) m/z: $[M+Na]^+$ calculated for $C_{25}H_{23}NaClO_2S$ 445.1005, found 445.1004.

20) (*Z*)-2-((4-bromophenyl)thio)-1,2-diphenylvinyl pivalate (3ta)

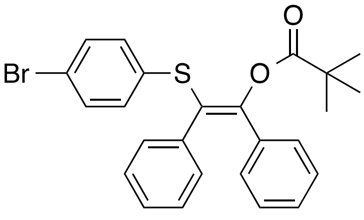

3ta

The general procedure was followed using **1t** (136.6 mg, 0.5 mmol, 1.0 equiv), $Rh_2(OAc)_4$ (4.4 mg, 0.01 mmol, 2.0 mol%,) and **2a** (166.7 mg, 0.75 mmol, 1.5 equiv) by method A (R_f = 0.66, PE/EtOAc = 20:1). After purification by column chromatography (PE/EtOAc 30:1), **3ta** (188.9 mg, 81%) was obtained as white solid; m.p. = 137 – 139 ℃; ^{1}H NMR (500 MHz, $CDCl_3$) δ 7.22 – 7.17 (m, 4H), 7.16 – 7.08 (m, 7H), 7.07 – 7.03 (m, 3H), 1.35 (s, 9H); ^{13}C NMR (125 MHz, $CDCl_3$) δ 176.4, 147.2, 135.9, 135.0, 132.9, 132.6, 131.5, 130.7, 128.7, 128.3, 128.0, 127.9, 127.7, 126.2, 120.8, 39.2, 27.1; HRMS (ESI-TOF) m/z: $[M+Na]^+$ calculated for $C_{25}H_{23}BrNaO_2S$ 489.0500, found 489.0488.

21) (*Z*)-2-((4-chlorophenyl)thio)-1,2-diphenylvinyl benzoate (3ua)

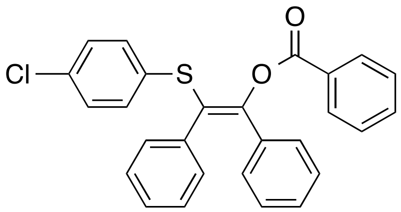

3ua

The general procedure was followed using **1u** (124.4 mg, 0.5 mmol, 1.0 equiv), $Rh_2(OAc)_4$ (4.4 mg, 0.01 mmol, 2.0 mol%,) and **2a** (166.7 mg, 0.75 mmol, 1.5 equiv) by method A (R_f = 0.56, PE/EtOAc = 20:1). After purification by column chromatography (PE/EtOAc 30:1), **3ua** (141.3 mg, 64%) was obtained as white solid; m.p. = 135 – 137 ℃; ^{1}H NMR (500 MHz, $CDCl_3$) δ 8.19 (d, *J* = 8.2 Hz, 2H), 7.61 (t, *J* = 7.4 Hz, 1H), 7.49 (t, *J* = 7.8 Hz, 2H), 7.32 – 7.25 (m, 2H), 7.25 – 7.07 (m, 10H), 7.03 (d, *J* = 8.6 Hz, 2H); ^{13}C NMR (125 MHz, $CDCl_3$) δ 164.8, 146.6, 135.8, 134.9, 133.6, 132.9, 131.7, 130.8, 130.2, 129.2, 128.8, 128.7, 128.6, 128.5, 128.4, 128.1, 128.0, 127.8, 127.1; HRMS (ESI-TOF) m/z: $[M+Na]^+$ calculated for $C_{27}H_{19}ClNaO_2S$ 465.0692, found 465.0688.

22) (*Z*)-2-((4-bromophenyl)thio)-1,2-diphenylvinyl benzoate (3va)

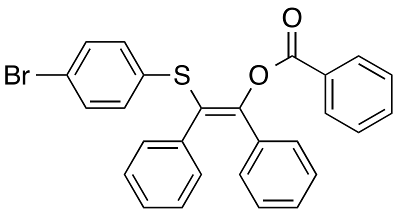

3va

The general procedure was followed using **1v** (146.6 mg, 0.5 mmol, 1.0 equiv), $Rh_2(OAc)_4$ (4.4 mg, 0.01 mmol, 2.0 mol%,) and **2a** (166.7 mg, 0.75 mmol, 1.5 equiv) by method A (R_f = 0.56, PE/EtOAc = 20:1). After purification by column chromatography (PE/EtOAc 30:1), **3va** (147.1 mg, 60%) was obtained as white solid; m.p. = 140 – 142 ℃; ^{1}H NMR (500 MHz, $CDCl_3$) δ 8.18 (d, *J* = 8.4 Hz, 2H), 7.62 (t, *J* = 7.5 Hz, 1H), 7.49 (t, *J* = 7.8 Hz, 2H), 7.31 – 7.26 (m, 2H), 7.24 – 7.07 (m, 12H); ^{13}C NMR (125 MHz, $CDCl_3$) δ 164.8, 146.9, 135.8, 134.9, 133.6, 133.0, 132.5, 131.5, 130.8, 130.2, 129.2, 128.8, 128.5, 128.4, 128.1, 128.0, 127.9, 126.8, 120.9; HRMS (ESI-TOF) m/z: $[M+Na]^+$ calculated for $C_{27}H_{19}BrNaO_2S$ 509.0187, found 509.0178.

23) (*Z*)-2-((4-bromophenyl)thio)-1,2-diphenylvinyl dimethylcarbamate (3wa)

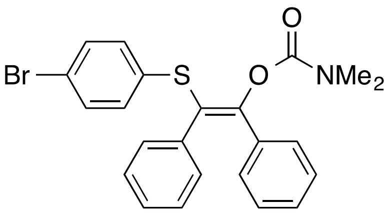

3wa

The general procedure was followed using **1w** (130.0 mg, 0.5 mmol, 1.0 equiv), $Rh_2(OAc)_4$ (4.4 mg, 0.01 mmol, 2.0 mol%,) and **2a** (166.7 mg, 0.75 mmol, 1.5 equiv) by method A (R_f = 0.50, PE/EtOAc = 5:1). After purification by column chromatography (PE/EtOAc 30:1 to 10:1), **3wa** (184.1 mg, 81%) was obtained as white solid; m.p. = 158 – 161 ℃; ^{1}H NMR (500 MHz, $CDCl_3$) δ 7.24 – 7.09 (m, 11H), 7.08 – 7.02 (m, 3H), 3.09 (s, 3H), 2.97 (s, 3H); ^{13}C NMR (125 MHz, $CDCl_3$) δ 154.2, 147.6, 136.1, 135.5, 132.9, 132.8, 131.5, 130.8, 128.9, 128.2, 128.0, 127.9, 127.6, 126.0, 120.7, 36.8, 36.5; HRMS (ESI-TOF) m/z: $[M+Na]^+$ calculated for $C_{23}H_{20}NNaO_2S$ 476.0296, found 476.0282.

24) ethyl (*Z*)-3-(2-acetoxy-1-(allylthio)-2-phenylvinyl)benzoate (3xg)

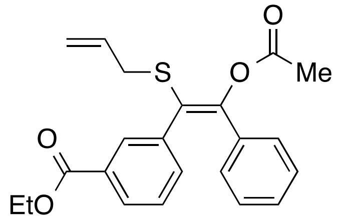

3xg

The general procedure was followed using **1x** (58.1 mg, 0.5 mmol, 1.0 equiv), $Rh_2(OAc)_4$ (4.4 mg, 0.01 mmol, 2.0 mol%,) and **2g** (220.7 mg, 0.75 mmol, 1.5 equiv) by method A (R_f = 0.40, PE/EtOAc = 10:1). After purification by column chromatography (PE/EtOAc 50:1 to 20:1), **3xg** (76.3 mg, 40%) was obtained as colorless oil. ^{1}H NMR (500 MHz, $CDCl_3$) δ 8.05 (t, *J* = 1.8 Hz, 1H), 7.91 (dt, *J* = 7.8, 1.5 Hz, 1H), 7.40 (dt, *J* = 7.8, 1.5 Hz, 1H), 7.27 (t, *J* = 7.8 Hz, 1H), 7.15 – 7.06 (m, 5H), 5.70 (ddt, *J* = 16.9, 10.0, 6.9 Hz, 1H), 4.98 (dd, *J* = 10.0, 1.2 Hz, 1H), 4.87 (dq, *J* = 16.9, 1.3 Hz, 1H), 4.35 (q, *J* = 7.1 Hz, 2H), 2.96 (d, *J* = 6.9 Hz, 2H), 2.29 (s, 3H), 1.38 (t, *J* = 7.1 Hz, 3H); ^{13}C NMR (125 MHz, $CDCl_3$) δ 168.7, 166.2, 146.1, 136.3, 135.2, 134.9, 133.8, 131.7, 130.8, 129.0, 128.7, 128.3, 128.1, 127.9, 126.4, 117.5, 61.1, 34.8, 20.9, 14.3; HRMS (ESI-TOF) m/z: $[M+Na]^+$ calculated for $C_{24}H_{22}NaO_4S$ 405.1136, found 405.1146.

25) ethyl (*Z*)-3-(2-acetoxy-2-phenyl-1-(prop-2-yn-1-ylthio)vinyl)benzoate (3yg)

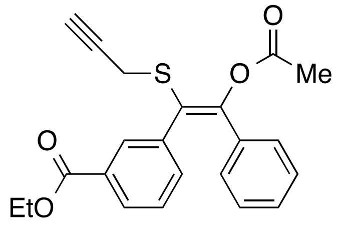

3yg

The general procedure was followed using **1y** (57.1 mg, 0.5 mmol, 1.0 equiv), $Rh_2(OAc)_4$ (4.4 mg, 0.01 mmol, 2.0 mol%,) and **2g** (220.7 mg, 0.75 mmol, 1.5 equiv) by method A (R_f = 0.37, PE/EtOAc = 10:1). After purification by column chromatography (PE/EtOAc 20:1), **3yg** (91.0 mg, 48%) was obtained as colorless oil. ^{1}H NMR (500 MHz, $CDCl_3$) δ 8.08 (t, *J* = 1.8 Hz, 1H), 7.93 (dt, *J* = 7.8, 1.5 Hz, 1H), 7.45 (dt, *J* = 7.8, 1.5 Hz, 1H), 7.29 (t, *J* = 7.7 Hz, 1H), 7.15 – 7.09 (m, 5H), 4.35 (q, *J* = 7.1 Hz, 2H), 3.03 (d, *J* = 2.6 Hz, 2H), 2.29 (s, 3H), 2.18 (t, *J* = 2.6 Hz, 1H), 1.37 (t, *J* = 7.1 Hz, 3H); ^{13}C NMR (125 MHz, $CDCl_3$) δ 168.6, 166.0, 146.9, 135.7, 135.1, 134.6, 131.6, 130.9, 129.2, 128.7, 128.5, 128.3, 127.9, 125.3, 79.2, 71.8, 61.1, 20.8, 20.0, 14.2; HRMS (ESI-TOF) m/z: $[M+Na]^+$ calculated for $C_{24}H_{22}NaO_4S$ 403.0980, found 403.0979.

26) (*Z*)-2-((4-methoxyphenyl)thio)-1,2-diphenylvinyl propionate (3za)

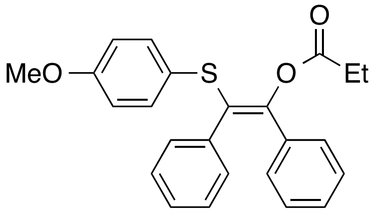

3za

The general procedure was followed using **1z** (98.1 mg, 0.5 mmol, 1.0 equiv), $Rh_2(OAc)_4$ (4.4 mg, 0.01 mmol, 2.0 mol%,) and **2a** (166.7 mg, 0.75 mmol, 1.5 equiv) by method A (R_f = 0.49, PE/EtOAc = 10:1). After purification by column chromatography (PE/EtOAc 10:1), **3za** (174.2 mg, 89%) was obtained as white solid; m.p. = 60 - 62 °C ; ^{1}H NMR (500 MHz, $CDCl_3$) δ 7.17 (d, *J* = 8.8 Hz, 2H), 7.15 – 7.05 (m, 7H), 7.04 – 6.99 (m, 3H), 6.61 (d, *J* = 8.8 Hz, 2H), 3.66 (s, 3H), 2.60 (q, *J* = 7.6 Hz, 2H), 1.26 (t, *J* = 7.6 Hz, 3H); ^{13}C NMR (125 MHz, $CDCl_3$) δ 172.6, 159.2, 144.3, 135.8, 135.2, 134.8, 130.8, 128.6, 128.5, 127.9, 127.8, 127.8, 127.4, 122.9, 114.0, 55.1, 27.6, 9.0; HRMS (ESI-TOF) m/z: $[M+Na]^+$ calculated for $C_{24}H_{22}NaO_3S$ 413.1187, found 413.1178.

27) (*Z*)-2-(4-chlorophenyl)-1-phenyl-2-(phenylthio)vinyl acetate (3ab)

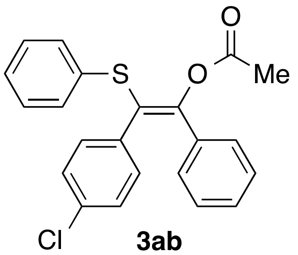

3ab

The general procedure was followed using **1a** (76.1 mg, 0.5 mmol, 1.0 equiv), $Rh_2(OAc)_4$ (4.4 mg, 0.01 mmol, 2.0 mol%) and **2b** (192.5 mg, 0.75 mmol, 1.5 equiv) by method A (R_f = 0.41, PE/EtOAc = 20:1). After purification by column chromatography (PE/EtOAc 30:1), **3ab** (175.7 mg, 92%) was obtained as white solid; m.p. = 70 – 73 ℃; ^1H NMR (500 MHz, $CDCl_3$) δ 7.25 – 7.23 (m, 2H), 7.20 – 7.13 (m, 7H), 7.13 – 7.06 (m, 3H), 7.00 (d, *J* = 8.5 Hz, 2H), 2.27 (s, 3H); ^{13}C NMR (125 MHz, $CDCl_3$) δ 169.1, 147.0, 134.8, 134.6, 133.4, 132.7, 132.1, 131.6, 128.8, 128.6, 128.5, 128.1, 128.1, 127.1, 126.2, 20.9; HRMS (ESI-TOF) m/z: $[M+Na]^+$ calculated for $C_{22}H_{17}ClNaO_2S$ 403.0535, found 403.0519.

28) (*Z*)-2-(4-bromophenyl)-1-phenyl-2-(phenylthio)vinyl acetate (3ac)

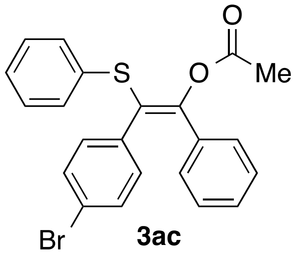

3ac

The general procedure was followed using **1a** (76.1 mg, 0.5 mmol, 1.0 equiv), $Rh_2(OAc)_4$ (4.4 mg, 0.01 mmol, 2.0 mol%) and **2c** (225.9 mg, 0.75 mmol, 1.5 equiv) by method A (R_f = 0.44, PE/EtOAc = 20:1). After purification by column chromatography (PE/EtOAc 30:1), **3ac** (188.1 mg, 88%) was obtained as yellow oil. ^1H NMR (500 MHz, $CDCl_3$) δ 7.25 – 7.22 (m, 2H), 7.20 – 7.14 (m, 7H), 7.13 – 7.06 (m, 5H), 2.27 (s, 3H); ^{13}C NMR (125 MHz, $CDCl_3$) δ 169.1, 147.1, 135.1, 134.8, 132.7, 132.4, 131.5, 131.1, 128.8, 128.7, 128.5, 128.1, 127.1, 126.2, 121.7, 20.9; HRMS (ESI-TOF) m/z: $[M+Na]^+$ calculated for $C_{22}H_{17}BrNaO_2S$ 447.0030, found 447.0014.

29) (*Z*)-2-(3-bromophenyl)-1-phenyl-2-(phenylthio)vinyl acetate (3ad)

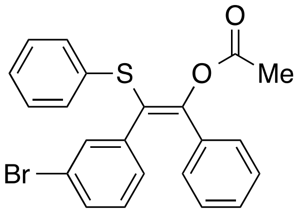

3ad

The general procedure was followed using **1a** (76.1 mg, 0.5 mmol, 1.0 equiv), $Rh_2(OAc)_4$ (4.4 mg, 0.01 mmol, 2.0 mol%) and **2d** (225.9 mg, 0.75 mmol, 1.5 equiv) by method A (R_f = 0.44, PE/EtOAc = 20:1). After purification by column chromatography (PE/EtOAc 30:1), **3ad** (180.2 mg, 85%) was obtained as white solid; m.p. = 81 – 84 ℃; ^1H NMR (500 MHz, $CDCl_3$) δ 7.41 (t, *J* = 1.8 Hz, 1H), 7.27 – 7.24 (m, 2H), 7.20 – 7.06 (m, 10H), 6.87 (t, *J* = 7.9 Hz, 1H), 2.27 (s, 3H); ^{13}C NMR (125 MHz, $CDCl_3$) δ 169.0, 147.2, 138.2, 134.6, 133.5, 132.4, 131.8, 130.6, 129.5, 129.3, 128.8, 128.6, 128.6, 128.1, 127.2, 125.9, 121.8, 20.9; HRMS (ESI-TOF) m/z: $[M+Na]^+$ calculated for $C_{22}H_{17}BrNaO_2S$ 447.0030, found 447.0006.

30) (*Z*)-2-(3-cyanophenyl)-1-phenyl-2-(phenylthio)vinyl acetate (3ae)

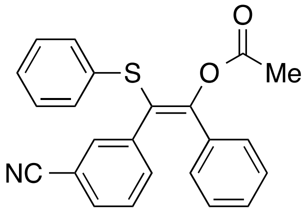

3ae

The general procedure was followed using **1a** (76.1 mg, 0.5 mmol, 1.0 equiv), $Rh_2(OAc)_4$ (4.4 mg, 0.01 mmol, 2.0 mol%) and **2e** (185.4 mg, 0.75 mmol, 1.5 equiv) by method A (R_f = 0.34, PE/EtOAc = 10:1). After purification by column chromatography (PE/EtOAc 30:1), **3ae** (166.5 mg, 90%) was obtained as colorless oil. ^{1}H NMR (500 MHz, $CDCl_3$) δ 7.49 (t, *J* = 1.7 Hz, 1H), 7.43 (dt, *J* = 7.9, 1.5 Hz, 1H), 7.30 (dt, *J* = 7.8, 1.4 Hz, 1H), 7.26 – 7.23 (m, 2H), 7.22 – 7.07 (m, 9H), 2.29 (s, 3H); ^{13}C NMR (125 MHz, $CDCl_3$) δ 168.9, 148.0, 137.7, 135.1, 134.3, 134.1, 132.0, 131.9, 131.0, 128.9, 128.9, 128.8, 128.7, 128.2, 127.5, 125.3, 118.3, 112.1, 20.9; HRMS (ESI-TOF) m/z: $[M+Na]^+$ calculated for $C_{23}H_{17}NNaO_2S$ 394.0878, found 394.0872.

31) methyl (*Z*)-4-(2-acetoxy-1-(naphthalen-2-ylthio)-2-phenylvinyl)benzoate (3lf)

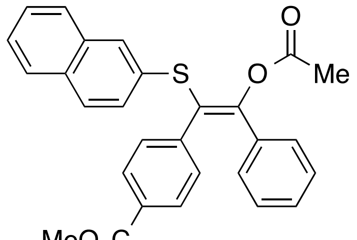

3lf

The general procedure was followed using **1l** (101.1 mg, 0.5 mmol, 1.0 equiv), $Rh_2(OAc)_4$ (4.4 mg, 0.01 mmol, 2.0 mol%) and **2f** (210.2 mg, 0.75 mmol, 1.5 equiv) by method A (R_f = 0.39, PE/EtOAc = 10:1). After purification by column chromatography (PE/EtOAc 30:1), **3lf** (183.2 mg, 81%) was obtained as colorless oil. ^{1}H NMR (500 MHz, $CDCl_3$) δ 7.74 (d, *J* = 1.2 Hz, 1H), 7.69 – 7.61 (m, 4H), 7.55 (d, *J* = 8.6 Hz, 1H), 7.40 – 7.33 (m, 4H), 7.31 (dd, *J* = 8.6, 1.8 Hz, 1H), 7.21 – 7.11 (m, 5H), 3.75 (s, 3H), 2.29 (s, 3H); ^{13}C NMR (125 MHz, $CDCl_3$) δ 169.0, 166.5, 148.0, 141.1, 134.7, 133.3, 132.1, 130.8, 130.2, 130.0, 129.1, 129.0, 128.9, 128.7, 128.6, 128.3, 128.1, 127.6, 127.2, 126.4, 126.2, 126.1, 51.9, 20.9; HRMS (ESI-TOF) m/z: $[M+Na]^+$ calculated for $C_{28}H_{22}NaO_2S$ 477.1136, found 477.1126.

32) (*Z*)-1-(4-fluorophenyl)-2-phenyl-2-(phenylthio)vinyl acetate (3ah)

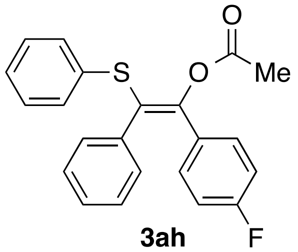

3ah

The general procedure was followed using **1a** (76.1 mg, 0.5 mmol, 1.0 equiv), $Rh_2(OAc)_4$ (4.4 mg, 0.01 mmol, 2.0 mol%) and **2h** (180.2 mg, 0.75 mmol, 1.5 equiv) by method A (R_f = 0.43, PE/EtOAc = 20:1). After purification by column chromatography (PE/EtOAc 30:1), **3ah** (171.8 mg, 94%) was obtained as white solid; m.p. = 106 – 108 ℃; ^{1}H NMR (500 MHz, $CDCl_3$) δ 7.28 – 7.23 (m, 2H), 7.22 – 7.17 (m, 2H), 7.16 – 7.11 (m, 2H), 7.11 – 6.98 (m, 6H), 6.88 – 6.75 (m, 2H), 2.26 (s, 3H); ^{13}C NMR (125 MHz, $CDCl_3$) δ 169.1, 163.2, 161.2, 145.1, 135.7, 132.7, 131.8, 131.3, 131.2, 130.8, 130.7, 130.6, 128.5, 127.9, 127.7, 127.4, 127.0, 115.1, 114.9, 20.9; HRMS (ESI-TOF) m/z: $[M+Na]^+$ calculated for $C_{22}H_{17}FNaO_2S$ 387.0831, found 387.0814.

33) (*Z*)-2-((4-bromophenyl)thio)-1,2-di(naphthalen-2-yl)vinyl acetate (3di)

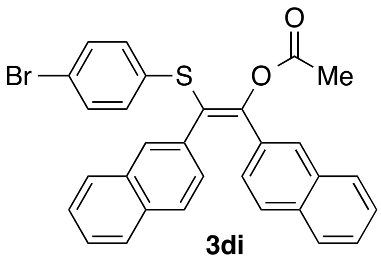

3di

The general procedure was followed using **1d** (115.6 mg, 0.5 mmol, 1.0 equiv), $Rh_2(OAc)_4$ (4.4 mg, 0.01 mmol, 2.0 mol%), **2i** (241.8 mg, 0.75 mmol, 1.5 equiv) and 4 mL DCM (due to poor solubility of **2i**) by method A (R_f = 0.35, PE/EtOAc = 20:1). After purification by column chromatography (PE/EtOAc 30:1), **3di** (222.8 mg, 85%) was obtained as yellow oil. ^{1}H NMR (500 MHz, $CDCl_3$) δ 7.28 (s, 1H), 7.78 (s, 1H), 7.68 – 7.61 (m, 3H), 7.58 (d, *J* = 7.9 Hz, 1H), 7.50 (d, *J* = 8.5 Hz, 1H), 7.45 (d, *J* = 8.6 Hz, 1H), 7.41 – 7.31 (m, 5H), 7.19 – 7.14 (m, 4H), 7.12 (dt, *J* = 8.6, 2.1 Hz, 1H), 2.34 (s, 3H); ^{13}C NMR (125 MHz, $CDCl_3$) δ 169.3, 147.8, 133.2, 132.93, 132.91, 132.8, 132.7, 132.6, 132.40, 132.36, 131.6, 130.4, 128.3, 128.1, 127.7, 127.51, 127.50, 127.48, 126.7, 126.6, 126.4, 126.2, 126.2, 126.0, 120.8, 21.0; HRMS (ESI-TOF) m/z: $[M+Na]^+$ calculated for $C_{30}H_{21}BrNaO_2S$ 547.0343, found 547.0328.

34) (*Z*)-1-(4-chlorophenyl)-1-(phenylthio)prop-1-en-2-yl acetate (3aj)

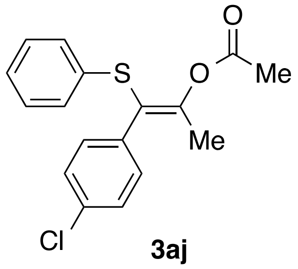

3aj

The general procedure was followed using **1a** (76.1 mg, 0.5 mmol, 1.0 equiv), $Rh_2(OAc)_4$ (4.4 mg, 0.01 mmol, 2.0 mol%) and **2j** (146.0 mg, 0.75 mmol, 1.5 equiv) by method A (R_f = 0.45, PE/EtOAc = 20:1). After purification by column chromatography (PE/EtOAc 30:1), **3aj** (142.3 mg, 89%) was obtained as colorless oil. ^{1}H NMR (500 MHz, $CDCl_3$) δ 7.25 – 7.21 (m, 2H), 7.20 – 7.14 (m, 4H), 7.13 – 7.05 (m, 3H), 2.23 (s, 3H), 1.98 (s, 3H); ^{13}C NMR (125 MHz, $CDCl_3$) δ 168.8, 148.2, 135.3, 133.4, 133.33, 131.27, 131.2, 128.6, 128.1, 126.8, 123.0, 20.9, 18.7; HRMS (ESI-TOF) m/z: $[M+Na]^+$ calculated for $C_{17}H_{15}ClNaO_2S$ 341.0379, found 341.0367.

35) (*Z*)-1-cyclopropyl-2-(2-fluorophenyl)-2-(phenylthio)vinyl acetate (3ak)

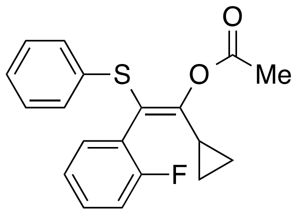

3ak

The general procedure was followed using **1a** (76.1 mg, 0.5 mmol, 1.0 equiv), $Rh_2(OAc)_4$ (4.4 mg, 0.01 mmol, 2.0 mol%) and **2k** (153.2 mg, 0.75 mmol, 1.5 equiv) by method A (R_f = 0.44, PE/EtOAc = 20:1). After purification by column chromatography (PE/EtOAc 30:1), **3ak** (153.1 mg, 93%) was obtained as colorless oil. ^{1}H NMR (500 MHz, $CDCl_3$) δ 7.38 (dt, *J* = 7.5, 1.8 Hz, 1H), 7.28 – 7.23 (m, 2H), 7.17 – 7.03 (m, 4H), 6.98 (dt, *J* = 7.5, 1.2 Hz, 1H), 6.96 – 6.89 (m, 1H), 2.21 (s, 3H), 1.64 – 1.57 (m, 1H), 0.85 – 0.54 (m, 4H); ^{13}C NMR (125 MHz, $CDCl_3$) δ 168.5, 161.0, 159.1, 152.5, 133.7, 132.3, 132.3, 130.8, 129.5, 129.4, 128.5, 126.7, 124.6, 124.5, 123.7, 123.7, 115.6, 115.5, 115.3, 20.6, 13.2, 13.1, 5.7; HRMS (ESI-TOF) m/z: $[M+Na]^+$ calculated for $C_{19}H_{17}FNaO_2S$ 351.0831, found 351.0826.

36) (*Z*)-1-phenyl-2-(phenylthio)prop-1-en-1-yl acetate (3al)

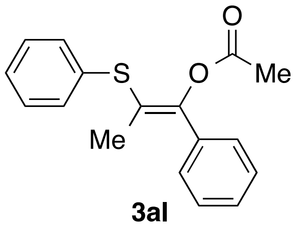

3al

The general procedure was followed using **1a** (76.1 mg, 0.5 mmol, 1.0 equiv), $Rh_2(OAc)_4$ (4.4 mg, 0.01 mmol, 2.0 mol%) and **2l** (120.1 mg, 0.75 mmol, 1.5 equiv) by method A (R_f = 0.71, PE/EtOAc = 10:1). After purification by column chromatography (PE/EtOAc 30:1), **3al** (78.5 mg, 55%) was obtained as colorless oil.^{1}H NMR (600 MHz, $CDCl_3$) δ 7.48 – 7.45 (m, 2H), 7.45 – 7.41 (m, 2H), 7.38 – 7.34 (m, 2H), 7.34 – 7.30 (m, 3H), 7.29 – 7.26 (m, 1H), 2.19 (s, 3H), 1.92 (s, 3H); ^{13}C NMR (150 MHz, $CDCl_3$) δ 169.0, 145.2, 134.9, 133.0, 132.4, 129.0, 128.7, 128.5, 128.2, 127.5, 121.7, 20.8, 19.0; HRMS (ESI-TOF) m/z: $[M+Na]^+$ calculated for $C_{17}H_{16}NaO_2S$ 307.0769, found 307.0763.

37) (*Z*)-1-phenyl-2-(phenylthio)vinyl acetate (3am)

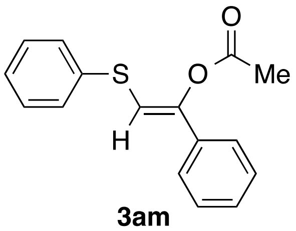

3am

The general procedure was followed using **1a** (76.1 mg, 0.5 mmol, 1.0 equiv), $Rh_2(OAc)_4$ (4.4 mg, 0.01 mmol, 2.0 mol%) and and **2m** (109.6 mg, 0.75 mmol, 1.5 equiv) by method A (R_f = 0.40, PE/EtOAc = 20:1). After purification by column chromatography (PE/EtOAc 50:1), **3am** (43.8 mg, 32%) was obtained as colorless oil. ^{1}H NMR (600 MHz, $CDCl_3$) δ 7.46 – 7.39 (m, 4H), 7.40 – 7.23 (m, 6H), 6.61 (s, 1H), 2.34 (s, 3H); ^{13}C NMR (150 MHz, $CDCl_3$) δ 168.0, 146.2, 134.7, 133.8, 130.0, 129.2, 128.6, 128.5, 127.2, 124.1, 113. 5, 20.5; HRMS (ESI-TOF) m/z: $[M+Na]^+$ calculated for $C_{16}H_{14}NaO_2S$ 293.0612, found 293.0604.

38) (*Z*)-1-((4-bromophenyl)thio)-1-(4-chlorophenyl)-4-phenylbut-1-en-3-yn-2-yl acetate (3dn)

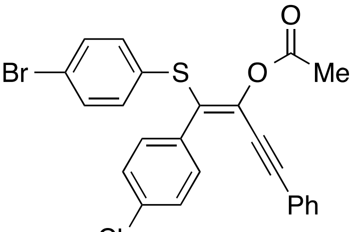

3dn

The general procedure was followed using **1d** (115.6 mg, 0.5 mmol, 1.0 equiv), $Rh_2(OAc)_4$ (4.4 mg, 0.01 mmol, 2.0 mol%) and **2n** (210.5 mg, 0.75 mmol, 1.5 equiv) by method A (R_f = 0.40, PE/EtOAc = 20:1). After purification by column chromatography (PE/EtOAc 30:1), **3dn** (198.1 mg, 82%) was obtained as white solid; m.p. = 134 – 136 ℃; ^{1}H NMR (500 MHz, $CDCl_3$) δ 7.58 (d, *J* = 8.5 Hz, 2H), 7.34 – 7.19 (m, 9H), 7.08 (d, *J* = 8.5 Hz, 2H), 2.28 (s, 3H); ^{13}C NMR (125 MHz, $CDCl_3$) δ 168.1, 134.5, 134.2, 133.3, 132.9, 131.9, 131.8, 131.6, 131.5, 130.3, 129.1, 128.3, 128.1, 121.6, 121.6, 94.3, 83.5, 20.7; HRMS (ESI-TOF) m/z: $[M+Na]^+$ calculated for $C_{24}H_{16}BrClNaO_2S$ 504.9641, found 504.9648.

39) (*Z*)-1-(4-chlorophenyl)-4-phenyl-1-(phenylthio)but-1-en-3-yn-2-yl acetate (3an)

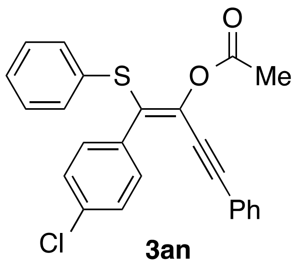

3an

The general procedure was followed using **1a** (76.1 mg, 0.5 mmol, 1.0 equiv), $Rh_2(OAc)_4$ (4.4 mg, 0.01 mmol, 2.0 mol%) and **2n** (210.5 mg, 0.75 mmol, 1.5 equiv) by method A (R_f = 0.39, PE/EtOAc = 20:1). After purification by column chromatography (PE/EtOAc 30:1), **3an** (174.3 mg, 86%) was obtained as light white solid; m.p. = 114 – 115 ℃; ^{1}H NMR (600 MHz, $CDCl_3$) δ 7.56 (d, *J* = 8.5 Hz, 2H), 7.31 – 7.25 (m, 5H), 7.24 – 7.21 (m, 2H), 7.19 (d, *J* = 8.5 Hz, 2H), 7.15 – 7.08 (m, 3H), 2.26 (s, 3H); ^{13}C NMR (150 MHz, $CDCl_3$) δ 168.1, 135.2, 134.3, 133.7, 132.5, 131.8, 131.7, 131.5, 129.6, 129.0, 128.8, 128.3, 127.9, 127.4, 121.8, 94.0, 83.7, 20.7; HRMS (ESI-TOF) m/z: $[M+Na]^+$ calculated for $C_{24}H_{17}ClNaO_2S$ 427.0535, found 427.0522.

40) (*Z*)-1-(4-bromophenyl)-4-phenyl-1-(phenylthio)but-1-en-3-yn-2-yl acetate (3ao)

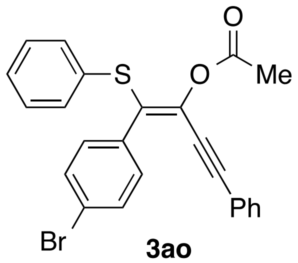

3ao

The general procedure was followed using **1a** (76.1 mg, 0.5 mmol, 1.0 equiv), $Rh_2(OAc)_4$ (4.4 mg, 0.01 mmol, 2.0 mol%) and **2o** (243.9 mg, 0.75 mmol, 1.5 equiv) by method A (R_f = 0.40, PE/EtOAc = 20:1). After purification by column chromatography (PE/EtOAc 30:1), **3ao** (189.2 mg, 84%) was obtained as light yellow solid; m.p. = 93 – 97 ℃; ^{1}H NMR (500 MHz, $CDCl_3$) δ 7.51 (d, *J* = 8.4 Hz, 2H), 7.35 (d, *J* = 8.5 Hz, 2H), 7.32 – 7.25 (m, 5H), 7.24 – 7.20 (m, 2H), 7.16 – 7.08 (m, 3H), 2.27 (s, 3H); ^{13}C NMR (125 MHz, $CDCl_3$) δ 168.2, 135.1, 134.1, 132.4, 132.0, 131.7, 131.5, 130.8, 129.6, 129.0, 128.8, 128.3, 127.4, 122.6, 121.7, 94.0, 83.6, 20.7; HRMS (ESI-TOF) m/z: $[M+Na]^+$ calculated for $C_{24}H_{17}BrNaO_2S$ 471.0030, found 471.0028.

41) (*Z*)-1-methoxy-2-phenyl-2-(phenylthio)vinyl acetate (3ap)

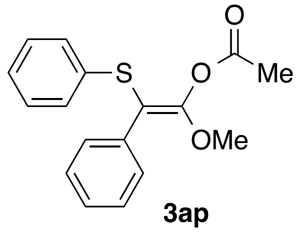

3ap

The general procedure was followed using **1a** (76.1 mg, 0.5 mmol, 1.0 equiv), $Rh_2(OAc)_4$ (4.4 mg, 0.01 mmol, 2.0 mol%) and **2p** (132.1 mg, 0.75 mmol, 1.5 equiv) by method A (R_f = 0.28, PE/EtOAc = 20:1). After purification by column chromatography (PE/EtOAc 30:1), **3ap** (93.2 mg, 62%) was obtained as colorless oil. ^{1}H NMR (600 MHz, $CDCl_3$) δ 7.61 (d, *J* = 7.6 Hz, 2H), 7.27 – 7.19 (m, 4H), 7.18 – 7.08 (m, 3H), 7.02 (t, *J* = 7.4 Hz, 1H), 3.66 (s, 3H), 2.24 (s, 3H); ^{13}C NMR (150 MHz, $CDCl_3$) δ 168.1, 154.2, 135.8, 135.5, 129.3, 128.6, 127.8, 127.7, 126.9, 125.5, 100.8, 57.0, 20.3; HRMS (ESI-TOF) m/z: $[M+Na]^+$ calculated for $C_{17}H_{16}NaO_3S$ 323.0718, found 323.0704.

42) (*Z*)-1-ethoxy-2-phenyl-2-(phenylthio)vinyl acetate (3aq)

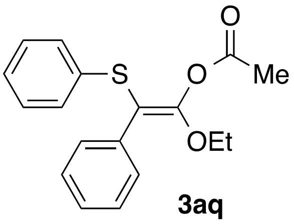

3aq

The general procedure was followed using **1a** (76.1 mg, 0.5 mmol, 1.0 equiv), $Rh_2(OAc)_4$ (4.4 mg, 0.01 mmol, 2.0 mol%) and **2q** (142.7 mg, 0.75 mmol, 1.5 equiv) by method A (R_f = 0.39, PE/EtOAc = 20:1). After purification by column chromatography (PE/EtOAc 30:1), **3aq** (102.2 mg, 66%) was obtained as colorless oil. ^{1}H NMR (500 MHz, $CDCl_3$) δ 7.64 (d, *J* = 7.9 Hz, 2H), 7.26 – 7.19 (m, 4H), 7.17 – 7.09 (m, 3H), 7.03 (t, *J* = 7.3 Hz, 1H), 3.97 (q, *J* = 7.1 Hz, 2H), 2.24 (s, 3H), 1.24 (t, *J* = 7.1 Hz, 2H); ^{13}C NMR (125 MHz, $CDCl_3$) δ 168.1, 153.7, 136.0, 135.6, 129.3, 128.6, 127.7, 127.7, 126.8, 125.5, 101.6, 66.5, 20.4, 14.8; HRMS (ESI-TOF) m/z: $[M+Na]^+$ calculated for $C_{18}H_{18}NaO_3S$ 337.0874, found 337.0863.

43) (*Z*)-1-isopropoxy-2-phenyl-2-(phenylthio)vinyl acetate (3ar)

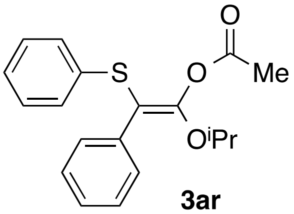

3ar

The general procedure was followed using **1a** (76.1 mg, 0.5 mmol, 1.0 equiv), $Rh_2(OAc)_4$ (4.4 mg, 0.01 mmol, 2.0 mol%) and **2r** (153.2 mg, 0.75 mmol, 1.5 equiv) by method A (R_f = 0.38, PE/EtOAc = 20:1). After purification by column chromatography (PE/EtOAc 30:1), **3ar** (128.9 mg, 78%) was obtained as light yellow oil. ^{1}H NMR (600 MHz, $CDCl_3$) δ 7.65 (d, *J* = 7.7 Hz, 2H), 7.24 – 7.18 (m, 4H), 7.16 – 7.09 (m, 3H), 7.03 (t, *J* = 7.3 Hz, 1H), 4.36 (sept, *J* = 6.1 Hz, 1H), 2.24 (s, 3H), 1.22 (d, *J* = 6.1Hz, 6H); ^{13}C NMR (150 MHz, $CDCl_3$) δ 167.9, 152.7, 136.0, 135.7, 129.5, 128.6, 127.9, 127.6, 126.7, 125.5, 102.9, 74.2, 22.3, 20.6; HRMS (ESI-TOF) m/z: $[M+Na]^+$ calculated for $C_{19}H_{20}NaO_3S$ 351.1031, found 351.1019.

44) (*Z*)-2-(4-bromophenyl)-1-methoxy-2-(phenylthio)vinyl acetate (3as)

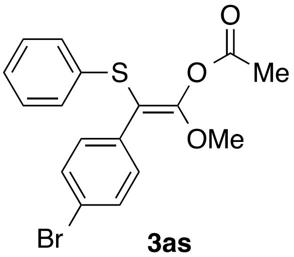

3as

The general procedure was followed using **1a** (76.1 mg, 0.5 mmol, 1.0 equiv), $Rh_2(OAc)_4$ (4.4 mg, 0.01 mmol, 2.0 mol%) and **2s** (191.3 mg, 0.75 mmol, 1.5 equiv) by method A (R_f = 0.54, PE/EtOAc = 20:1). After purification by column chromatography (PE/EtOAc 30:1), **3as** (125.7 mg, 66%) was obtained as white solid; m.p. = 74 – 75 ℃; ^{1}H NMR (600 MHz, $CDCl_3$) δ 7.50 (d, *J* = 8.7 Hz, 2H), 7.34 (d, *J* = 8.7 Hz, 2H), 7.21 – 7.17 (m, 2H), 7.16 – 7.12 (m, 2H), 7.05 (t, *J* = 7.2 Hz, 1H), 3.69 (s, 3H), 2.27 (s, 3H); ^{13}C NMR (150 MHz, $CDCl_3$) δ 167.9, 154.3, 135.4, 134.6, 130.9, 128.7, 127.8, 125.8, 120.7, 99.8, 56.9, 20.3; HRMS (ESI-TOF) m/z: $[M+Na]^+$ calculated for $C_{17}H_{15}BrNaO_3S$ 400.9823, found 400.9826.

45) (*Z*)-2-(2-ethynylphenyl)-1-methoxy-2-(phenylthio)vinyl acetate (3at)

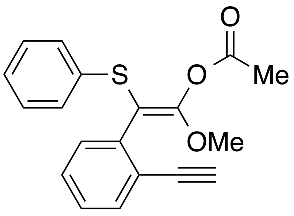

3at

The general procedure was followed using **1a** (76.1 mg, 0.5 mmol, 1.0 equiv), $Rh_2(OAc)_4$ (4.4 mg, 0.01 mmol, 2.0 mol%) and **2t** (150.1 mg, 0.75 mmol, 1.5 equiv) by method A (R_f = 0.54, PE/EtOAc = 20:1). After purification by column chromatography (PE/EtOAc 30:1), **3at** (132.7 mg, 82%) was obtained as yellow oil. ^{1}H NMR (600 MHz, $CDCl_3$) δ 7.48 (d, *J* = 7.7 Hz, 1H), 7.44 (d, *J* = 7.8 Hz, 1H), 7.40 (d, *J* = 7.8 Hz, 2H), 7.24 (t, *J* = 7.7 Hz, 1H), 7.21 (t, *J* = 7.7 Hz, 2H), 7.15 (t, *J* = 7.6 Hz, 1H), 7.11 (t, *J* = 7.4 Hz, 1H), 3.69 (s, 3H), 3.36 (s, 1H), 2.31 (s, 3H); ^{13}C NMR (150 MHz, $CDCl_3$) δ 167.7, 154.2, 139.1, 135.1, 132.9, 130.1, 129.0, 128.5, 128.4, 127.0, 125.9, 122.4, 98.4, 83.0, 80.7, 56.5, 20.3; HRMS (ESI-TOF) m/z: $[M+Na]^+$ calculated for $C_{19}H_{16}NaO_3S$ 347.0718, found 347.0711.

46) (*Z*)-1-methoxy-2-(phenylthio)-2-(4-(trifluoromethyl)phenyl)vinyl acetate (3au)

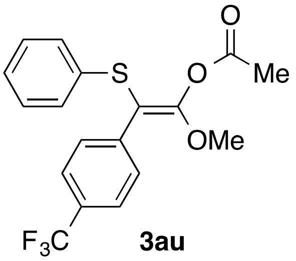

3au

The general procedure was followed using **1a** (76.1 mg, 0.5 mmol, 1.0 equiv), $Rh_2(OAc)_4$ (4.4 mg, 0.01 mmol, 2.0 mol%) and **2u** (183.1 mg, 0.75 mmol, 1.5 equiv) by method A (R_f = 0.37, PE/EtOAc = 10:1). After purification by column chromatography (PE/EtOAc 20:1), **3au** (117.3 mg, 64%) was obtained as colorelss oil. ^{1}H NMR (600 MHz, $CDCl_3$) δ 7.75 (d, *J* = 8.2 Hz, 2H), 7.48 (d, *J* = 8.1 Hz, 2H), 7.22 – 7.12 (m, 4H), 7.06 (t, *J* = 7.2 Hz, 1H), 3.72 (s, 3H), 2.29 (s, 3H); ^{13}C NMR (150 MHz, $CDCl_3$) δ 167.8, 155.1, 139.5, 135.4, 129.5, 128.8, 128.6 (q, *J* = 32.6 Hz), 127.6, 125.8, 124.7 (q, *J* = 4.2 Hz), 124.1 (q, *J* = 272.1 Hz), 99.6, 56.8, 20.3; HRMS (ESI-TOF) m/z: $[M+Na]^+$ calculated for $C_{18}H_{15}F_3NaO_3S$ 391.0592, found 391.0588.

47) (*Z*)-1-(*tert*-butoxy)-2-(phenylthio)-2-(4-(trifluoromethyl)phenyl)vinyl acetate (3av)

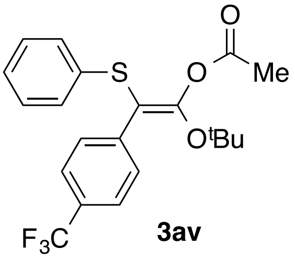

3av

The general procedure was followed using **1a** (76.1 mg, 0.5 mmol, 1.0 equiv), $Rh_2(OAc)_4$ (4.4 mg, 0.01 mmol, 2.0 mol%) and **2v** (214.7 mg, 0.75 mmol, 1.5 equiv) by method A (R_f = 0.39, PE/EtOAc = 10:1). After purification by column chromatography (PE/EtOAc 30:1), **3av** (165.9 mg, 81%) was obtained as white solid. m.p. = 133 – 136 ℃; ^{1}H NMR (500 MHz, $CDCl_3$) δ 7.79 (d, *J* = 8.1 Hz, 2H), 7.44 (d, *J* = 8.2 Hz, 2H), 7.25 – 7.19 (m, 2H), 7.13 (t, *J* = 7.6 Hz, 2H), 7.05 (t, *J* = 7.4 Hz, 1H), 2.23 (s, 3H), 1.28 (s, 9H); ^{13}C NMR (150 MHz, $CDCl_3$) δ 167.8, 152.5, 140.0, 135.0, 130.0, 128.7, 128.6 (q, *J* = 32.3 Hz), 128.5, 126.0, 124.4 (q, *J* = 3.8 Hz), 124.2 (q, *J* = 272.0 Hz), 107.2, 84.8, 28.8, 21.0; HRMS (ESI-TOF) m/z: $[M+Na]^+$ calculated for $C_{21}H_{21}F_3NaO_3S$ 433.1061, found 433.1063.

48) (*Z*)-2-phenyl-2-(phenylthio)-1-(2,2,2-trifluoroethoxy)vinyl acetate (3aw)

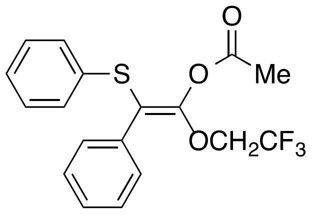

3aw

The general procedure was followed using **1a** (76.1 mg, 0.5 mmol, 1.0 equiv), $Rh_2(OAc)_4$ (4.4 mg, 0.01 mmol, 2.0 mol%) and **2w** (183.1 mg, 0.75 mmol, 1.5 equiv) by method A (R_f = 0.54, PE/EtOAc = 20:1). After purification by column chromatography (PE/EtOAc 30:1), **3aw** (144.2 mg, 78%) was obtained as white solid. m.p. = 32 – 34 ℃; ^{1}H NMR (600 MHz, $CDCl_3$) δ 7.55 (d, *J* = 7.3 Hz, 2H), 7.26 – 7.20 (m, 4H), 7.19 – 7.10 (m, 3H), 7.06 (t, *J* = 7.3 Hz, 1H), 4.13 (q, *J* = 8.2 Hz, 2H), 2.28 (s, 3H); ^{13}C NMR (151 MHz, $CDCl_3$) δ 167.9, 150.8, 134.4, 134.2, 129.5, 129.0, 128.7, 128.0, 127.7, 126.2, 122.6 (q, *J* = 278.3 Hz), 107.0, 66.4 (q, *J* = 36.3 Hz), 20.3; HRMS (ESI-TOF) m/z: $[M+Na]^+$ calculated for $C_{18}H_{15}F_3NaO_3S$ 391.0592, found 391.0576.

49) 9,10-diphenyl-8-oxa-11-thiaspiro[5.6]dodec-9-en-7-one (11a)

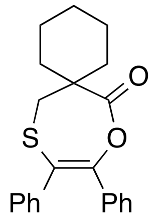

11a

The general procedure was followed using **10a** (78.1 mg, 0.5 mmol, 1.0 equiv), $Rh_2(OAc)_4$ (4.4 mg, 0.01 mmol, 2.0 mol%) and **2a** (166.7 mg, 0.75 mmol, 1.5 equiv) by method A (R_f = 0.57, PE/EtOAc = 20:1). After purification by column chromatography (PE/EtOAc 30:1), **11a** (128.4 mg, 73%) was obtained as colorless oil. ^{1}H NMR (600 MHz, $CDCl_3$) δ 7.31 – 7.27 (m, 2H), 7.24 – 7.21 (m, 2H), 7.20 – 7.16 (m, 3H), 7.16 – 7.11 (m, 3H), 3.06 (s, 3H), 2.26 – 2.17 (m, 2H), 1.73 – 1.65 (m, 2H), 1.64 – 1.55 (m, 5H), 1.45 – 1.37 (m, 1H); ^{13}C NMR (150 MHz, $CDCl_3$) δ 175.4, 149.1, 136.7, 135.0, 131.0, 129.0, 128.3, 128.0, 127.8, 127.7, 117.7, 54.0, 40.4, 33.2, 25.6, 22.8; HRMS (ESI-TOF) m/z: $[M+Na]^+$ calculated for $C_{22}H_{22}NaO_2S$ 373.1238, found 373.1218.

50) 6-benzyl-2,3-diphenyl-5,6-dihydro-7*H*-1,4-oxathiepin-7-one (11b)

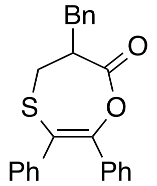

11b

The general procedure was followed using **10b** (71.3 mg, 0.4 mmol, 1.0 equiv), $Rh_2(OAc)_4$ (3.5 mg, 0.008 mmol, 2.0 mol%) and **2a** (133.3 mg, 0.6 mmol, 1.5 equiv) by method A (R_f = 0.50, PE/EtOAc = 10:1). After purification by column chromatography (PE/EtOAc 20:1), **11b** (77.2 mg, 52%) was obtained as white solid; m.p. = 192 – 193 ℃; ^{1}H NMR (600 MHz, $CDCl_3$) δ 7.28 (t, *J* = 7.5 Hz, 2H), 7.25 – 7.10 (m, 13H), 3.64 – 3.53 (m, 2H), 3.26 (dd, *J* = 14.4, 7.2 Hz, 1H), 3.07 (t, *J* = 10.6 Hz, 1H), 2.86 (dd, *J* = 14.5, 6.7 Hz, 1H); ^{13}C NMR (150 MHz, $CDCl_3$) δ 171.6, 152.0, 138.7, 137.8, 133.3, 130.0, 129.1, 128.9, 128.7, 128.4, 128.1, 128.0, 126.8, 119.3, 45.8, 39.8, 36.8; HRMS (ESI-TOF) m/z: $[M+Na]^+$ calculated for $C_{24}H_{20}NaO_2S$ 395.1082, found 395.1069.

51) (*Z*)-*N*-(8-oxo-2,3-diphenyl-5,6,7,8-tetrahydro-1,4-oxathiocin-7-yl)acetamide (11c)

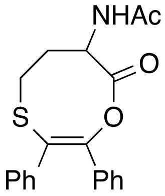

11c

The general procedure was followed using **10c** (79.6 mg, 0.5 mmol, 1.0 equiv), $Rh_2(OAc)_4$ (4.4 mg, 0.01 mmol, 2.0 mol%) and **2a** (166.7 mg, 0.75 mmol, 1.5 equiv) by method A (R_f = 0.25, PE/EtOAc = 1:1). After purification by column chromatography (PE/EtOAc 10:1), **11c** (141.3 mg, 80%) was obtained as light brown solid; m.p. = 187 – 189 ℃; ^{1}H NMR (600 MHz, $CDCl_3$) δ 7.38 – 7.33 (m, 2H), 7.27 – 7.11 (m, 8H), 6.45 (d, *J* = 7.8 Hz, 1H), 5.02 (dt, *J* = 12.0, 6.2 Hz, 1H), 3.16 (t, *J* = 13.4 Hz, 1H), 2.92 (dt, *J* = 15.1, 3.2 Hz, 1H), 2.63 – 2.54 (m, 1H), 2.11 (q, *J* = 12.9 Hz, 1H), 2.08 (s, 3H); ^{13}C NMR (150 MHz, $CDCl_3$) δ 173.4, 169.9, 147.7, 136.7, 133.4, 130.8, 128.8, 128.5, 128.4, 128.4, 127.9, 124.0, 49.8, 36.0, 30.1, 23.1; HRMS (ESI-TOF) m/z: $[M+Na]^+$ calculated for $C_{20}H_{19}NNaO_3S$ 376.0983, found 376.0967.

52) (*Z*)-2,3-diphenyl-5,6,7,8-tetrahydro-9*H*-1,4-oxathionin-9-one (11d)

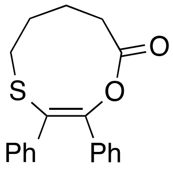

11d

The general procedure was followed using **10d** (46.5 mg, 0.4 mmol, 1.0 equiv), $Rh_2(OAc)_4$ (3.5 mg, 0.008 mmol, 2.0 mol%) and **2a** (133.3 mg, 0.6 mmol, 1.5 equiv) by method A (R_f = 0.48, PE/EtOAc = 10:1). After purification by column chromatography (PE/EtOAc 20:1), **11d** (99.6 mg, 80%) was obtained as white solid; m.p. = 151 – 152 ℃; ^{1}H NMR (500 MHz, $CDCl_3$) δ 7.41 – 7.35 (m, 2H), 7.23 – 7.18 (m, 3H), 7.16 – 7.09 (m, 5H), 3.04 (t, *J* = 6.1 Hz, 2H), 2.69 – 2.64 (m, 2H), 2.16 – 2.08 (m, 2H), 2.02 – 1.96 (m, 2H); ^{13}C NMR (125 MHz, $CDCl_3$) δ 173.6, 146.5, 137.3, 135.2, 130.9, 128.3, 128.2, 128.1, 127.9, 127.9, 35.1, 34.4, 30.5, 23.9; HRMS (ESI-TOF) m/z: $[M+Na]^+$ calculated for $C_{19}H_{18}NaO_2S$ 333.0925, found 333.0922.

53) (*Z*)-3-(4-chlorophenyl)-2-phenyl-5,6,7,8-tetrahydro-9*H*-1,4-oxathionin-9-one (11e)

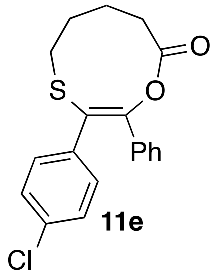

11e

The general procedure was followed using **10d** (58.1 mg, 0.5 mmol, 1.0 equiv), $Rh_2(OAc)_4$ (4.4 mg, 0.01 mmol, 2.0 mol%) and **2b** (192.5 mg, 0.75 mmol, 1.5 equiv) by method A (R_f = 0.51, PE/EtOAc = 10:1). After purification by column chromatography (PE/EtOAc 15:1), **11e** (134.7 mg, 78%) was obtained as white solid; m.p. = 146 – 148 ℃; ^{1}H NMR (500 MHz, $CDCl_3$) δ 7.31 (d, *J* = 8.5 Hz, 2H), 7.21 – 7.08 (m, 7H), 3.05 (t, *J* = 6.1 Hz, 2H), 2.69 – 2.61 (m, 2H), 2.16 – 2.08 (m, 2H), 2.02 – 1.96 (m, 2H); ^{13}C NMR (125 MHz, $CDCl_3$) δ 173.4, 146.8, 135.9, 134.9, 133.9, 132.3, 128.5, 128.3, 128.2, 128.1, 127.0, 35.1, 34.5, 30.4, 23.9; HRMS (ESI-TOF) m/z: $[M+Na]^+$ calculated for $C_{19}H_{17}ClNaO_2S$ 367.0535, found 367.0529.

54) (*Z*)-3-(4-chlorophenyl)-2-methyl-5,6,7,8-tetrahydro-9*H*-1,4-oxathionin-9-one (11f)

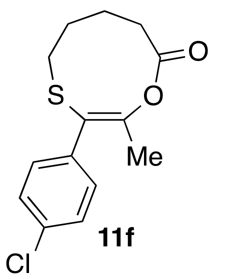

11f

The general procedure was followed using **10d** (58.1 mg, 0.5 mmol, 1.0 equiv), $Rh_2(OAc)_4$ (4.4 mg, 0.01 mmol, 2.0 mol%) and **2j** (146.0 mg, 0.75 mmol, 1.5 equiv) by method A (R_f = 0.45, PE/EtOAc = 20:1). After purification by column chromatography (PE/EtOAc 20:1), **11f** (105.1 mg, 74%) was obtained as colorless oil. ^{1}H NMR (600 MHz, $CDCl_3$) δ 7.40 – 7.25 (m, 4H), 2.89 (q, *J* = 5.9 Hz, 2H), 2.60 – 2.52 (m, 2H), 2.13 – 2.06 (m, 2H), 2.08 (s, 3H), 1.99 – 1.89 (m, 2H); ^{13}C NMR (150 MHz, $CDCl_3$) δ 173.7, 148.3, 136.3, 133.6, 131.0, 128.4, 123.4, 35.2, 34.1, 30.3, 23.3, 18.9; HRMS (ESI-TOF) m/z: $[M+Na]^+$ calculated for $C_{14}H_{15}ClNaO_2S$ 305.0379, found 305.0378.

55) 2,3,4,5-tetrahydro-6*H*-naphtho[2,1-*b*][1,4]oxathionin-6-one (11g)

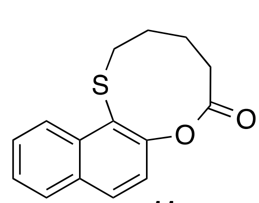

11g

The general procedure was followed using **10d** (58.1 mg, 0.5 mmol, 1.0 equiv), $Rh_2(OAc)_4$ (4.4 mg, 0.01 mmol, 2.0 mol%) and **2y** (127.6 mg, 0.75 mmol, 1.5 equiv) by method A (R_f = 0.54, PE/EtOAc = 10:1). After purification by column chromatography (PE/EtOAc 10:1), **11g** (67.0 mg, 52%) was obtained as light brown solid; m.p. = 93 – 95 ℃; ^{1}H NMR (600 MHz, $CDCl_3$) δ 8.54 (d, *J* = 8.5 Hz, 1H), 7.84 (d, *J* = 8.1 Hz, 1H), 7.82 (d, *J* = 8.9 Hz, 1H), 7.56 (t, *J* = 7.7 Hz, 1H), 7.48 (t, *J* = 7.5 Hz, 1H), 7.36 (d, *J* = 8.6 Hz, 1H), 3.01 (t, *J* = 6.2 Hz, 2H), 2.73 – 2.67 (m, 2H), 2.33 – 2.26 (m, 2H), 2.07 – 2.00 (m, 2H); ^{13}C NMR (150 MHz, $CDCl_3$) δ 173.5, 149.7, 134.7, 131.9, 128.9, 128.3, 126.9, 126.4, 125.9, 125.8, 122.2, 35.1, 35.0, 30.0, 23.1; HRMS (ESI-TOF) m/z: $[M+Na]^+$ calculated for $C_{15}H_{14}NaO_2S$ 281.0612, found 281.0614.

56) (*Z*)-4,5-diphenyl-1,7-dihydro-3*H*-benzo[*g*][1,5]oxathionin-3-one (11h)

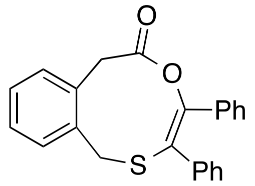

11h

The general procedure was followed using **10e** (82.1 mg, 0.5 mmol, 1.0 equiv), $Rh_2(OAc)_4$ (4.4 mg, 0.01 mmol, 2.0 mol%) and **2a** (166.7 mg, 0.75 mmol, 1.5 equiv) by method A (R_f = 0.42, PE/EtOAc = 10:1). After purification by column chromatography (PE/EtOAc 20:1), **11h** (111.2 mg, 62%) was obtained as white solid; m.p. = 188 – 190 ℃; ^{1}H NMR (500 MHz, $CDCl_3$) δ 7.36 – 7.25 (m, 6H), 7.23 – 7.06 (m, 8H), 3.95 (s, 2H), 3.91 (s, 2H); ^{13}C NMR (125 MHz, $CDCl_3$) δ 171.3, 147.1, 139.2, 136.7, 134.6, 132.6, 131.5, 131.0, 130.5, 128.4, 128.3, 128.3, 128.2, 128.0, 128.0, 127.7, 124.7, 39.1, 36.2; HRMS (ESI-TOF) m/z: $[M+Na]^+$ calculated for $C_{23}H_{18}NaO_2S$ 381.0925, found 381.0923.

57) (*Z*)-2,3-diphenyl-6,7,8,9-tetrahydro-1,4-oxathiecin-10(5*H*)-one (11i)

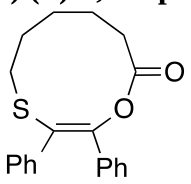

11i

The general procedure was followed using **10f** (52.0 mg, 0.4 mmol, 1.0 equiv), $Rh_2(OAc)_4$ (3.5 mg, 0.008 mmol, 2.0 mol%) and **2a** (133.3 mg, 0.6 mmol, 1.5 equiv) by method A (R_f = 0.48, PE/EtOAc = 10:1). After purification by column chromatography (PE/EtOAc 30:1), **11i** (69.1 mg, 53%) was obtained as white solid; m.p. = 132 – 134 ℃; ^{1}H NMR (600 MHz, $CDCl_3$) δ 7.33 – 7.29 (m, 2H), 7.22 – 7.18 (m, 3H), 7.13 – 7.04 (m, 5H), 3.05 (t, *J* = 6.4 Hz, 2H), 2.63 – 2.59 (m, 2H), 1.97 – 1.92 (m, 2H), 1.84 – 1.76 (m, 4H); ^{13}C NMR (150 MHz, $CDCl_3$) δ 172.6, 145.3, 137.9, 135.9, 130.5, 128.6, 128.3, 127.9, 127.8, 127.7, 126.9, 34.0, 32.2, 28.4, 24. 5, 22.2; HRMS (ESI-TOF) m/z: $[M+Na]^+$ calculated for $C_{20}H_{20}NaO_2S$ 347.1082, found 347.1081.

58) (3*S*,8*S*,9*S*,10*R*,13*R*,14*S*,17*R*)-10,13-dimethyl-17-((*R*)-6-methylheptan-2-yl)-2,3,4,7,8,9,10,11,12, 13,14,15,16,17-tetradecahydro-1*H*-cyclopenta[*a*]phenanthren-3-yl 3-(((*Z*)-2-acetoxy-1,2-diphenylvinyl)thio)propanoate (4)

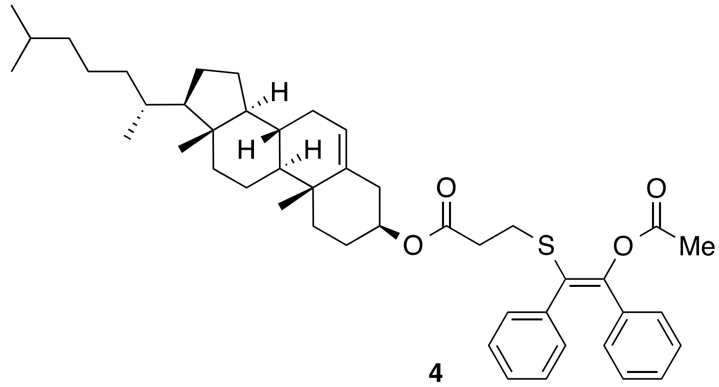

4

The general procedure was followed using **1aa** (258.4 mg, 0.5 mmol, 1.0 equiv), $Rh_2(OAc)_4$ (4.4 mg, 0.01 mmol, 2.0 mol%) and **2a** (166.7 mg, 0.75 mmol, 1.5 equiv) by method A (R_f = 0.20, PE/EtOAc = 20:1). After purification by column chromatography (PE/EtOAc 20:1), **4** (101.4 mg, 29 %) was obtained as colorless oil. ^{1}H NMR (600 MHz, $CDCl_3$) δ 7.35 – 7.30 (m, 2H), 7.27 – 7.22 (m, 3H), 7.14 – 7.05 (m, 5H), 5.36 (d, *J* = 4.6 Hz, 1H), 4.62 – 4.51 (m, 1H), 2.55 (t, *J* = 7.3 Hz, 2H), 2.39 (t, *J* = 7.3Hz, 2H), 2.32 – 2.26 (m, 2H), 2.27 (s, 3H), 2.03 – 1.94 (m, 2H), 1.87 – 1.77 (m, 3H), 1.59 – 1.22 (m, 12H), 1.19 – 1.04 (m, 7H), 1.01 (s, 3H), 1.00 – 0.93 (m, 2H), 0.91 (d, *J* = 6.5 Hz, 3H), 0.86 (dd, *J* = 6.7, 2.7 Hz, 6H), 0.68 (s, 3H); ^{13}C NMR (150 MHz, $CDCl_3$) δ 171.0, 168.9, 145.4, 139.6, 135.4, 135.1, 130.6, 128.6, 128.5, 128.0, 127.9, 127.8, 127.0, 122.6, 74.2, 56.7, 56.1, 50.0, 42.3, 39.7, 39.5, 38.0, 36.9, 36.6, 36.2, 35.8, 35.2, 31.9, 31.8, 28.2, 28.0, 27.7, 26.3, 24.3, 23.8, 22.8, 22.5, 21.0, 20.9, 19.3, 18.7, 11.8; HRMS (ESI-TOF) m/z: $[M+Na]^+$ calculated for $C_{46}H_{62}NaO_4S$ 733.4267, found 733.4251.

59) (8*R*,9*S*,13*S*,14*S*,17*S*)-17-((3-(((*Z*)-2-acetoxy-1,2-diphenylvinyl)thio)propanoyl) oxy)-13-methyl-7,8,9,11,12,13,14,15,16,17-decahydro-6*H*-cyclopenta[*a*] phenanthren-3-yl benzoate (5)

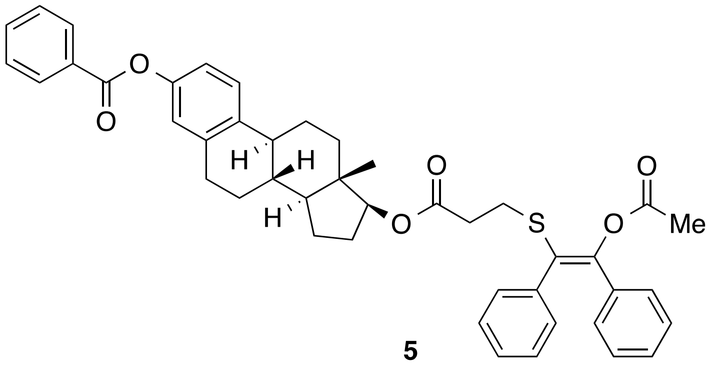

5

The general procedure was followed using **1bb** (253.3 mg, 0.5 mmol, 1.0 equiv), $Rh_2(OAc)_4$ (4.4 mg, 0.01 mmol, 2.0 mol%) and **2a** (189.0 mg, 0.85 mmol, 1.7 equiv) by method A (R_f = 0.35, PE/EtOAc = 20:1). After purification by column chromatography (PE/EtOAc 20:1), **5** (142.5 mg, 41 %) was obtained as colorless oil. ^{1}H NMR (500 MHz, $CDCl_3$) δ 8.20 (d, *J* = 8.4 Hz, 2H), 7.63 (t, *J* = 7.4 Hz, 1H), 7.51 (t, *J* = 7.6 Hz, 2H), 7.36 – 7.30 (m, 3H), 7.28 – 7.22 (m, 3H), 7.16 – 7.05 (m, 5H), 6.97 (d, *J* = 8.5 Hz, 1H), 6.93 (s, 1H), 4.66 (t, *J* = 8.4 Hz, 1H), 2.95 – 2.85 (m, 2H), 2.57 (t, *J* = 7.3 Hz, 2H), 2.43 (t, *J* = 7.3 Hz, 2H), 2.35 – 2.13 (m, 3H), 2.28 (s, 3H), 1.93 – 1.83 (m, 2H), 1.78 – 1.70 (m, 1H), 1.58 – 1.21(m, 8H), 0.79 (s, 3H); ^{13}C NMR (125 MHz, $CDCl_3$) δ 171.5, 168.9, 165.4, 148.7, 145.2, 138.2, 137.8, 135.4, 135.1, 133.5, 130.6, 130.1, 129.7, 128.6, 128.5, 128.5, 128.1, 127.9, 127.8, 127.0, 126.5, 121.6, 118.7, 82.8, 49.8, 44.0, 43.0, 38.2, 36.8, 35.2, 29.5, 27.5, 27.0, 26.5, 26.0, 23.3, 20.9, 12.1; HRMS (ESI-TOF) m/z: $[M+Na]^+$ calculated for $C_{44}H_{44}NaO_6S$ 723.2756, found 723.2750.

60) benzyl (*Z*)-(3-((2-acetoxy-1,2-diphenylvinyl)thio)-2-benzylpropanoyl)glycinate (6)

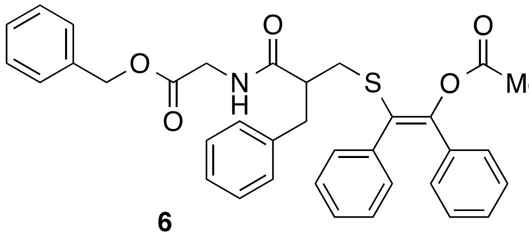

6

The general procedure was followed using Racecadotril (193.0 mg, 0.5 mmol, 1.0 equiv), $Rh_2(OAc)_4$ (4.4 mg, 0.01 mmol, 2.0 mol%) and **2a** (166.7 mg, 0.75 mmol, 1.5 equiv) by method A (R_f = 0.50, PE/EtOAc = 1:1). After purification by column chromatography (PE/EtOAc 5:1 to 2:1), **6** (175.8 mg, 61%) was obtained as colorless oil. ^{1}H NMR (500 MHz, $CDCl_3$) δ 7.38 – 7.30 (m, 5H), 7.28 – 7.24 (m, 2H), 7.22 – 7.03 (m, 11H), 7.01 – 6.96 (m, 2H), 6.04 (t, *J* = 5.3 Hz, 1H), 5.15 (s, 2H), 4.02 – 3.83 (m, 2H), 2.80 – 2.66 (m, 2H), 2.48 – 2.39 (m, 1H), 2.38 – 2.28 (m, 2H), 2.19 (s, 3H); ^{13}C NMR (125 MHz, $CDCl_3$) δ 173.4, 169.7, 169.2, 145.1, 138.7, 135.2, 135.1, 134.8, 130.4, 128.7, 128.6, 128.5, 128.5, 128.4, 128.3, 128.3, 128.1, 127.9, 127.8, 127.4, 126.2, 66.9, 50.2, 41.4, 38.2, 33.3, 20.9; HRMS (ESI-TOF) m/z: $[M+Na]^+$ calculated for $C_{35}H_{33}NNaO_5S$ 602.1977, found 602.1973.

61) (*Z*)-2-((4-bromophenyl)thio)-1,2-diphenylvinyl 5-(2,5-dimethylphenoxy)-2,2-dimethylpentanoate (7)

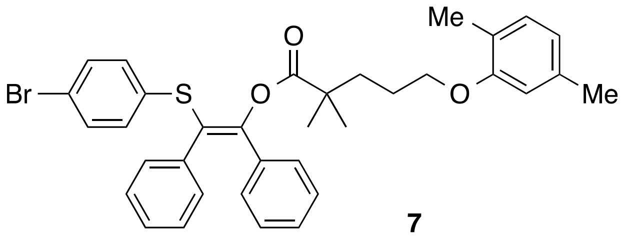

7

The general procedure was followed using **1cc** (210.7 mg, 0.5 mmol, 1.0 equiv), $Rh_2(OAc)_4$ (4.4 mg, 0.01 mmol, 2.0 mol%) and **2a** (166.7 mg, 0.75 mmol, 1.5 equiv) by method A (R_f = 0.56, PE/EtOAc = 20:1). After purification by column chromatography (PE/EtOAc 30:1), **7** (180.2 mg, 59%) was obtained as colorless oil. ^{1}H NMR (600 MHz, $CDCl_3$) δ 7.22 – 7.02 (m, 14H), 6.99 (d, *J* = 7.4 Hz, 1H), 6.65 (d, *J* = 7.4 Hz, 1H), 6.57 (s, 1H), 3.89 (t, *J* = 5.8 Hz, 2H), 2.29 (s, 3H), 2.15 (s, 3H), 1.91 – 1.81 (m, 4H), 1.36 (s, 6H); ^{13}C NMR (150 MHz, $CDCl_3$) δ 175.8, 157.0, 147.6, 136.4, 135.9, 135.1, 132.6, 131.5, 130.7, 130.2, 128.8, 128.4, 128.0, 127.9, 127.7, 126.1, 123.6, 120.7, 120.7, 111.9, 68.0, 42.5, 36.9, 25.1, 21.4, 15.8; HRMS (ESI-TOF) m/z: $[M+Na]^+$ calculated for $C_{35}H_{35}BrNaO_3S$ 637.1388, found 637.1373.

62) (*Z*)-2-((4-bromophenyl)thio)-1,2-diphenylvinyl 2-acetoxybenzoate (8)

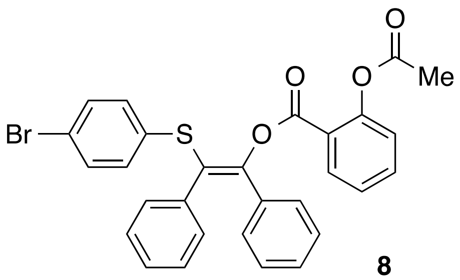

8

The general procedure was followed using **1dd** (175.6 mg, 0.5 mmol, 1.0 equiv), $Rh_2(OAc)_4$ (4.4 mg, 0.01 mmol, 2.0 mol%) and **2a** (166.7 mg, 0.75 mmol, 1.5 equiv) by method A (R_f = 0.30, PE/EtOAc = 10:1). After purification by column chromatography (PE/EtOAc 10:1), **8** (174.7 mg, 64%) was obtained as colorless oil. ^{1}H NMR (600 MHz, $CDCl_3$) δ 8.19 (dd, *J* = 7.9, 1.7 Hz, 1H), 7.60 (td, *J* = 7.8, 1.8 Hz, 1H), 7.35 (t, *J* = 7.4 Hz, 1H), 7.29 – 7.26 (m, 2H), 7.21 – 7.06 (m, 13H), 2.25 (s, 3H); ^{13}C NMR (150 MHz, $CDCl_3$) δ 169.6, 162.5, 151.1, 146.5, 135.7, 134.5, 134.4, 133.0, 132.4, 132.2, 131.5, 130.8, 128.8, 128.4, 128.1, 128.0, 127.9, 127.1, 126.1, 124.0, 122.6, 121.0, 21.0; HRMS (ESI-TOF) m/z: $[M+Na]^+$ calculated for $C_{29}H_{21}BrNaO_4S$ 567.0242, found 567.0240.

63) (*Z*)-2-(butylthio)-1,2-diphenylvinyl 2-(1-(4-chlorobenzoyl)-5-methoxy-2-methyl-1*H*-indol-3-yl)acetate (9)

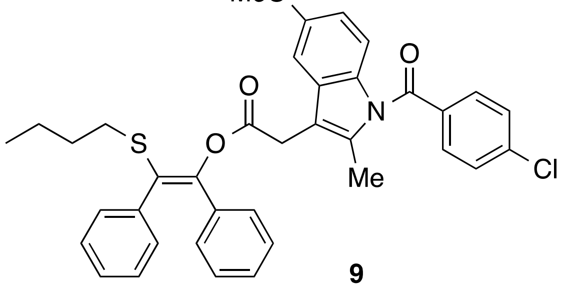

9

The general procedure was followed using **1ee** (215.0 mg, 0.5 mmol, 1.0 equiv), $Rh_2(OAc)_4$ (4.4 mg, 0.01 mmol, 2.0 mol%) and **2a** (166.7 mg, 0.75 mmol, 1.5 equiv) by method A (R_f = 0.36, PE/EtOAc = 10:1). After purification by column chromatography (PE/EtOAc 20:1 to 10:1), **9** (161.2 mg, 52%) was obtained as yellow oil ^{1}H NMR (500 MHz, $CDCl_3$) δ 7.66 (d, *J* = 8.5 Hz, 2H), 7.46 (d, *J* = 8.5 Hz, 2H), 7.30 – 7.26 (m, 2H), 7.25

– 7.20 (m, 2H), 7.09 – 7.05 (m, 1H), 7.04 – 6.99 (m, 3H), 6.98 – 6.89 (m, 3H), 6.68 (dd, *J* = 9.0, 2.5 Hz, 1H), 3.91 (s, 2H), 3.79 (s, 3H), 2.42 (s, 3H), 2.21 (t, *J* = 7.4 Hz, 2H), 1.34 – 1.28 (m, 2H), 1.21 – 1.13 (m, 2H), 0.73 (t, *J* = 7.3 Hz, 3H); ^{13}C NMR (125 MHz, $CDCl_3$) δ 168.8, 168.3, 156.0, 144.2, 139.2, 136.1, 135.6, 135.0, 133.9, 131.2, 130.8, 130.7, 130.5, 129.1, 128.4, 128.4, 127.9, 127.72, 127.70, 114.9, 112.3, 111.9, 101.3, 55.6, 31.9, 31.1, 30.1, 21.6, 13.53, 13.47; HRMS (ESI-TOF) m/z: $[M+Na]^+$ calculated for $C_{37}H_{34}ClNNaO_4S$ 646.1795, found 646.1778.

4. General procedure for the synthesis of 1 and 10

General synthesis methods B and C to obtain 1

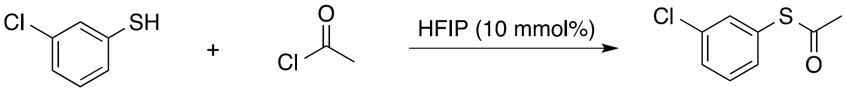

1i

Method B[1]**:** To a mixture of 3-chlorothiophenol (1446.2 mg, 10 mmol, 1.0 equiv) and acetyl chloride (942.0 mg, 12 mmol, 1.2 equiv) in an open flask, and add HFIP (118.0 mg, 1 mmol, 10 mol %) dropwise at room temperature in 1 min. The mixture is reacted at room temperature. (Be careful: the reaction must be open) Normally, the reaction can be completed within 30 minutes and consumed thiol completely determined by TLC analysis, then condensation reaction liquid removes most of the HFIP and acid chloride. The residue was purified by column chromatography on silica gel using PE/EtOAc (20:1) as the eluent and concentrated to obtain the product thioester **1i** (1550.3 mg, 83%). Synthesize **1i-1k**, **1s**, **1t**, **1z** according to this method.

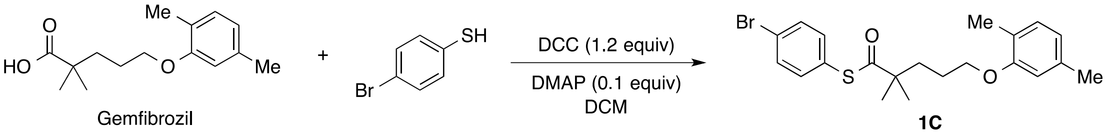

1C

Method C: To a mixture of Gemfibrozil (1251.7 mg, 5 mmol, 1.0 equiv), 4-bromobenzenethiol (1039.0 mg, 5.5 mmol, 1.1 equiv), dicyclohexylcarbodiimide (DCC 1238.0 mg, 6.0 mmol, 1.2 equiv), and 4-dimethylaminopyridine (DMAP 61.1 mg, 0.5mmol, 0.1equiv) into the flask, then dissolve with 10mL dichloromethane (DCM), and stir the mixture at room temperature. Consumed alcohol completely determined by TLC analysis, then filter off the insoluble white solid on diatomaceous earth, wash the filter cake twice with dichloromethane, concentrate the filtrate, the residue was purified by column chromatography on silica gel using PE/EtOAc (30:1) as the eluent and concentrated to obtain the product thioester **1cc** (1368.7 mg, 65%). Synthesize **1aa-1ee** according to this method.

1) S-(4-fluorophenyl) thioacetate (1b)[2]

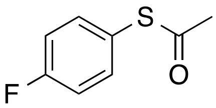

1b

^{1}H NMR (500 MHz, $CDCl_3$) δ 7.39 – 7.35 (m, 2H), 7.10 (t, *J* = 8.5 Hz, 2H), 2.41 (s, 3H).

2) S-(4-chlorophenyl) thioacetate (1c)[1]

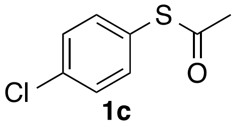

1c

^{1}H NMR (500 MHz, $CDCl_3$) δ 7.38 (d, *J* = 8.5 Hz, 2H), 7.33 (d, *J* = 8.7 Hz, 2H), 2.42 (s, 3H).

3) S-(4-bromophenyl) thioacetate (1d)[1]

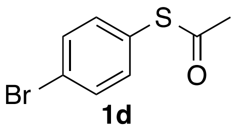

1d

^{1}H NMR (500 MHz, $CDCl_3$) δ 7.53 (d, *J* = 8.5 Hz, 2H), 7.26 (d, *J* = 8.5 Hz, 2H), 2.42 (s, 3H).

4) S-(4-iodophenyl) thioacetate (1e)[3]

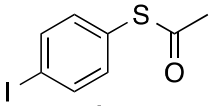

1e

^{1}H NMR (500 MHz, $CDCl_3$) δ 7.74 (d, *J* = 8.5 Hz, 2H), 7.13 (d, *J* = 8.5 Hz, 2H), 2.42 (s, 3H).

5) S-(4-nitrophenyl) thioacetate (1f)[4]

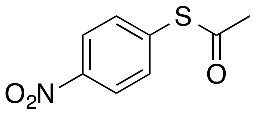

1f

^{1}H NMR (500 MHz, $CDCl_3$) δ 8.27 (d, *J* = 8.7 Hz, 2H), 7.62 (d, *J* = 8.8 Hz, 2H), 2.51 (s, 3H).

6) S-(4-methylphenyl) thioacetate (1g) [4]

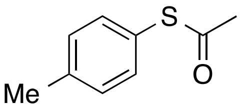

1g

^{1}H NMR (500 MHz, $CDCl_3$) δ 7.29 (d, *J* = 8.1 Hz, 2H), 7.21 (d, *J* = 8.0 Hz, 2H), 2.34 (s, 3H), 2.36 (s, 3H).

7) S-(4-methoxyphenyl) thioacetate (1h)[5]

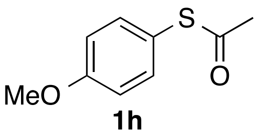

1h

^{1}H NMR (500 MHz, $CDCl_3$) δ 7.31 (d, *J* = 8.8 Hz, 2H), 6.93 (d, *J* = 8.8 Hz, 2H), 3.81 (s, 3H), 2.38 (s, 3H).

8) S-(3-chlorophenyl) thioacetate (1i)

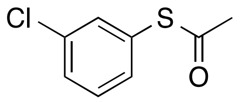

1i

The general procedure was followed using 3-chlorobenzenethiol (1446.2 mg, 10 mmol, 1.0 equiv), acetyl chloride (942.0 mg, 12 mmol, 1.2 equiv) and HFIP (118.0 mg, 1 mmol, 10 mol %) by method B (R_f = 0.59, PE/EtOAc = 20:1). After purification by column chromatography (PE/EtOAc 30:1), **1i** (1550.3 mg, 83%) was obtained as a colorless oil. ^{1}H NMR (500 MHz, $CDCl_3$) δ 7.42 – 7.28 (m, 4H), 2.42 (s, 3H); ^{13}C NMR (125 MHz, $CDCl_3$) δ 192.9, 134.7, 134.1, 132.5, 130.1, 129.6, 129.5, 30.2; HRMS (ESI-TOF) m/z: $[M+Na]^+$ calculated for $C_8H_7ClNaOS$ 208.9804, found 208.9792.

9) S-(2-bromophenyl) thioacetate (1j)

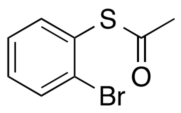

1j

The general procedure was followed using 2-bromobenzenethiol (1891.0 mg, 10 mmol, 1.0 equiv), acetyl chloride (942.0 mg, 12 mmol, 1.2 equiv) and HFIP (118.0 mg, 1 mmol, 10 mol %) by method B (R_f = 0.54, PE/EtOAc = 20:1). After purification by column chromatography (PE/EtOAc 20 :1), **1j** (1872.8 mg, 81%) was obtained as a colorless oil. ^{1}H NMR (600 MHz, $CDCl_3$) δ 7.69 (dd, *J* = 8.0, 1.3 Hz, 1H), 7.53 (dd, *J* = 7.7, 1.7 Hz, 1H), 7.35 (td, *J* = 7.6, 1.4 Hz, 1H), 7.26 (td, *J* = 7.7, 1.7 Hz, 1H), 2.44 (s, 3H); ^{13}C NMR (150 MHz, $CDCl_3$) δ 192.1, 137.0, 133.5, 131.1, 129.6, 129.2, 127.9, 30.2; HRMS (ESI-TOF) m/z: $[M+Na]^+$ calculated for $C_8H_7BrNaOS$ 252.9299, found 252.9287.

10) S-(3,5-bistrifluoromethylphenyl) thioacetate (1k)

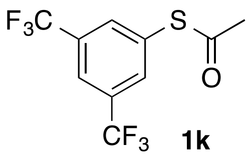

1k

The general procedure was followed using 3,5-bis(trifluoromethyl)benzenethiol (2462.0 mg, 10 mmol, 1.0 equiv), acetyl chloride (942.0 mg, 12 mmol, 1.2 equiv) and HFIP (118.0 mg, 1 mmol, 10 mol %) by method B (R_f = 0.67, PE/EtOAc = 20:1). After purification by column chromatography (PE/EtOAc 20 :1), **1k** (2160.6 mg, 75%) was obtained as a colorless oil. ^{1}H NMR (500 MHz, $CDCl_3$) δ 7.90 (s, 1H), 7.86 (s, 2H), 2.49 (s, 3H); ^{13}C NMR (150 MHz, $CDCl_3$) δ 191.4, 134.3 (d, *J* = 2.9 Hz), 132.4 (q, *J* = 33.8 Hz), 131.1, 123.2 (quint, *J* = 3.7 Hz), 122.8 (q, *J* = 273.0 Hz).

11) S-(2-naphthyl) thioacetate (1l)[1]

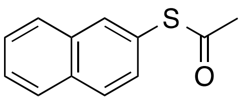

1l

^{1}H NMR (500 MHz, $CDCl_3$) δ 7.94 (s, 1H), 7.90 – 7.80 (m, 3H), 7.56 – 7.48 (m, 2H), 7.45 (m, 1H), 2.45 (s, 3H).

12) S-(benzyl) thioacetate (1m) [4]

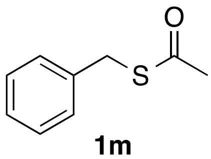

1m

^{1}H NMR (500 MHz, $CDCl_3$) δ 7.32 – 7.19 (m, 5H), 4.11 (s, 2H), 2.33 (s, 3H).

13) S-cyclohexyl ethanethioate (1o)[5]

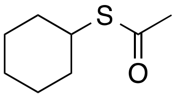

1o

^{1}H NMR (500 MHz, $CDCl_3$) δ 3.56 – 3.44 (m, 1H), 2.29 (s, 1H), 1.95 – 1.87 (m, 2H), 1.75 – 1.65 (m, 2H), 1.62 – 1.54 (m, 1H), 1.47 – 1.36 (m, 4H), 1.30 – 1.23 (m, 1H).

14) methyl *β*-(acetylthio)propionate (1p)[4]

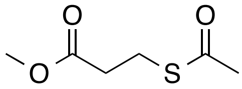

1p

^{1}H NMR (500 MHz, $CDCl_3$) δ 3.70 (s, 3H), 3.12 (t, J = 7.0 Hz, 2H), 2.64 (t, J = 7.0 Hz, 2H), 2.33 (s, 3H).

15) S-(4-chlorophenyl) thiopivalate (1s)

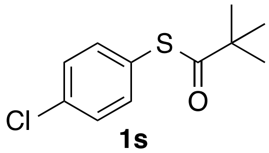

1s

The general procedure was followed using 4-chlorobenzenethiol (2892.4 mg, 20 mmol, 1.0 equiv), pivaloyl chloride (2894.0 mg, 24 mmol, 1.2 equiv) and HFIP (336.0 mg, 2 mmol, 10 mol %) by method B (R_f = 0.79, PE/EtOAc = 20:1). After purification by column chromatography (PE/EtOAc 20 :1), **1s** (3648.7 mg, 80%) was obtained as a colorless oil. ^{1}H NMR (500 MHz, $CDCl_3$) δ 7.39 – 7.27 (m, 4H), 1.30 (s, 9H). ^{13}C NMR (125 MHz, $CDCl_3$) δ 203.9, 136.1, 135.4, 129.2, 126.5, 46.9, 27.3; HRMS (ESI-TOF) m/z: $[M+Na]^+$ calculated for $C_{11}H_{13}ClNaOS$ 251.0273, found 251.0264.

16) S-(4-bromophenyl) thiopivalate (1t)

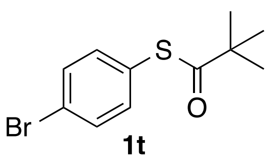

1t

The general procedure was followed using 4-bromobenzenethiol (1890.7 mg, 10 mmol, 1.0 equiv), pivaloyl chloride (1447.0 mg, 12 mmol, 1.2 equiv) and HFIP (118.0 mg, 1 mmol, 10 mol %) by method B (R_f = 0.79, PE/EtOAc = 20:1). After purification by column chromatography (PE/EtOAc 20 :1), **1t** (2121.1 mg, 78%) was obtained as a colorless oil. ^{1}H NMR (500 MHz, $CDCl_3$) δ 7.55 – 7.50 (m, 2H), 7.27 – 7.22 (m, 2H), 1.31 (s, 9H); ^{13}C

NMR (125 MHz, $CDCl_3$) δ 204.0, 136.4, 132.2, 127.2, 123.7, 47.0, 27.3; HRMS (ESI-TOF) m/z: $[M+Na]^+$ calculated for $C_{11}H_{13}BrNaOS$ 294.9768, found 294.9757.

17) S-(4-chlorophenyl) thiobenzoate (1u)[1]

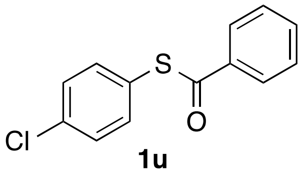

1u

^{1}H NMR (500 MHz, $CDCl_3$) δ 8.06 – 7.98 (m, 2H), 7.65 – 7.59 (m, 1H), 7.52 – 7.47 (m, 2H), 7.46 – 7.39 (m, 4H).

18) S-(4-bromophenyl) thiobenzoate (1v) [1]

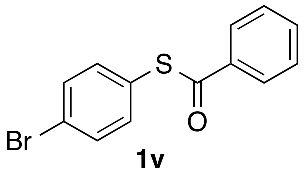

1v

^{1}H NMR (500 MHz, $CDCl_3$) δ 8.01 (d, *J* = 7.8 Hz, 2H), 7.65 – 7.56 (m, 3H), 7.52 – 7.45 (m, 2H), 7.42 – 7.33 (m, 2H).

19) S-(4-bromophenyl) dimethylcarbamothioate (1w)[6]

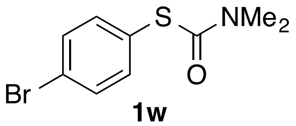

1w

^{1}H NMR (500 MHz, $CDCl_3$) δ 7.52 (d, *J* = 8.4 Hz, 2H), 7.37 (d, *J* = 8.4 Hz, 2H), 3.10 (s, 3H), 3.04 (s, 3H).

20) S-allyl ethanethioate (1x) [7]

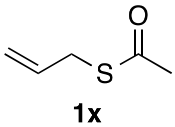

1x

^{1}H NMR (500 MHz, $CDCl_3$) δ 5.82 – 5.70 (m, 1H), 5.19 (d, *J* = 17.0 Hz, 1H), 5.06 (d, *J* = 10.1 Hz, 2H), 3.49 (d, *J* = 7.0 Hz, 2H), 2.30 (s, 3H).

21) S-propargyl ethanethiolate (1y) [8]

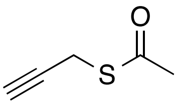

1y

^{1}H NMR (500 MHz, $CDCl_3$) δ 3.64 (d, *J* = 2.7 Hz, 2H), 2.37 (s, 3H), 2.18 (t, *J* = 2.7 Hz, 1H).

22) S-(4-methoxyphenyl) thiopropionate (1z)

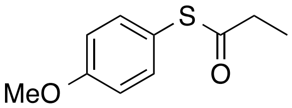

1z

The general procedure was followed using 4-methoxybenzenethiol (1402.0 mg, 10 mmol, 1.0 equiv), propionyl chloride (1110.2 mg, 12 mmol, 1.2 equiv) and HFIP (118.0 mg, 1 mmol, 10 mol %) by method B (R_f = 0.54, PE/EtOAc = 10:1). After purification by column chromatography (PE/EtOAc 10 :1), **1z** (1688.8 mg, 86%) was obtained as a colorless oil. ^{1}H NMR (500 MHz, $CDCl_3$) δ 7.35 (d, *J* = 8.9 Hz, 2H), 6.96 (d, *J* = 8.9 Hz, 2H), 2.68 (q, *J* = 7.5 Hz, 2H), 1.24 (t, *J* = 7.5 Hz, 3H). ^{13}C NMR (125 MHz, $CDCl_3$) δ 199.2, 160.5, 136.0, 118.4, 114.7, 55.2, 36.7, 9.5; HRMS (ESI-TOF) m/z: $[M+Na]^+$ calculated for $C_{10}H_{12}NaO_2S$ 219.0456, found 219.0447.

23) (3*S*,8*S*,9*S*,10*R*,13*R*,14*S*,17*R*)-10,13-dimethyl-17-((*R*)-6-methylheptan-2-yl)-2,3,4,7,8,9,10,11,12,13,14,15,16,17-tetradecahydro-1*H*-cyclopenta[*a*]phenanthren-3-yl 3-(acetylthio)propanoate (1A)

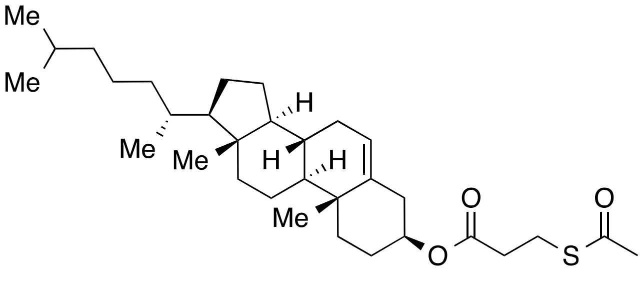

1A

The general procedure was followed using Cholesterol (2319.9 mg, 6 mmol, 1.2 equiv), 3-(acetylthio)propionic acid (740.9 mg, 5.0 mmol, 1.0 equiv), DCC (1133.0 mg, 5.5 mmol, 1.1 equiv), and DMAP (61.1 mg, 0.5 mmol, 0.1equiv) by method C (R_f = 0.45, PE/EtOAc = 20:1). After purification by column chromatography (PE/EtOAc 20:1), **1A** (1201.5 mg, 46%) was obtained as a white solid. ^{1}H NMR (500 MHz, $CDCl_3$) δ 5.42 – 5.34 (m, 1H), 4.69 – 4.58 (m, 1H), 3.11 (t, *J* = 6.9 Hz, 2H), 2.60 (t, *J* = 6.9 Hz, 2H), 2.39 (s, 3H), 2.38 – 2.26 (m, 2H), 2.06 – 1.92 (m, 2H), 1.91 – 1.78 (m, 3H), 1.63 – 1.05 (m, 19H), 1.02 (s, 3H), 1.02 – 0.95 (m, 2H), 0.91 (d, *J* = 6.3 Hz, 3H), 0.86 (d, *J* = 6.3 Hz, 6H), 0.68 (s, 3H). ^{13}C NMR (125 MHz, $CDCl_3$) δ 195.5, 171.0, 139.5, 122.7, 74.5, 56.7, 56.1, 50.0, 42.3, 39.7, 39.5, 38.0, 36.9, 36.6, 36.2, 35.8, 34.7, 31.9, 31.8, 30.5, 28.2, 28.0, 27.7, 24.3, 23.8, 22.8, 22.5, 21.0, 19.3, 18.7, 11.8; HRMS (ESI-TOF) m/z: $[M+Na]^+$ calculated for $C_{32}H_{52}NaO_3S$ 539.3535, found 539.3524.

24) (8*R*,9*S*,13*S*,14*S*,17*S*)-17-((3-(acetylthio)propanoyl)oxy)-13-methyl-7,8,9,11,12,13,14,15,16,17-decahydro-6*H*-cyclopenta[*a*]phenanthren-3-yl benzoate (1B)

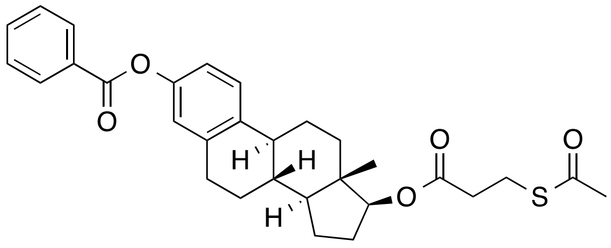

1B

The general procedure was followed using Estradiol benzoate (903.6 mg, 2.4 mmol, 1.2 equiv), 3-(acetylthio)propionic acid (300 mg, 2.0 mmol, 1.0 equiv), DCC (453.0 mg, 2.2 mmol, 1.1 equiv), and DMAP (25.0 mg, 0.2 mmol, 0.1 equiv) by method C (R_f = 0.40, PE/EtOAc = 20:1). After purification by column chromatography (PE /EtOAc 20:1), **1B** (701.2 mg, 69%) was obtained as a white solid. ^{1}H NMR (500 MHz, $CDCl_3$) δ 8.19 (d, *J* = 7.8 Hz, 2H), 7.63 (t, *J* = 7.4 Hz, 1H), 7.50 (d, *J* = 7.7 Hz, 2H), 7.33 (d, *J* = 8.5 Hz, 1H), 6.97 (dd, *J* = 8.4, 2.2 Hz, 1H), 6.92 (d, *J* = 2.3 Hz, 1H), 4.73 (t, *J* = 8.4 Hz, 1H), 3.13 (t, *J* = 6.9 Hz, 2H), 2.93 – 2.87 (m, 2H), 2.65 (t, *J* = 6.9 Hz, 2H), 2.34 (s, 3H), 2.32 – 2.19 (m, 2H), 1.95 – 1.87 (m, 2H), 1.80 – 1.73 (m, 1H), 1.64 – 1.25 (m, 8H), 0.84 (s, 3H); ^{13}C NMR (125 MHz, $CDCl_3$) δ 195.5, 171.6, 165.4, 148.7, 138.2, 137.8, 133.4, 130.1, 129.7, 128.5, 126.4, 121.6, 118.7, 83.1, 49.8, 44.0, 42.9, 38.2, 36.9, 34.6, 30.5, 29.5, 27.5, 27.0, 26.0, 24.3, 23.3, 12.1; HRMS (ESI-TOF) m/z: $[M+Na]^+$ calculated for $C_{30}H_{34}NaO_5S$ 529.2025, found 259.2006.

25) *S*-(4-bromophenyl) 5-(2,5-dimethylphenoxy)-2,2-dimethylpentanethioate (1C)

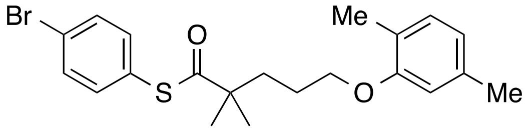

1C

The general procedure was followed using Gemfibrozil (1251.7 mg, 5.0 mmol, 1.0 equiv), 4-bromobenzenethiol (1039.0 mg, 5.5 mmol, 1.1 equiv), DCC (1238.0 mg, 6.0 mmol, 1.2 equiv), and DMAP (61.1 mg, 0.5mmol, 0.1equiv) by method C (R_f = 0.57, PE/EtOAc = 20:1). After purification by column chromatography (PE/EtOAc 30 :1), **1C** (1368.7 mg, 65%) was obtained as a colorless oil. ^{1}H NMR (500 MHz, $CDCl_3$) δ 7.59 (d, *J* = 8.4 Hz, 2H), 7.29 (d, *J* = 8.4 Hz, 2H), 7.10 (d, *J* = 7.5 Hz, 1H), 6.76 (d, *J* = 7.5 Hz, 1H), 6.70 (s, 1H), 4.02 (t, *J* = 5.8 Hz, 2H), 2.40 (s, 3H), 2.29 (s, 3H), 1.98 – 1.84 (m, 4H), 1.42 (s, 6H); ^{13}C NMR (125 MHz, $CDCl_3$) δ 203.3, 156.7, 136.4, 136.3, 132.2, 130.3, 127.0, 123.7, 123.5, 120.7, 111.8, 67.5, 50.1, 37.5, 25.2, 24.8, 21.4, 15.8; HRMS (ESI-TOF) m/z: $[M+Na]^+$ calculated for $C_{21}H_{25}BrNaO_2S$ 443.0656, found 443.0650.

26) 2-(((4-bromophenyl)thio)carbonyl)phenyl acetate (1D)

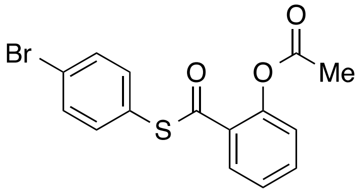

1D

The general procedure was followed using Aspirin (900.8 mg, 5.0 mmol, 1.0 equiv), 4-bromobenzenethiol (1040.0 mg, 5.5 mmol, 1.1 equiv), DCC (1236 mg, 6.0 mmol, 1.2 equiv), and DMAP (61.1 mg, 0.5mmol, 0.1equiv) by method C (R_f = 0.36, PE/EtOAc = 10:1). After purification by column chromatography (PE/EtOAc 10:1 to 5:1), **1D** (1103.7 mg, 63%) was

obtained as a white solid. ^{1}H NMR (600 MHz, $CDCl_3$) δ 8.02 (dd, *J* = 7.8, 1.6 Hz, 1H), 7.62 – 7.55 (m, 3H), 7.39 – 7.32 (m, 3H), 7.16 (dd, *J* = 8.2, 1.2 Hz, 1H), 2.31 (s, 3H); ^{13}C NMR (150 MHz, $CDCl_3$) δ 187.5, 169.3, 148.2, 136.4, 134.1, 132.5, 129.7, 129.5, 126.5, 126.2, 124.4, 124.1, 21.1; HRMS (ESI-TOF) m/z: $[M+Na]^+$ calculated for $C_{15}H_{11}BrNaO_3S$ 372.9510, found 372.9505.

27) *S*-butyl 2-(1-(4-chlorobenzoyl)-5-methoxy-2-methyl-1*H*-indol-3-yl)ethanethioate (1E)

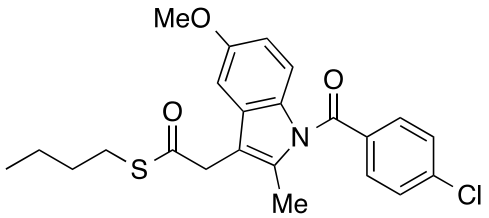

1E

The general procedure was followed using Indometacin (1789.0 mg, 5.0 mmol, 1.0 equiv), butanethiol (496.0 mg, 5.5 mmol, 1.1 equiv), DCC (1236.0 mg, 6.0 mmol, 1.2 equiv), and DMAP (61.1 mg, 0.5mmol, 0.1equiv) by method C (R_f = 0.44, PE/EtOAc = 10:1). After purification by column chromatography (PE/EtOAc 10:1 to 5:1), **1E** (1706.2 mg, 79%) was obtained as a yellow oil. ^{1}H NMR (500 MHz, $CDCl_3$) δ 7.66 (d, *J* = 8.5 Hz, 2H), 7.47 (d, *J* = 8.5 Hz, 2H), 6.95 (d, *J* = 2.5 Hz, 1H), 6.89 (d, *J* = 9.0 Hz, 1H), 6.68 (dd, *J* = 9.0, 2.6 Hz, 1H), 3.85 (s, 2H), 3.83 (s, 3H), 2.86 (t, *J* = 7.4 Hz, 2H), 2.39 (s, 3H), 1.57 – 1.49 (m, 2H), 1.39 – 1.32 (m, 2H), 0.89 (t, *J* = 7.4 Hz, 3H); ^{13}C NMR (125 MHz, $CDCl_3$) δ 197.2, 168.2, 156.1, 139.3, 136.5, 133. 8, 131.2, 130.8, 130.6, 129.1, 114.9, 112.3, 111.8, 101.2, 55.7, 39.4, 31.4, 28.9, 21.9, 13.5, 13.5; HRMS (ESI-TOF) m/z: $[M+Na]^+$ calculated for $C_{23}H_{24}ClNNaO_3S$ 452.1063, found 452.1059.

28) 2-thiaspiro[3.5]nonan-1-one (10a)[9]

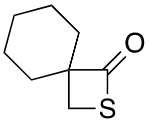

10a

^{1}H NMR (600 MHz, $CDCl_3$) δ 2.80 (s, 3H), 1.92 – 1.76 (m, 4H), 1.75 – 1.67 (m, 2H), 1.55 – 1.46 (m, 1H), 1.44 – 1.24 (m, 3H).

29) 3-benzylthietan-2-one (10b)[10]

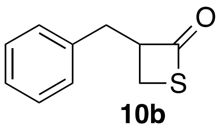

10b

^{1}H NMR (600 MHz, $CDCl_3$) δ 7.35 – 7.29 (m, 2H), 7.27 – 7.25 (m, 1H), 7.18 (d, *J* = 7.5 Hz, 2H), .4.46 (tt, *J* = 8.3, 4.4 Hz, 1H), 3.12 (dd, *J* = 14.3, 5.3 Hz, 1H), 3.05 – 2.99 (m, 2H), 2.81 (dd, *J* = 8.8, 4.0 Hz, 1H).

30) tetrahydro-2H-thiopyran-2-one (10d)[11]

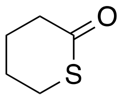

10d

^{1}H NMR (500 MHz, $CDCl_3$) δ 3.16 (t, *J* = 6.1 Hz, 2H), 2.61 (t, *J* = 6.3 Hz, 2H), 2.05 – 1.95 (m, 4H).

31) isothiochroman-3-one (10e)[12]

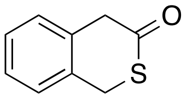

10e

^{1}H NMR (500 MHz, $CDCl_3$) δ 7.38 – 7.17 (m, 4H), 4.22 (s, 2H), 3.79 (s, 2H).

32) thiepan-2-one (10f)[13,14]

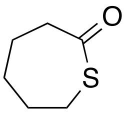

10f

^{1}H NMR (600 MHz, $CDCl_3$) δ 3.05 – 2.99 (m, 2H), 2.88 – 2.81 (m, 2H), 2.17 – 2.06 (m, 2H), 1.88 – 1.80 (m, 2H), 1.80 – 1.74 (m, 2H).

5. General procedure for the synthesis of 2

General synthesis methods D and E to obtain 2

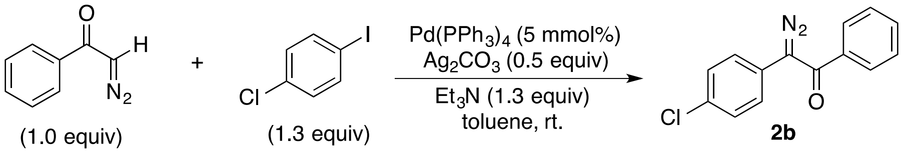

Method D: 2-diazo-1-phenylethan-1-one (438.0 mg, 3.0 mmol, 1.0 equiv), 1-chloro-4-iodobenzene (930.0 mg, 3.9 mmol, 1.3 equiv), $Pd(PPh_3)_4$ (173.3 mg, 0.15 mmol, 5 mol%), Ag_2CO_3 (413.6 mg, 1.5 mmol, 0.5 equiv) and Et_3N (395.0 mg, 3.9 mmol, 1.3 equiv) were introduced into a dried glass tube under N_2 protection. Add dry toluene (5 mL) as a solvent, stir at room temperature. Consumed diazo completely determined by TLC analysis, filter the insoluble solids, concentrate the solvent, and the residue was purified by column chromatography on silica gel using ethyl acetate/petroleum ether (1:20) as the eluent and concentrated to obtain the product diazoketone **2b** (476.8 mg, 62%). Synthesize **2b** – **2g** according to this method.

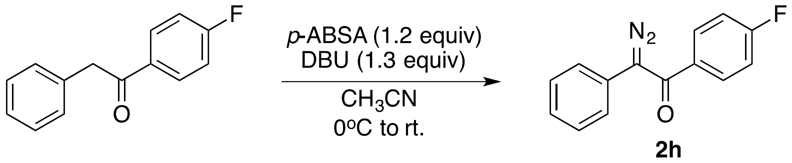

Method E: 1-(4-fluorophenyl)-2-phenylethan-1-one (1071.2 mg, 5.0 mmol, 1.0 equiv) and *p*-acetamidobenzenesulfonyl azide (*p*-ABSA 1441.0 mg, 6.0 mmol, 1.2 equiv) were introduced into a dried glass tube under N_2 protection, and add 10 mL dry CH_3CN as solvent. Then add 1,8-diazabicyclo[5.4.0]undec-7-ene (DBU 990.0 mg, 6.5 mmol, 1.3 equiv) dropwise at 0°C, and gradually return to room temperature to react after the addition is complete. Consumed ketone completely determined by TLC analysis. Concentrated most of the solvent at low temperature, then add 50 ml of water and extract three times with ether. The combined organic phases are dried with anhydrous sodium sulfate. The ether is removed at low temperature and the residue was purified by column chromatography on silica gel using ethyl acetate/petroleum ether (1:20) as the eluent and concentrated to obtain the product diazoketone **2h** (835.7 mg, 70%). Synthesize **2h**, **2j**, **2k**, **2n**, **2o**, **2v**, **2w** according to this method.

1) 2-diazo-1,2-diphenylethan-1-one (2a)[15]

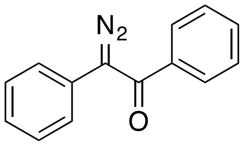

2a

^{1}H NMR (500 MHz, $CDCl_3$) δ 7.62 (d, *J* = 8.3 Hz, 2H), 7.52 – 7.38 (m, 7H), 7.29 – 7.24 (m, 1H).

2) *p*-chlorophenylbenzoyldiazomethane (2b)

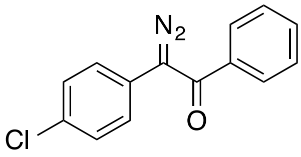

2b

The general procedure was followed using 2-diazo-1-phenylethan-1-one (438.0 mg, 3.0 mmol, 1.0 equiv), 1-chloro-4-iodobenzene (930.0 mg, 3.9 mmol, 1.3 equiv), $Pd(PPh_3)_4$ (173.3 mg, 0.15 mmol, 5 mol%), Ag_2CO_3 (413.6 mg, 1.5 mmol, 0.5equiv) and Et_3N (395.0 mg, 3.9 mmol, 1.3 equiv) by method D (R_f = 0.60, PE/EtOAc = 10:1). After purification by column chromatography (PE/EtOAc 10 :1), **2b** (476.8 mg, 62%) was obtained as an orange solid. ^{1}H NMR (500 MHz, $CDCl_3$) δ 7.61 (d, *J* = 7.0 Hz, 2H), 7.54 – 7.49 (m, 1H), 7.47 – 7.42 (m, 4H), 7.40 – 7.35 (m, 2H); ^{13}C NMR (125 MHz, $CDCl_3$) δ 188.1, 137.7, 132.7, 131.9, 129.2, 128.6, 127.6, 127.0, 124.6, 72.4.

3) *p*-bromophenylbenzoyldiazomethane (2c)

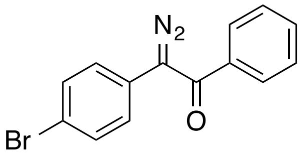

2c

The general procedure was followed using 2-diazo-1-phenylethan-1-one (438.0 mg, 3.0 mmol, 1.0 equiv), 1-bromo-4-iodobenzene (1103.0 mg, 3.9 mmol, 1.3 equiv), $Pd(PPh_3)_4$ (173.3 mg, 0.15 mmol, 5 mol%), Ag_2CO_3 (413.6 mg, 1.5 mmol, 0.5equiv) and Et_3N (395.0 mg, 3.9 mmol, 1.3 equiv) by method D (R_f = 0.60, PE/EtOAc = 10:1). After purification by column chromatography (PE/EtOAc 10 :1), **2c** (524.1 mg, 58%) was obtained as an orange solid. ^{1}H NMR (500 MHz, $CDCl_3$) δ 7.63 – 7.59 (m, 2H), 7.55 – 7.49 (m, 3H), 7.46 – 7.41 (m, 2H), 7.40 – 7.34 (m, 2H); ^{13}C NMR (125 MHz, $CDCl_3$) δ 188.1, 137.7, 132.1, 131.9, 128.3, 127.6, 127.2, 125.2, 120.6, 72.5.

4) *m*-bromophenylbenzoyldiazomethane (2d)

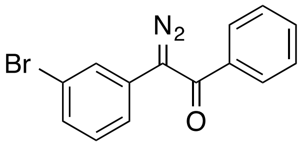

2d

The general procedure was followed using 2-diazo-1-phenylethan-1-one (438.0 mg, 3.0 mmol, 1.0 equiv), 1-bromo-3-iodobenzene (1103.0 mg, 3.0 mmol, 1.0 equiv), $Pd(PPh_3)_4$ (173.3 mg, 0.15 mmol, 5 mol%), Ag_2CO_3 (413.6 mg, 1.5 mmol, 0.5equiv) and Et_3N (395.0 mg, 3.9 mmol, 1.3 equiv) by method D (R_f = 0.58, PE/EtOAc = 10:1). After purification by column chromatography (PE/EtOAc 10 :1), **2d** (470.0 mg, 52%) was obtained as an orange solid. ^{1}H NMR (500 MHz, $CDCl_3$) δ 7.70 (t, *J* = 1.9 Hz, 1H), 7.64 – 7.59 (m, 2H), 7.55 – 7.51 (m, 1H), 7.47 – 7.36 (m, 4H), 7.27 (t, *J* = 8.0 Hz, 1H); ^{13}C NMR (125 MHz, $CDCl_3$) δ 187.9, 137.6, 132.0, 130.4, 129.9, 128.7, 128.4, 128.3, 127.6, 124.2, 123.1.

5) *m*-cyanophenylbenzoyldiazomethane (2e)

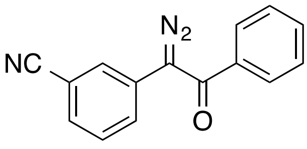

2e

The general procedure was followed using 2-diazo-1-phenylethan-1-one (730.7 mg, 5.0 mmol, 1.3equiv), 3-iodobenzonitrile (1374.1 mg, 6.0 mmol, 1.2 equiv), $Pd(PPh_3)_4$ (289.0 mg, 0.25 mmol, 5 mol%), Ag_2CO_3 (689.4 mg, 2.5 mmol, 0.5equiv) and Et_3N (658.0 mg, 6.5 mmol, 1.3 equiv) by method D (R_f

= 0.53, PE/EtOAc = 5:1). After purification by column chromatography (PE/EtOAc 10 :1), **2e** (889.7 mg, 72%) was obtained as an orange solid. ^{1}H NMR (500 MHz, $CDCl_3$) δ 7.92 – 7.90 (m, 1H), 7.79 – 7.74 (m, 1H), 7.67 – 7.61 (m, 2H), 7.58 – 7.52 (m, 3H), 7.51 – 7.45 (m, 2H); ^{13}C NMR (125 MHz, $CDCl_3$) δ 187.7, 137.3, 132.2, 130.1, 129.7, 129.1, 128.8, 128.5, 128.3, 127.5, 118.3, 113.4, 72.1.

6) methyl 4-(1-diazo-2-oxo-2-phenylethyl)benzoate (2f)[16]

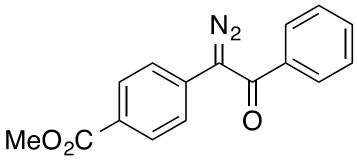

2f

The general procedure was followed using 2-diazo-1-phenylethan-1-one (1460.0 mg, 10 mmol, 1.0 equiv), methyl 4-iodobenzoate (3144.0 mg, 12 mmol, 1.2 equiv), $Pd(PPh_3)_4$ (577.8 mg, 0.5 mmol, 5 mol%), Ag_2CO_3 (1378.8 mg, 5 mmol, 0.5 equiv) and Et_3N (1315.0 mg, 13 mmol, 1.3 equiv) by method D (R_f = 0.41, PE/EtOAc = 10:1). After purification by column chromatography (PE/EtOAc 10:1 to 5:1), **2f** (1978.2 mg, 71%) was obtained as an orange solid. ^{1}H NMR (500 MHz, $CDCl_3$) δ 8.06 (d, J = 8.5 Hz, 2H), 7.63 (d, J = 8.2 Hz, 2H), 7.58 (d, J = 8.0 Hz, 2H), 7.56 – 7.51 (m, 1H), 7.48 – 7.43 (m, 2H), 3.92 (s, 3H); ^{13}C NMR (125 MHz, $CDCl_3$) δ 187.9, 166.5, 137.7, 132.0, 131.4, 130.2, 128.7, 128.1, 127.6, 124.8, 73.3, 52.1.

7) ethyl 3-(1-diazo-2-oxo-2-phenylethyl)benzoate (2g)

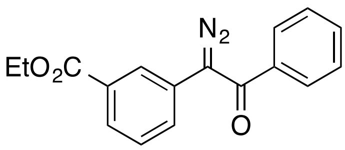

2g

The general procedure was followed using 2-diazo-1-phenylethan-1-one (438.0 mg, 3.0 mmol, 1.0 equiv), ethyl 3-iodobenzoate (1076.7 mg, 3.9 mmol, 1.3 equiv), $Pd(PPh_3)_4$ (173.3 mg, 0.15 mmol, 5 mol%), Ag_2CO_3 (413.6 mg, 1.5 mmol, 0.5equiv) and Et_3N (395.0 mg, 3.9 mmol, 1.3 equiv) by method D (R_f = 0.39, PE/EtOAc = 10:1). After purification by column chromatography (PE/EtOAc 10:1 to 5:1), **2g** (561.1 mg, 63%) was obtained as an orange solid. ^{1}H NMR (500 MHz, $CDCl_3$) δ 8.09 (s, 1H), 7.94 (d, J = 7.8 Hz, 1H), 7.78 (d, J = 7.9 Hz, 1H), 7.63 (d, J = 7.0 Hz, 2H), 7.55 – 7.41 (m, 4H), 4.39 (q, J = 7.1 Hz, 2H), 1.39 (t, J = 7.1 Hz, 3H); ^{13}C NMR (125 MHz, $CDCl_3$) δ 188.1, 166.0, 137.7, 131.9, 131.3, 130.4, 129.1, 128.6, 127.9, 127.6, 126.8, 126.2, 72.6, 61.2, 14.3.

8) 2-diazo-1-(4-fluorophenyl)-2-phenylethan-1-one (2h)

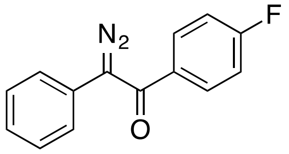

2h

The general procedure was followed using 1-(4-fluorophenyl)-2-phenylethan-1-one (1071.2 mg, 5.0 mmol, 1.0 equiv), *p*-ABSA (1441.0 mg, 6.0 mmol, 1.2 equiv) and DBU (990.0 mg, 6.5 mmol, 1.3 equiv) by method E (R_f = 0.60, PE/EtOAc = 10:1). After purification by column chromatography (PE/EtOAc 20:1), **2h** (835.7 mg, 70%) was obtained as an orange oil. ^{1}H NMR (600 MHz, $CDCl_3$) δ 7.63 (dd, J = 8.1, 5.6 Hz, 2H), 7.45 – 7.38 (m, 4H), 7.29 – 7.26 (m, 1H), 7.09 (t, J = 8.6 Hz, 2H).

9) 2-diazo-1,2-di(naphthalen-2-yl)ethan-1-one (2i)

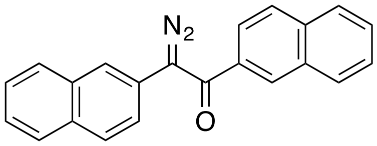

2i

1,2-di(naphthalen-2-yl)ethan-1-one (2961.0 mg, 10.0 mmol, 1.0 equiv), and TsN_3 (75% in EtOAc) (3944.0 mg, 15 mmol, 1.5 equiv) were introduced into a dried glass tube under N_2 protection, and add 30 mL dry CH_3CN as solvent. Then add DBU (2280.0 mg, 15 mmol, 1.5 equiv) in 5 mL dry CH_3CN dropwise at -10°C and keep this temperature to react. Consumed ketone completely determined by TLC analysis (R_f = 0.61, PE/EtOAc = 10:1). Concentrated most of the solvent at low temperature, then add 50 ml of water and extract three times with ether. The combined organic phases are dried with anhydrous sodium sulfate. The ether is removed at low temperature and the residue was purified by column chromatography on silica gel using ethyl acetate/petroleum ether (1:20) as the eluent and concentrated to obtain the product **2i** (1401.5 mg, 43%) as an orange solid. ^{1}H NMR (500 MHz, DMSO) δ 8.41 (s, 1H), 8.21 (d, *J* = 1.9 Hz, 1H), 8.08 – 7.98 (m, 4H), 7.96 – 7.90 (m, 2H), 7.77 (dd, *J* = 8.5, 1.8 Hz, 1H), 7.70 – 7.60 (m, 3H), 7.57 – 7.50 (m, 2H); ^{13}C NMR (125 MHz, $CDCl_3$) δ 188.2, 135.2, 134.7, 133.5, 132.3, 132.0, 129.0, 128.7, 128.43, 128.41, 127.9, 127.8, 127.7, 127.6, 126.8, 126.6, 126.2, 124.7, 124.3, 123.8, 123.4, 73.7.

10) 1-(4-chlorophenyl)-1-diazopropan-2-one (2j)

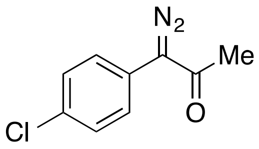

2j

The general procedure was followed using 1-(4-chlorophenyl)propan-2-one (843.0 mg, 5.0 mmol, 1.0 equiv), *p*-ABSA (1441.0 mg, 6.0 mmol, 1.2 equiv) and DBU (990.0 mg, 6.5 mmol, 1.3 equiv) by method E (R_f = 0.43, PE/EtOAc = 20:1). After purification by column chromatography (PE/EtOAc 20:1), **2j** (782.4 mg, 80%) was obtained as an orange solid. ^{1}H NMR (500 MHz, $CDCl_3$) δ 7.44 (d, *J* = 8.4 Hz, 2H), 7.37 (d, *J* = 8.6 Hz, 2H), 2.38 (s, 3H); ^{13}C NMR (125 MHz, $CDCl_3$) δ 189.6, 132.6, 129.1, 126.7, 123.9, 72.2, 26.9.

11) 1-cyclopropyl-2-diazo-2-(2-fluorophenyl)ethan-1-one (2k)

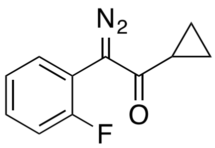

2k

The general procedure was followed using 1-cyclopropyl-2-(2-fluorophenyl)ethan-1-one (891.0 mg, 5.0 mmol, 1.0 equiv), *p*-ABSA (1441.0 mg, 6.0 mmol, 1.2 equiv) and DBU (1141.8 mg, 7.5 mmol, 1.5 equiv) by method E (R_f = 0.65, PE/EtOAc = 10:1). After purification by column chromatography (PE/DCM 6:1), **2k** (502.0 mg, 49%) was obtained as an orange oil. This compound is prone to deterioration, use immediately after rapid treatment. ^{1}H NMR (500 MHz, $CDCl_3$) δ 7.58 (t, *J* = 7.5 Hz, 1H), 7.38 – 7.30 (m, 1H), 7.22 (t, *J* = 7.6 Hz, 1H), 7.14 (t, *J* = 9.4 Hz, 1H), 2.06 – 1.96 (m, 1H), 1.23 – 1.16 (m, 2H), 0.94 – 0.87 (m, 2H).

12) 2-diazo-1-phenylpropan-1-one (2l)[17]

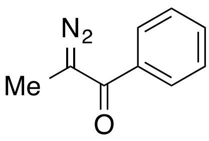

2l

^{1}H NMR (500 MHz, $CDCl_3$) δ 7.57 (d, *J* = 7.4 Hz, 2H), 7.51 – 7.46 (m, 1H), 7.45 – 7.40 (m, 2H), 2.41 (s, 3H).

13) 2-diazo-1-phenylethan-1-one (2m)[18]

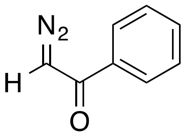

2m

^{1}H NMR (500 MHz, $CDCl_3$) δ 7.76 (d, *J* = 7.6 Hz, 2H), 7.58 – 7.52 (m, 1H), 7.49 – 7.41 (m, 2H), 5.92 (s, 1H).

14) 1-(4-chlorophenyl)-1-diazo-4-phenylbut-3-yn-2-one (2n)

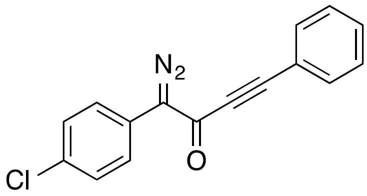

2n

The general procedure was followed using 1-(4-chlorophenyl)-4-phenylbut-3-yn-2-one (2750.8 mg, 10.8 mmol, 1.0 equiv), *p*-ABSA (3890.0 mg, 16.2 mmol, 1.5 equiv) and DBU (2466.0 mg, 16.2 mmol, 1.5 equiv) by method E (R_f = 0.70, PE/EtOAc = 10:1). After purification by column chromatography (PE/EtOAc 20:1), **2n** (2294.2 mg, 82%) was obtained as an orange solid. ^{1}H NMR (500 MHz, $CDCl_3$) δ 7.63 – 7.53 (m, 4H), 7.47 (t, *J* = 7.5 Hz, 1H), 7.43 – 7.35 (m, 4H); ^{13}C NMR (125 MHz, $CDCl_3$) δ 168.8, 132.8, 130.9, 129.2, 128.7, 126.0, 119.5, 77.9.

15) 1-(4-bromophenyl)-1-diazo-4-phenylbut-3-yn-2-one (2o)

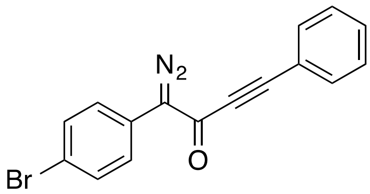

2o

The general procedure was followed using 1-(4-bromophenyl)-4-phenylbut-3-yn-2-one (1500.0 mg, 5 mmol, 1.0 equiv), *p*-ABSA (1802.0 mg, 7.5 mmol, 1.5 equiv) and DBU (1142.0 mg, 7.5 mmol, 1.5 equiv) by method E (R_f = 0.70, PE/EtOAc = 10:1). After purification by column chromatography (PE/EtOAc 20:1), **2n** (1270.5 mg, 78%) was obtained as an orange solid. ^{1}H NMR (600 MHz, $CDCl_3$) δ 7.62 – 7.52 (m, 4H), 7.51 – 7.44 (m, 3H), 7.42 – 7.35 (m, 2H); ^{13}C NMR (150 MHz, $CDCl_3$) δ 168.7, 132.8, 132.2, 130.9, 128.7, 126.2, 123.6, 120.8, 119.5, 91.1, 84.8, 77.9.

16) methyl 2-diazo-2-phenylacetate (2p)[19]

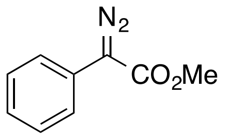

2p

^{1}H NMR (500 MHz, $CDCl_3$) δ 7.48 (d, *J* = 8.3 Hz, 2H), 7.40 (t, *J* = 7.6 Hz, 2H), 7.18 (t, *J* = 7.4 Hz, 1H), 3.86 (s, 3H).

17) ethyl 2-diazo-2-phenylacetate (2q)[19]

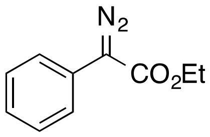

2q

^{1}H NMR (500 MHz, $CDCl_3$) δ 7.48 (d, *J* = 8.3 Hz, 2H), 7.38 (t, *J* = 7.2 Hz, 2H), 7.17 (t, *J* = 7.4 Hz, 1H), 4.33 (q, *J* = 7.1 Hz, 2H), 1.34 (t, *J* = 7.1 Hz, 3H).

18) isopropyl 2-diazo-2-phenylacetate (2r)[19]

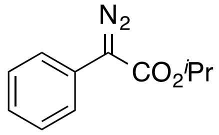

2r

^{1}H NMR (600 MHz, $CDCl_3$) δ 7.48 (d, *J* = 8.5 Hz, 2H), 7.37 (t, *J* = 7.9 Hz, 2H), 7.17 (t, *J* = 7.4 Hz, 1H), 5.20 (sept, *J* = 6.3 Hz, 1H), 1.32 (d, *J* = 6.3 Hz, 6H).

19) methyl 2-(4-bromophenyl)-2-diazoacetate (2s)[20]

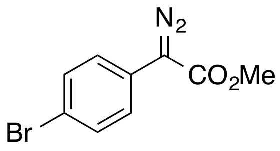

2s

^{1}H NMR (500 MHz, $CDCl_3$) δ 7.50 (d, *J* = 8.7 Hz, 2H), 7.36 (d, *J* = 8.7 Hz, 2H), 3.87 (s, 3H).

20) methyl 2-diazo-2-(2-ethynylphenyl)acetate (2t)[21]

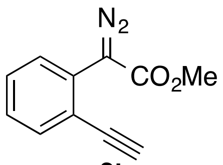

2t

^{1}H NMR (500 MHz, $CDCl_3$) δ 7.60 (d, *J* = 8.0 Hz, 1H), 7.54 (d, *J* = 7.8 Hz, 1H), 7.41 (t, *J* = 7.7 Hz, 1H), 7.27 (t, *J* = 6.6 Hz, 1H), 3.85 (s, 3H), 3.47 (s, 1H).

21) methyl 2-diazo-2-(4-(trifluoromethyl)phenyl)acetate (2u)[20]

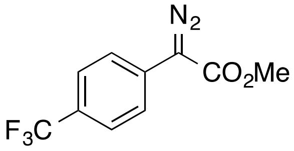

2u

^{1}H NMR (500 MHz, $CDCl_3$) δ 7.66 – 7.57 (m, 4H), 3.89 (s, 3H).

22) *tert*-butyl 2-diazo-2-(4-(trifluoromethyl)phenyl)acetate (2v)

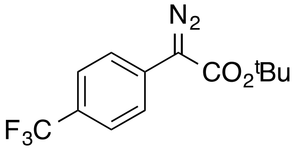

2v

The general procedure was followed using tert-butyl 2-(4-(trifluoromethyl)phenyl) acetate (1301.0 mg, 5.0 mmol, 1.0 equiv), *p*-ABSA (1441.0 mg, 6.0 mmol, 1.2 equiv) and DBU (990.0 mg, 6.5 mmol, 1.3 equiv) by method E (R_f = 0.80, PE/EtOAc = 10:1). After purification by column chromatography (PE/EtOAc 20:1), **2v** (684.2 mg, 48%) was obtained as an orange oil. ^{1}H NMR (600 MHz, $CDCl_3$) δ 7.62 – 7.56 (m, 4H), 1.56 (s, 9H); ^{13}C NMR (150 MHz, $CDCl_3$) δ 163.7, 130.7, 127.22 (q, *J* = 32.7 Hz), 125.7 (q, *J* = 3.8 Hz), 124.1 (q, *J* = 271.4 Hz), 123.40, 82.6, 28.3.

23) 2,2,2-trifluoroethyl 2-diazo-2-phenylacetate (2w)

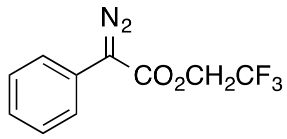

2w

The general procedure was followed using 2,2,2-trifluoroethyl 2-phenylacetate (1091.0 mg, 5.0 mmol, 1.0 equiv), *p*-ABSA (1441.0 mg, 6.0 mmol, 1.2 equiv) and DBU (990.0 mg, 6.5 mmol, 1.3 equiv) by method E (R_f = 0.80, PE/EtOAc = 20:1). After purification by column chromatography (PE/EtOAc 20:1), **2w** (431.3 mg, 35%) was obtained as an orange solid. This compound is prone to deterioration, use immediately after rapid treatment. ^{1}H NMR (500 MHz, $CDCl_3$) δ 7.49 – 7.36 (m, 4H), 4.65 (q, *J* = 8.3 Hz, 2H).

24) 1-diazonaphthalen-2(1*H*)-one (2x)[22]

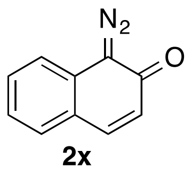

2x

^{1}H NMR (600 MHz, $CDCl_3$) δ 7.65 (d, *J* = 9.7 Hz, 1H), 7.59 (d, *J* = 7.7 Hz, 1H), 7.52 (t, *J* = 7.6 Hz, 1H), 7.31 – 7.27 (m, 2H), 6.68 (d, *J* = 9.7 Hz, 1H).

6. Gram scale preparation of 3 and synthetic application

6.1 Gram scale preparation of 3aa

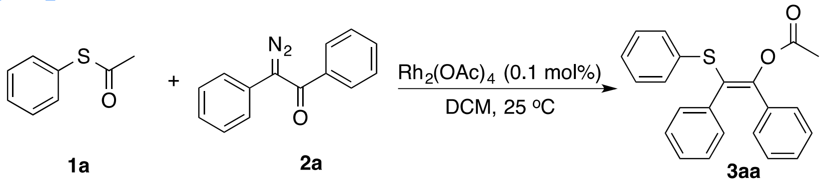

1a **2a** **3aa**
1441 mg, 83%

Thioester **1a** (1125.0 mg, 7.4 mmol, 1.5 equiv), and $Rh_2(OAc)_4$ (2.21 mg, 0.005 mmol, 0.1 mol%) were introduced into a dried glass tube under N_2 protection, and add 2 mL dry DCM as solvent, then the diazoketone **2a** (1110.0 mg, 5.0 mmol, 1.0 equiv) was dissolved in 8 ml of DCM and add dropwise in 30 min at room temperature. After the addition, continue to react for 15 minute consumed diazo completely determined by TLC analysis. The mixture was purified by column chromatography on silica gel using PE/EtOAc (30:1) as the eluent and concentrated to obtain the product **3aa** (1141.0 mg, 83%).

6.2 Gram scale preparation of 3da

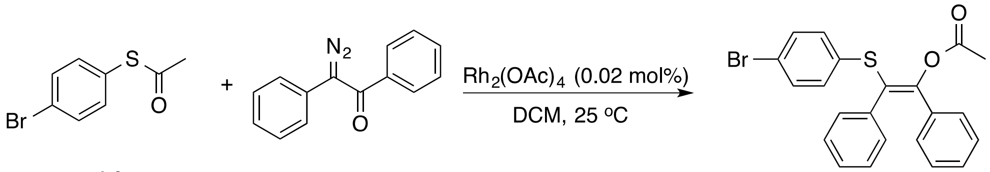

1d
(6.24 g, 27 mmol)

2a
(5.00 g, 22.5 mmol)

3da
first recrystallization: 6988 mg, 73%

Thioester **1d** (6.24 g, 27 mmol, 1.2 equiv) and $Rh_2(OAc)_4$ (2.0 mg, 0.0045 mmol, 0.02 mol%,) were introduced into a 100mL dried Schlenk tube under N_2 protection, and add 12mL dry DCM as solvent, then the diazoketone **2a** (5.0 g, 22.5 mmol, 1.0 equiv) was dissolve in five-times into a total of 15 mL dry DCM and add dropwise in 60min at room temperature (At the beginning of dripping **2a**, the bubbles were not obvious. TLC analysis monitors the reaction system during the reaction. After about 2.0 g **2a** is added dropwise, a large amount of gas will continue to be generated until the reaction is complete.) After the addition, continue to react for 15 minute consumed diazo completely determined by TLC analysis. A light-yellow solid can be obtained after directly concentrating the solvent (Figure S1 A). The crude product was dissolved in 20 mL of DCM, and then 300 mL of methanol was added to recrystallize at 4°C. After 2 hours, a large amount of solid precipitated (Figure S1 B). After filtration, the solid was washed twice with cold methanol and after vacuum drying to obtain a white solid **3da** (6988.9 mg, 73%, Figure S1 C, D).

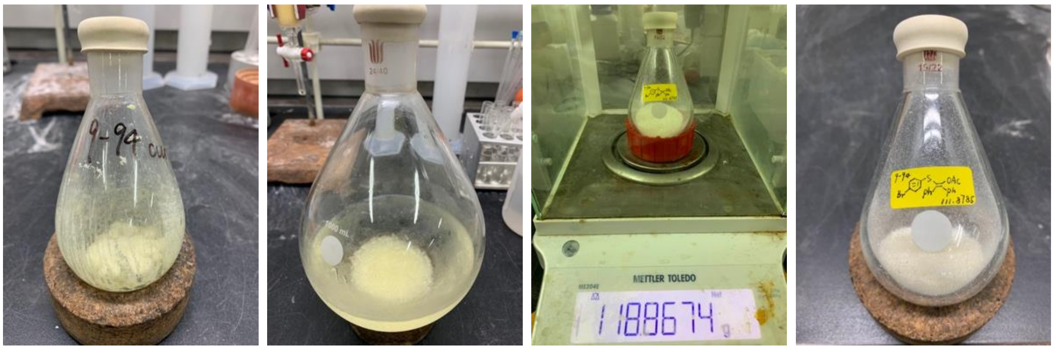

A B C D

Supplementary Figure 1. Gram scale preparation of **3da**

6.3 Synthetic application

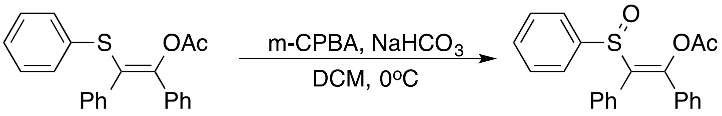

3aa **12**

3aa (173.1 mg, 0.5 mmol, 1.0 equiv) and $NaHCO_3$ (42.0 mg, 0.5 mmol, 1.0 equiv) were introduced into a 25mL flask. Add DCM (2 mL) as a solvent, stir at 0°C. m-CPBA (75%, 139.0 mg, 0.6 mmol, 1.2 equiv) dissolved in 2 mL of DCM was added dropwise to the reaction at 0°C. Consumed completely determined by TLC analysis (R_f = 0.26, PE/EtOAc = 5:1). Add 10 mL of H_2O, extract three times with DCM, wash the combined organic layer twice with $NaHCO_3$ solution, dry with Na_2SO_4 and filter, then concentrate the solvent and the residue was purified by column chromatography on silica gel using PE/EtOAc (20:1 to 5:1) as the eluent and concentrated to obtain the product **12** (146.1 mg, 81%) as white solid; m.p. = 124 – 126 ℃; ^{1}H NMR (500 MHz, $CDCl_3$) δ 7.44 – 7.38 (m, 2H), 7.37 – 7.31 (m, 3H), 7.22 – 7.16 (m, 4H), 7.15 – 7.08 (m, 4H), 6.82 (d, J = 7.6 Hz, 2H), 2.41 (s, 3H); ^{13}C NMR (125 MHz, $CDCl_3$) δ 168.8, 149.9, 141.8, 136.2, 132.7, 131.1, 130.5, 129.6, 128.5, 128.5, 128.4, 128.3, 128.0, 127.7, 124.4, 20.7; HRMS (ESI-TOF) m/z: $[M+Na]^+$ calculated for $C_{22}H_{18}NaO_3S$ 385.0874, found 385.0858.

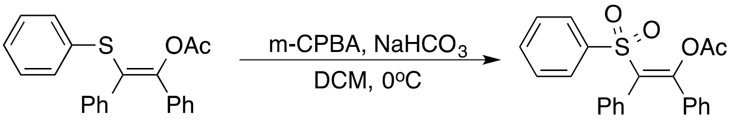

3aa **13**

3aa (173.1 mg, 0.5 mmol, 1.0 equiv) and $NaHCO_3$ (42.0 mg, 0.5 mmol, 1.0 equiv) were introduced into a 25mL flask. Add DCM (2 mL) as a solvent, stir at 0°C. m-CPBA (75%, 575.3 mg, 2.5 mmol, 5.0 equiv) dissolved in 5 mL of DCM was added dropwise to the reaction at 0°C. Consumed completely determined by TLC analysis (R_f = 0.30, PE/EtOAc = 5:1). Add 10 mL of H_2O, extract three times with DCM, wash the combined organic layer twice with $NaHCO_3$ solution, dry with Na_2SO_4 and filter, then concentrate the solvent and the residue was purified by column chromatography on silica gel using PE/EtOAc (20:1 to 5:1) as the eluent and concentrated to obtain the product **13** (122.7 mg, 65%) as white solid; m.p. = 170

– 172 ℃; ^{1}H NMR (500 MHz, $CDCl_3$) δ 7.75 (d, *J* = 7.0 Hz, 2H), 7.57 (t, *J* = 7.4 Hz, 1H), 7.45 (t, *J* = 7.8 Hz, 2H), 7.28 – 7.24 (m, 2H), 7.21 – 7.15 (m, 3H), 7.15 – 7.07 (m, 6H), 2.35 (s, 3H); ^{13}C NMR (125 MHz, $CDCl_3$) δ 168.3, 153.9, 140.8, 133. 7, 133.3, 132.8, 132.2, 131.0, 129.9, 128.9, 128.8, 128.7, 128.2, 128.1, 128.0, 21.2; HRMS (ESI-TOF) m/z: $[M+Na]^+$ calculated for $C_{22}H_{18}NaO_4S$ 401.0823, found 401.0806.

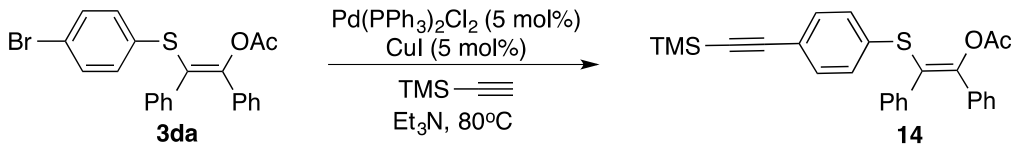

3da (212.0 mg, 0.5 mmol, 1.0 equiv), $Pd(PPh_3)_2Cl_2$ (18.0 mg, 0.025 mmol, 5 mol%), CuI (5.0 mg, 0.025 mmol, 5 mol%) and trimethylsilylacetylene (98.2 mg, 1.0 mmol, 2.0 equiv) were introduced into a 25mL dried Schlenk tube under N_2 protection. Then add 2mL dry Et_3N as solvent and react at 80°C for 3 hours. Consumed completely determined by TLC analysis (R_f = 0.52, PE/EtOAc = 20:1). Concentrate the solvent and then the mixture was purified by column chromatography on silica gel using PE/EtOAc (30:1) as the eluent and concentrated to obtain the product **14** (210.1 mg, 95%) as white solid; m.p. = 135 – 136 ℃; ^{1}H NMR (500 MHz, $CDCl_3$) δ 7.23 – 7.13 (m, 1H), 7.07 – 7.01 (m, 3H), 2.27 (s, 3H), 0.20 (s, 9H); ^{13}C NMR (125 MHz, $CDCl_3$) δ 169.1, 147.1, 135.7, 135.0, 134.2, 132.0, 130.8, 130.7, 128.8, 128.4, 128.0, 127.9, 127.8, 126.5, 121.2, 104.6, 94.9, 20.9, -0.11; HRMS (ESI-TOF) m/z: $[M+Na]^+$ calculated for $C_{27}H_{26}NaO_2SSi$ 465.1320, found 465.1316.

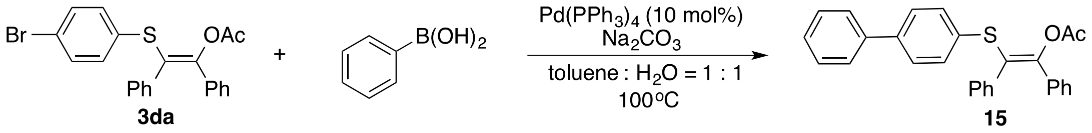

3da (825.2 mg, 1.94 mmol, 1.0 equiv), $Pd(PPh_3)_4$ (224.0 mg, 0.194 mmol, 10 mol%), Na_2CO_3 (411.0 mg, 3.88 mmol, 2.0 equiv) and phenylboronic acid (355.0 mg, 2.91 mmol, 1.5 equiv) were introduced into a 50mL dried Schlenk tube under N_2 protection. Then add 16mL solvent (toluene : H_2O = 1:1) and react at 100°C for 8 hours. Consumed completely determined by TLC analysis (R_f = 0.43, PE/EtOAc = 20:1). Concentrate the solvent and then the mixture was purified by column chromatography on silica gel using PE/EtOAc (20:1) as the eluent and concentrated to obtain the product **15** (660.0 mg, 58%) as white solid; m.p. = 102 – 104 ℃; ^{1}H NMR (600 MHz, $CDCl_3$) δ 7.49 – 7.44 (m, 2H), 7.40 – 7.23 (m, 9H), 7.21 – 7.11 (m, 5H), 7.10 – 7.00 (m, 3H), 2.27 (s, 3H); ^{13}C NMR (150 MHz, $CDCl_3$) δ 169.1, 146.6, 140.2, 139.6, 136.0, 135.1, 132.2, 131.7, 130.8, 128.8, 128.7, 128.2, 127.9, 127.7, 127.3, 127.1, 127.0, 126.8, 20.9; HRMS (ESI-TOF) m/z: $[M+Na]^+$ calculated for $C_{28}H_{22}NaO_2S$ 445.1238, found 445.1225.

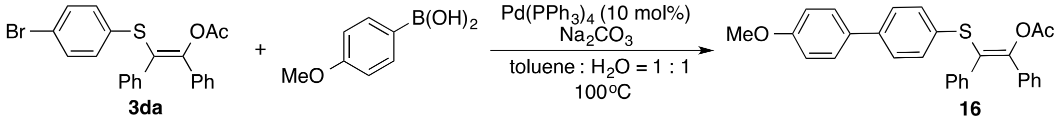

3da (212.0 mg, 0.5 mmol, 1.0 equiv), $Pd(PPh_3)_4$ (58.0 mg, 0.05 mmol, 10 mol%), Na_2CO_3 (106.0 mg, 1.0 mmol, 2.0 equiv) and 4-Methoxyphenylboronic acid (114.0 mg, 0.75 mmol, 1.5 equiv) were introduced into a 25mL dried Schlenk tube under N_2 protection. Then add 2 mL solvent (toluene : H_2O = 1:1) and react at 100°C for 3 hours. Consumed completely determined by TLC analysis (R_f = 0.24, PE/EtOAc = 20:1). Concentrate the solvent and then the mixture was purified by column chromatography on silica gel using PE/EtOAc (10:1) as the eluent and concentrated to obtain the product **16** (197.0 mg, 87%) as white solid; m.p. = 109 - 111 ℃; ^{1}H NMR (600 MHz, $CDCl_3$) δ 7.40 (d, J = 8.7 Hz, 2H), 7.33 – 7.23 (m, 6H), 7.20 – 7.10 (m, 5H), 7.08 – 7.00 (m, 3H), 6.90 (d, J = 8.7 Hz, 2H), 3.80 (s, 3H), 2.28 (s, 3H); ^{13}C NMR (150 MHz, $CDCl_3$) δ 169.1, 159.2, 146.4, 139.2, 136.0, 135.1, 132.7, 131.9, 131.2, 130.84, 128.75, 128.2, 127.9, 127.8, 127.6, 127.2, 126.7, 114.1, 55.3, 20.9; HRMS (ESI-TOF) m/z: $[M+Na]^+$ calculated for $C_{29}H_{24}NaO_3S$ 475.1344, found 475.1340.

7. Crossover experiment

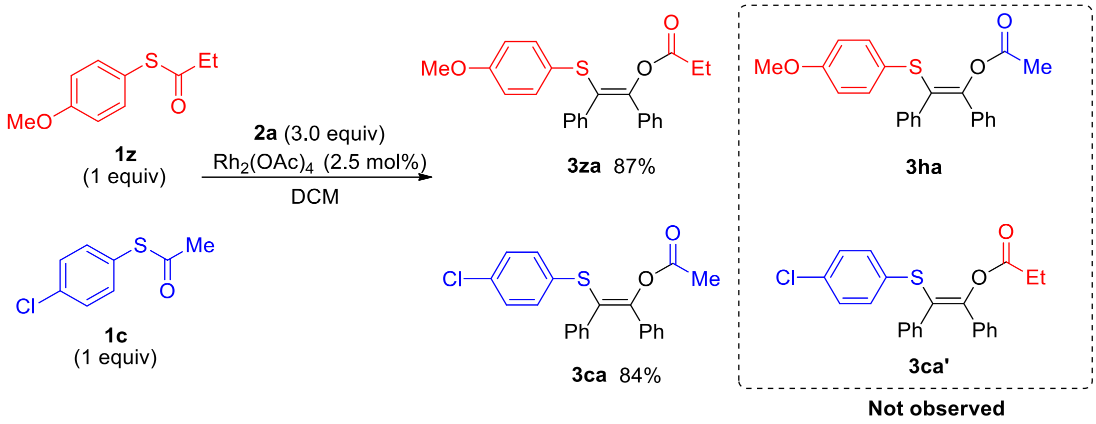

1c (93.3 mg, 0.5 mmol, 1.0 equiv), **1z** (98.1 mg, 0.5 mmol, 1.0 equiv), $Rh_2(OAc)_4$ (11.0 mg, 0.025 mmol, 2.5 mol%) were introduced into a dried Schlenk tube under N_2 protection, and add 2 mL dry DCM as solvent, then the diazoketone **2a** (333.5 mg, 1.5 mmol, 3.0 equiv) was dissolved in 2mL dry DCM and add dropwise in 10 mins at room temperature. After the addition, continue to react for 1 minute consumed diazo completely determined by TLC analysis. The mixture was purified by column chromatography on silica gel using ethyl acetate/petroleum ether (1:30 to 1:10) as the eluent. Collect the product **3ca** (160.0 mg, 84%) eluting at ethyl acetate/petroleum ether =1:30, and the product **3za** (169.0 mg, 87%) eluting at ethyl acetate/petroleum ether =1:10. Compounds **3ha** and **3ca'** are not detected by crude ^{1}H NMR.

8. DFT Calculation

To shed light on the mechanism of the title reaction, density functional theory (DFT) calculations were then carried out with the Gaussian 09 software package.[23-26] The geometry optimizations were carried out with M062X method and combined basis set.[27] That is, 6-31G(d) for C, H, O, N, S atoms and SDD for Rh atom.[28,29] Harmonic vibrational frequency calculations were performed for all of the stationary points to determine whether they are local minima or transition structures. All the energies discussed in the text are the relative Gibbs free energies (GFE), which are obtained by the addition of the thermal Gibbs free energy corrections (TGC) at the M062X/6-31G(d)/SDD level and the single-point energies (SPE) at a higher computational level of M062X/6-31+G(d,p)/SDD. Intrinsic reaction coordinate calculations were performed to ensure every TS is connect with corresponding equilibrium structure. Truhlar and coworkers' SMD solvation model was employed to consider the solvent effect of dichloromethane (ε=8.93).[30]

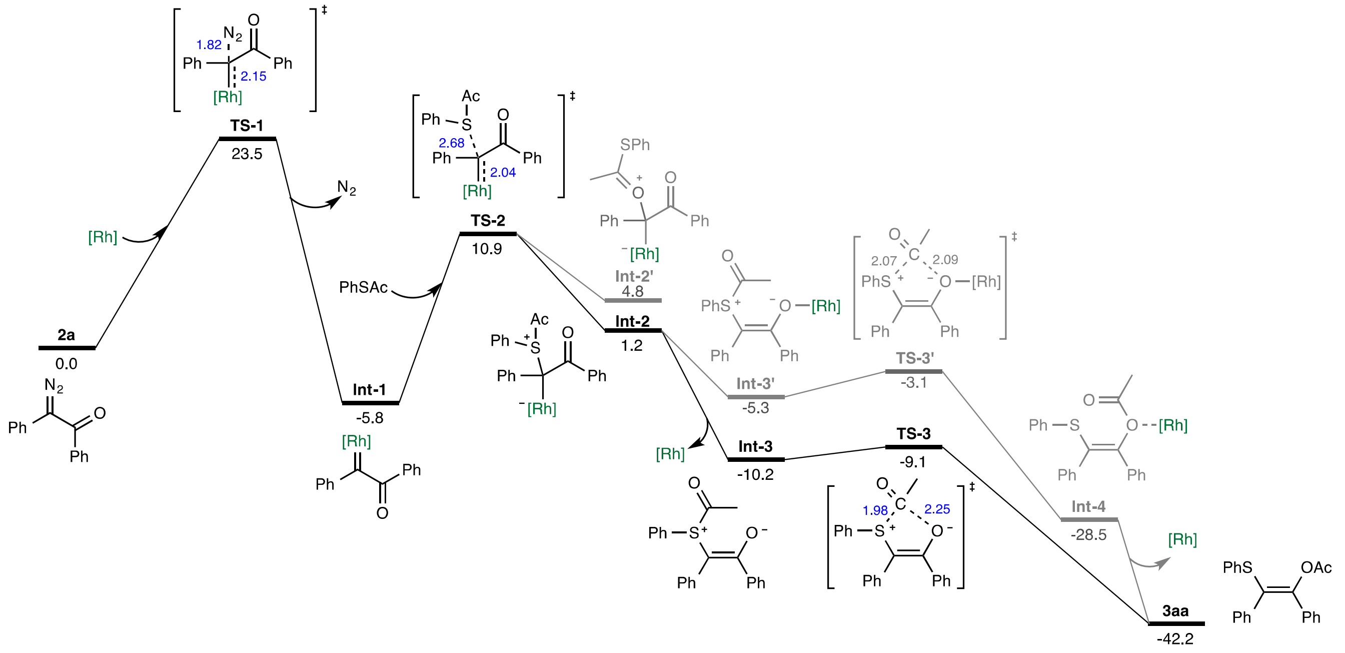

Supplementary Figure 2. Free-energy reaction profiles (kcal mol^{-1}) for the reaction of **2a** and **1a**. [Rh] = $Rh_2(OAc)_4$.

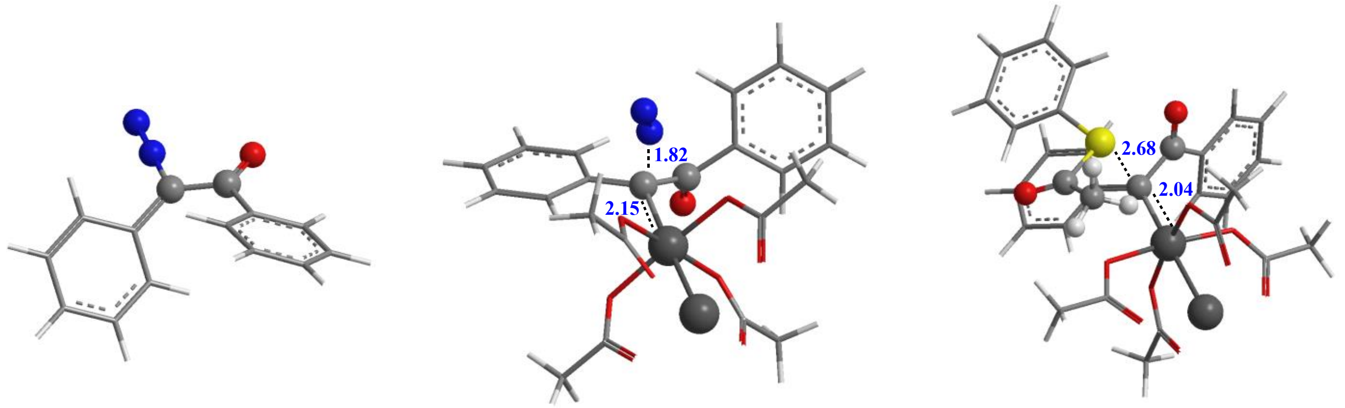

2a **TS-1** **TS-2**

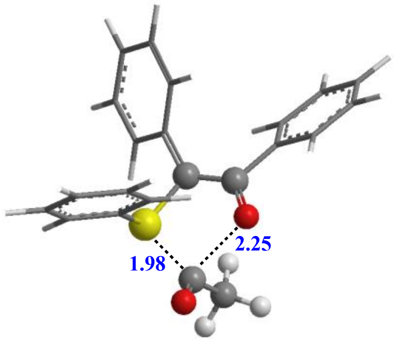

TS-3

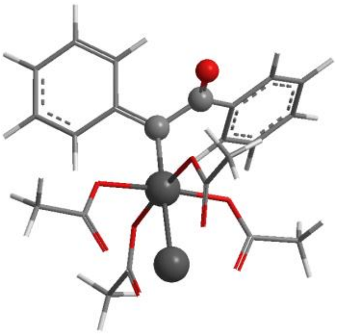

Int-1

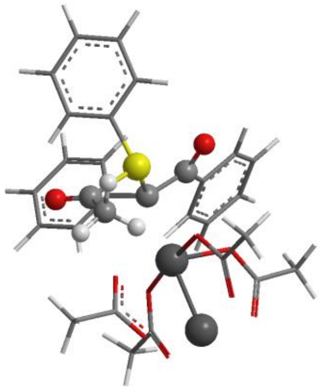

Int-2

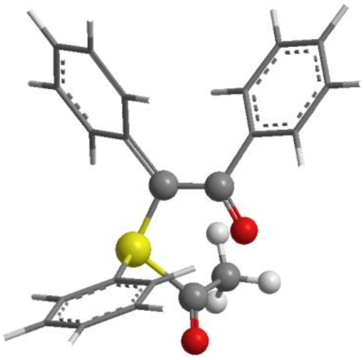

Int-3

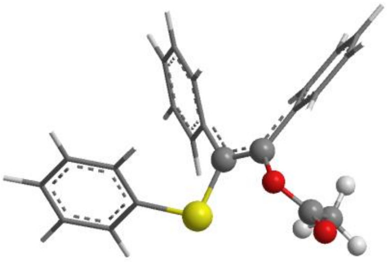

3aa

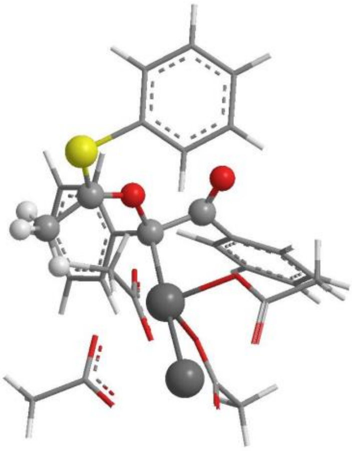

Int-2'

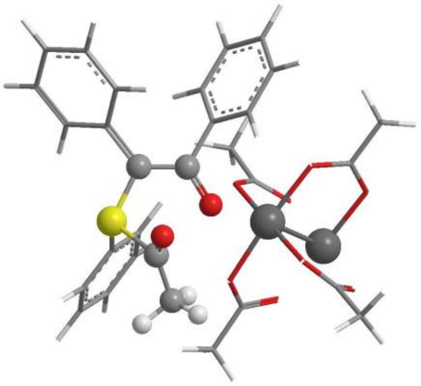

Int-3'

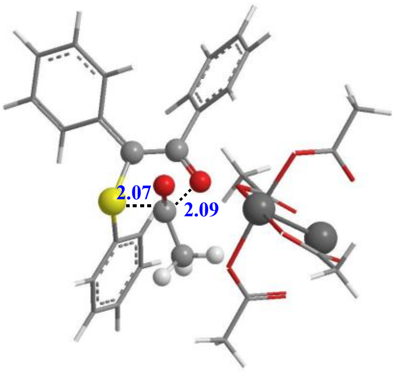

TS-3'

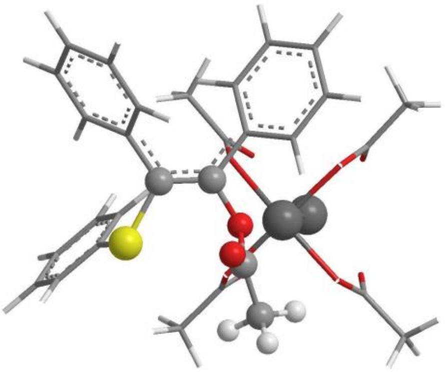

Int-4

The reaction of **2a** and *S*-phenyl ethanethioate **1a** is selected as the model reaction. As illustrated in Scheme S1, the reaction path includes formation of Rhodium carbene **Int-1**, then generation of free sulfur ylide **Int-3** via a metal-bound ylide intermediate **Int-2**, and finally [1,4]-shift of acetyl group to form product **3aa**, in which the formation of Rhodium carbene **Int-1** is the determining step. The barrier is 23.5 kcal/mol, which means this reaction can proceed smoothly at room temperature. This is in good line with the experiment. The shift process is almost barrierless, with a barrier of only 1.1 kcal/mol (Scheme S1).

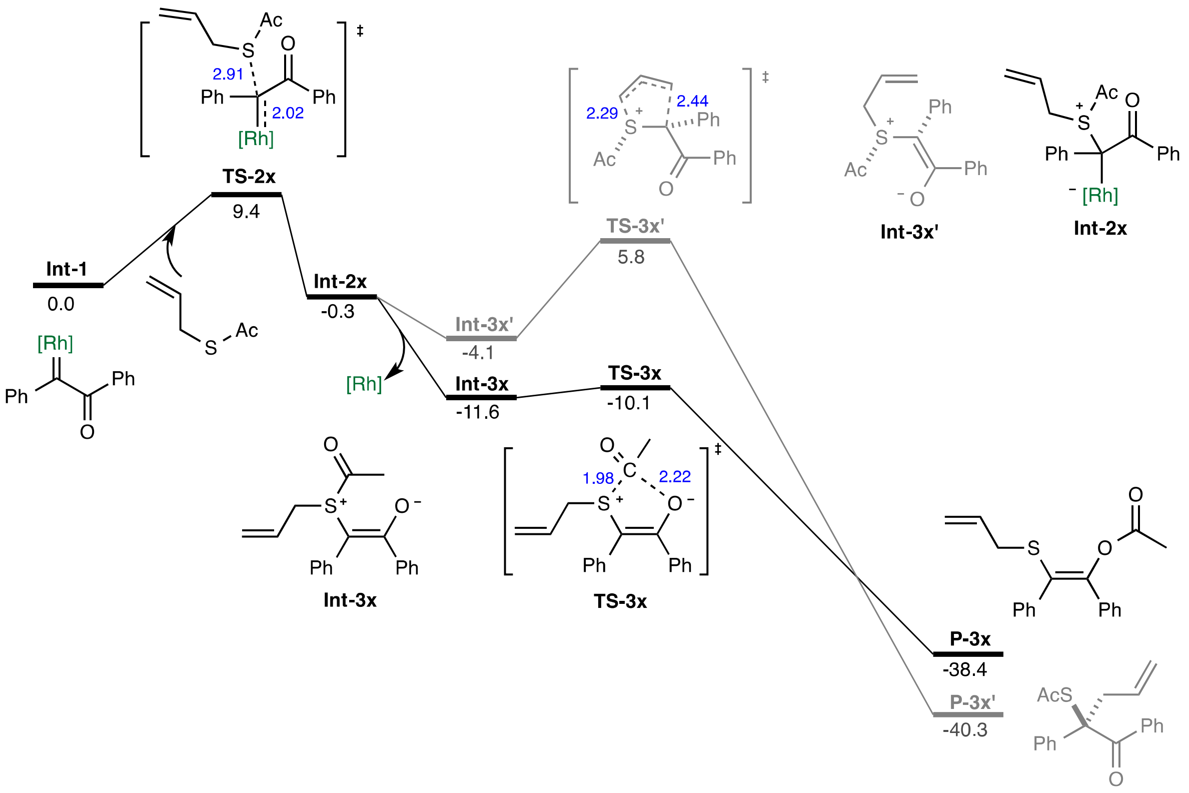

Supplementary Figure 3. Free-energy reaction profiles (kcal mol^{-1}) for the reaction of **2a** and **1x**. [Rh] = $Rh_2(OAc)_4$.

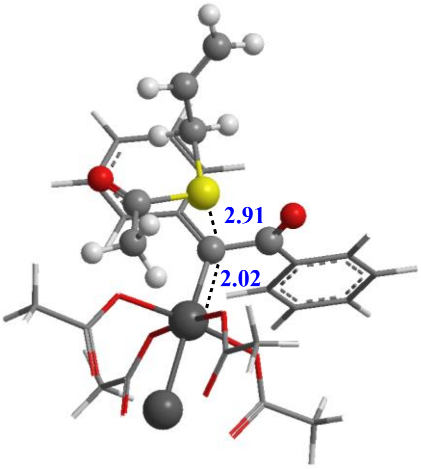

TS-2x

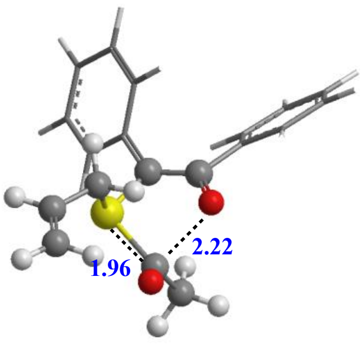

TS-3x

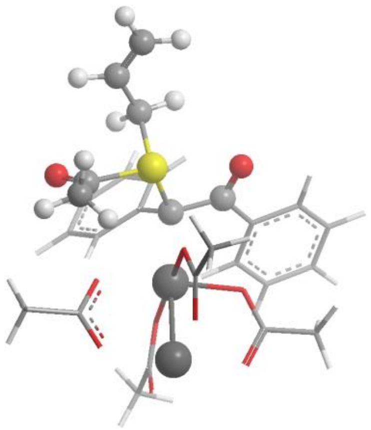

Int-2x

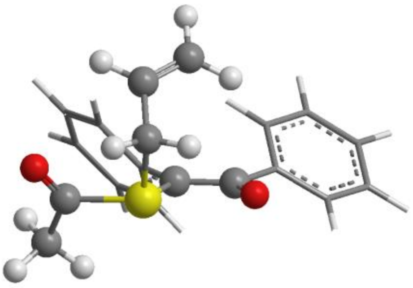

Int-3x

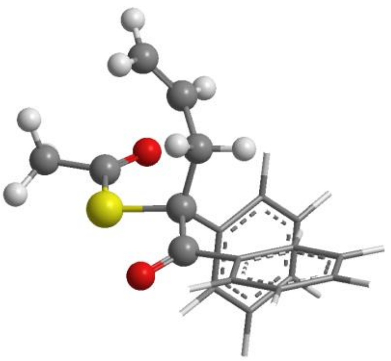

Int-3x'

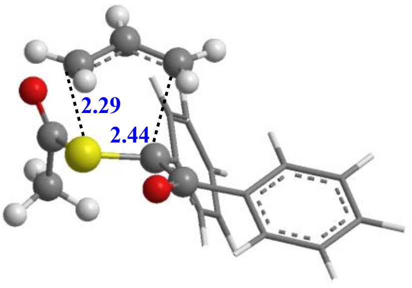

TS-3x'

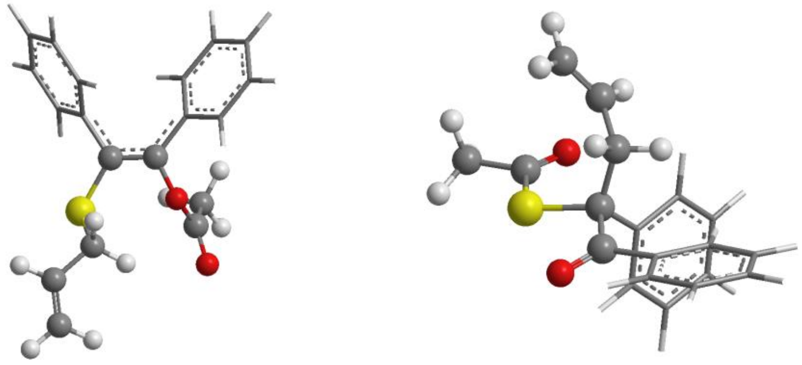

P-3x **P-3x'**

When *S*-allyl ethanethioate **1x** was used as reactans, similar reaction paths can proceeds as well to form products **P-3x** (Scheme S2). Meanwhile, [2,3]-sigmatropic rearrangements of sulfur ylide **Int-3x** was also possible to generate **P-3x‘** (in red). First, **Int-3x** is transformed to a unstable conformation **Int-3x’**, then the rearrangement proceeds via 5-membered ring transition state **TS-3x’**. Since the transformation from **Int-3x** to **P-3x** is almost barrierless, while the barrier of [2,3]-sigmatropic rearrangement is up to 17.4 kcal/mol, [2,3]-sigmatropic rearrangement should not be observed in this reaction. The reaction of *S*-prop-2-ynyl ethanethioate 1y and 2a was carried out via the similar path (Scheme S3).

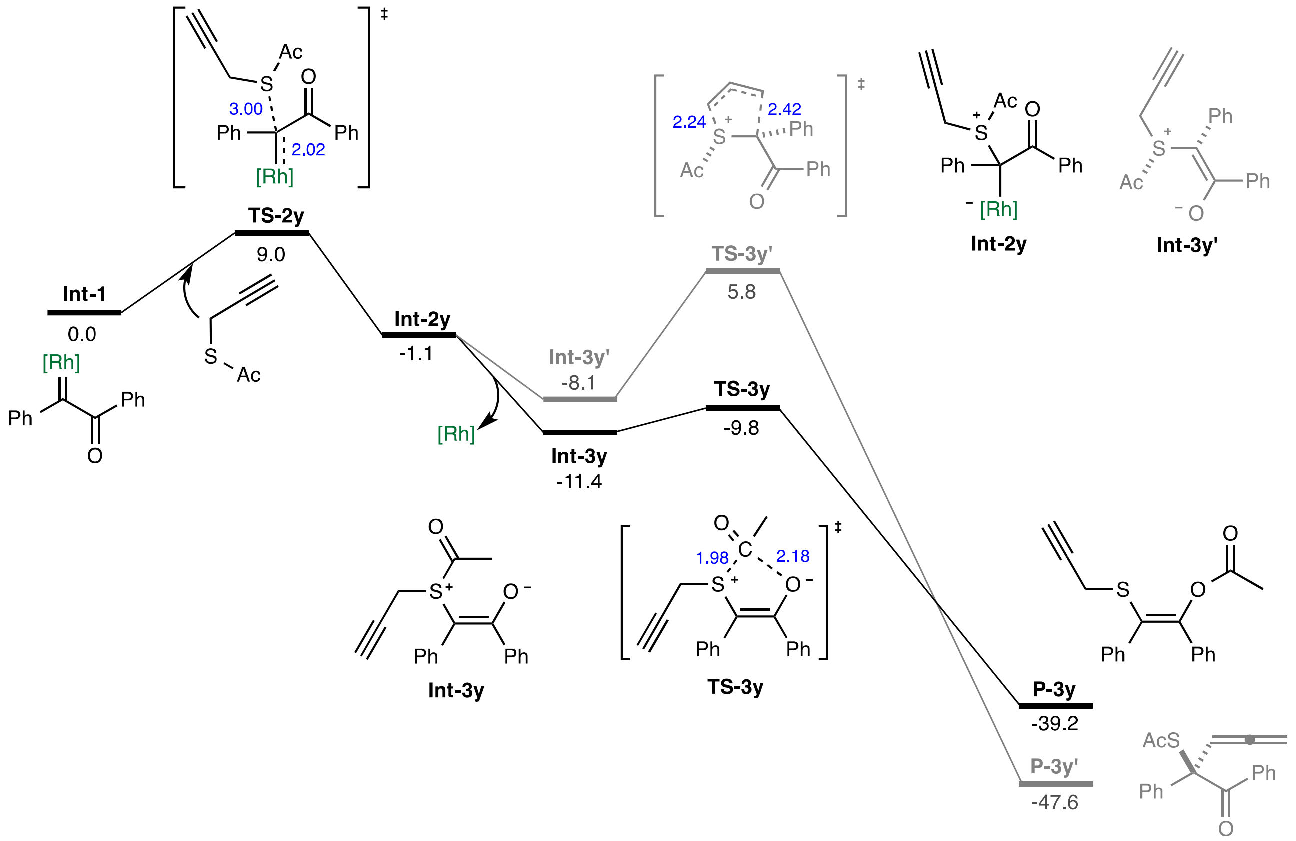

Supplementary Figure 4. Free-energy reaction profiles (kcal mol^{-1}) for the reaction of **2a** and **1y**. [Rh] = $Rh_2(OAc)_4$.

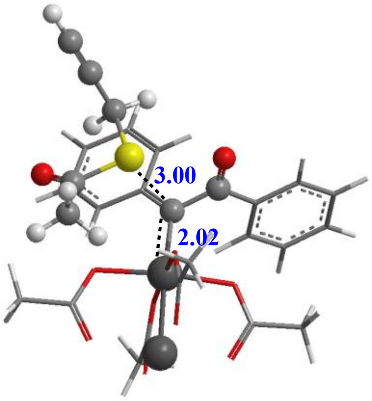

TS-2y

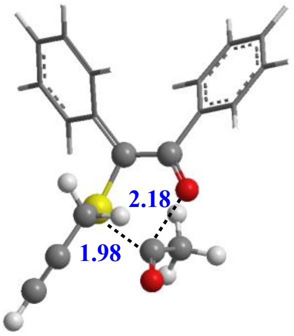

TS-3y

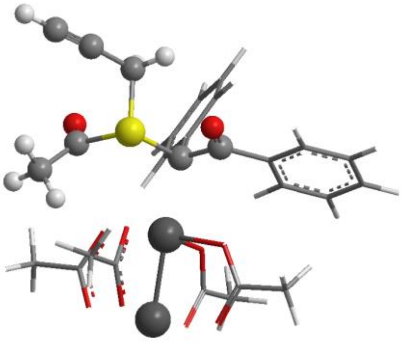

Int-2y

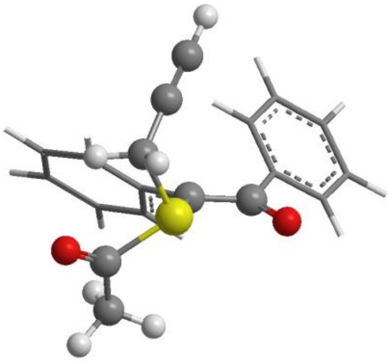

Int-3y

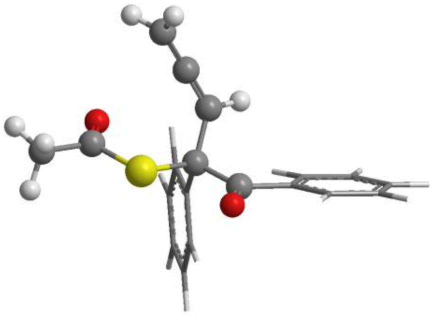

Int-3y'

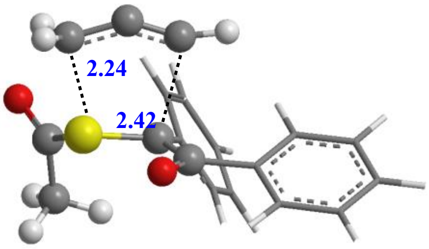

TS-3y'

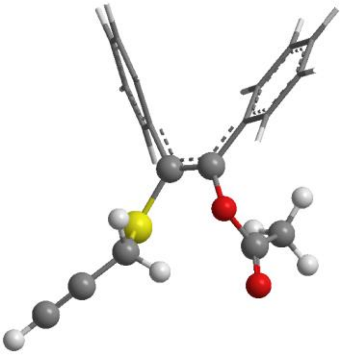

P-3y

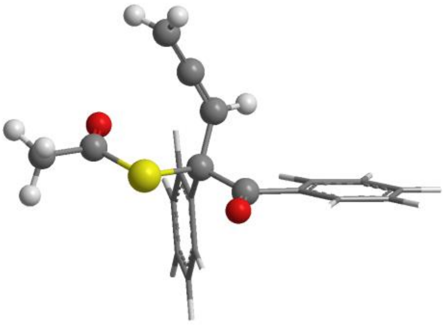

P-3y'

Supplementary Table 2. TCG, Absolute SPE and GFE of the optimized structures.

Optimized structures.	TCG(a.u.)	SPE(a.u.)	GFE(a.u.)
**2a**	0.166810	-723.733248	-723.566438
**$Rh_2(OAc)_4$**	0.164003	-1134.445919	-1134.281916
**TS-1**	0.352274	-1858.163227	-1857.810953
**Int-1**	0.346652	-1748.710501	-1748.363849
**N_2**	-0.012671	-109.481118	-109.493789
**PhSAc**	0.104833	-782.777626	-782.672793
**TS-2**	0.478221	-2531.488245	-2531.010024
**Int-2**	0.479034	-2531.504535	-2531.025501
**Int-3**	0.288089	-1397.049831	-1396.761742
**TS-3**	0.289770	-1397.049712	-1396.759942
**3aa**	0.289763	-1397.102436	-1396.812673
**Int-2'**	0.478872	-2531.514662	-2531.035790
**Int-3'**	0.479734	-2531.512053	-2531.032319
**TS-3'**	0.481256	-2531.554048	-2531.072792
**Int-4**	0.482536	-2532.056921	-2532.056921
**1x**	0.086255	-668.489132	-668.402877
**TS-2x**	0.458435	-2417.200953	-2416.742518
**Int-2x**	0.461665	-2417.219533	-2416.757868
**Int-3x**	0.269772	-1282.763812	-1282.494040
**TS-3x**	0.271022	-1282.762697	-1282.491675
**P-3x**	0.270624	-1282.807357	-1282.536733
**Int-3x'**	0.273039	-1282.755121	-1282.482082
**TS-3x'**	0.272436	-1282.738755	-1282.466319
**P-3x'**	0.272932	-1282.812634	-1282.539702
**1y**	0.062659	-667.265339	-667.202680
**TS-2y**	0.434907	-2415.977770	-2415.542863
**Int-2y**	0.437294	-2415.996329	-2415.559035
**Int-3y**	0.246185	-1281.539733	-1281.293548
**TS-3y**	0.247592	-1281.538535	-1281.290943
**P-3y**	0.246026	-1281.583847	-1281.337821
**Int-3y'**	0.245143	-1281.533444	-1281.288301
**TS-3y'**	0.247364	-1281.513405	-1281.266041
**P-3y'**	0.247817	-1281.598948	-1281.351131

9. X-ray crystal data for 3aa, 3aw, 11c, 11d

9.1 Crystal Structure Information of 3aa

0.1 mL of DCM was added to a 10mL oven-dried glass sample bottle with 10 mg pure **3aa** to dissolve the sample, then 5mL *n*-hexane was slowly added to the solution, sealed with perforated paper, and then the solvent was slowly dried at room temperature to obtain crystals. Single crystal X-ray diffraction data were collected on Bruker Smart Apex II CCD diffractometer. The crystal structure has been deposited at the Cambridge Crystallographic Data Centre (CCDC)：2033240

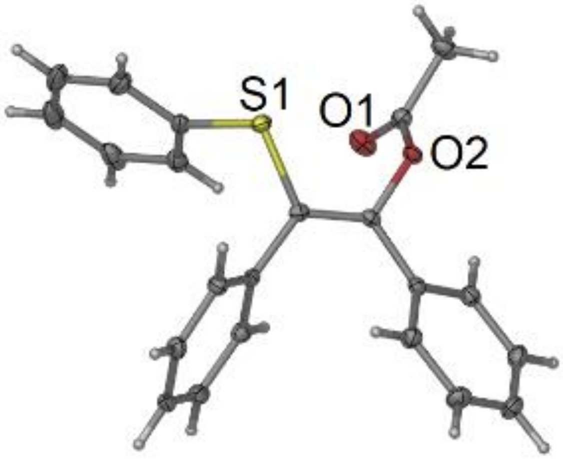

Supplementary Figure 5. The structure of the **3aa**.

Supplementary Table 3. Crystal data for compound **3aa**

Compound	**3aa**
Empirical formula	$C_{22}H_{18}O_2S$
Formula weight	346.42
Temperature/K	100.02(10)
Crystal system	monoclinic
Space group	$P2_1/c$
a/Å	11.28450(10)
b/Å	19.22880(10)
c/Å	9.01840(2)
α/°	90
β/°	112.8910(10)
γ/°	90
Volume/Å^3	1802.77(3)
Z	4
ρ_{calc}g/cm^3	1.276
μ/mm^{-1}	1.679
F(000)	728.0
Crystal size/mm^3	0.42 × 0.36 × 0.34
Radiation	CuKα (λ = 1.54184)
2Θ range for data collection/°	8.506 to 134.146
Index ranges	$-13 \leq h \leq 13$, $-22 \leq k \leq 22$, $-10 \leq l \leq 10$
Reflections collected	40896
Independent reflections	3217 [R_{int} = 0.0408, R_{sigma} = 0.0137]
Data/restraints/parameters	3217/0/227
Goodness-of-fit on F^2	1.112
Final R indexes [I>=2σ (I)]	R_1 = 0.0333, wR_2 = 0.0825
Final R indexes [all data]	R_1 = 0.0334, wR_2 = 0.0826
Largest diff. peak/hole / e Å^{-3}	0.17/-0.33

9.2 Crystal Structure Information of 3aw

0.1 mL of DCM was added to a 10mL oven-dried glass sample bottle with 10 mg pure **3aw** to dissolve the sample, then 5mL *n*-hexane was slowly added to the solution, sealed with perforated paper, and then the solvent was slowly dried at room temperature to obtain crystals. Single crystal X-ray diffraction data were collected on Bruker Smart Apex II CCD diffractometer. The crystal structure has been deposited at the Cambridge Crystallographic Data Centre (CCDC)：2033239

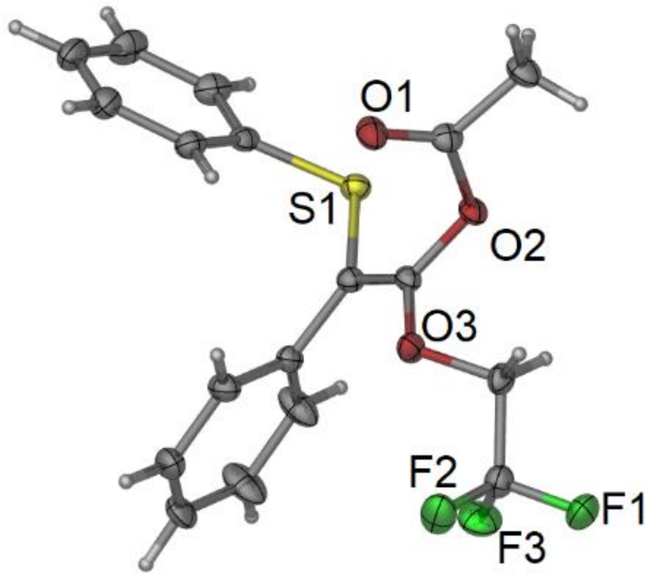

Supplementary Figure 6. The structure of the **3aw**.

Supplementary Table 4. Crystal data for compound **3aw**

Compound	**3aw**
Empirical formula	$C_{18}H_{15}F_3O_3S$
Formula weight	368.36
Temperature/K	100.00(10)
Crystal system	monoclinic
Space group	$P2_1/c$
a/Å	10.2572(2)
b/Å	18.8710(3)
c/Å	9.2459(2)
α/°	90
β/°	108.736(3)
γ/°	90
Volume/Å^3	1694.83(6)
Z	4
ρ_{calc}g/cm^3	1.444
μ/mm^{-1}	2.125
F(000)	760.0
Crystal size/mm^3	0.33 × 0.15 × 0.11
Radiation	CuKα (λ = 1.54184)
2Θ range for data collection/°	9.104 to 152.15
Index ranges	$-12 \leq h \leq 12$, $-23 \leq k \leq 23$, $-11 \leq l \leq 10$
Reflections collected	40501
Independent reflections	3444 [R_{int} = 0.1035, R_{sigma} = 0.0513]
Data/restraints/parameters	3444/0/227
Goodness-of-fit on F^2	1.058
Final R indexes [I>=2σ (I)]	R_1 = 0.0432, wR_2 = 0.1102
Final R indexes [all data]	R_1 = 0.0510, wR_2 = 0.1158
Largest diff. peak/hole / e Å^{-3}	0.31/-0.35

9.3 Crystal Structure Information of 11c

0.1 mL of DCM was added to a 10mL oven-dried glass sample bottle with 10 mg pure **11c** to dissolve the sample, then 5mL *n*-hexane was slowly added to the solution, sealed with perforated paper, and then the solvent was slowly dried at room temperature to obtain crystals. Single crystal X-ray diffraction data were collected on Bruker Smart Apex II CCD diffractometer. The crystal structure has been deposited at the Cambridge Crystallographic Data Centre (CCDC)：2033243

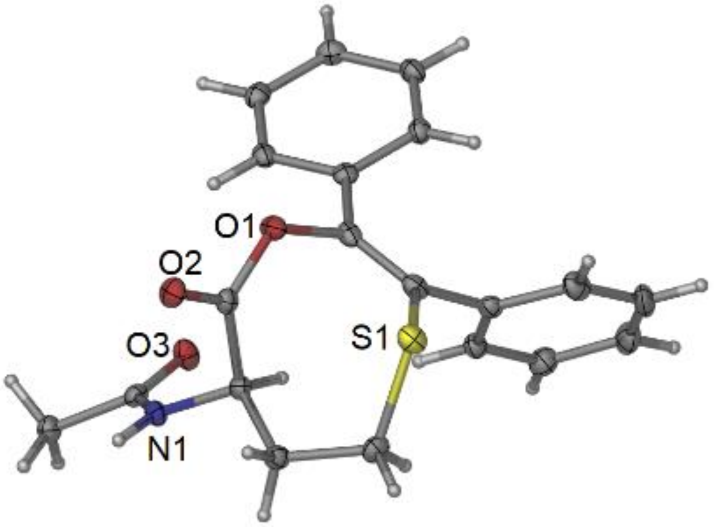

Supplementary Figure 7. The structure of the **11c**.

Supplementary Table 5. Crystal data for compound **11c**

Compound	**11c**
Empirical formula	$C_{20}H_{19}NO_3S$
Formula weight	353.42
Temperature/K	100.00(10)
Crystal system	monoclinic
Space group	Cc
a/Å	8.37910(10)
b/Å	28.1144(2)
c/Å	7.70870(10)
α/°	90
β/°	105.9220(10)
γ/°	90
Volume/Å^3	1746.30(3)
Z	4
ρ_{calc}g/cm^3	1.344
μ/mm^{-1}	1.802
F(000)	744.0
Crystal size/mm^3	0.42 × 0.26 × 0.18
Radiation	CuKα (λ = 1.54184)
2Θ range for data collection/°	11.424 to 134.086
Index ranges	-9 ≤ h ≤ 9, -33 ≤ k ≤ 33, -9 ≤ l ≤ 8
Reflections collected	16339
Independent reflections	2946 [R_{int} = 0.0296, R_{sigma} = 0.0182]
Data/restraints/parameters	2946/2/227
Goodness-of-fit on F^2	1.049
Final R indexes [I>=2σ (I)]	R_1 = 0.0207, wR_2 = 0.0544
Final R indexes [all data]	R_1 = 0.0208, wR_2 = 0.0544
Largest diff. peak/hole / e Å^{-3}	0.16/-0.14
Flack parameter	0.003(6)

9.4 Crystal Structure Information of 11d

0.1 mL of DCM was added to a 10mL oven-dried glass sample bottle with 10 mg pure **11d** to dissolve the sample, then 5mL *n*-hexane was slowly added to the solution, sealed with perforated paper, and then the solvent was slowly dried at room temperature to obtain crystals. Single crystal X-ray diffraction data were collected on Bruker Smart Apex II CCD diffractometer. The crystal structure has been deposited at the Cambridge Crystallographic Data Centre (CCDC)：2033242

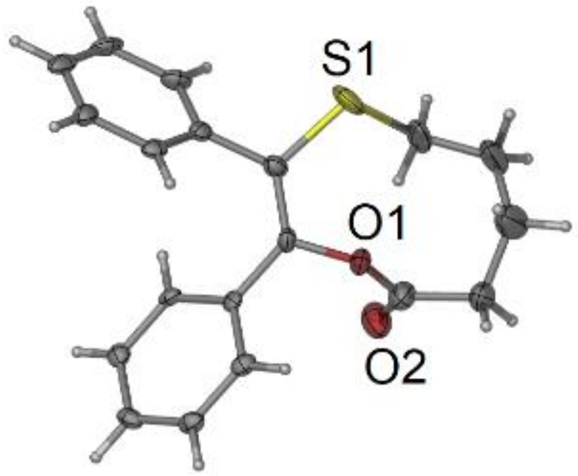

Supplementary Figure 8. The structure of the **11d**.

Supplementary Table 6. Crystal data for compound **11d**

Compound	**11d**
Empirical formula	$C_{19}H_{18}O_2S$
Formula weight	310.39
Temperature/K	100.00(10)
Crystal system	orthorhombic
Space group	$Pna2_1$
a/Å	14.61460(10)
b/Å	19.00140(10)
c/Å	5.64700(10)
α/°	90
β/°	90
γ/°	90
Volume/Å^3	1586.16(3)
Z	4
ρ_{calc}g/cm^3	1.315
μ/mm^{-1}	1.862
F(000)	656.0
Crystal size/mm^3	0.38 × 0.32 × 0.26
Radiation	CuKα (λ = 1.54184)
2Θ range for data collection/°	7.632 to 134.016
Index ranges	$-17 \le h \le 17$, $-22 \le k \le 22$, $-6 \le l \le 6$
Reflections collected	32181
Independent reflections	2763 [R_{int} = 0.0486, R_{sigma} = 0.0202]
Data/restraints/parameters	2763/34/199
Goodness-of-fit on F^2	1.025
Final R indexes [I>=2σ (I)]	R_1 = 0.0727, wR_2 = 0.1955
Final R indexes [all data]	R_1 = 0.0732, wR_2 = 0.1959
Largest diff. peak/hole / e Å^{-3}	1.13/-0.71
Flack parameter	0.014(9)

10. Photophysical properties of products

After developing a method for the synthesis of sulfur-containing tetra-substituted olefins, we investigated their AIE performance, the corresponding results are shown in **Figure S21-S40**. The photophysical properties of representative compounds are summarized in **Table S6** and the corresponding spectra are given.

Supplementary Table 7. Photophysical data of representative AIE-gens[a]

Compound	Solution [nm]			Solid		α_{AIE}[b]	Photo images[c]
	λ_{abs}	λ_{em}	Stoke's shift	λ_{em} [nm]	τ_F (μs)		
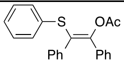 3aa	313	426	113	401	6.1	4.52	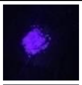
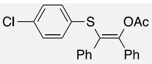 3ca	312	426	114	407<br>637	4.9<br>534	2.60	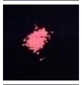
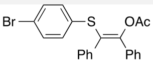 3da	315	425	110	402<br>640	5.5<br>479	2.91	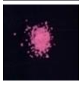
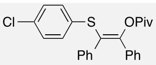 3sa	314	425	114	383<br>631	5.1<br>451	13.18	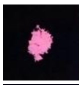
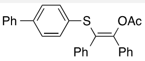 15	290	420	130	388	4.7	2.38	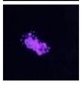
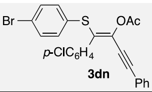 3dn	332	425	93	401	5.3	4.69	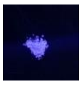

[a] λ_{abs} and λ_{em} refer to optimal absorption wavelength and photoluminescence (PL) peak, respectively. [b] α_{AIE} is defined as PL intensity in aggregation state/PL intensity in solution state. [c] Photo images of the solid under UV light (λ_{ex} = 365nm).

10.1 Investigation on the Emission Spectra

Using **3aa**, **3ca**, **3da**, **3sa**, **3dn** and **15** as representative compounds, their solid-state emission spectra are shown in Figure S6-S11. It can be seen that compounds **3ca**, **3da** and **3sa** has double emission peaks in the solid state.

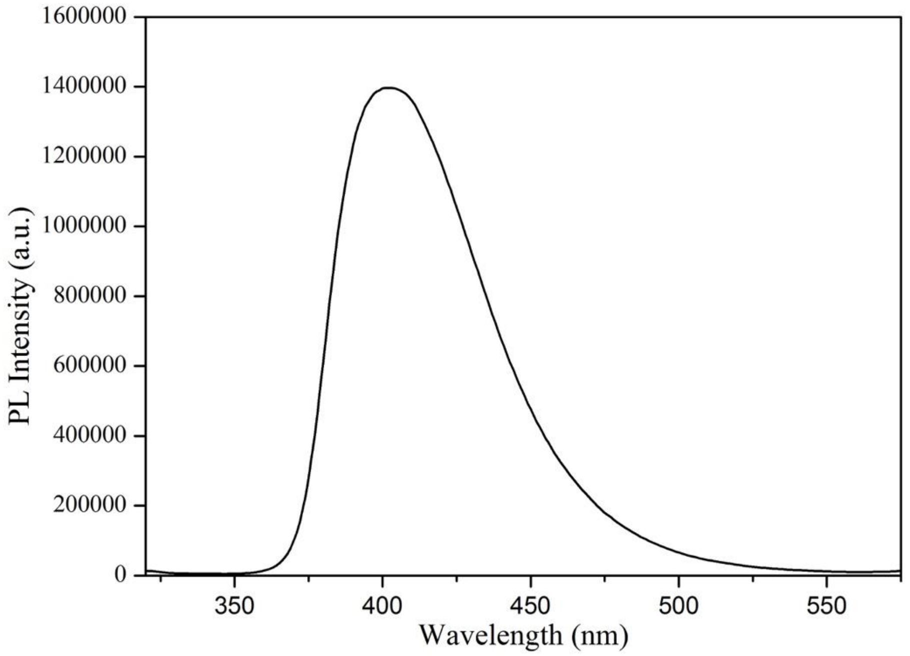

Supplementary Figure 9. Emission spectrum of compound **3aa** in solid state (λ_{ex} = 296 nm)

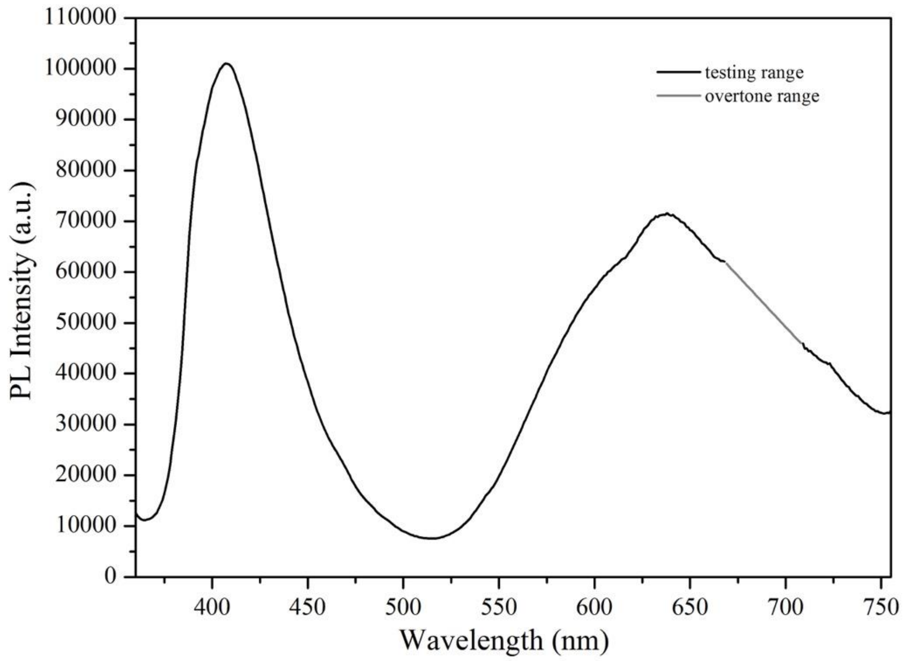

Supplementary Figure 10. Emission spectrum of compound **3ca** in solid state (λ_{ex} = 345 nm)

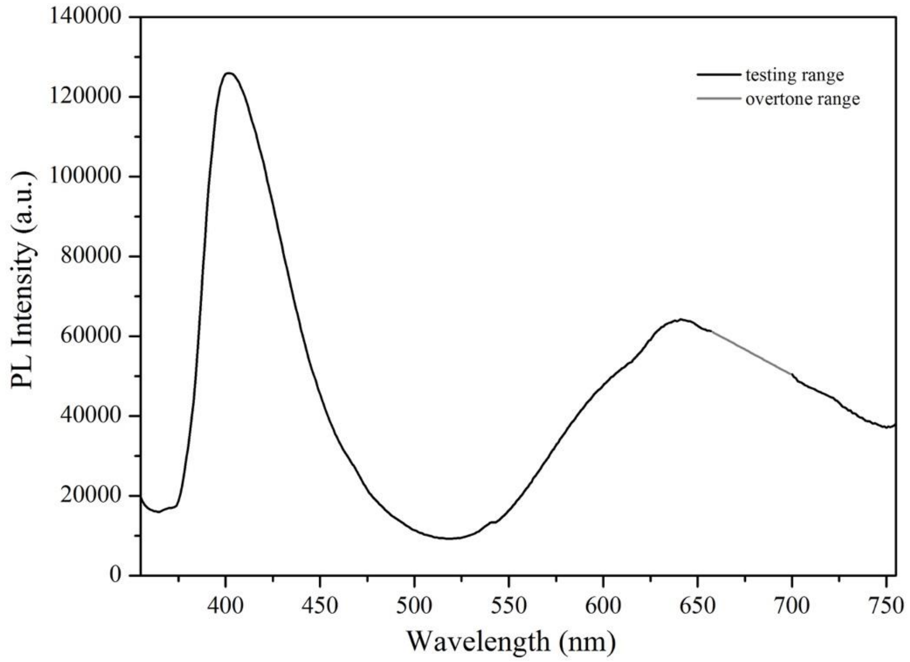

Supplementary Figure 11. Emission spectrum of compound **3da** in solid state (λ_{ex} = 340 nm)

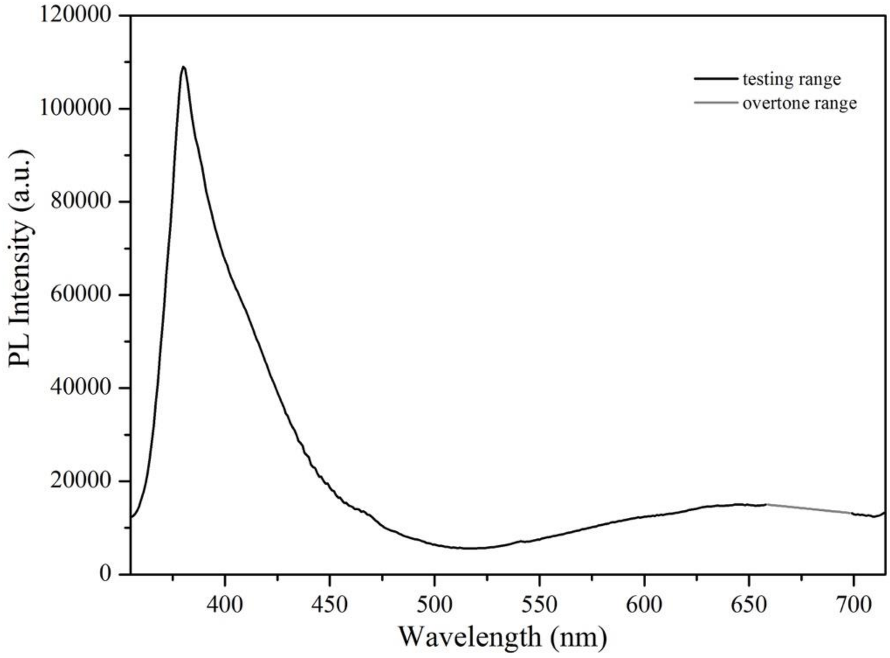

Supplementary Figure 12. Emission spectrum of compound **3sa** in solid state (λ_{ex} = 365 nm)

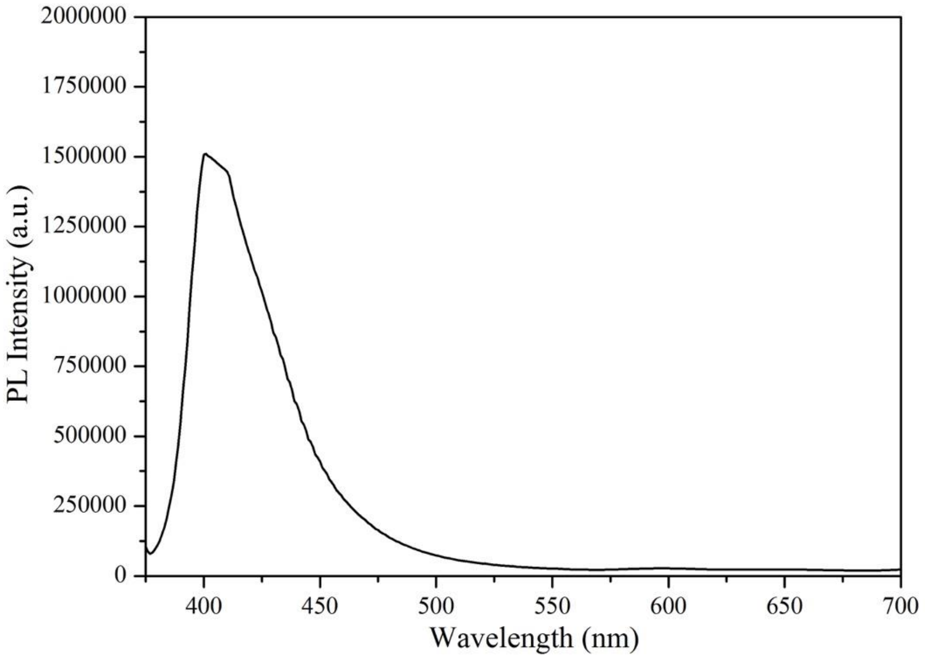

Supplementary Figure 13. Emission spectrum of compound **3dn** in solid state (λ_{ex} = 365 nm)

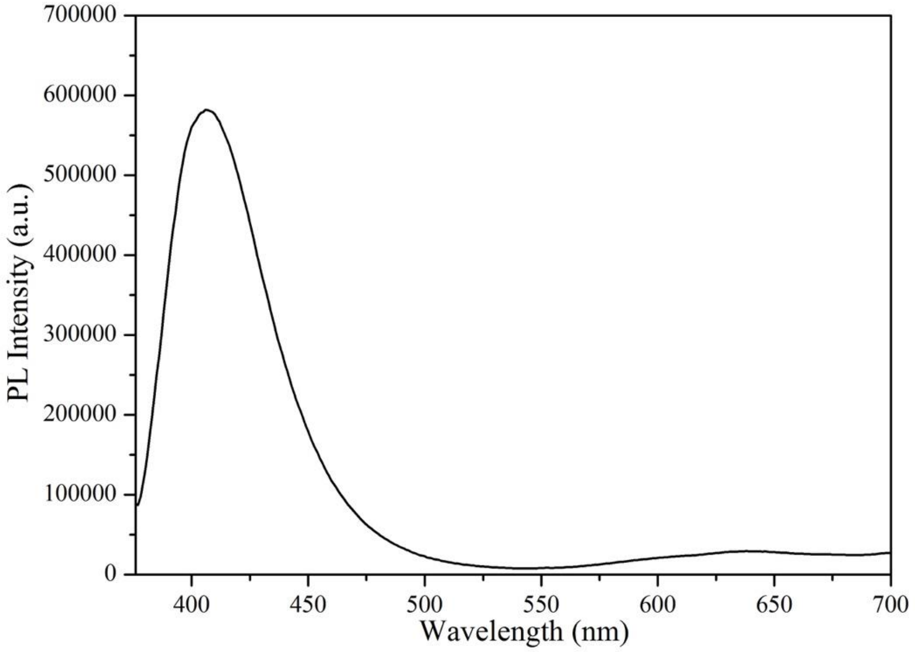

Supplementary Figure 14. Emission spectrum of compound **15** in solid state (λ_{ex} = 365 nm)

10.2 Investigation on the Fluorescence Lifetime

The study of solid emission spectroscopy shows that the presence of different substituents on the aromatic ring will affect the emission spectrum, especially the halogen substitution will have a special double emission phenomenon, and will also affect other fluorescence behaviors, such as fluorescence lifetime. It is worth noting that the fluorescence lifetime of compound **3ca**, **3da** and **3sa** with dual emission at different emission wavelengths is very different, and the fluorescence lifetime in the red-light region can reach 0.5ms.

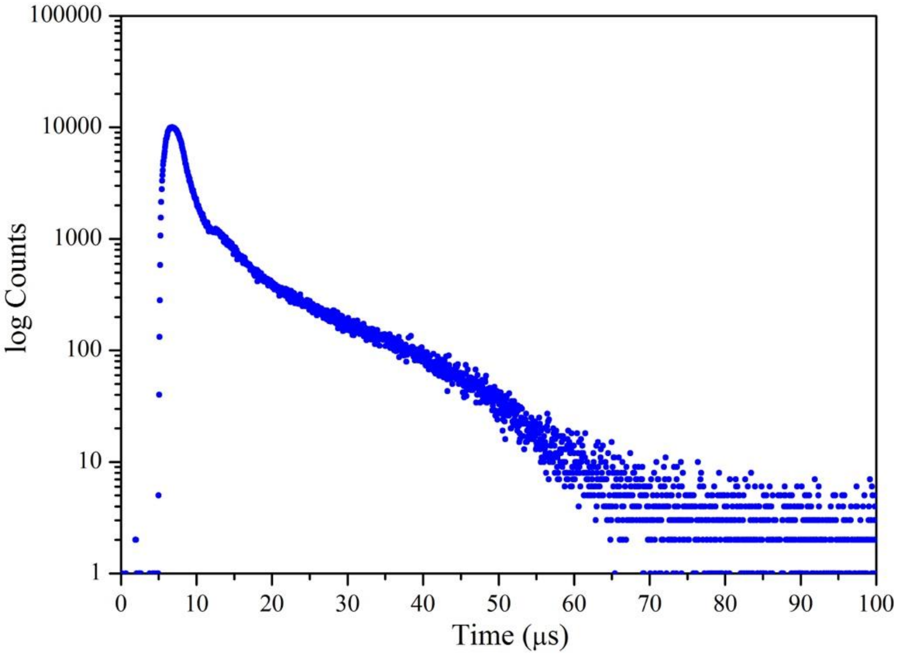

Supplementary Figure 15. Fluorescence decay curves of **3aa** in solid state (λ_{em} = 401 nm)

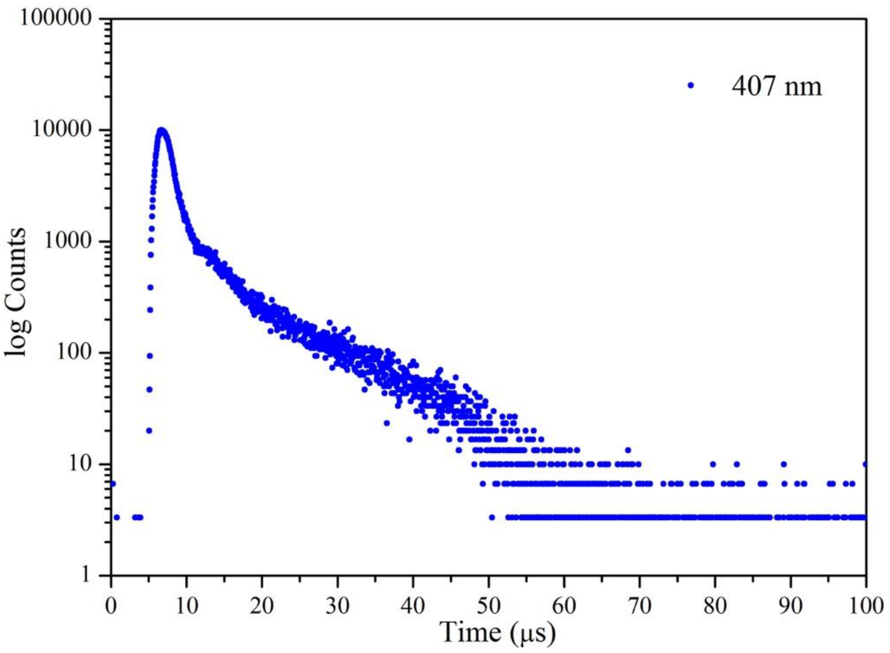

Supplementary Figure 16. Fluorescence decay curves of **3ca** in solid state (λ_{em} = 407 nm)

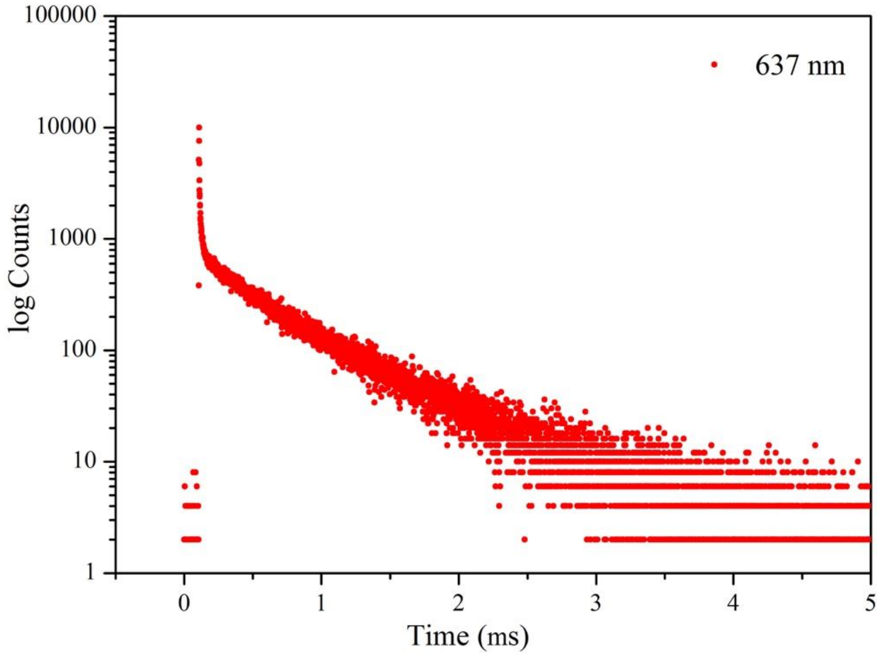

Supplementary Figure 17. Fluorescence decay curves of **3ca** in solid state (λ_{em} = 637 nm)

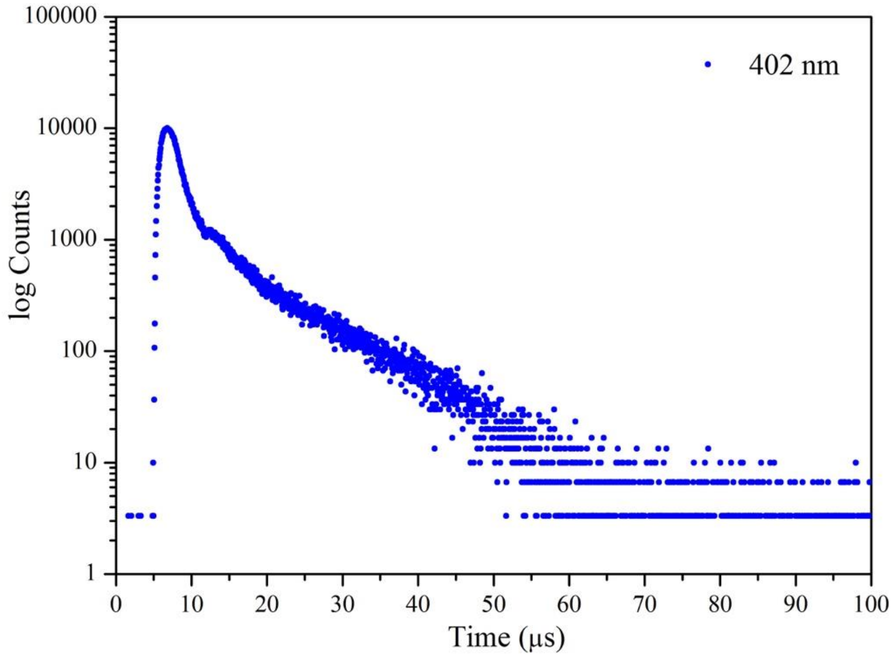

Supplementary Figure 18. Fluorescence decay curves of **3da** in solid state (λ_{em} = 402 nm)

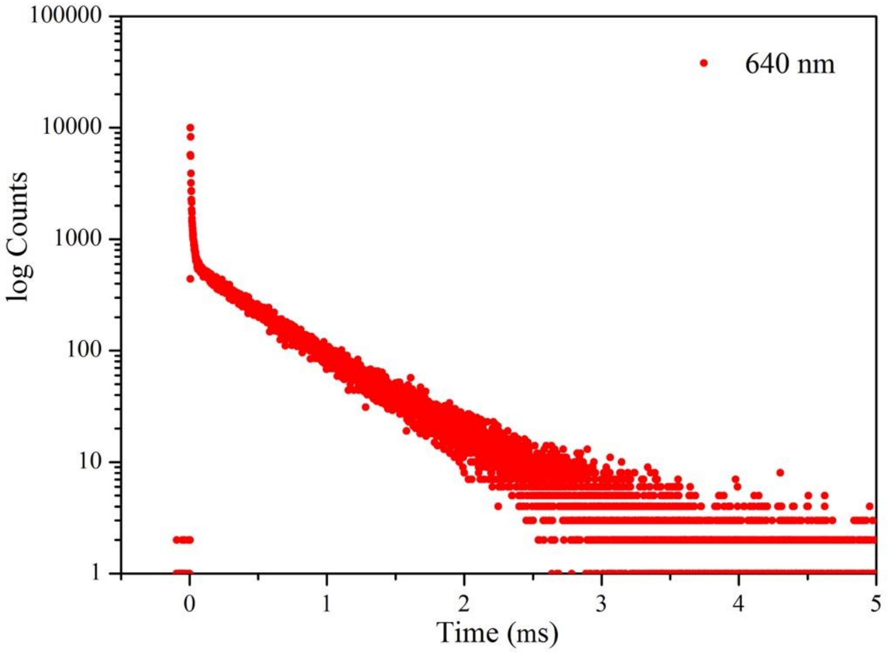

Supplementary Figure 19. Fluorescence decay curves of **3da** in solid state (λ_{em} = 640 nm)

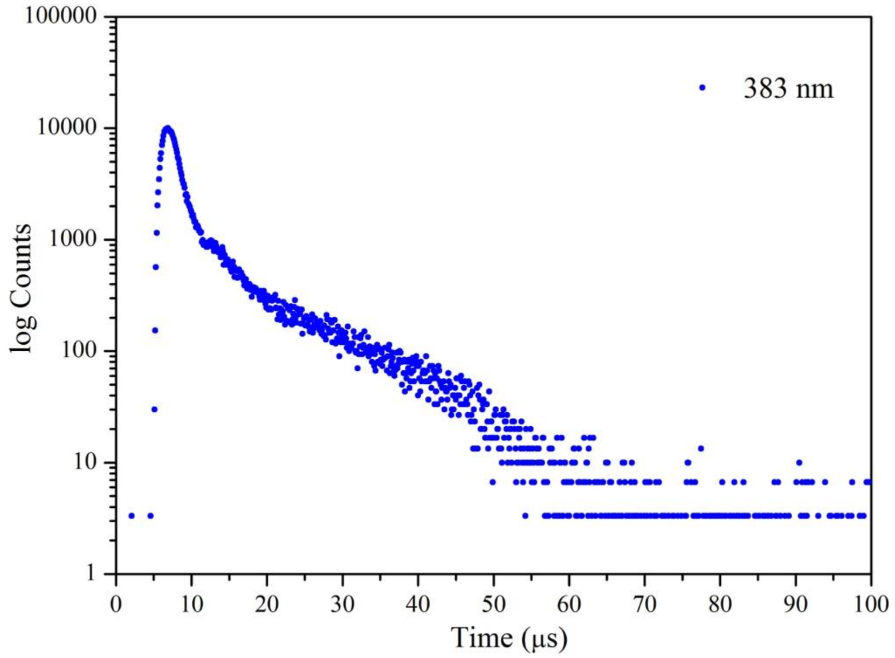

Supplementary Figure 20. Fluorescence decay curves of **3sa** in solid state (λ_{em} = 383 nm)

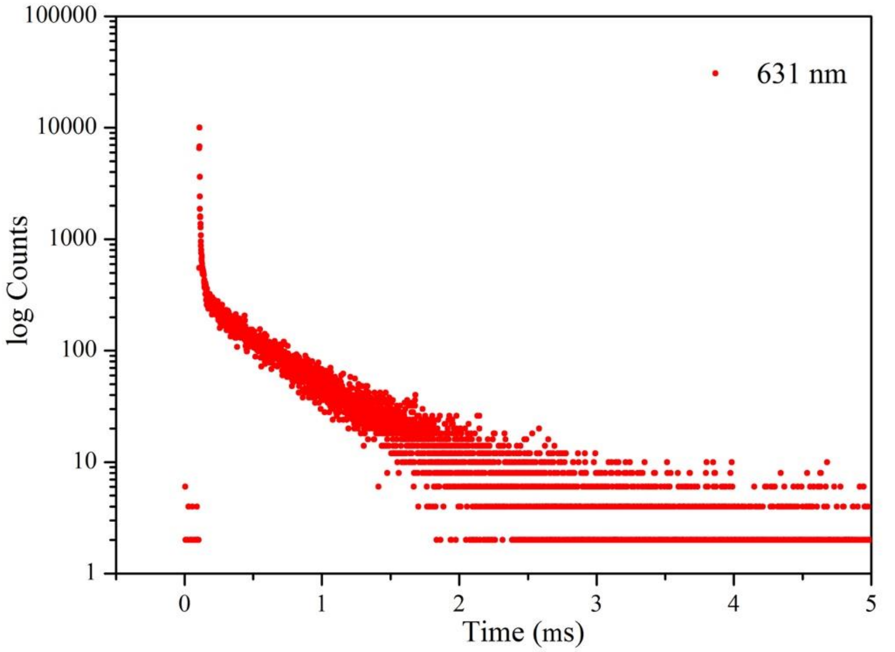

Supplementary Figure 21. Fluorescence decay curves of **3sa** in solid state (λ_{em} = 631 nm)

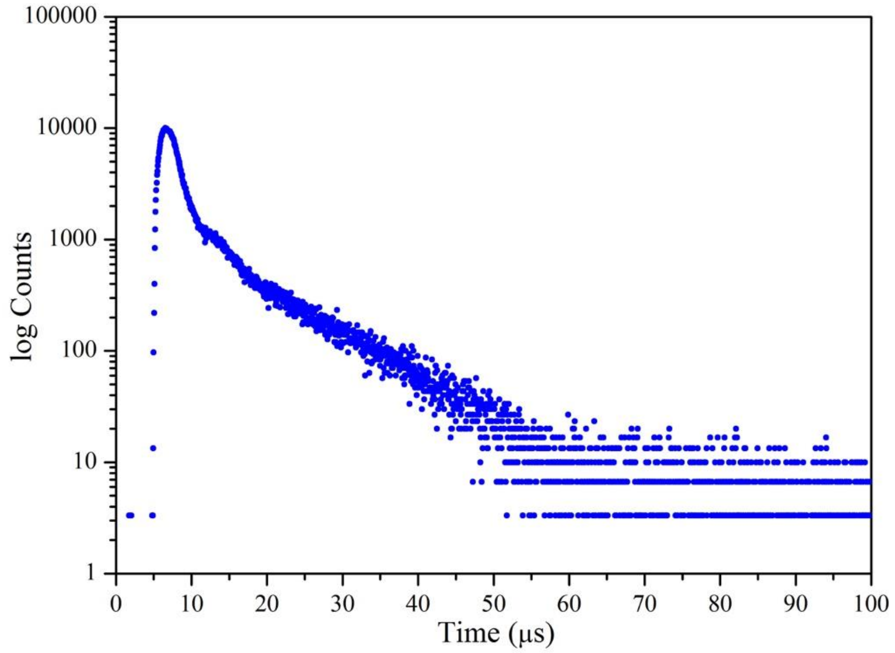

Supplementary Figure 22. Fluorescence decay curves of **3dn** in solid state (λ_{em} = 401 nm)

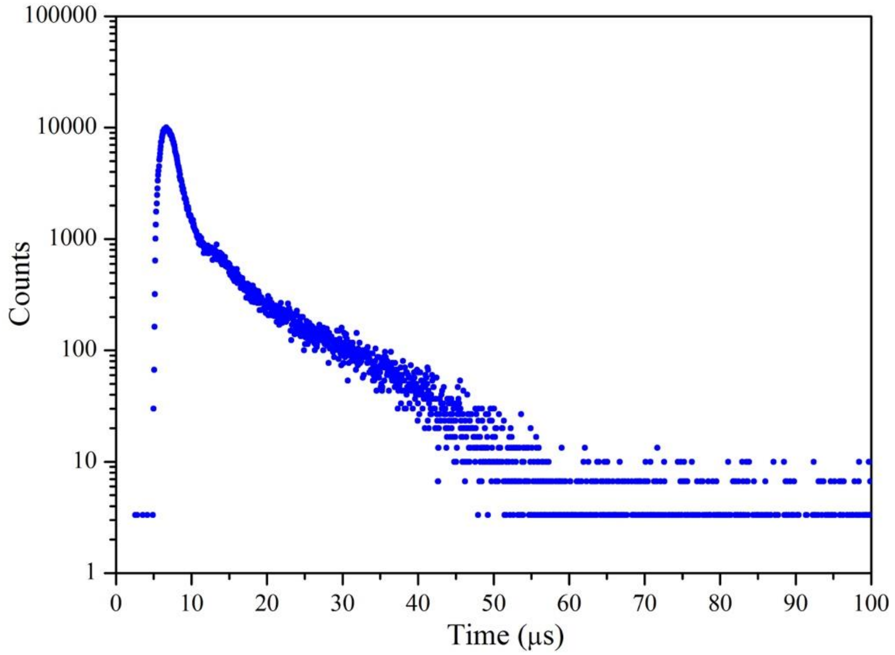

Supplementary Figure 23. Fluorescence decay curves of **15** in solid state (λ_{em} = 388 nm)

10.3 Fluorescence Behaviour of Compounds 3 in 1,4-dioxane/H_2O Mixture

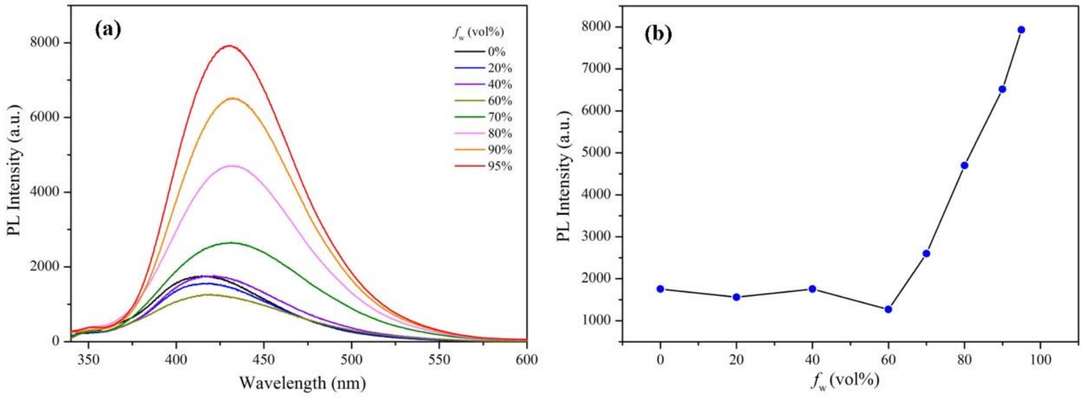

Supplementary Figure 24. (a) Emission spectra and (b) emission profile of compound **3aa** in 1,4-dioxane /H_2O mixture with increasing f_w to 95% (c = 150 μM, λ_{ex} = 313 nm).

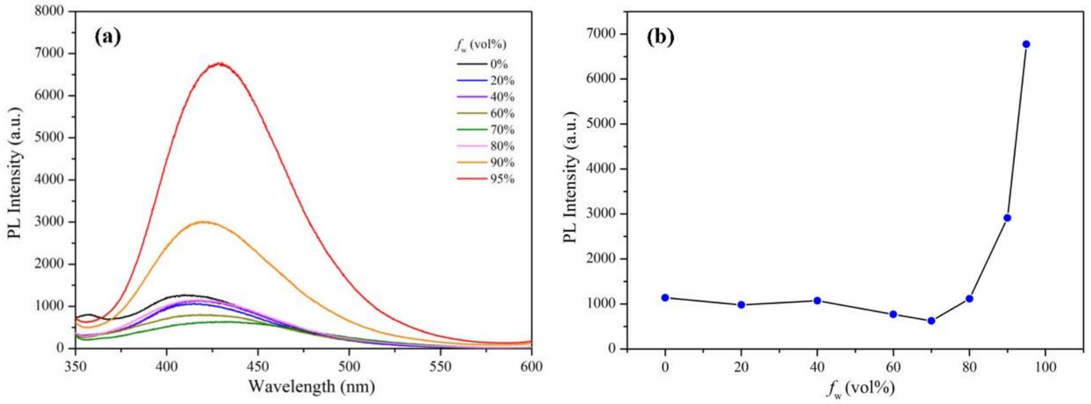

Supplementary Figure 25. (a) Emission spectra and (b) emission profile of compound **3ba** in 1,4-dioxane /H_2O mixture with increasing f_w to 95% (c = 150 μM, λ_{ex} = 309 nm).

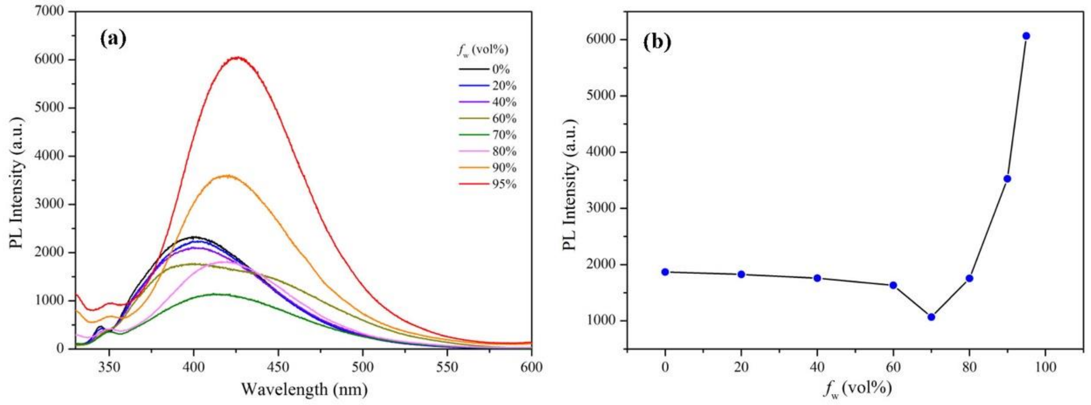

Supplementary Figure 26. (a) Emission spectra and (b) emission profile of compound **3ca** in 1,4-dioxane /H_2O mixture with increasing f_w to 95% (c = 150 μM, λ_{ex} = 312 nm).

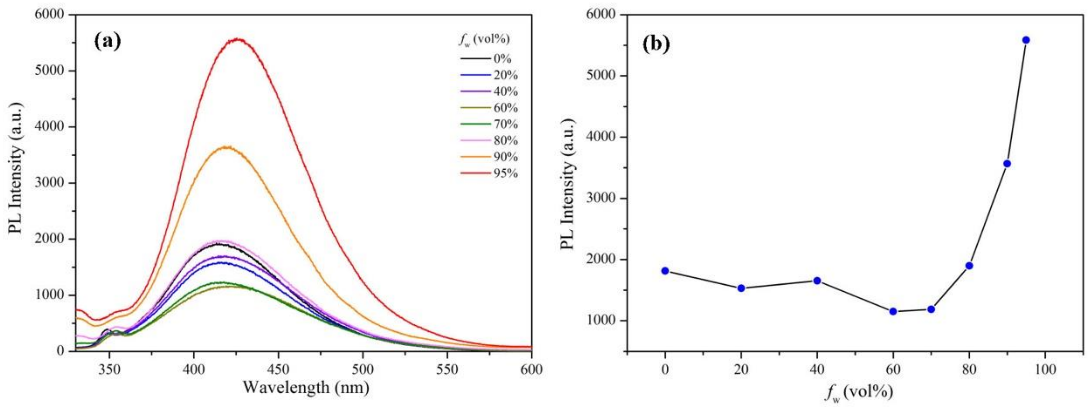

Supplementary Figure 27. (a) Emission spectra and (b) emission profile of compound **3da** in 1,4-dioxane /H_2O mixture with increasing f_w to 95% (c = 150 μM, λ_{ex} = 315 nm).

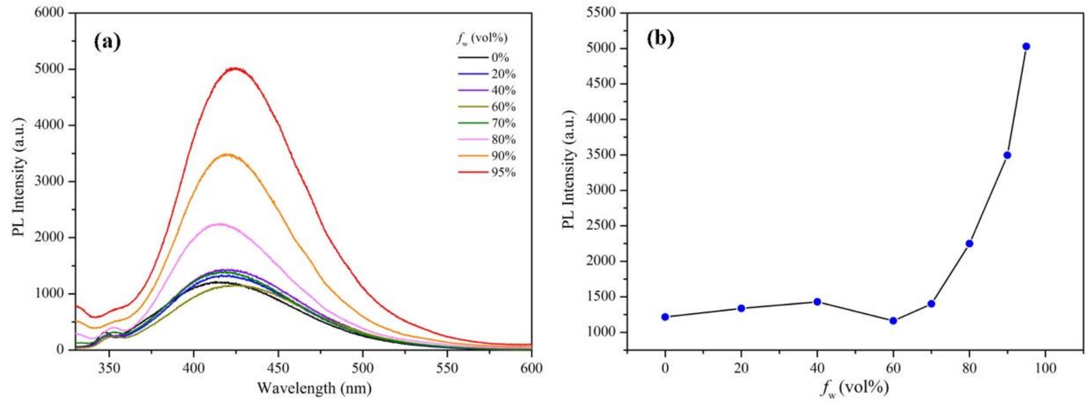

Supplementary Figure 28. (a) Emission spectra and (b) emission profile of compound **3ea** in 1,4-dioxane /H_2O mixture with increasing f_w to 95% (c = 150 μM, λ_{ex} = 314 nm).

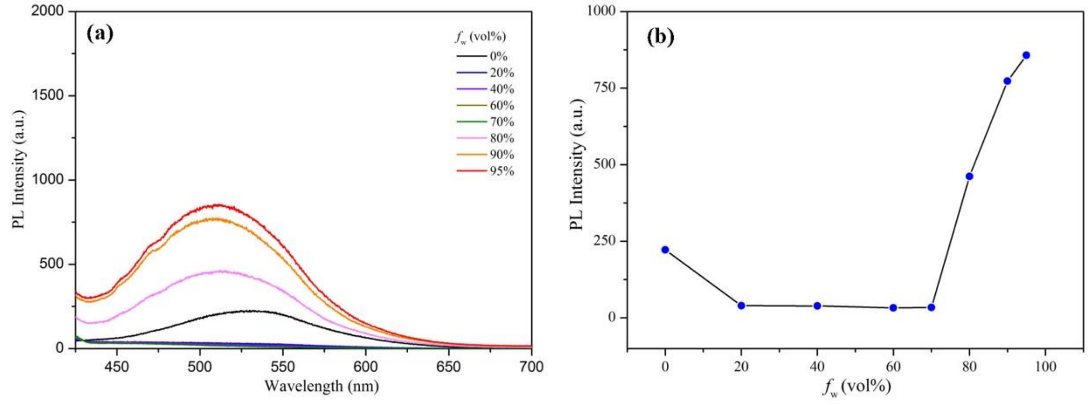

Supplementary Figure 29. (a) Emission spectra and (b) emission profile of compound **3fa** in 1,4-dioxane /H_2O mixture with increasing f_w to 95% (c = 150 μM, λ_{ex} = 370 nm).

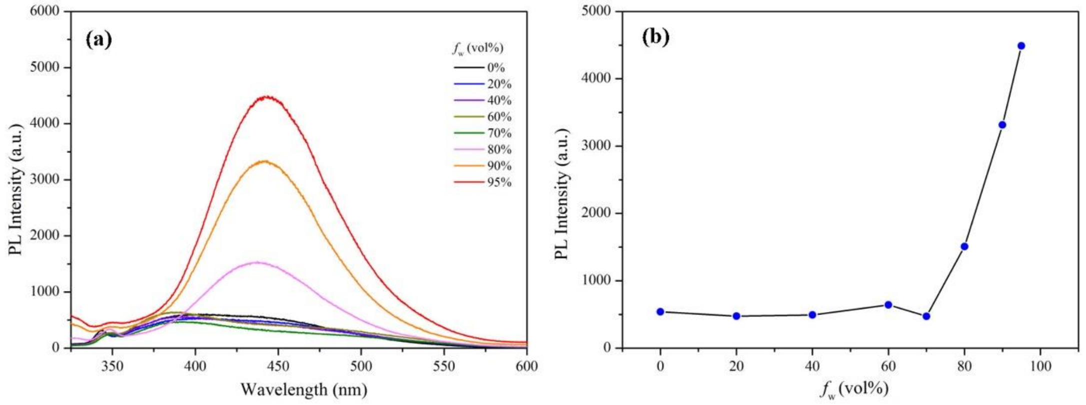

Supplementary Figure 30. (a) Emission spectra and (b) emission profile of compound **3ha** in 1,4-dioxane /H_2O mixture with increasing f_w to 95% (c = 150 μM, λ_{ex} = 311 nm).

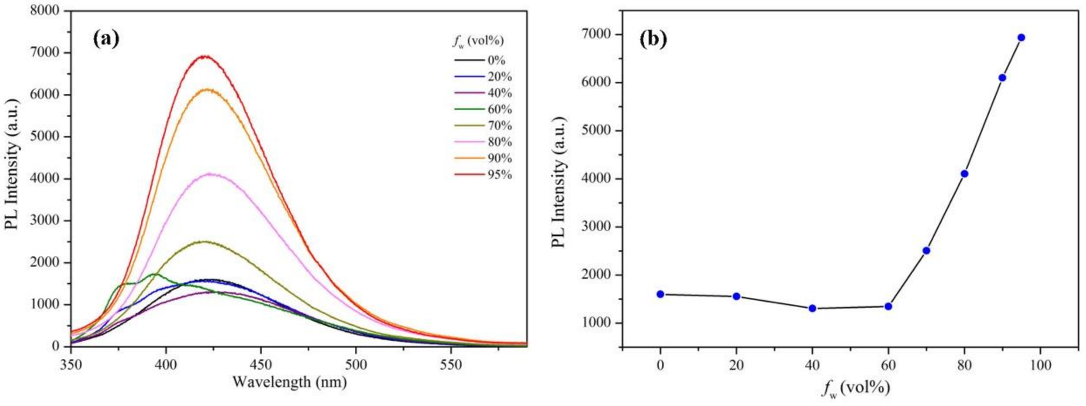

Supplementary Figure 31. (a) Emission spectra and (b) emission profile of compound **3la** in 1,4-dioxane /H_2O mixture with increasing f_w to 95% (c = 150 μM, λ_{ex} = 307 nm).

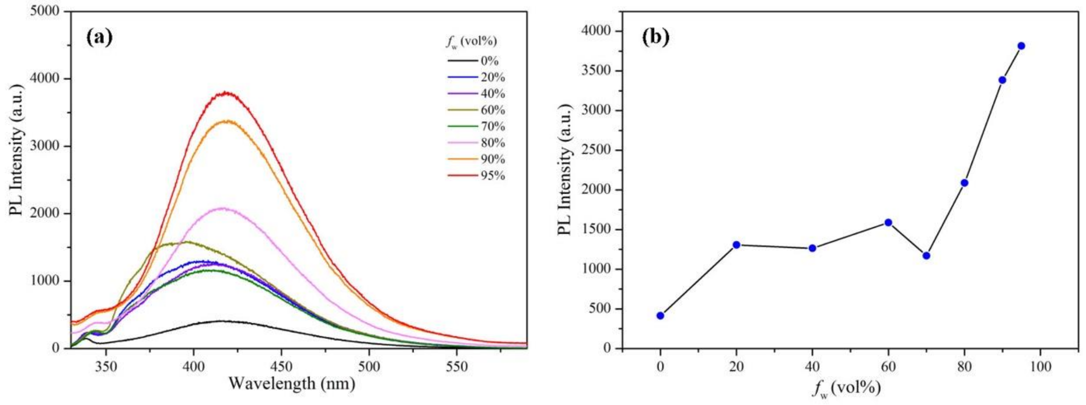

Supplementary Figure 32. (a) Emission spectra and (b) emission profile of compound **3ma** in 1,4-dioxane /H_2O mixture with increasing f_w to 95% (c = 150 μM, λ_{ex} = 307 nm).

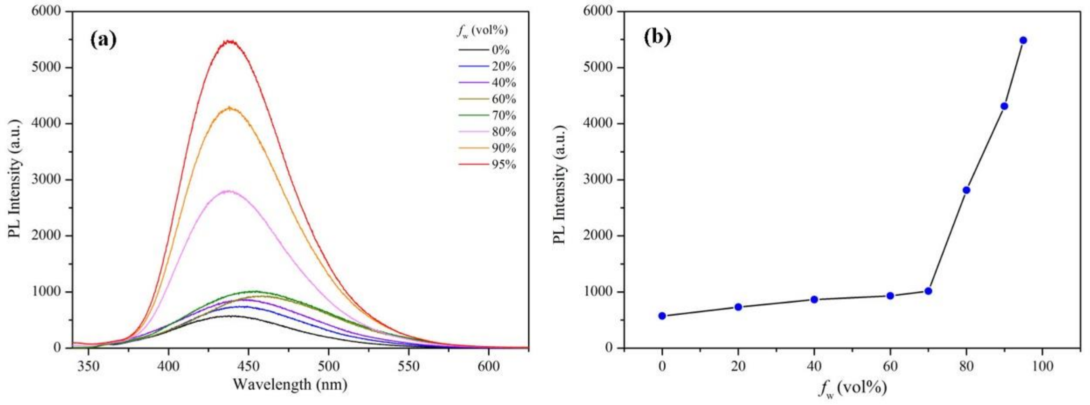

Supplementary Figure 33. (a) Emission spectra and (b) emission profile of compound **3of** in 1,4-dioxane /H_2O mixture with increasing f_w to 95% (c = 150 μM, λ_{ex} = 325 nm).

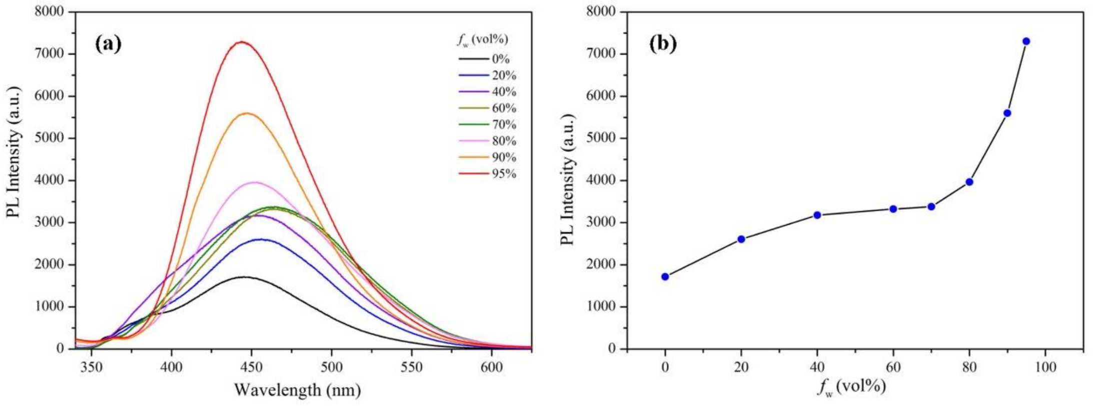

Supplementary Figure 34. (a) Emission spectra and (b) emission profile of compound **3rf** in 1,4-dioxane /H_2O mixture with increasing f_w to 95% (c = 150 μM, λ_{ex} = 323 nm).

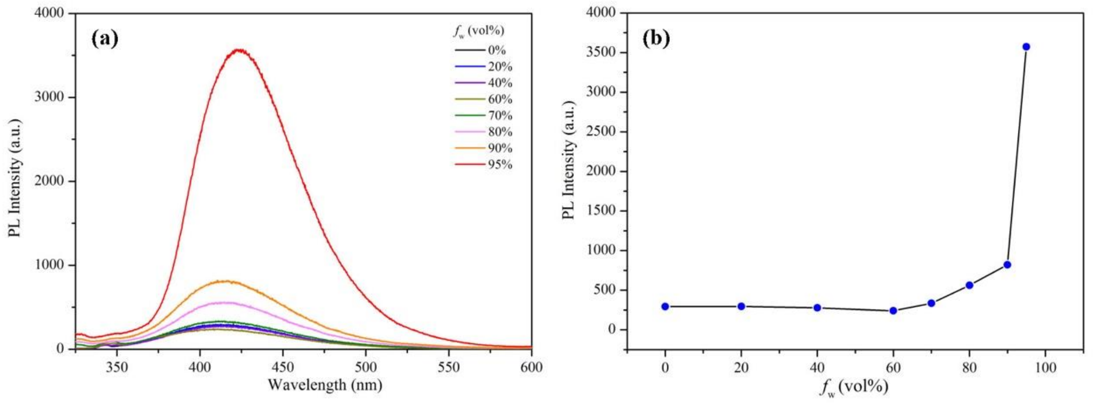

Supplementary Figure 35. (a) Emission spectra and (b) emission profile of compound **3sa** in 1,4-dioxane /H_2O mixture with increasing f_w to 95% (c = 150 μM, λ_{ex} = 310 nm).

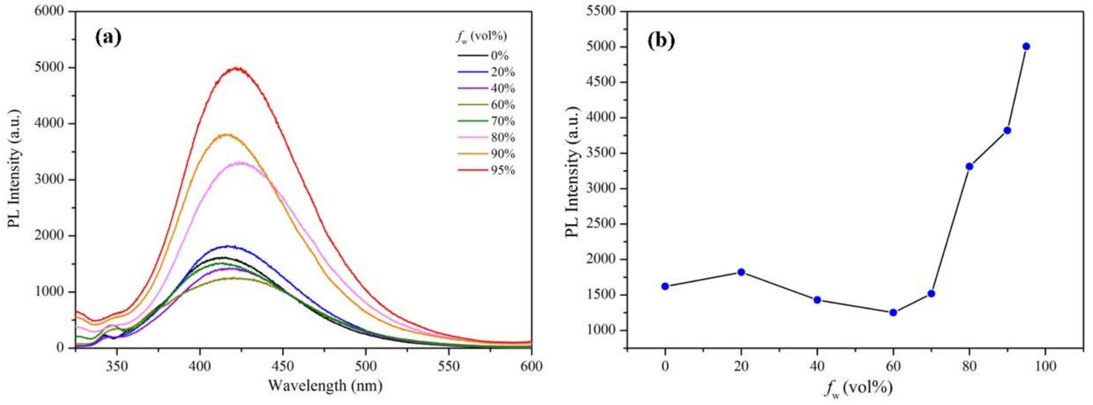

Supplementary Figure 36. (a) Emission spectra and (b) emission profile of compound **3ta** in 1,4-dioxane /H_2O mixture with increasing f_w to 95% (c = 150 μM, λ_{ex} = 310 nm).

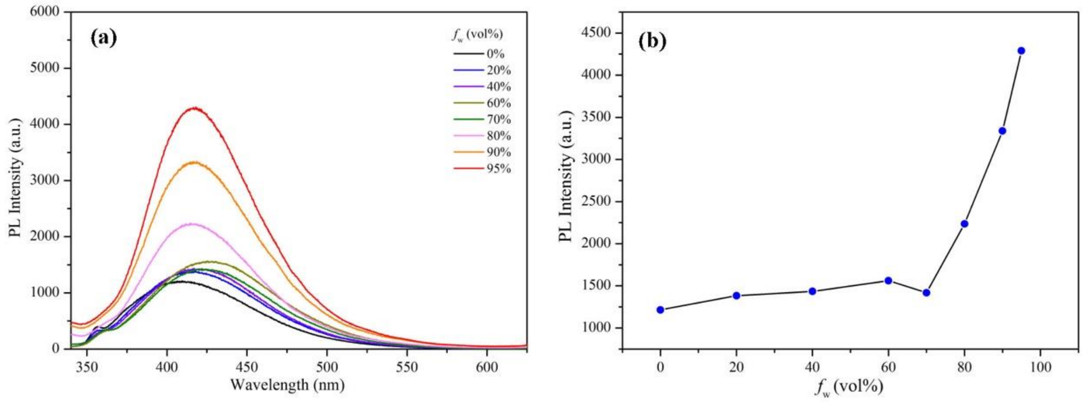

Supplementary Figure 37. (a) Emission spectra and (b) emission profile of compound **3wa** in 1,4-dioxane /H_2O mixture with increasing f_w to 95% (c = 150 μM, λ_{ex} = 321 nm).

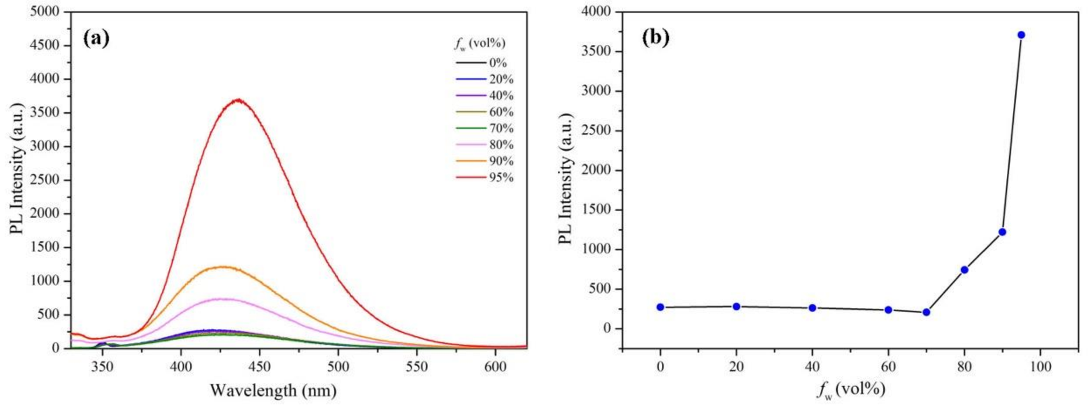

Supplementary Figure 38. (a) Emission spectra and (b) emission profile of compound **3ab** in 1,4-dioxane /H_2O mixture with increasing f_w to 95% (c = 150 μM, λ_{ex} = 317 nm).

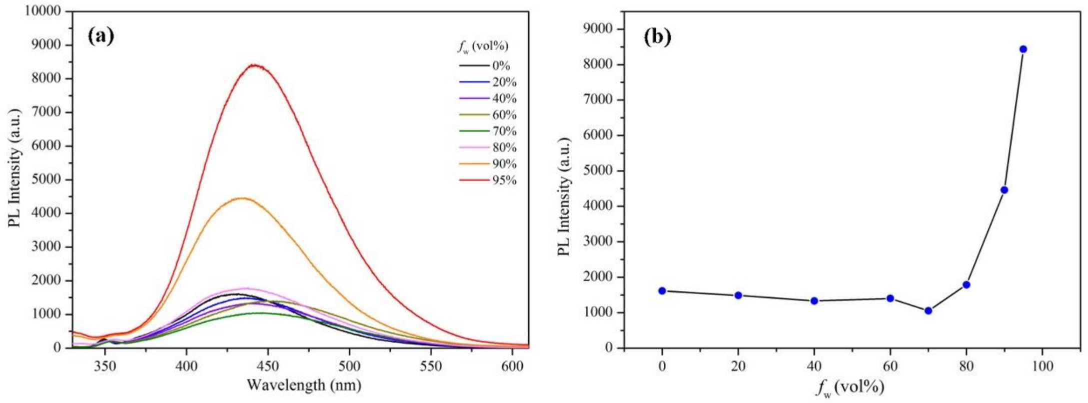

Supplementary Figure 39. (a) Emission spectra and (b) emission profile of compound **3ae** in 1,4-dioxane /H_2O mixture with increasing f_w to 95% (c = 150 μM, λ_{ex} = 316 nm).

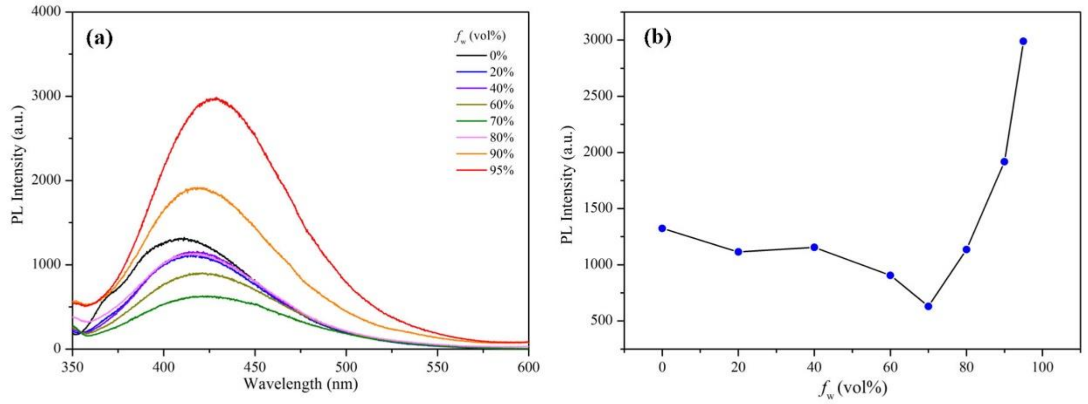

Supplementary Figure 40. (a) Emission spectra and (b) emission profile of compound **3ah** in 1,4-dioxane /H_2O mixture with increasing f_w to 95% (c = 150 μM, λ_{ex} = 312 nm).

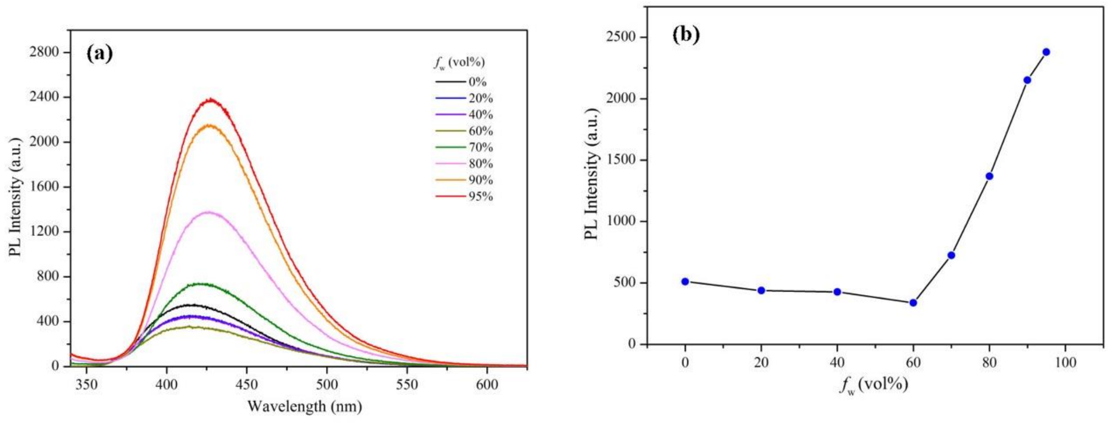

Supplementary Figure 41. (a) Emission spectra and (b) emission profile of compound **3dn** in 1,4-dioxane /H_2O mixture with increasing f_w to 95% (c = 150 μM, λ_{ex} = 330 nm).

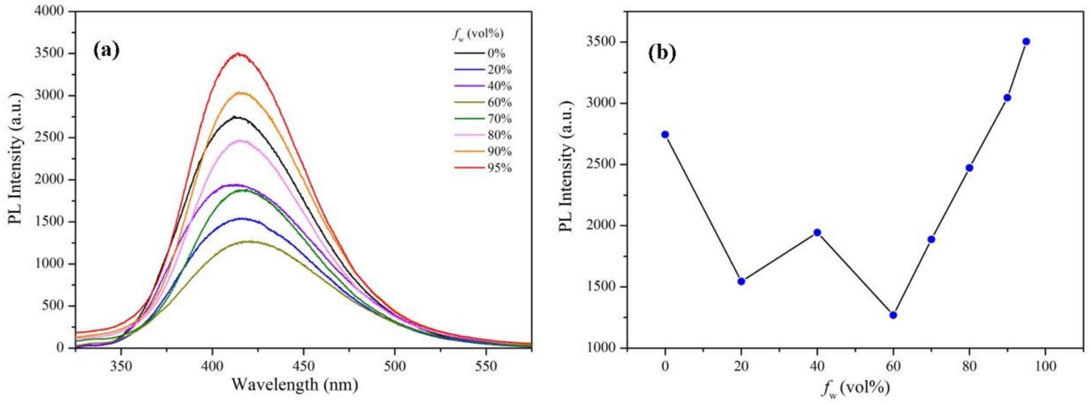

Supplementary Figure 42. (a) Emission spectra and (b) emission profile of compound **14** in 1,4-dioxane /H_2O mixture with increasing f_w to 95% (c = 150 μM, λ_{ex} = 300 nm).

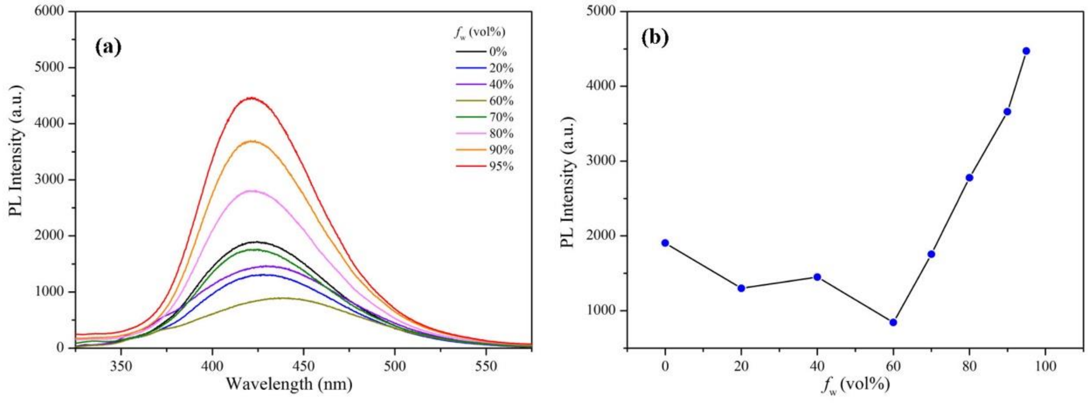

Supplementary Figure 43. (a) Emission spectra and (b) emission profile of compound **15** in 1,4-dioxane /H_2O mixture with increasing f_w to 95% (c = 150 μM, λ_{ex} = 300 nm).

11. NMR Spectra

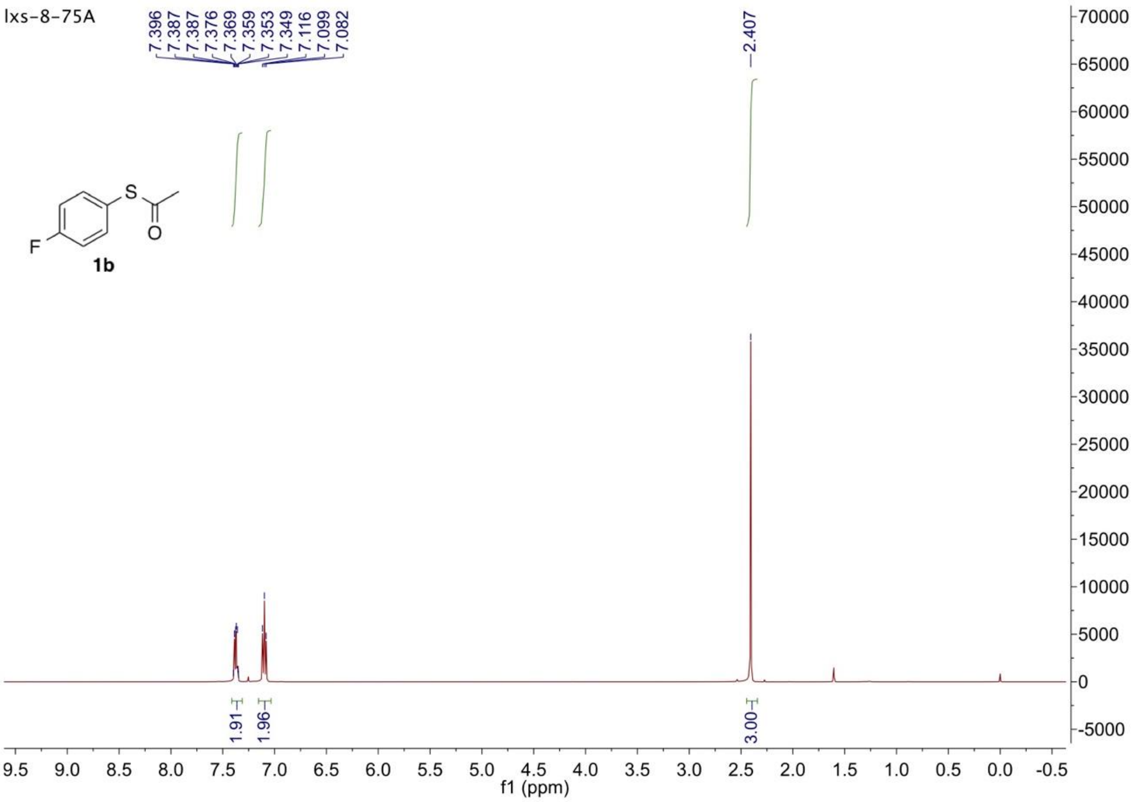

Supplementary Figure 44. ^{1}H NMR (500 MHz, $CDCl_3$) of compound **1b**

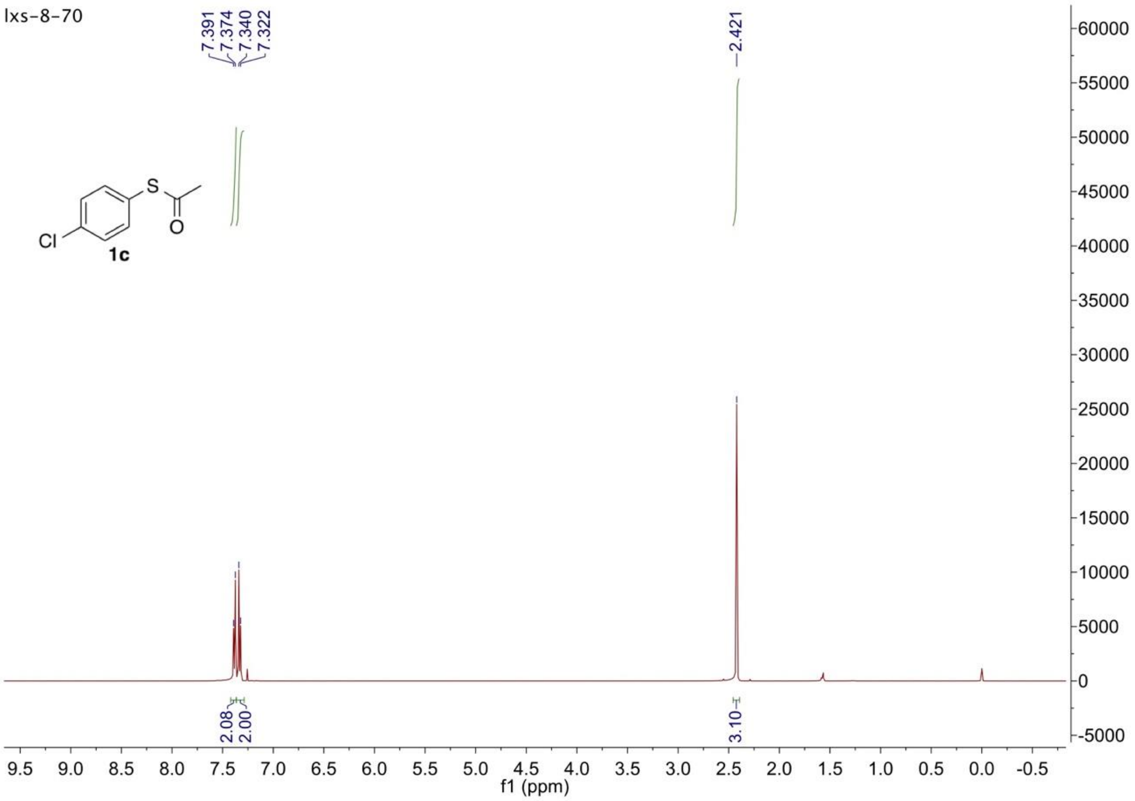

Supplementary Figure 45. ^{1}H NMR (500 MHz, $CDCl_3$) of compound **1c**

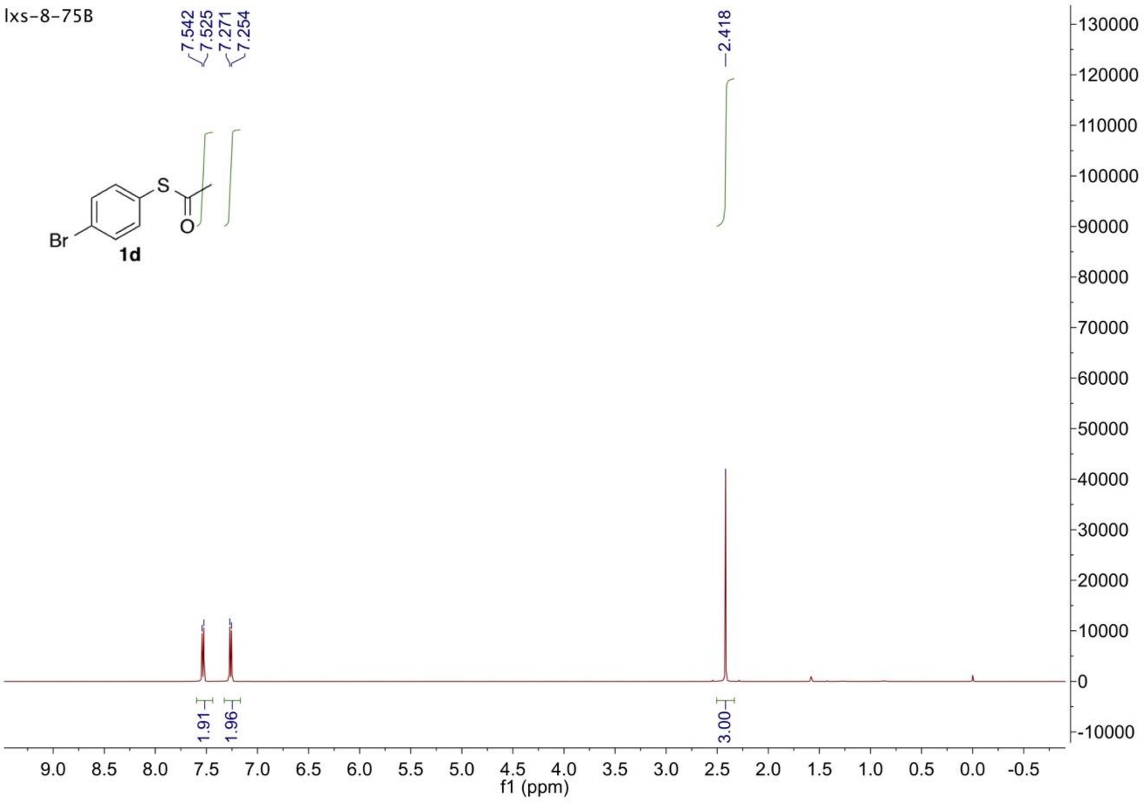

Supplementary Figure 46. ^{1}H NMR (500 MHz, $CDCl_3$) of compound **1d**

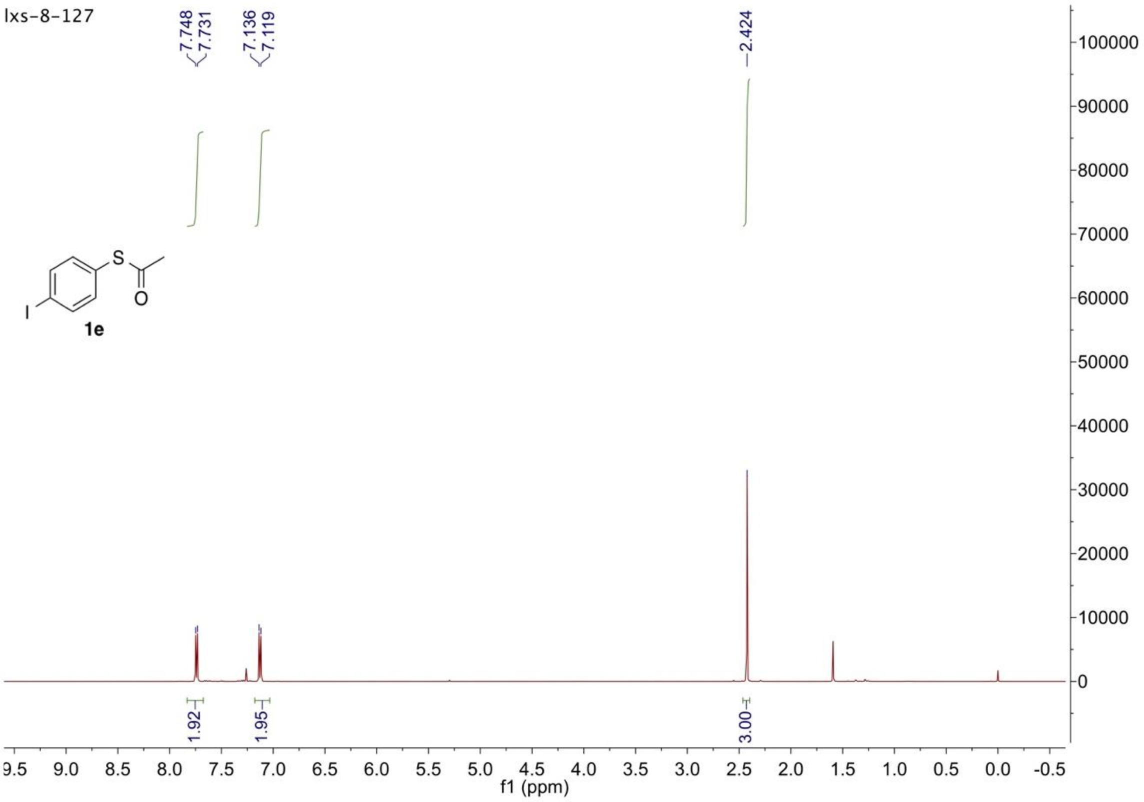

Supplementary Figure 47. ^{1}H NMR (500 MHz, $CDCl_3$) of compound **1e**

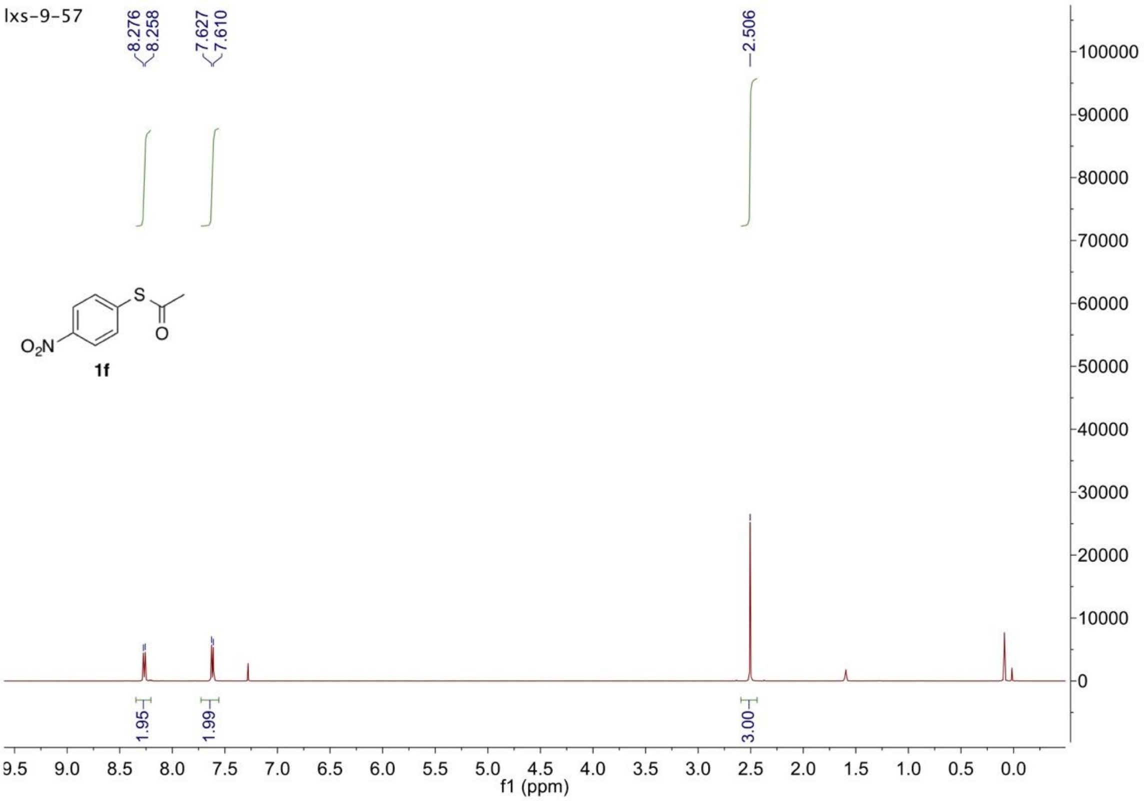

Supplementary Figure 48. ^{1}H NMR (500 MHz, $CDCl_3$) of compound **1f**

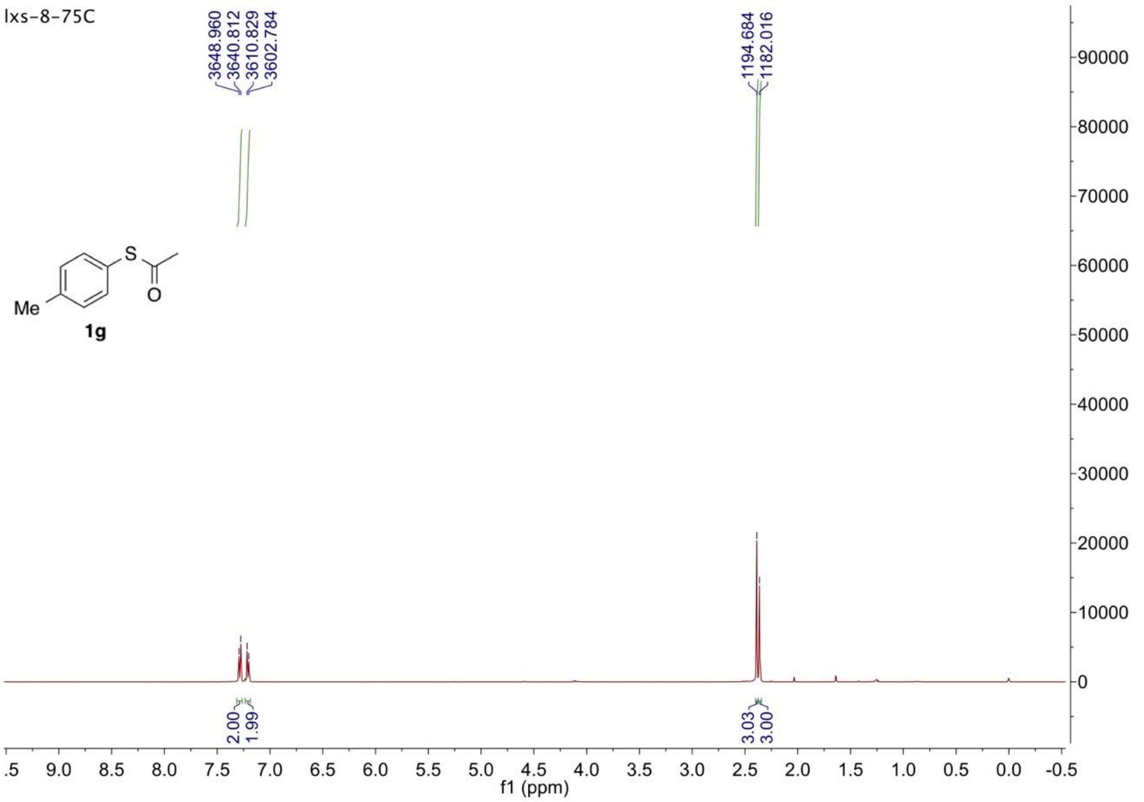

Supplementary Figure 49. ^{1}H NMR (500 MHz, $CDCl_3$) of compound **1g**

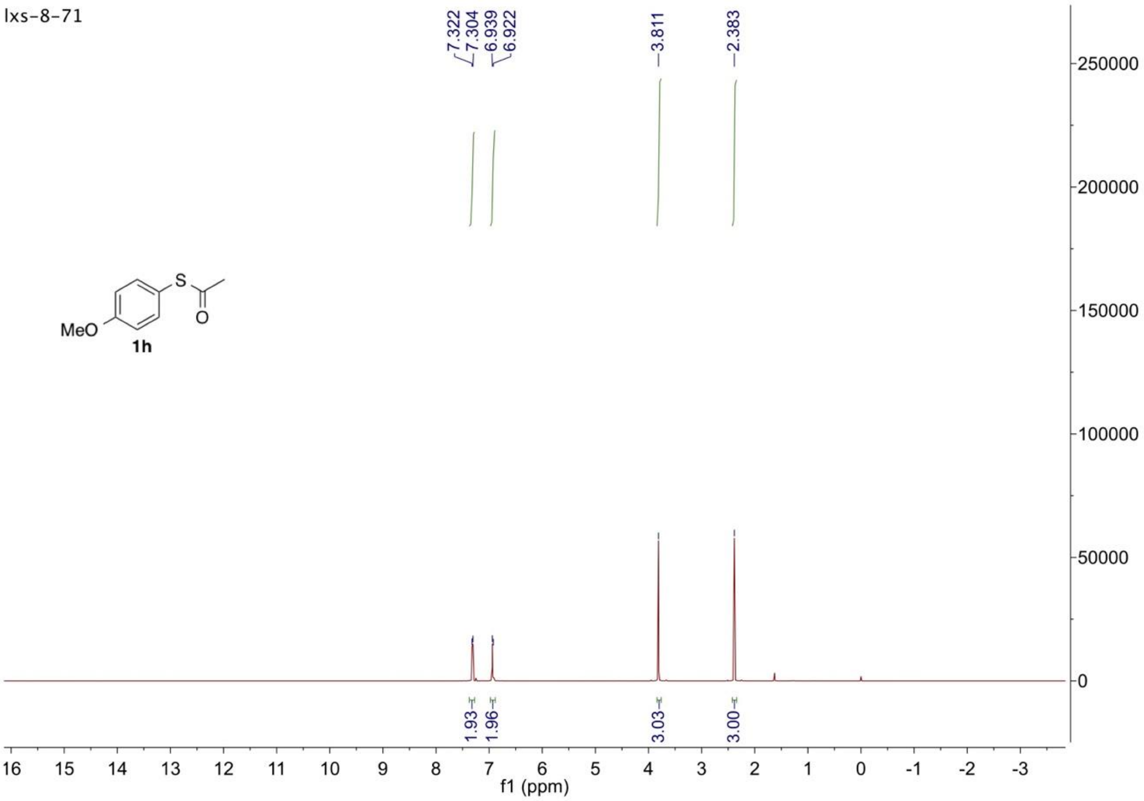

Supplementary Figure 50. ^{1}H NMR (500 MHz, $CDCl_3$) of compound **1h**

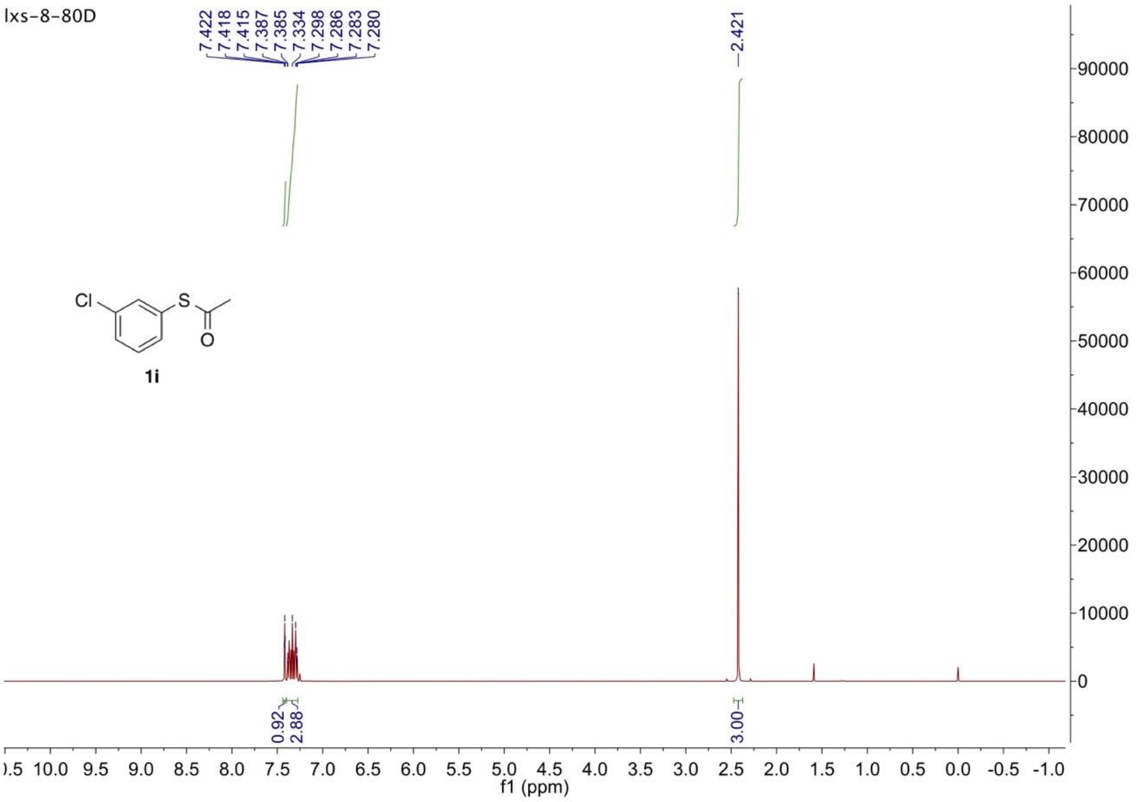

Supplementary Figure 51. ^{1}H NMR (500 MHz, $CDCl_3$) of compound **1i**

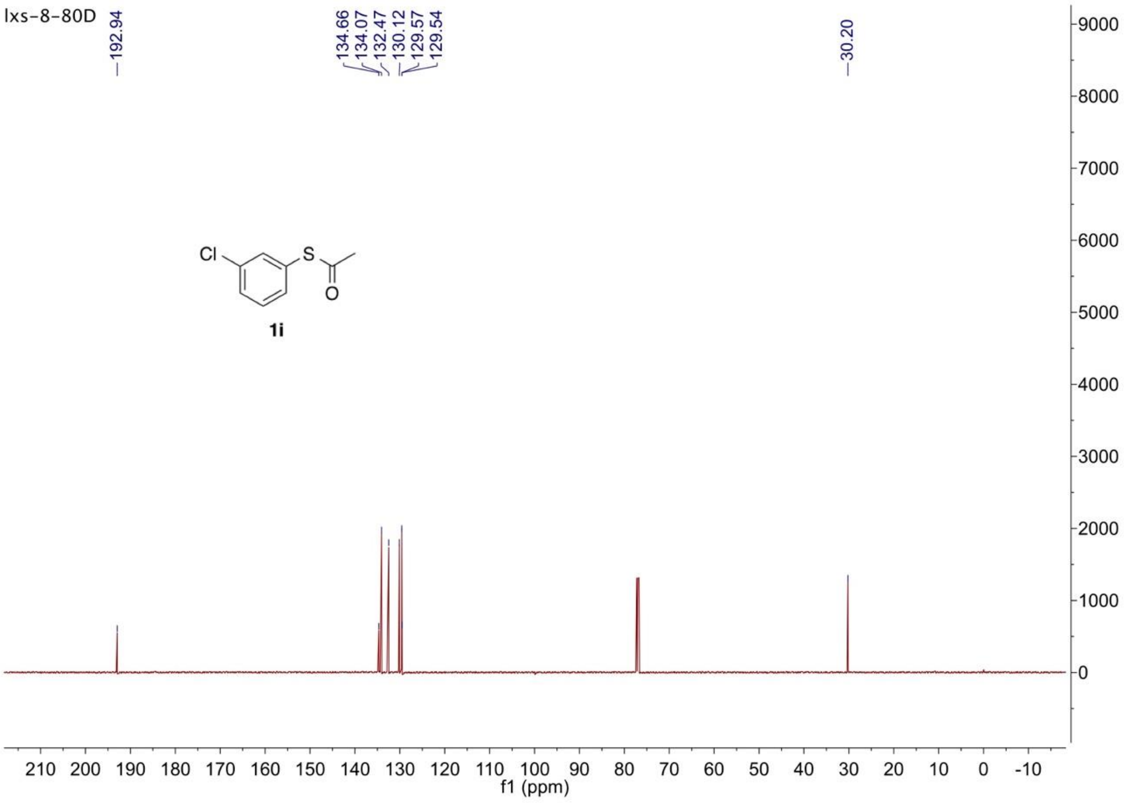

Supplementary Figure 52. ^{13}C NMR (125 MHz, $CDCl_3$) of compound **1i**

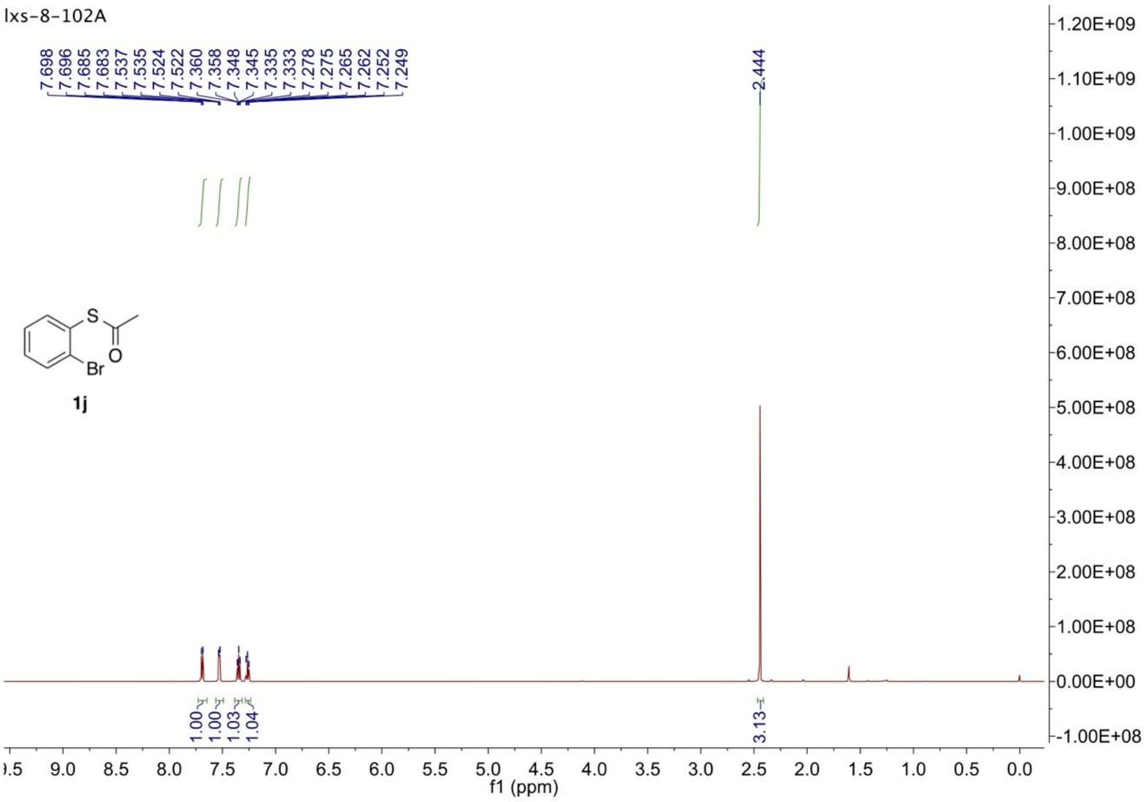

Supplementary Figure 53. ^{1}H NMR (600 MHz, $CDCl_3$) of compound **1j**

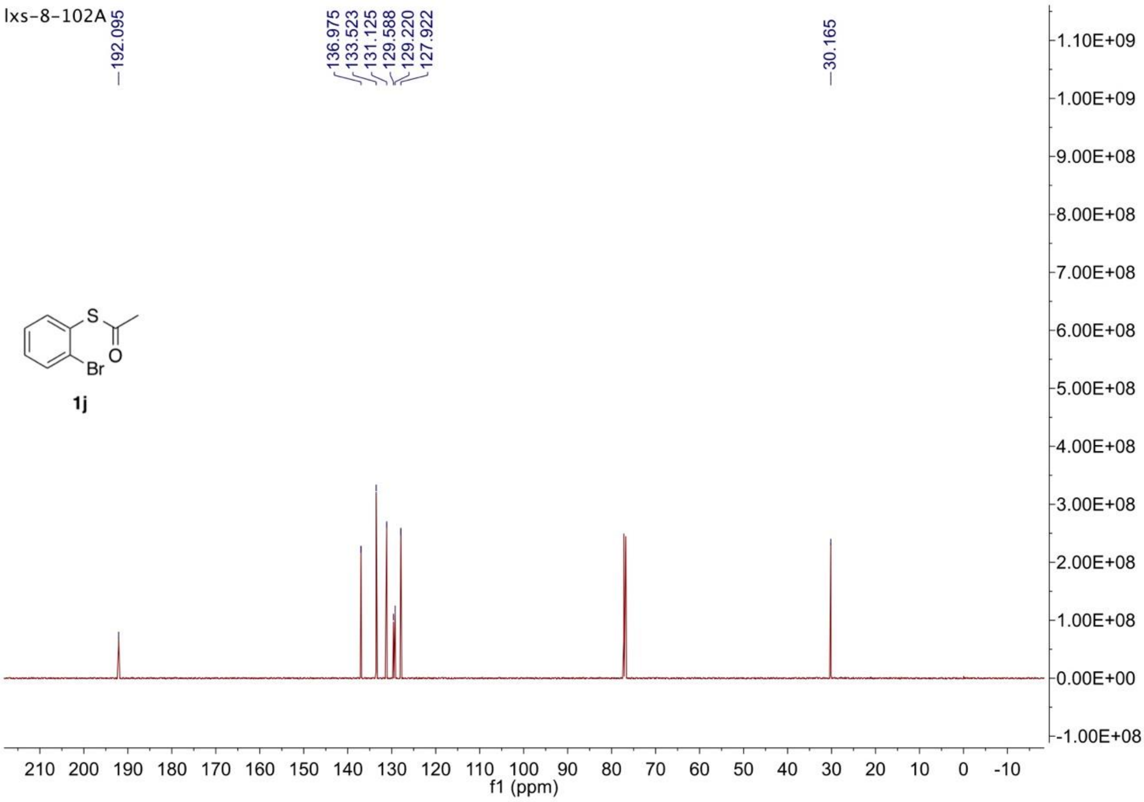

Supplementary Figure 54. ^{13}C NMR (150 MHz, $CDCl_3$) of compound **1j**

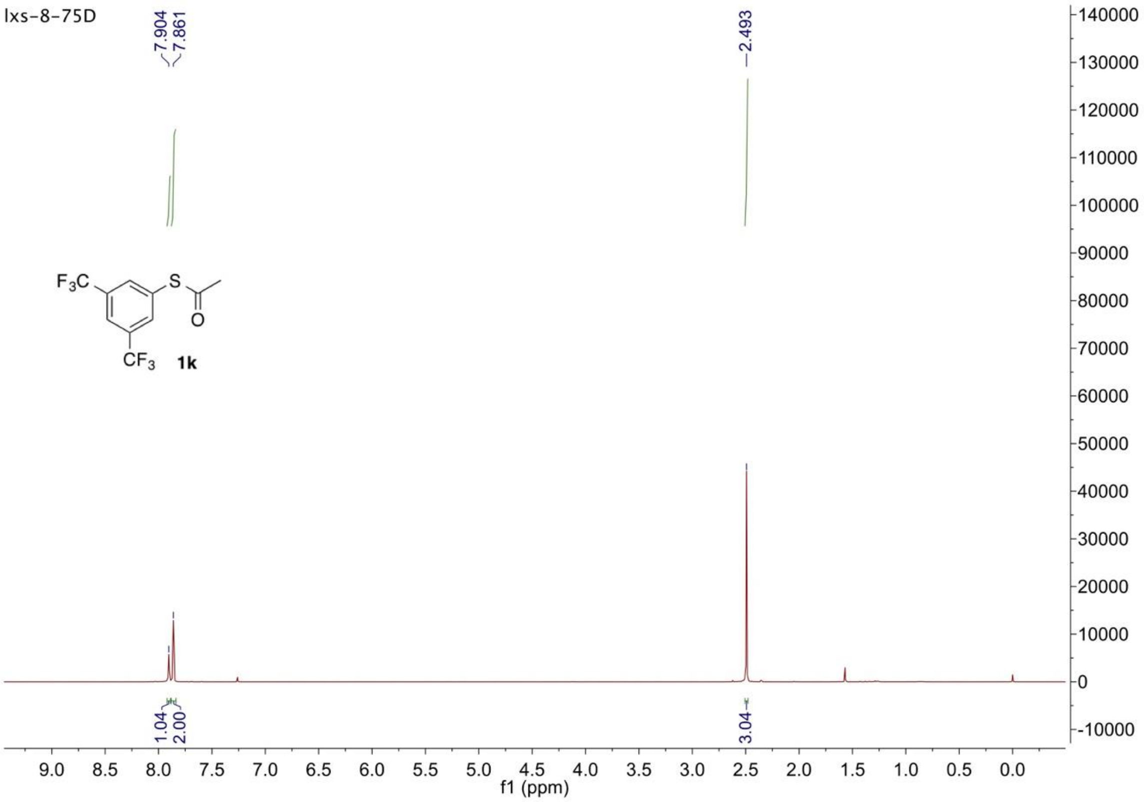

Supplementary Figure 55. ^{1}H NMR (500 MHz, $CDCl_3$) of compound **1k**

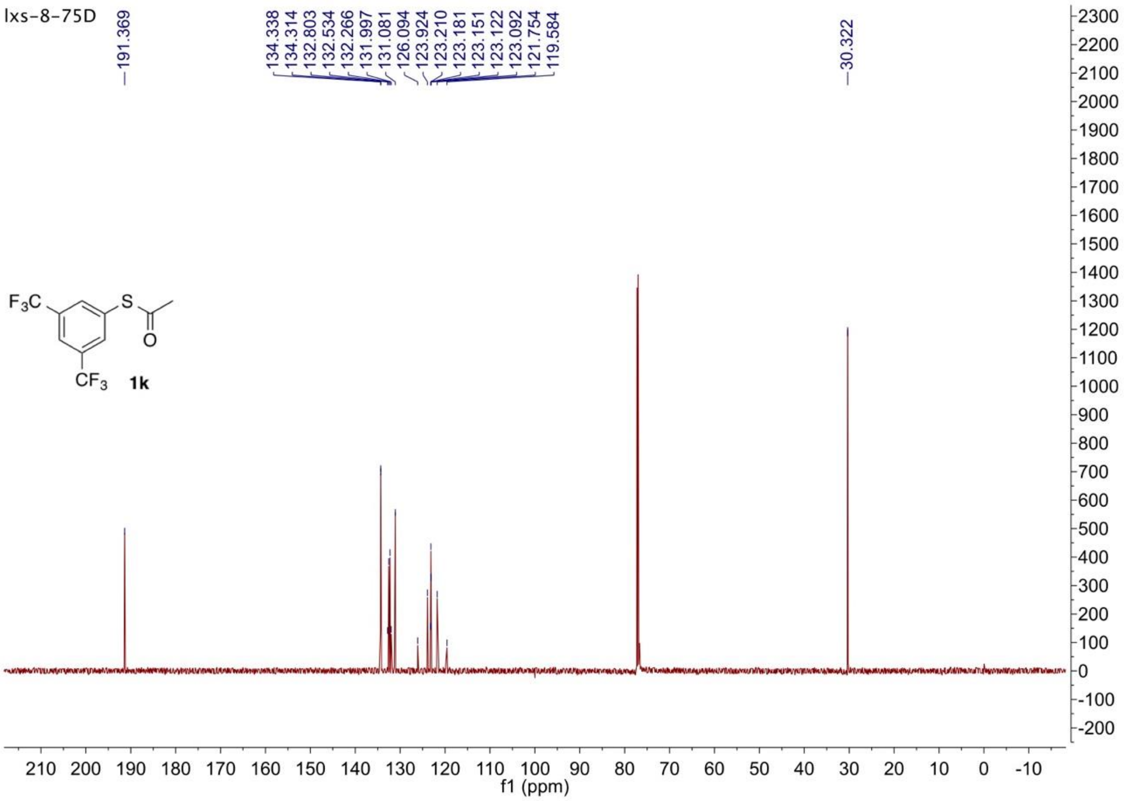

Supplementary Figure 56. ^{13}C NMR (150 MHz, $CDCl_3$) of compound **1k**

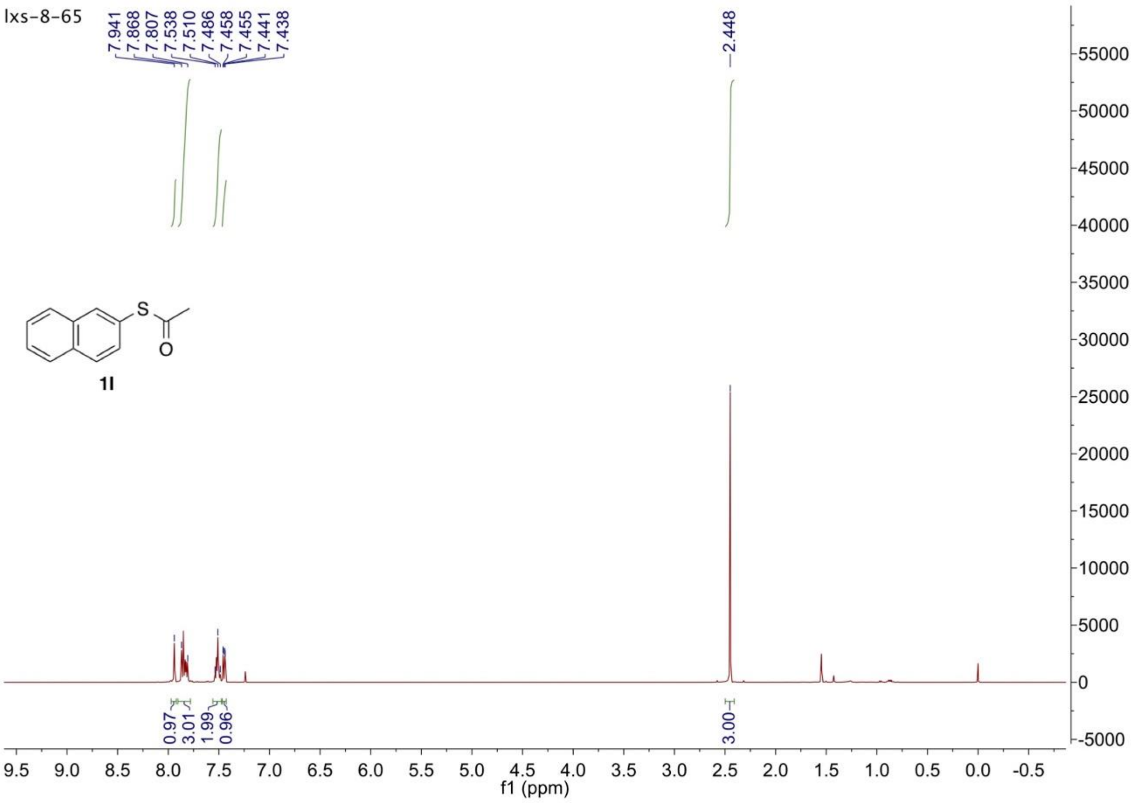

Supplementary Figure 57. ^{1}H NMR (500 MHz, $CDCl_3$) of compound **1l**

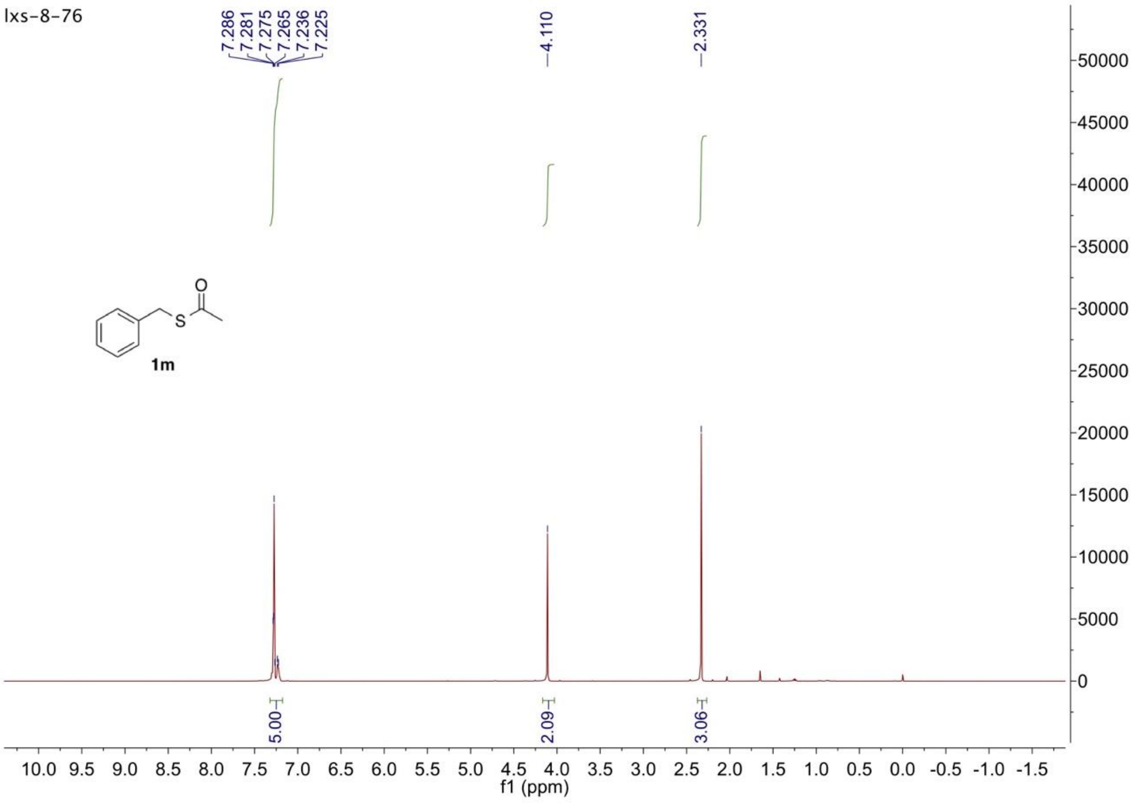

Supplementary Figure 58. ^{1}H NMR (500 MHz, $CDCl_3$) of compound **1m**

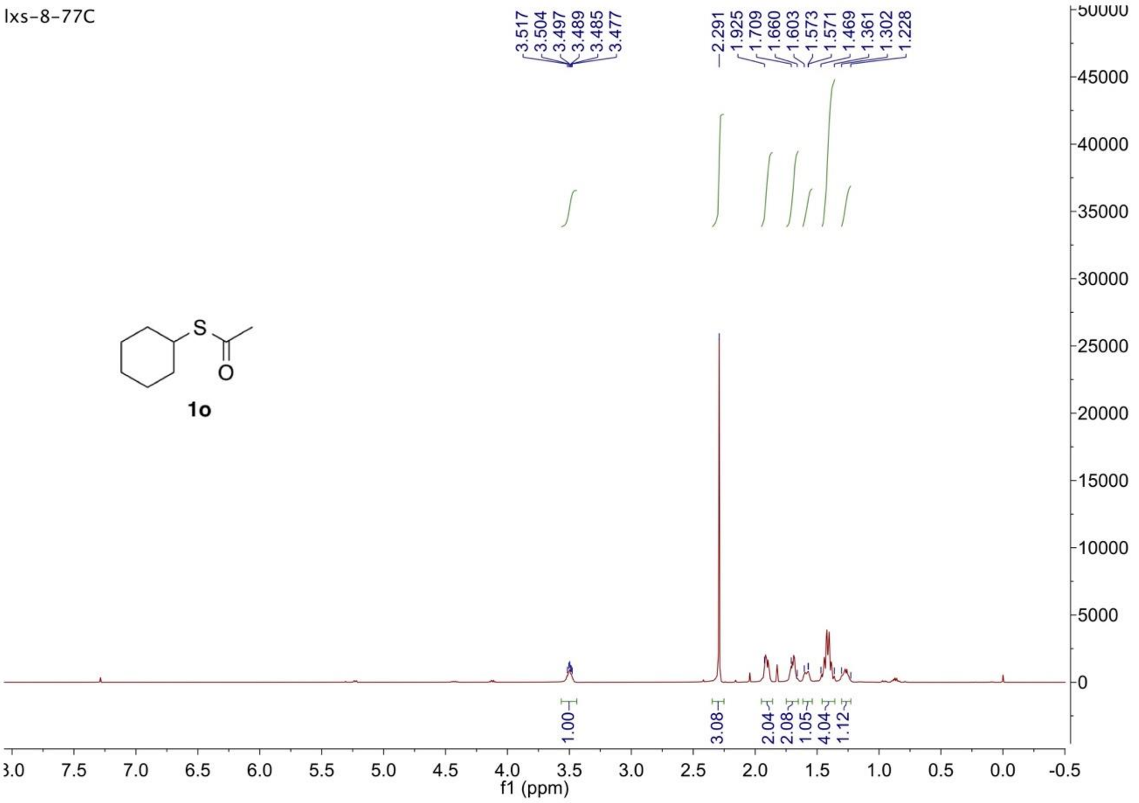

Supplementary Figure 59. ^{1}H NMR (500 MHz, $CDCl_3$) of compound **1o**

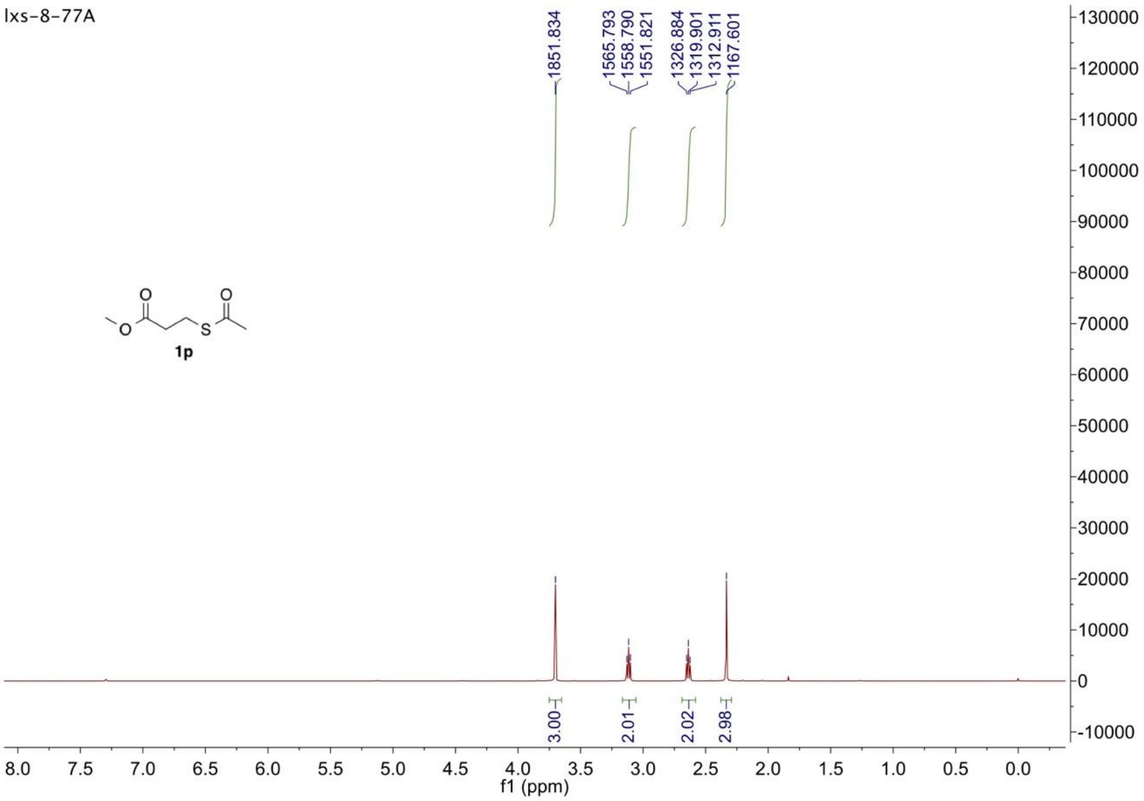

Supplementary Figure 60. ^{1}H NMR (500 MHz, $CDCl_3$) of compound **1p**

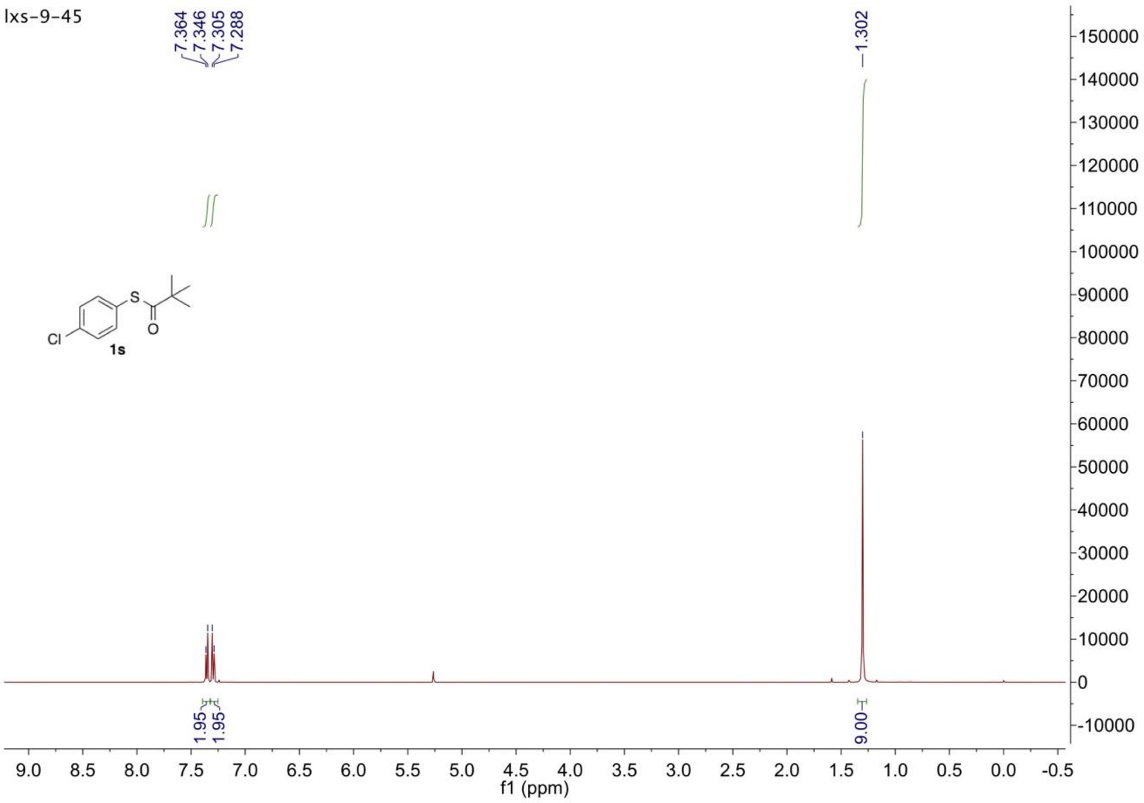

Supplementary Figure 61. ^{1}H NMR (500 MHz, $CDCl_3$) of compound **1s**

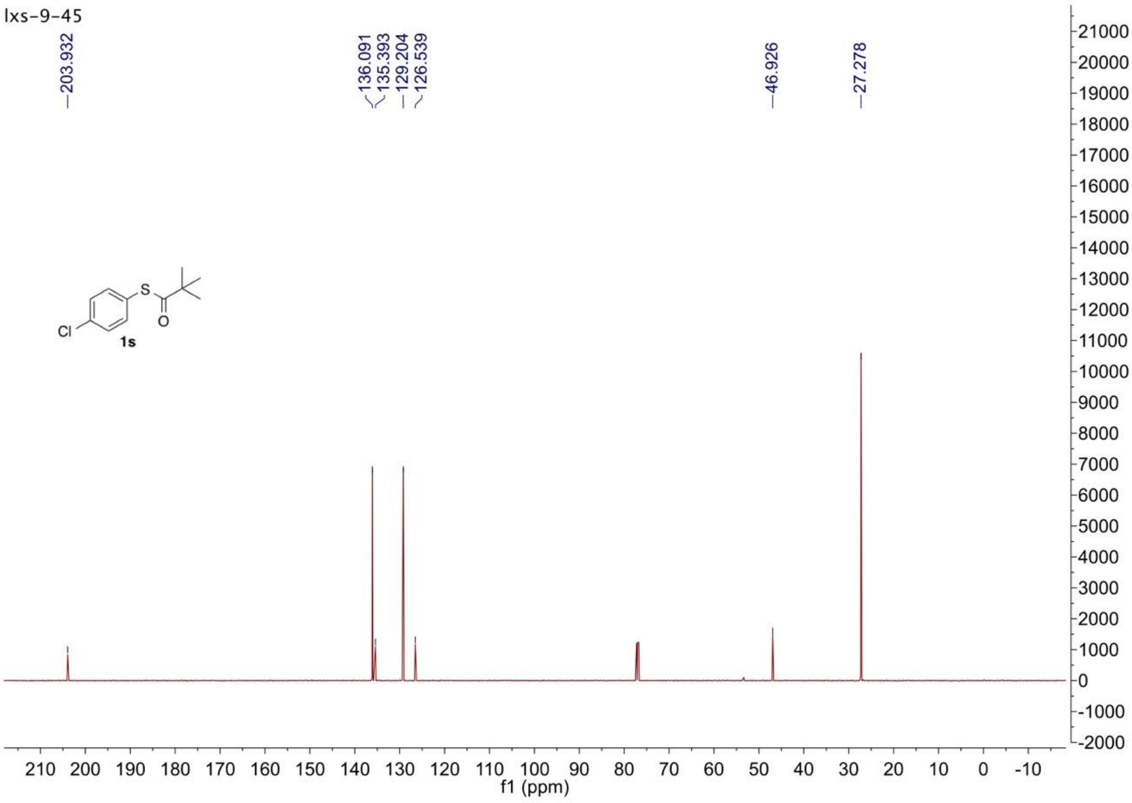

Supplementary Figure 62. ^{13}C NMR (125 MHz, $CDCl_3$) of compound **1s**

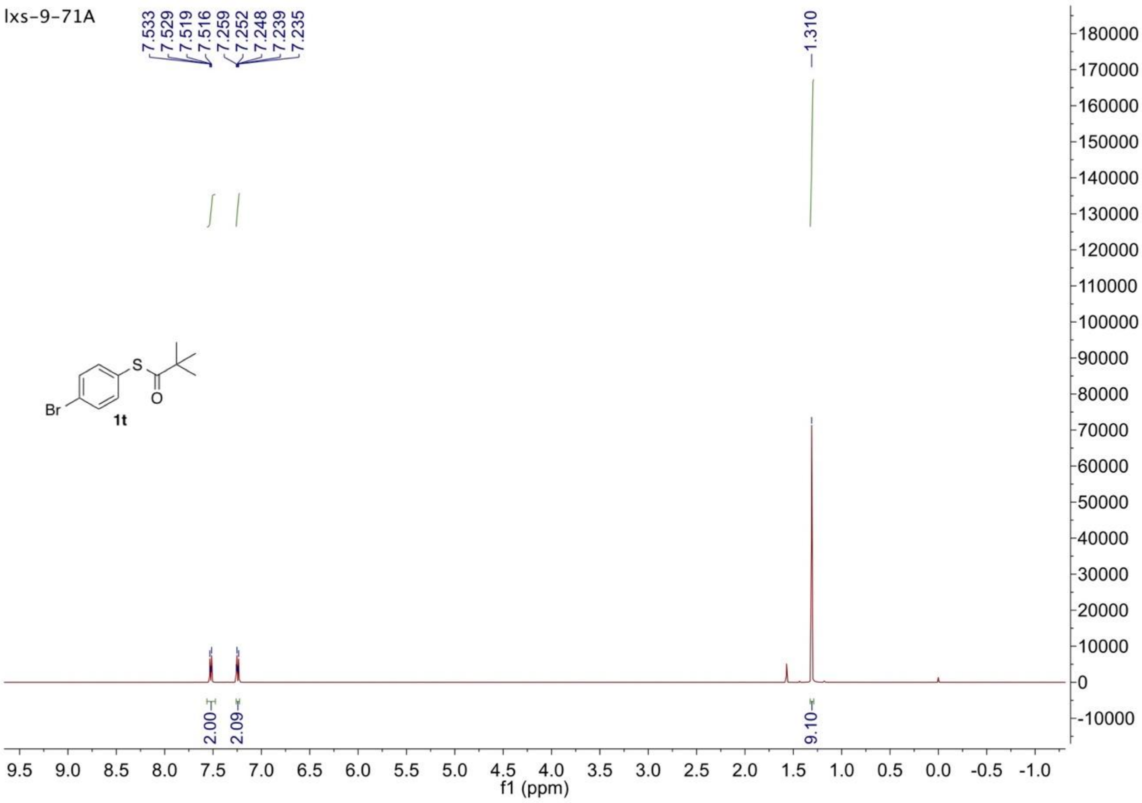

Supplementary Figure 63. ^{1}H NMR (500 MHz, $CDCl_3$) of compound **1t**

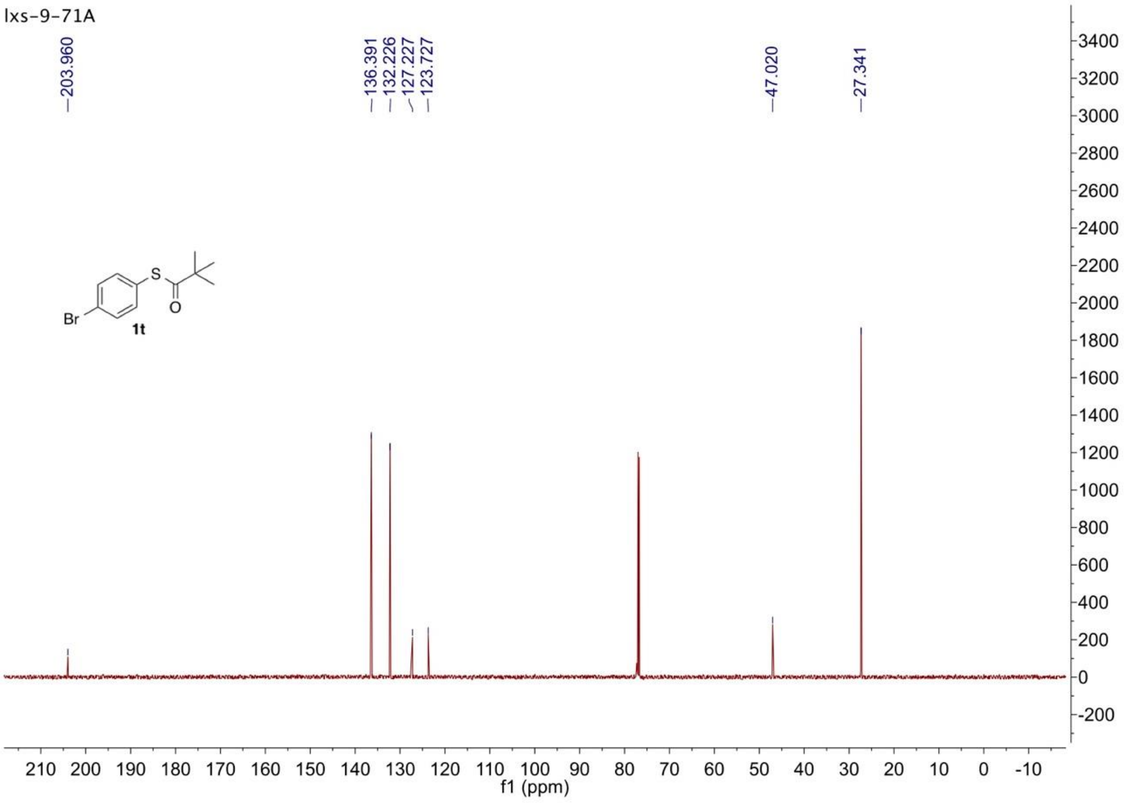

Supplementary Figure 64. ^{13}C NMR (125 MHz, $CDCl_3$) of compound **1t**

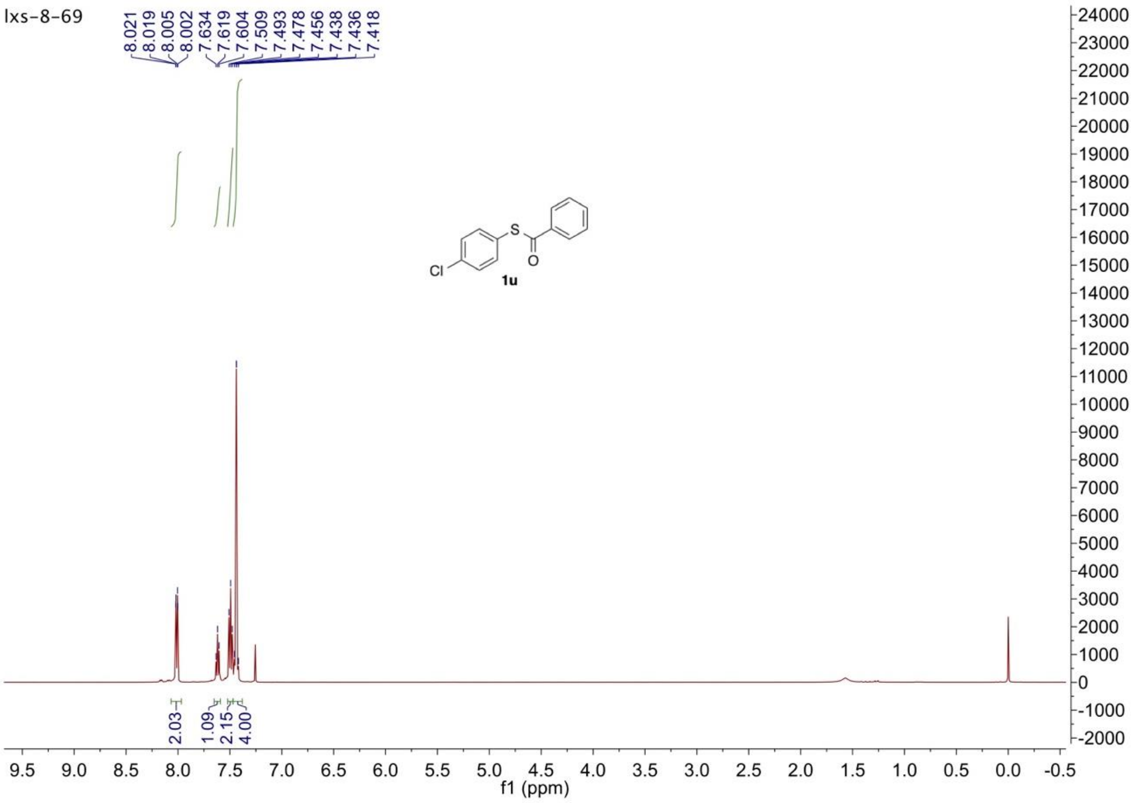

Supplementary Figure 65. ^{1}H NMR (500 MHz, $CDCl_3$) of compound **1u**

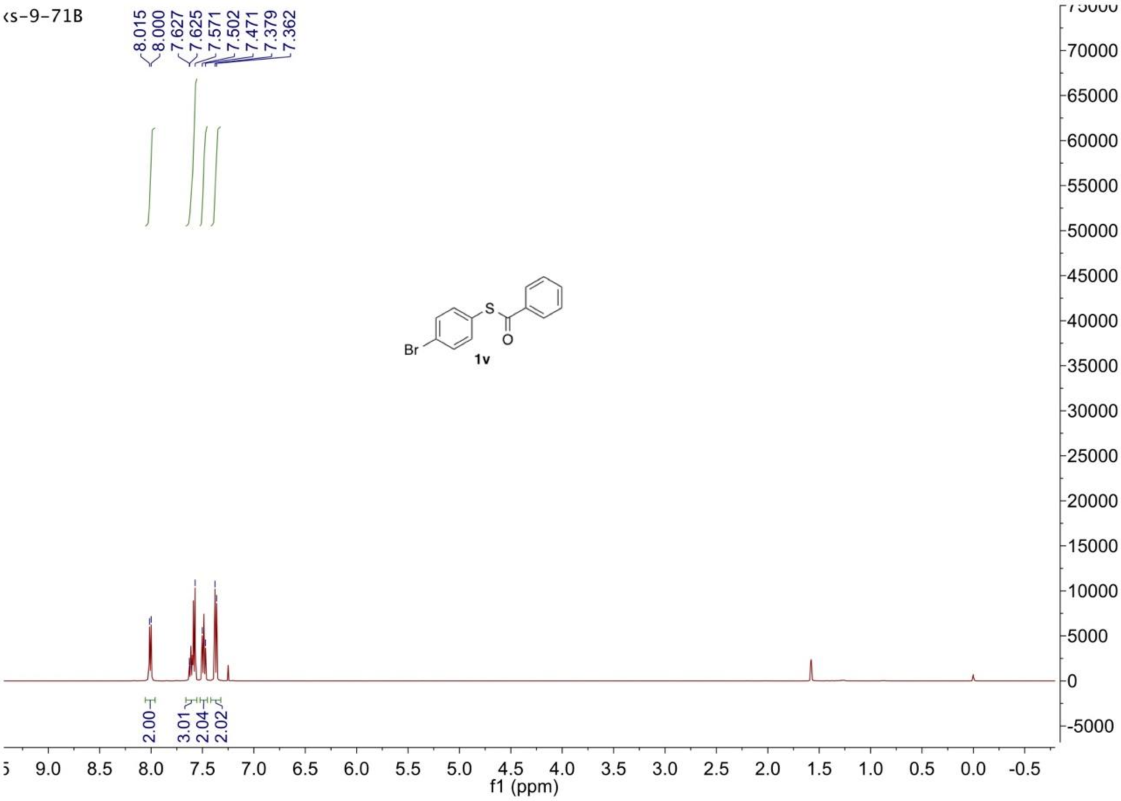

Supplementary Figure 66. ^{1}H NMR (500 MHz, $CDCl_3$) of compound **1v**

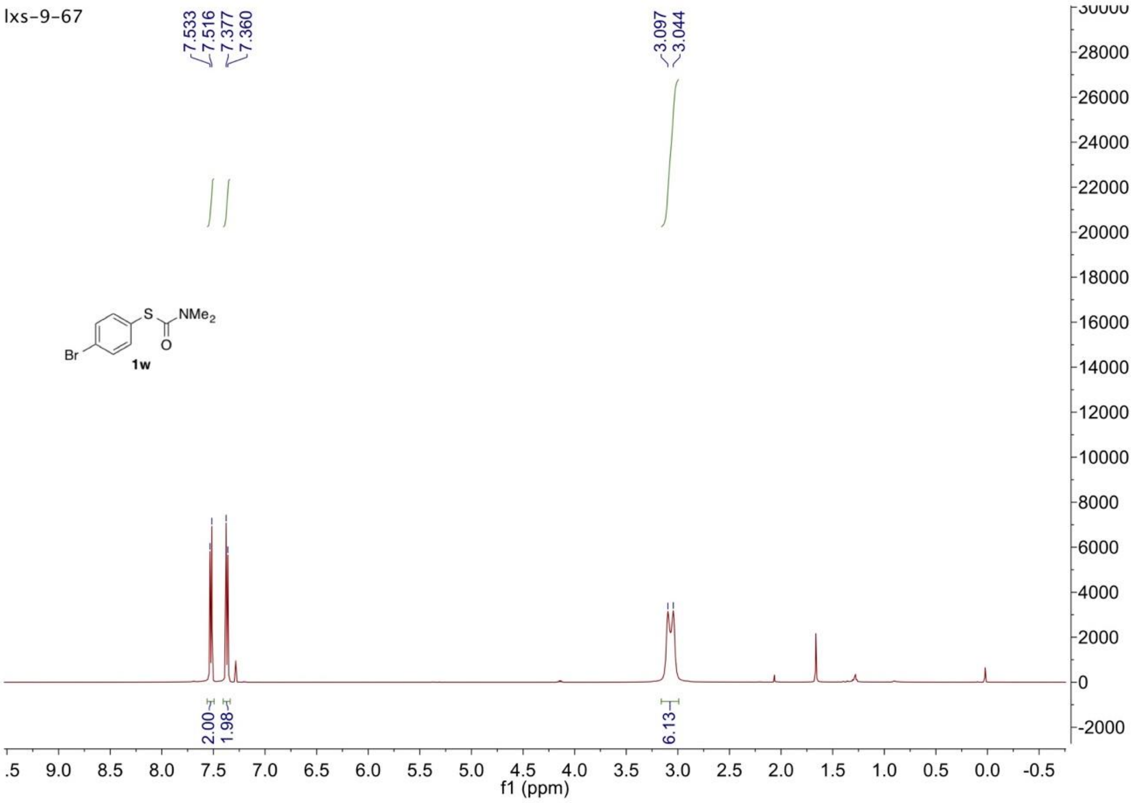

Supplementary Figure 67. ^{1}H NMR (500 MHz, $CDCl_3$) of compound **1w**

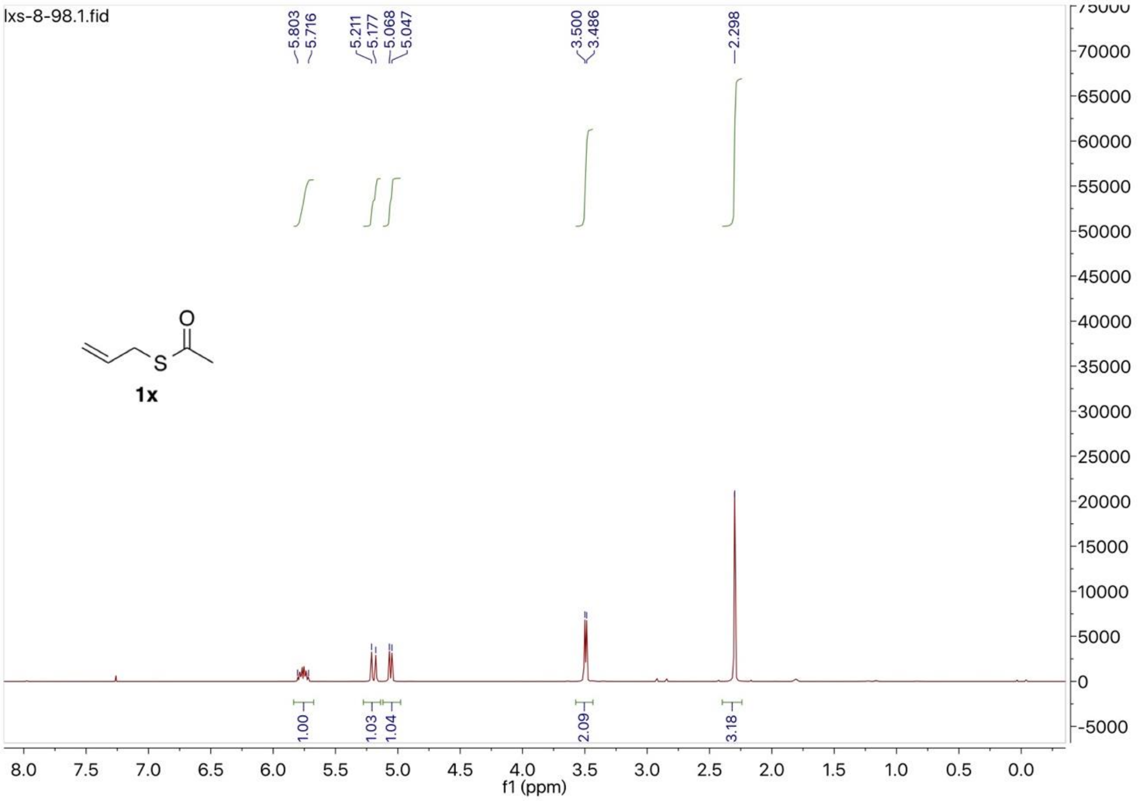

Supplementary Figure 68. ^{1}H NMR (500 MHz, $CDCl_3$) of compound **1x**

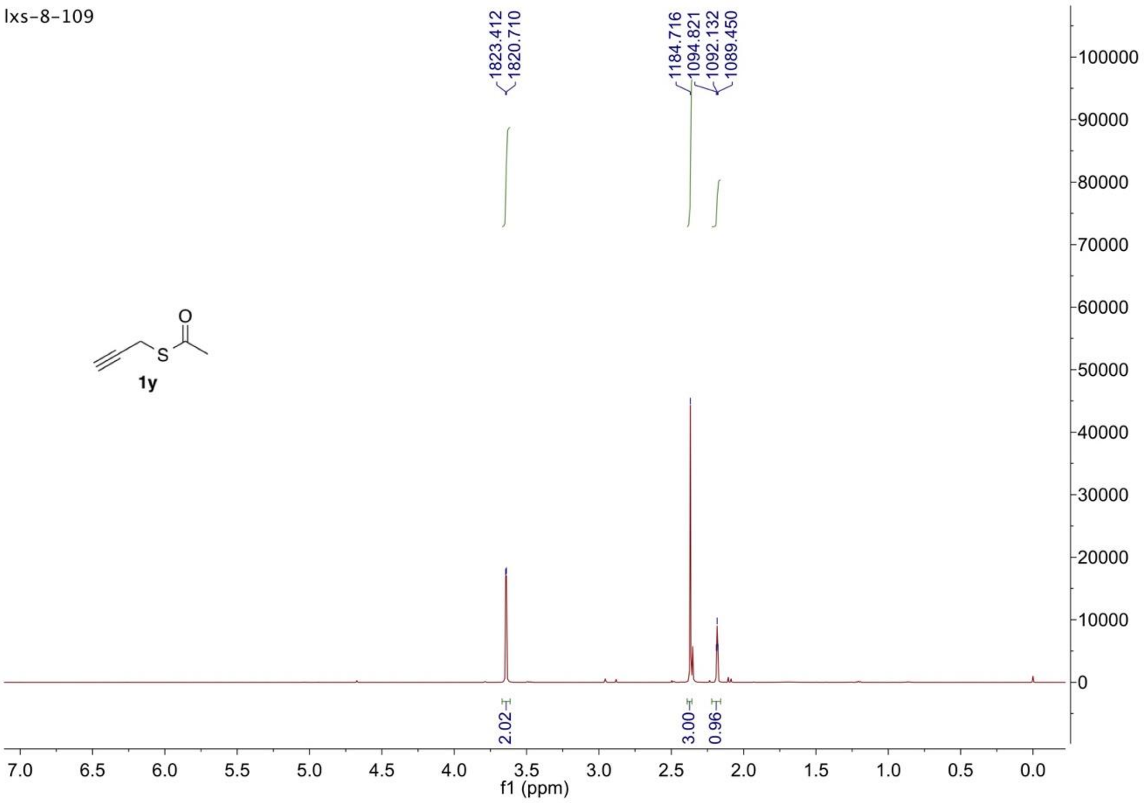

Supplementary Figure 69. ^{1}H NMR (500 MHz, $CDCl_3$) of compound **1y**

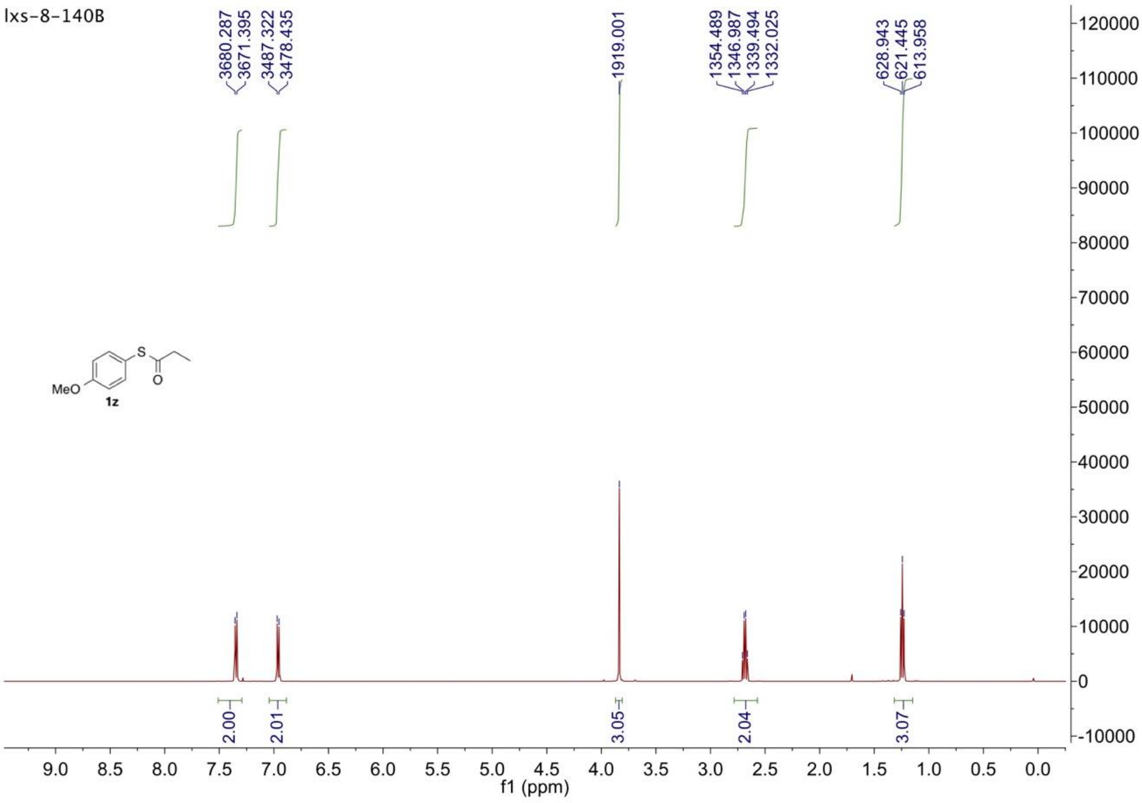

Supplementary Figure 70. ^{1}H NMR (500 MHz, $CDCl_3$) of compound **1z**

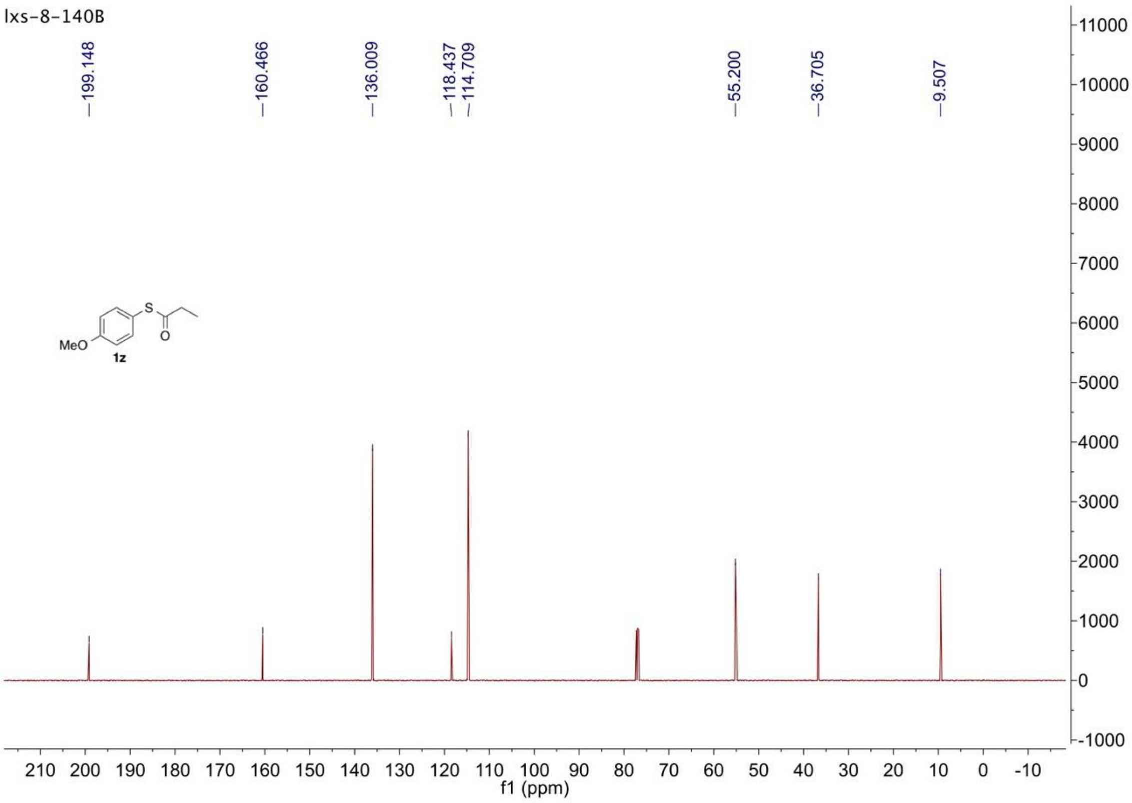

Supplementary Figure 71. ^{13}C NMR (125 MHz, $CDCl_3$) of compound **1z**

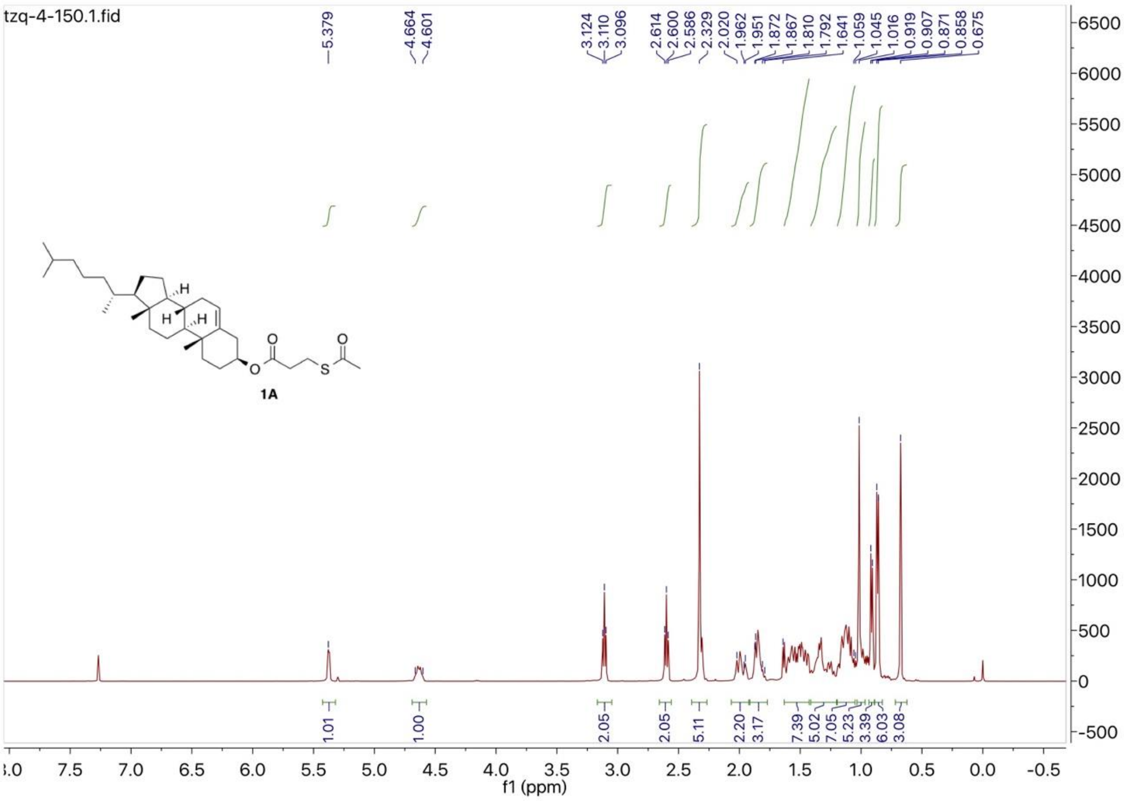

Supplementary Figure 72. ^{1}H NMR (500 MHz, $CDCl_3$) of compound **1A**

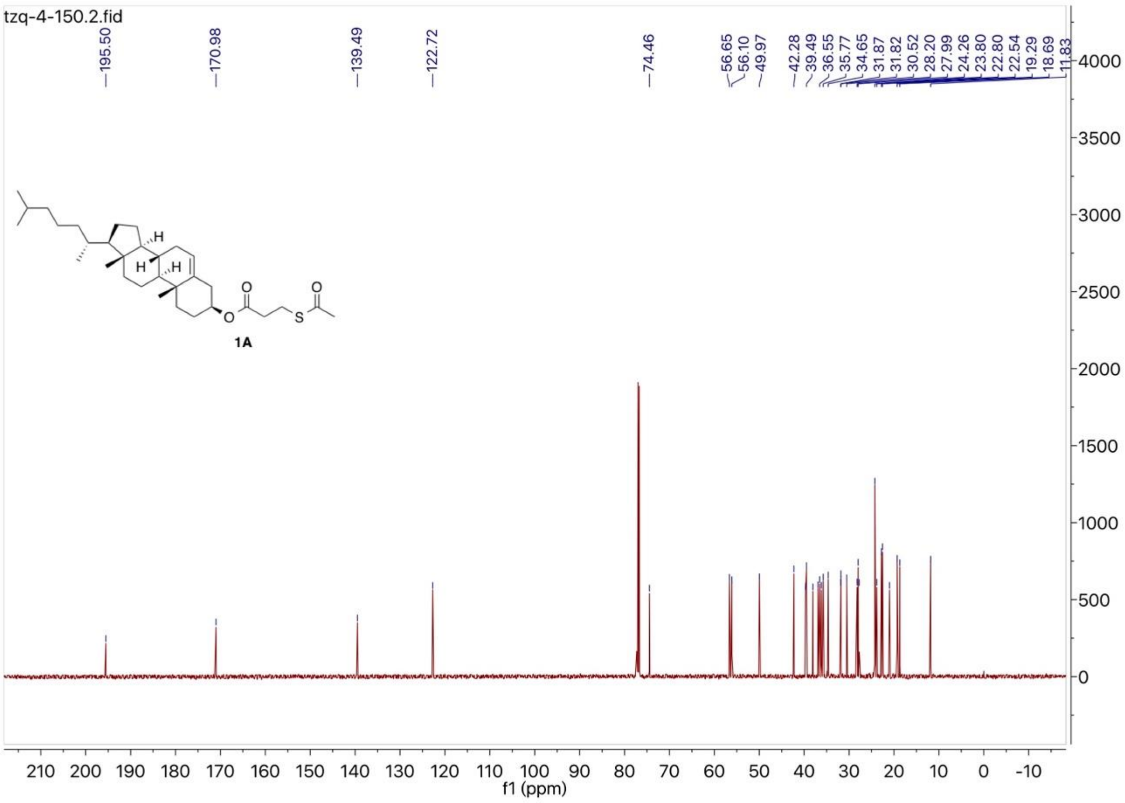

Supplementary Figure 73. ^{13}C NMR (125 MHz, $CDCl_3$) of compound **1A**

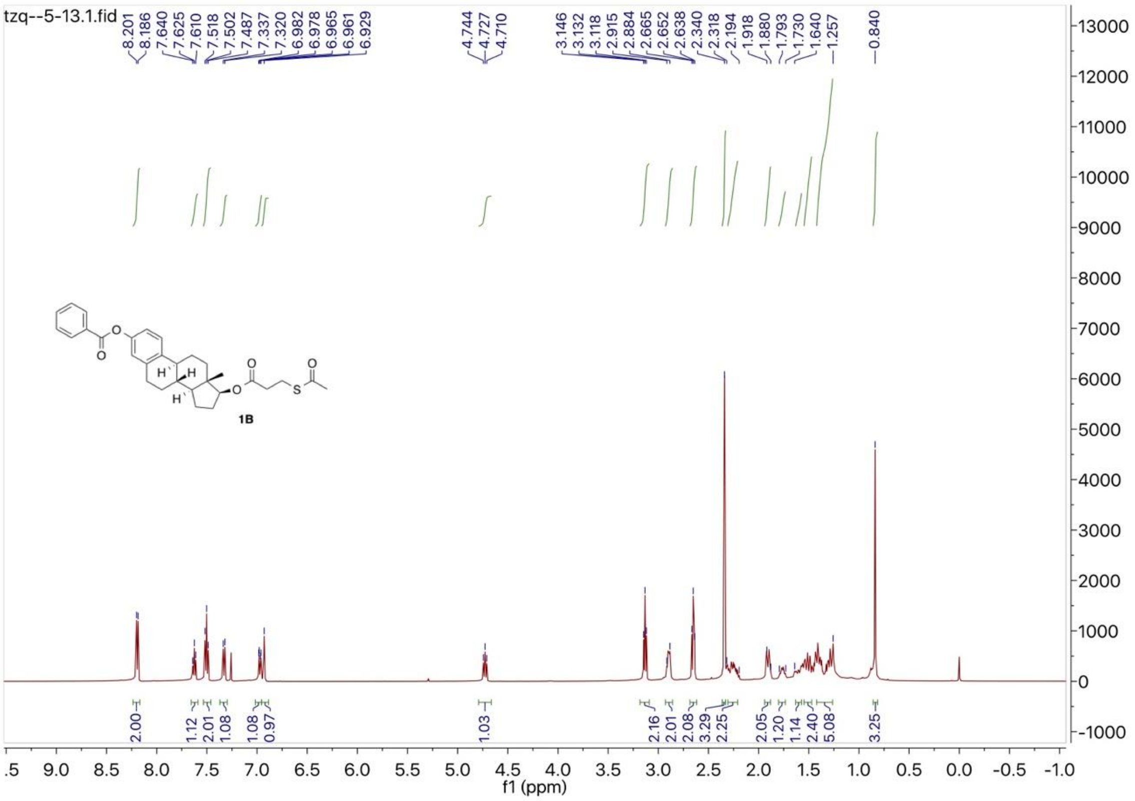

Supplementary Figure 74. ^{1}H NMR (500 MHz, $CDCl_3$) of compound **1B**

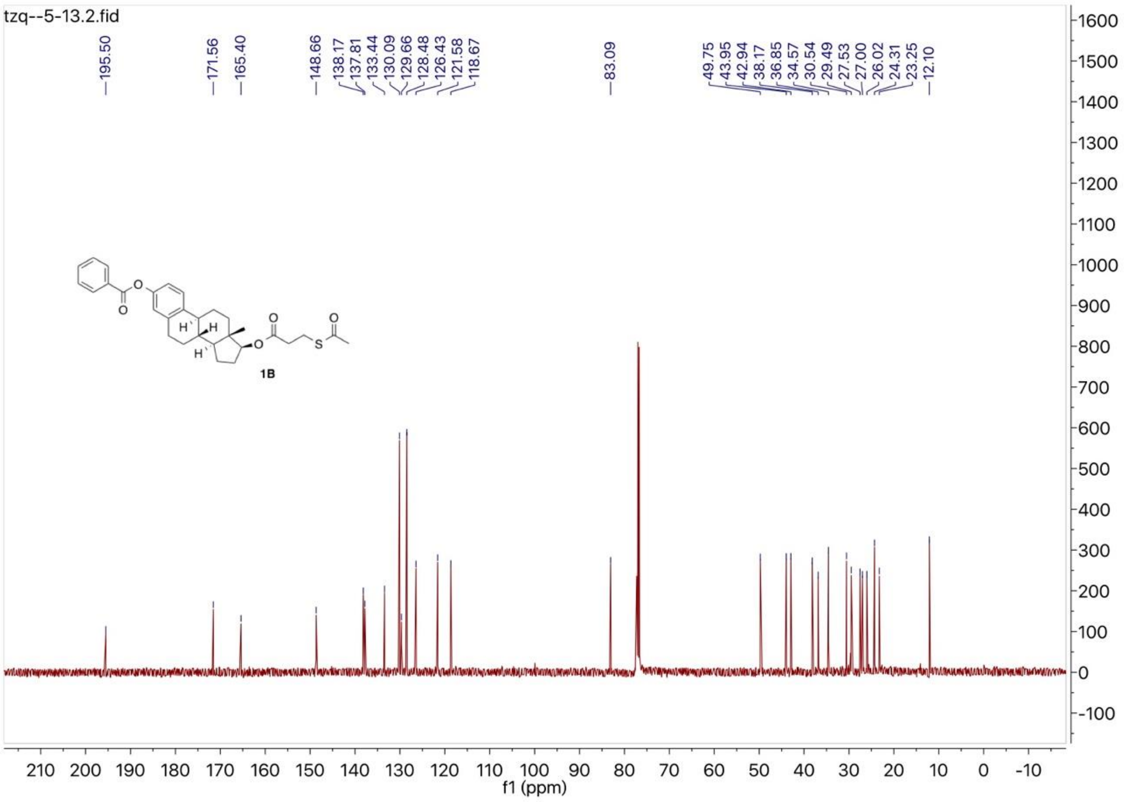

Supplementary Figure 75. ^{13}C NMR (125 MHz, $CDCl_3$) of compound **1B**

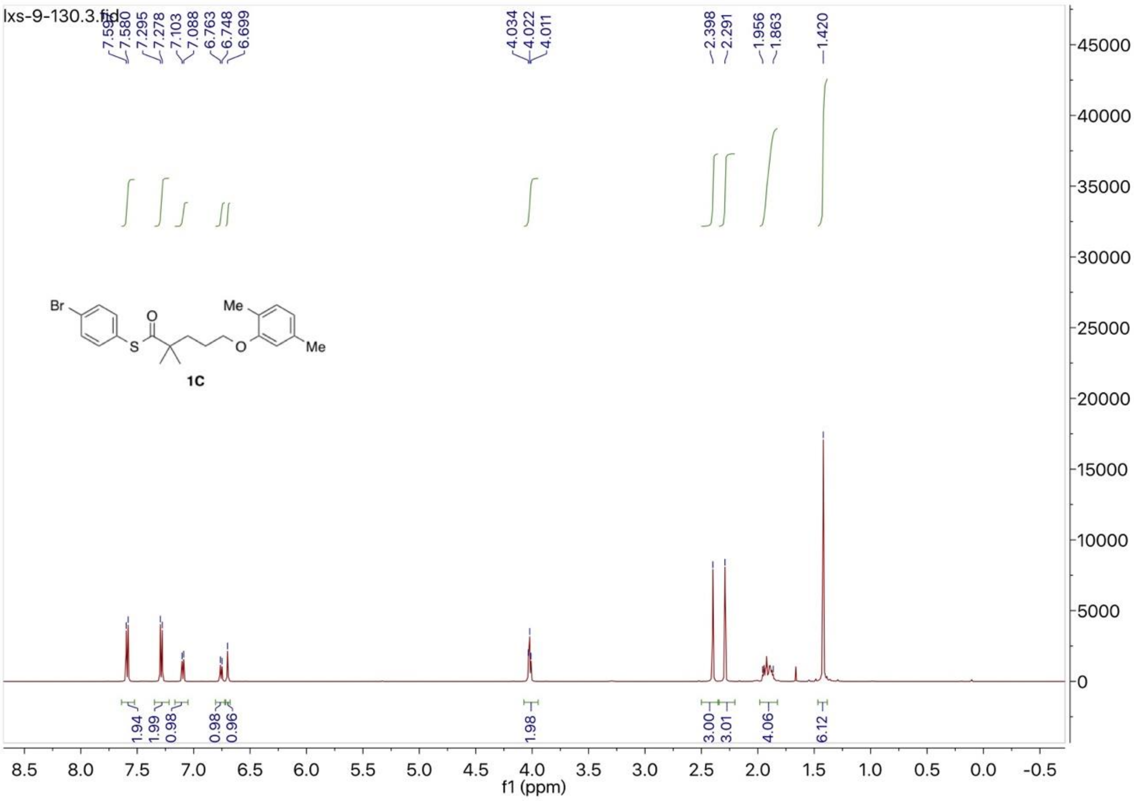

Supplementary Figure 76. ^{1}H NMR (500 MHz, $CDCl_3$) of compound **1C**

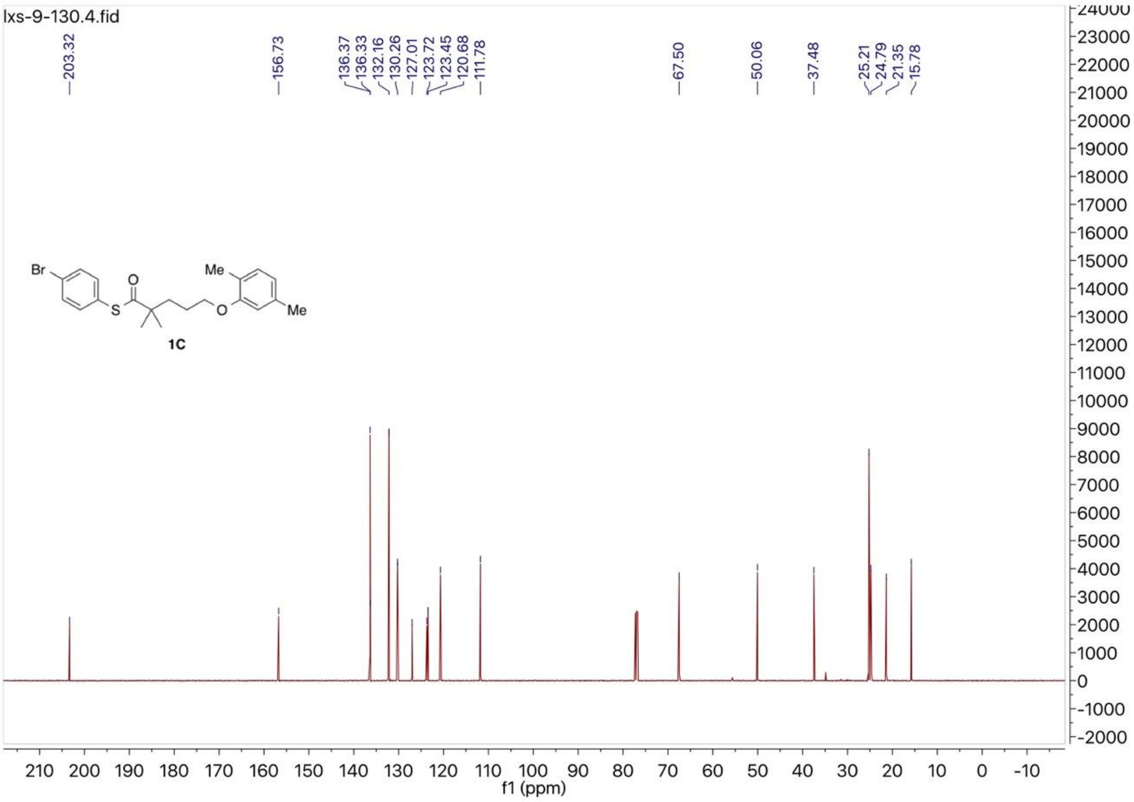

Supplementary Figure 77. ^{13}C NMR (125 MHz, $CDCl_3$) of compound **1C**

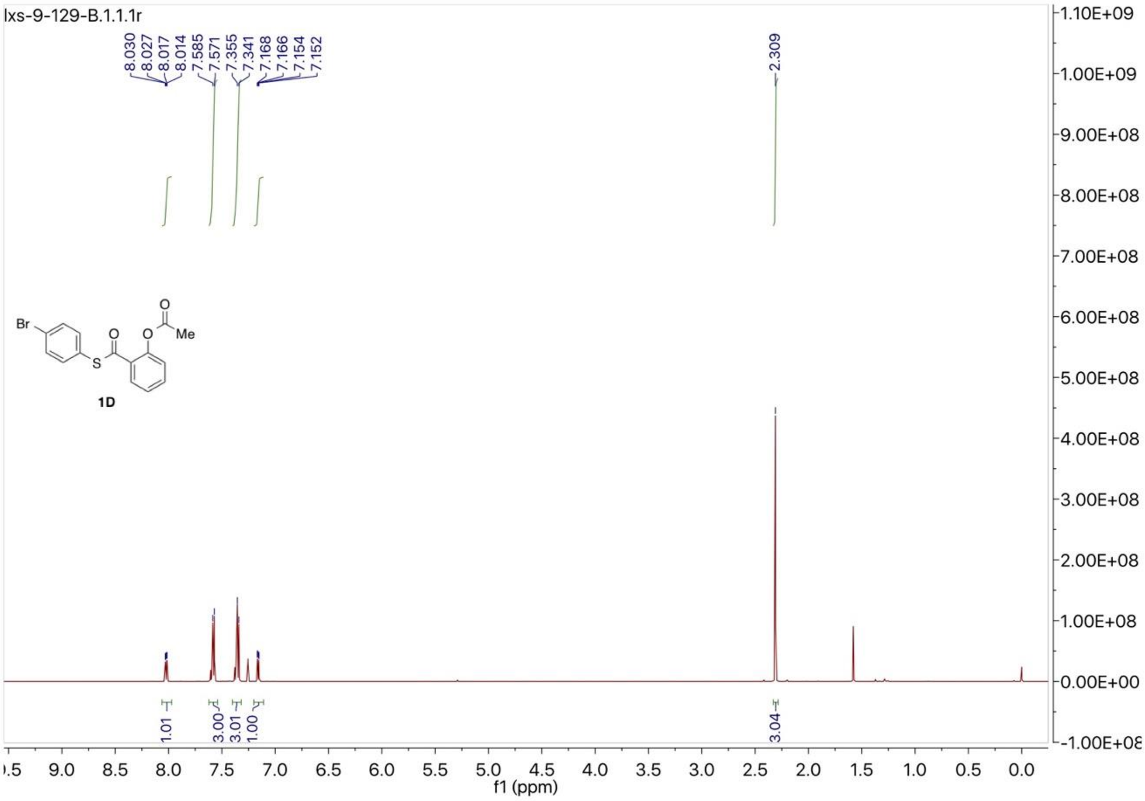

Supplementary Figure 78. ^{1}H NMR (600 MHz, $CDCl_3$) of compound **1D**

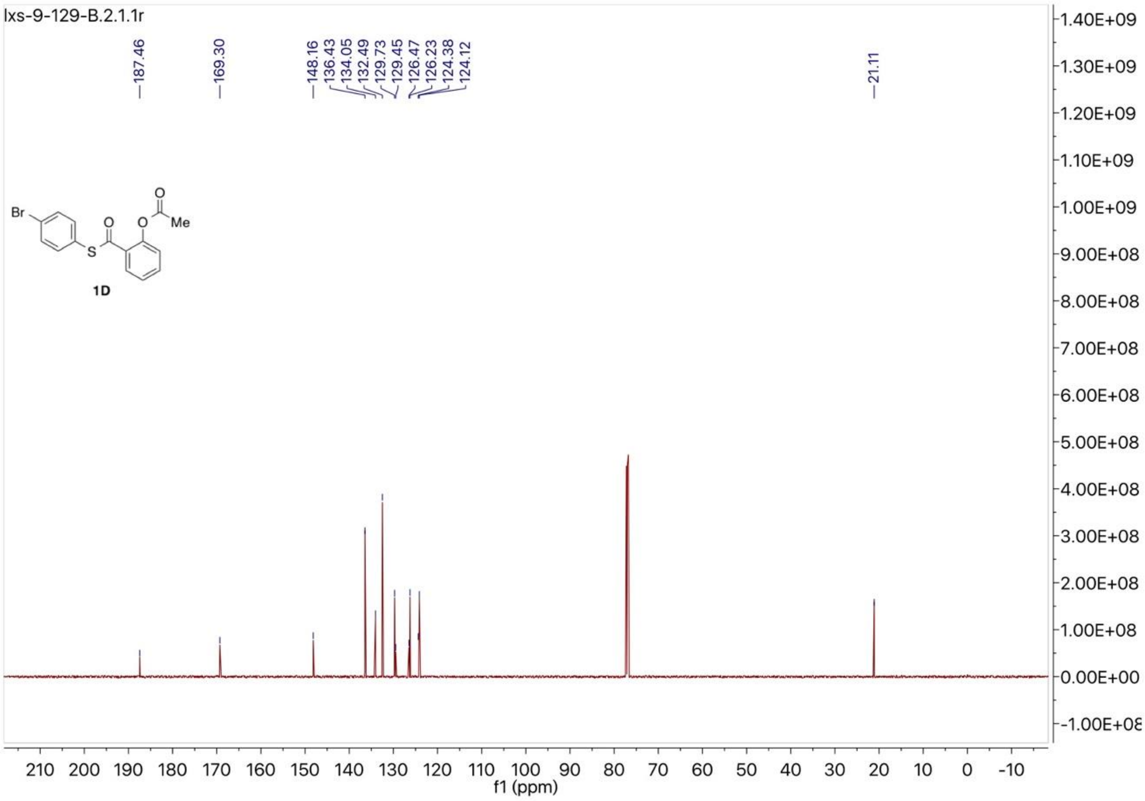

Supplementary Figure 79. ^{13}C NMR (150 MHz, $CDCl_3$) of compound **1D**

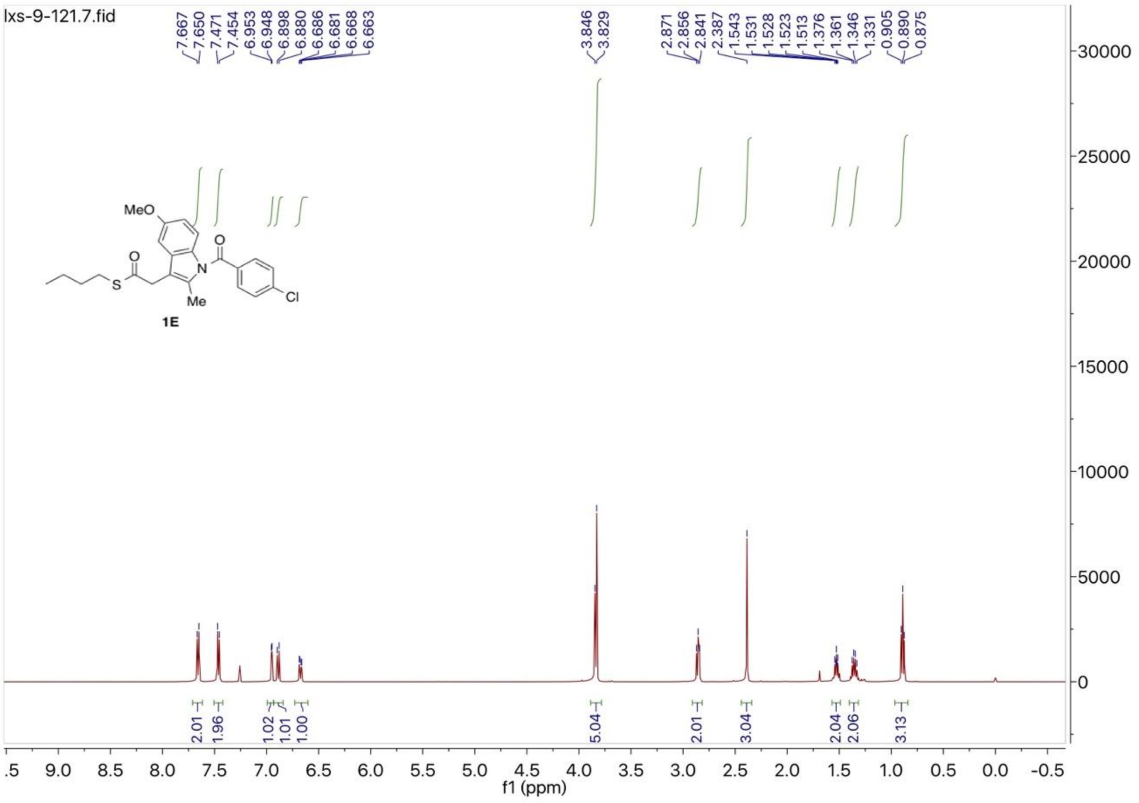

Supplementary Figure 80. ^{1}H NMR (500 MHz, $CDCl_3$) of compound **1E**

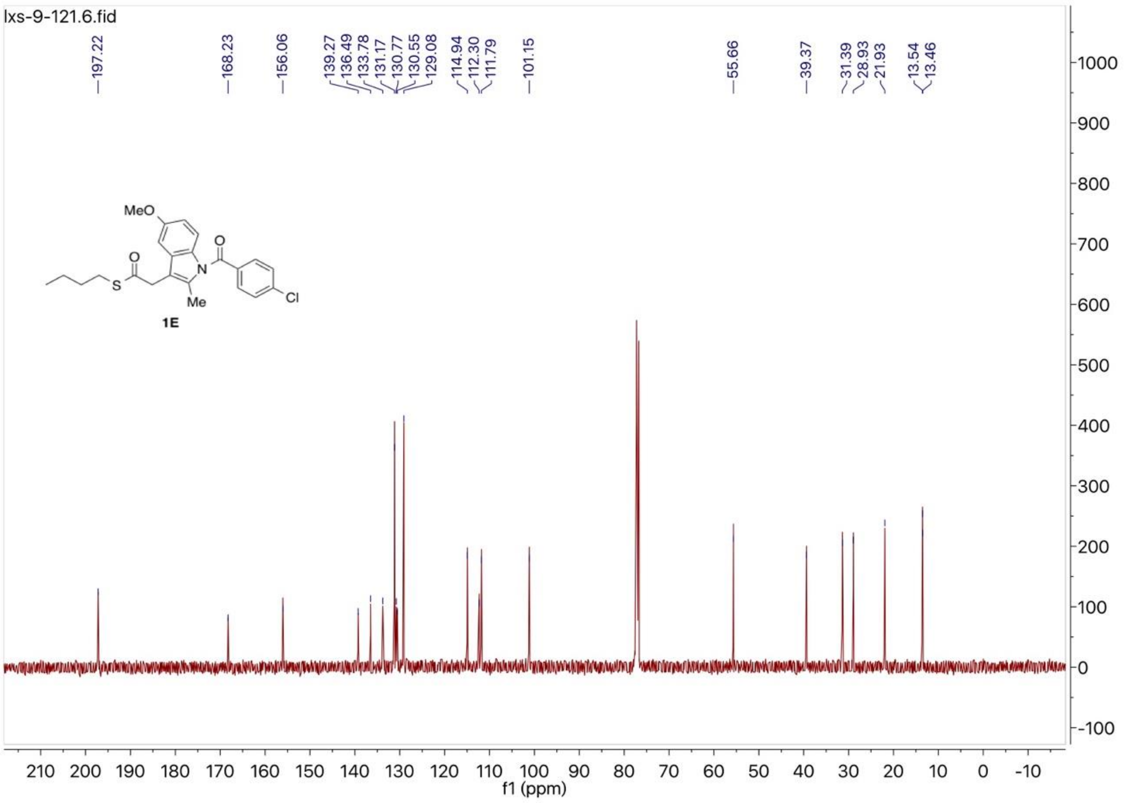

Supplementary Figure 81. ^{13}C NMR (125 MHz, $CDCl_3$) of compound **1E**

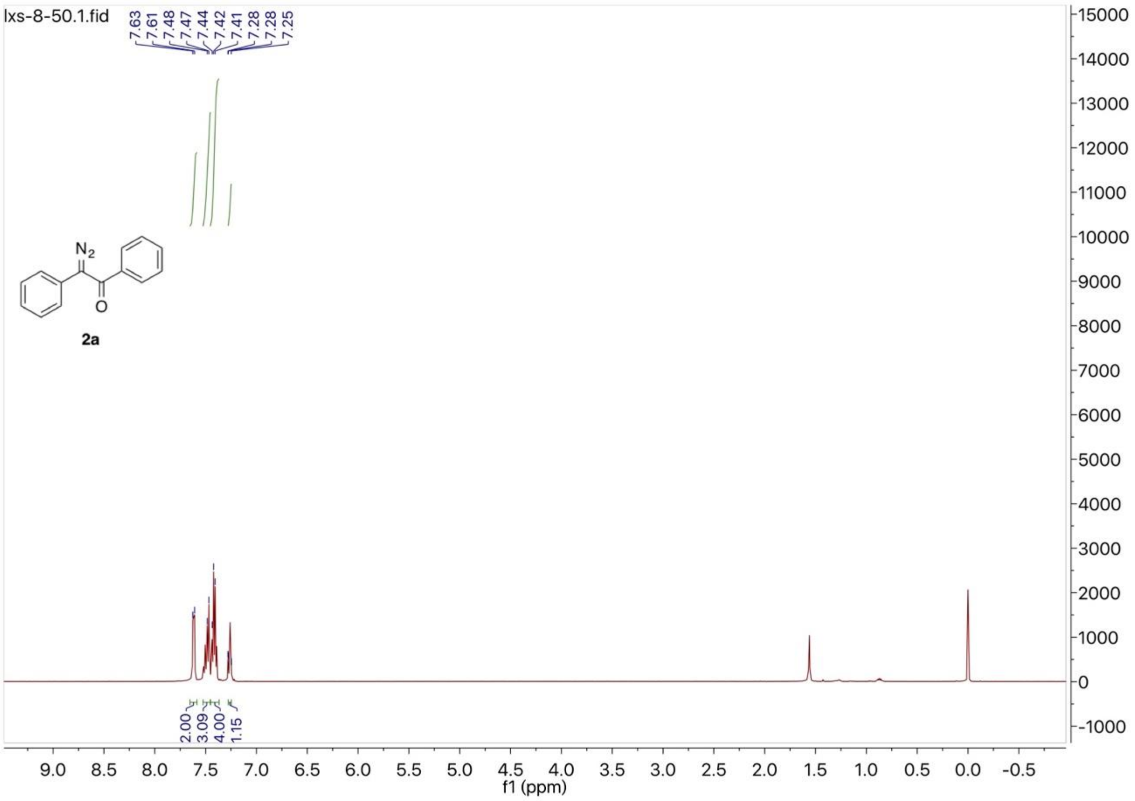

Supplementary Figure 82. ^{1}H NMR (500 MHz, $CDCl_3$) of compound **2a**

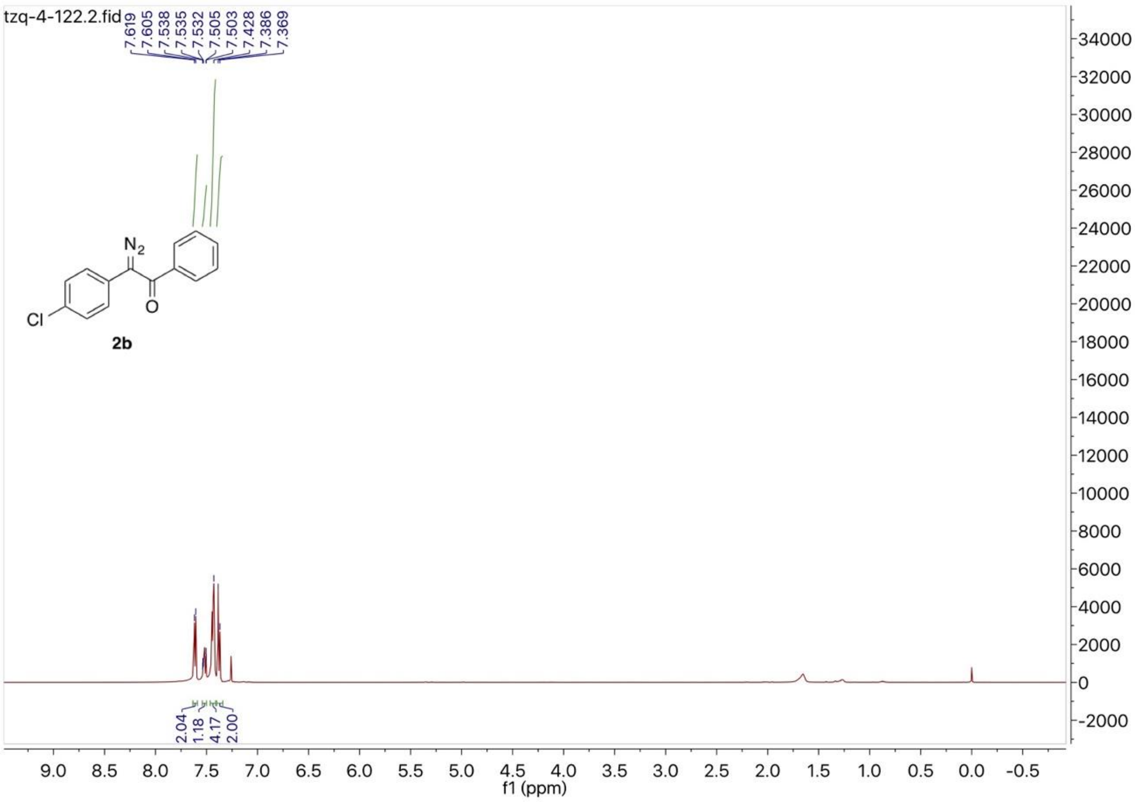

Supplementary Figure 83. ^{1}H NMR (500 MHz, $CDCl_3$) of compound **2b**

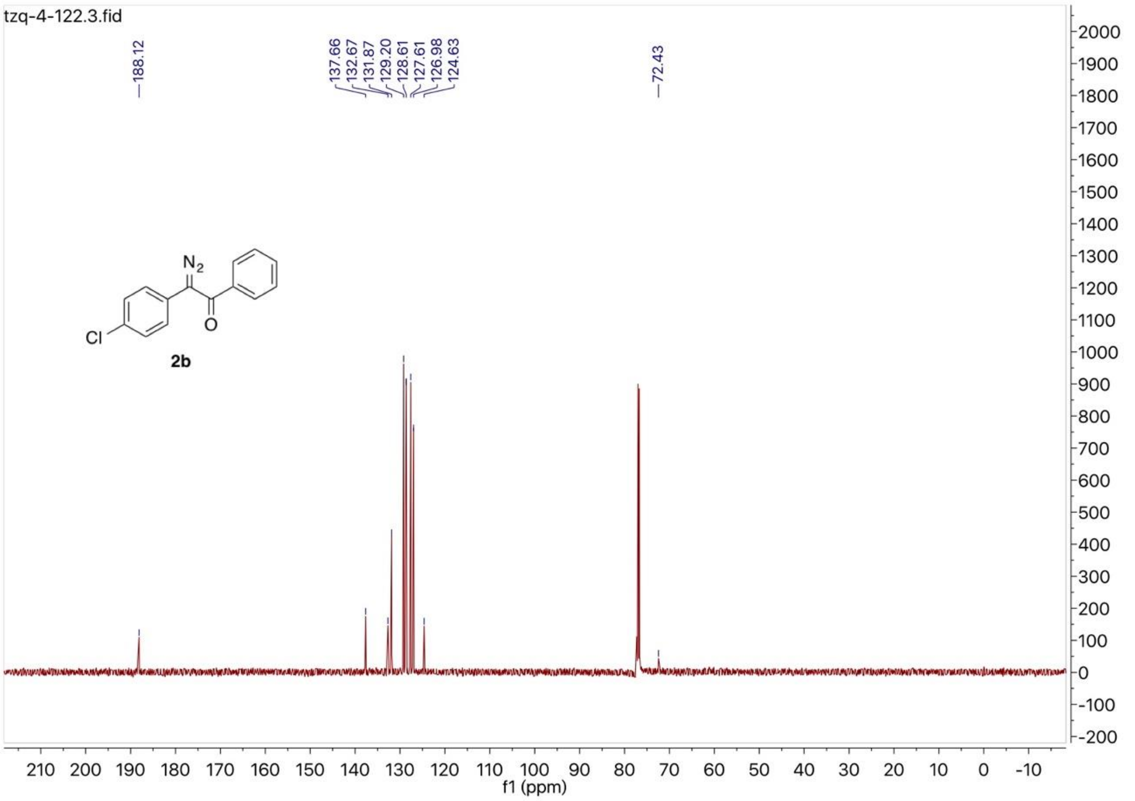

Supplementary Figure 84. ^{13}C NMR (125 MHz, $CDCl_3$) of compound **2b**

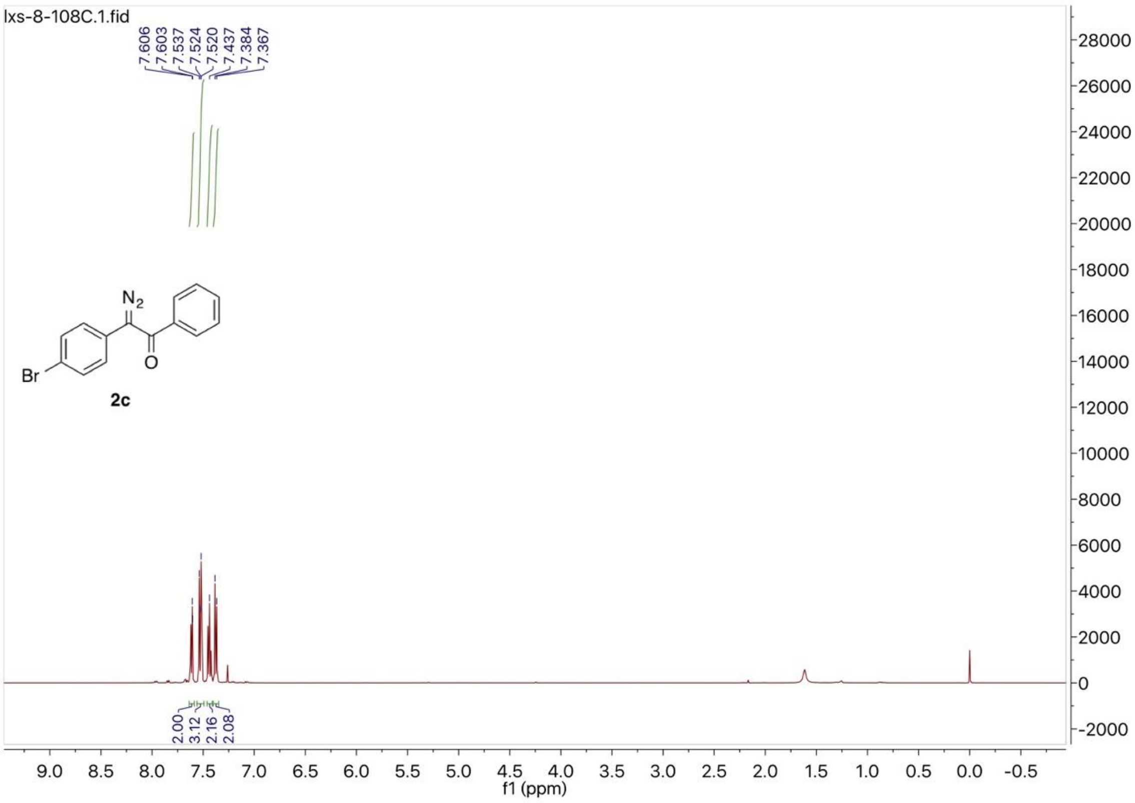

Supplementary Figure 85. ^{1}H NMR (500 MHz, $CDCl_3$) of compound **2c**

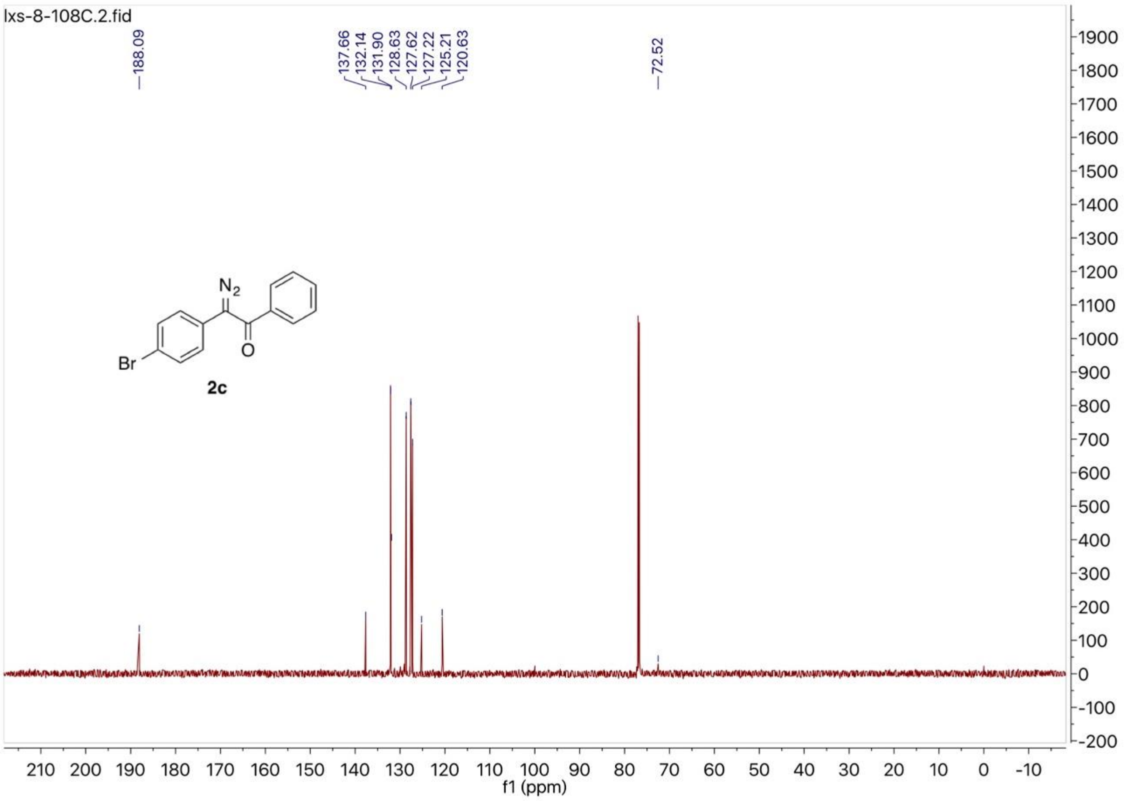

Supplementary Figure 86. ^{13}C NMR (125 MHz, $CDCl_3$) of compound **2c**

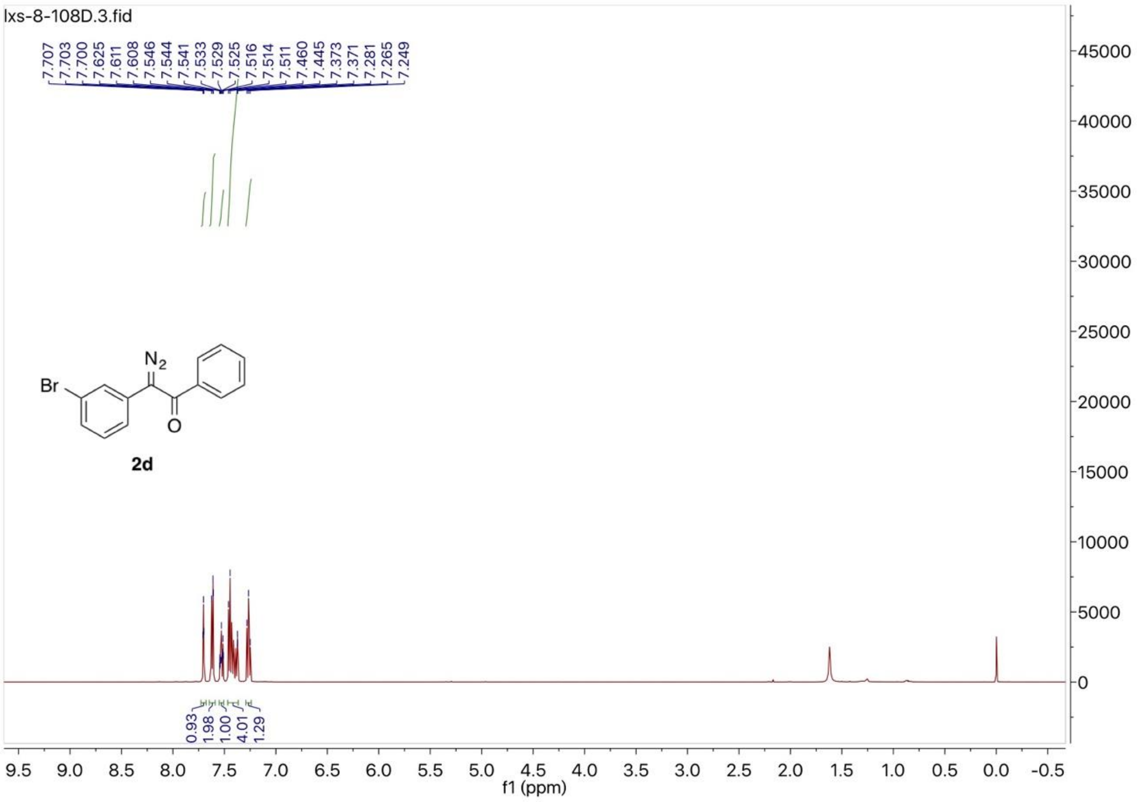

Supplementary Figure 87. ^{1}H NMR (500 MHz, $CDCl_3$) of compound **2d**

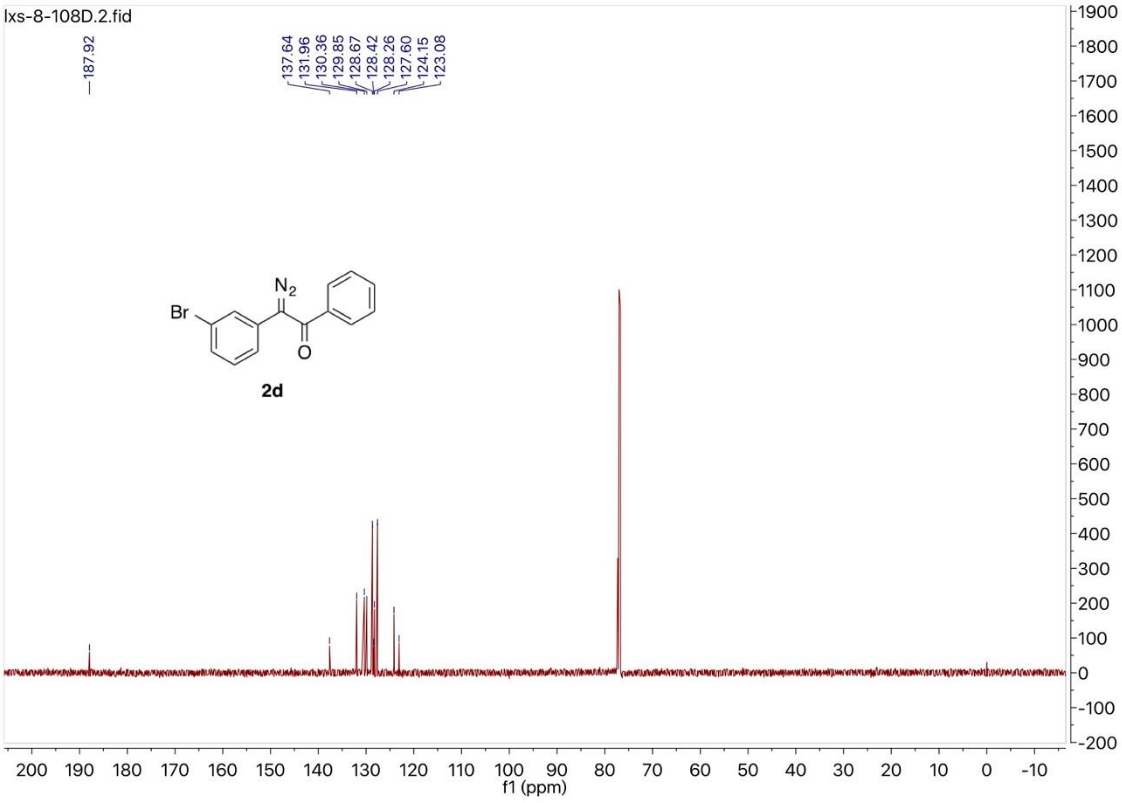

Supplementary Figure 88. ^{13}C NMR (125 MHz, $CDCl_3$) of compound **2d**

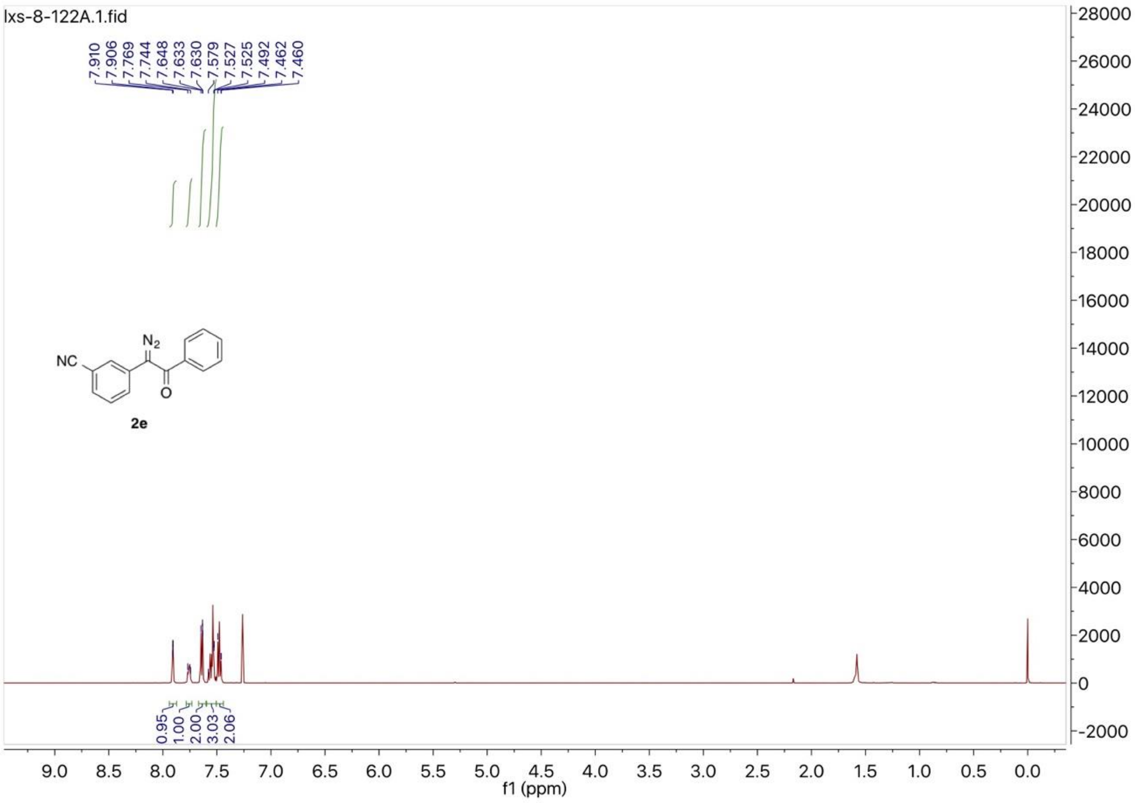

Supplementary Figure 89. ^{1}H NMR (500 MHz, $CDCl_3$) of compound **2e**

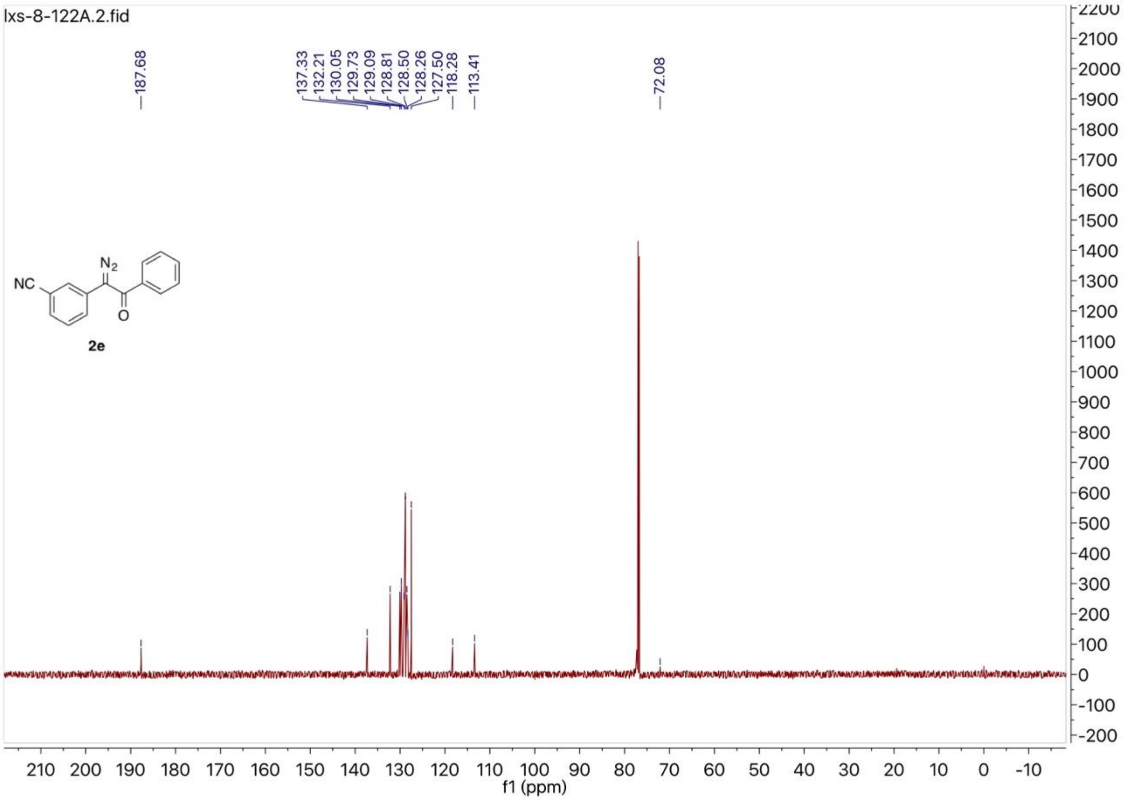

Supplementary Figure 90. ^{13}C NMR (125 MHz, $CDCl_3$) of compound **2e**

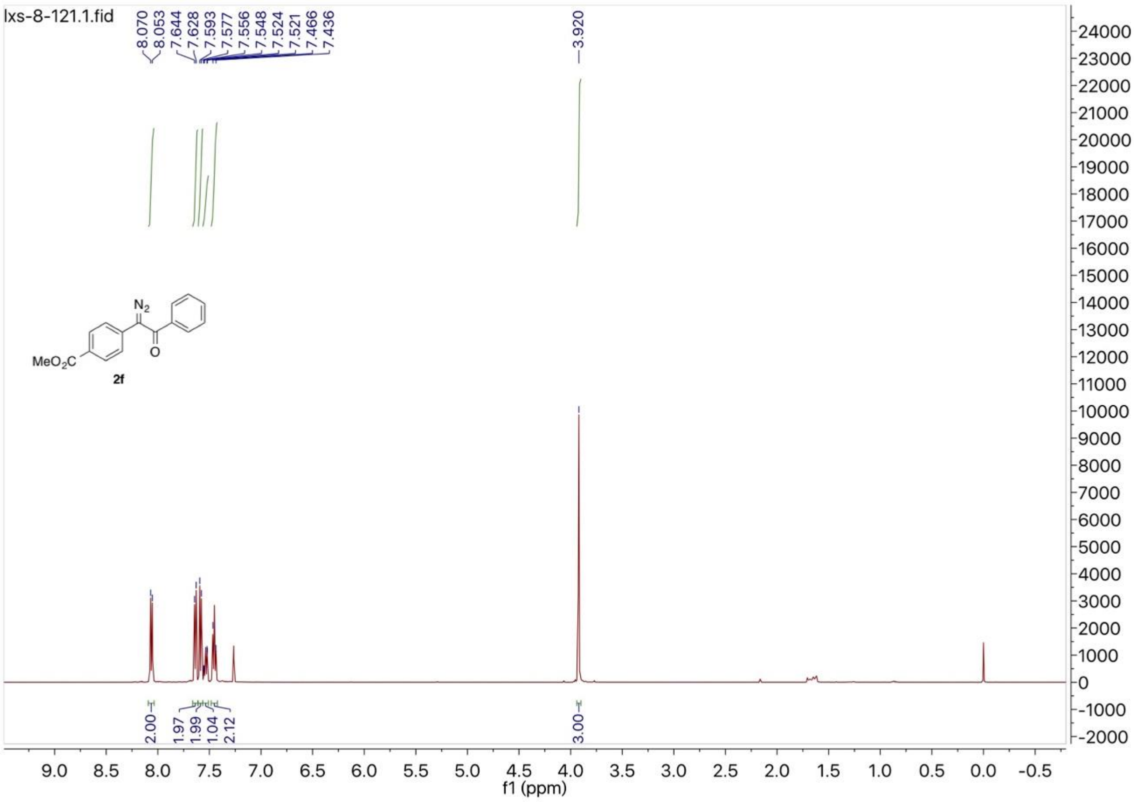

Supplementary Figure 91. ^{1}H NMR (500 MHz, $CDCl_3$) of compound **2f**

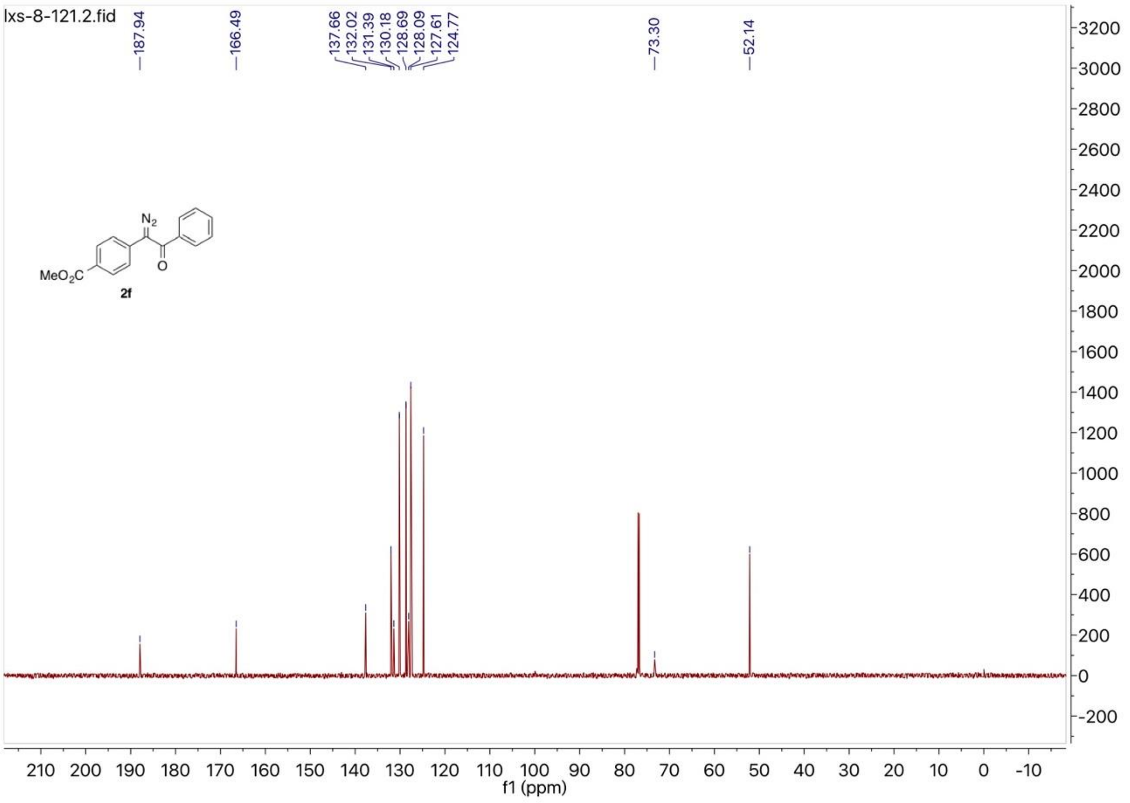

Supplementary Figure 92. ^{13}C NMR (125 MHz, $CDCl_3$) of compound **2f**

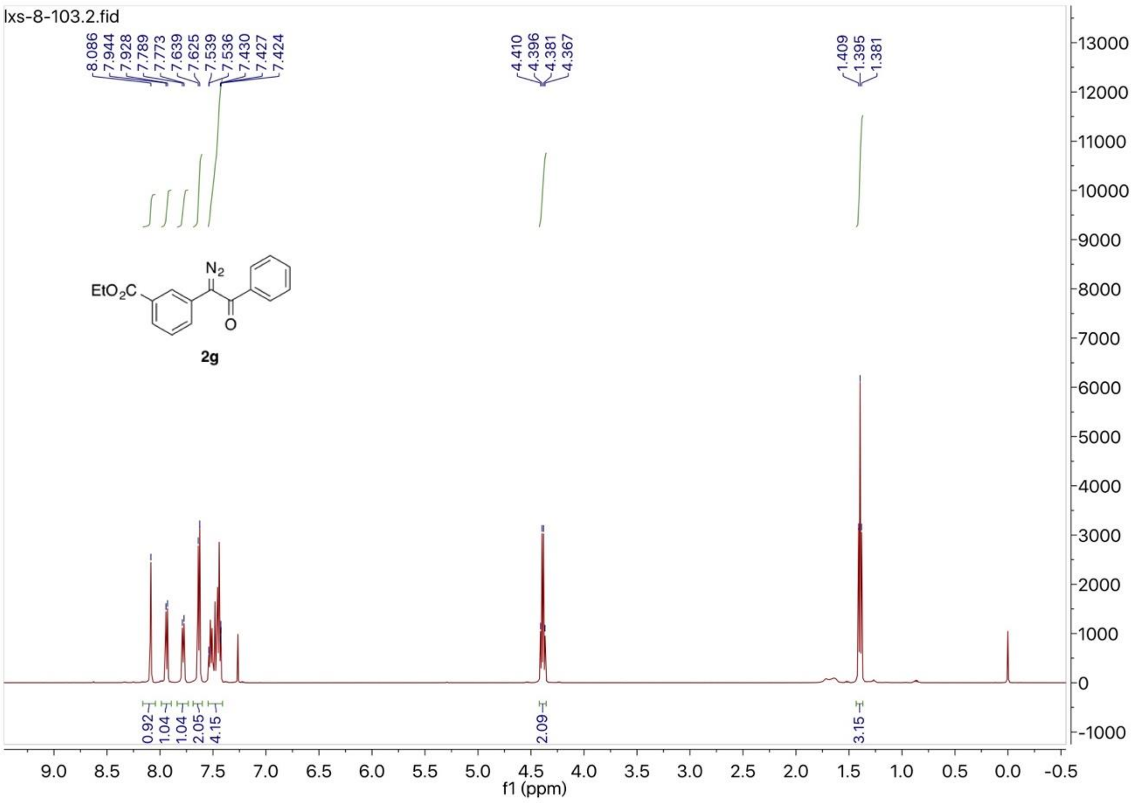

Supplementary Figure 93. ^{1}H NMR (500 MHz, $CDCl_3$) of compound **2g**

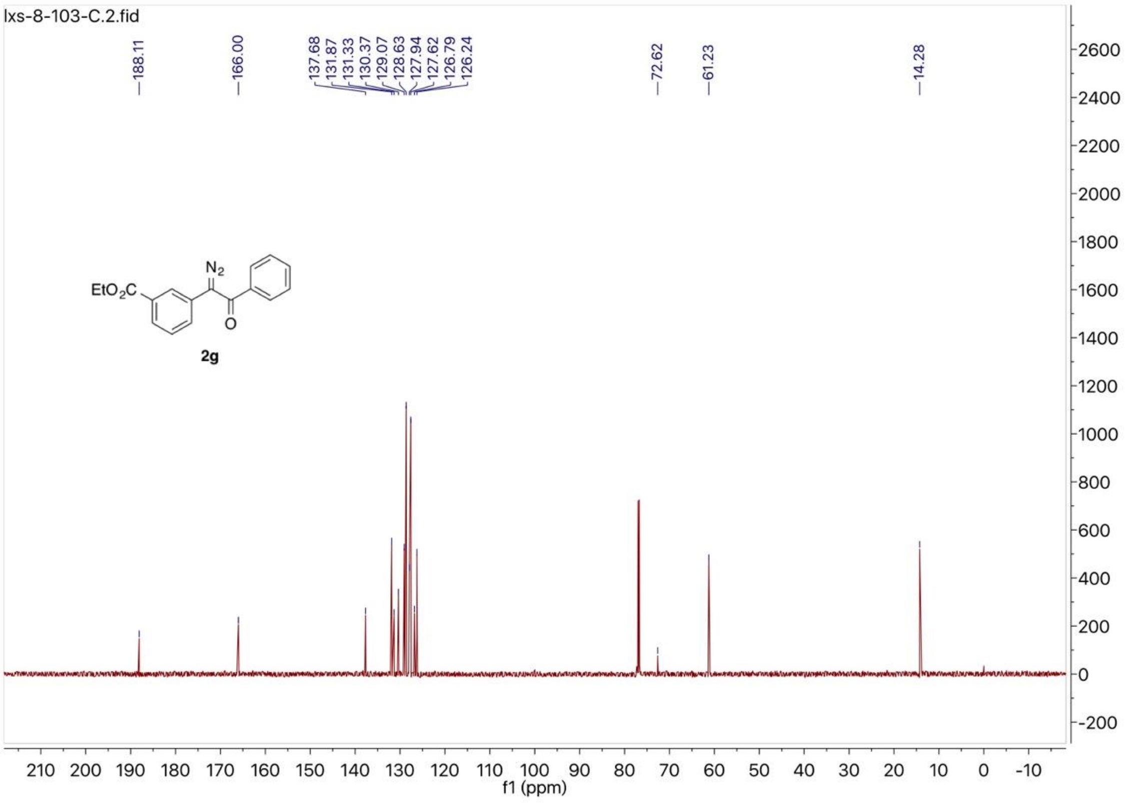

Supplementary Figure 94. ^{13}C NMR (125 MHz, $CDCl_3$) of compound **2g**

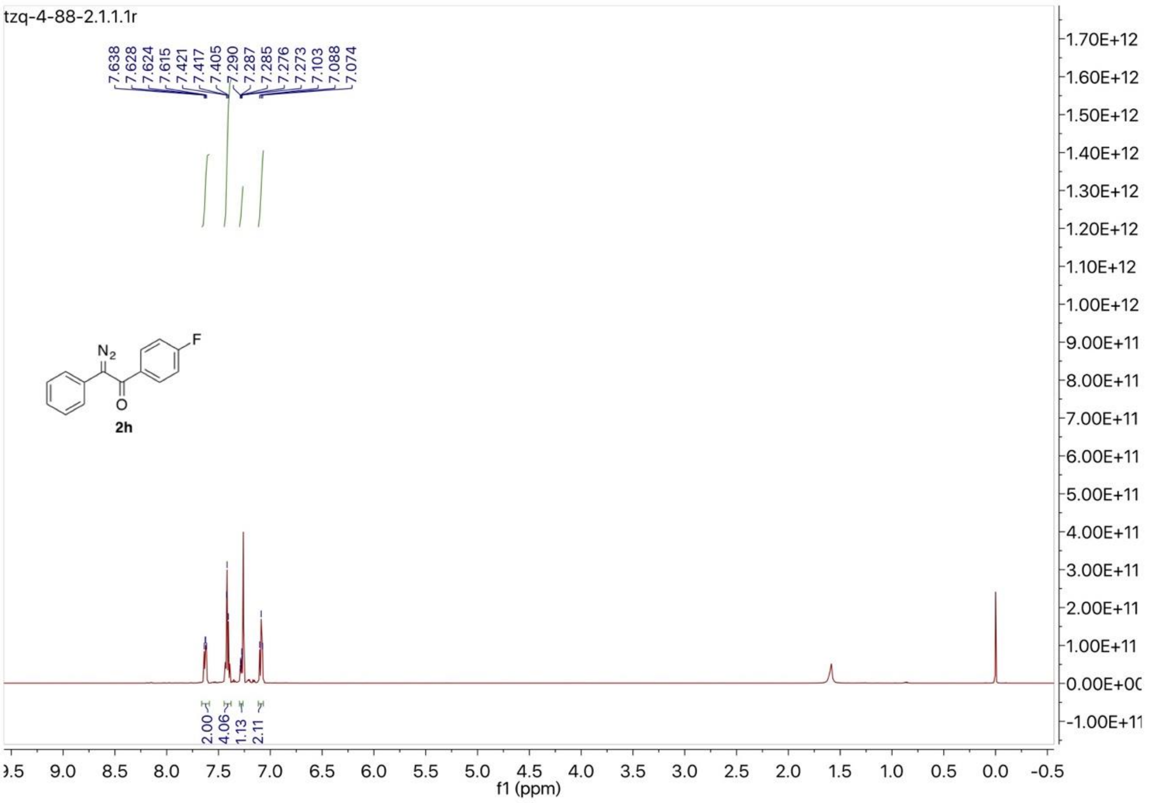

Supplementary Figure 95. ^{1}H NMR (600 MHz, $CDCl_3$) of compound **2h**

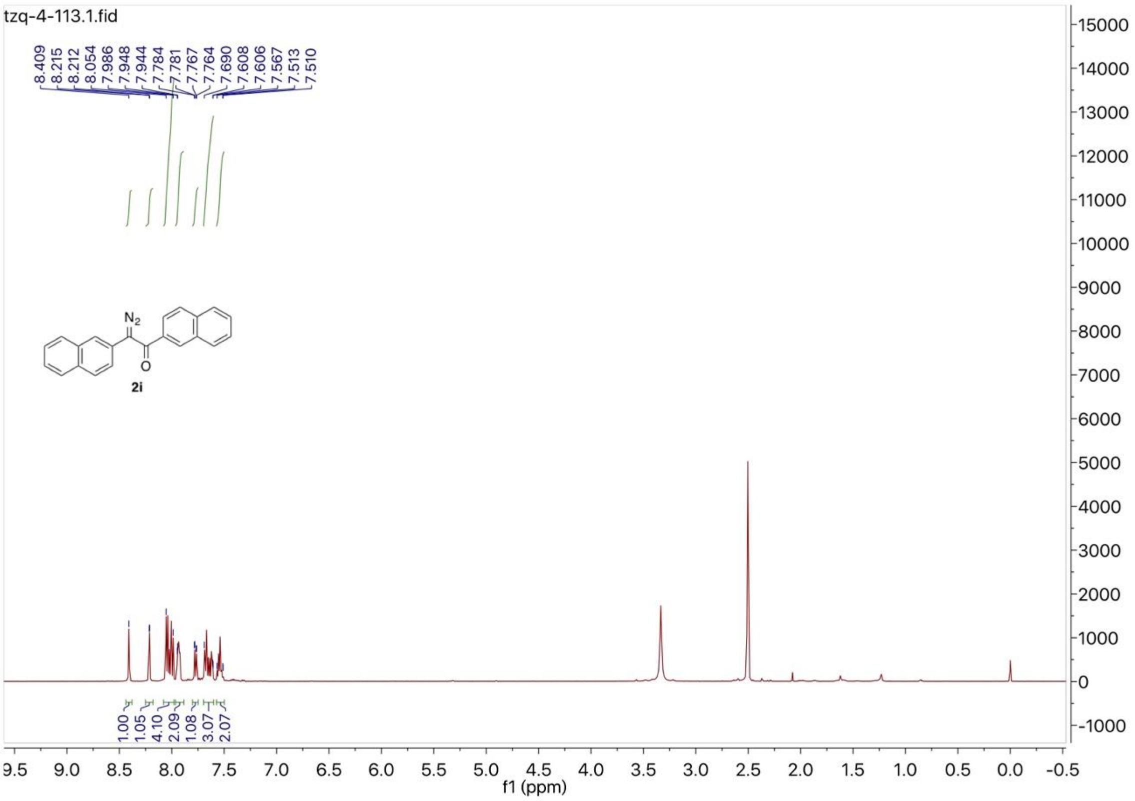

Supplementary Figure 96. ^{1}H NMR (500 MHz, $CDCl_3$) of compound **2i**

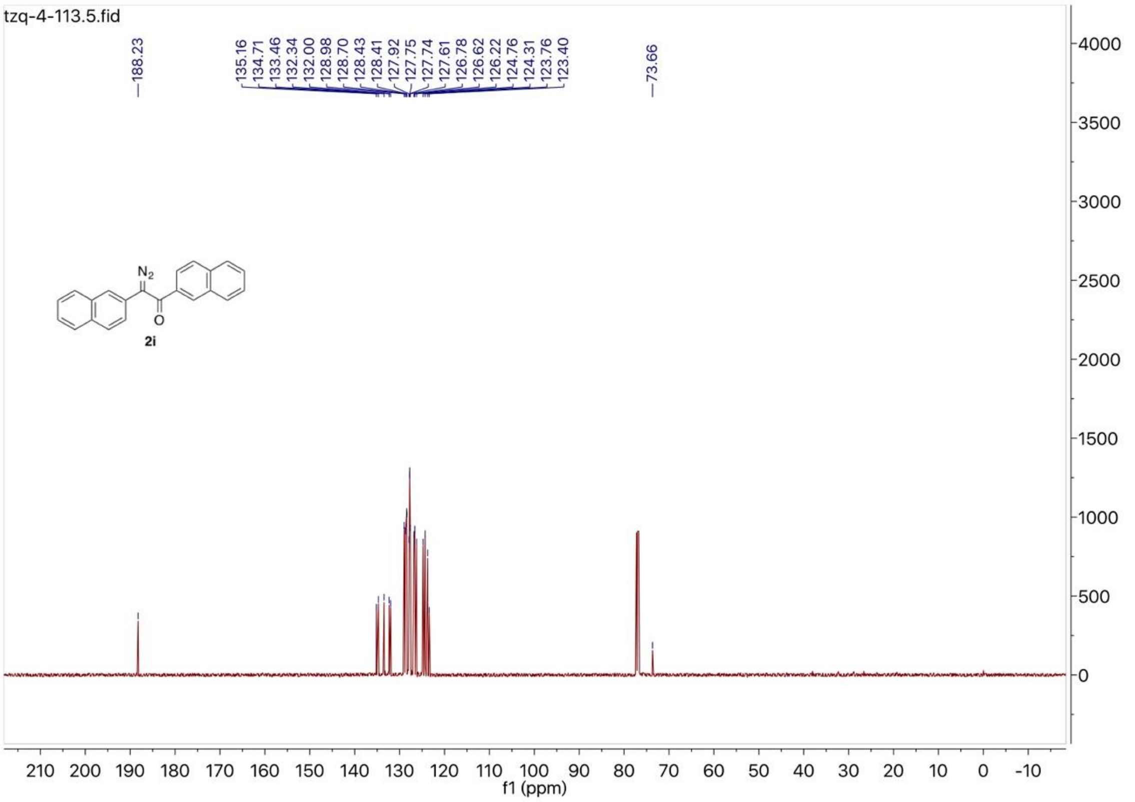

Supplementary Figure 97. ^{13}C NMR (125 MHz, $CDCl_3$) of compound **2i**

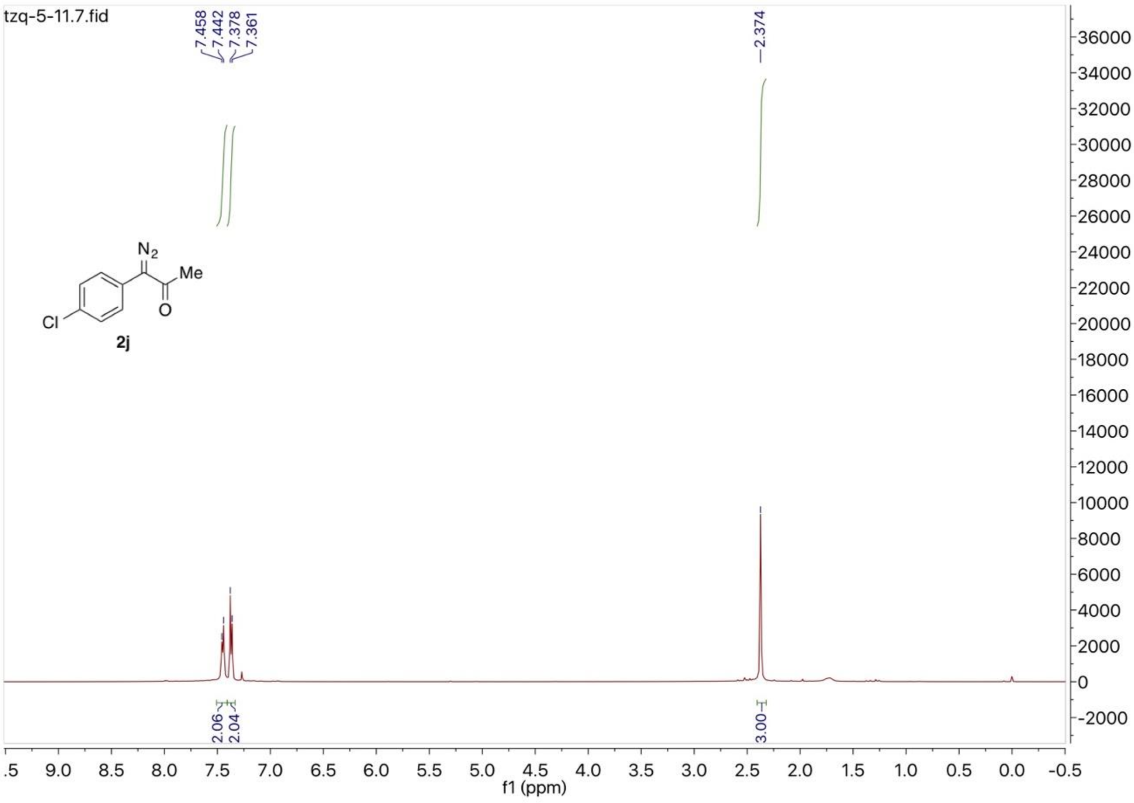

Supplementary Figure 98. ^{1}H NMR (500 MHz, $CDCl_3$) of compound **2j**

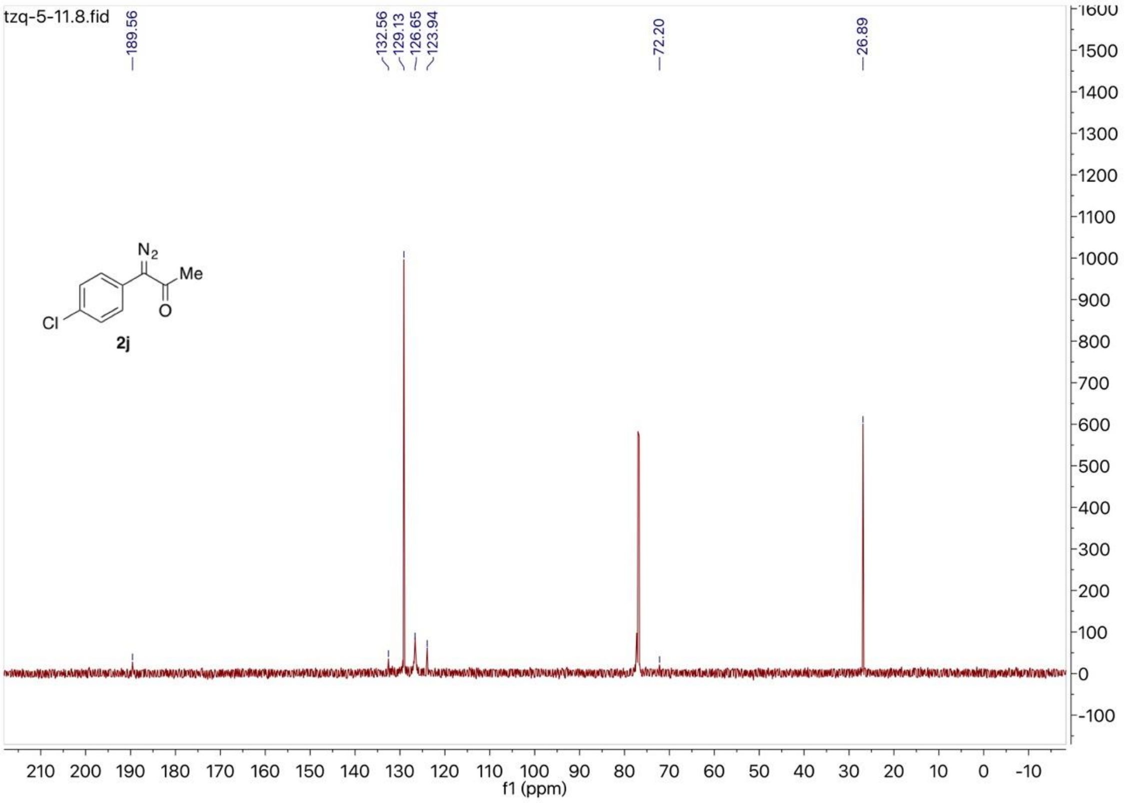

Supplementary Figure 99. ^{13}C NMR (125 MHz, $CDCl_3$) of compound **2j**

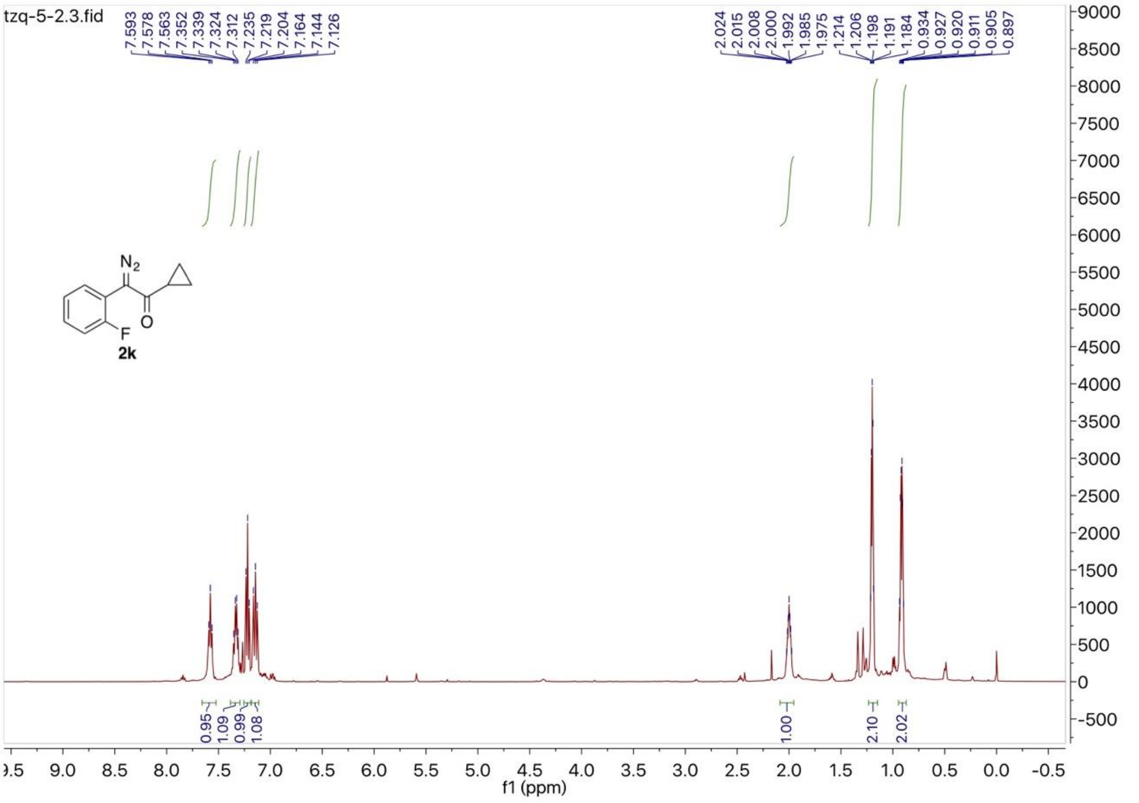

Supplementary Figure 100. ^{1}H NMR (500 MHz, $CDCl_3$) of compound **2k**

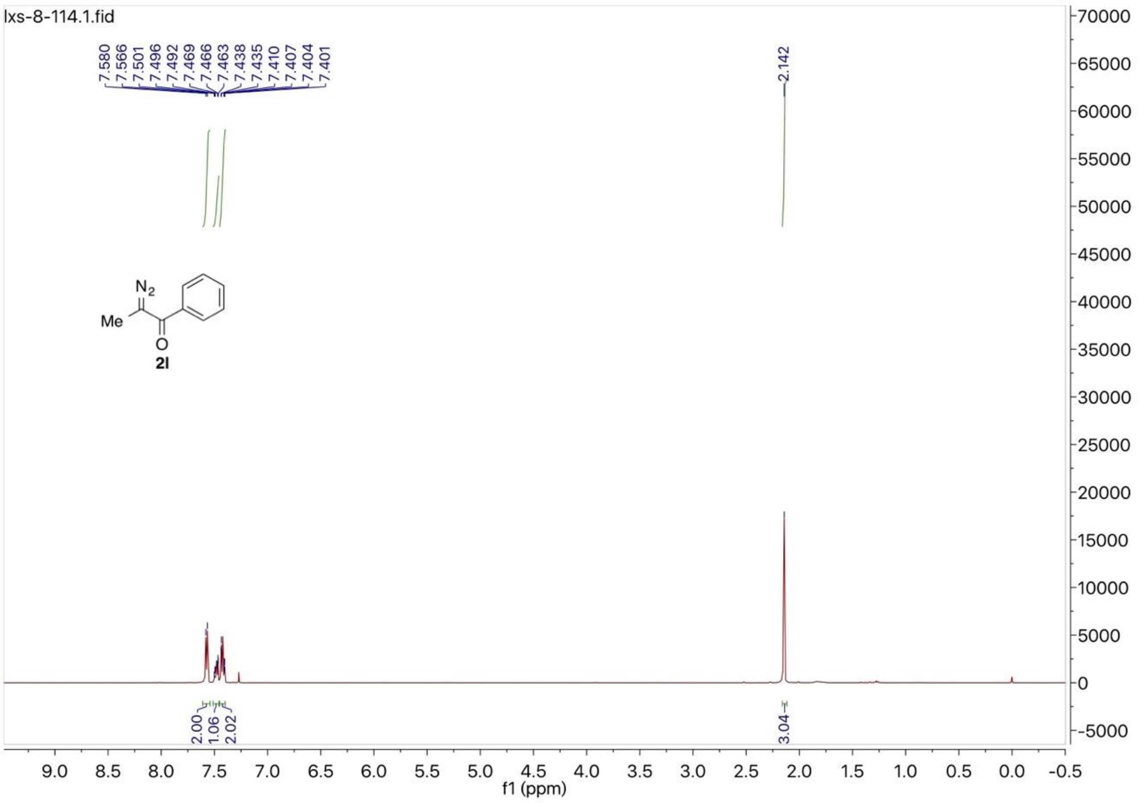

Supplementary Figure 101. ^{1}H NMR (500 MHz, $CDCl_3$) of compound **2l**

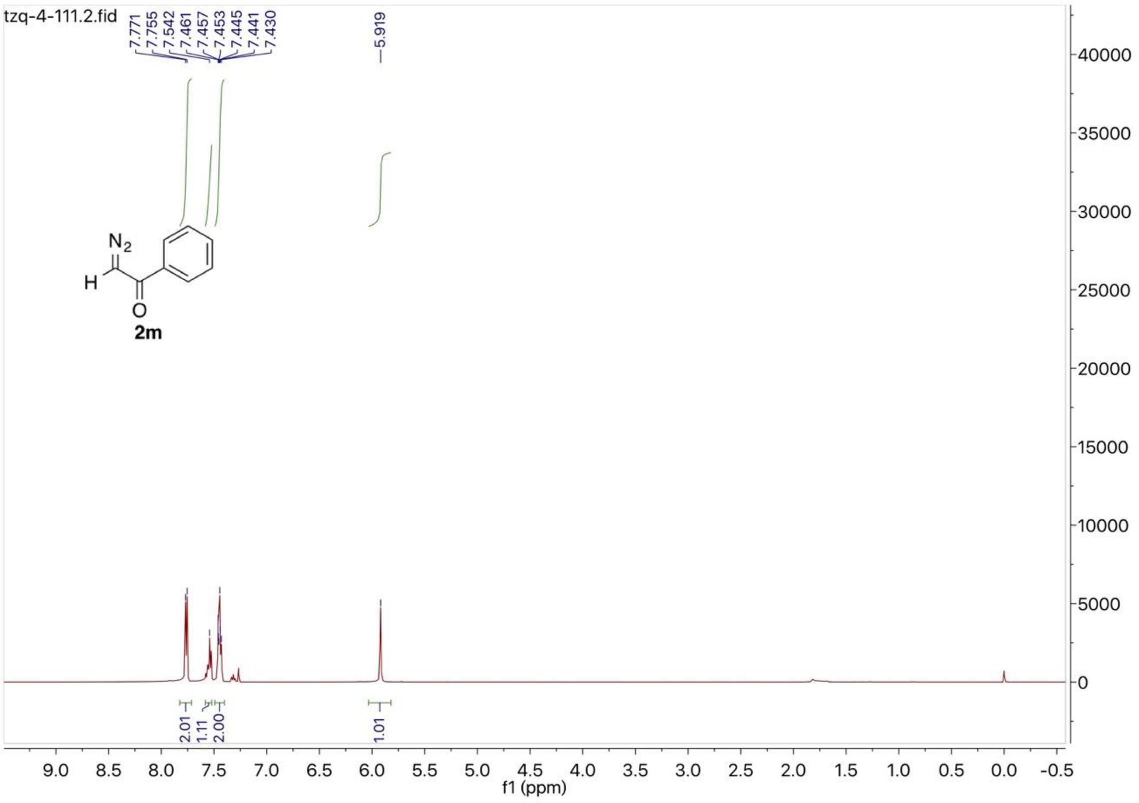

Supplementary Figure 102. ^{1}H NMR (500 MHz, $CDCl_3$) of compound **2m**

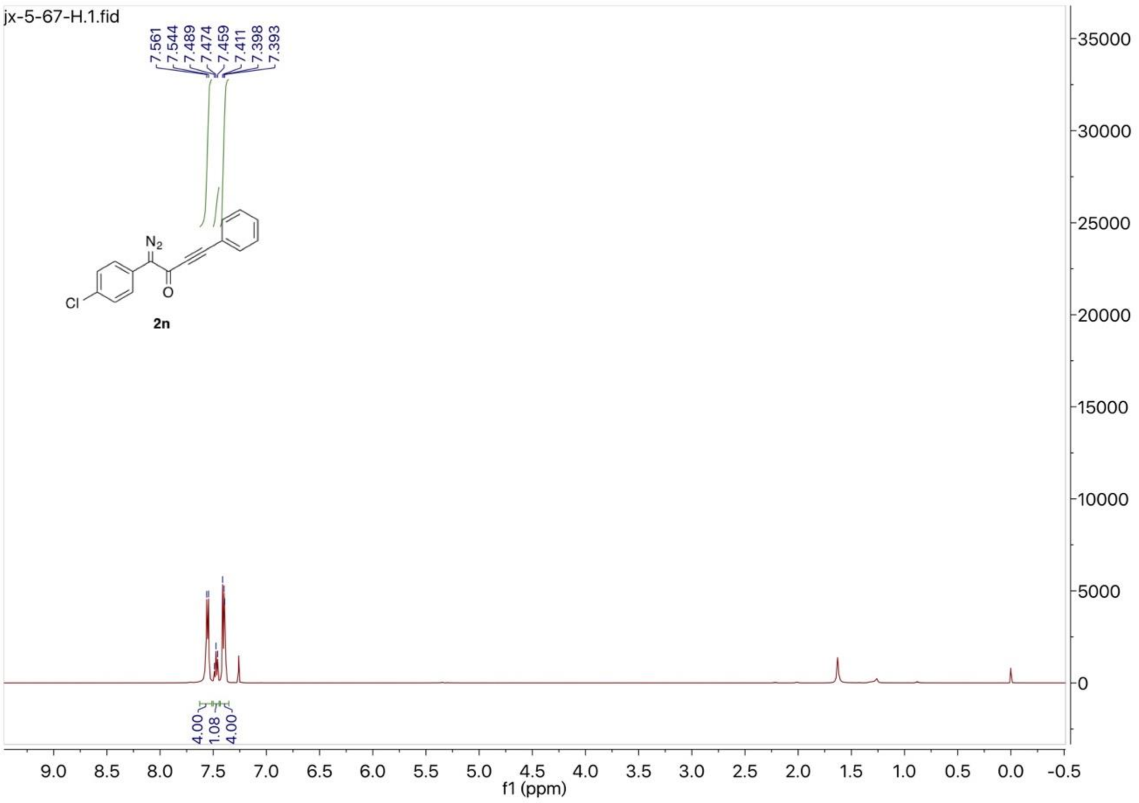

Supplementary Figure 103. ^{1}H NMR (500 MHz, $CDCl_3$) of compound **2n**

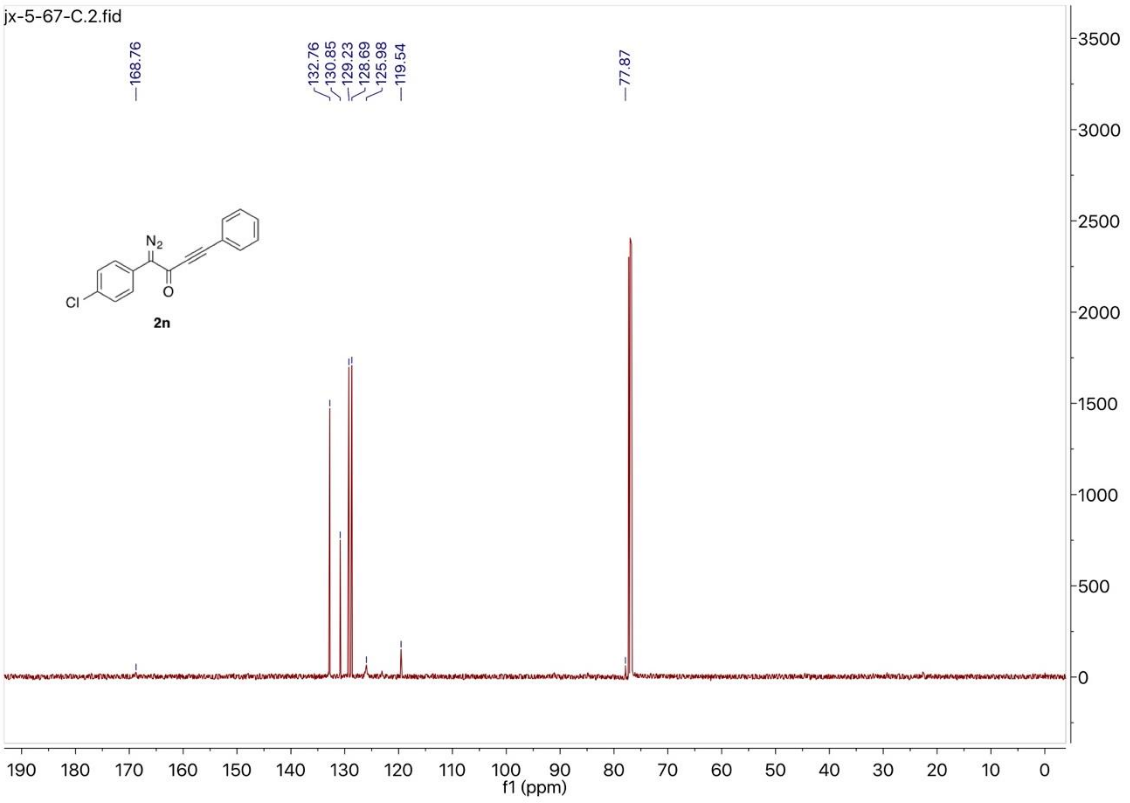

Supplementary Figure 104. ^{13}C NMR (125 MHz, $CDCl_3$) of compound **2n**

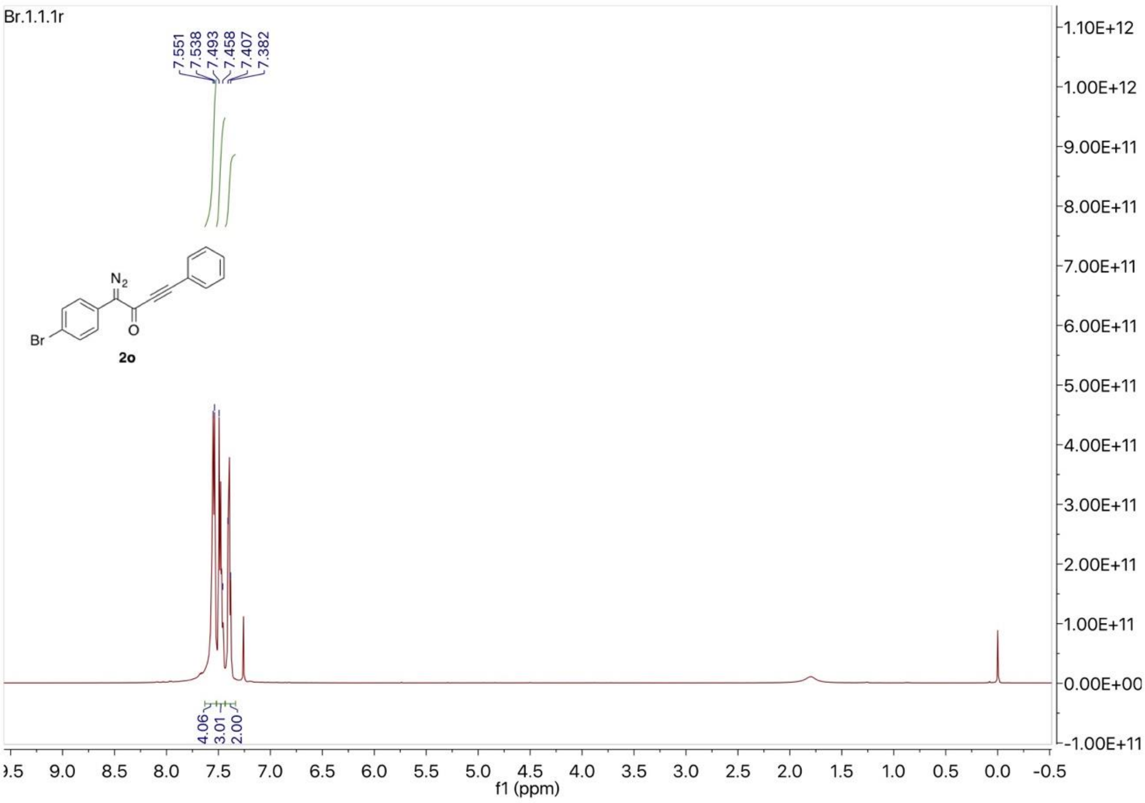

Supplementary Figure 105. ^{1}H NMR (600 MHz, $CDCl_3$) of compound **2o**

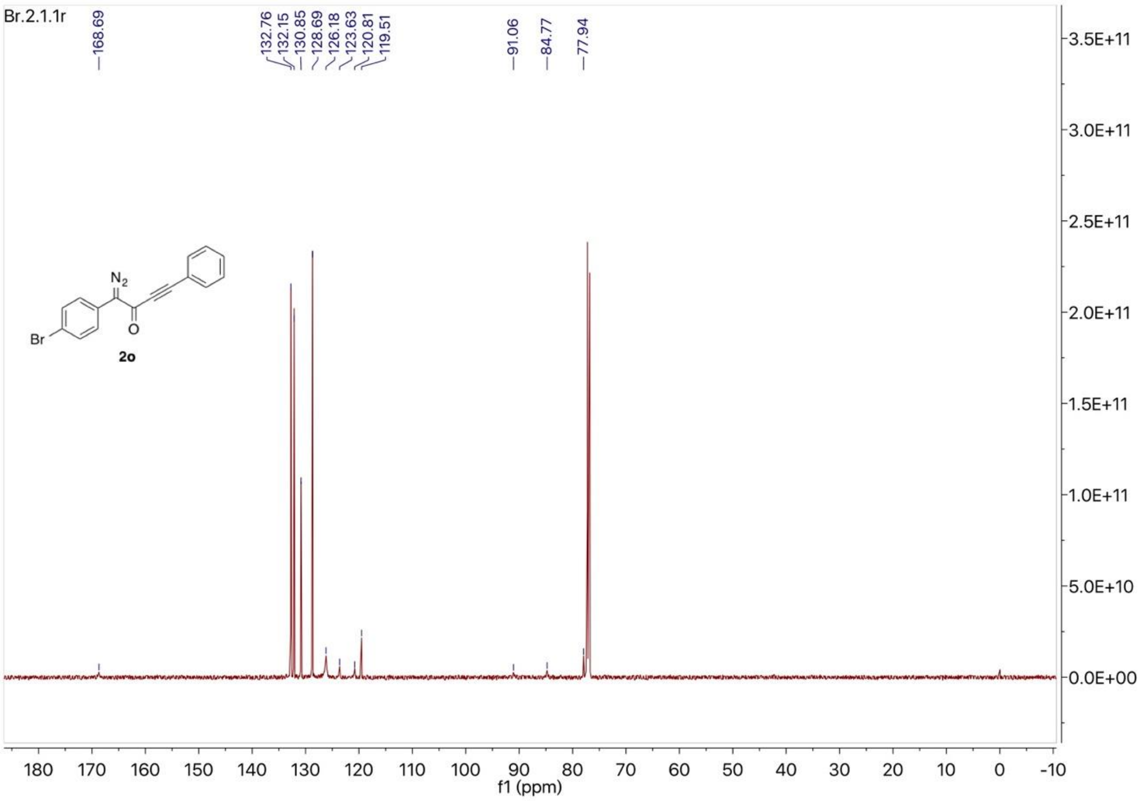

Supplementary Figure 106. ^{13}C NMR (150 MHz, $CDCl_3$) of compound **2o**

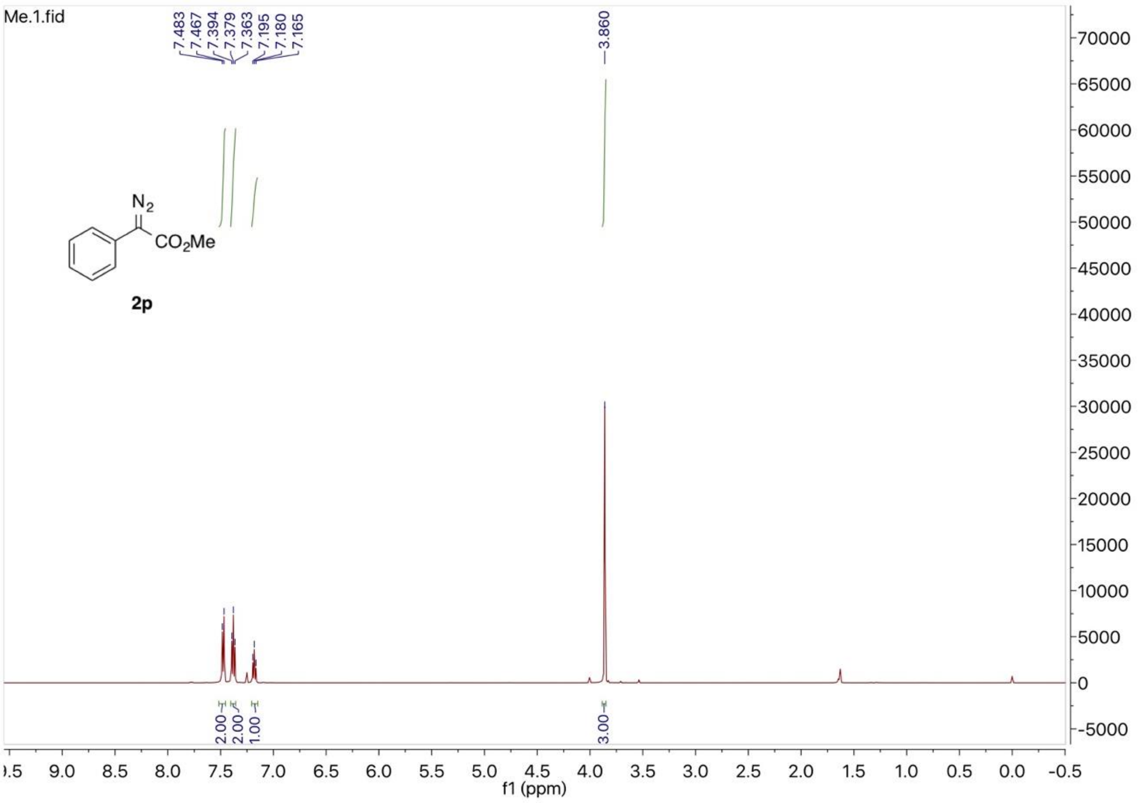

Supplementary Figure 107. ^{1}H NMR (500 MHz, $CDCl_3$) of compound **2p**

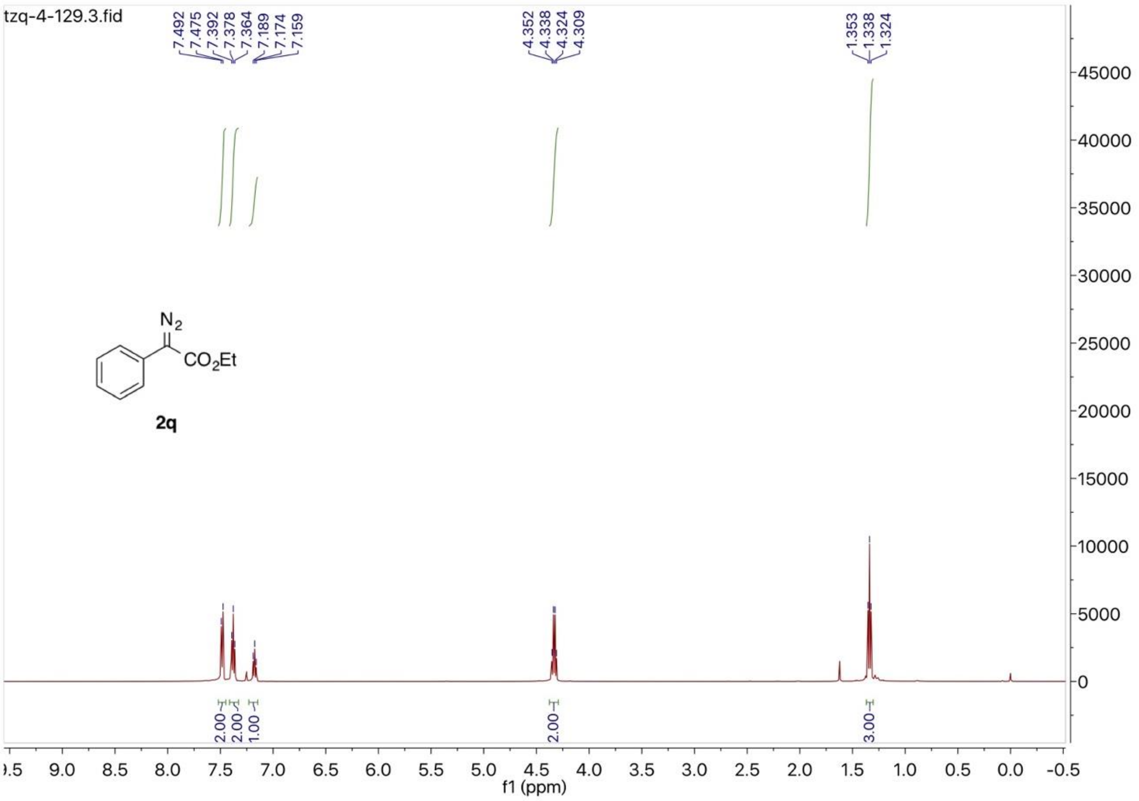

Supplementary Figure 108. ^{1}H NMR (500 MHz, $CDCl_3$) of compound **2q**

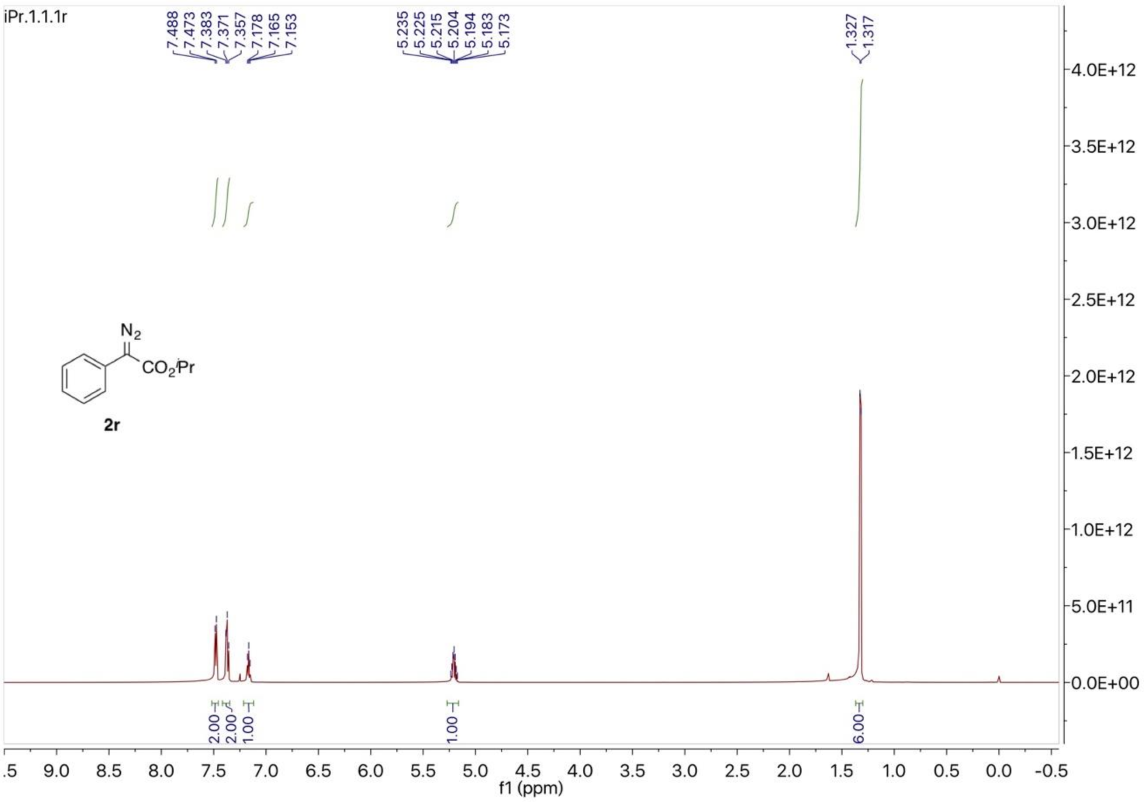

Supplementary Figure 109. ^{1}H NMR (600 MHz, $CDCl_3$) of compound **2r**

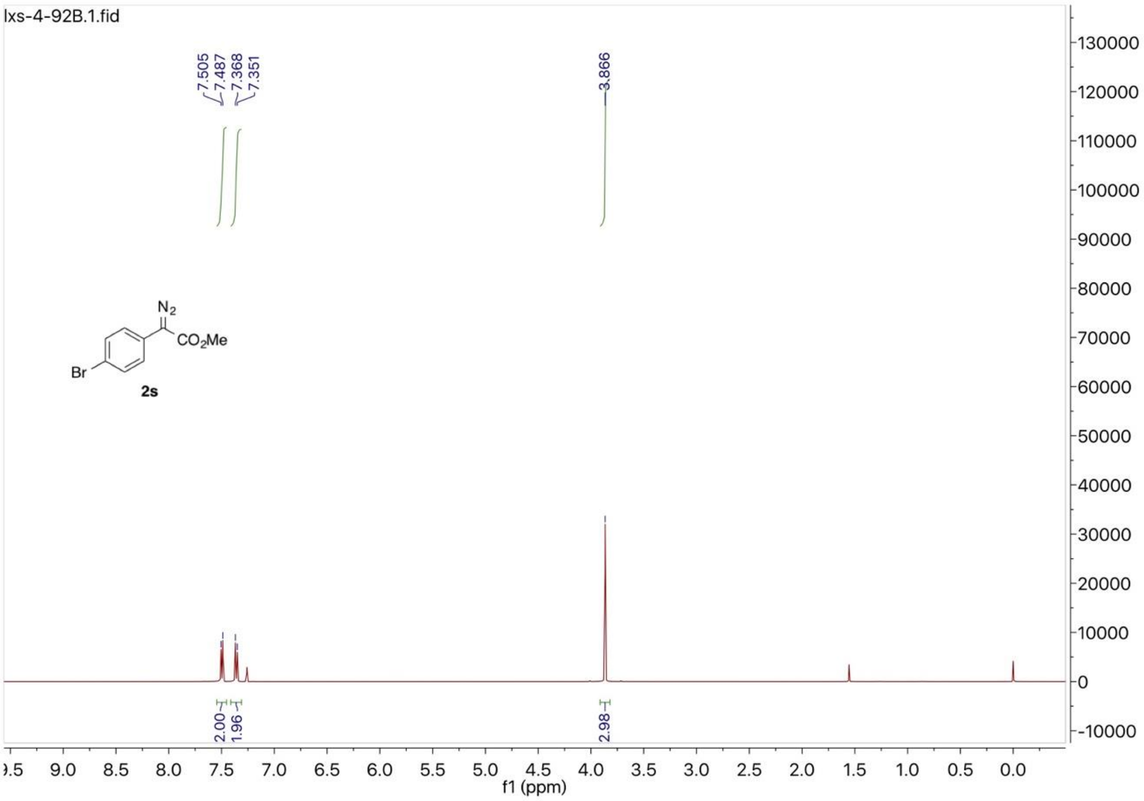

Supplementary Figure 110. ^{1}H NMR (500 MHz, $CDCl_3$) of compound **2s**

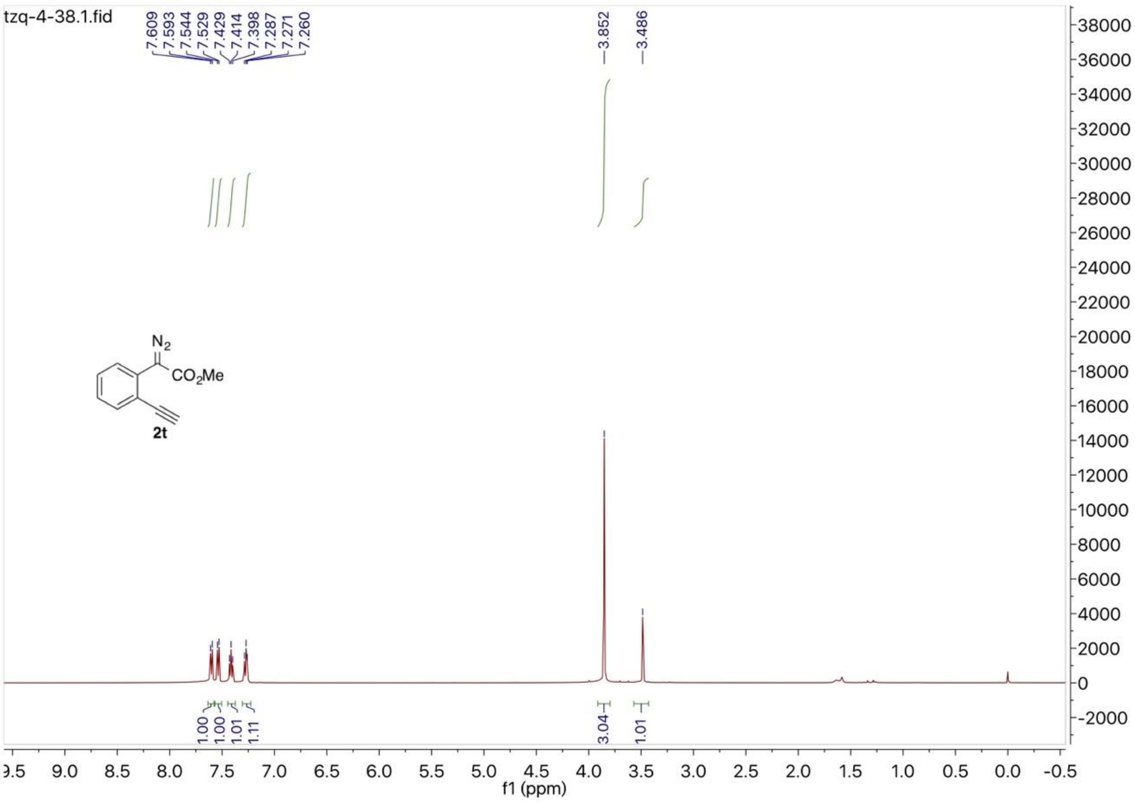

Supplementary Figure 111. ^{1}H NMR (500 MHz, $CDCl_3$) of compound **2t**

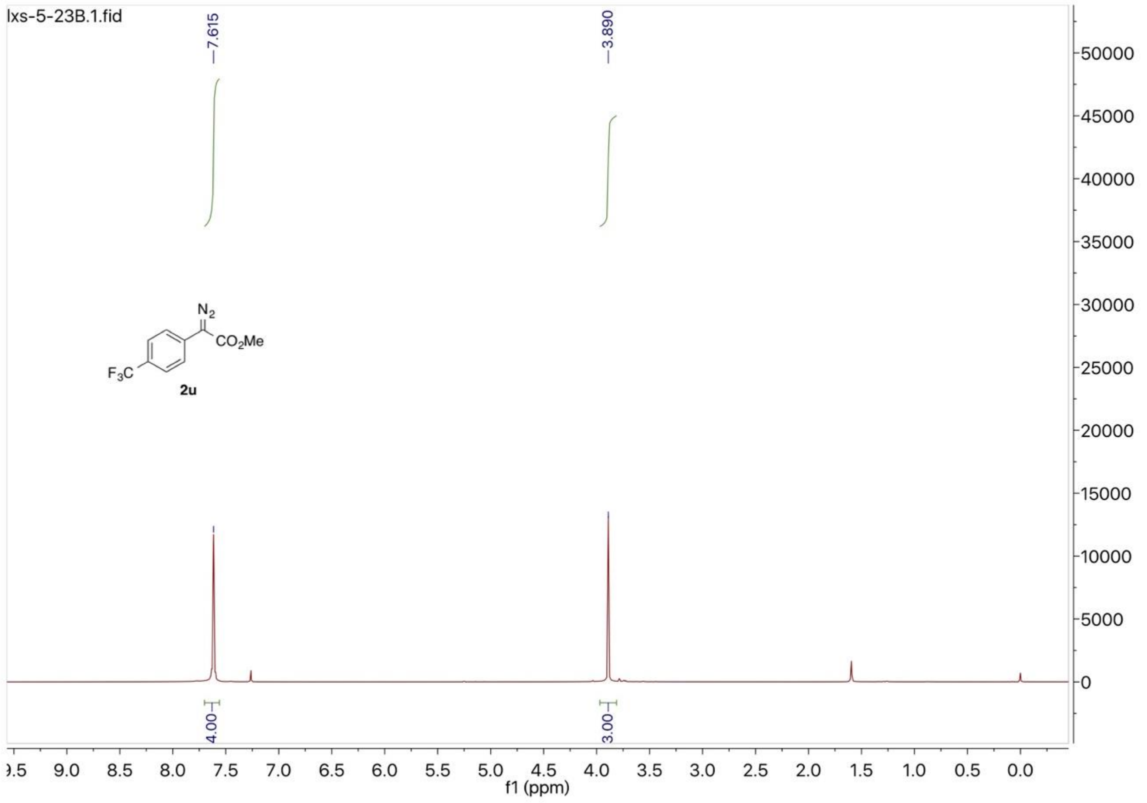

Supplementary Figure 112. ^{1}H NMR (500 MHz, $CDCl_3$) of compound **2u**

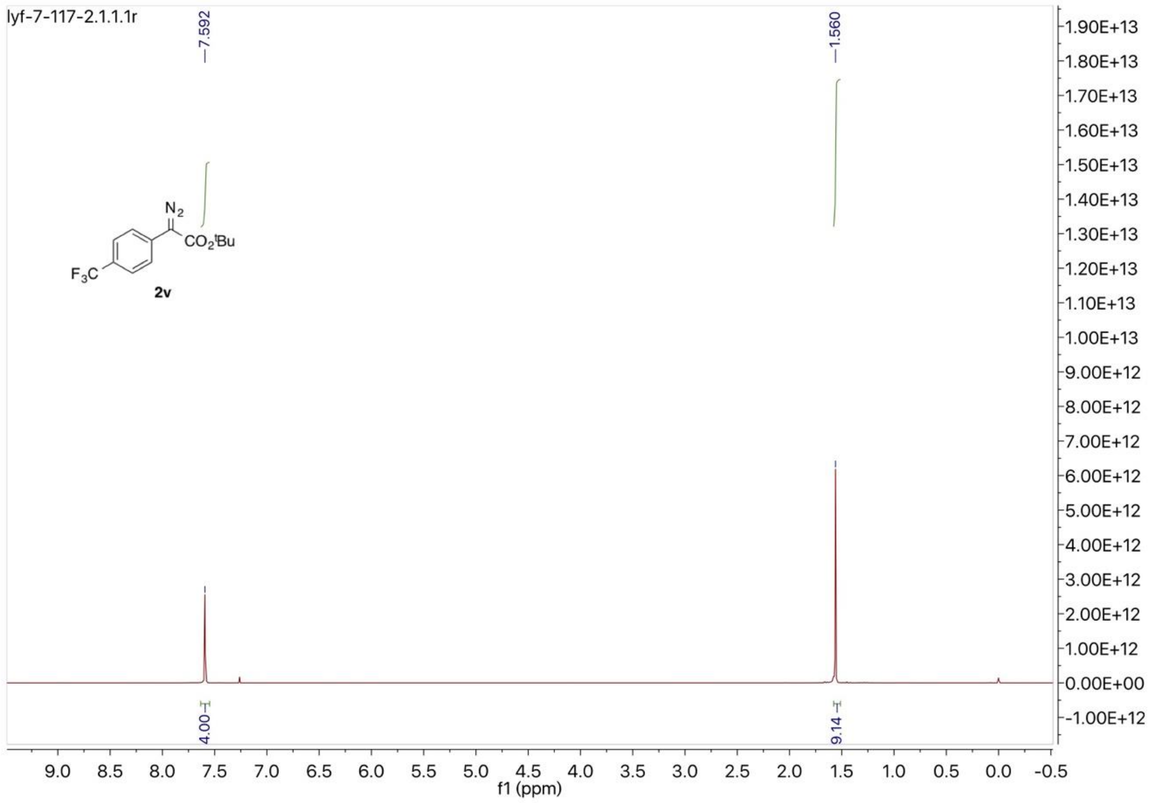

Supplementary Figure 113. ^{1}H NMR (600 MHz, $CDCl_3$) of compound **2v**

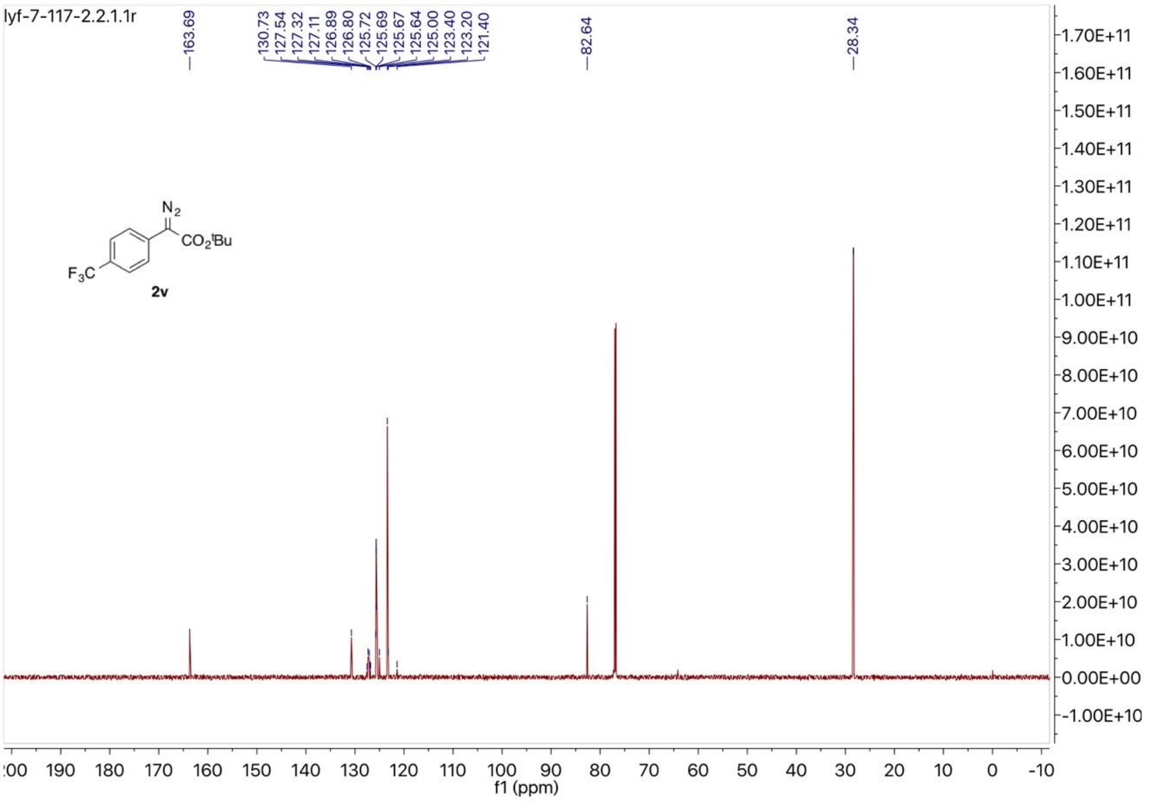

Supplementary Figure 114. ^{13}C NMR (150 MHz, $CDCl_3$) of compound **2v**

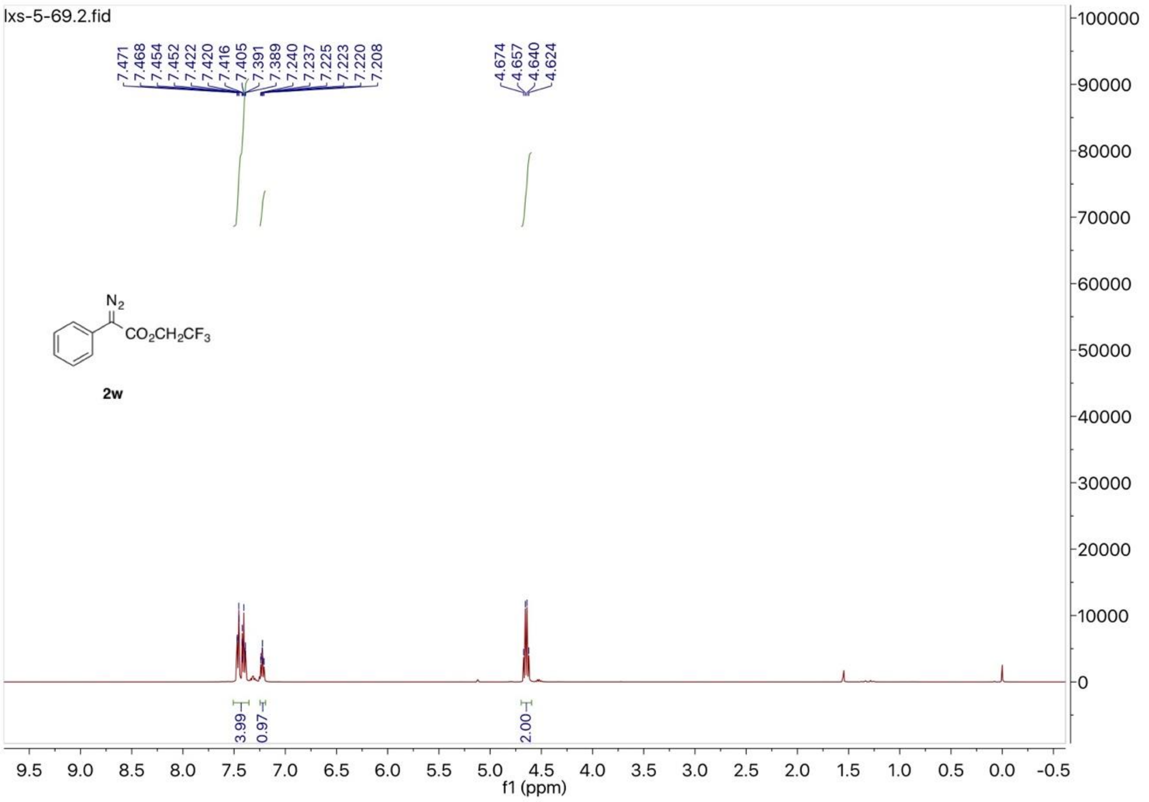

Supplementary Figure 115. ^{1}H NMR (500 MHz, $CDCl_3$) of compound **2w**

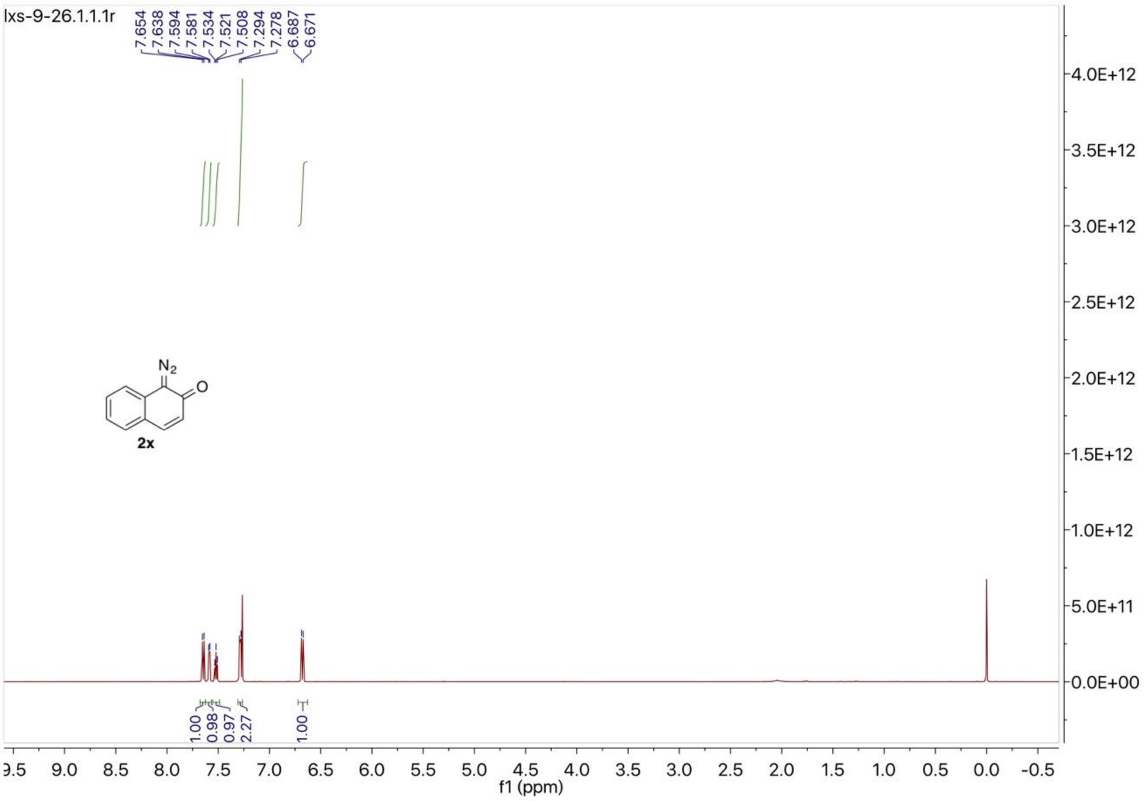

Supplementary Figure 116. ^{1}H NMR (600 MHz, $CDCl_3$) of compound **2x**

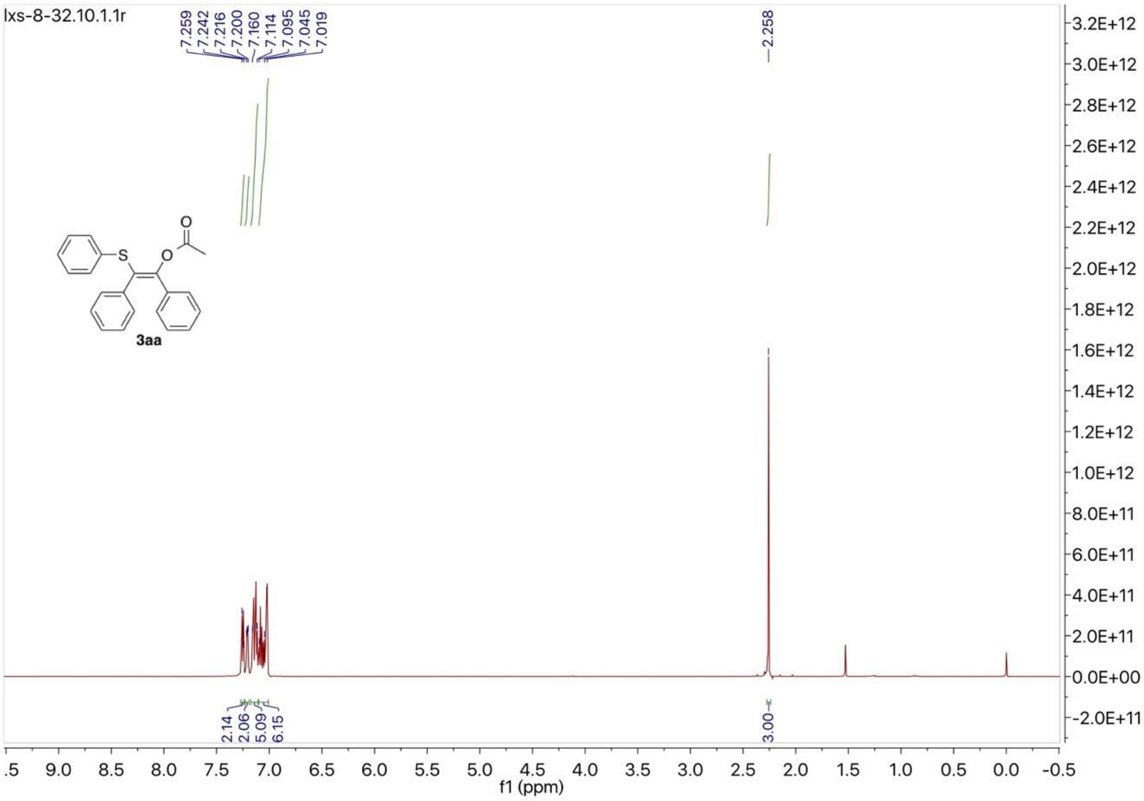

Supplementary Figure 117. ^{1}H NMR (600 MHz, $CDCl_3$) of compound **3aa**

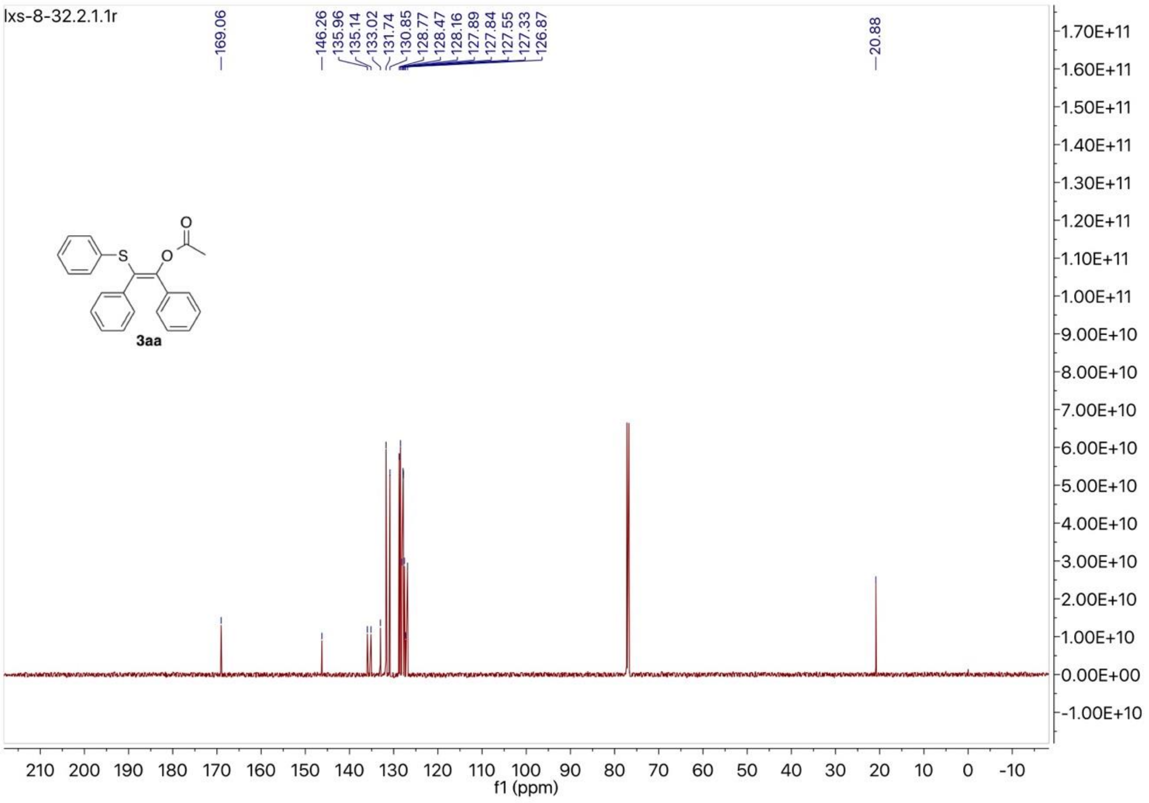

Supplementary Figure 118. ^{13}C NMR (150 MHz, $CDCl_3$) of compound **3aa**

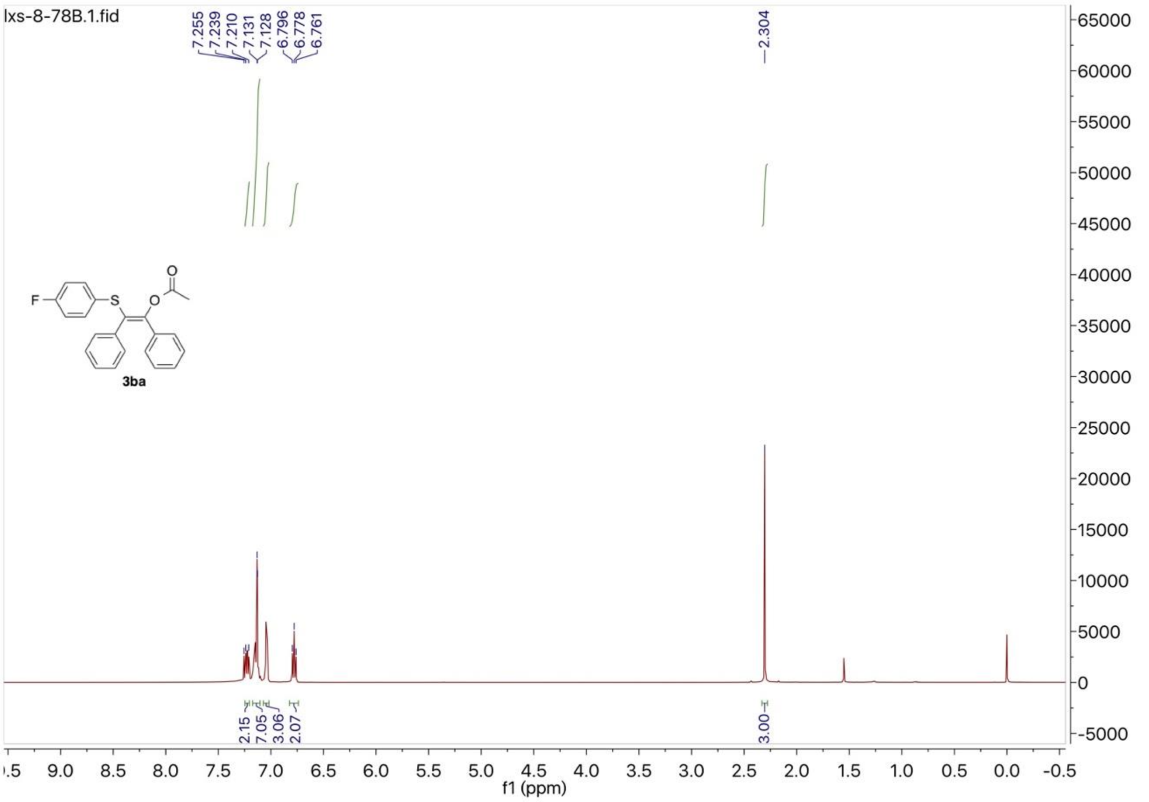

Supplementary Figure 119. ^{1}H NMR (500 MHz, $CDCl_3$) of compound **3ba**

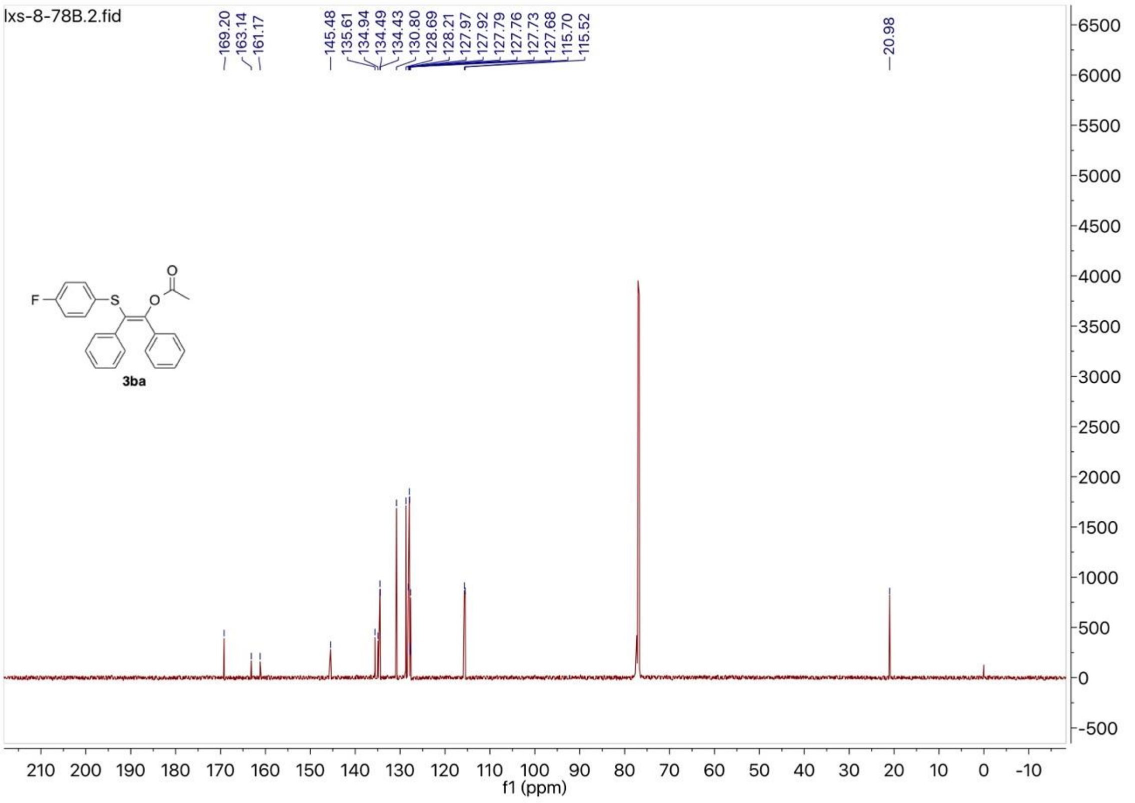

Supplementary Figure 120. ^{13}C NMR (125 MHz, $CDCl_3$) of compound **3ba**

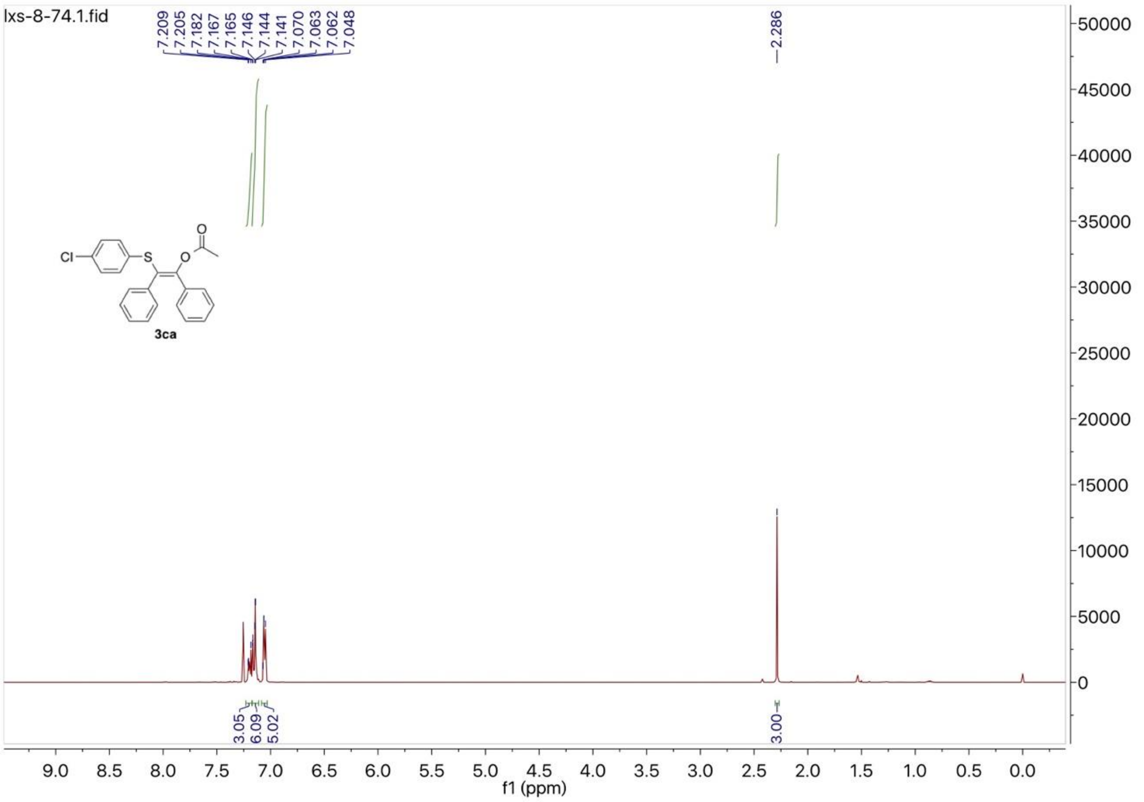

Supplementary Figure 121. ^{1}H NMR (500 MHz, $CDCl_3$) of compound **3ca**

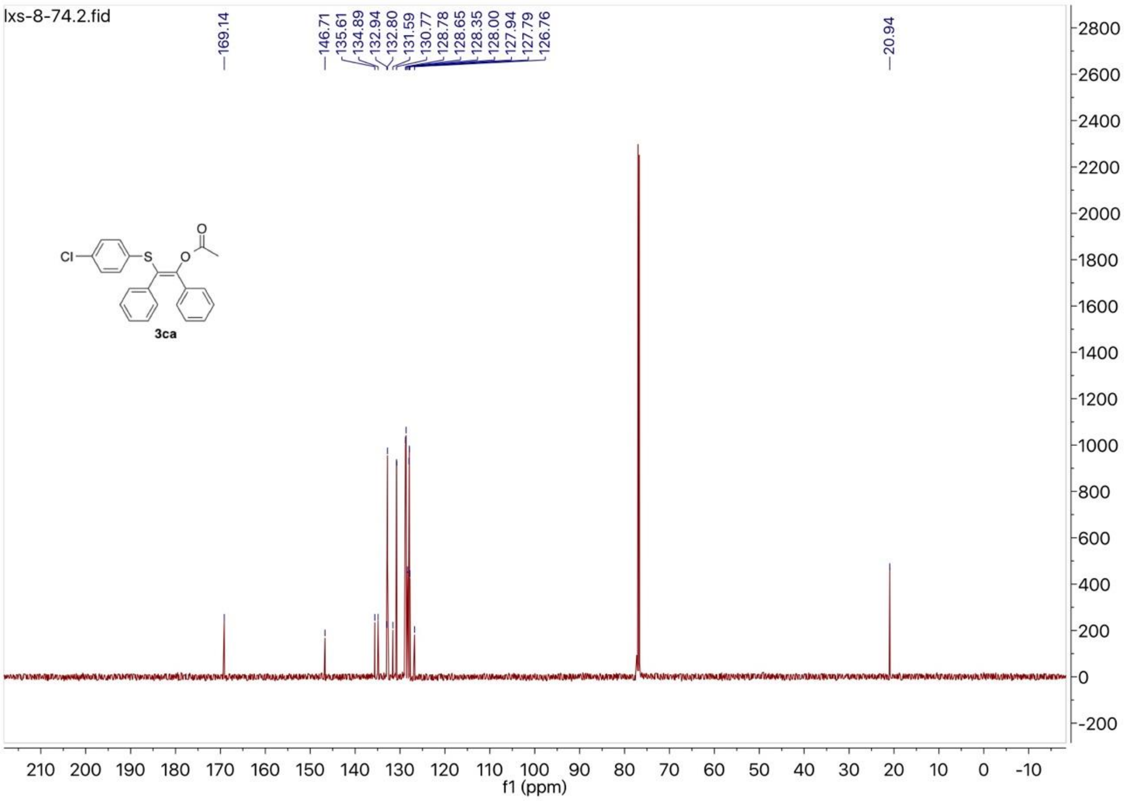

Supplementary Figure 122. ^{13}C NMR (125 MHz, $CDCl_3$) of compound **3ca**

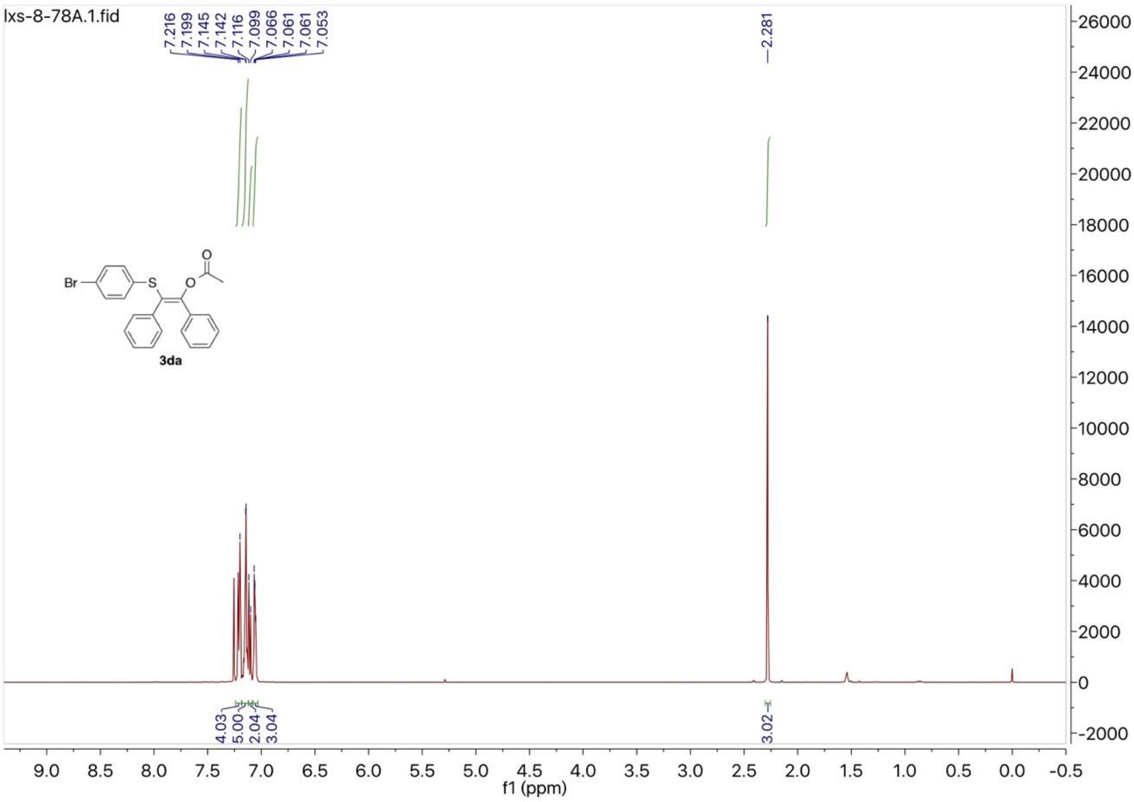

Supplementary Figure 123. ^{1}H NMR (500 MHz, $CDCl_3$) of compound **3da**

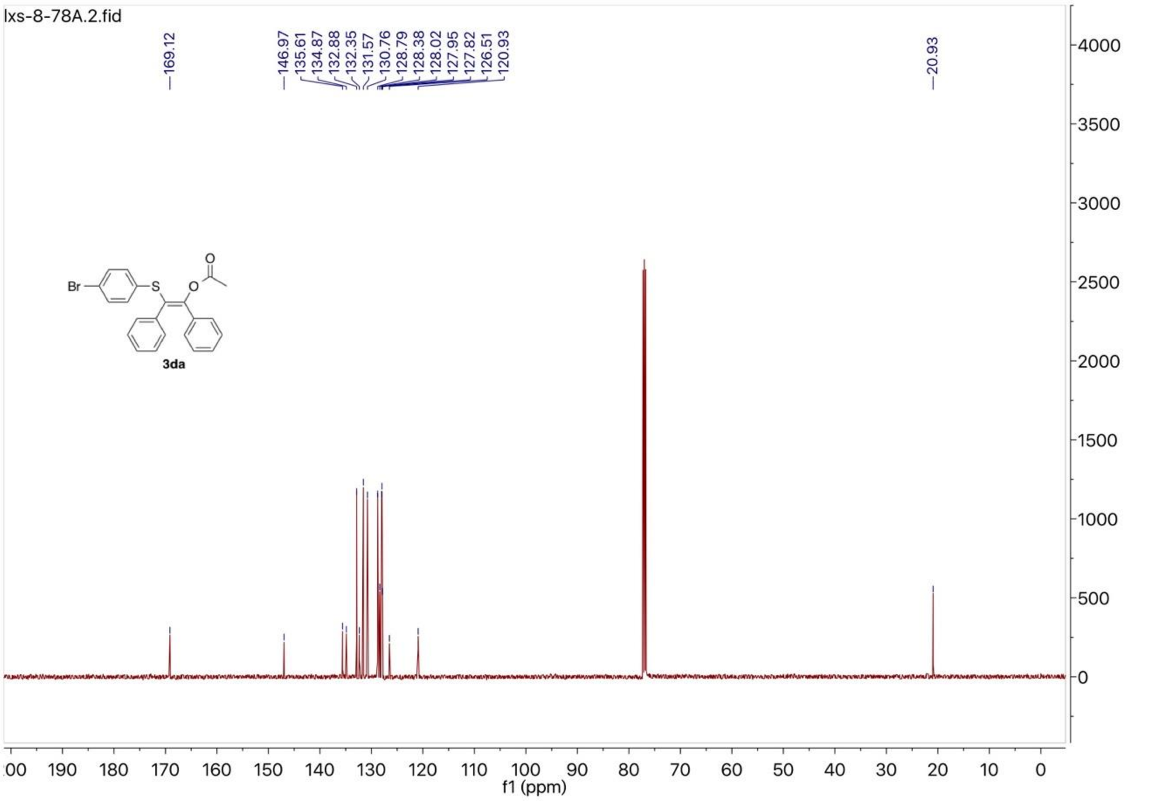

Supplementary Figure 124. ^{13}C NMR (125 MHz, $CDCl_3$) of compound **3da**

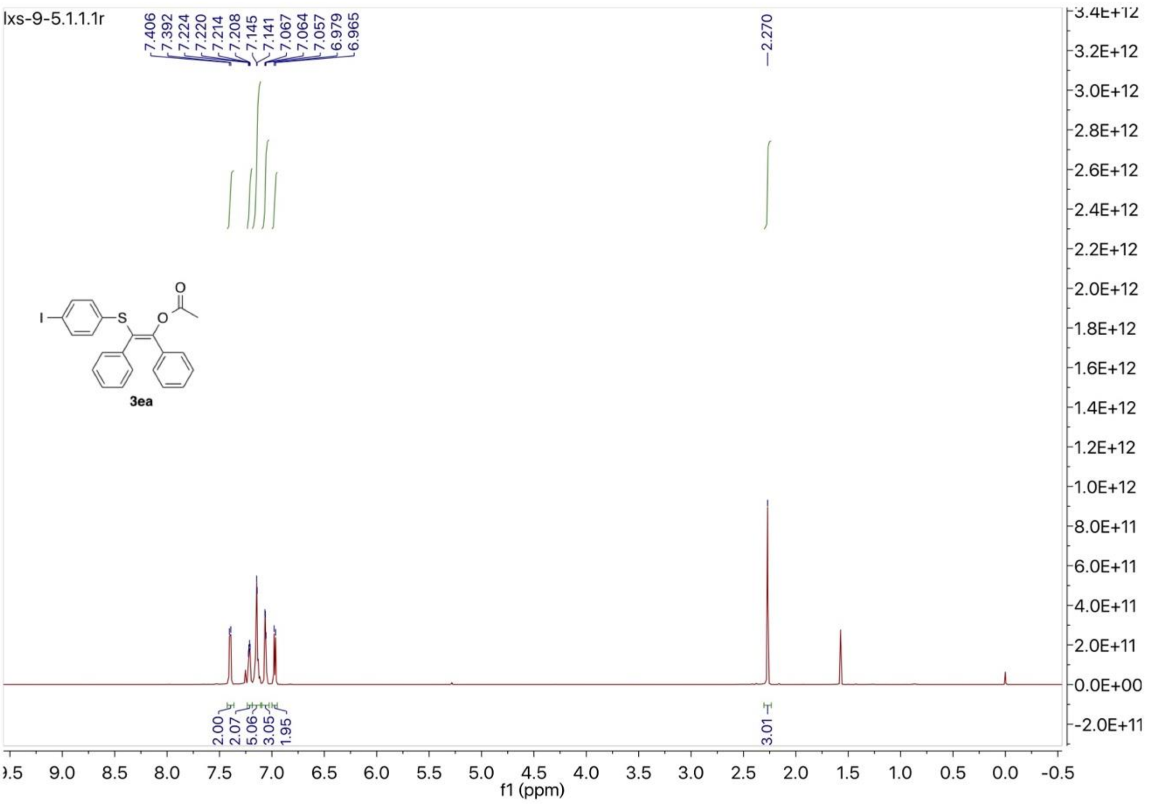

Supplementary Figure 125. ^{1}H NMR (600 MHz, $CDCl_3$) of compound **3ea**

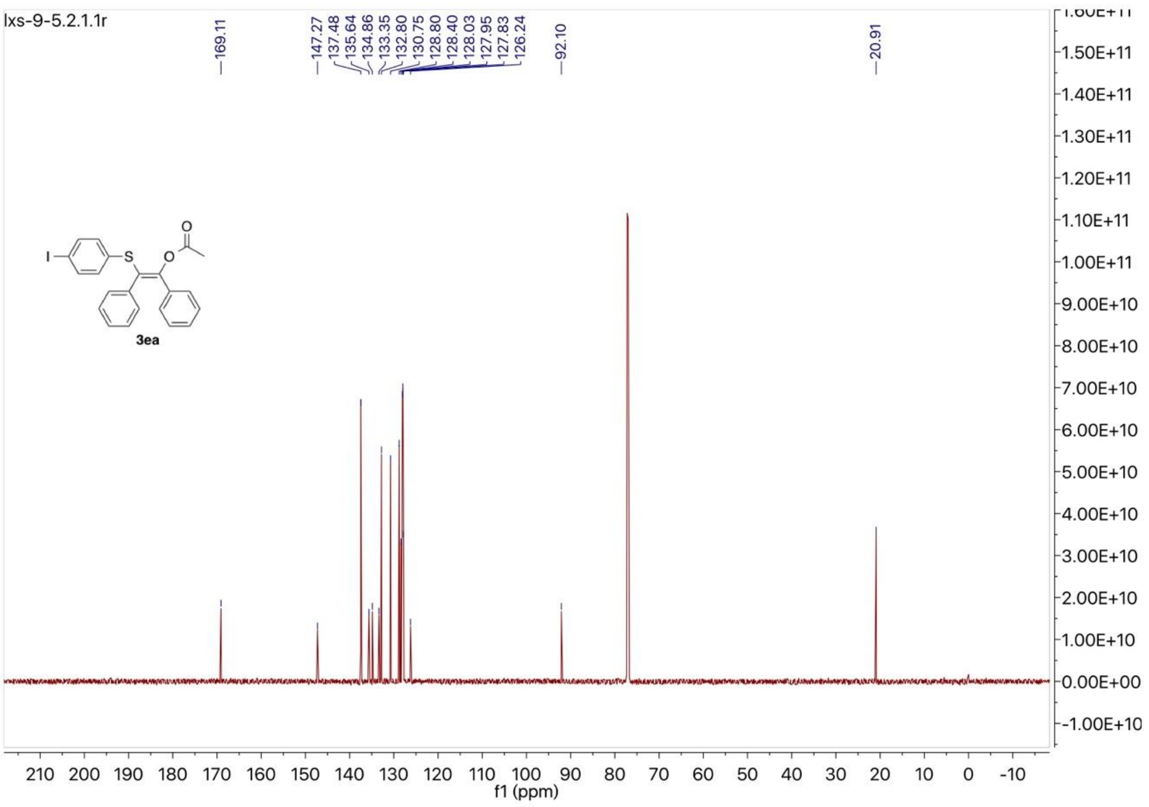

Supplementary Figure 126. ^{13}C NMR (150 MHz, $CDCl_3$) of compound **3ea**

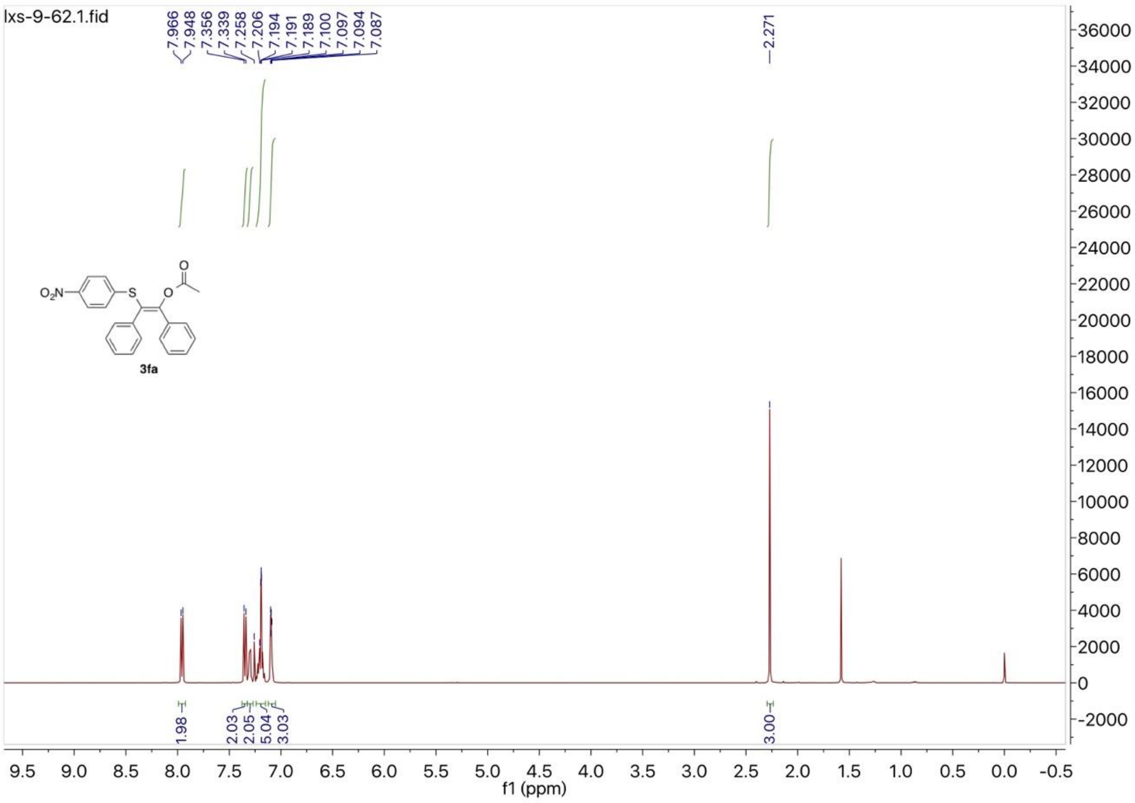

Supplementary Figure 127. ^{1}H NMR (500 MHz, $CDCl_3$) of compound **3fa**

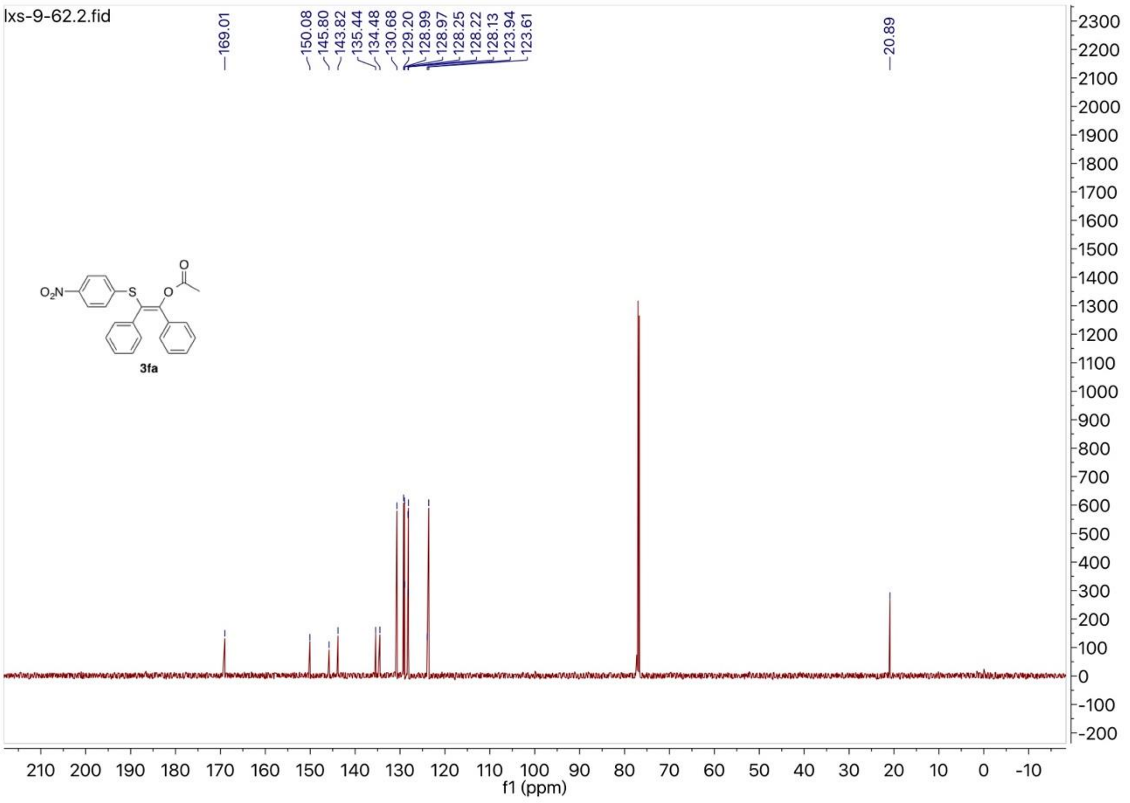

Supplementary Figure 128. ^{13}C NMR (125 MHz, $CDCl_3$) of compound **3fa**

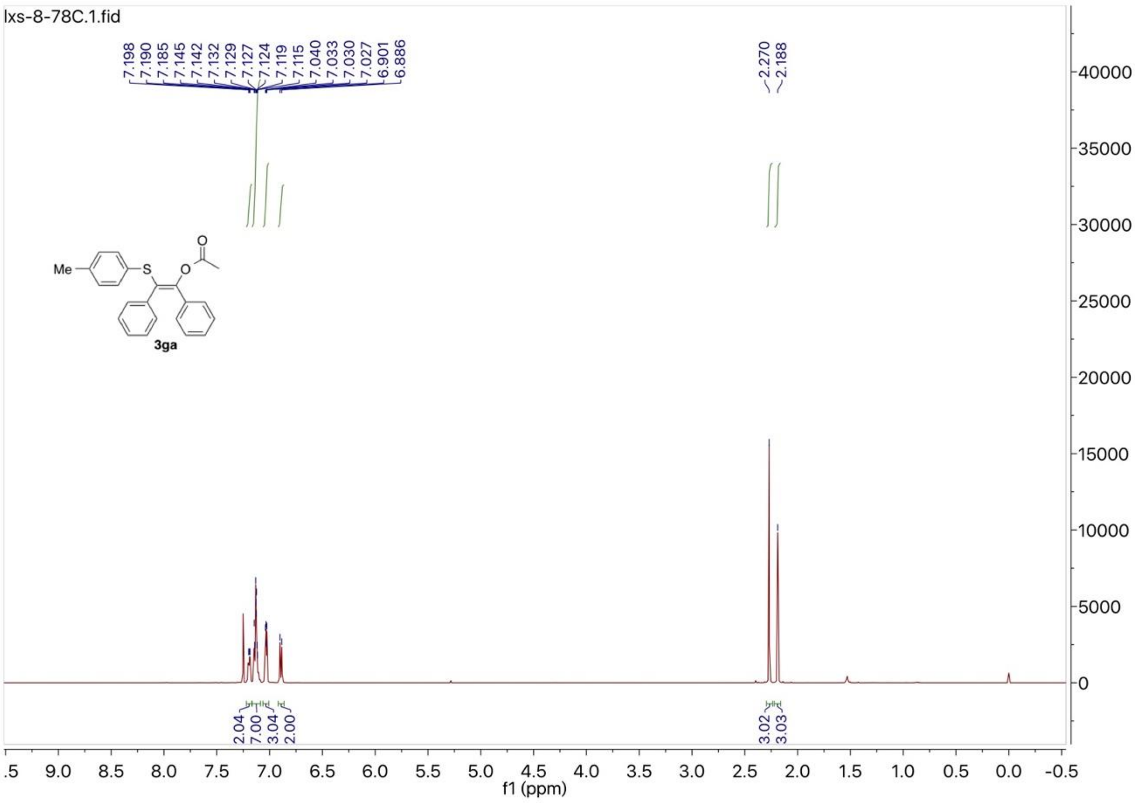

Supplementary Figure 129. ^{1}H NMR (500 MHz, $CDCl_3$) of compound **3ga**

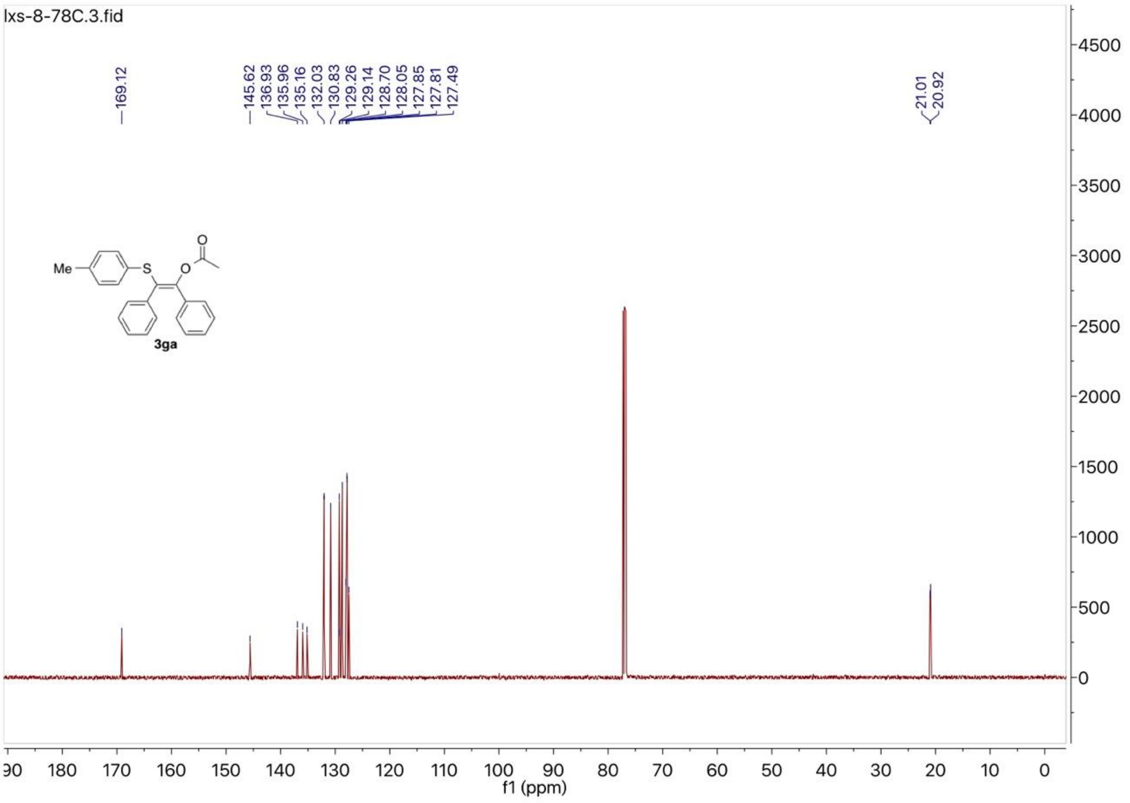

Supplementary Figure 130. ^{13}C NMR (125 MHz, $CDCl_3$) of compound **3ga**

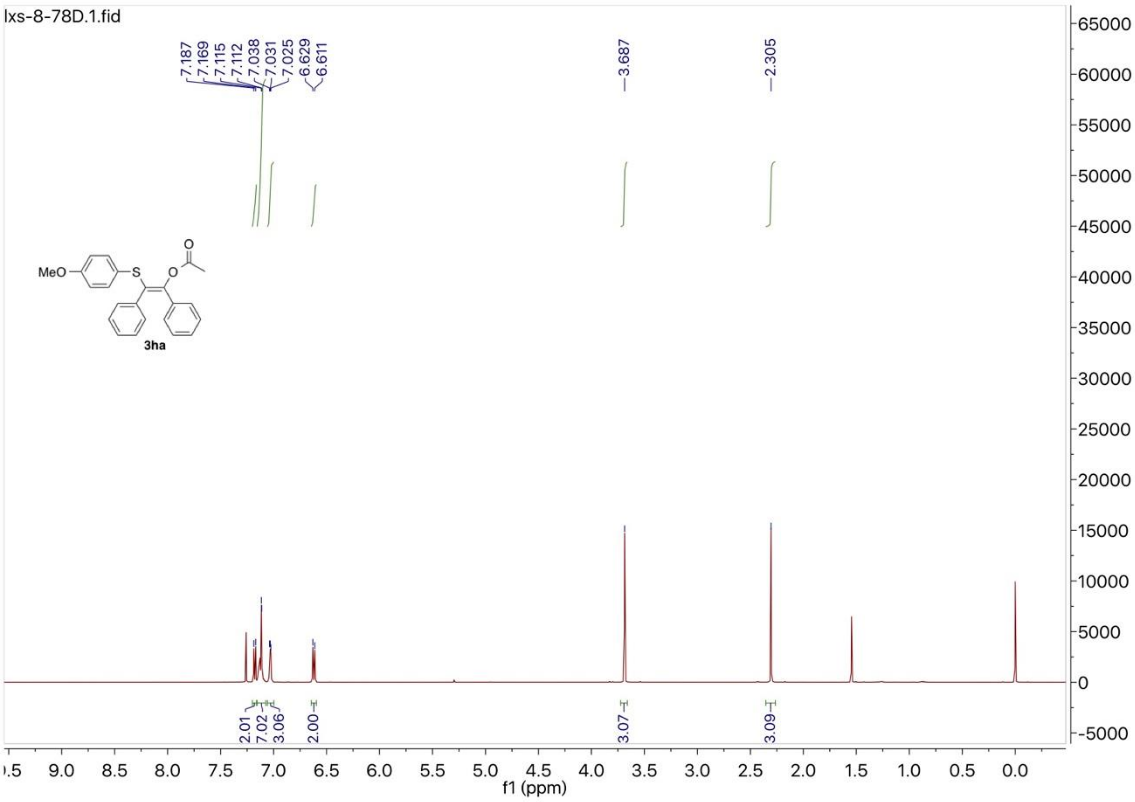

Supplementary Figure 131. ^{1}H NMR (500 MHz, $CDCl_3$) of compound **3ha**

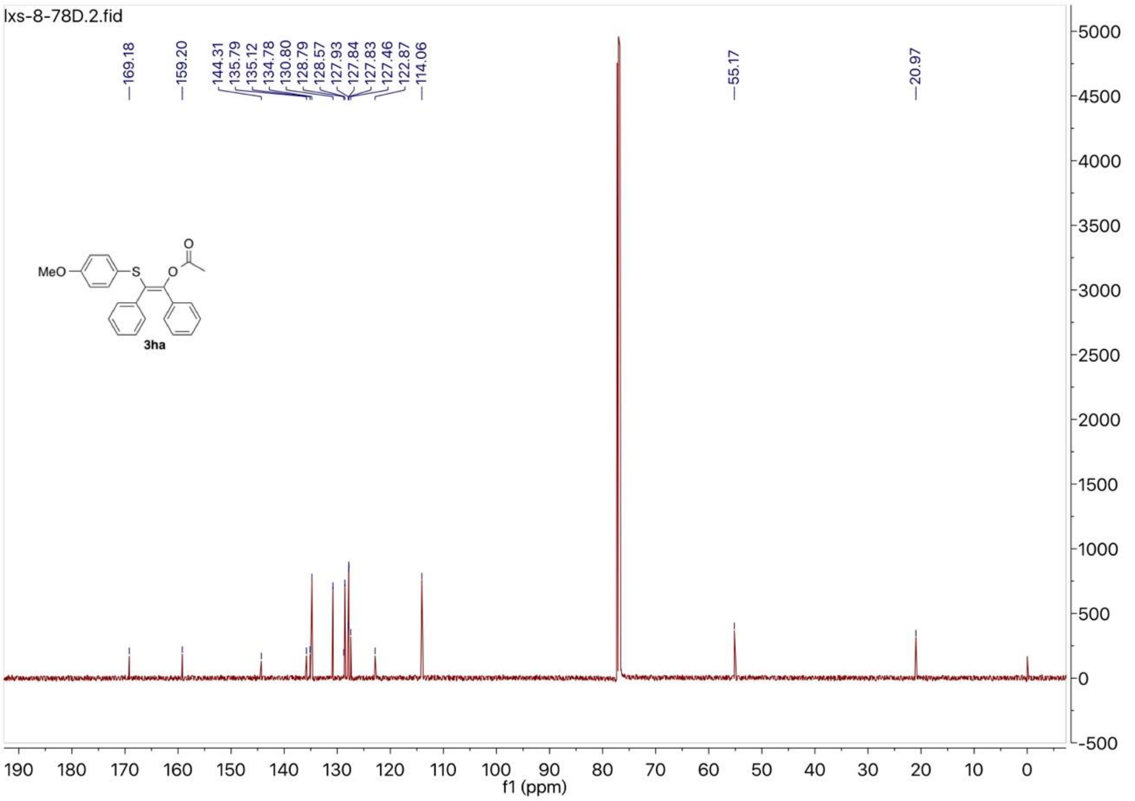

Supplementary Figure 132. ^{13}C NMR (125 MHz, $CDCl_3$) of compound **3ha**

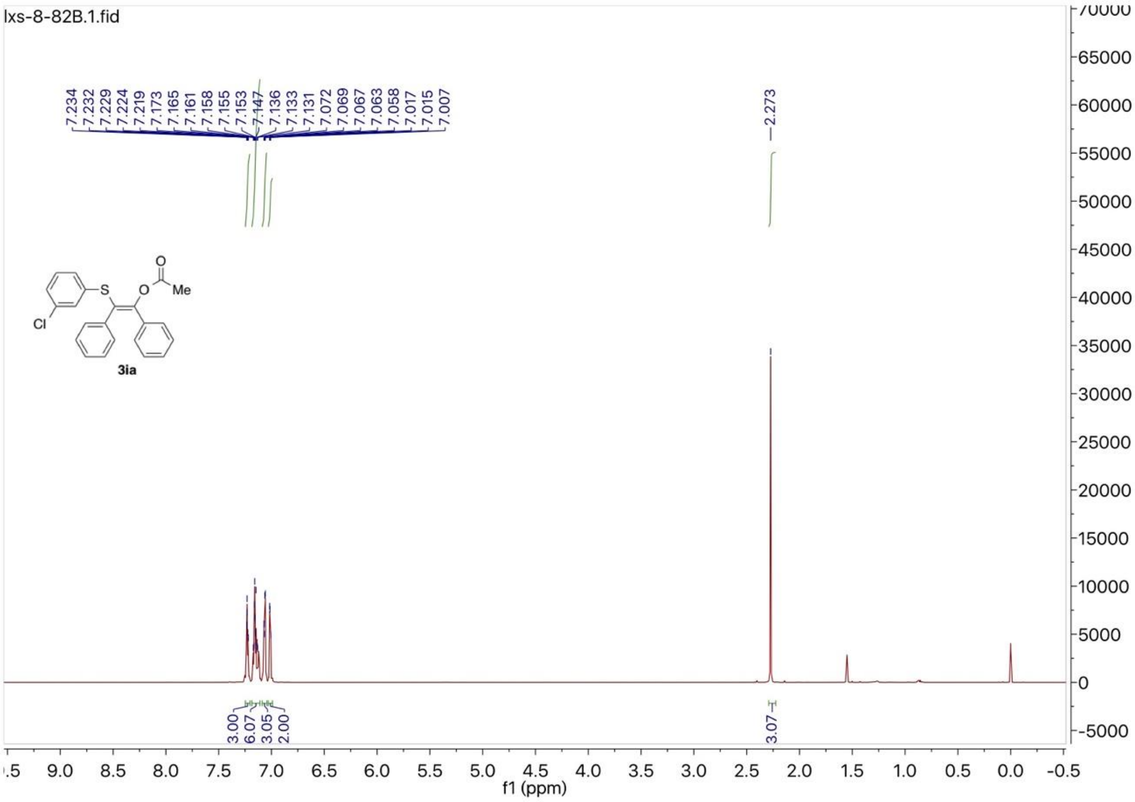

Supplementary Figure 133. ^{1}H NMR (500 MHz, $CDCl_3$) of compound **3ia**

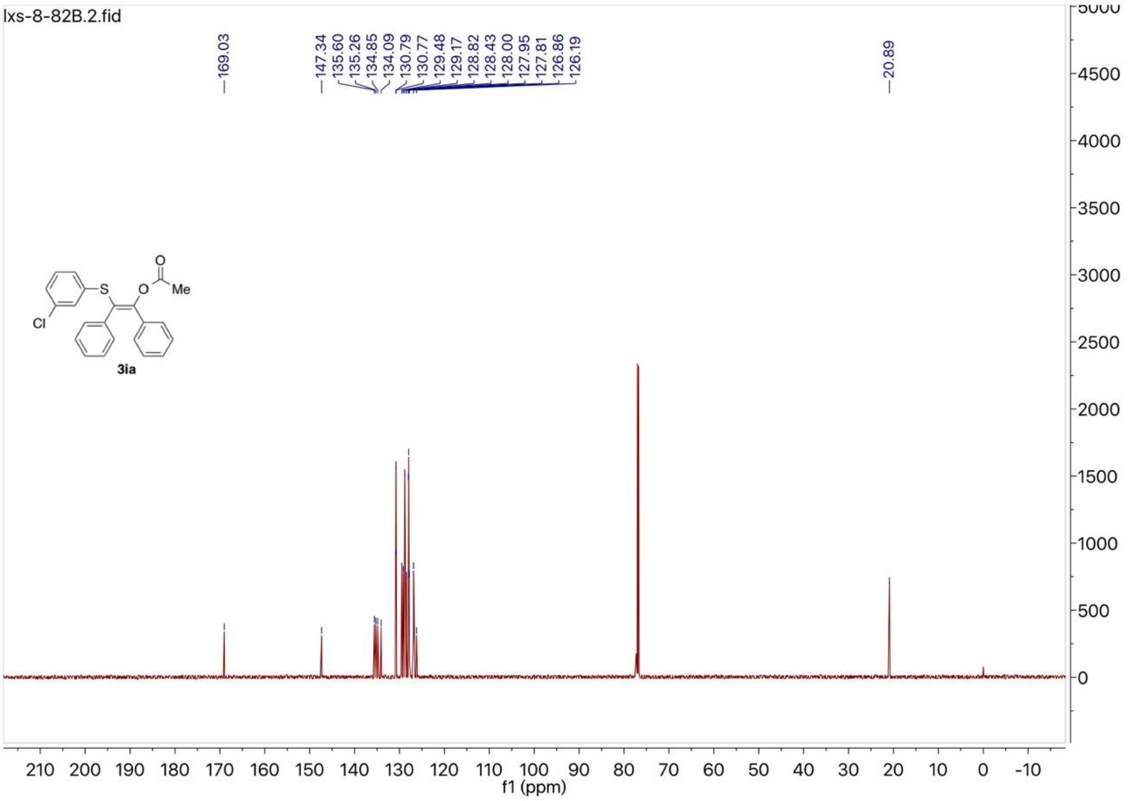

Supplementary Figure 134. ^{13}C NMR (125 MHz, $CDCl_3$) of compound **3ia**

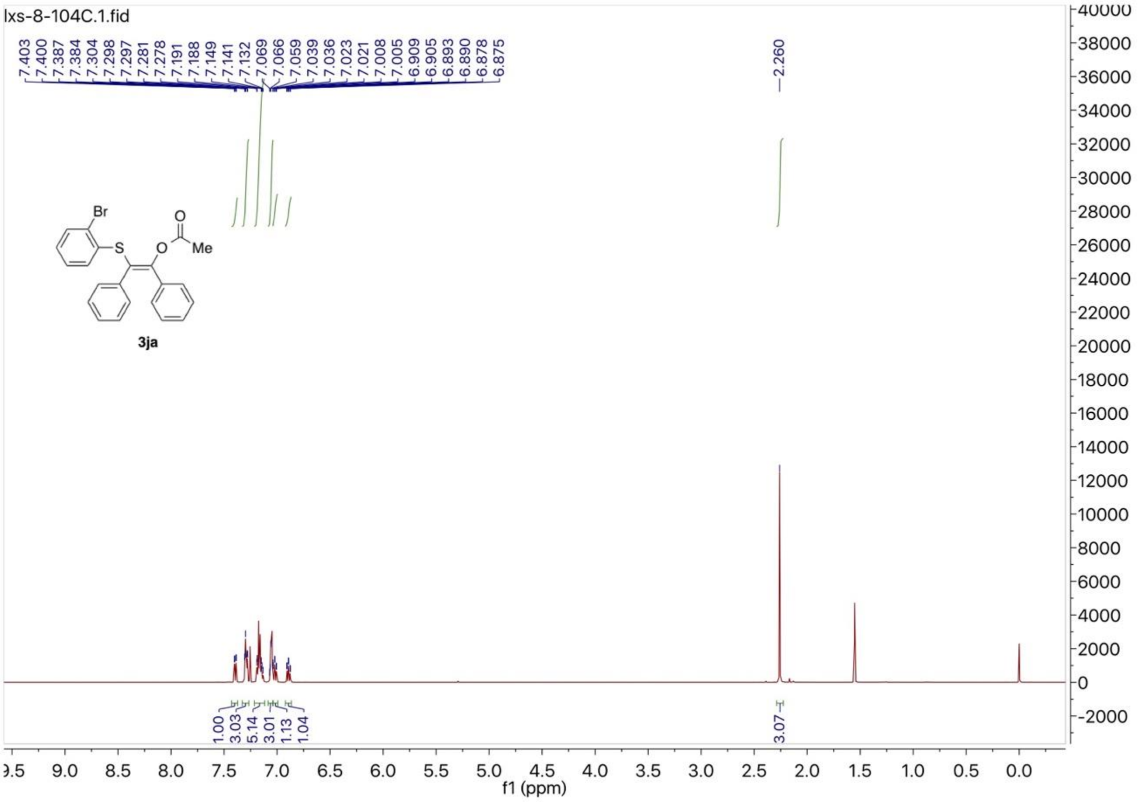

Supplementary Figure 135. ^{1}H NMR (500 MHz, $CDCl_3$) of compound **3ja**

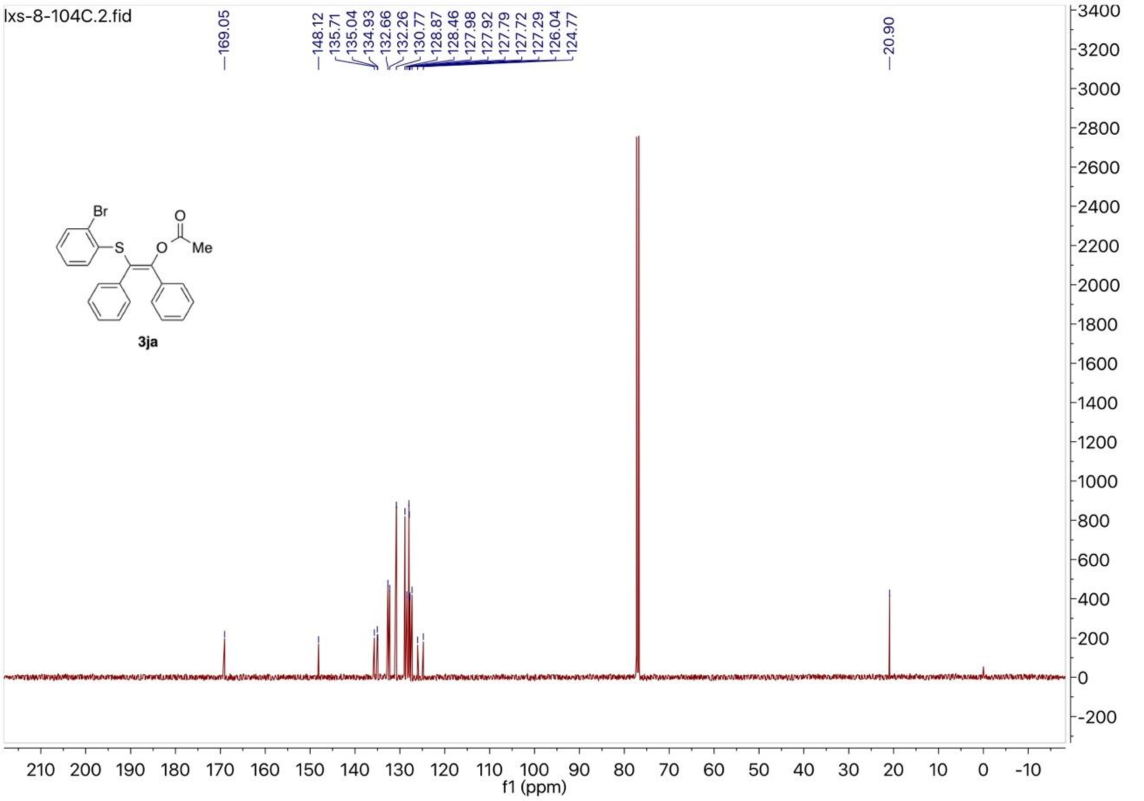

Supplementary Figure 136. ^{13}C NMR (125 MHz, $CDCl_3$) of compound **3ja**

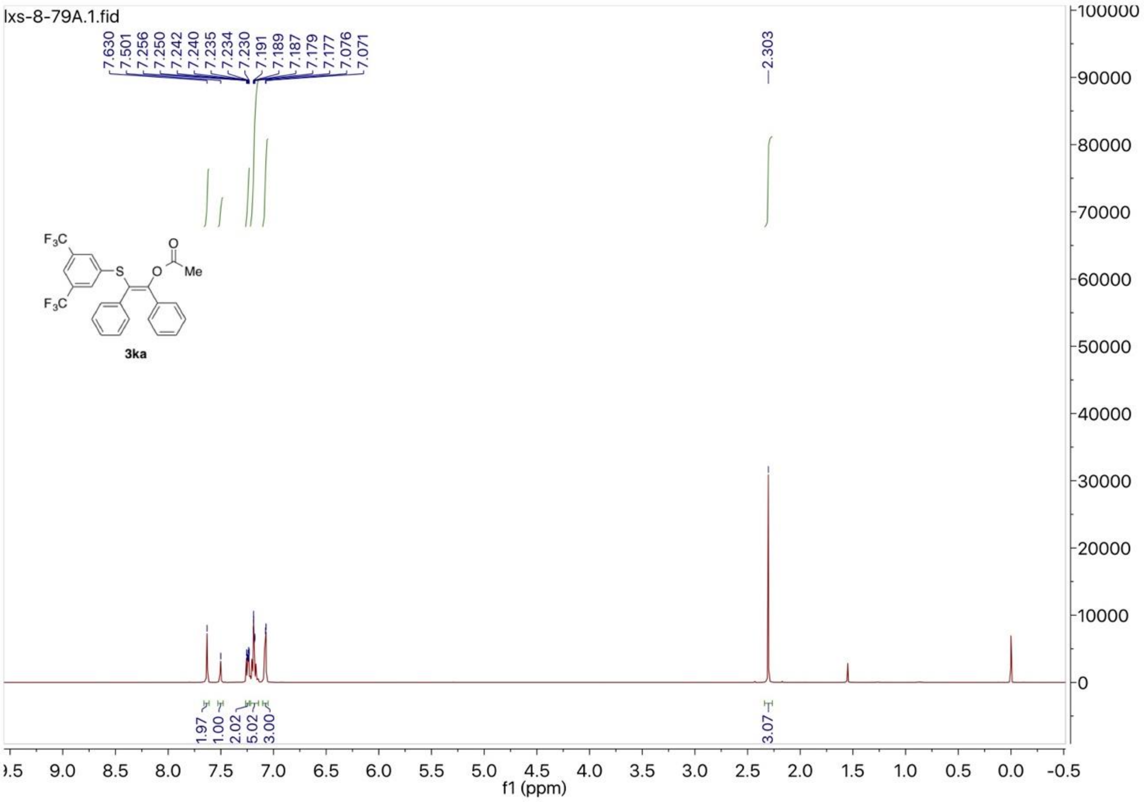

Supplementary Figure 137. ^{1}H NMR (500 MHz, $CDCl_3$) of compound **3ka**

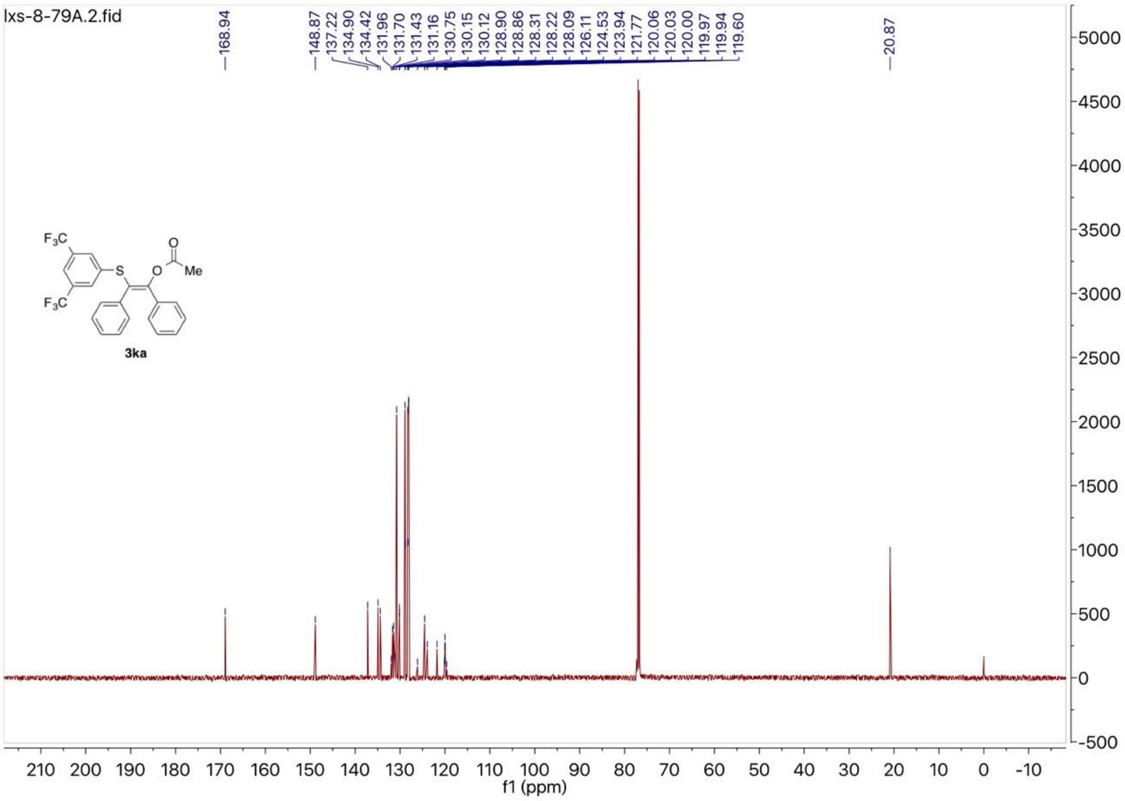

Supplementary Figure 138. ^{13}C NMR (125 MHz, $CDCl_3$) of compound **3ka**

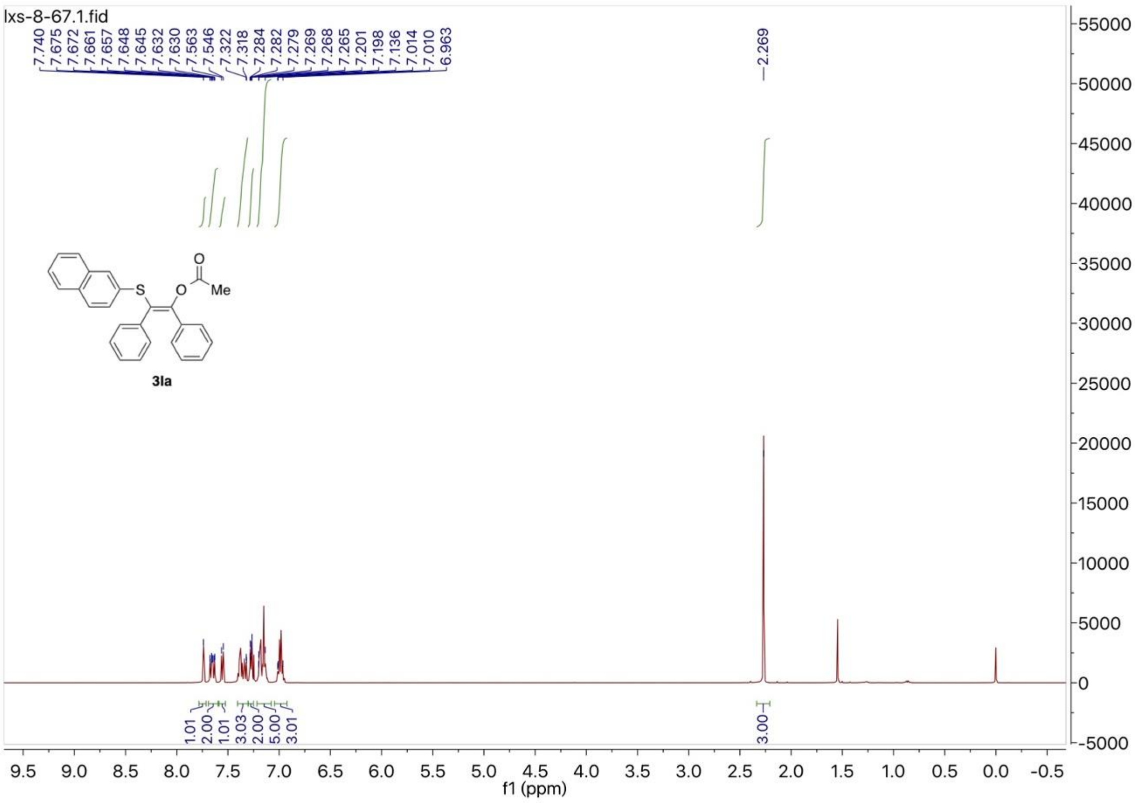

Supplementary Figure 139. ^{1}H NMR (500 MHz, $CDCl_3$) of compound **3la**

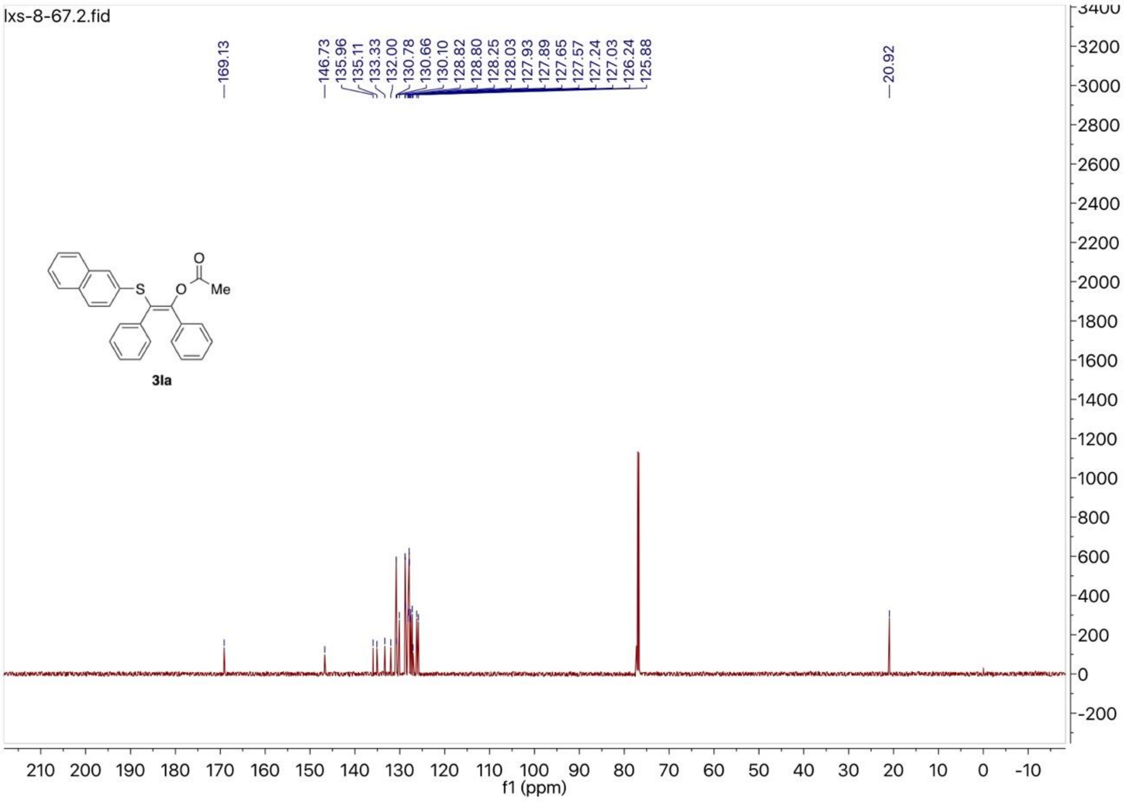

Supplementary Figure 140. ^{13}C NMR (125 MHz, $CDCl_3$) of compound **3la**

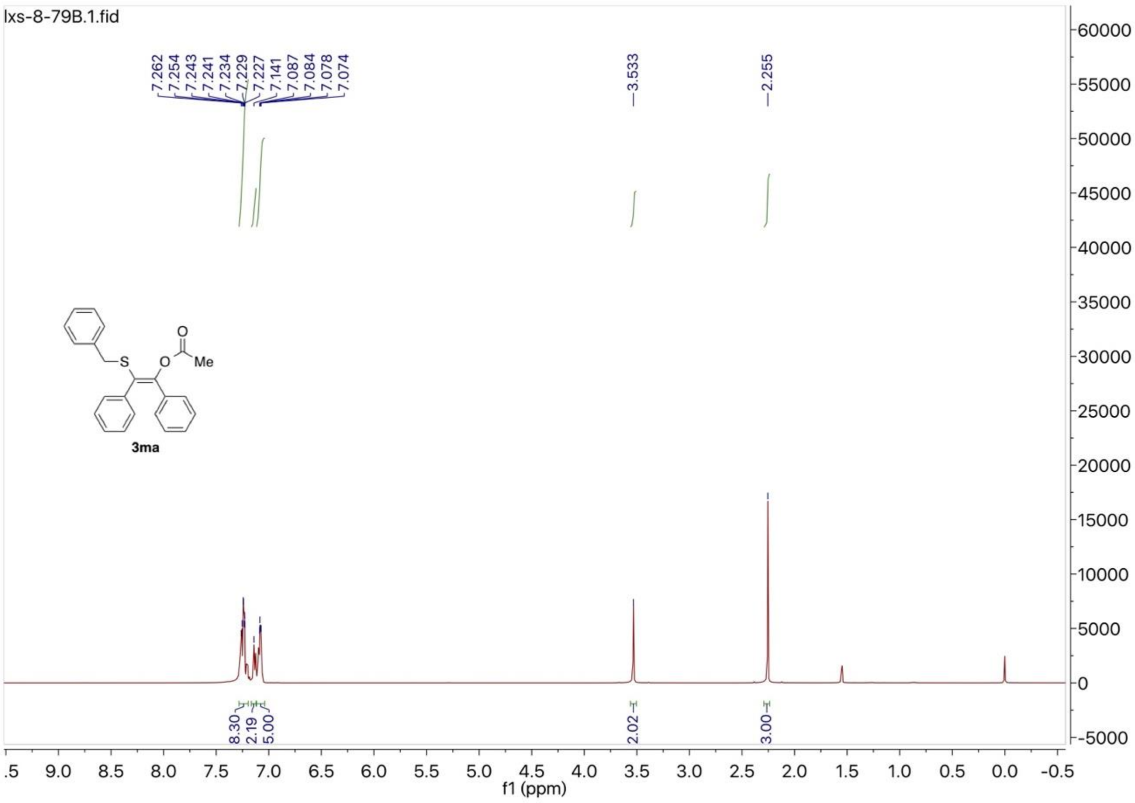

Supplementary Figure 141. ^{1}H NMR (500 MHz, $CDCl_3$) of compound **3ma**

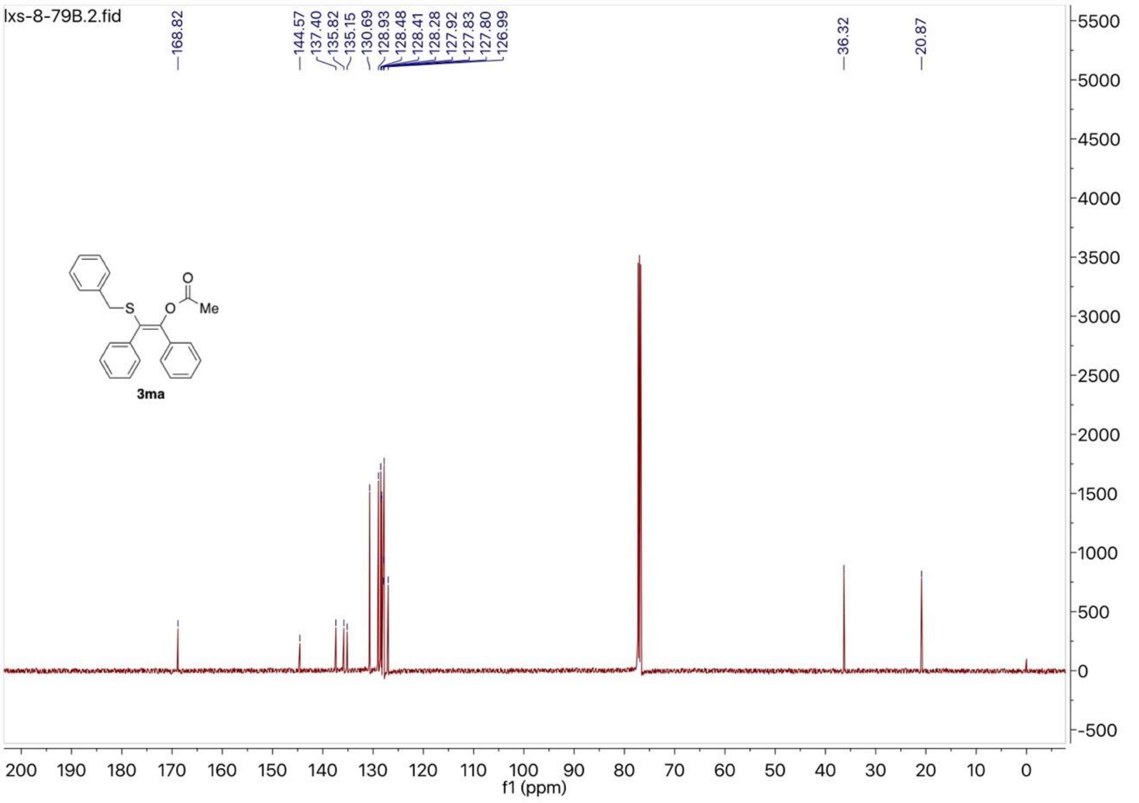

Supplementary Figure 142. ^{13}C NMR (125 MHz, $CDCl_3$) of compound **3ma**

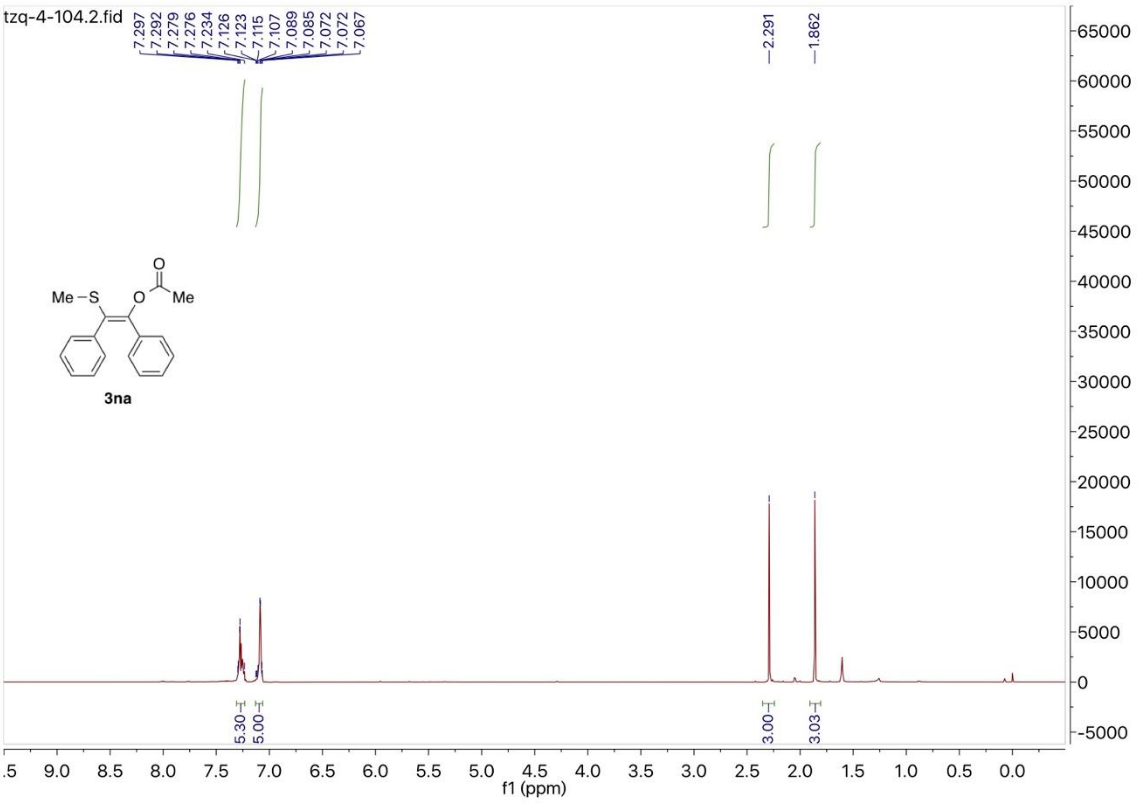

Supplementary Figure 143. ^{1}H NMR (500 MHz, $CDCl_3$) of compound **3na**

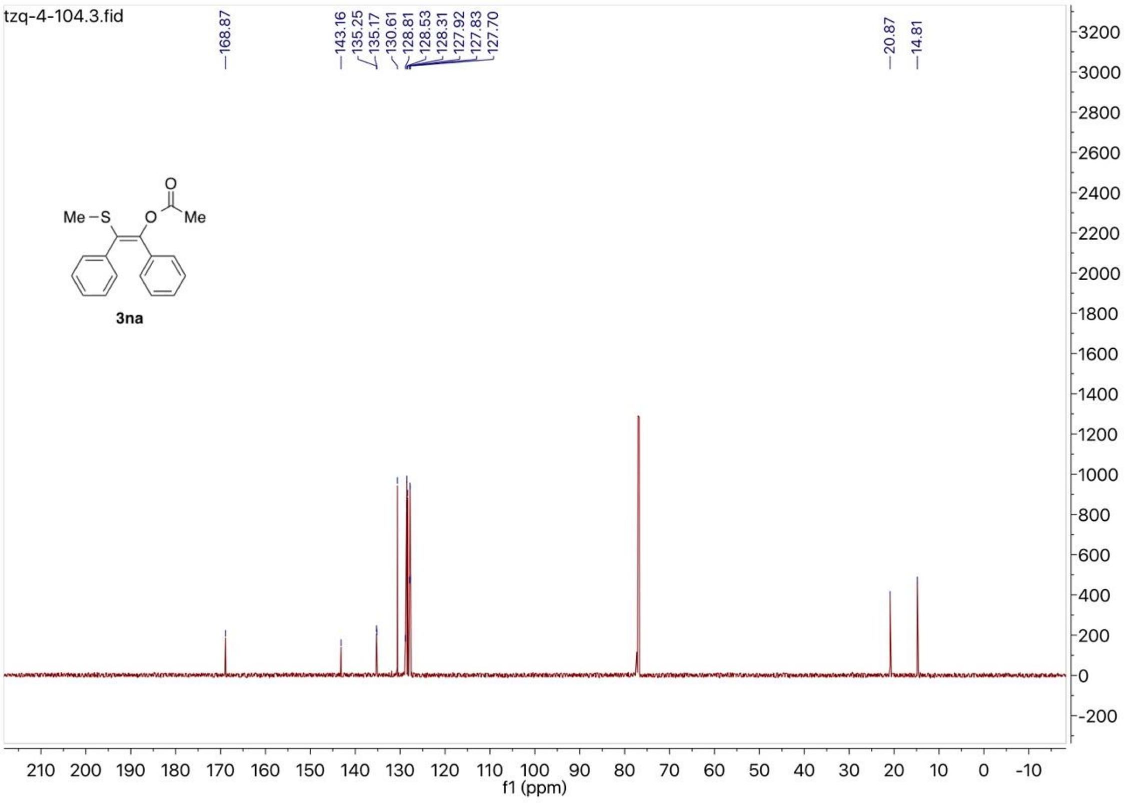

Supplementary Figure 144. ^{13}C NMR (125 MHz, $CDCl_3$) of compound **3na**

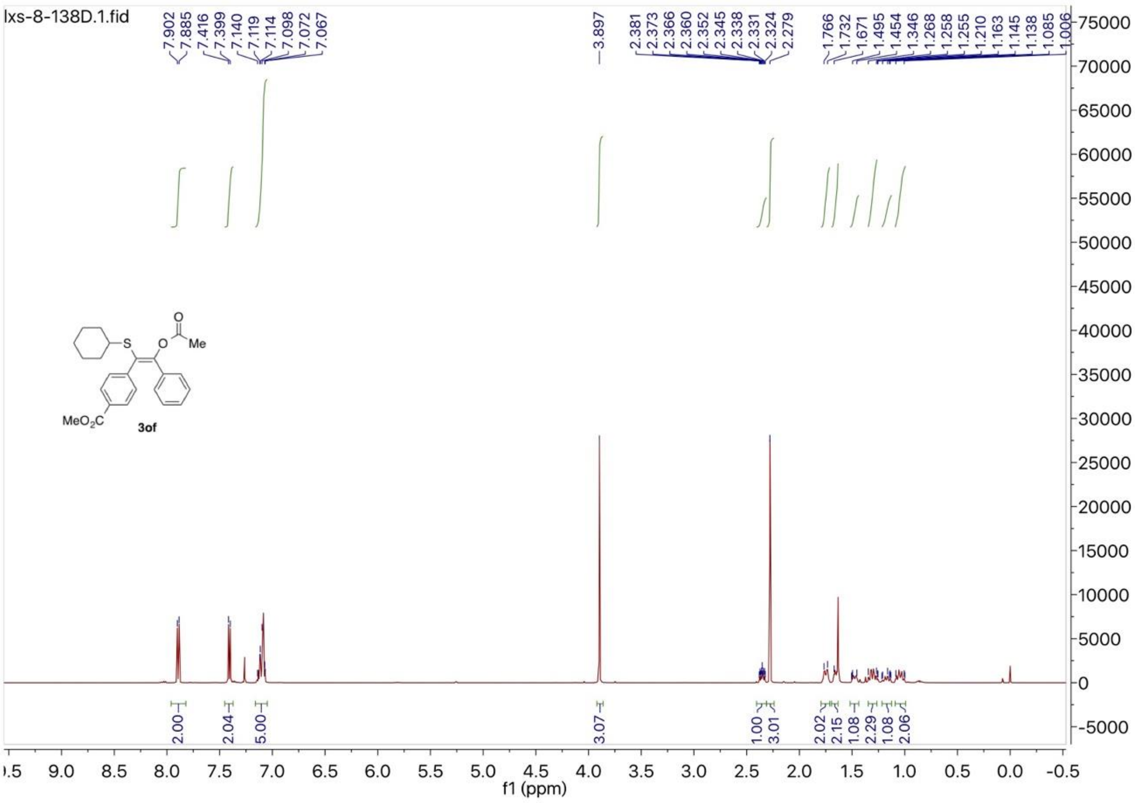

Supplementary Figure 145. ^{1}H NMR (500 MHz, $CDCl_3$) of compound **3of**

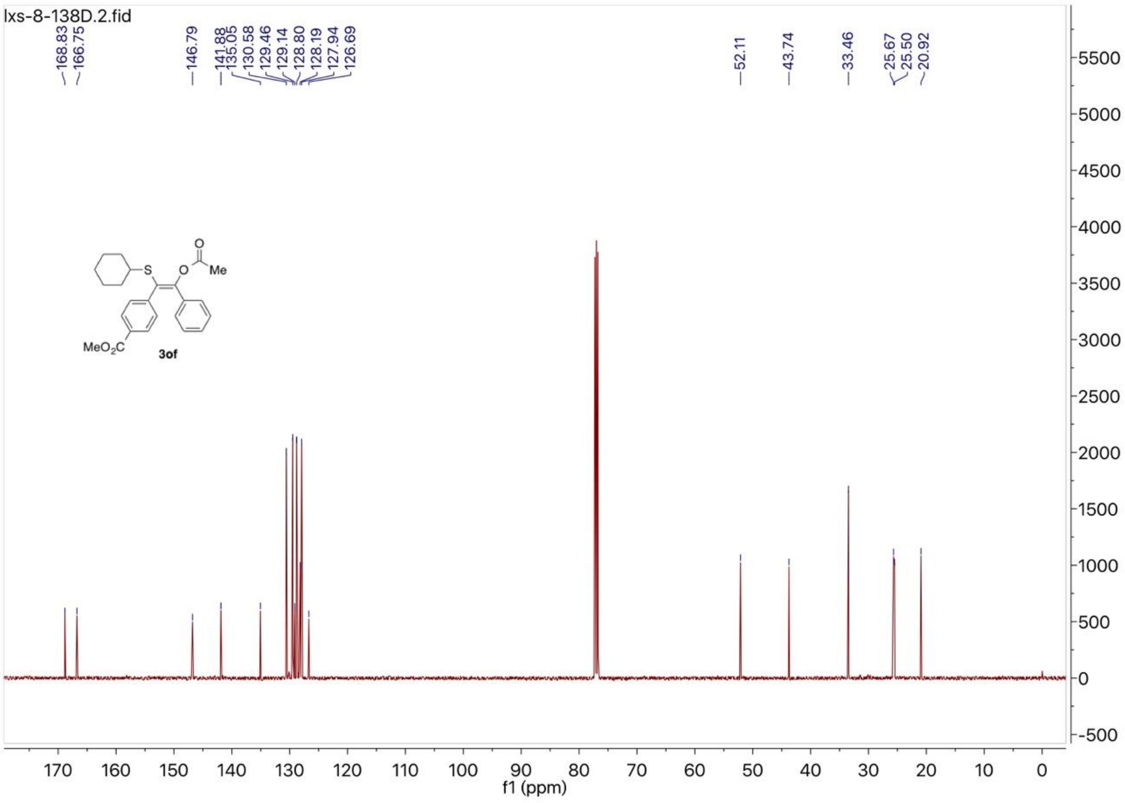

Supplementary Figure 146. ^{13}C NMR (125 MHz, $CDCl_3$) of compound **3of**

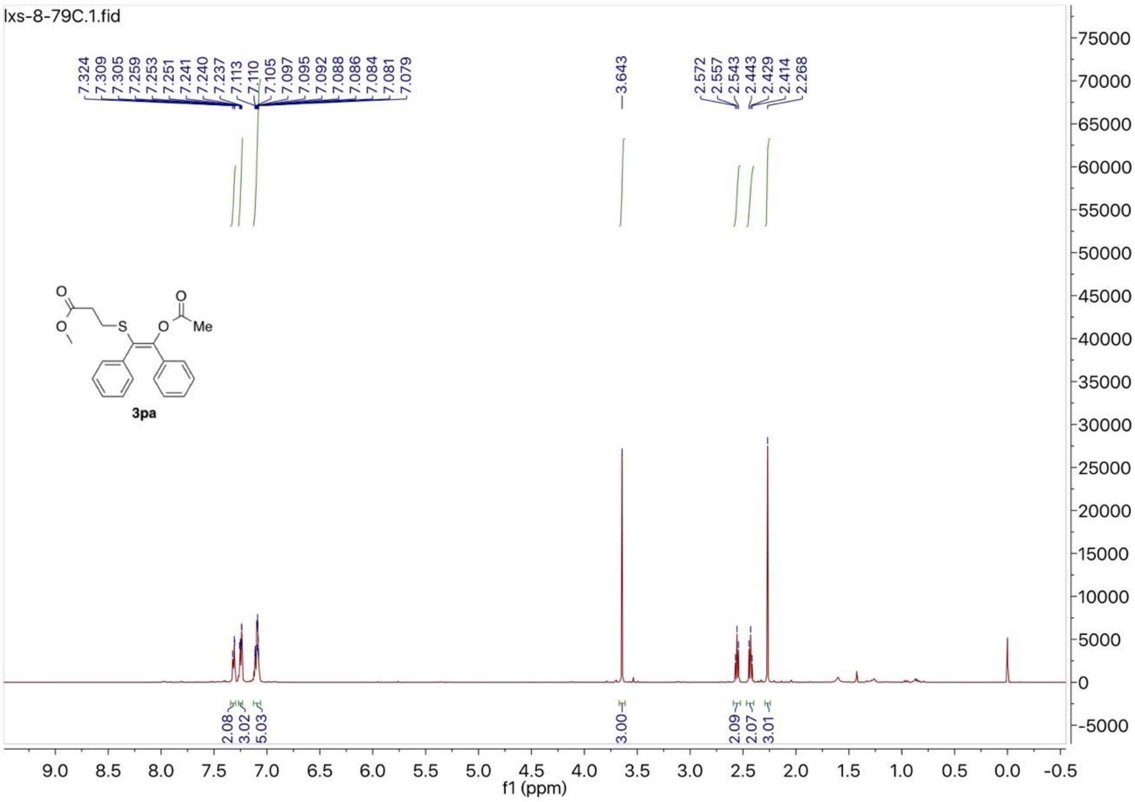

Supplementary Figure 147. ^{1}H NMR (500 MHz, $CDCl_3$) of compound **3pa**

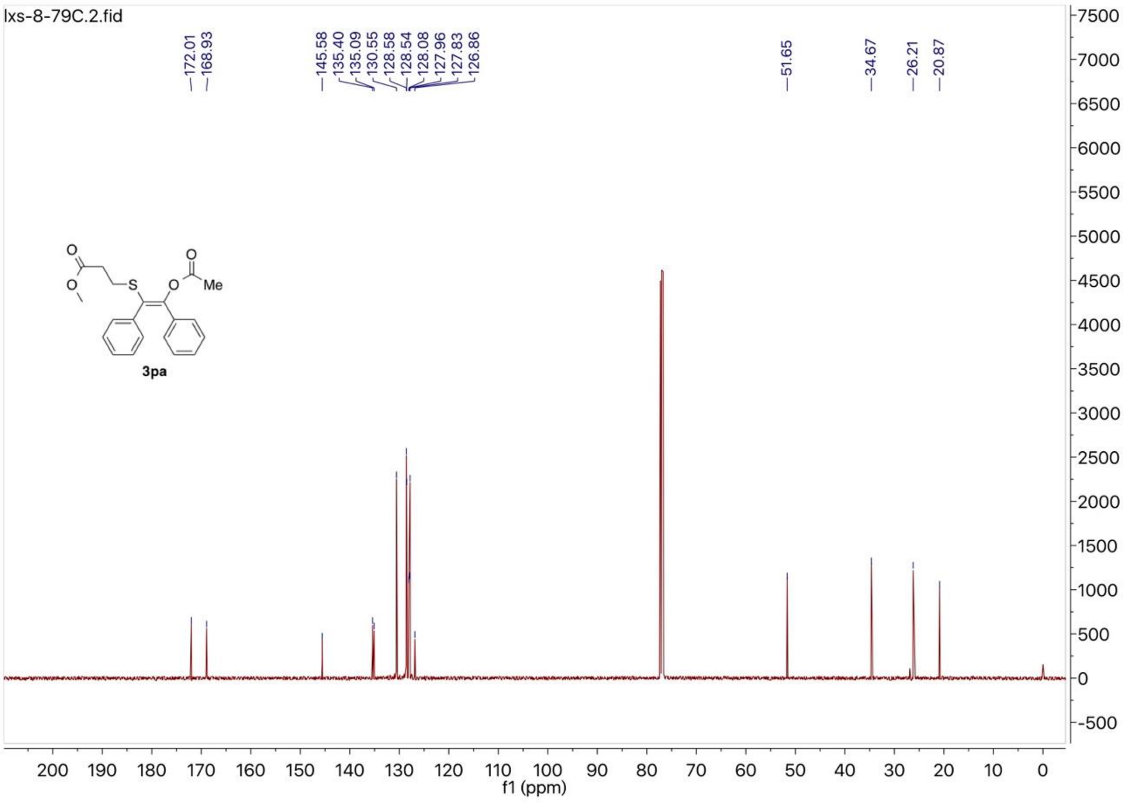

Supplementary Figure 148. ^{13}C NMR (125 MHz, $CDCl_3$) of compound **3pa**

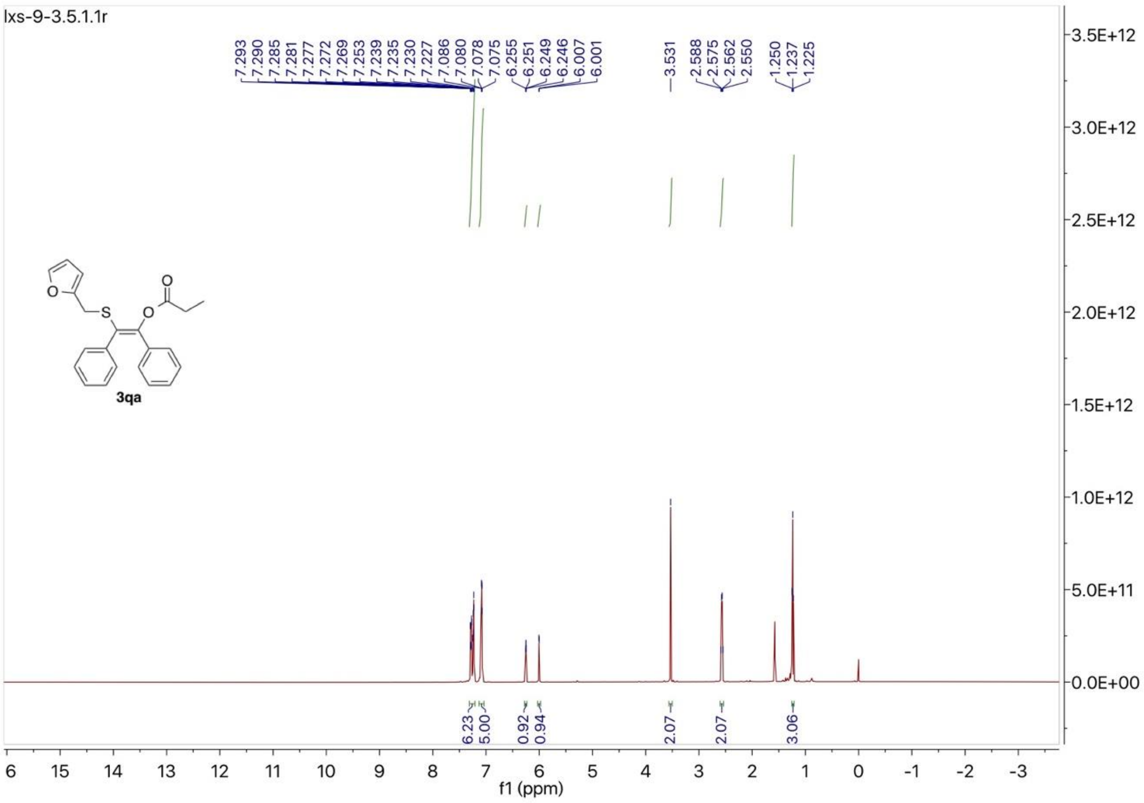

Supplementary Figure 149. ^{1}H NMR (600 MHz, $CDCl_3$) of compound **3qa**

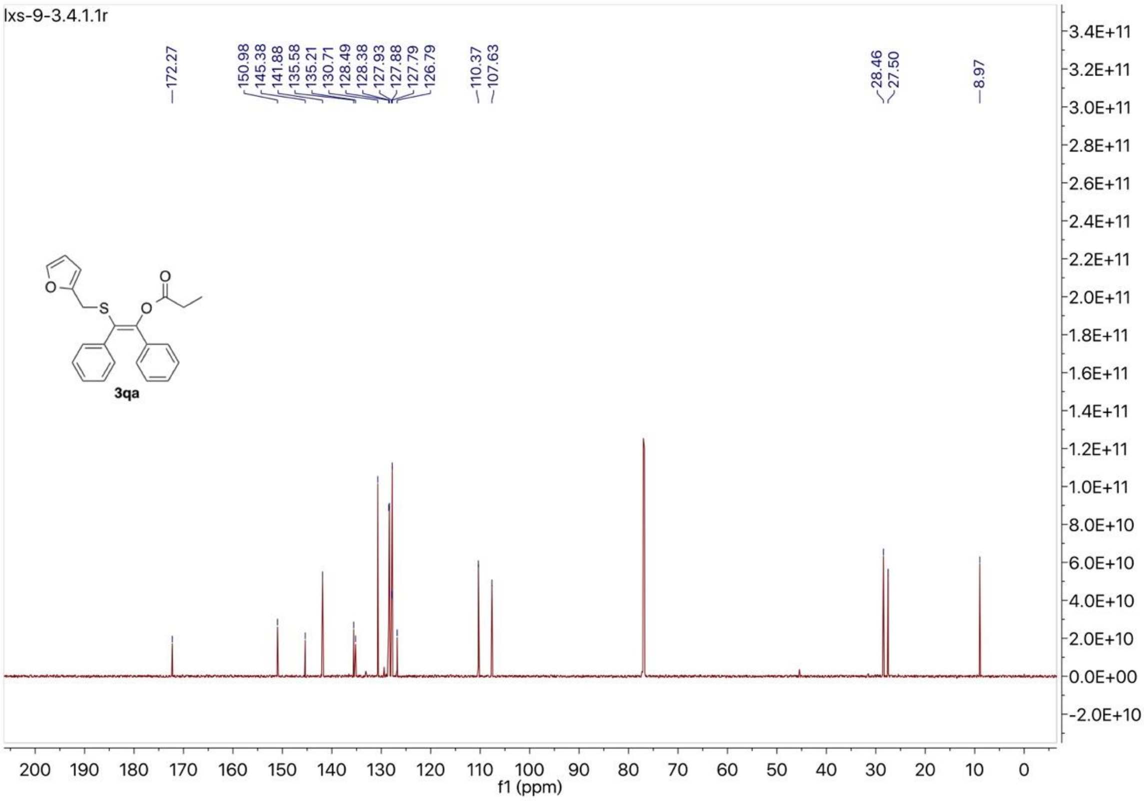

Supplementary Figure 150. ^{13}C NMR (150 MHz, $CDCl_3$) of compound **3qa**

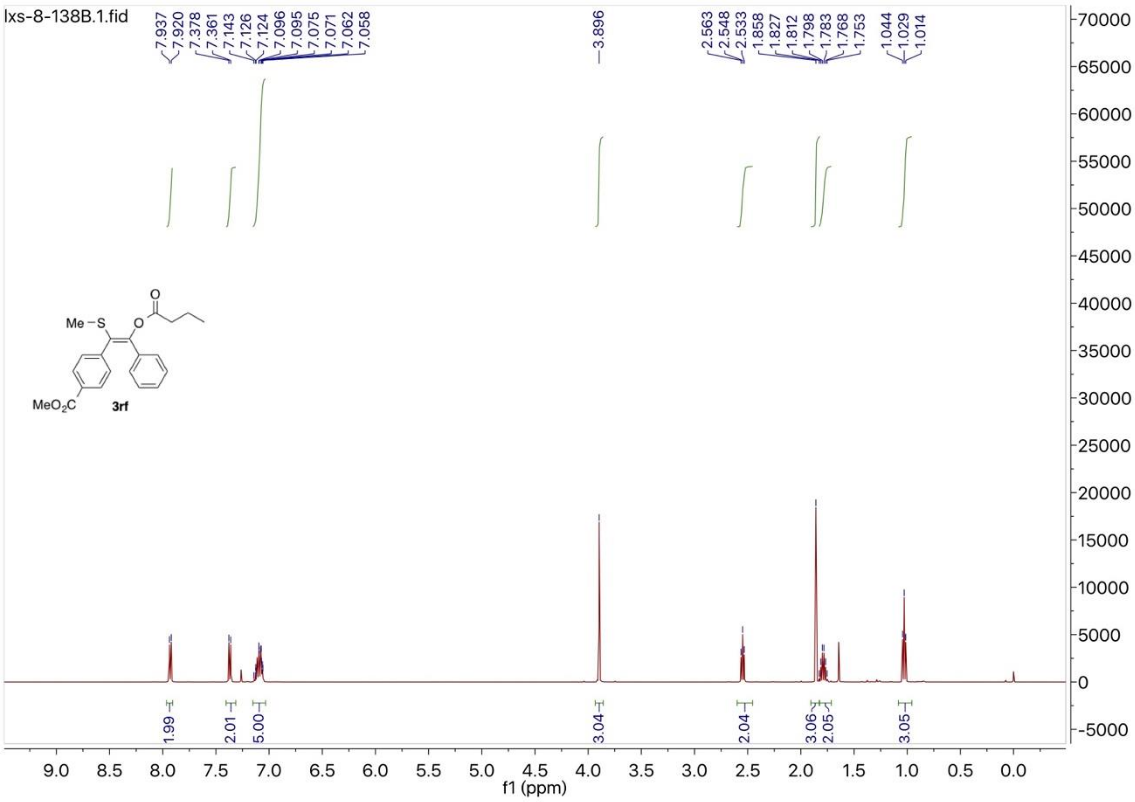

Supplementary Figure 151. ^{1}H NMR (500 MHz, $CDCl_3$) of compound **3rf**

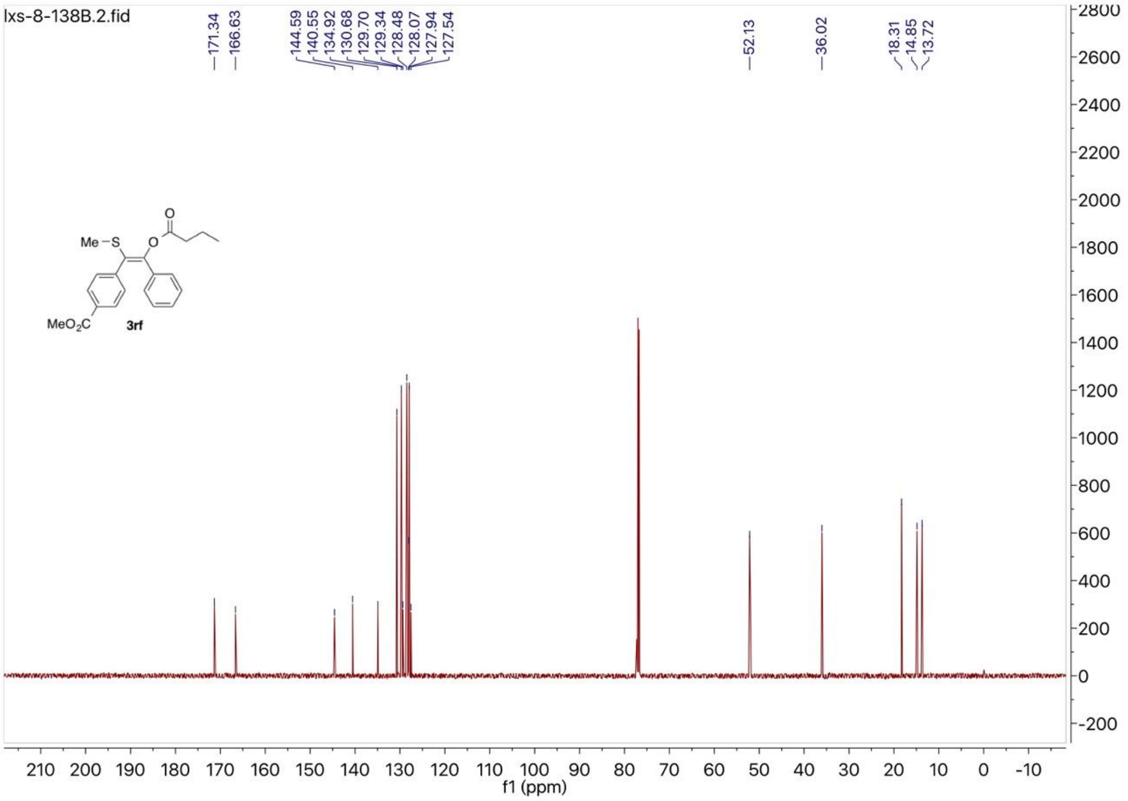

Supplementary Figure 152. ^{13}C NMR (125 MHz, $CDCl_3$) of compound **3rf**

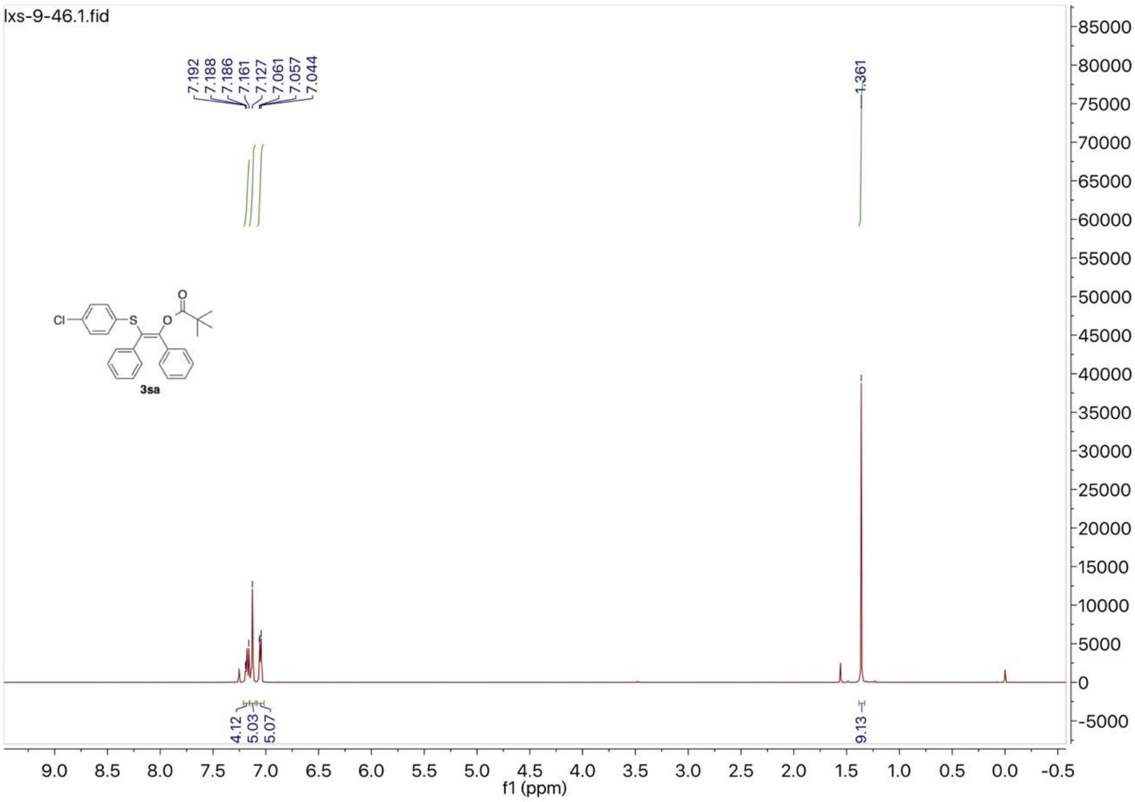

Supplementary Figure 153. ^{1}H NMR (500 MHz, $CDCl_3$) of compound **3sa**

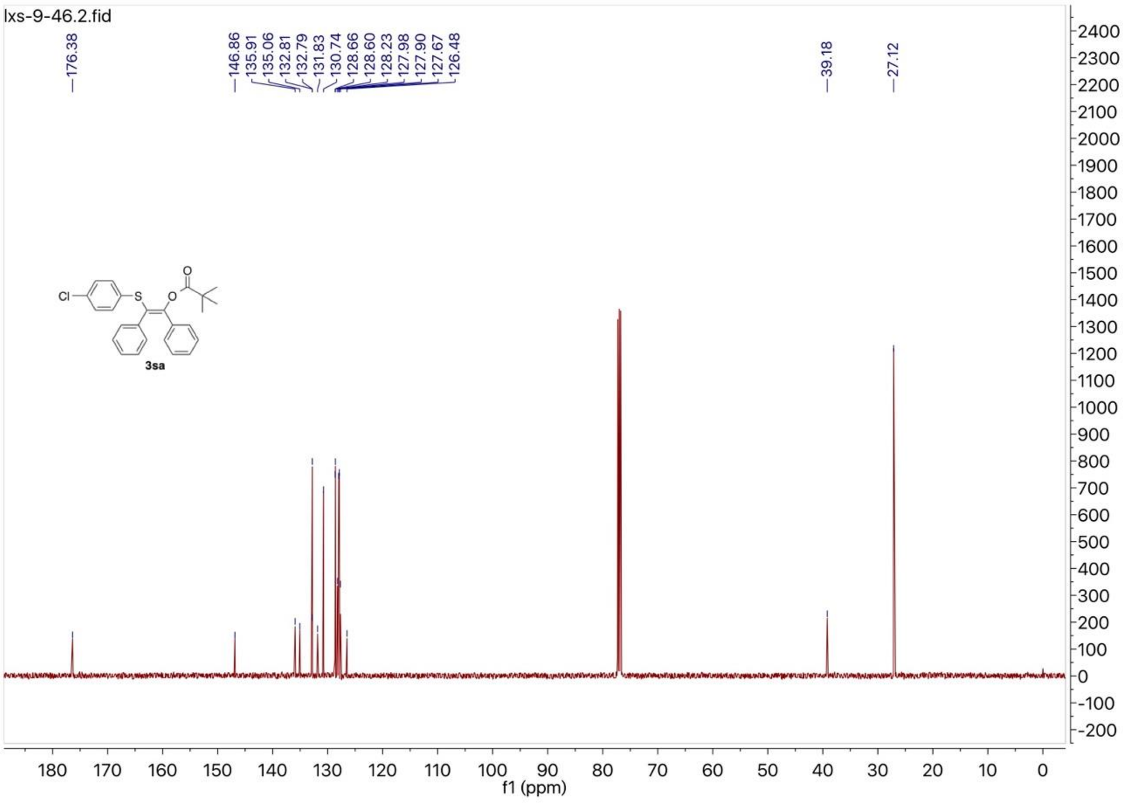

Supplementary Figure 154. ^{13}C NMR (125 MHz, $CDCl_3$) of compound **3sa**

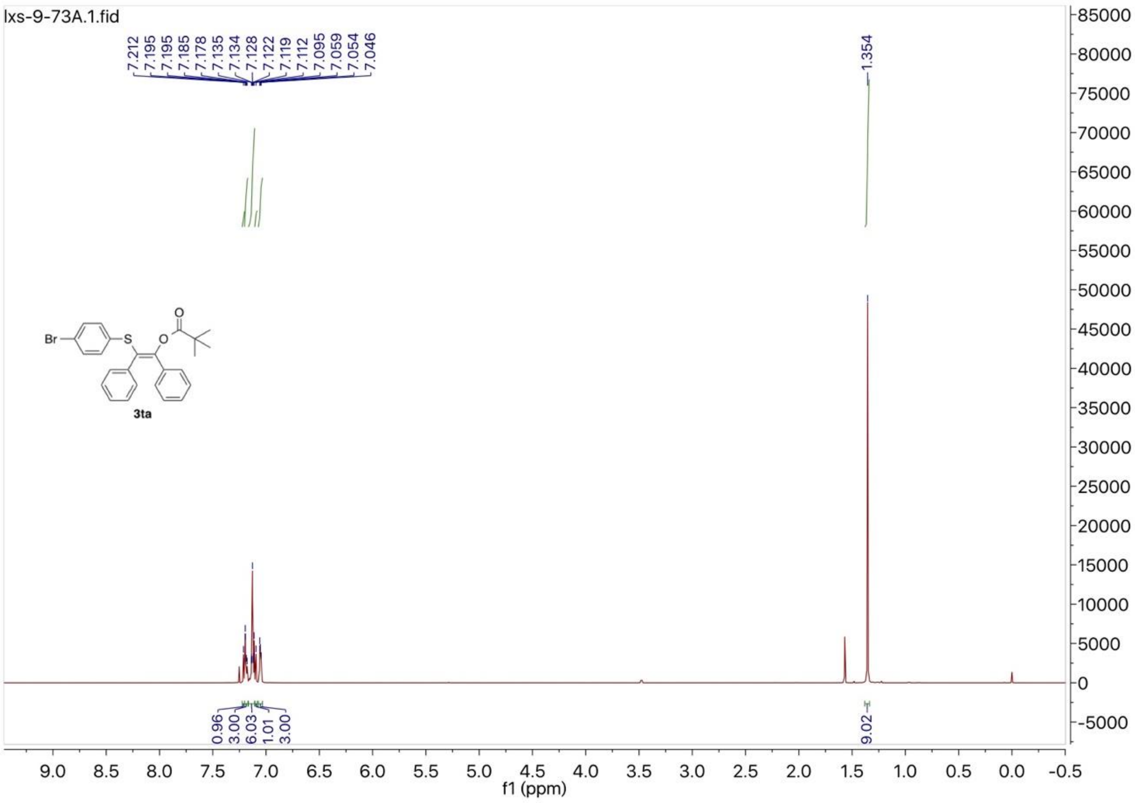

Supplementary Figure 155. ^{1}H NMR (500 MHz, $CDCl_3$) of compound **3ta**

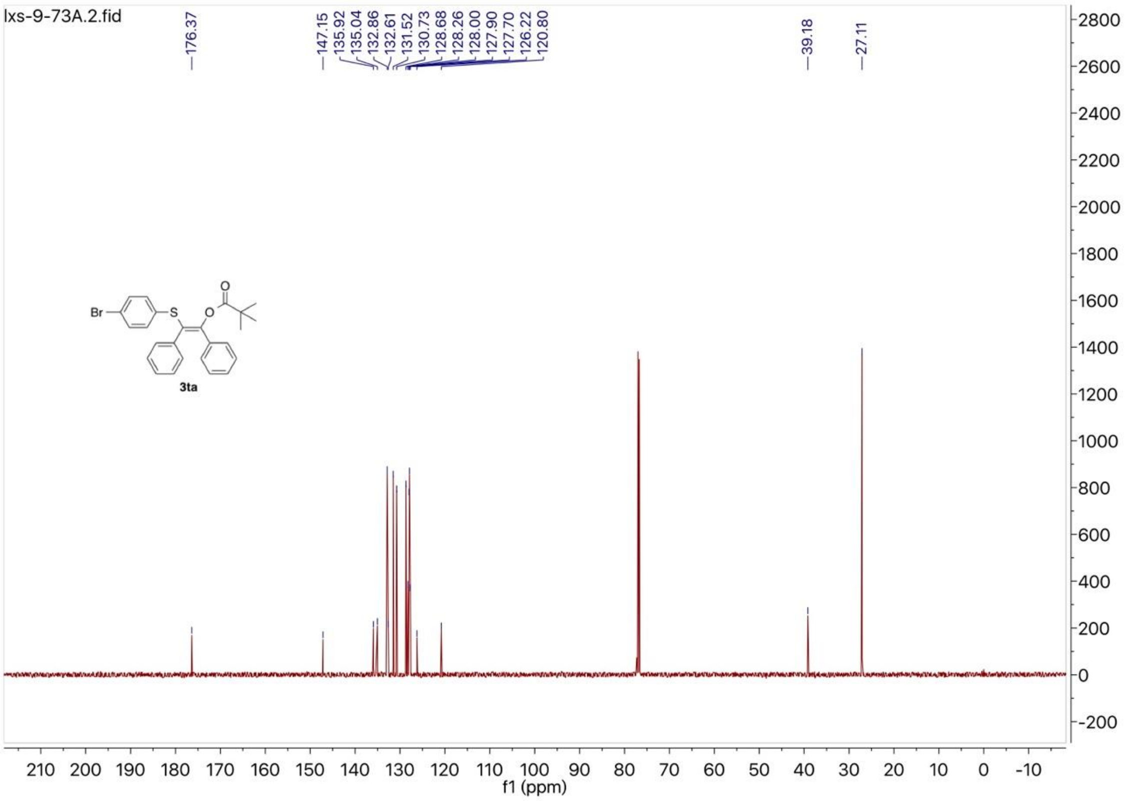

Supplementary Figure 156. ^{13}C NMR (125 MHz, $CDCl_3$) of compound **3ta**

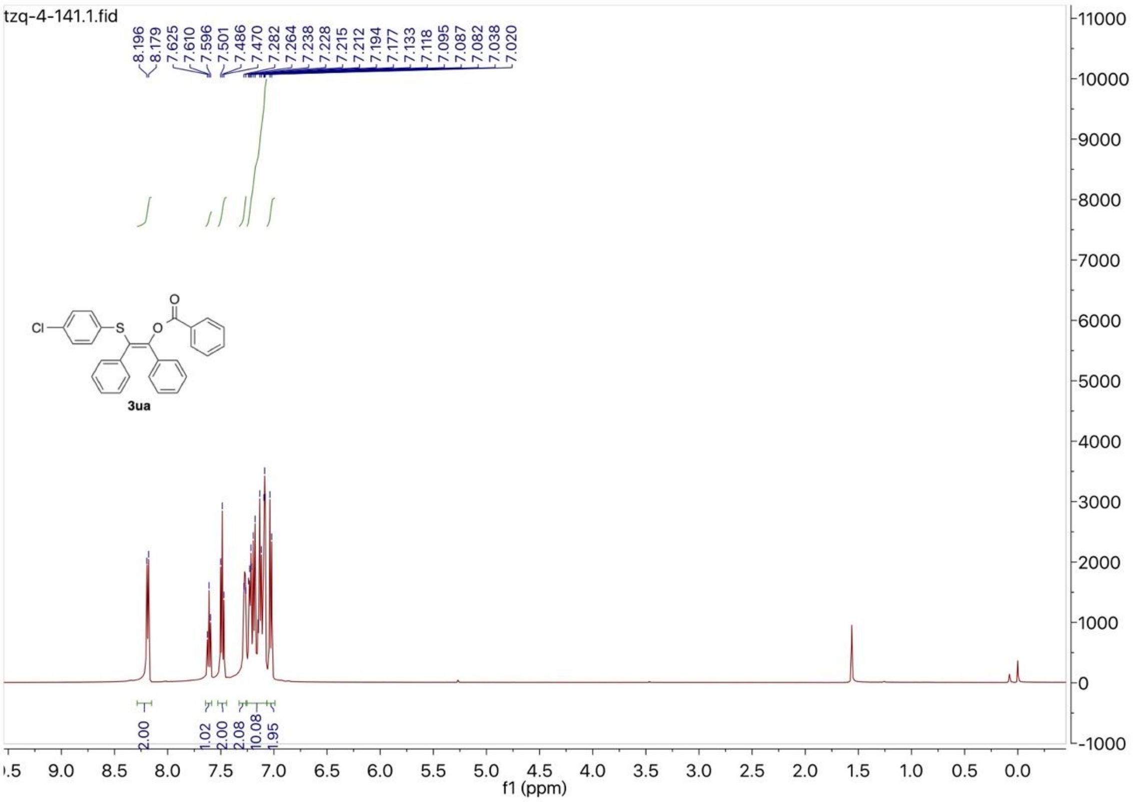

Supplementary Figure 157. ^{1}H NMR (500 MHz, $CDCl_3$) of compound **3ua**

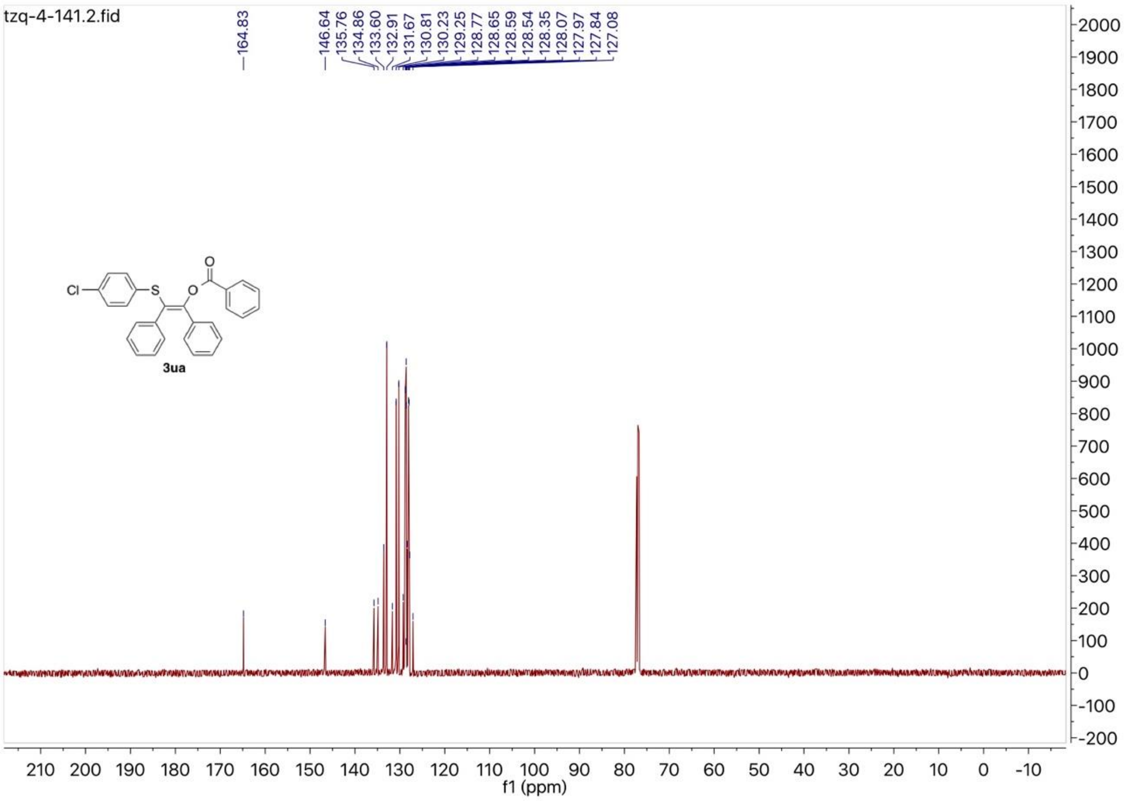

Supplementary Figure 158. ^{13}C NMR (125 MHz, $CDCl_3$) of compound **3ua**

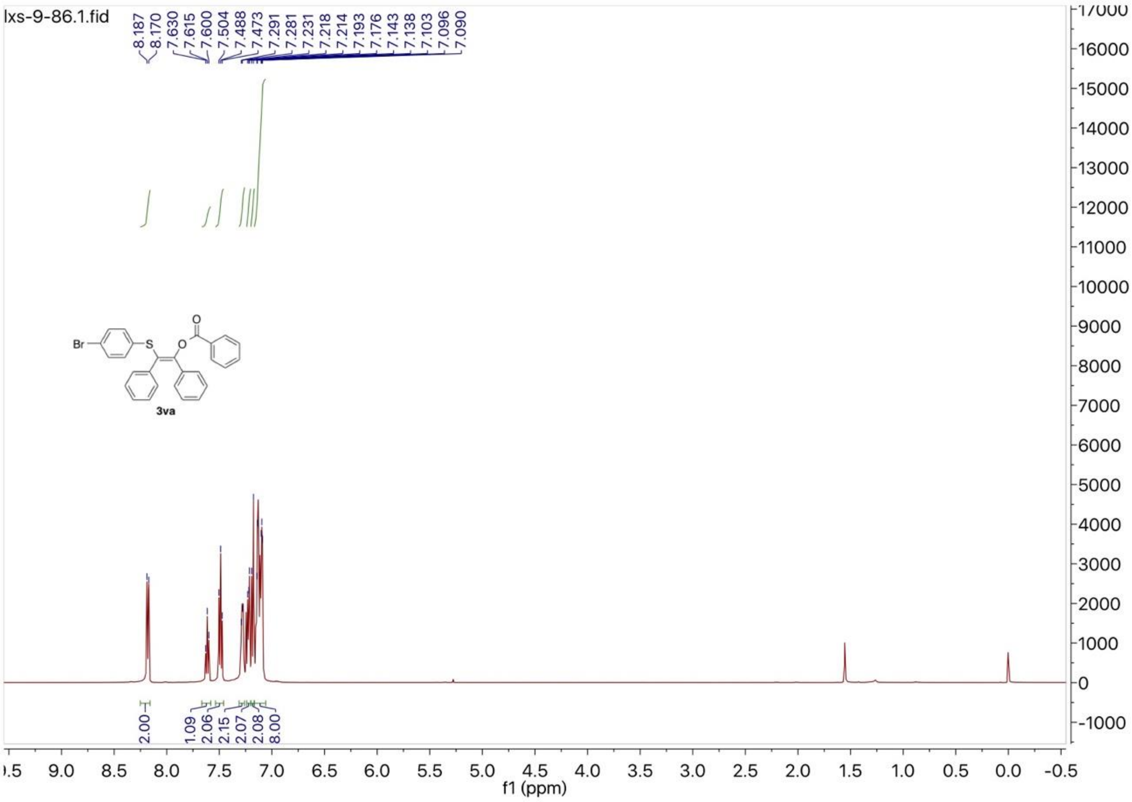

Supplementary Figure 159. ^{1}H NMR (500 MHz, $CDCl_3$) of compound **3va**

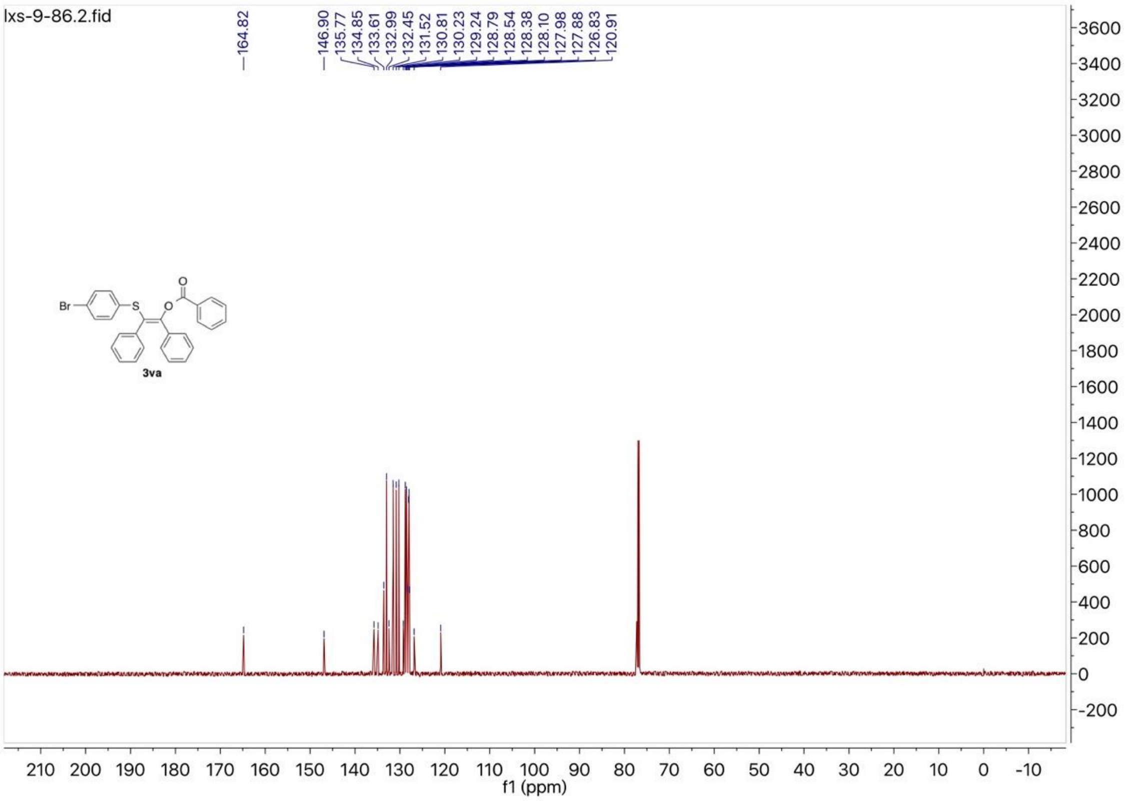

Supplementary Figure 160. ^{13}C NMR (125 MHz, $CDCl_3$) of compound **3va**

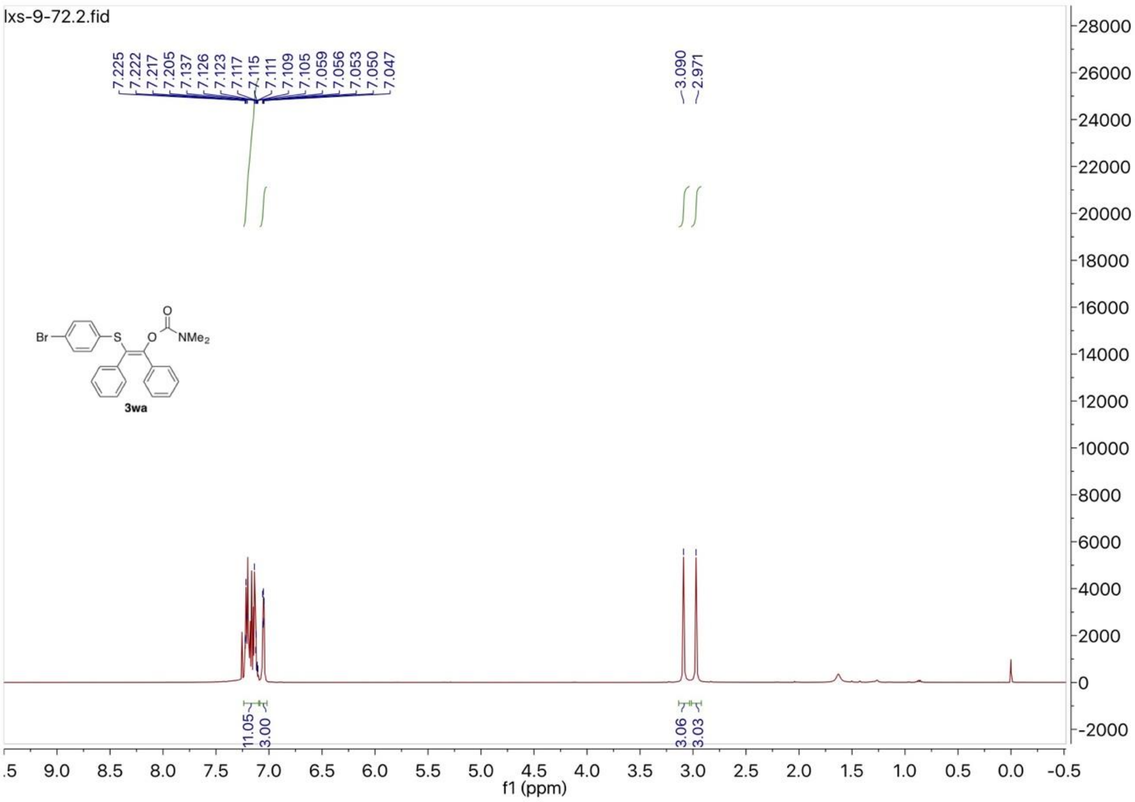

Supplementary Figure 161. ^{1}H NMR (500 MHz, $CDCl_3$) of compound **3wa**

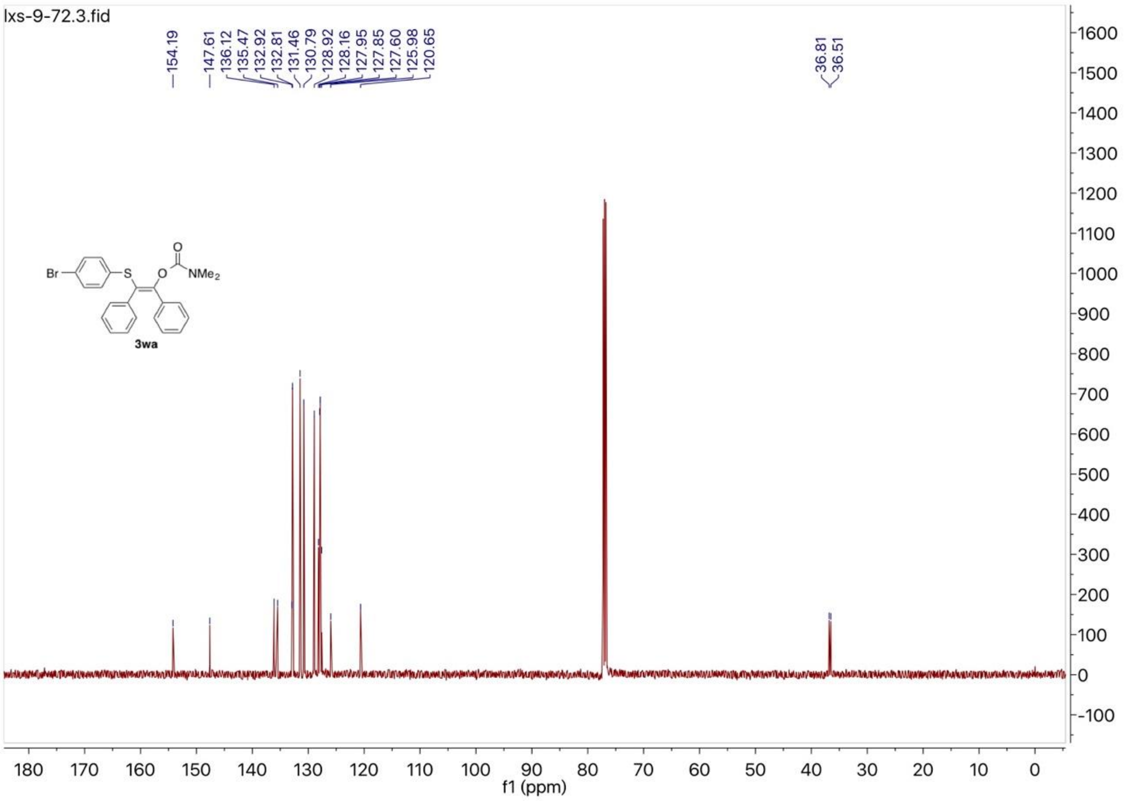

Supplementary Figure 162. ^{13}C NMR (125 MHz, $CDCl_3$) of compound **3wa**

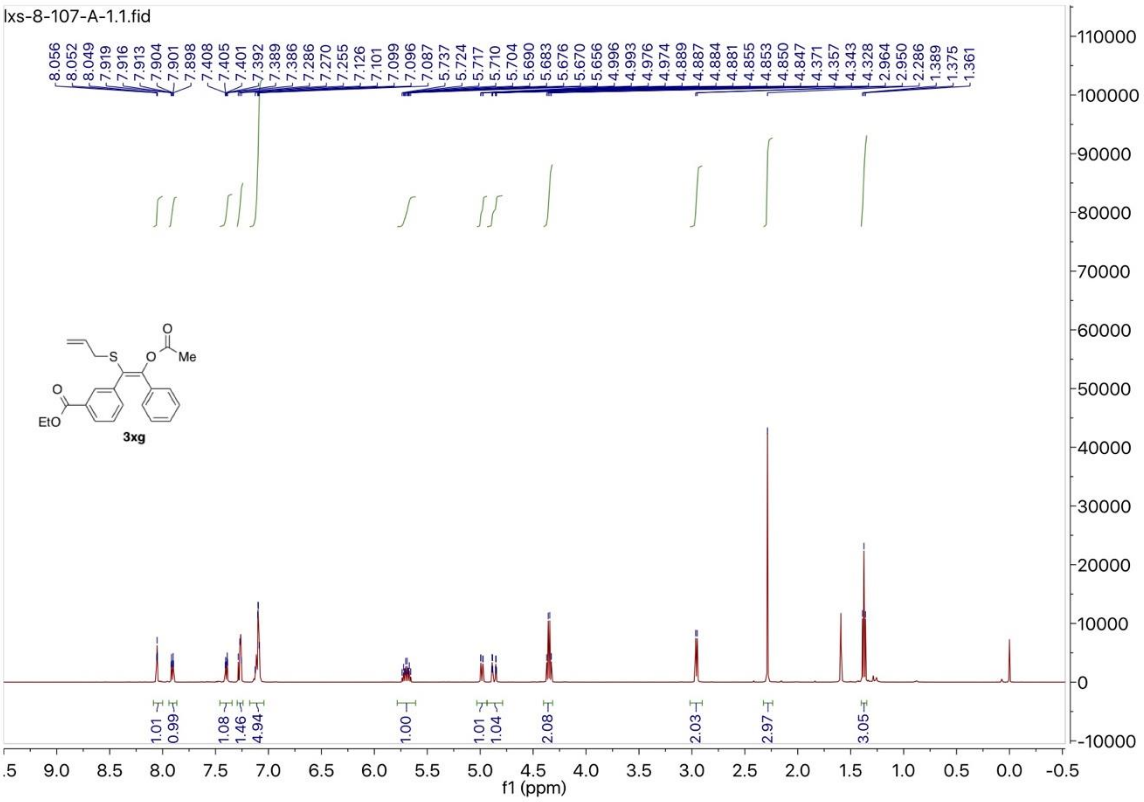

Supplementary Figure 163. ^{1}H NMR (500 MHz, $CDCl_3$) of compound **3xg**

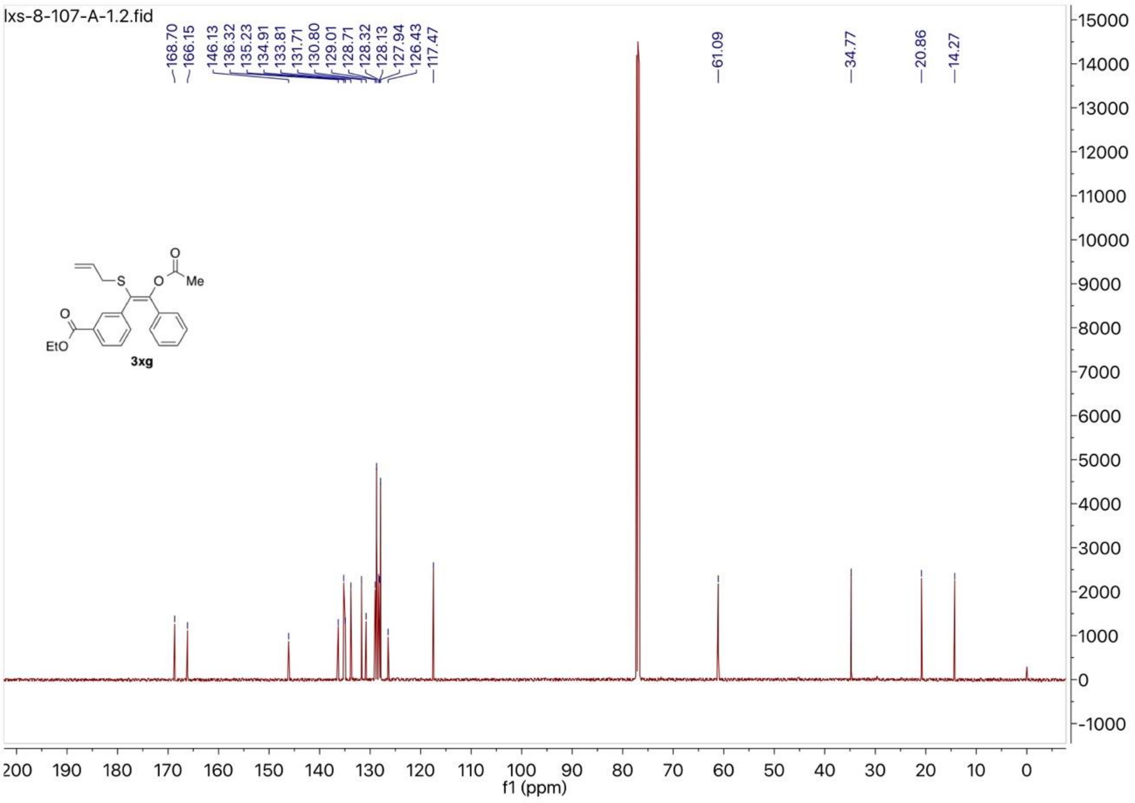

Supplementary Figure 164. ^{13}C NMR (125 MHz, $CDCl_3$) of compound **3xg**

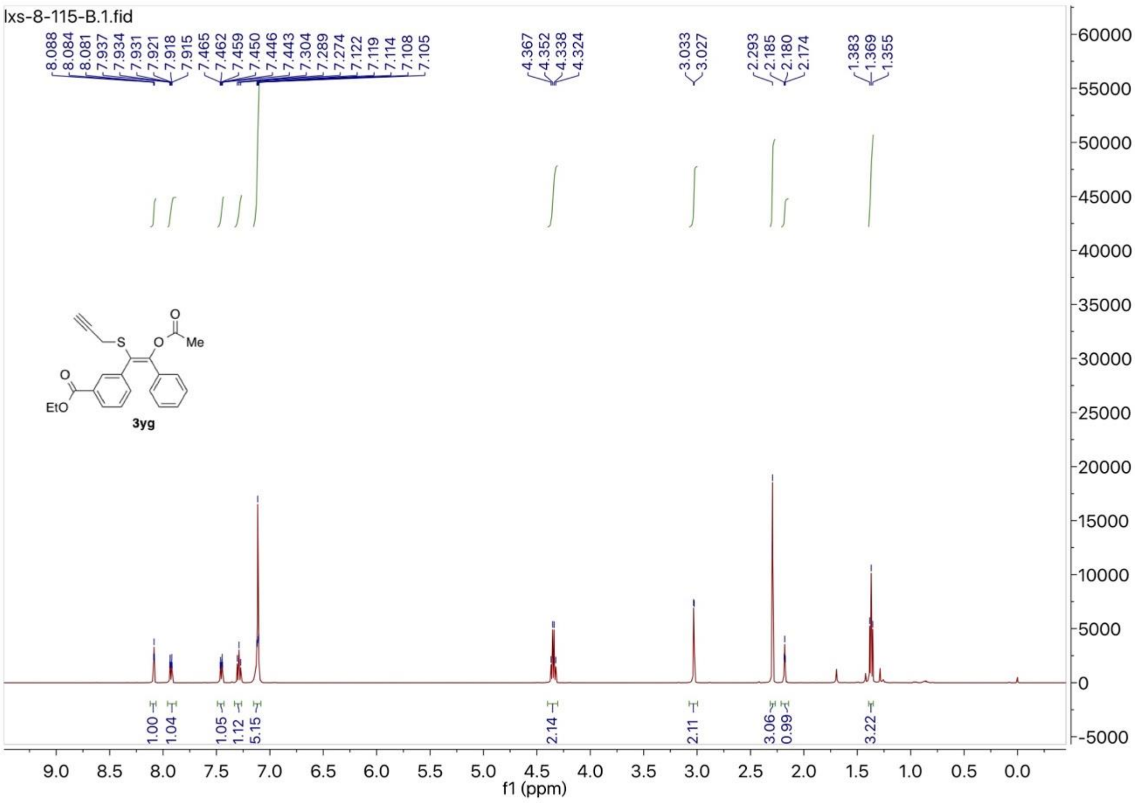

Supplementary Figure 165. ^{1}H NMR (500 MHz, $CDCl_3$) of compound **3yg**

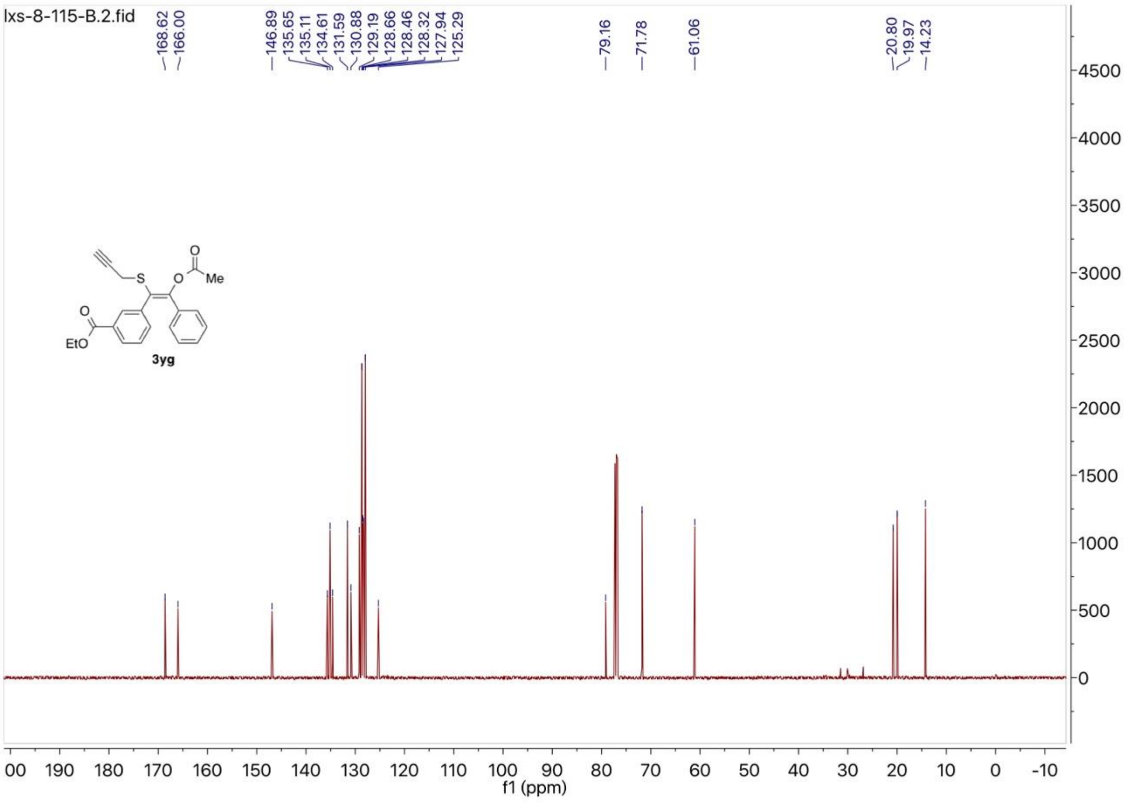

Supplementary Figure 166. ^{13}C NMR (125 MHz, $CDCl_3$) of compound **3yg**

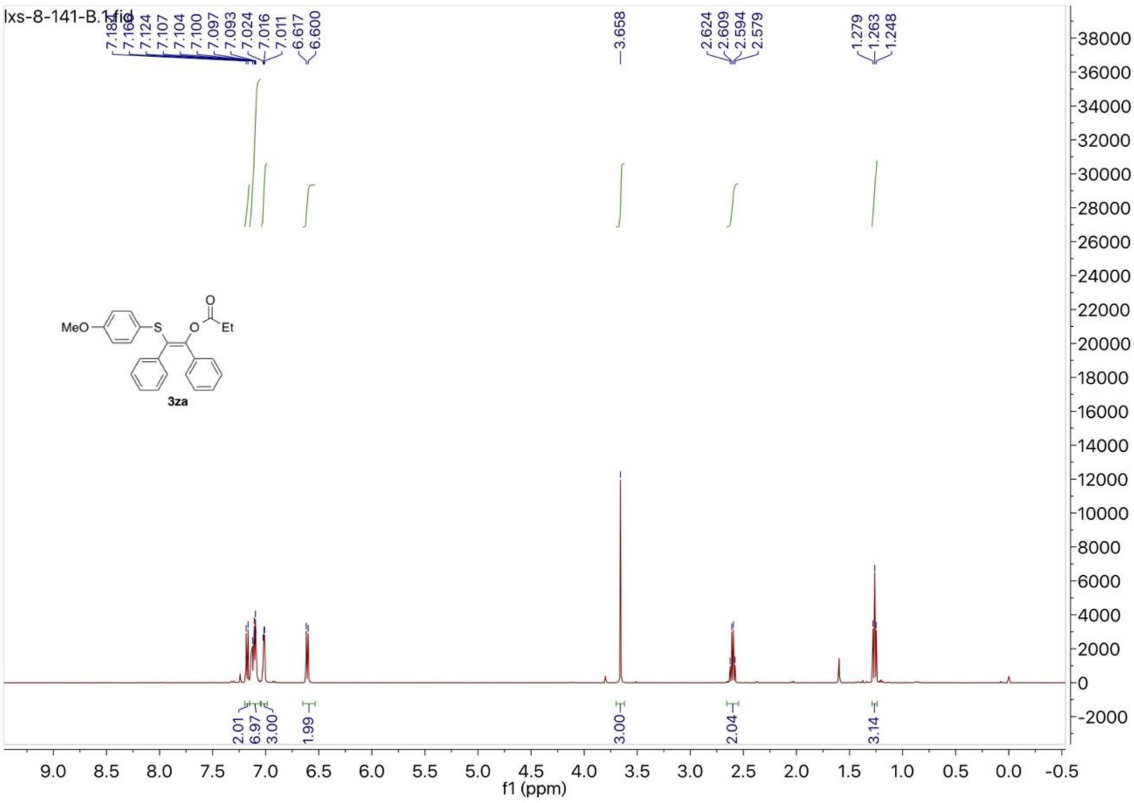

Supplementary Figure 167. ^{1}H NMR (500 MHz, $CDCl_3$) of compound **3za**

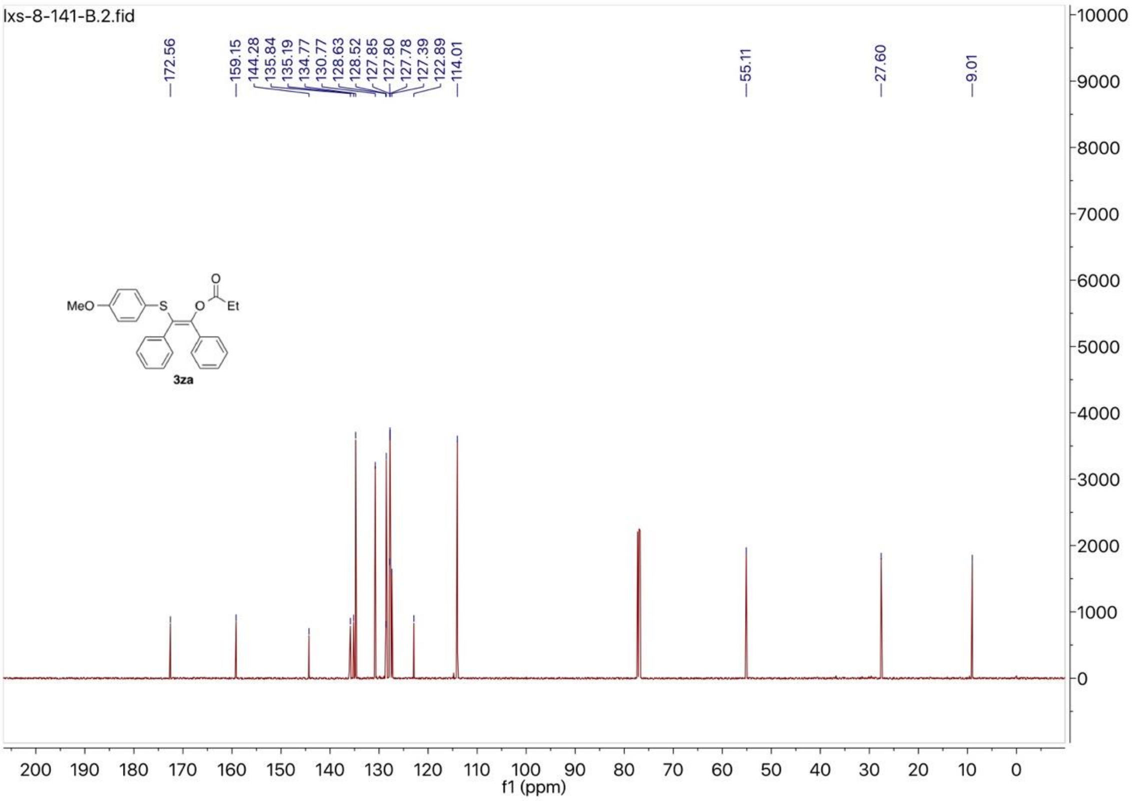

Supplementary Figure 168. ^{13}C NMR (125 MHz, $CDCl_3$) of compound **3za**

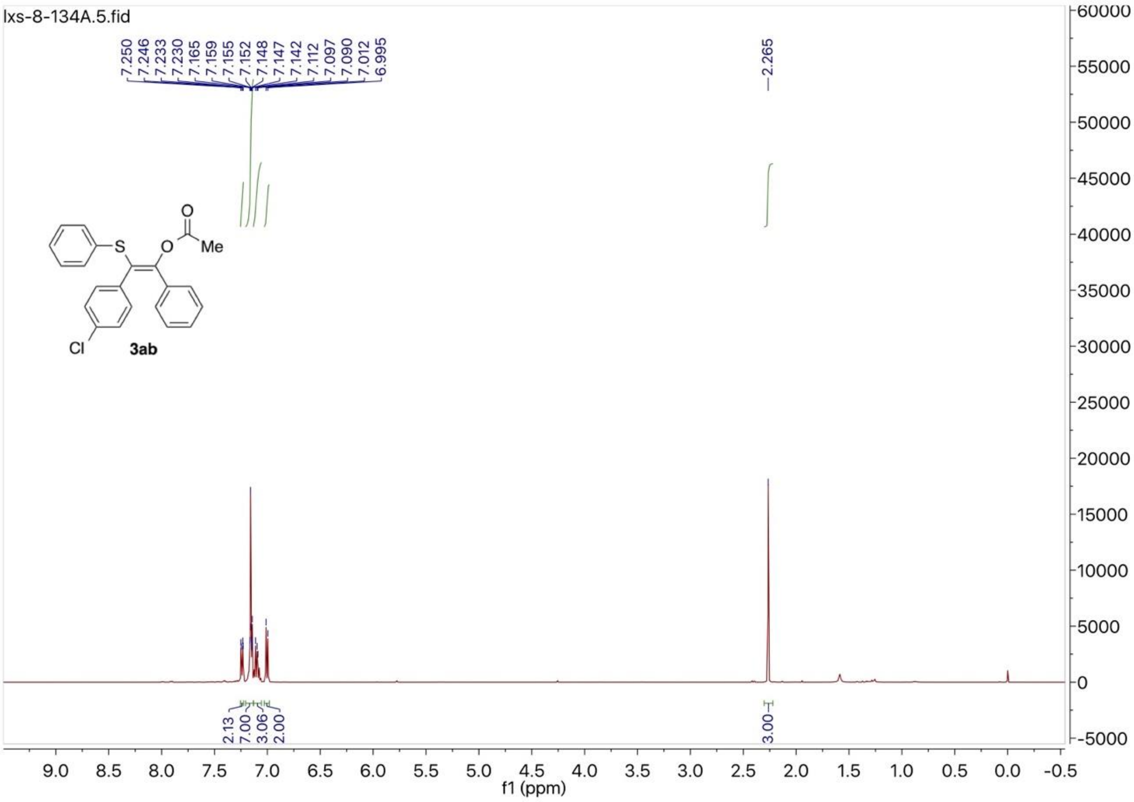

Supplementary Figure 169. ^{1}H NMR (500 MHz, $CDCl_3$) of compound **3ab**

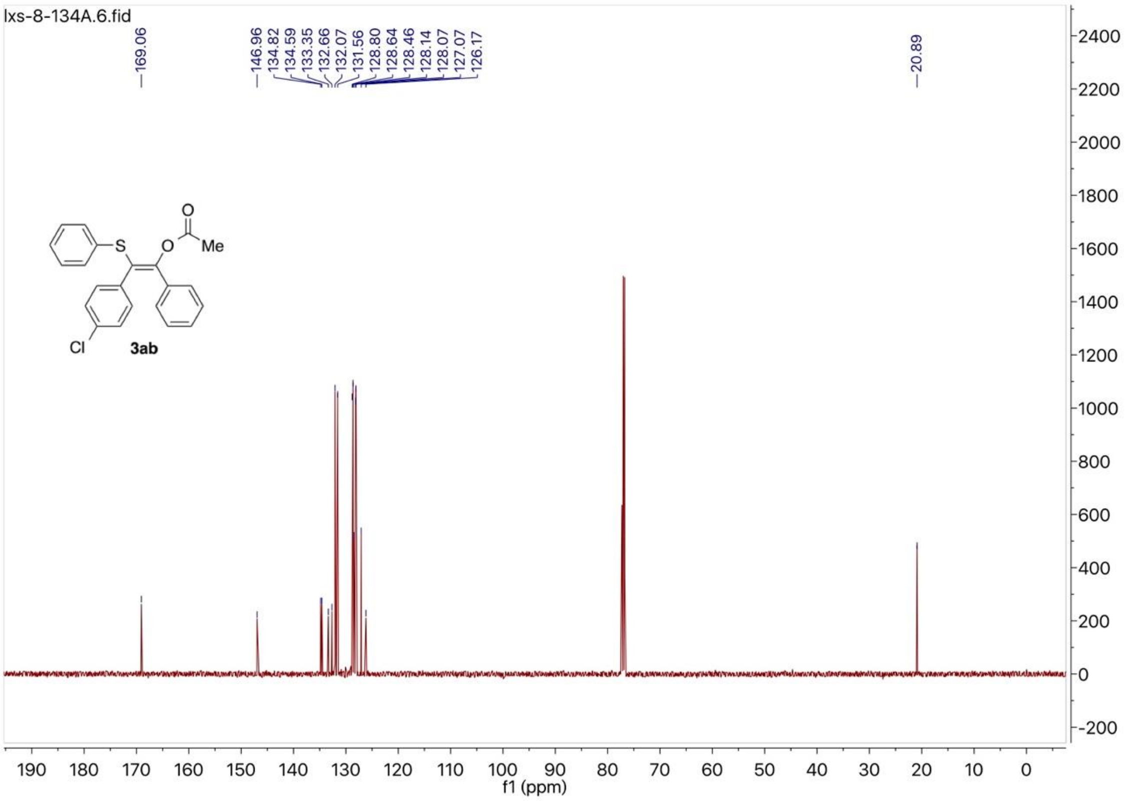

Supplementary Figure 170. ^{13}C NMR (125 MHz, $CDCl_3$) of compound **3ab**

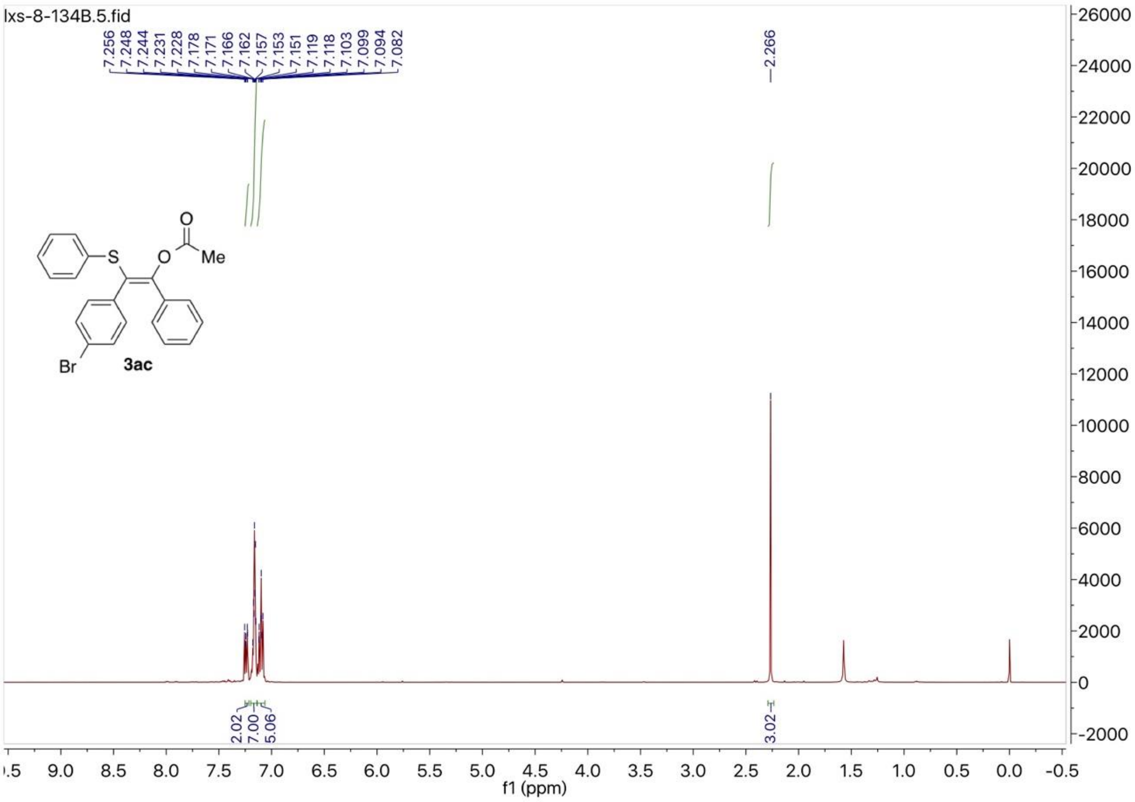

Supplementary Figure 171. ^{1}H NMR (500 MHz, $CDCl_3$) of compound **3ac**

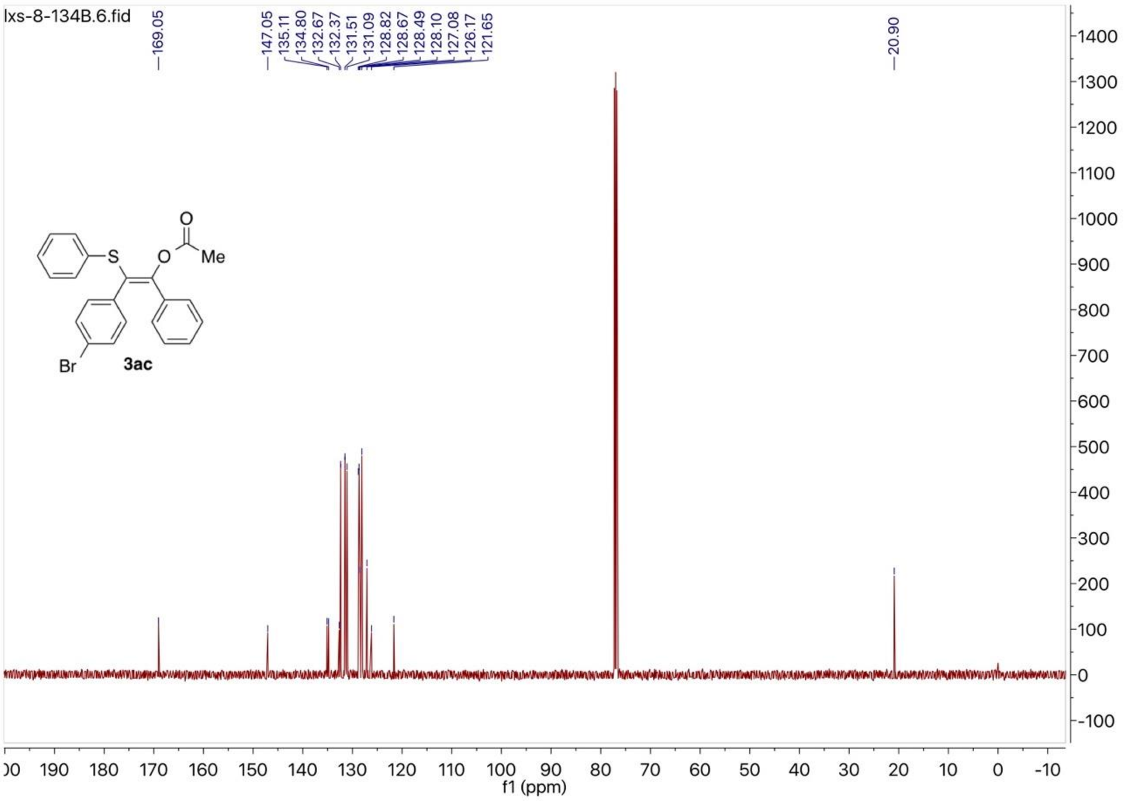

Supplementary Figure 172. ^{13}C NMR (125 MHz, $CDCl_3$) of compound **3ac**

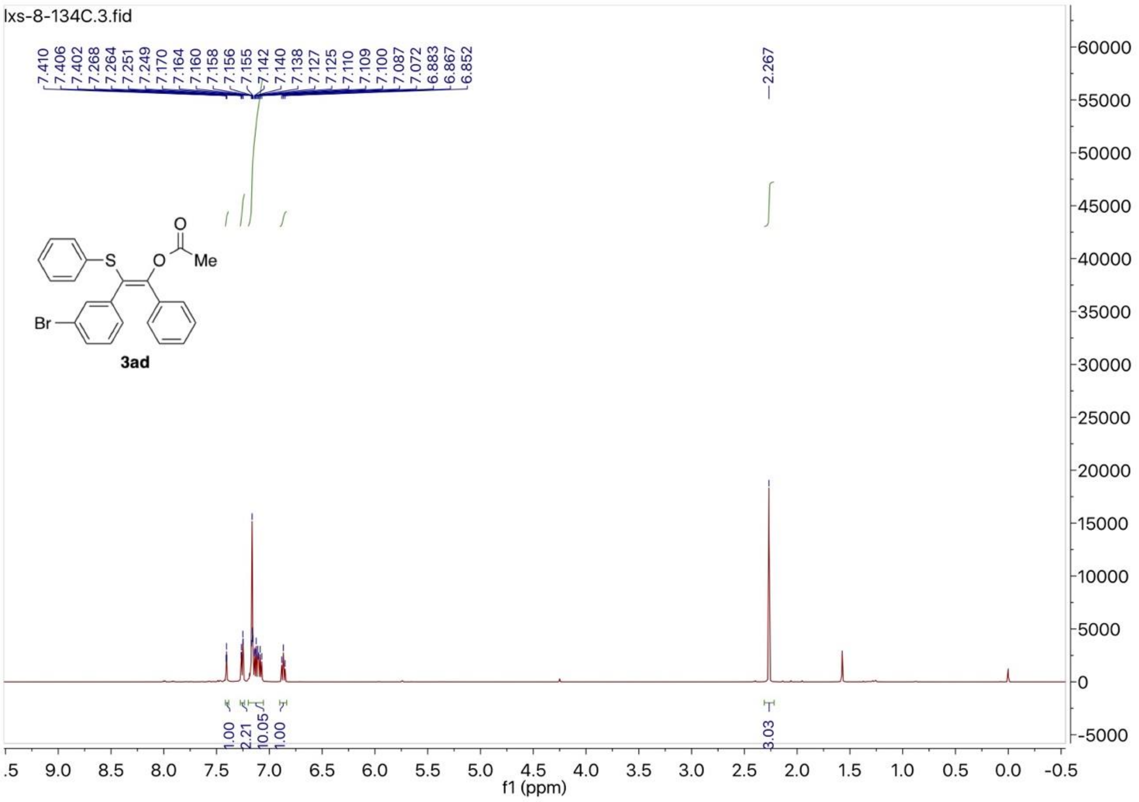

Supplementary Figure 173. ^{1}H NMR (500 MHz, $CDCl_3$) of compound **3ad**

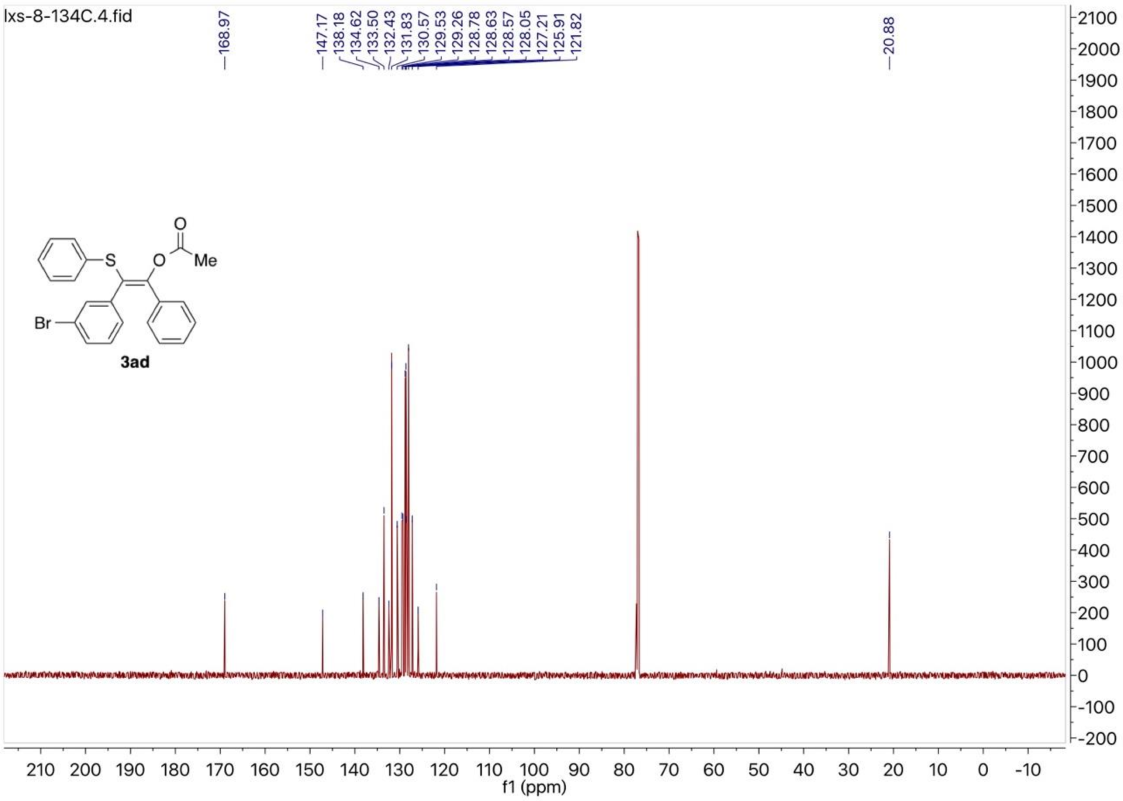

Supplementary Figure 174. ^{13}C NMR (125 MHz, $CDCl_3$) of compound **3ad**

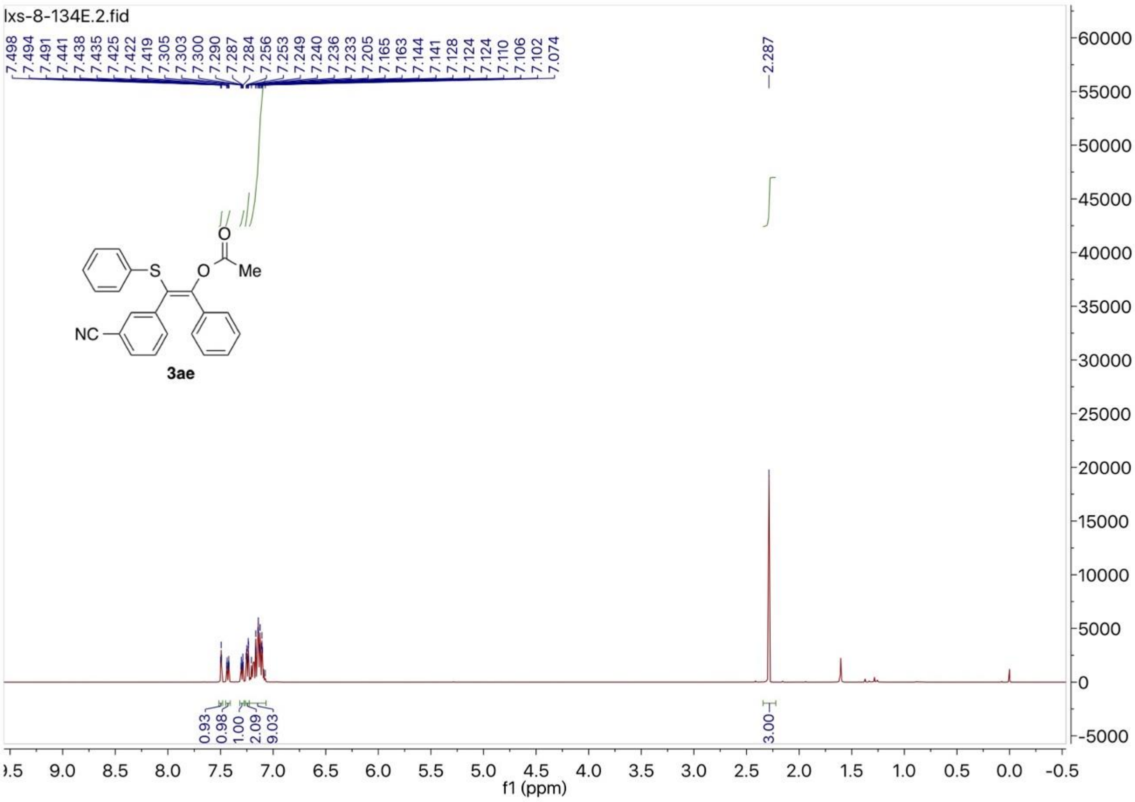

Supplementary Figure 175. ^{1}H NMR (500 MHz, $CDCl_3$) of compound **3ae**

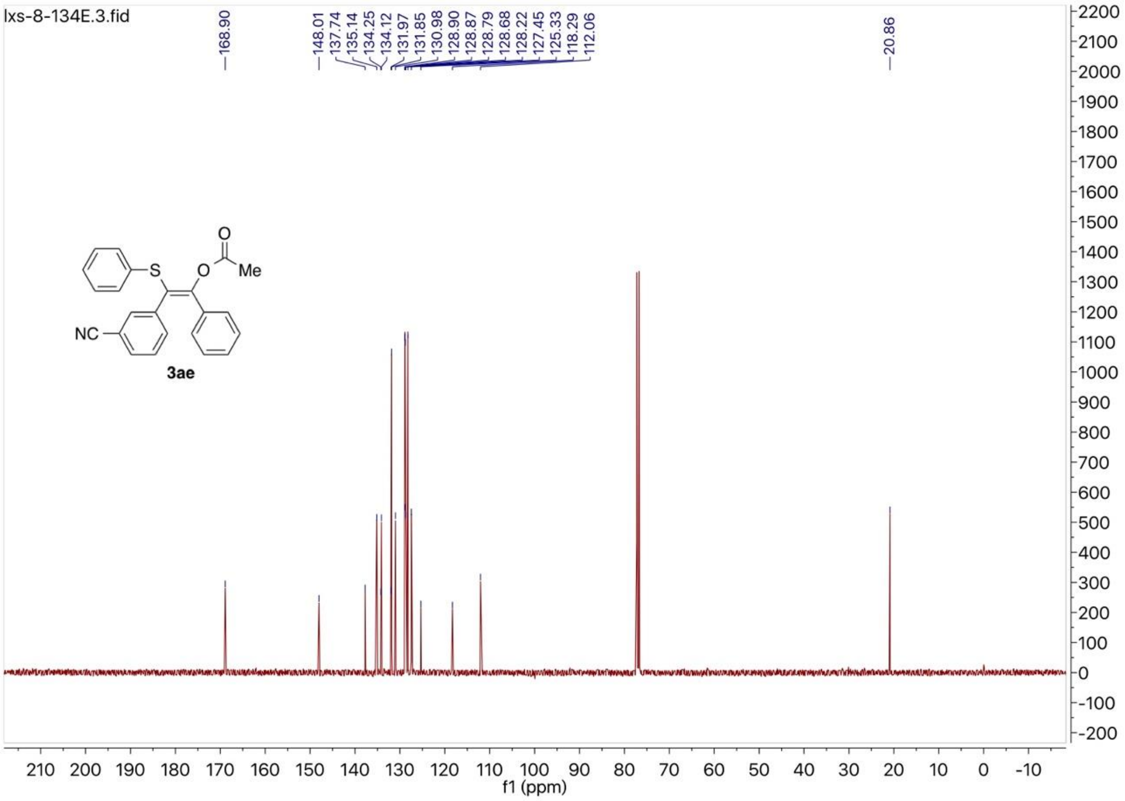

Supplementary Figure 176. ^{13}C NMR (125 MHz, $CDCl_3$) of compound **3ae**

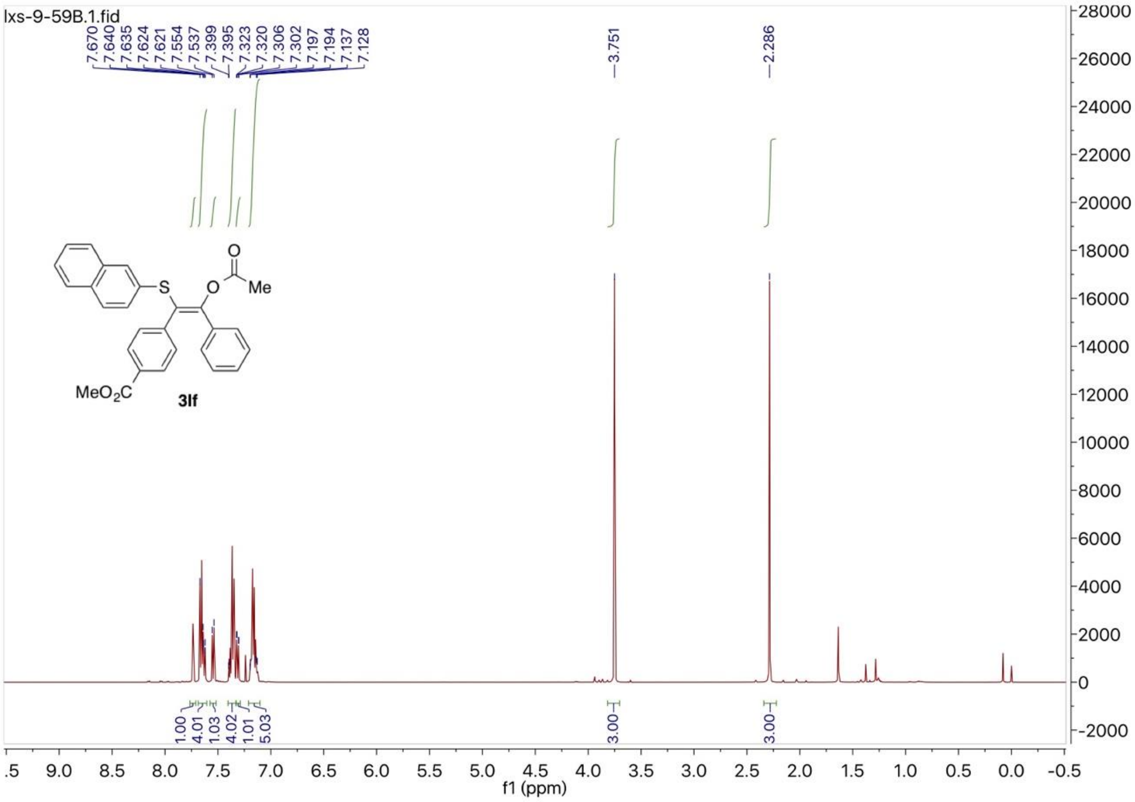

Supplementary Figure 177. ^{1}H NMR (500 MHz, $CDCl_3$) of compound **3lf**

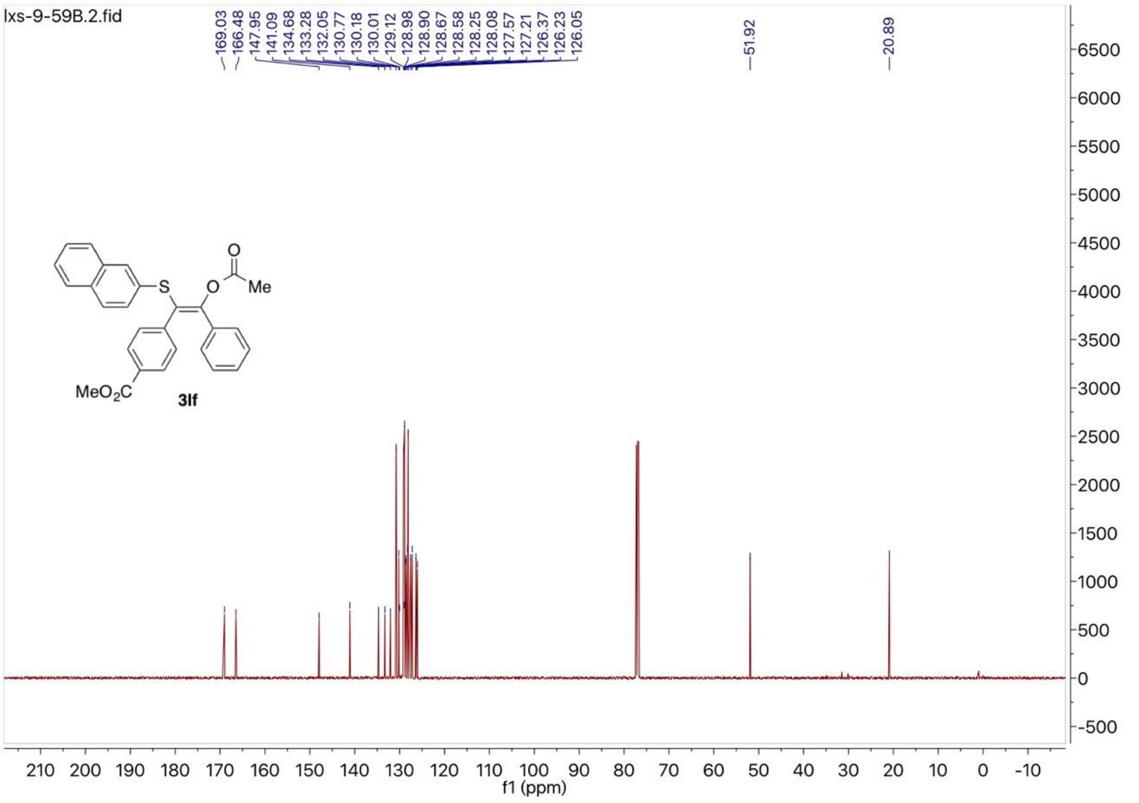

Supplementary Figure 178. ^{13}C NMR (125 MHz, $CDCl_3$) of compound **3lf**

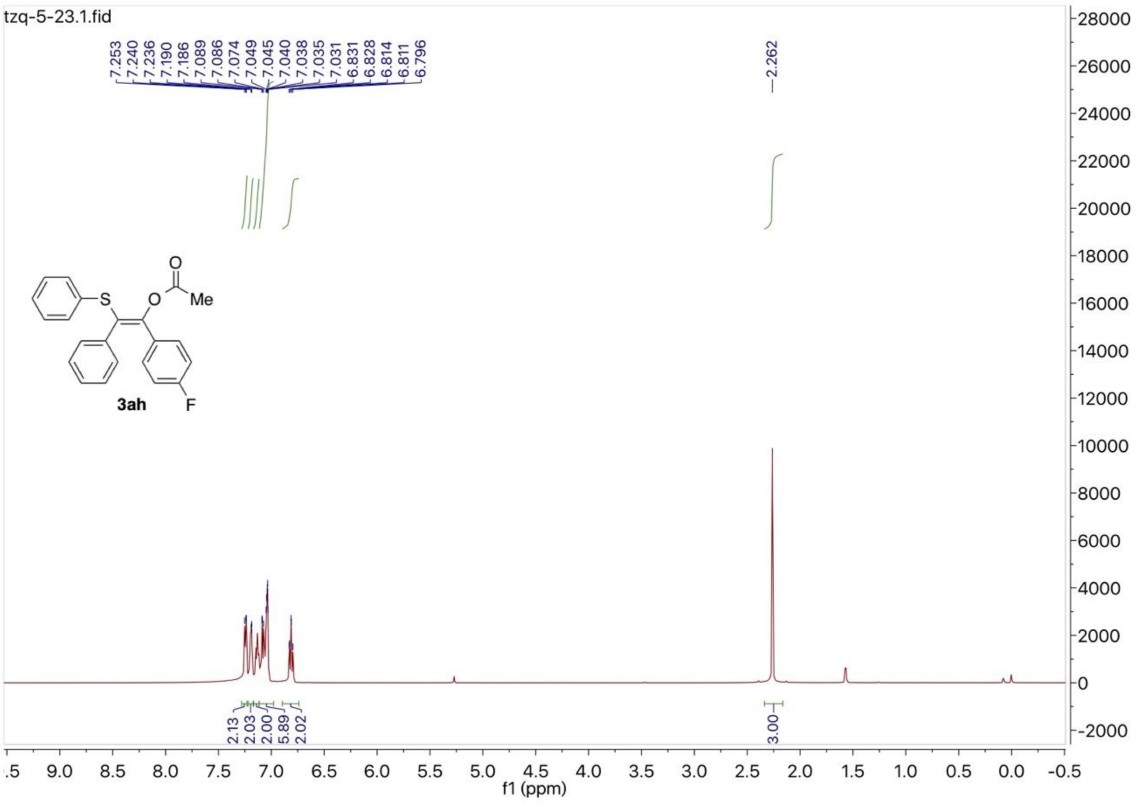

Supplementary Figure 179. ^{1}H NMR (500 MHz, $CDCl_3$) of compound **3ah**

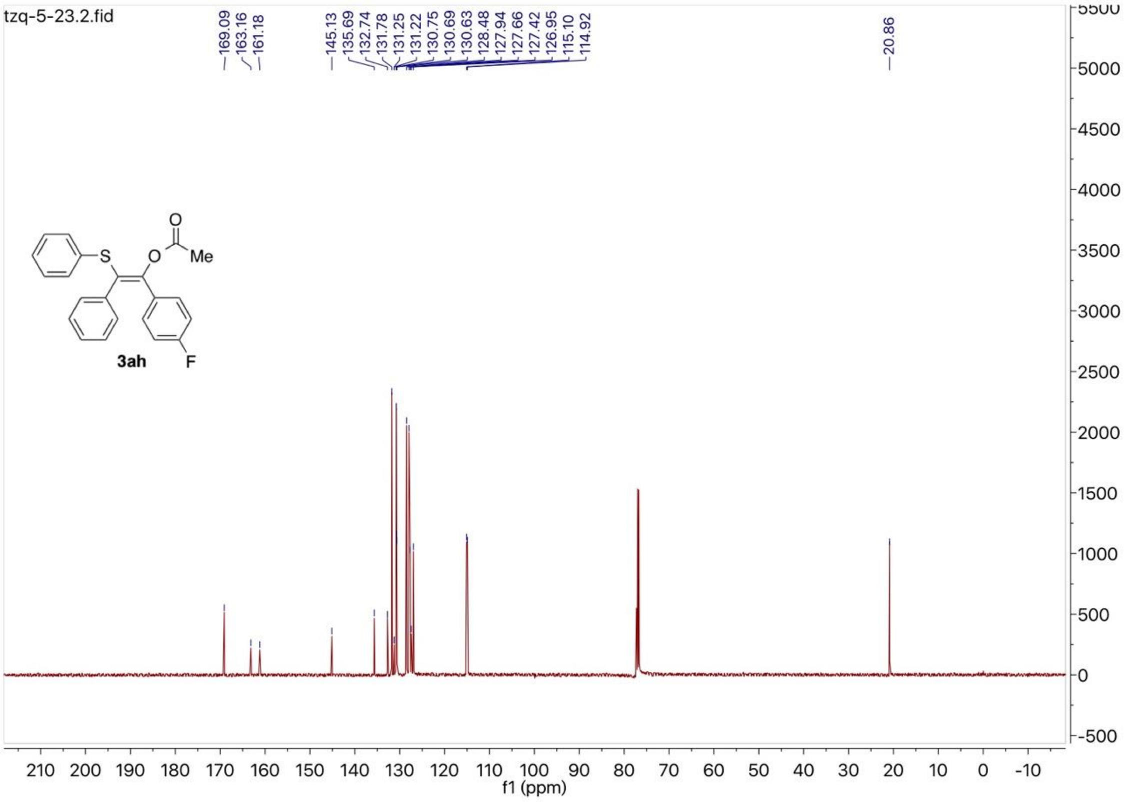

Supplementary Figure 180. ^{13}C NMR (125 MHz, $CDCl_3$) of compound **3ah**

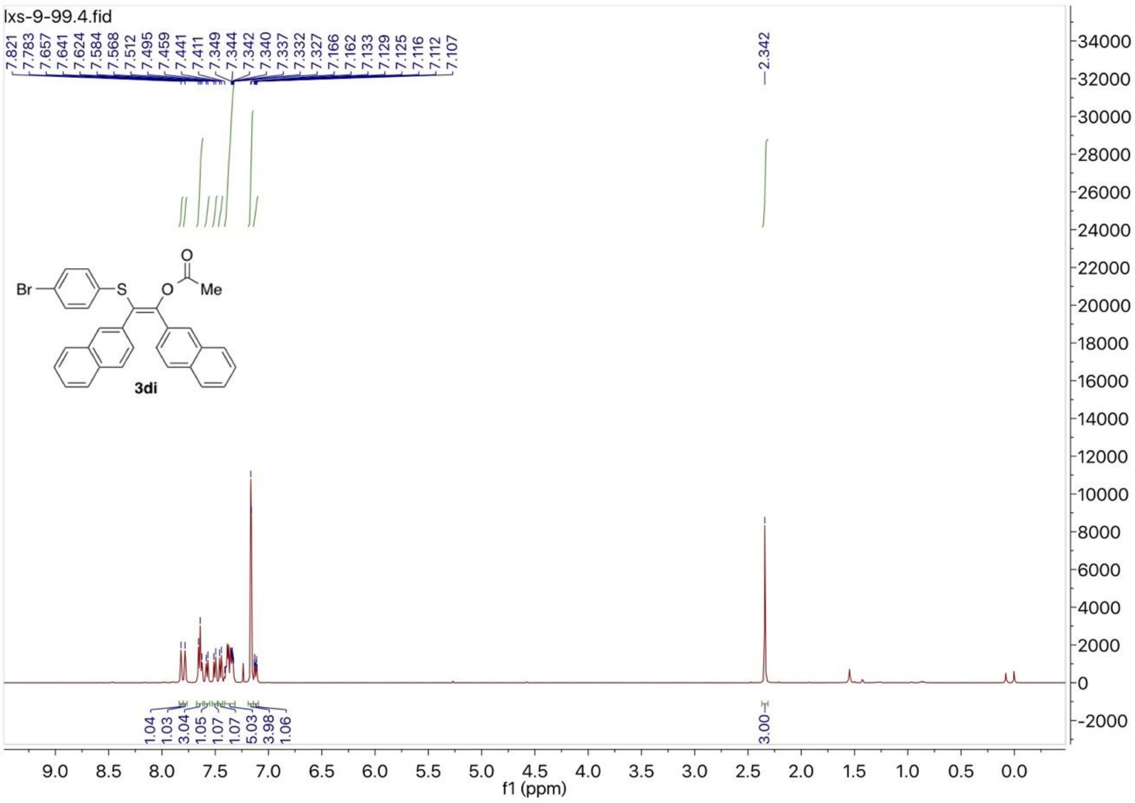

Supplementary Figure 181. ^{1}H NMR (500 MHz, $CDCl_3$) of compound **3di**

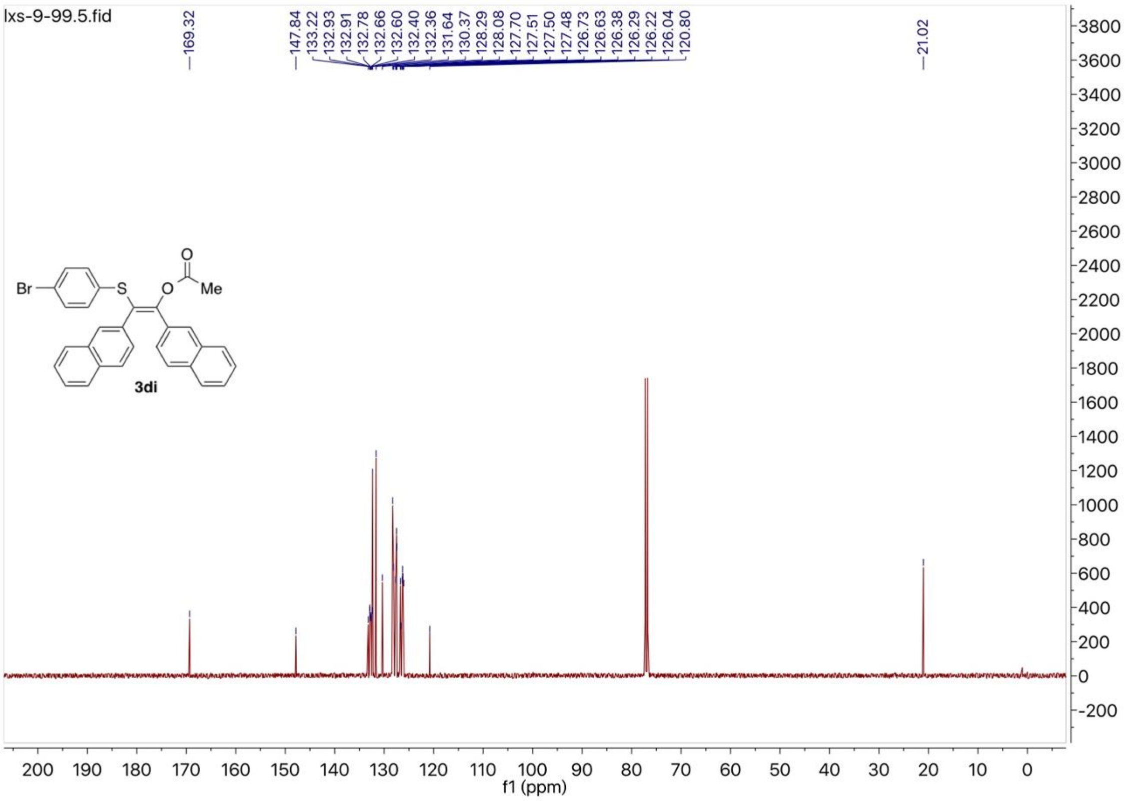

Supplementary Figure 182. ^{13}C NMR (125 MHz, $CDCl_3$) of compound **3di**

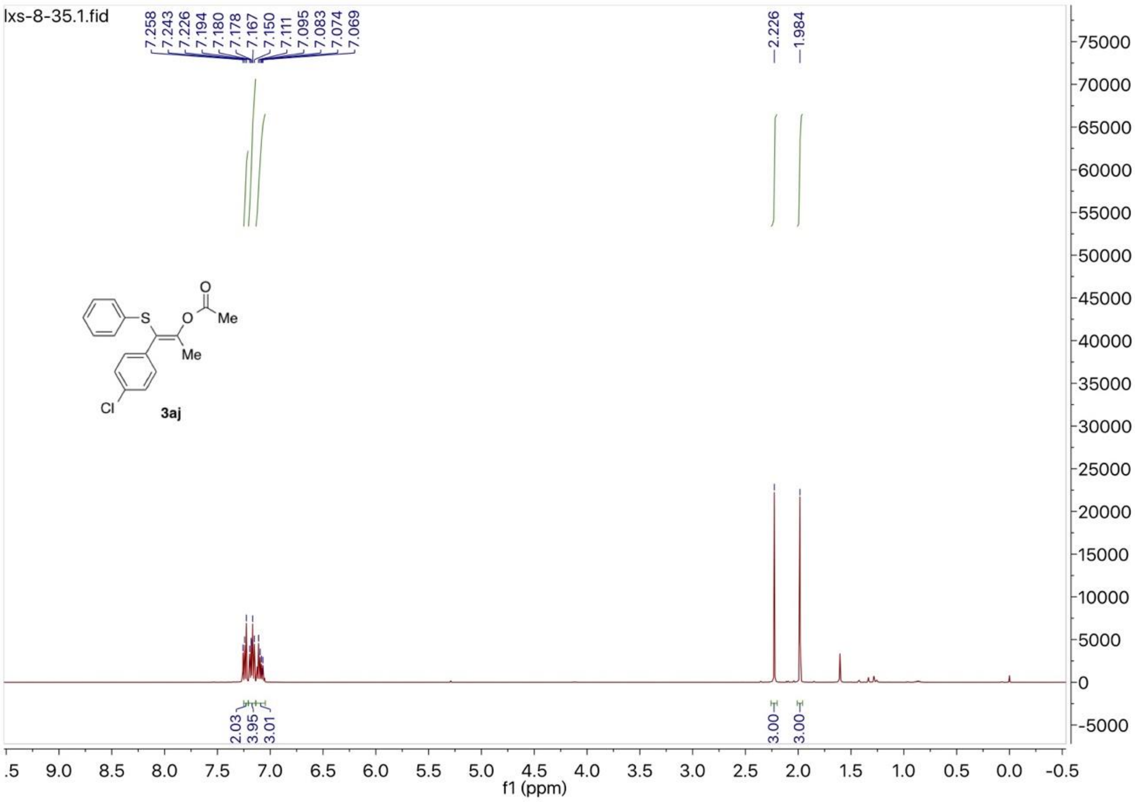

Supplementary Figure 183. ^{1}H NMR (500 MHz, $CDCl_3$) of compound **3aj**

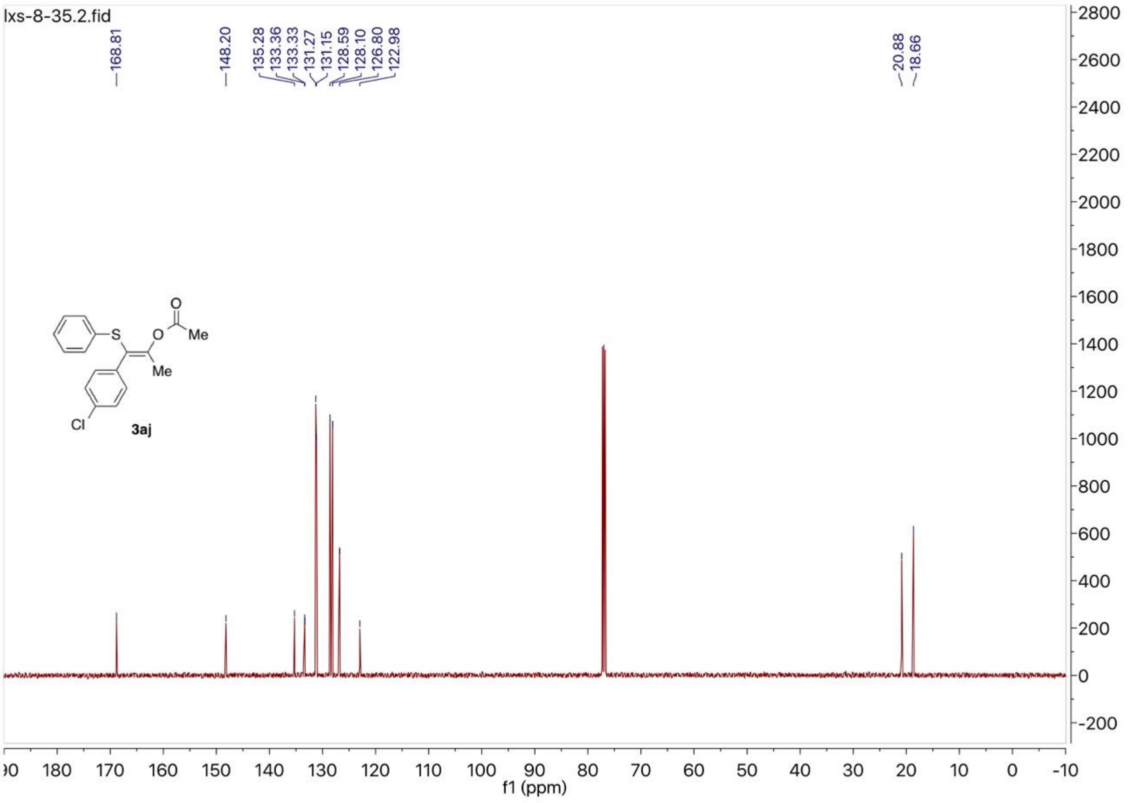

Supplementary Figure 184. ^{13}C NMR (125 MHz, $CDCl_3$) of compound **3aj**

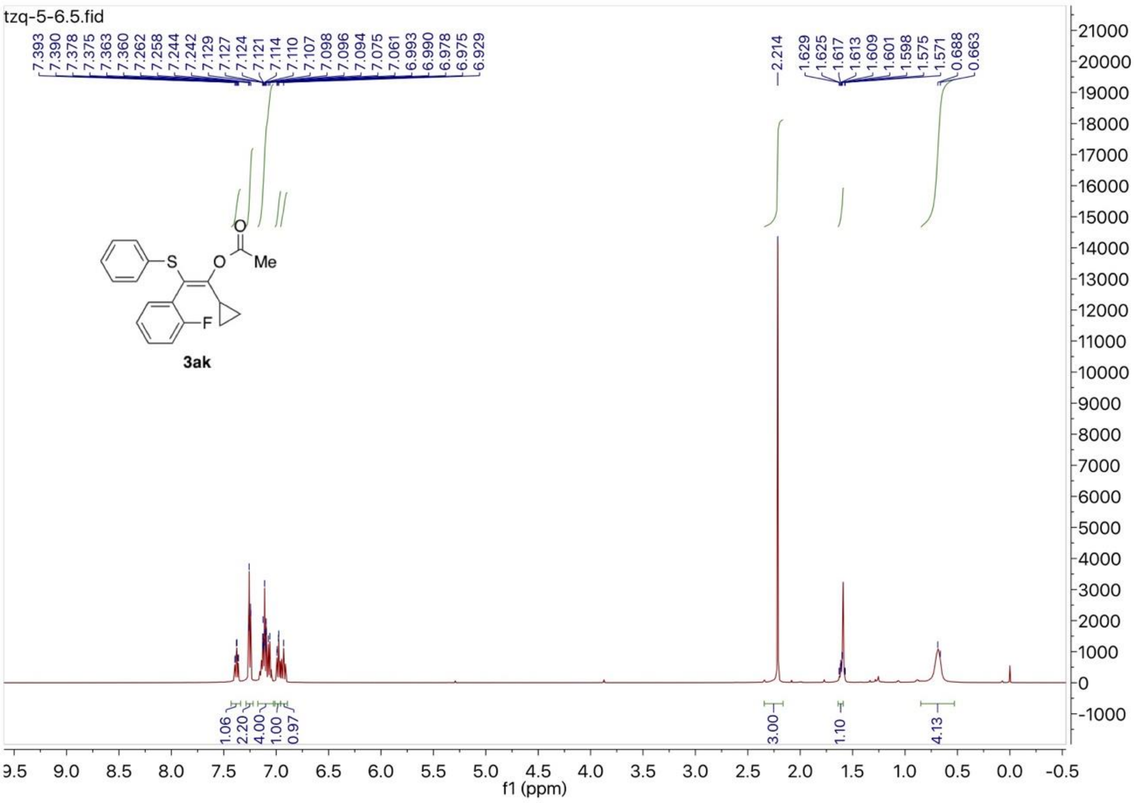

Supplementary Figure 185. ^{1}H NMR (500 MHz, $CDCl_3$) of compound **3ak**

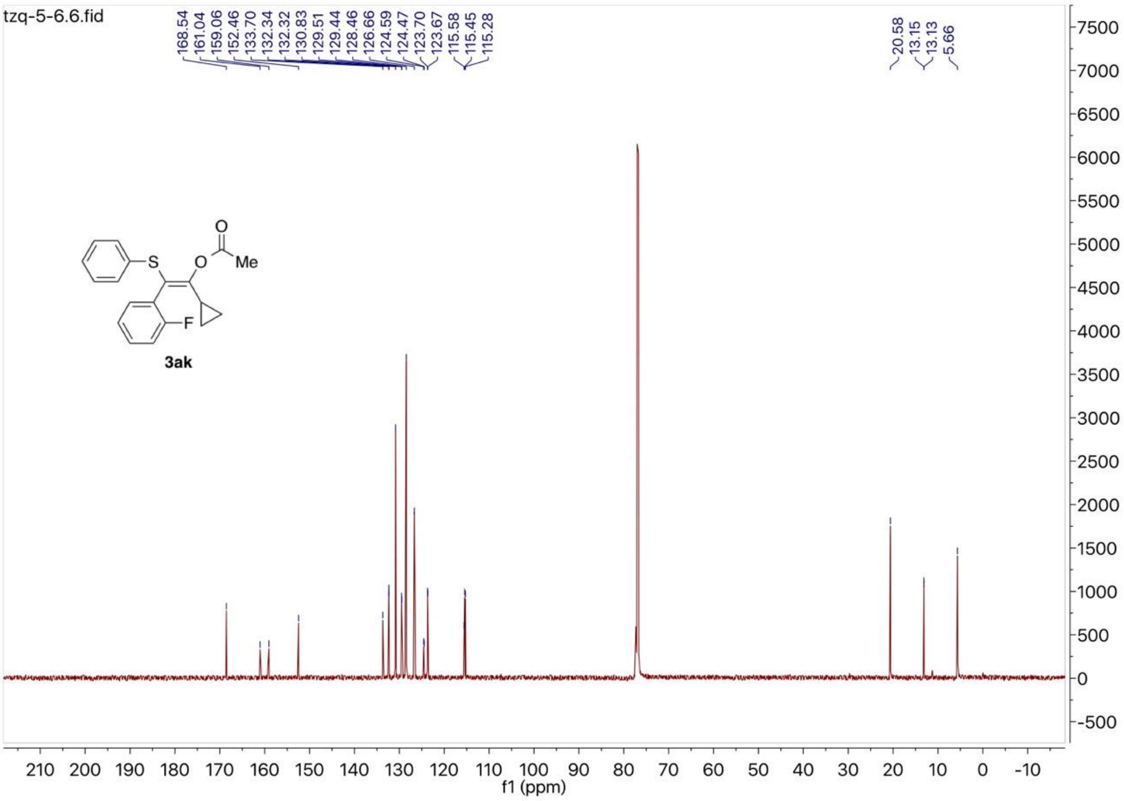

Supplementary Figure 186. ^{13}C NMR (125 MHz, $CDCl_3$) of compound **3ak**

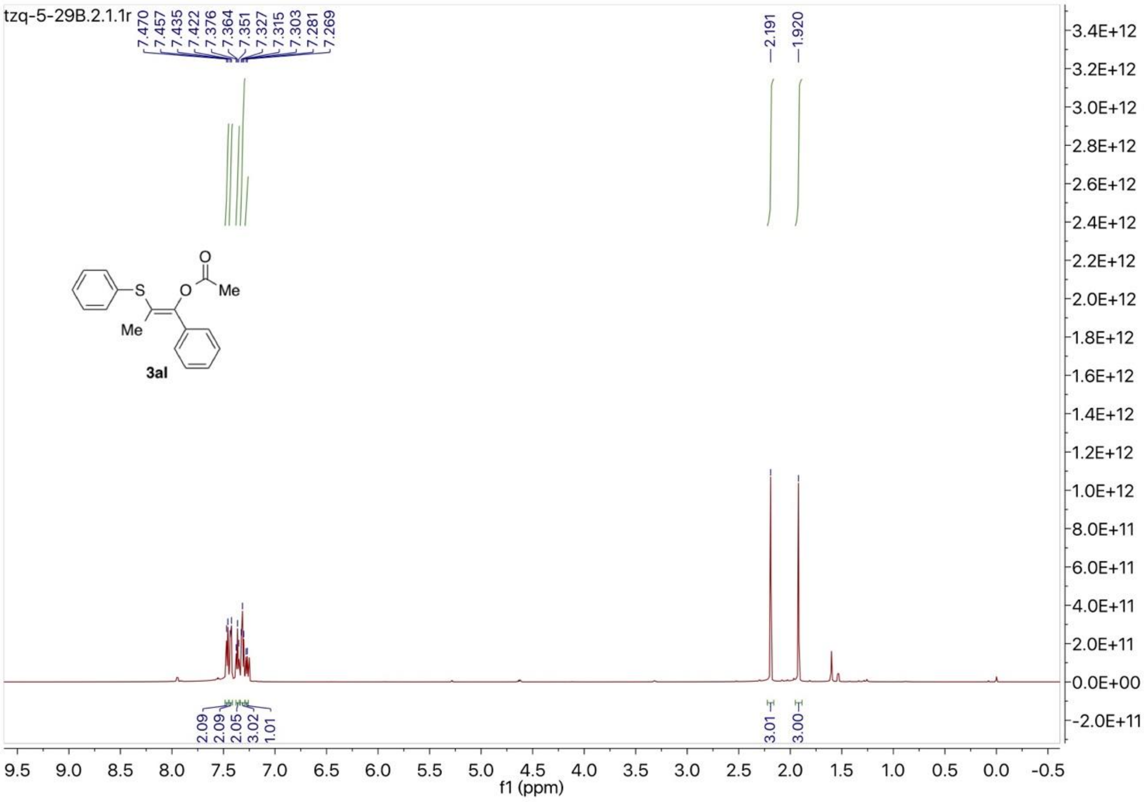

Supplementary Figure 187. ^{1}H NMR (600 MHz, $CDCl_3$) of compound **3al**

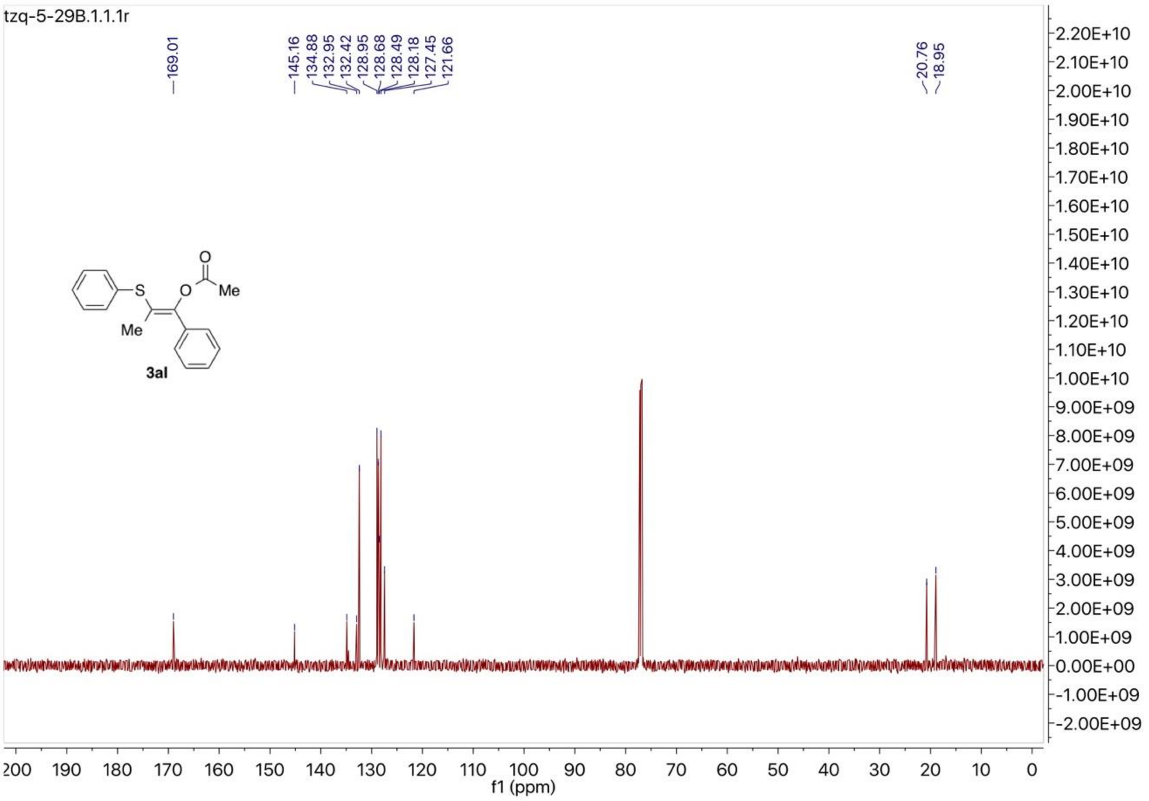

Supplementary Figure 188. ^{13}C NMR (150 MHz, $CDCl_3$) of compound **3al**

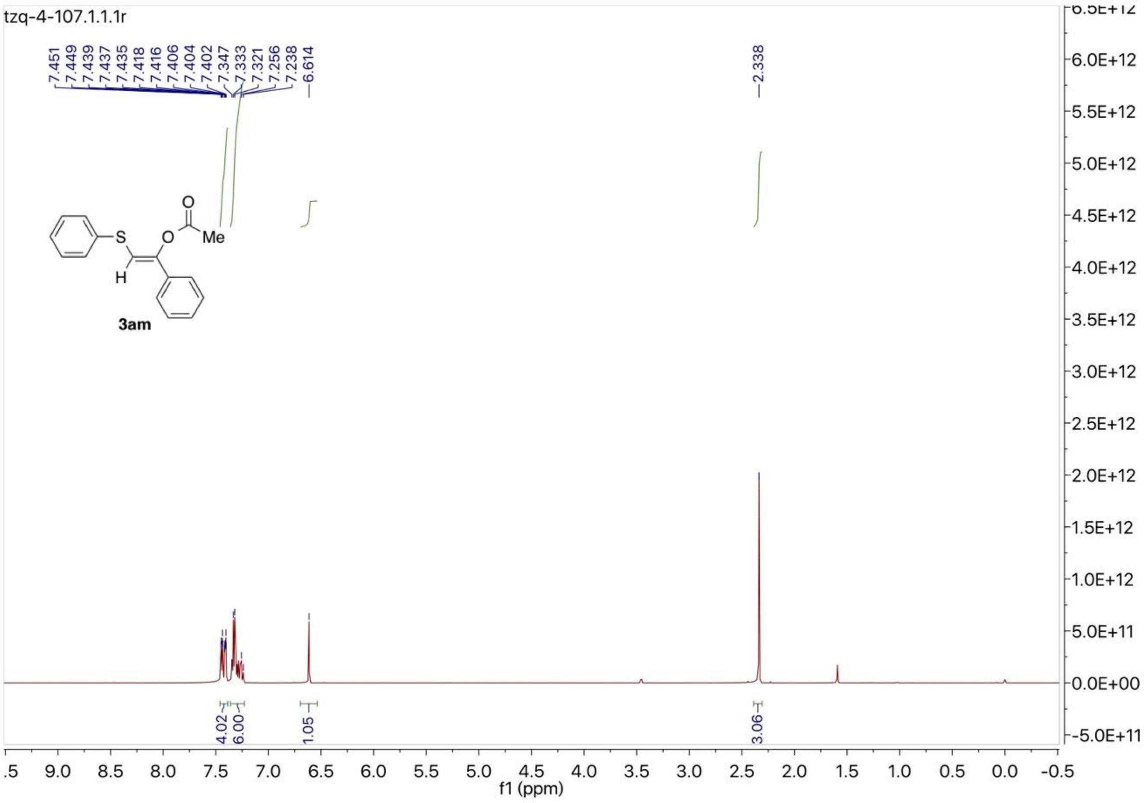

Supplementary Figure 189. ^{1}H NMR (600 MHz, $CDCl_3$) of compound **3am**

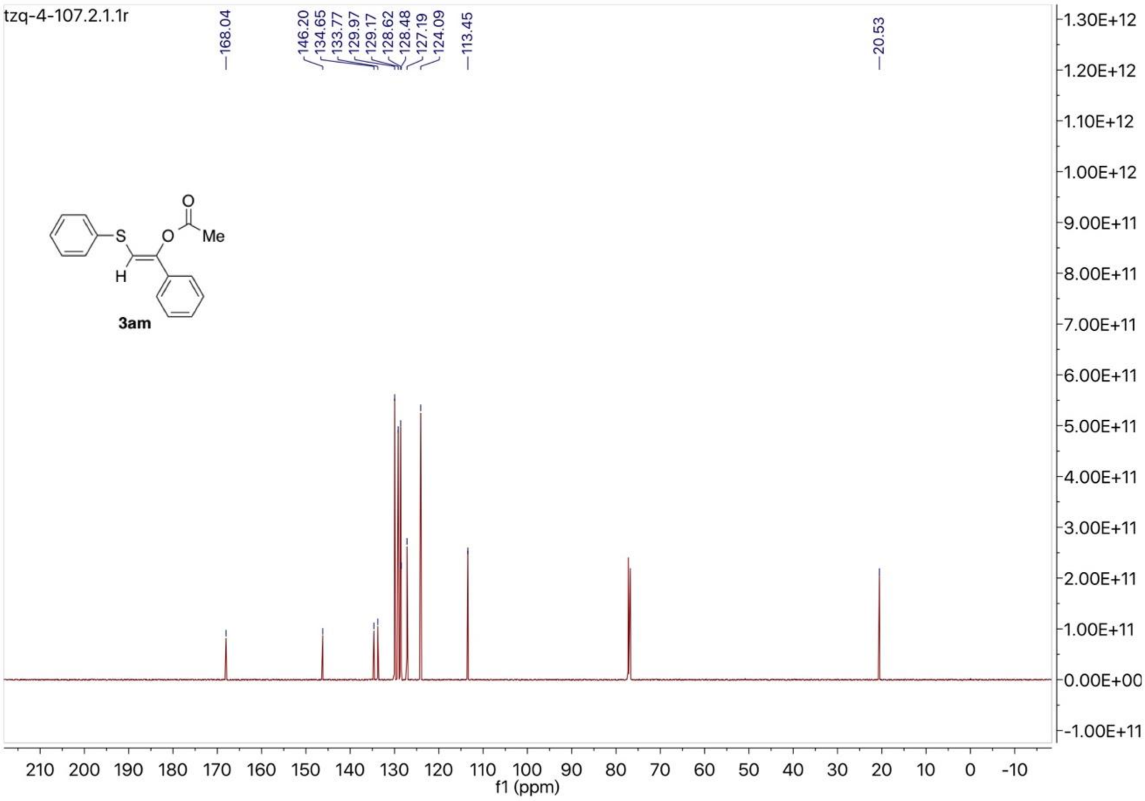

Supplementary Figure 190. ^{13}C NMR (150 MHz, $CDCl_3$) of compound **3am**

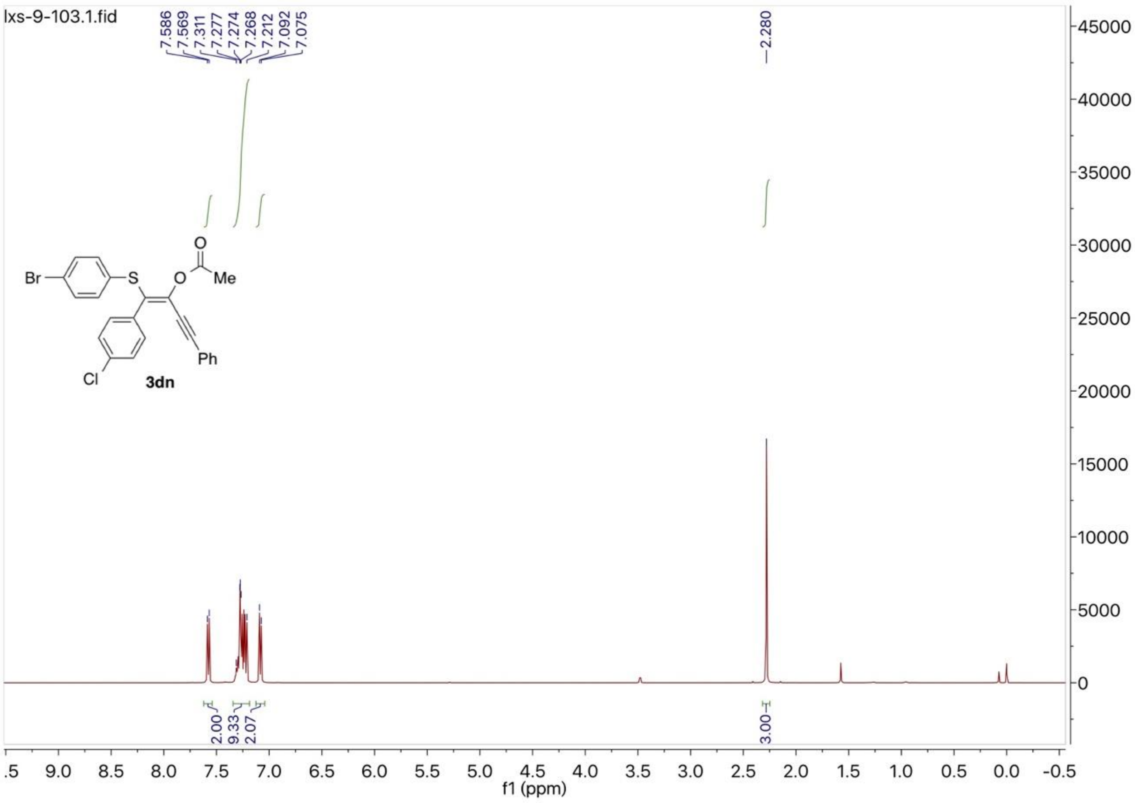

Supplementary Figure 191. ^{1}H NMR (500 MHz, $CDCl_3$) of compound **3dn**

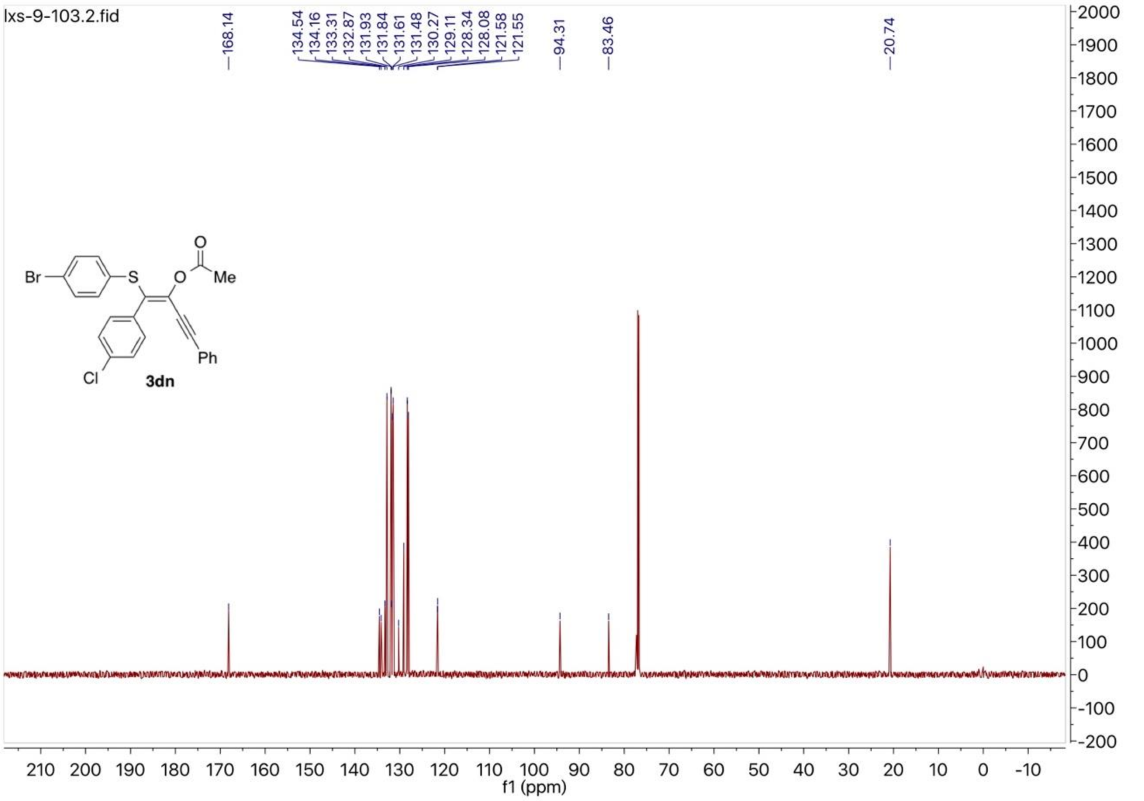

Supplementary Figure 192. ^{13}C NMR (125 MHz, $CDCl_3$) of compound **3dn**

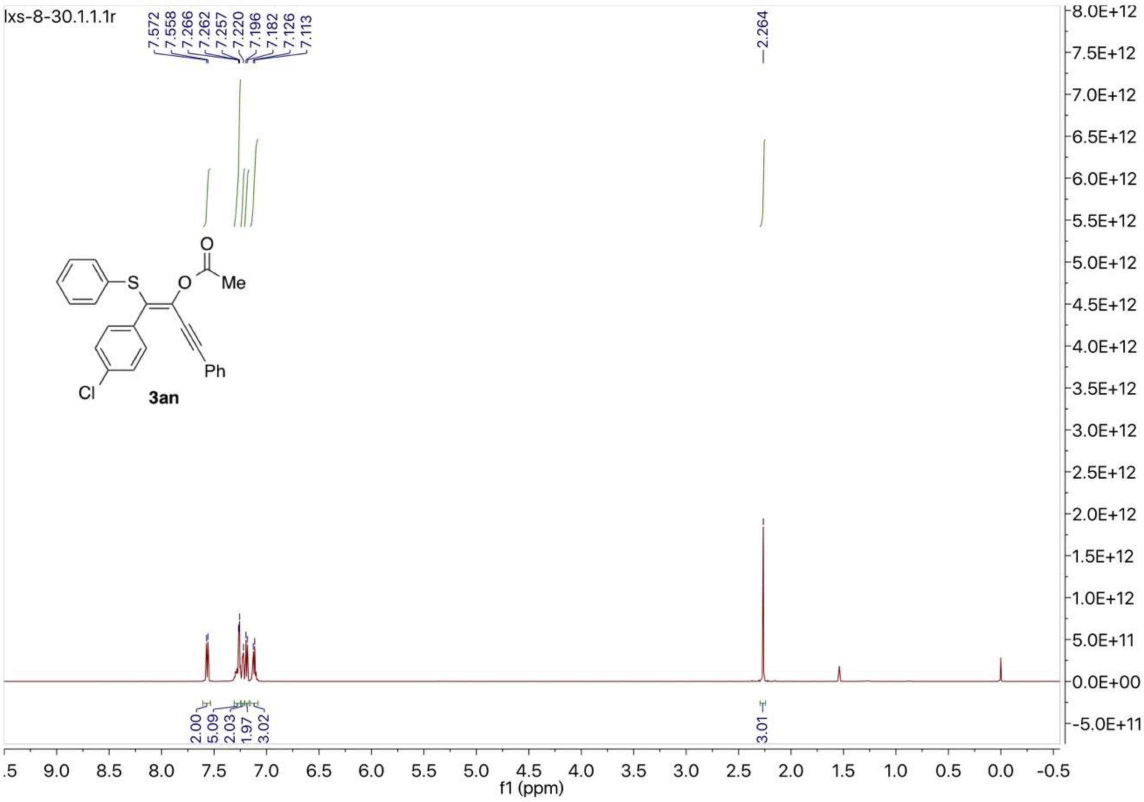

Supplementary Figure 193. ^{1}H NMR (500 MHz, $CDCl_3$) of compound **3an**

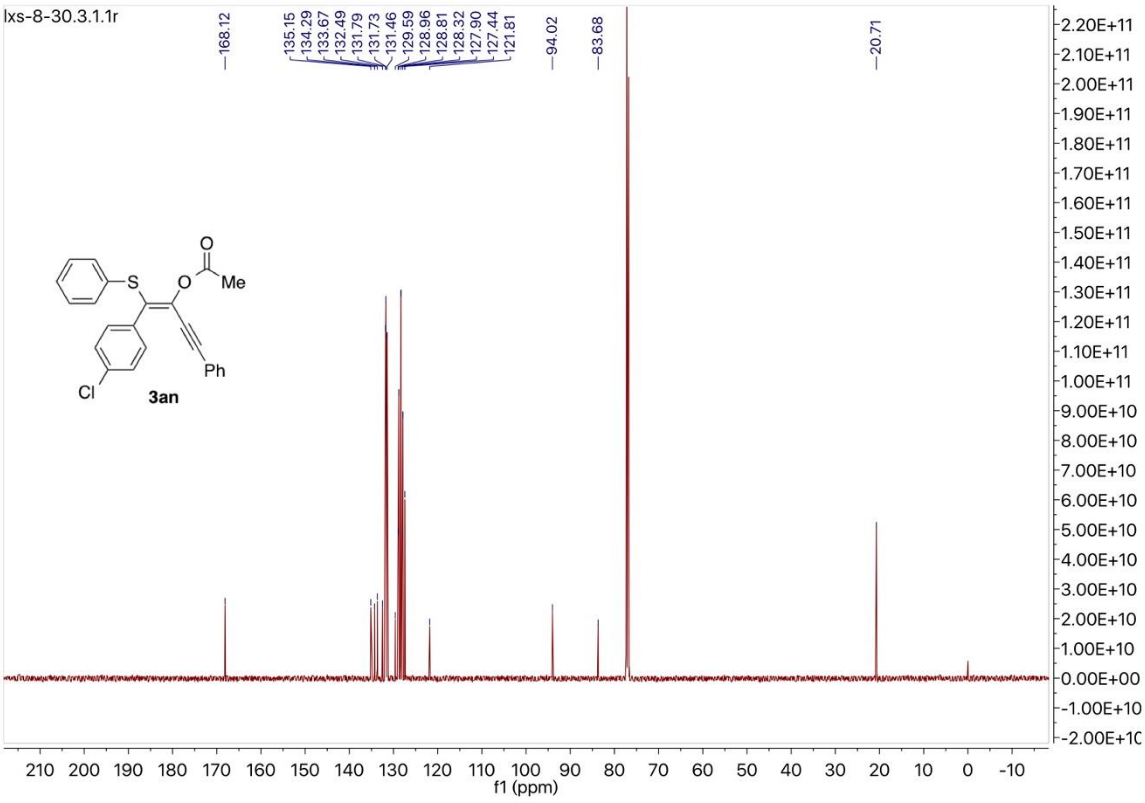

Supplementary Figure 194. ^{13}C NMR (125 MHz, $CDCl_3$) of compound **3an**

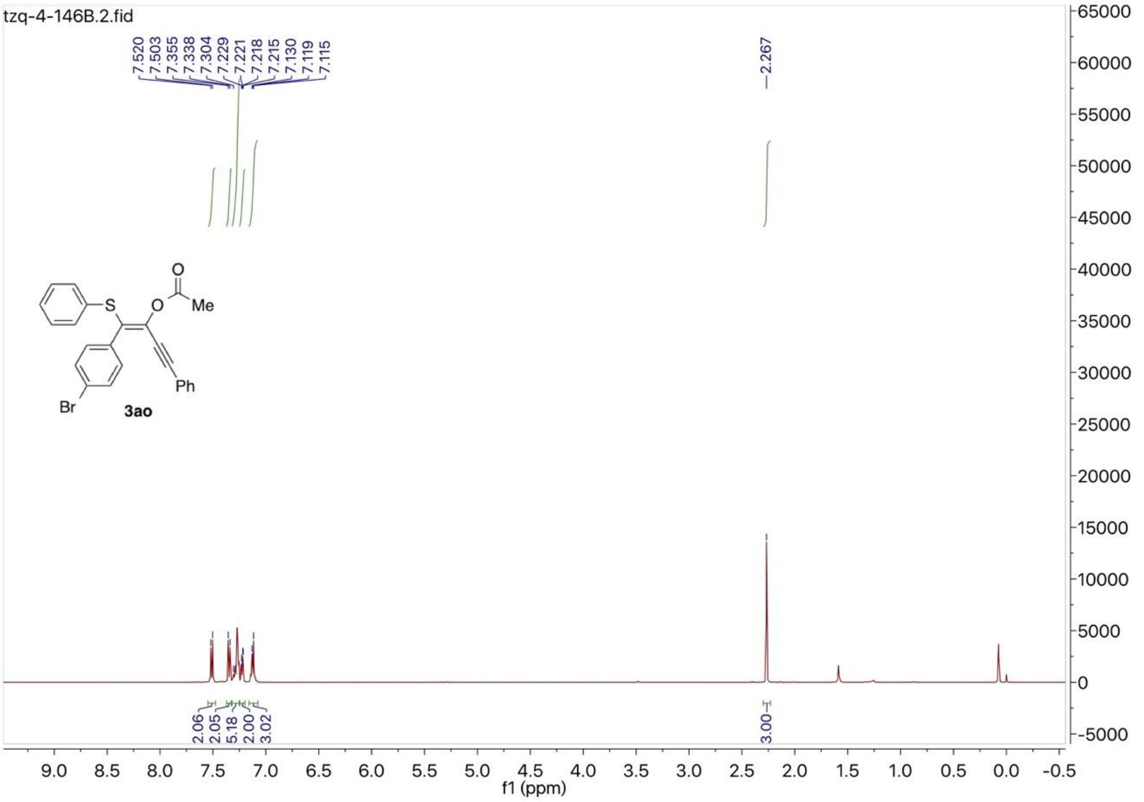

Supplementary Figure 195. ^{1}H NMR (500 MHz, $CDCl_3$) of compound **3ao**

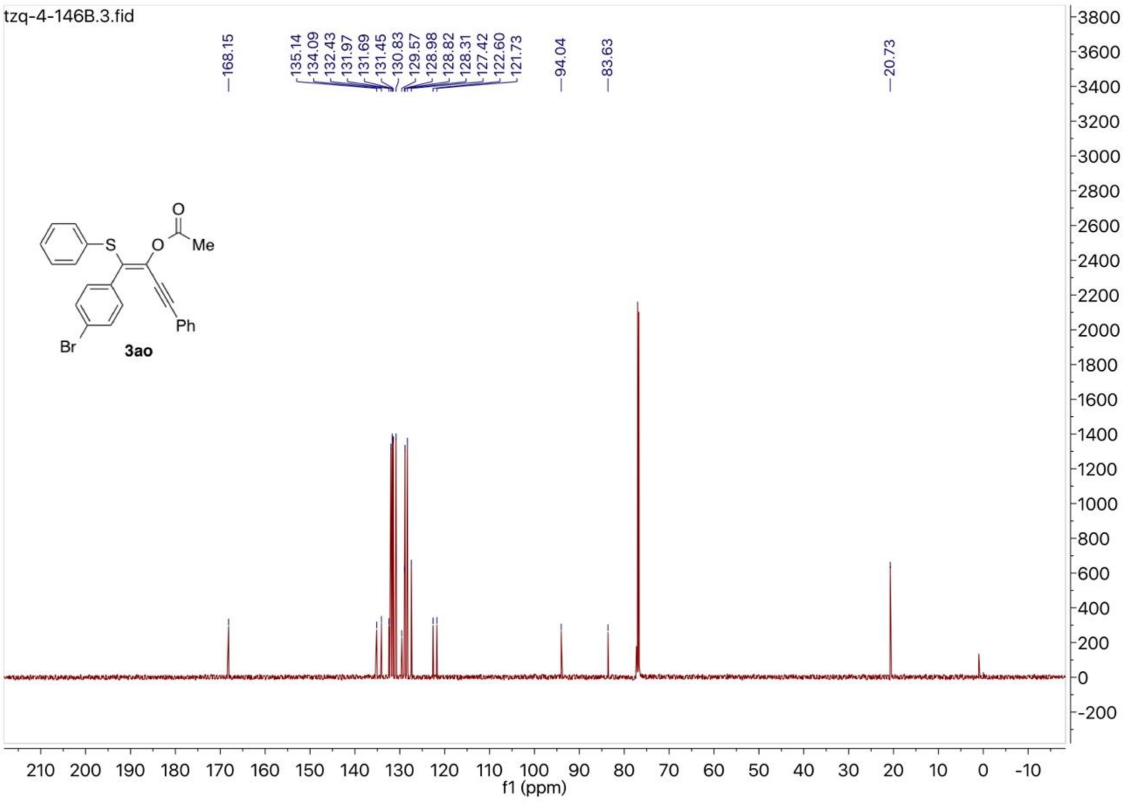

Supplementary Figure 196. ^{13}C NMR (125 MHz, $CDCl_3$) of compound **3ao**

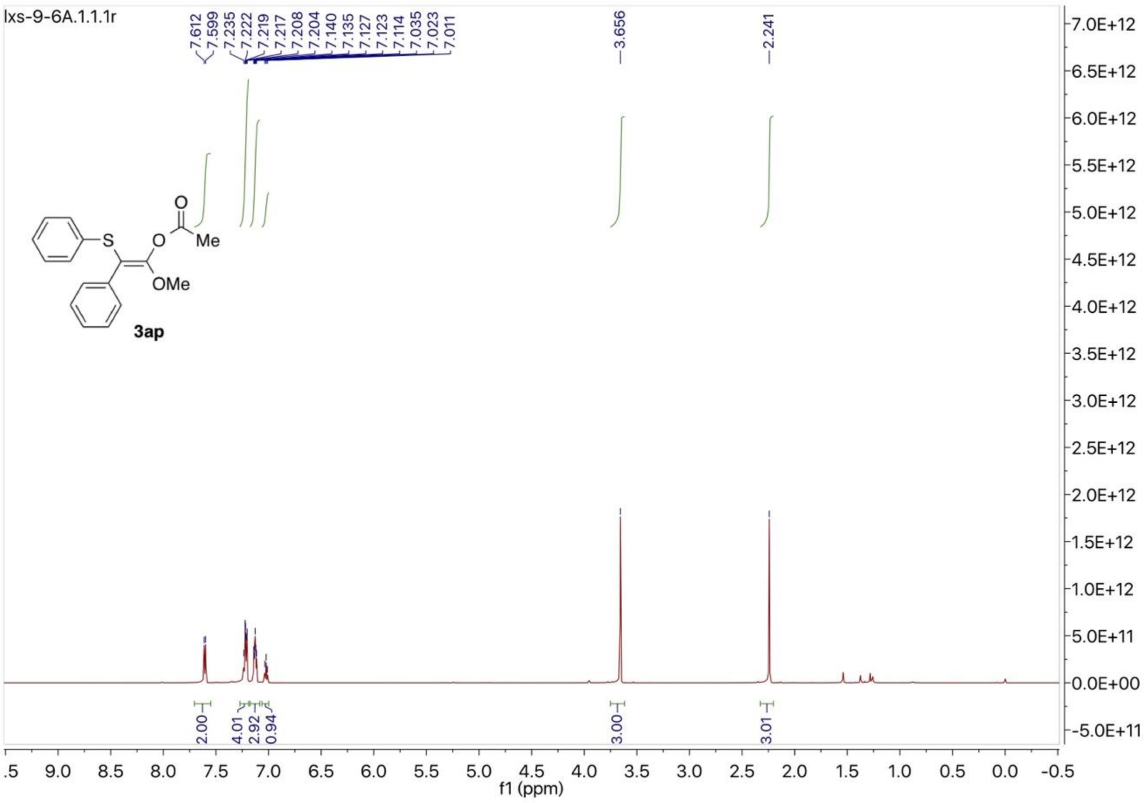

Supplementary Figure 197. ^{1}H NMR (600 MHz, $CDCl_3$) of compound **3ap**

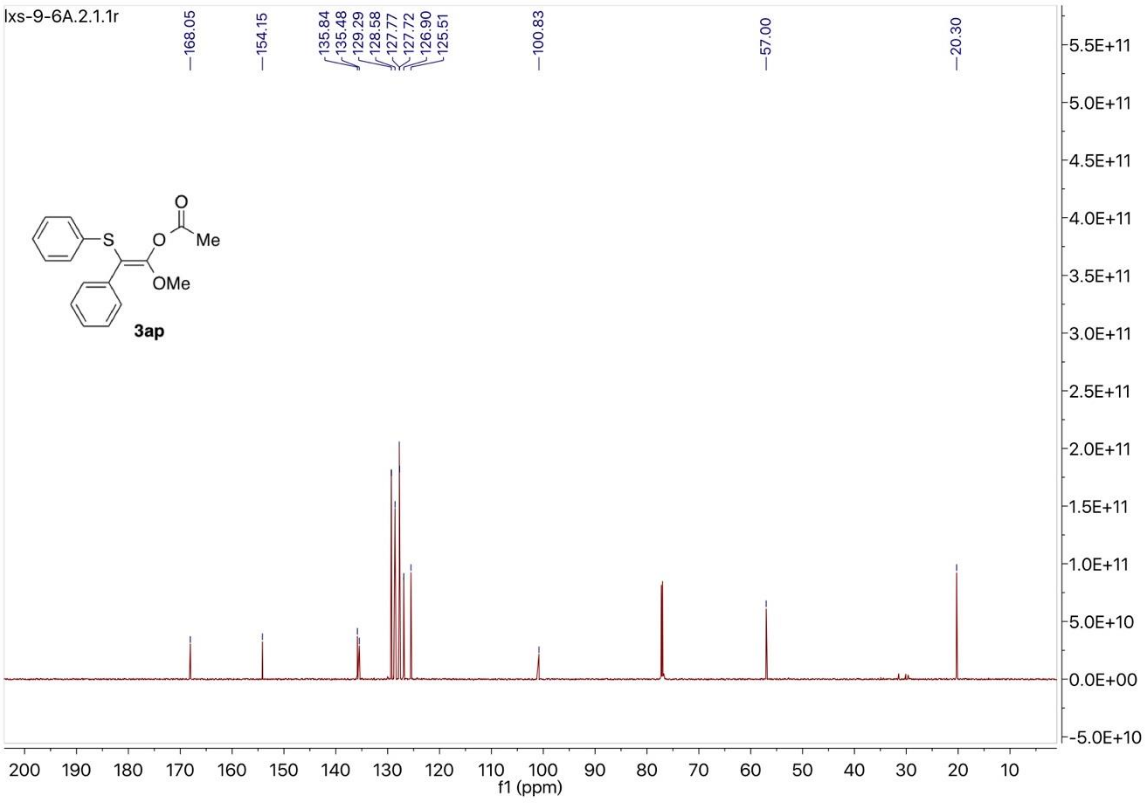

Supplementary Figure 198. ^{13}C NMR (150 MHz, $CDCl_3$) of compound **3ap**

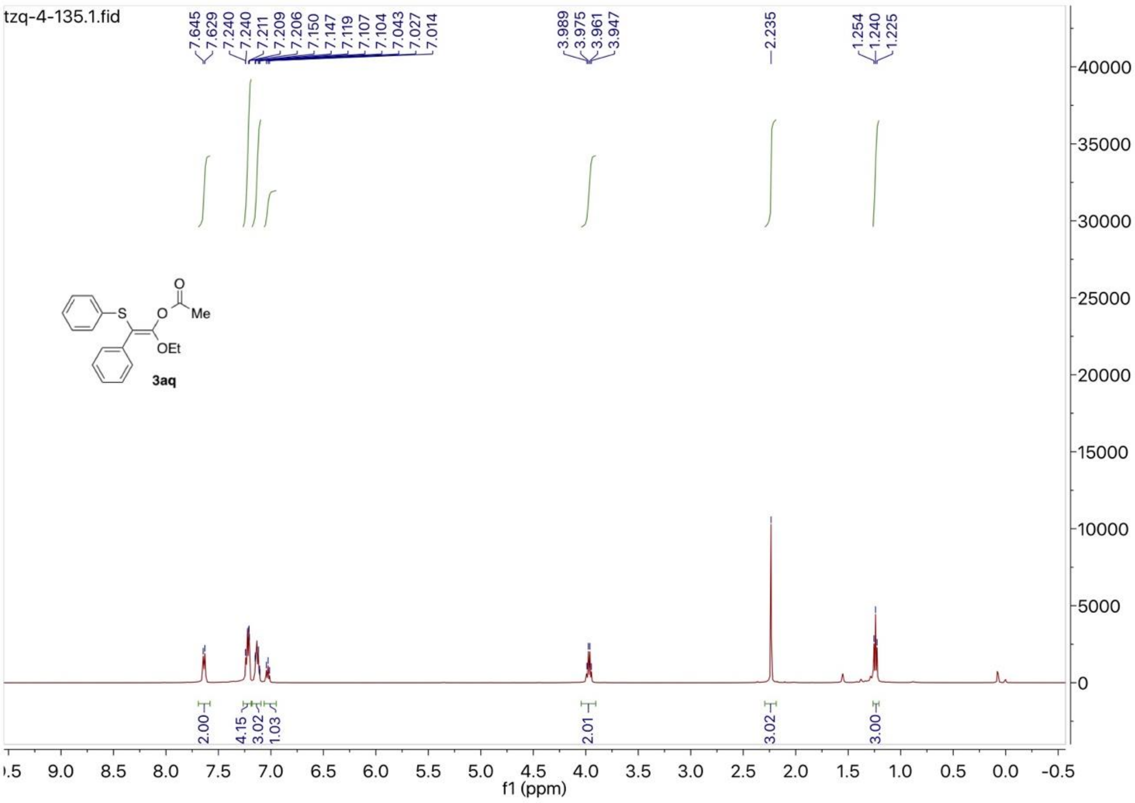

Supplementary Figure 199. ^{1}H NMR (500 MHz, $CDCl_3$) of compound **3aq**

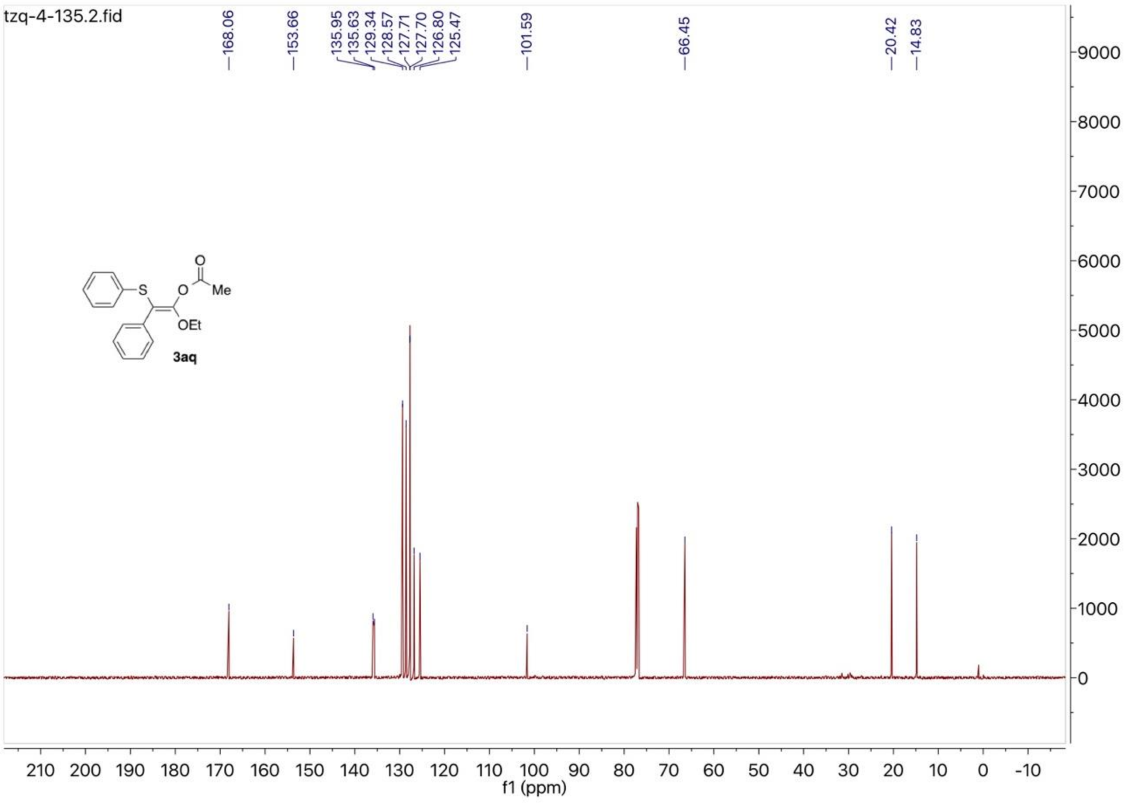

Supplementary Figure 200. ^{13}C NMR (125 MHz, $CDCl_3$) of compound **3aq**

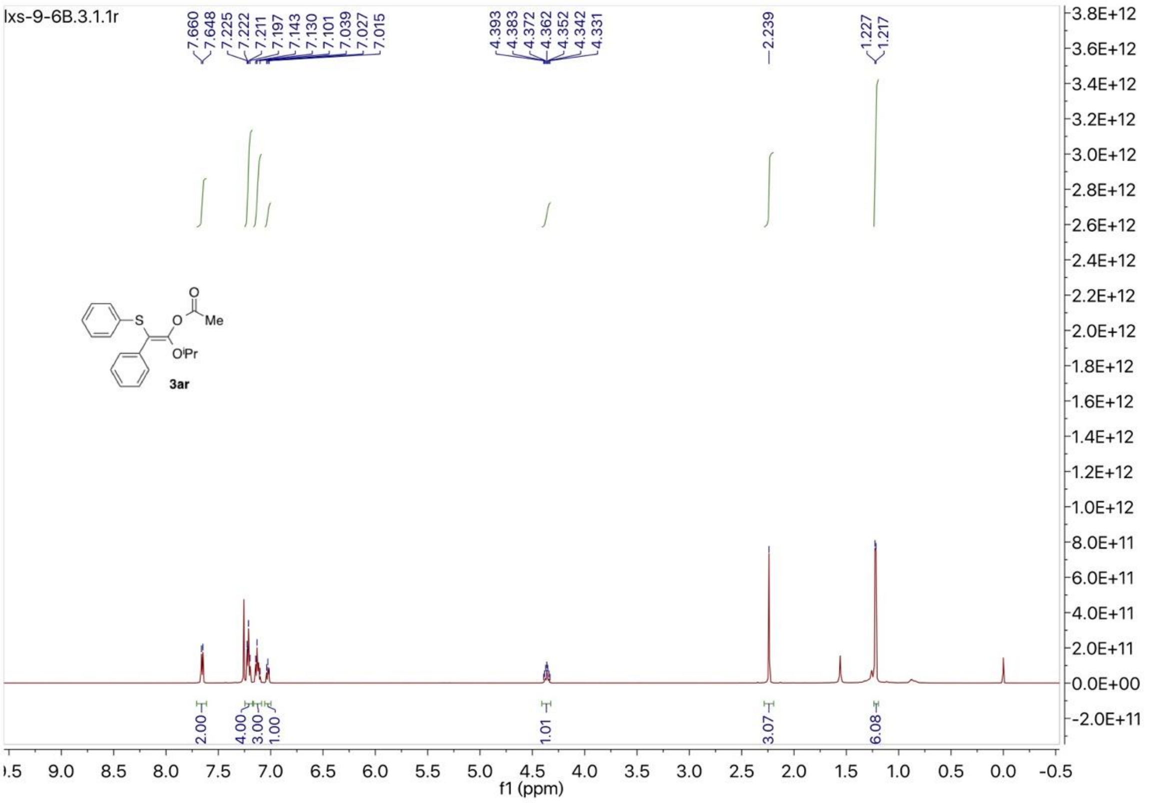

Supplementary Figure 201. ^{1}H NMR (600 MHz, $CDCl_3$) of compound **3ar**

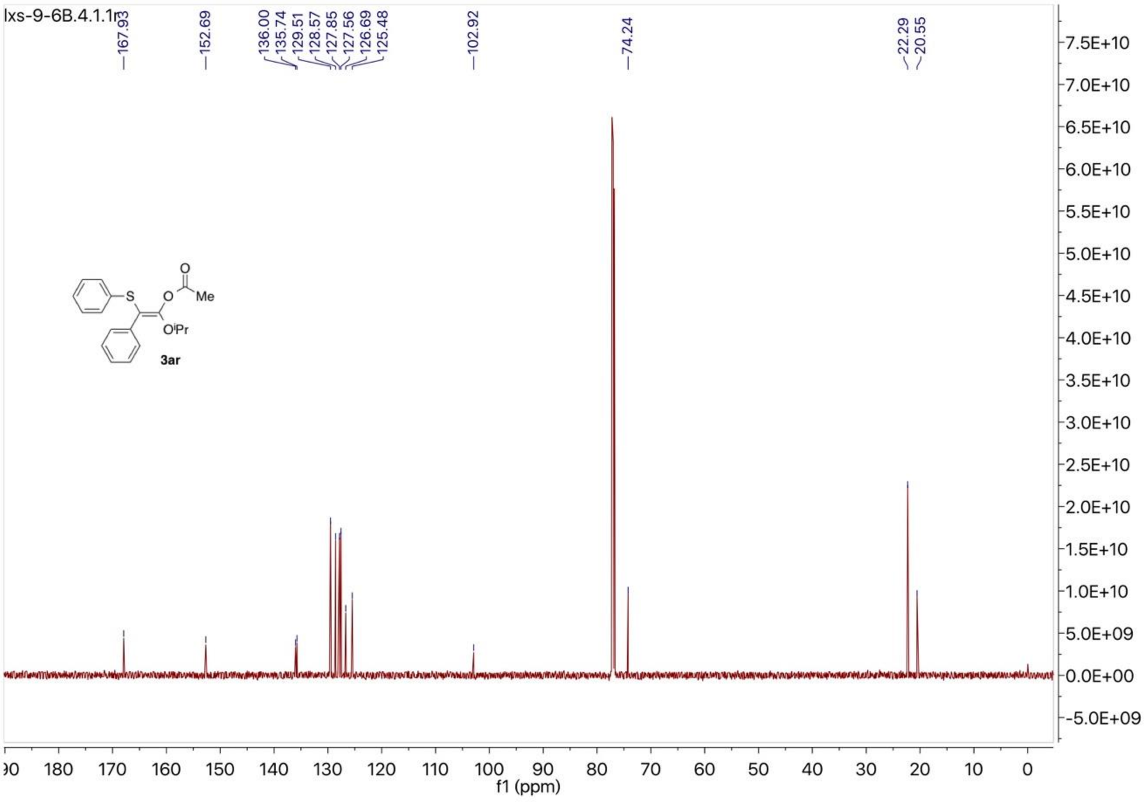

Supplementary Figure 202. ^{13}C NMR (150 MHz, $CDCl_3$) of compound **3ar**

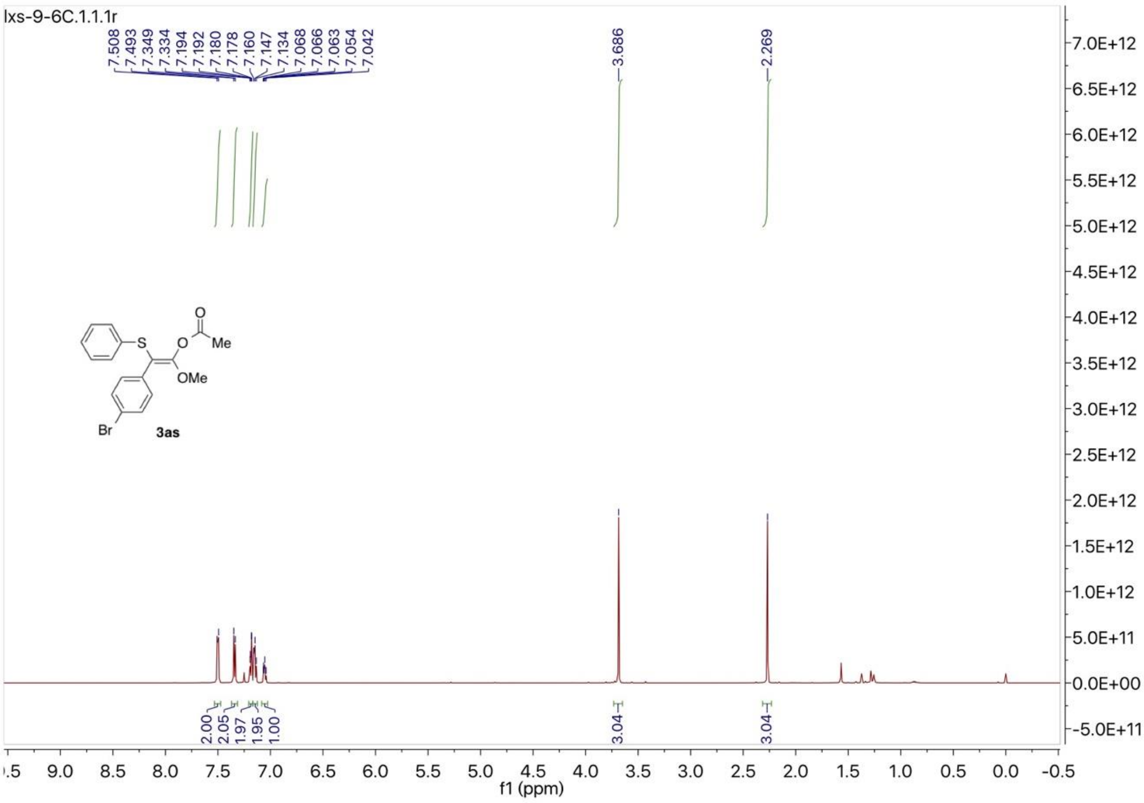

Supplementary Figure 203. ^{1}H NMR (600 MHz, $CDCl_3$) of compound **3as**

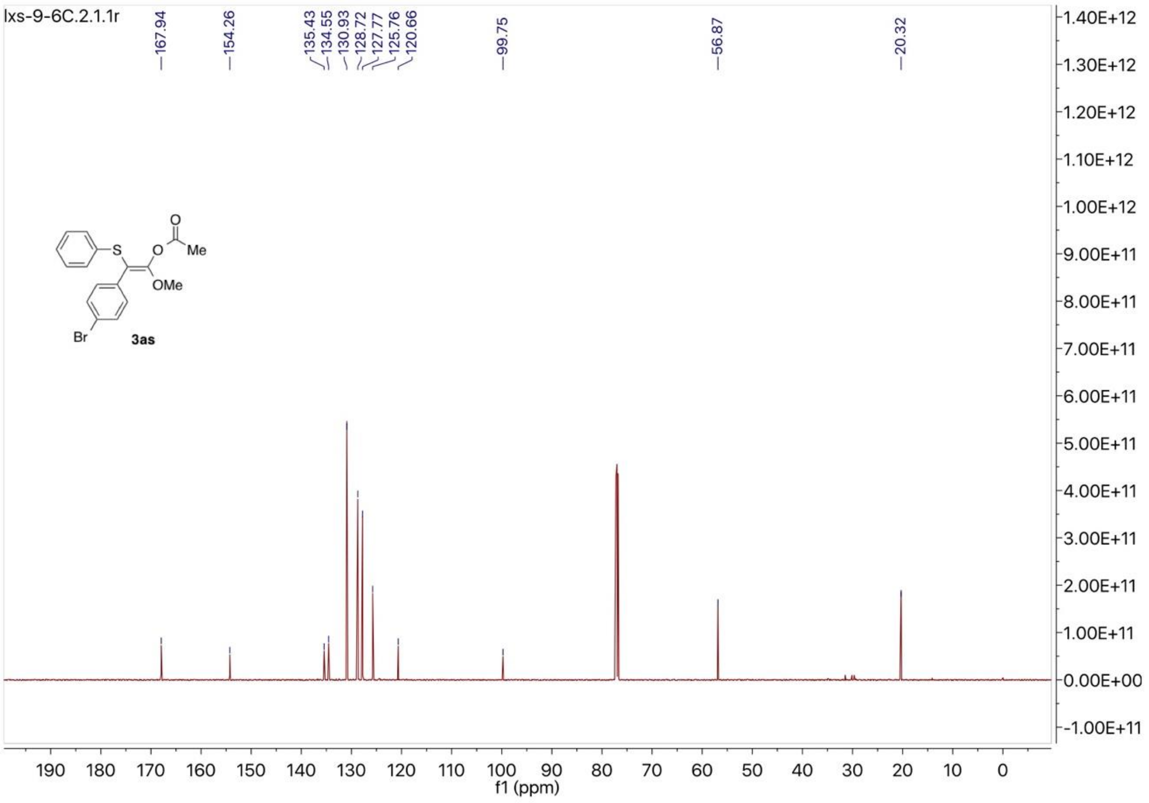

Supplementary Figure 204. ^{13}C NMR (150 MHz, $CDCl_3$) of compound **3as**

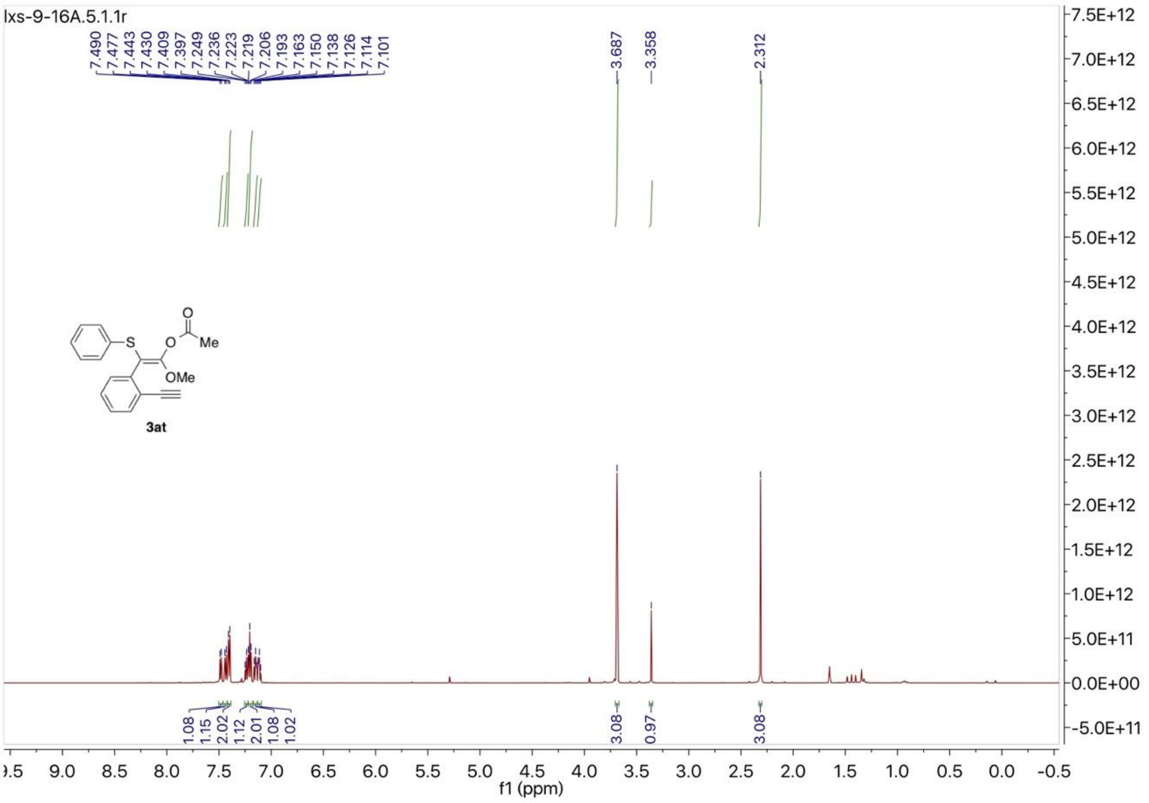

Supplementary Figure 205. ^{1}H NMR (600 MHz, $CDCl_3$) of compound **3at**

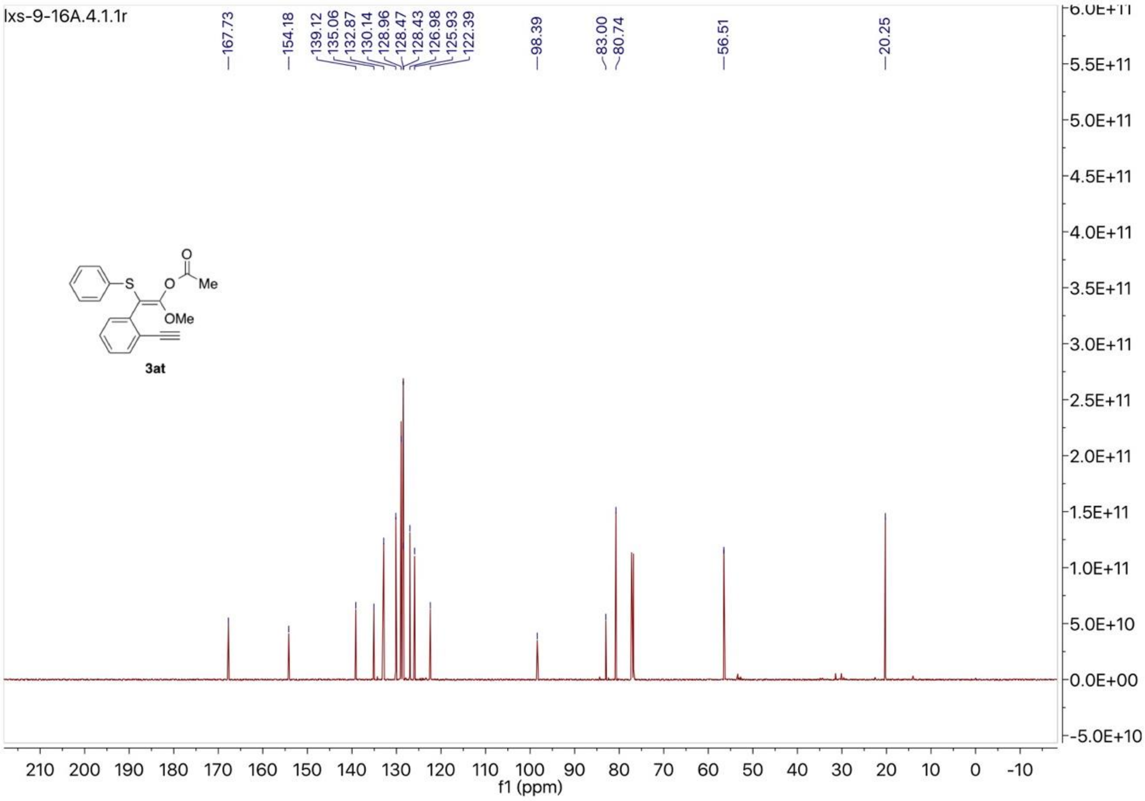

Supplementary Figure 206. ^{13}C NMR (150 MHz, $CDCl_3$) of compound **3at**

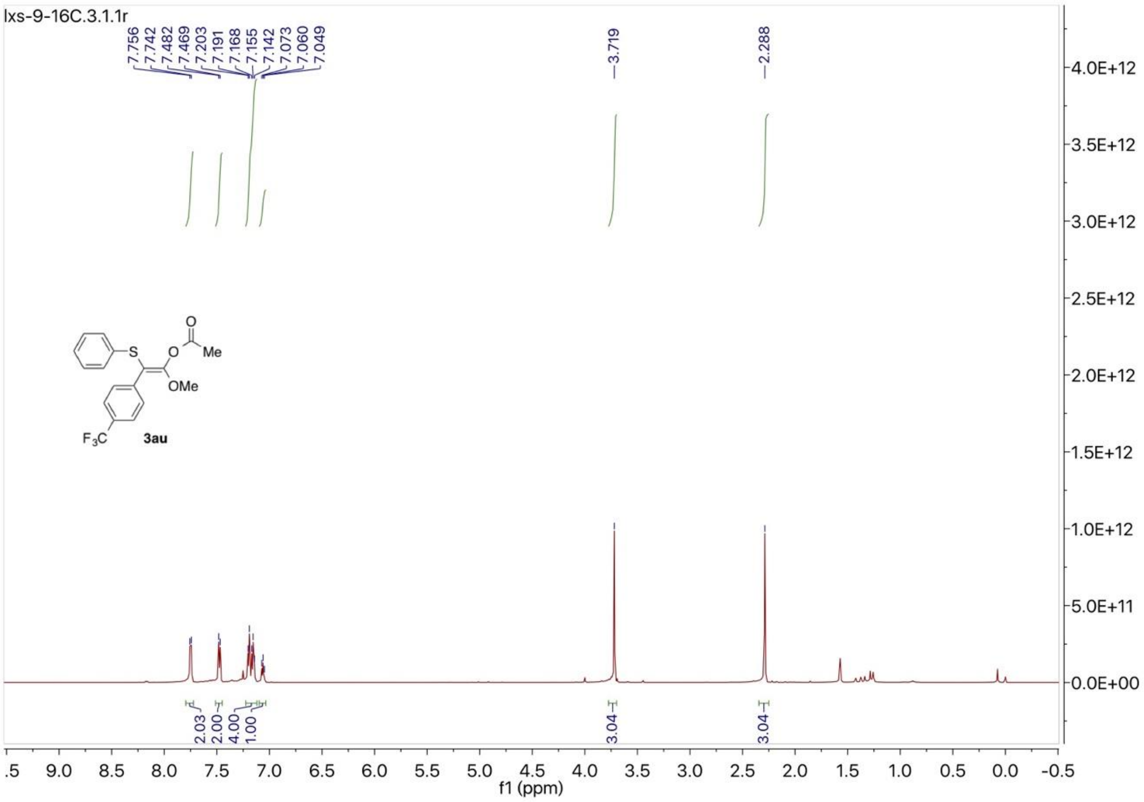

Supplementary Figure 207. ^{1}H NMR (600 MHz, $CDCl_3$) of compound **3au**

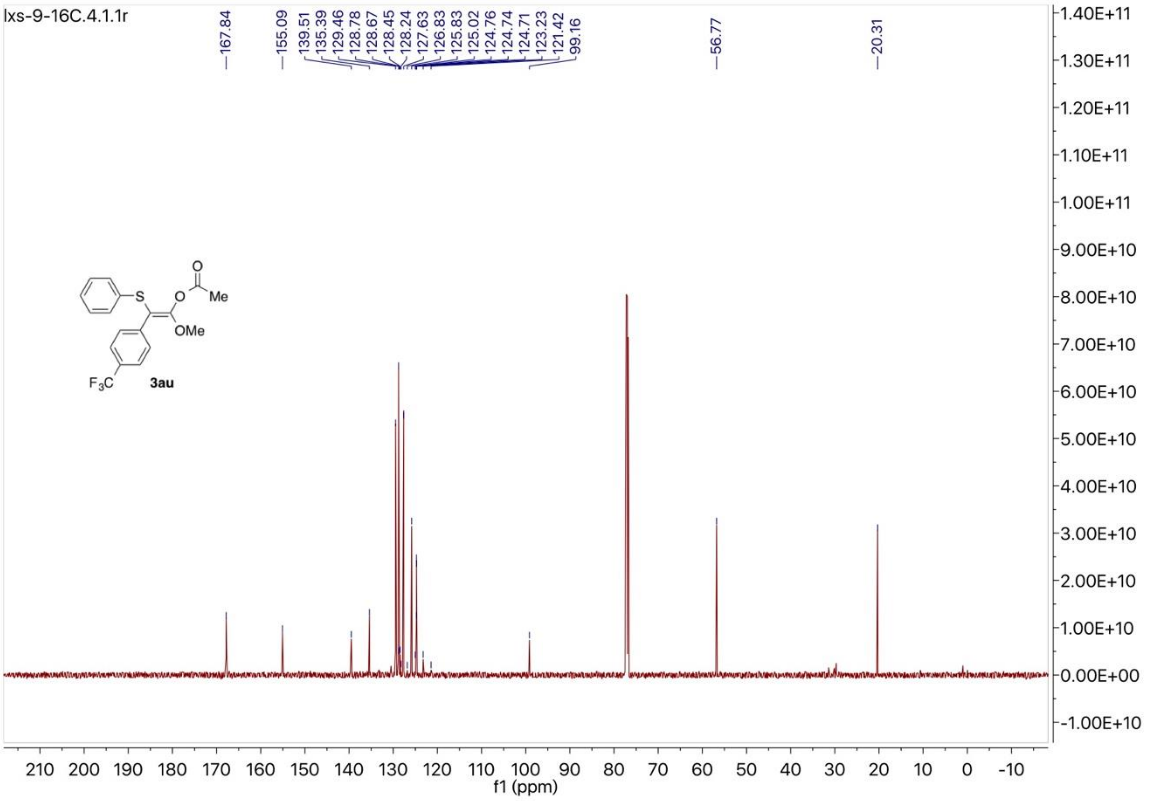

Supplementary Figure 208. ^{13}C NMR (150 MHz, $CDCl_3$) of compound **3au**

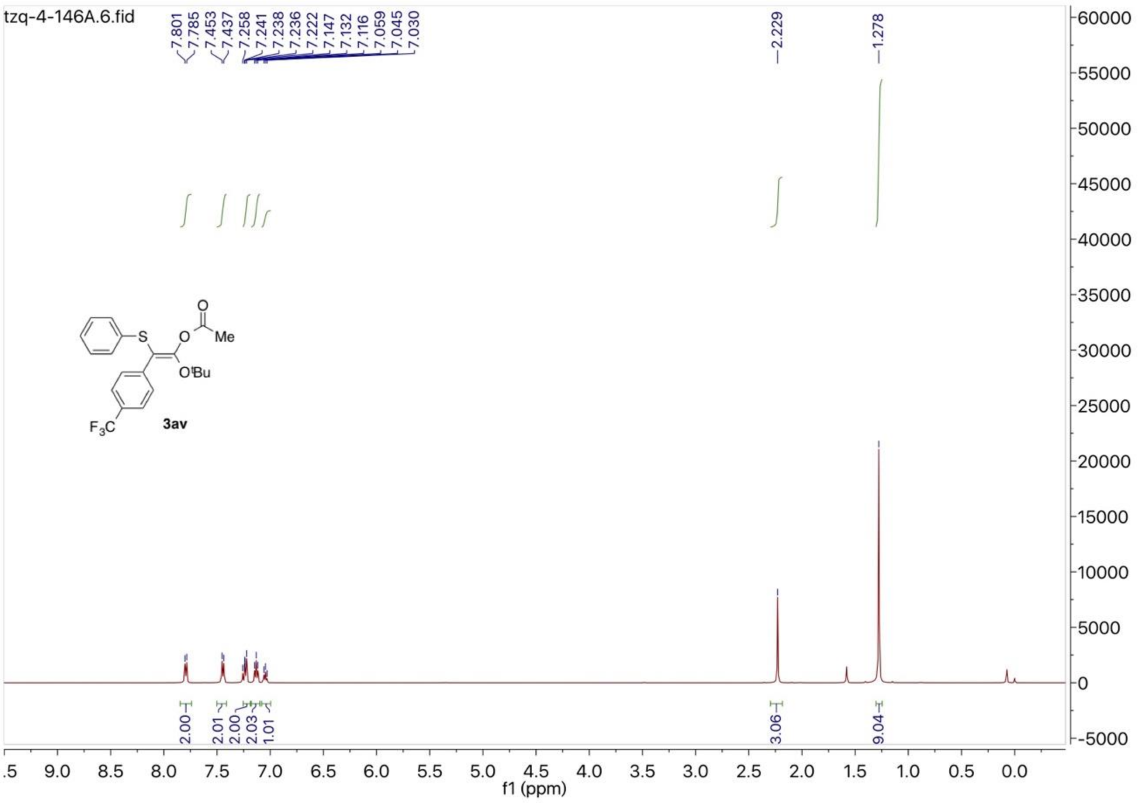

Supplementary Figure 209. ^{1}H NMR (500 MHz, $CDCl_3$) of compound **3av**

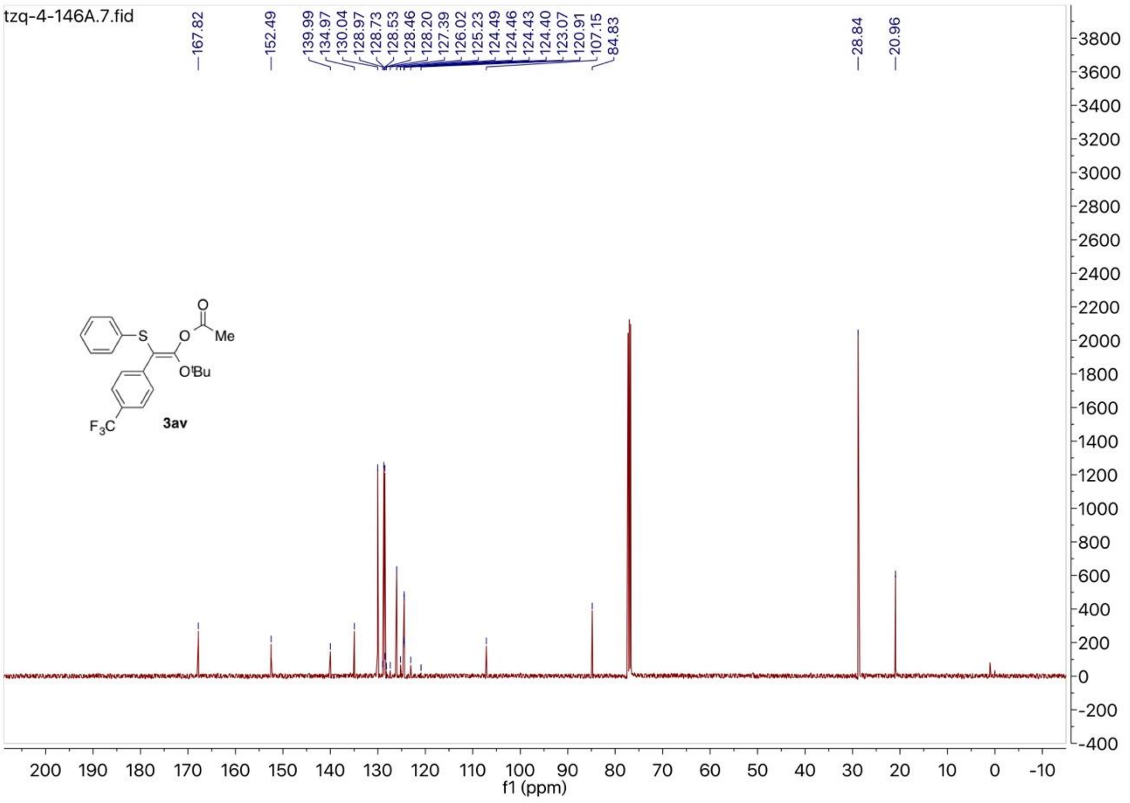

Supplementary Figure 210. ^{13}C NMR (125 MHz, $CDCl_3$) of compound **3av**

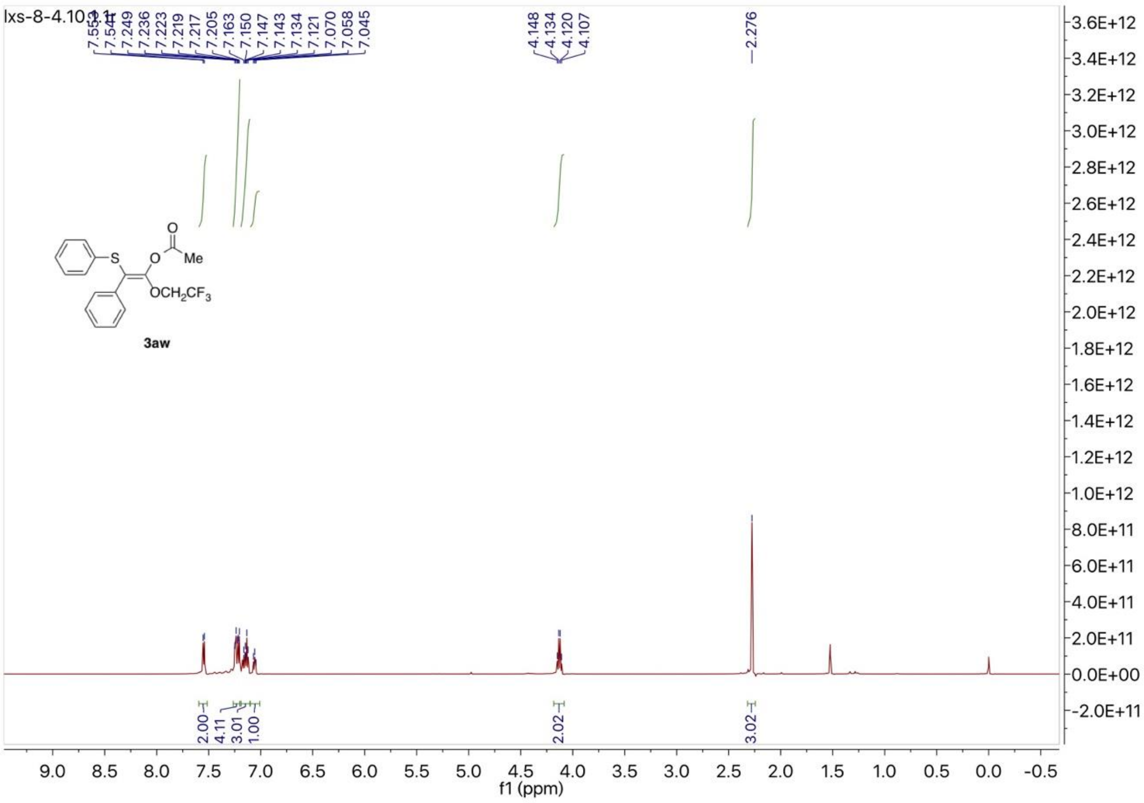

Supplementary Figure 211. ^{1}H NMR (600 MHz, $CDCl_3$) of compound **3aw**

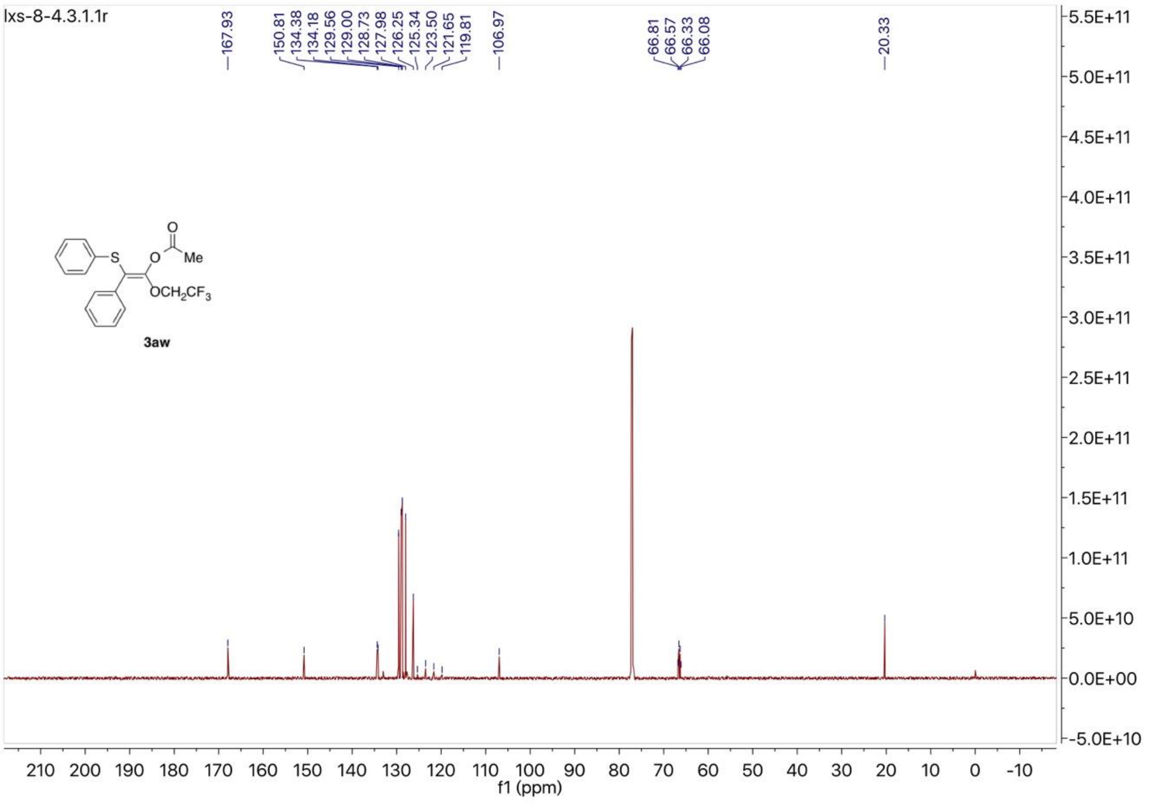

Supplementary Figure 212. ^{13}C NMR (150 MHz, $CDCl_3$) of compound **3aw**

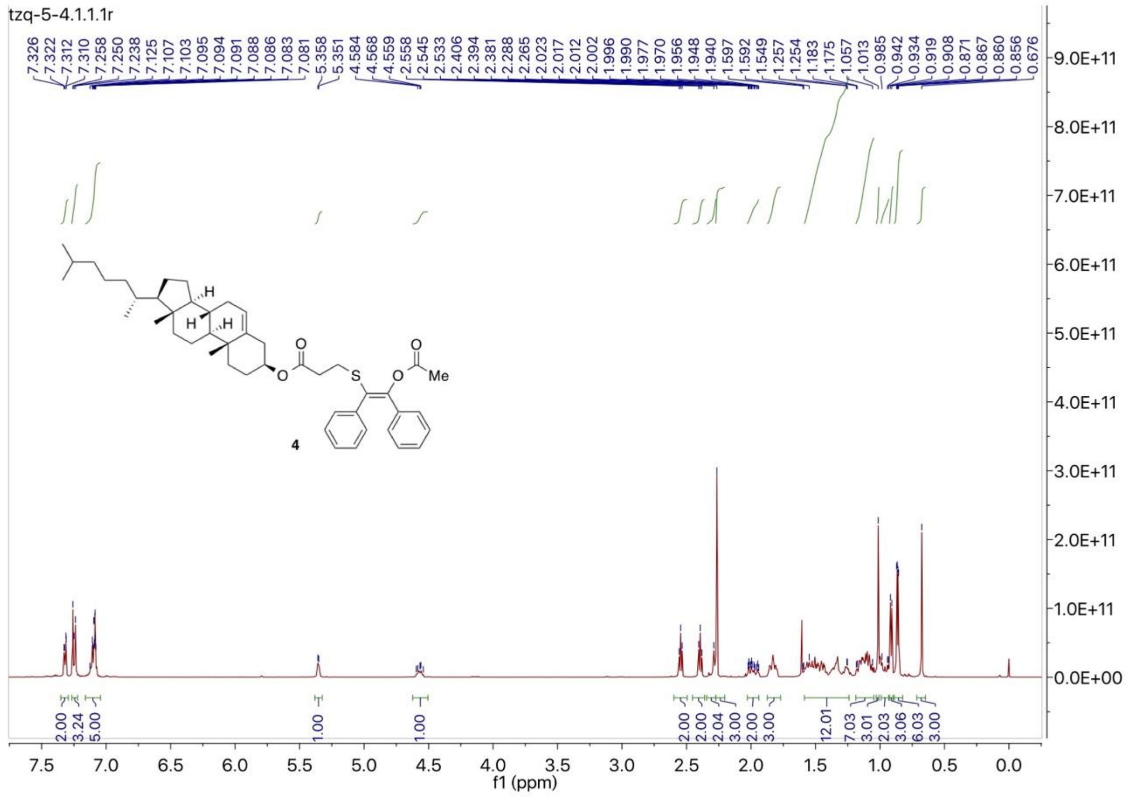

Supplementary Figure 213. ^{1}H NMR (600 MHz, $CDCl_3$) of compound **4**

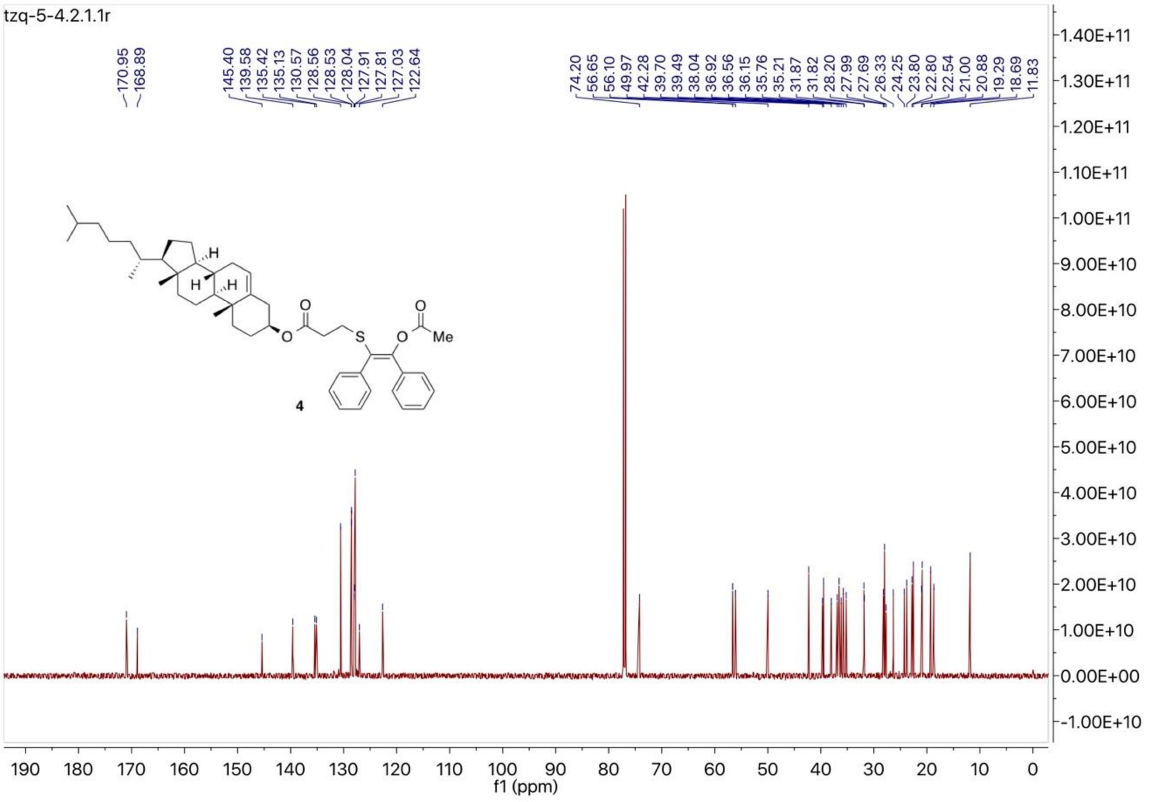

Supplementary Figure 214. ^{13}C NMR (150 MHz, $CDCl_3$) of compound **4**

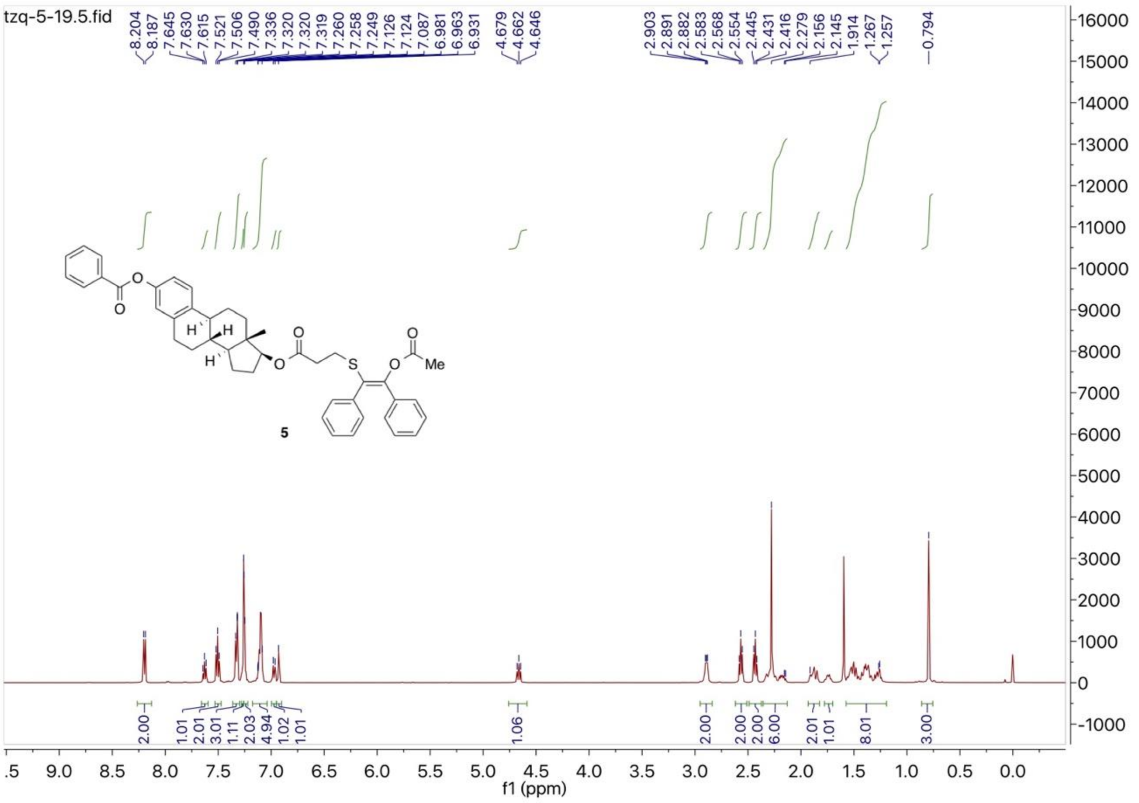

Supplementary Figure 215. ^{1}H NMR (500 MHz, $CDCl_3$) of compound **5**

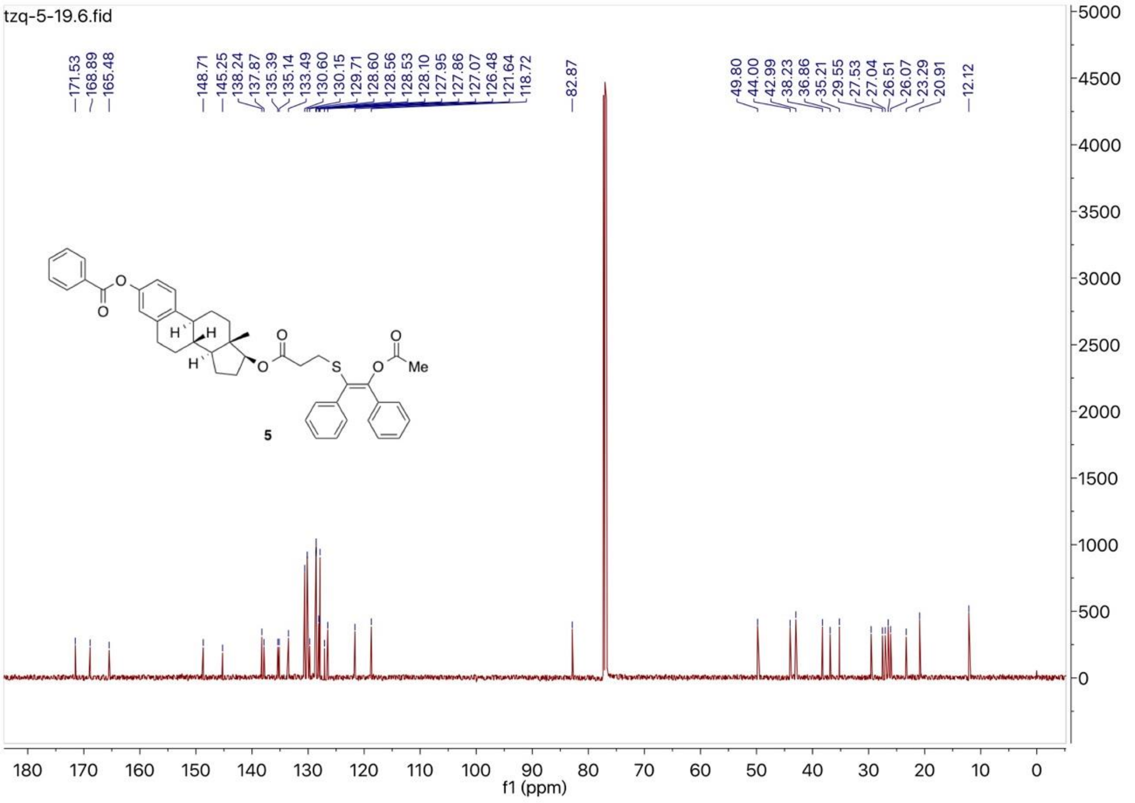

Supplementary Figure 216. ^{13}C NMR (125 MHz, $CDCl_3$) of compound **5**

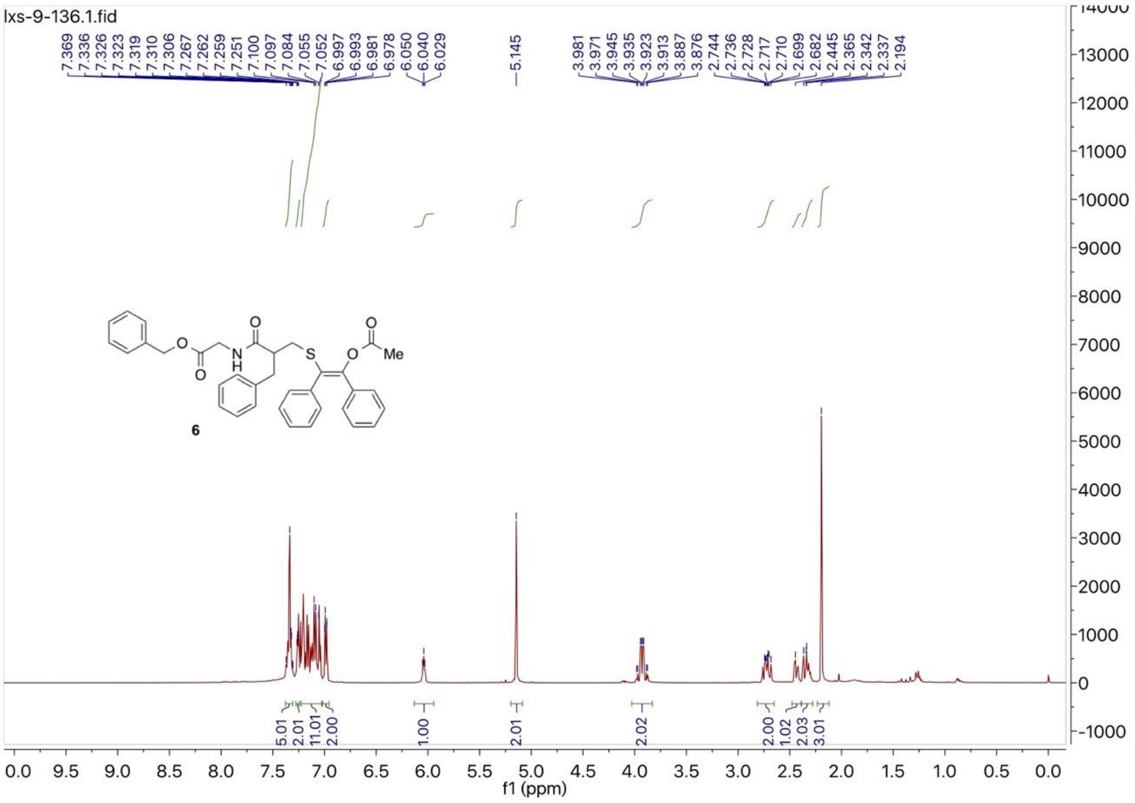

Supplementary Figure 217. ^{1}H NMR (500 MHz, $CDCl_3$) of compound **6**

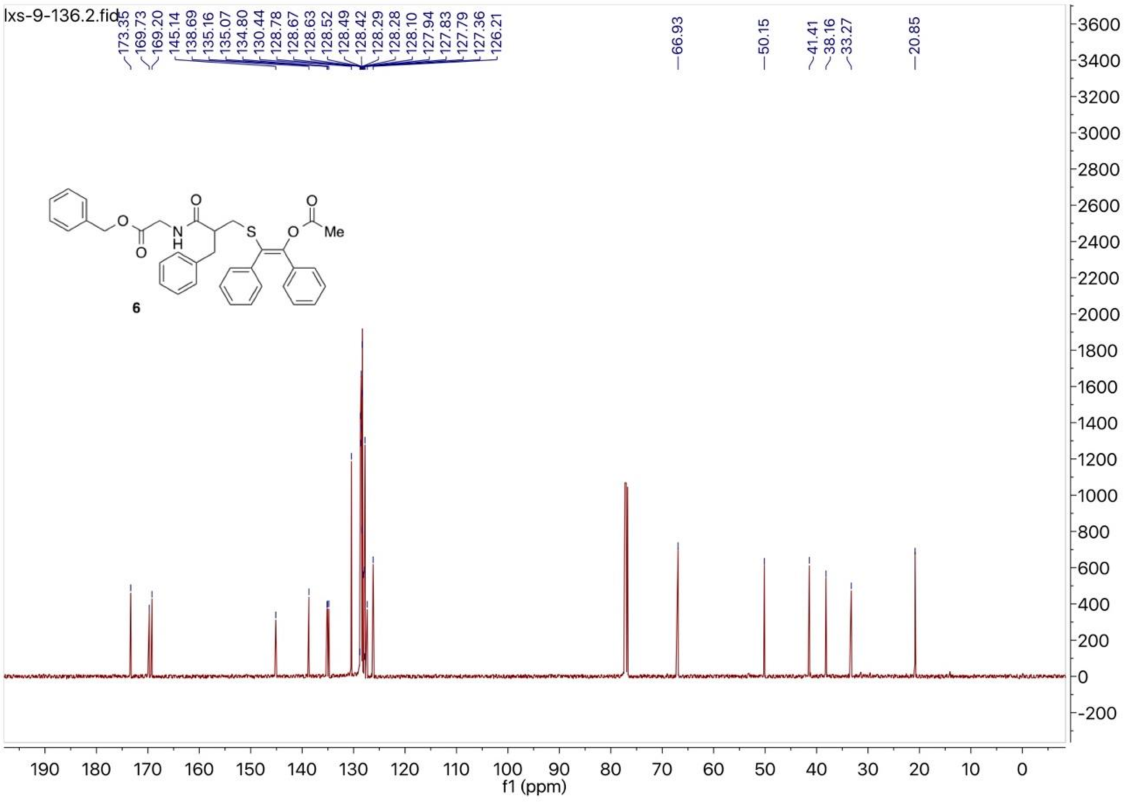

Supplementary Figure 218. ^{13}C NMR (125 MHz, $CDCl_3$) of compound **6**

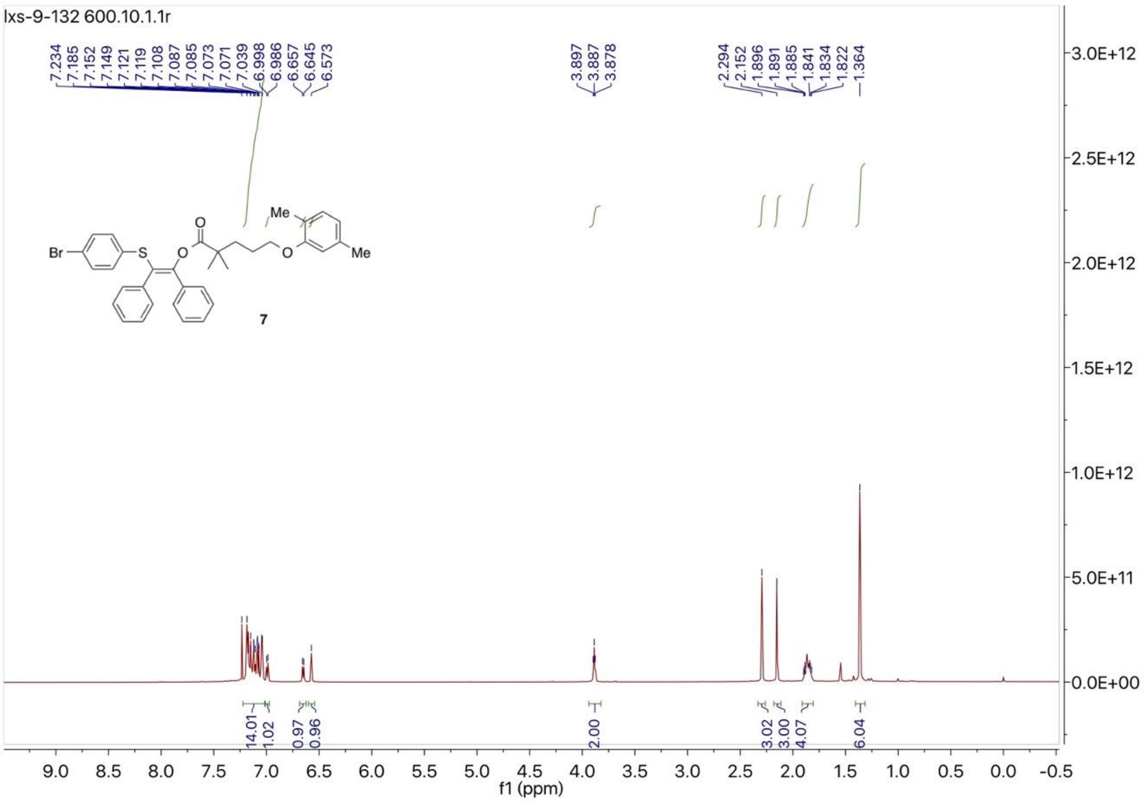

Supplementary Figure 219. ^{1}H NMR (600 MHz, $CDCl_3$) of compound **7**

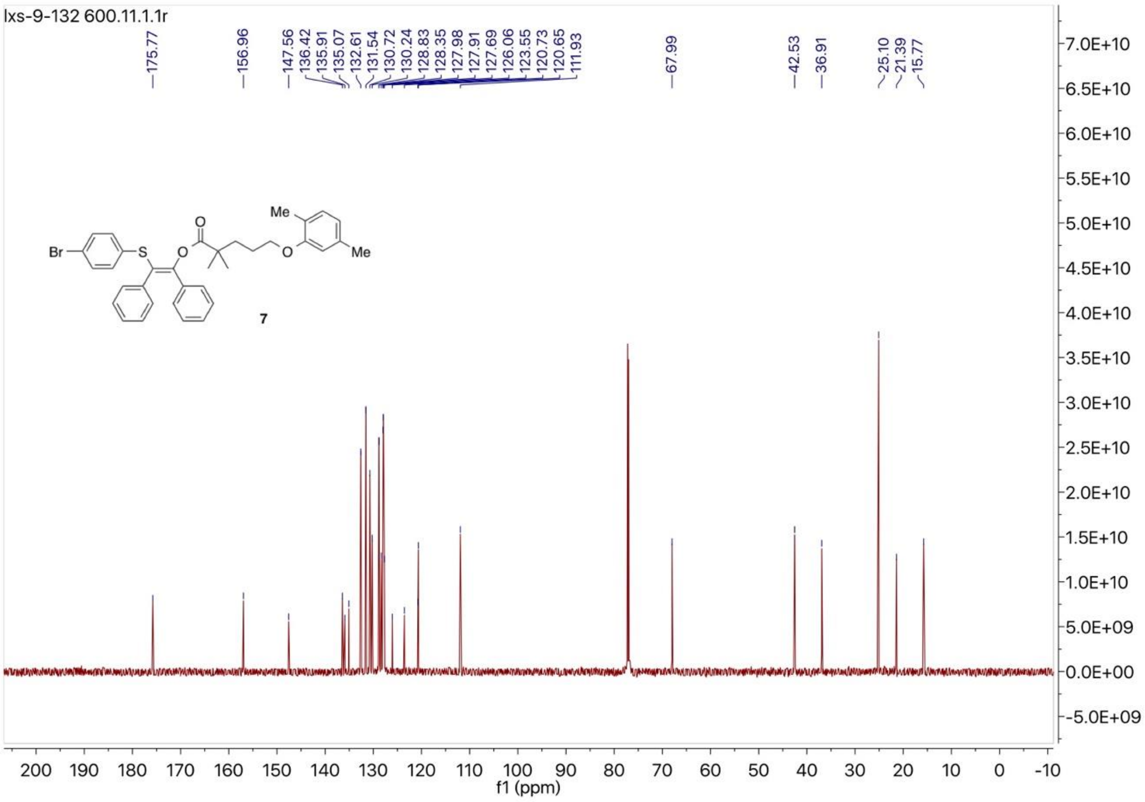

Supplementary Figure 220. ^{13}C NMR (150 MHz, $CDCl_3$) of compound **7**

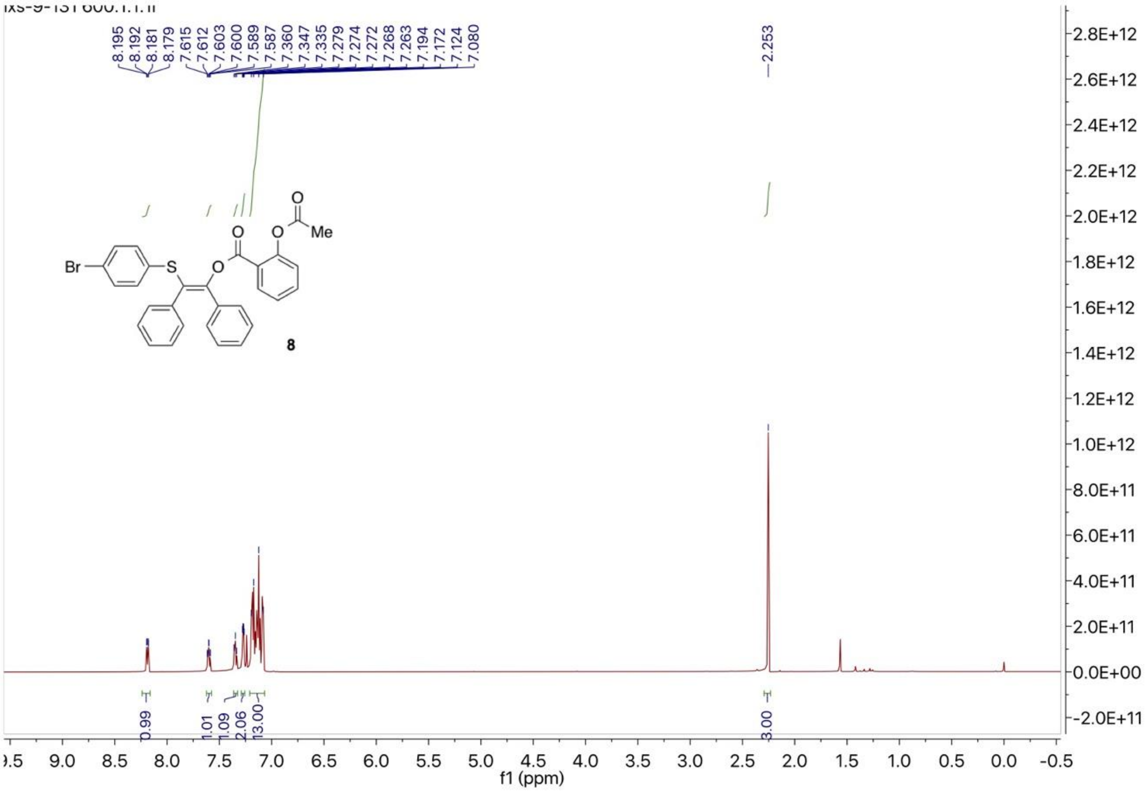

Supplementary Figure 221. ^{1}H NMR (600 MHz, $CDCl_3$) of compound **8**

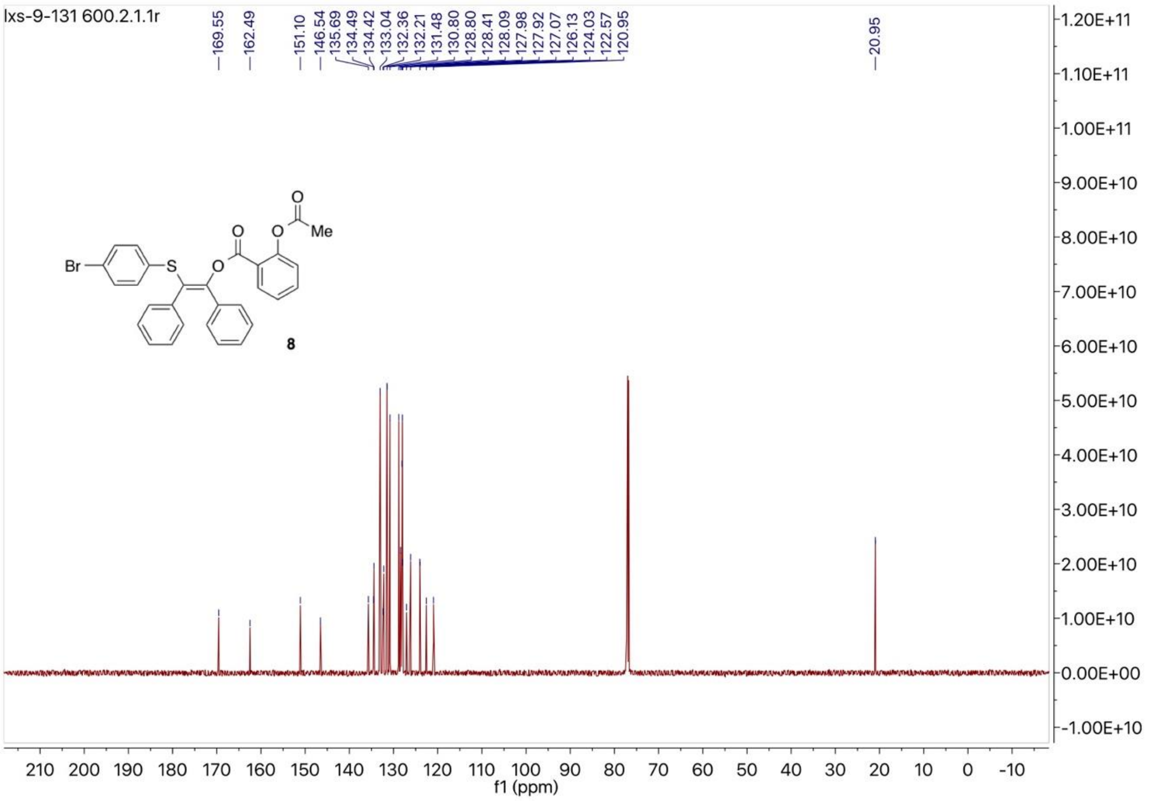

Supplementary Figure 222. ^{13}C NMR (150 MHz, $CDCl_3$) of compound **8**

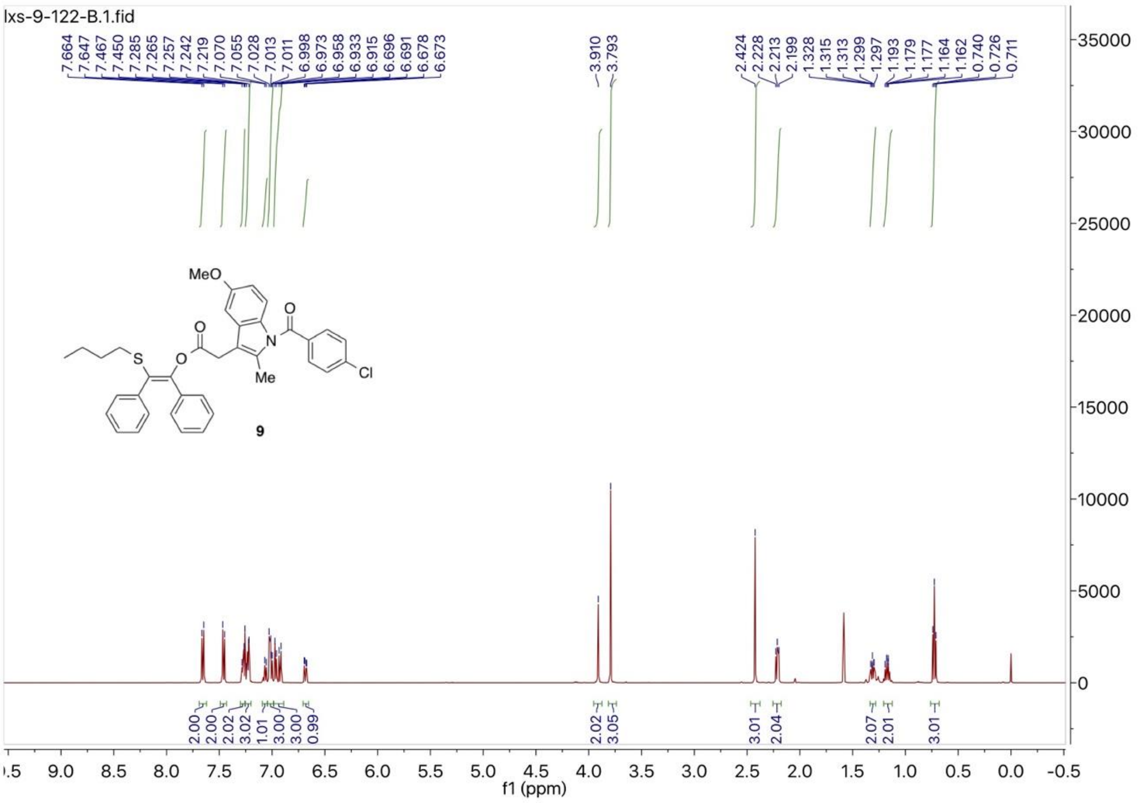

Supplementary Figure 223. ^{1}H NMR (500 MHz, $CDCl_3$) of compound **9**

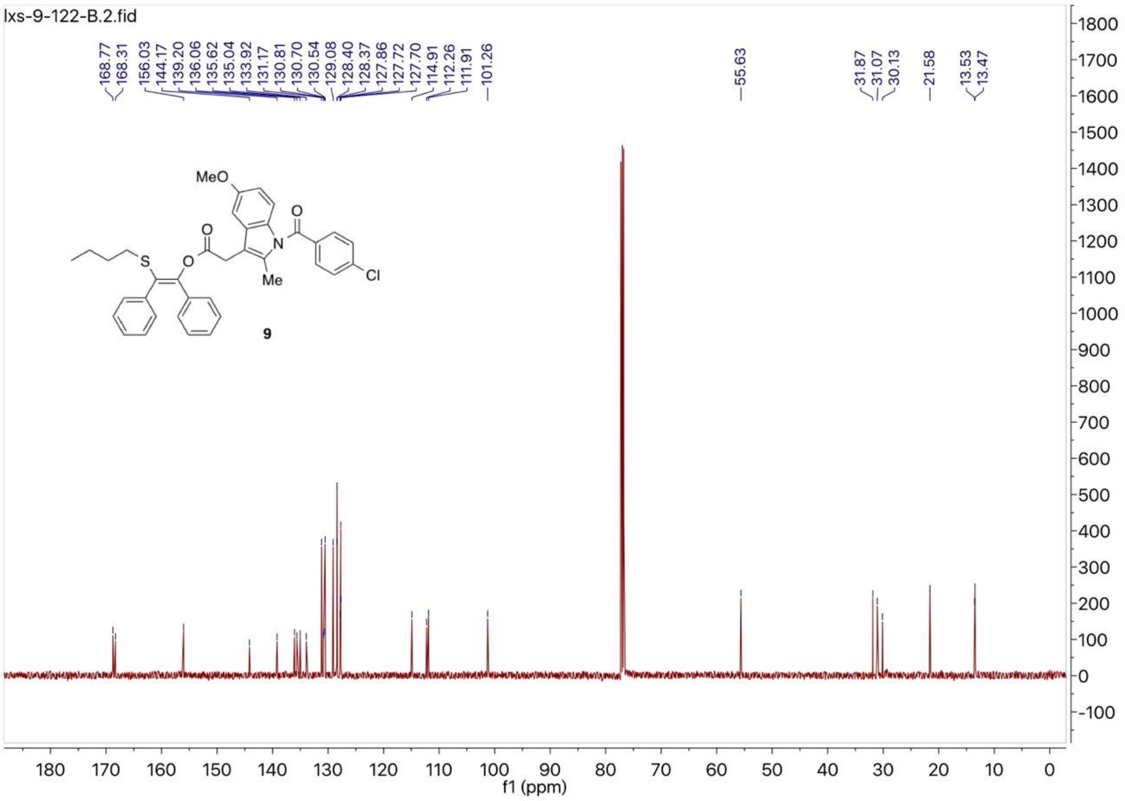

Supplementary Figure 224. ^{13}C NMR (125 MHz, $CDCl_3$) of compound **9**

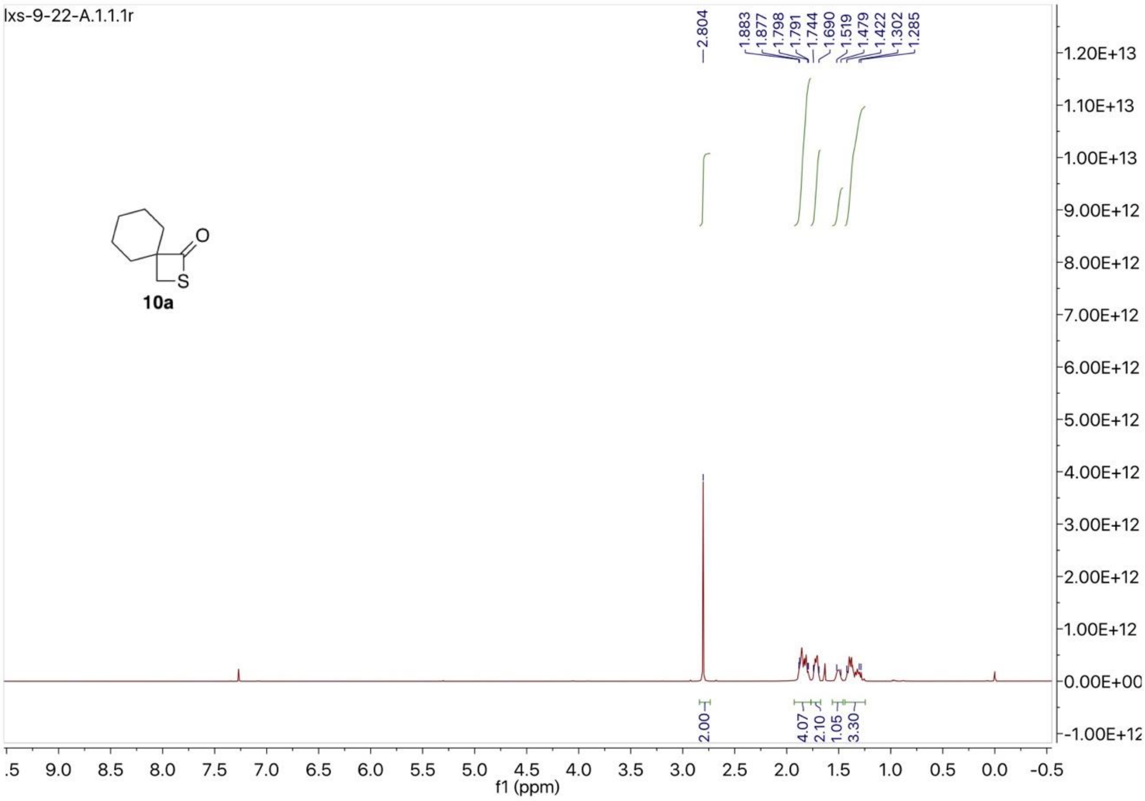

Supplementary Figure 225. ^{1}H NMR (600 MHz, $CDCl_3$) of compound **10a**

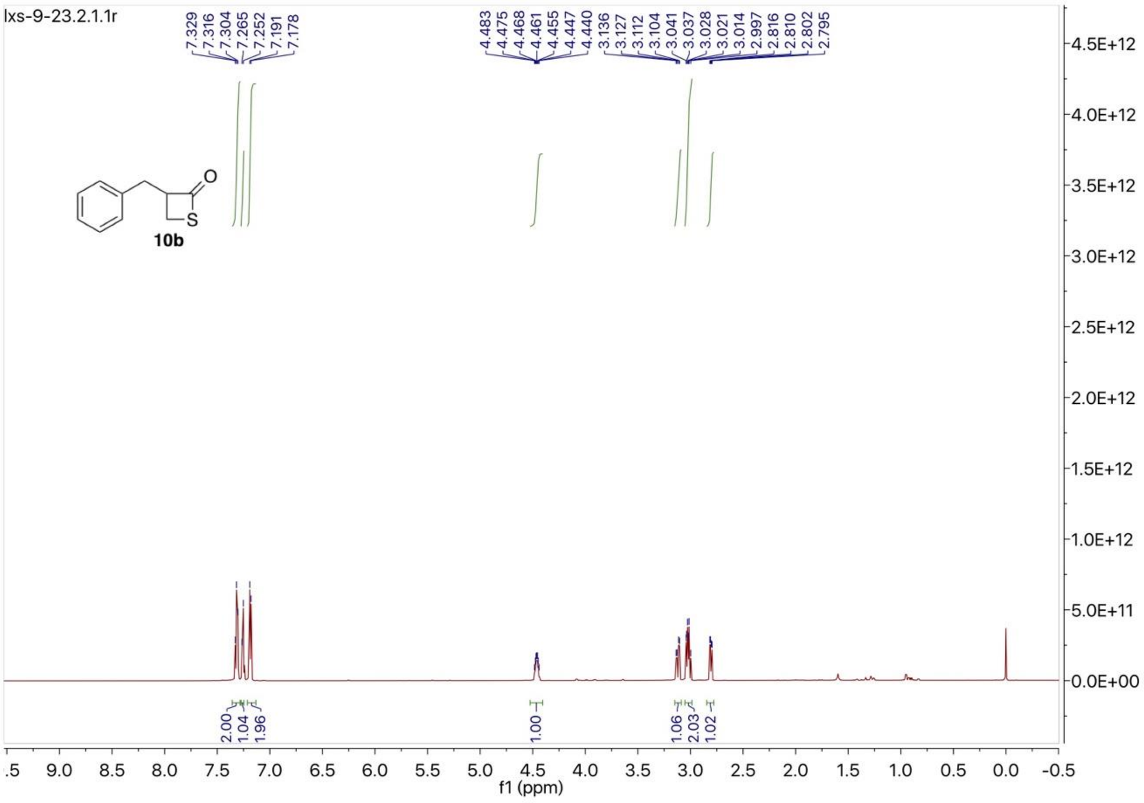

Supplementary Figure 226. ^{1}H NMR (600 MHz, $CDCl_3$) of compound **10b**

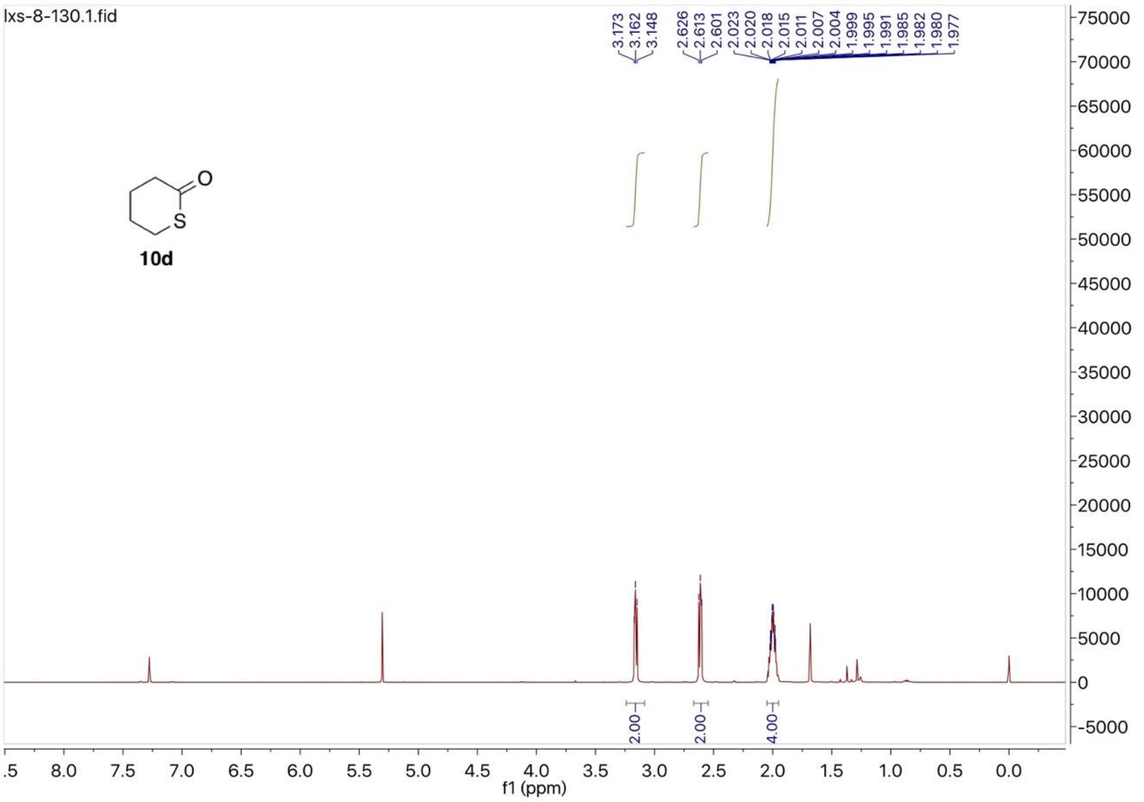

Supplementary Figure 227. ^{1}H NMR (500 MHz, $CDCl_3$) of compound **10d**

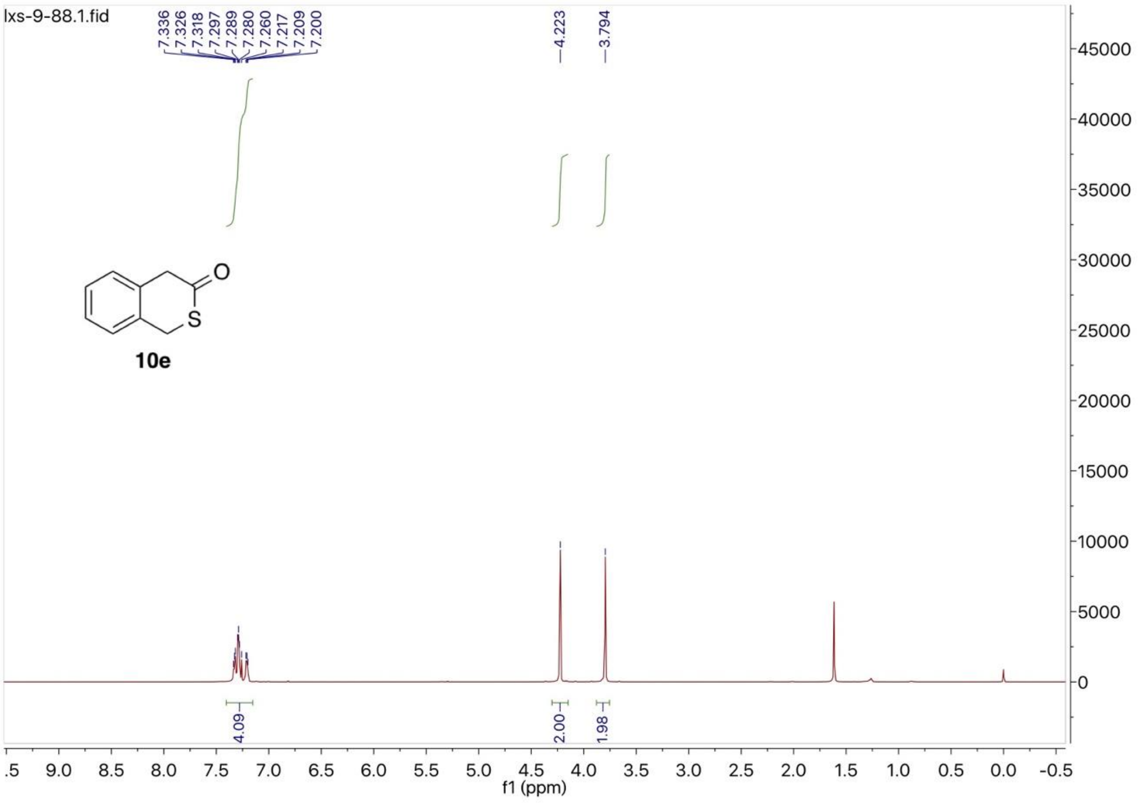

Supplementary Figure 228. ^{1}H NMR (500 MHz, $CDCl_3$) of compound **10e**

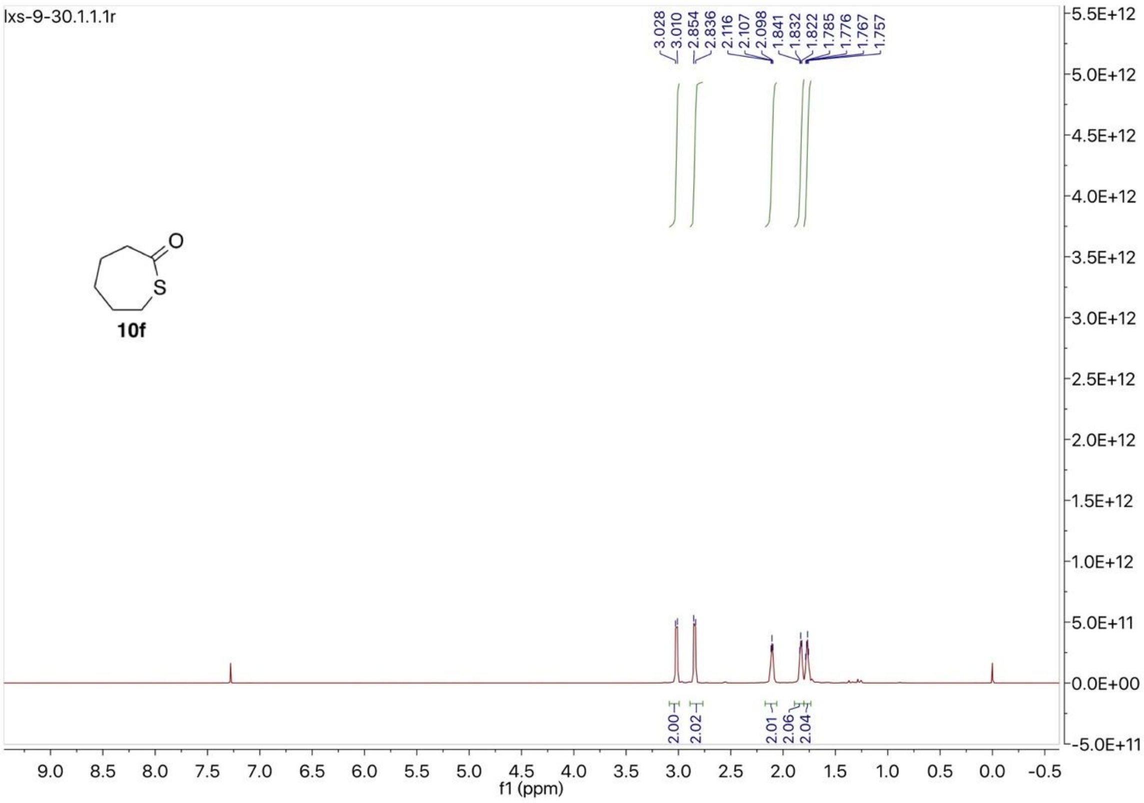

Supplementary Figure 229. ^{1}H NMR (600 MHz, $CDCl_3$) of compound **10f**

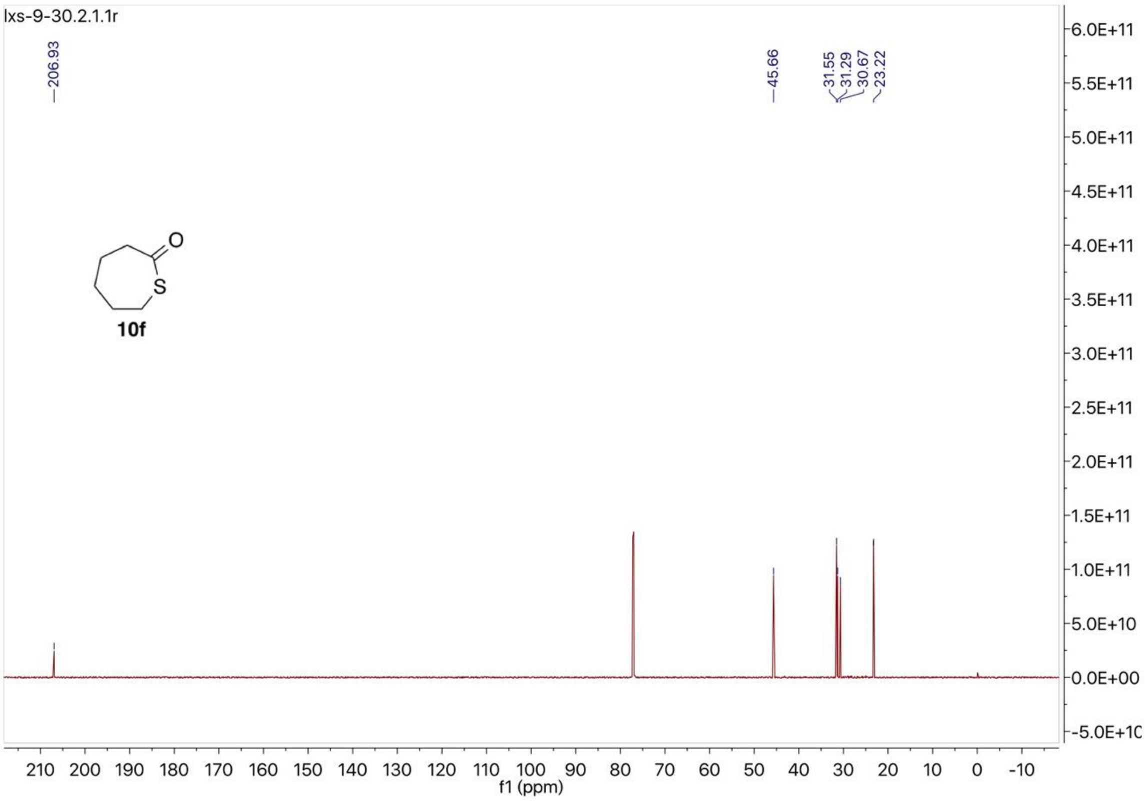

Supplementary Figure 230. ^{1}H NMR (150 MHz, $CDCl_3$) of compound **10f**

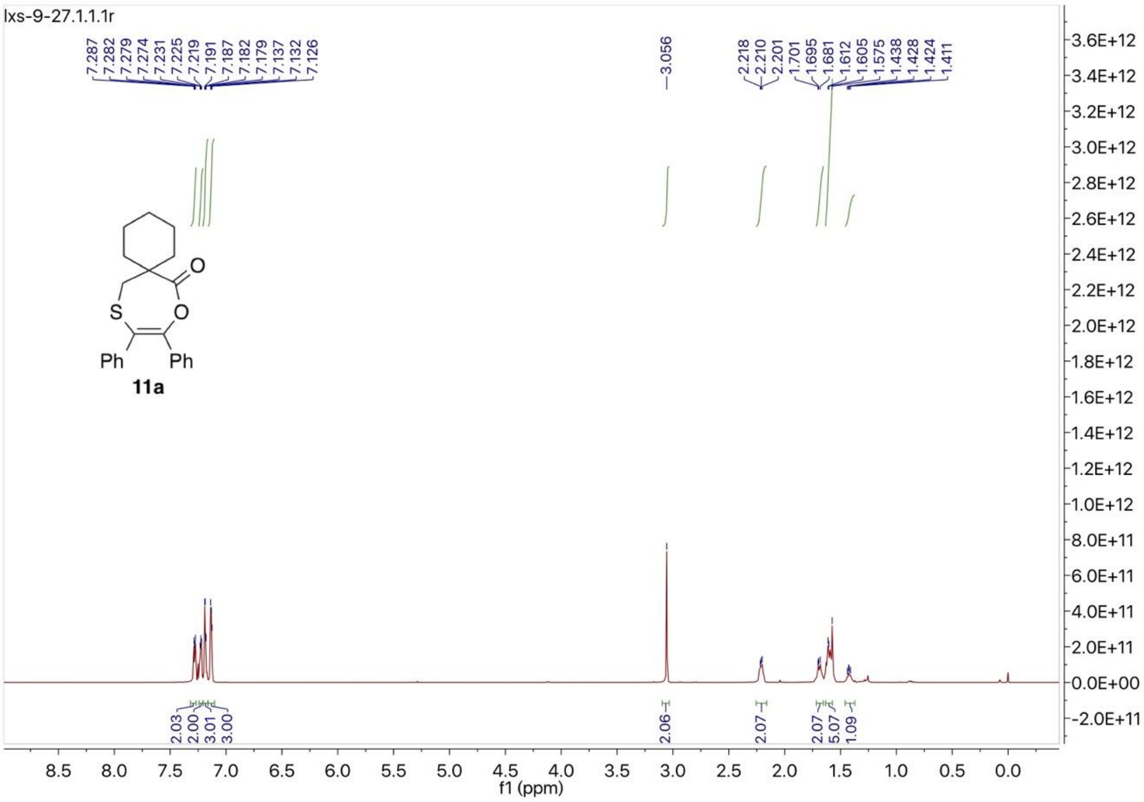

Supplementary Figure 231. ^{1}H NMR (600 MHz, $CDCl_3$) of compound **11a**

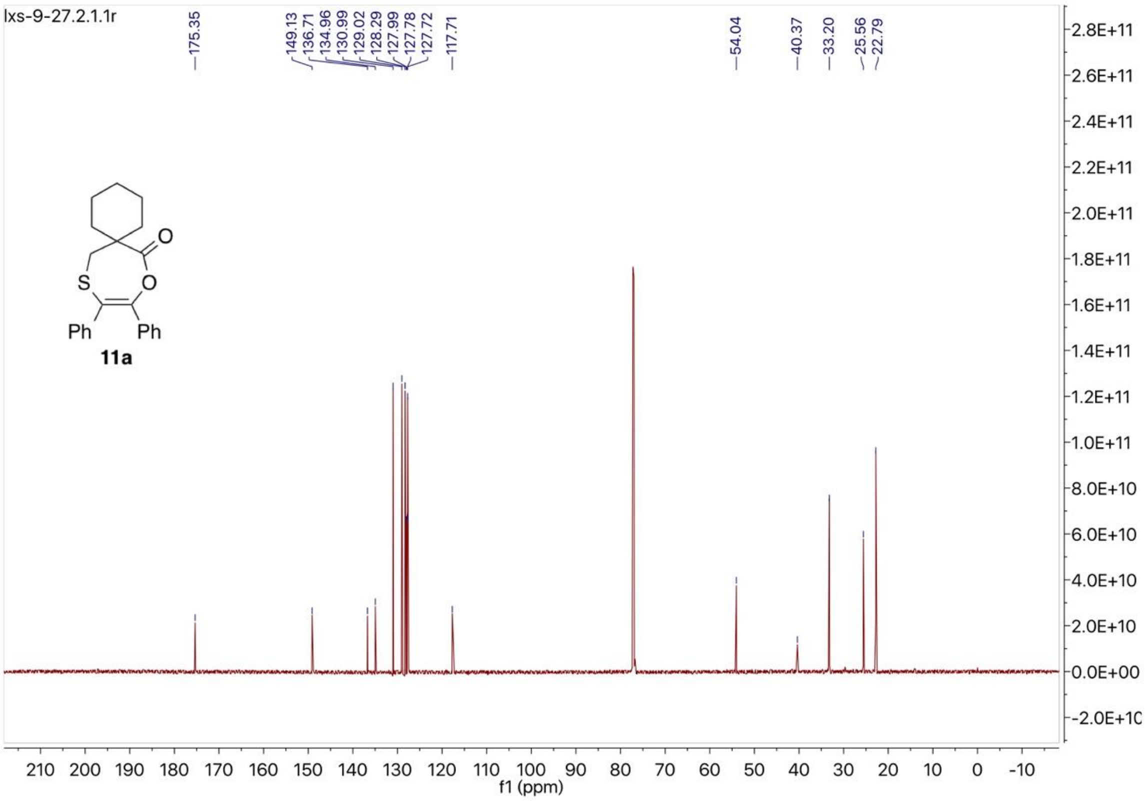

Supplementary Figure 232. ^{13}C NMR (150 MHz, $CDCl_3$) of compound **11a**

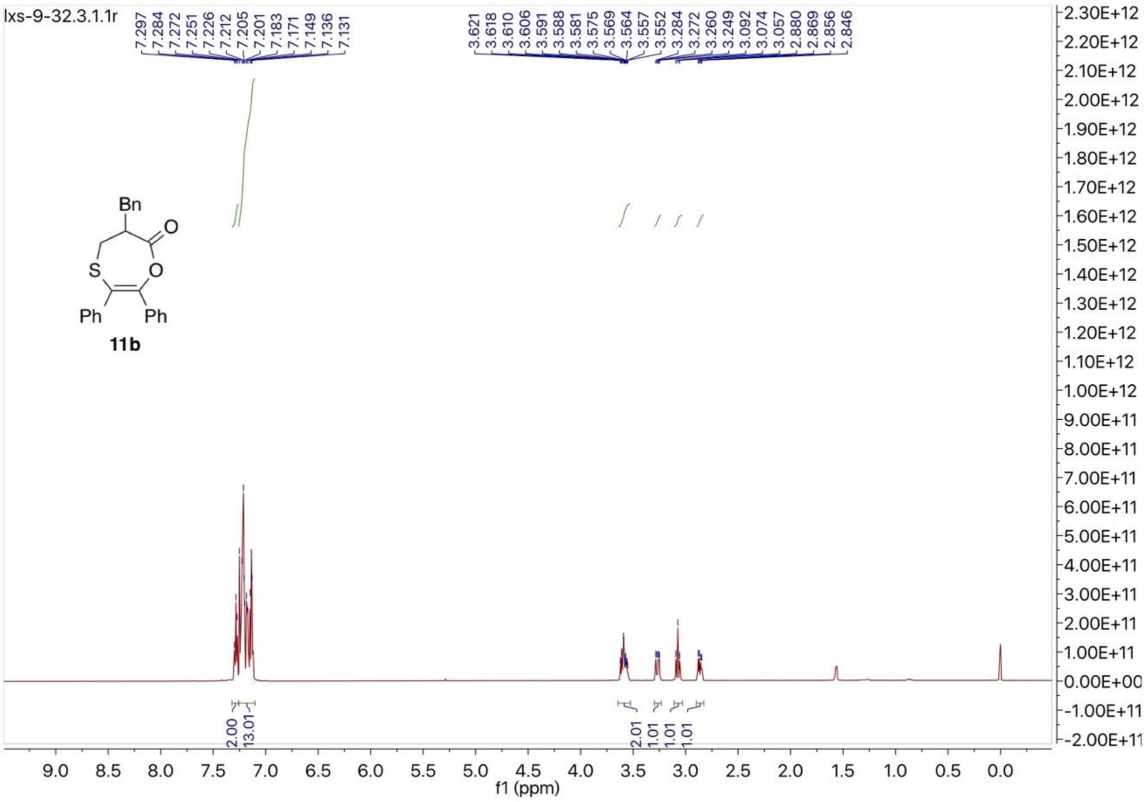

Supplementary Figure 233. ^{1}H NMR (600 MHz, $CDCl_3$) of compound **11b**

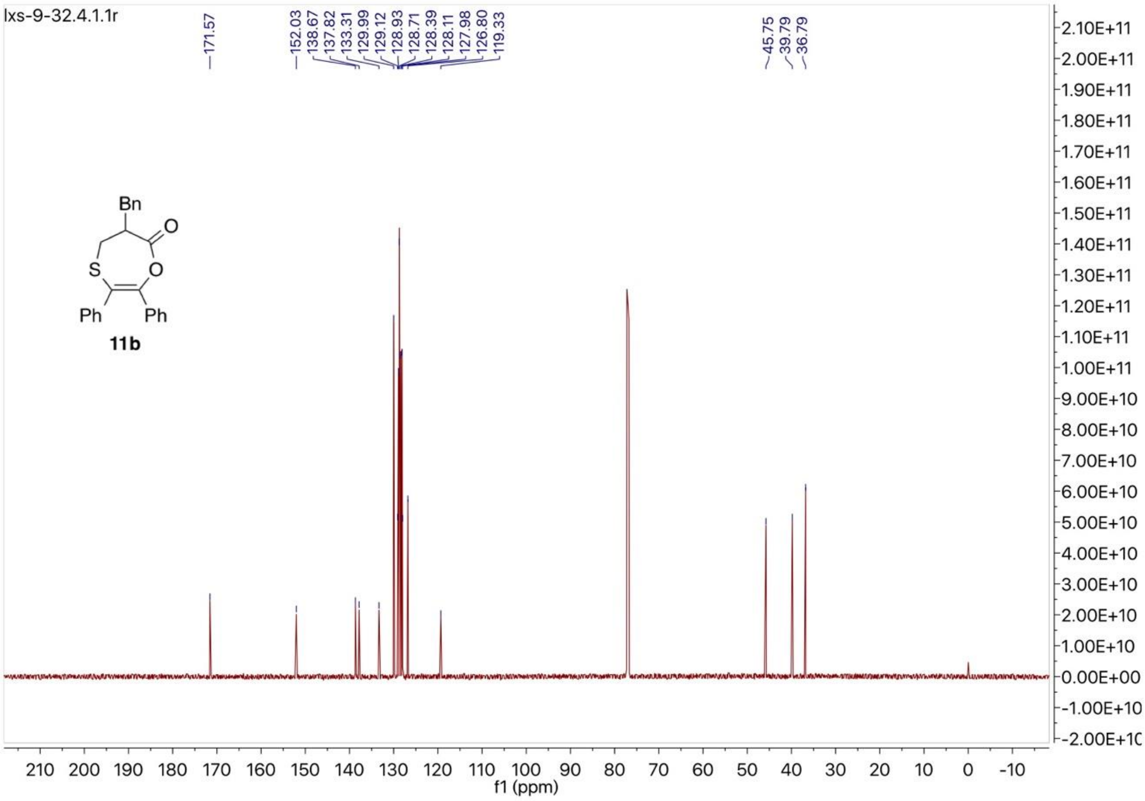

Supplementary Figure 234. ^{13}C NMR (150 MHz, $CDCl_3$) of compound **11b**

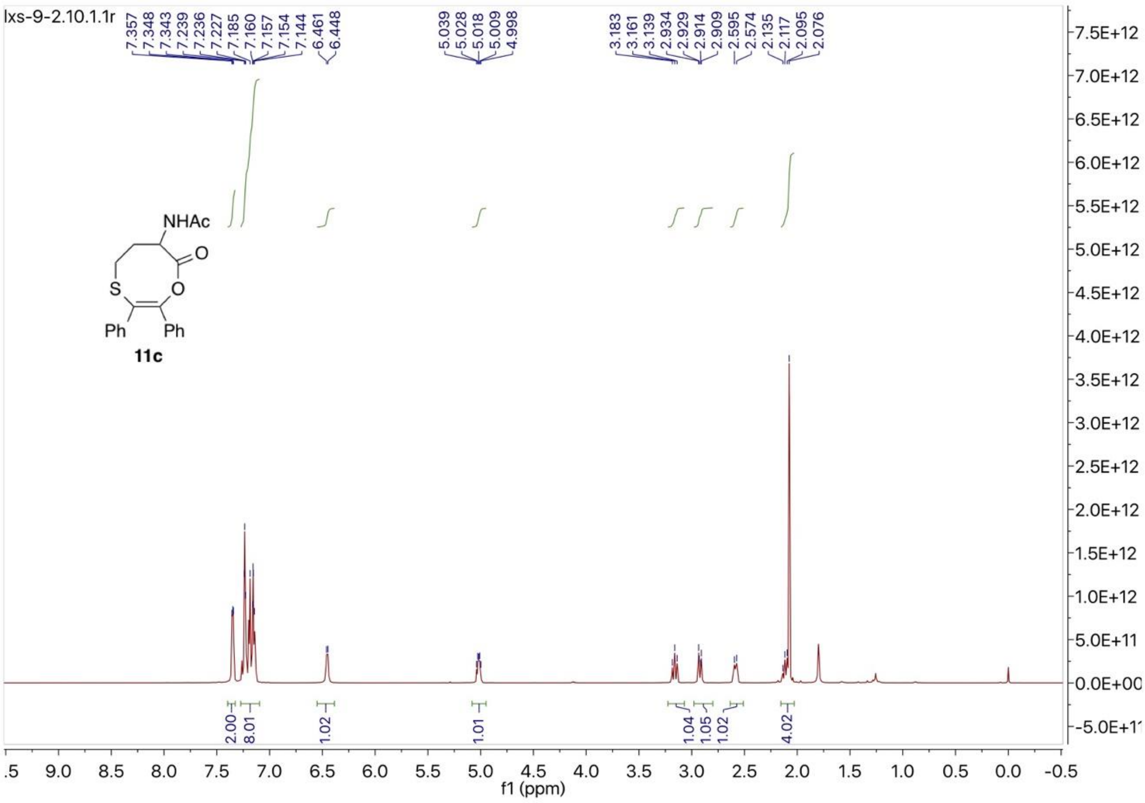

Supplementary Figure 235. ^{1}H NMR (600 MHz, $CDCl_3$) of compound **11c**

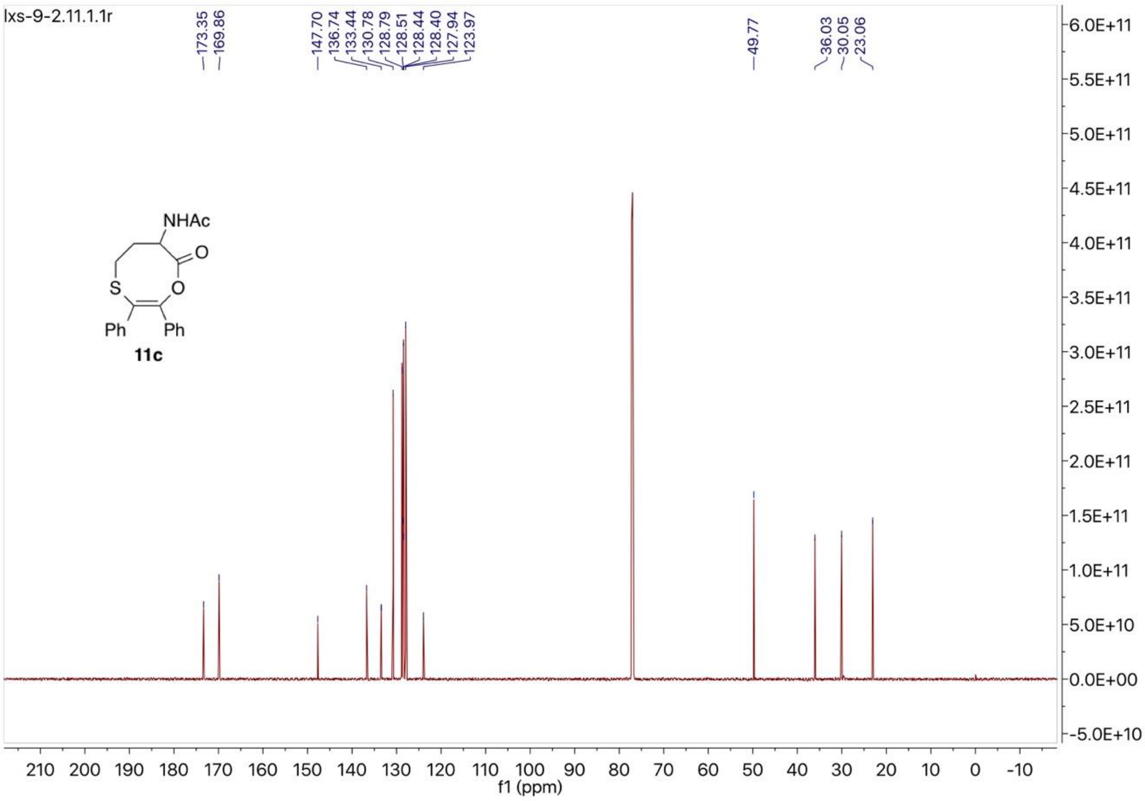

Supplementary Figure 236. ^{13}C NMR (150 MHz, $CDCl_3$) of compound **11c**

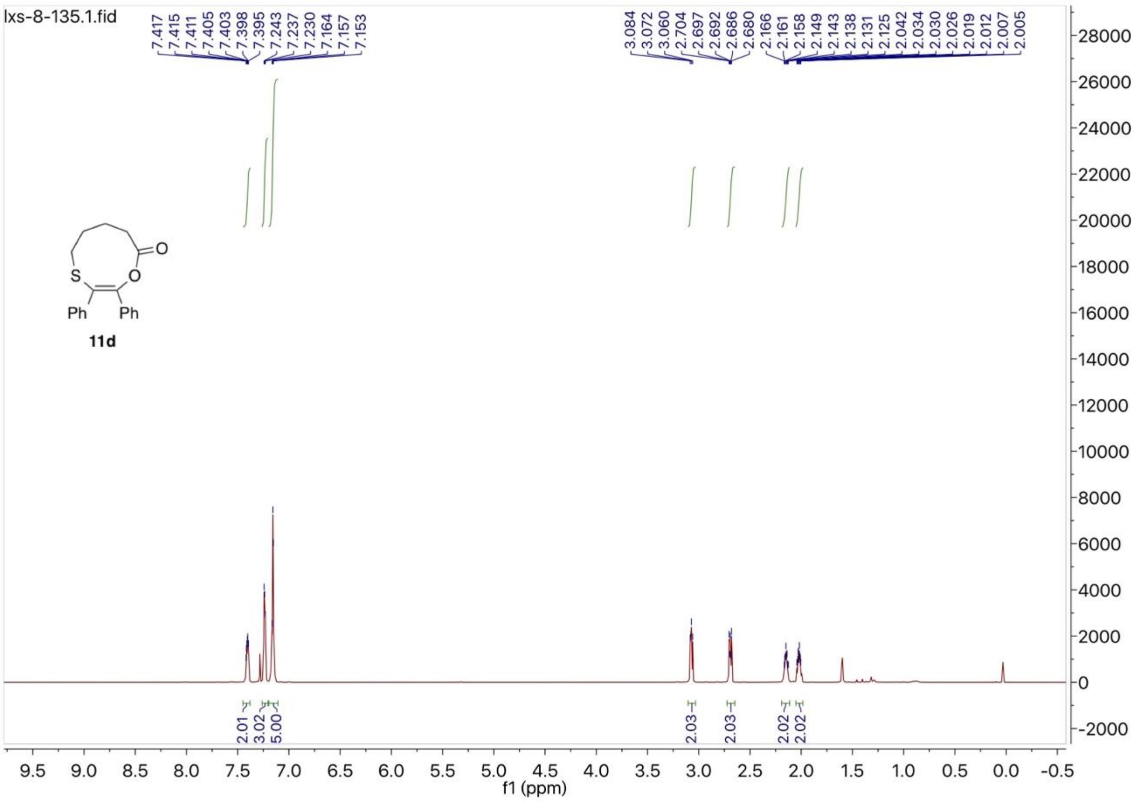

Supplementary Figure 237. ^{1}H NMR (500 MHz, $CDCl_3$) of compound **11d**

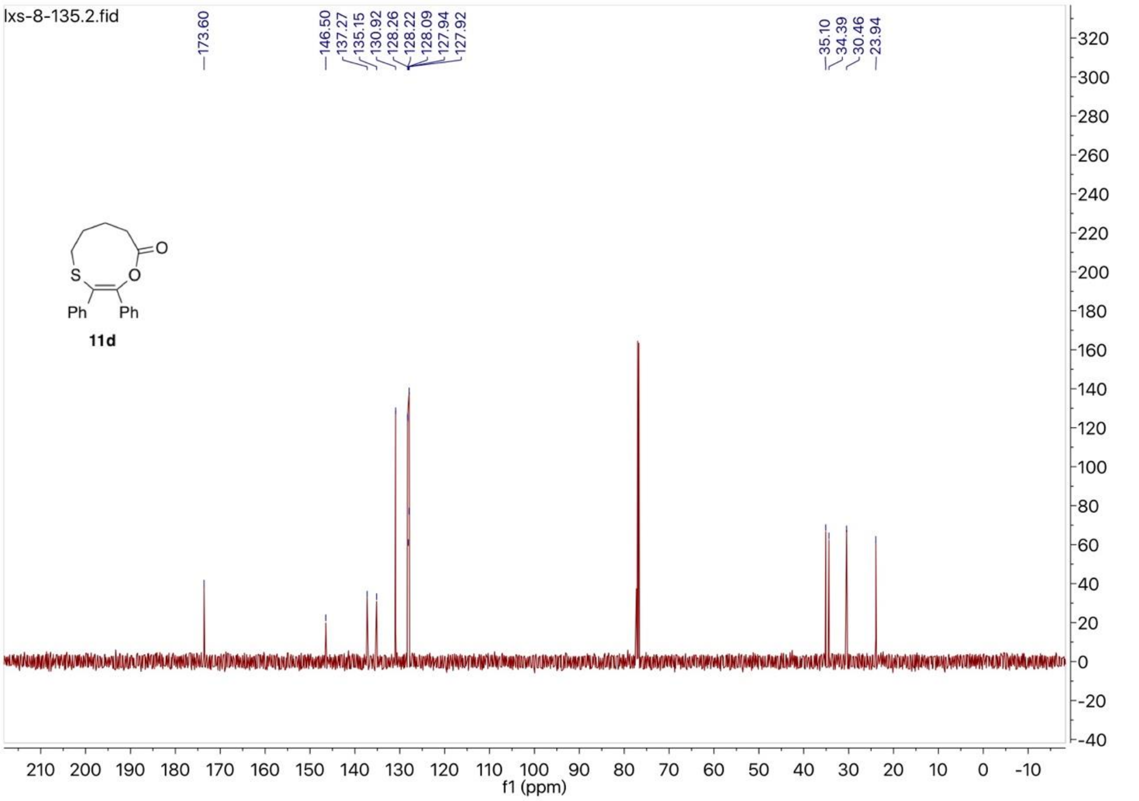

Supplementary Figure 238. ^{13}C NMR (125 MHz, $CDCl_3$) of compound **11d**

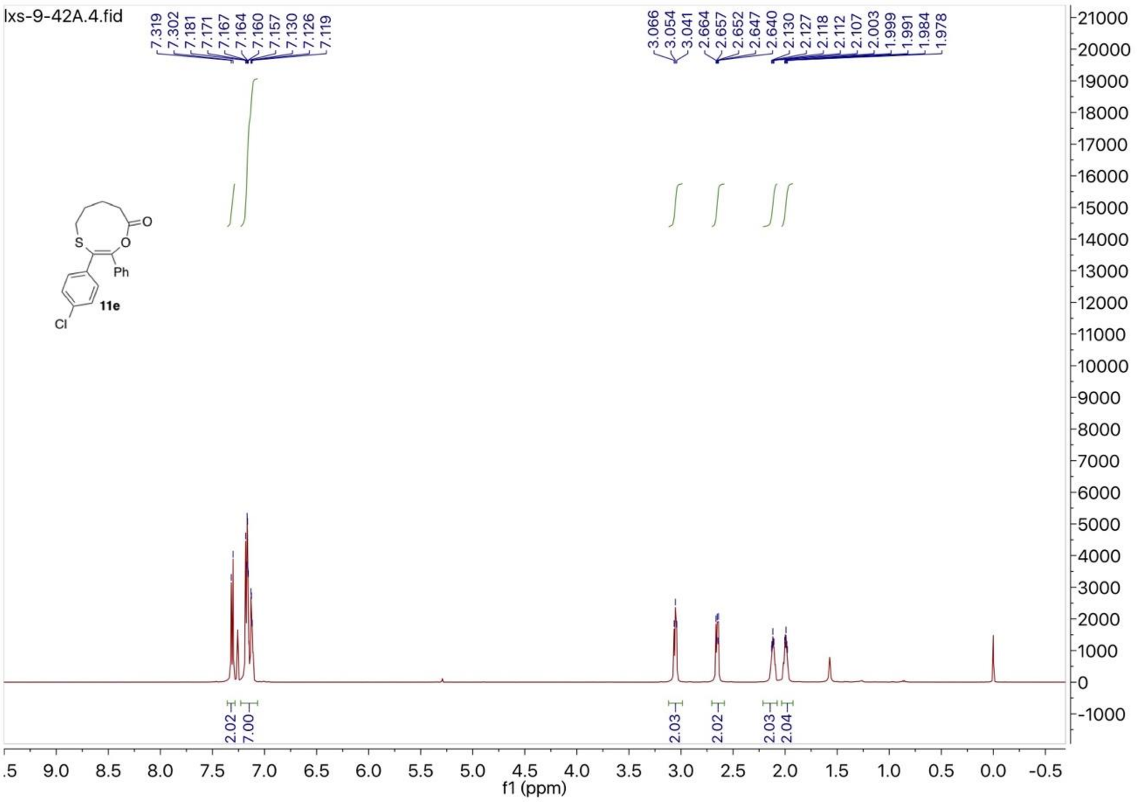

Supplementary Figure 239. ^{1}H NMR (500 MHz, $CDCl_3$) of compound **11e**

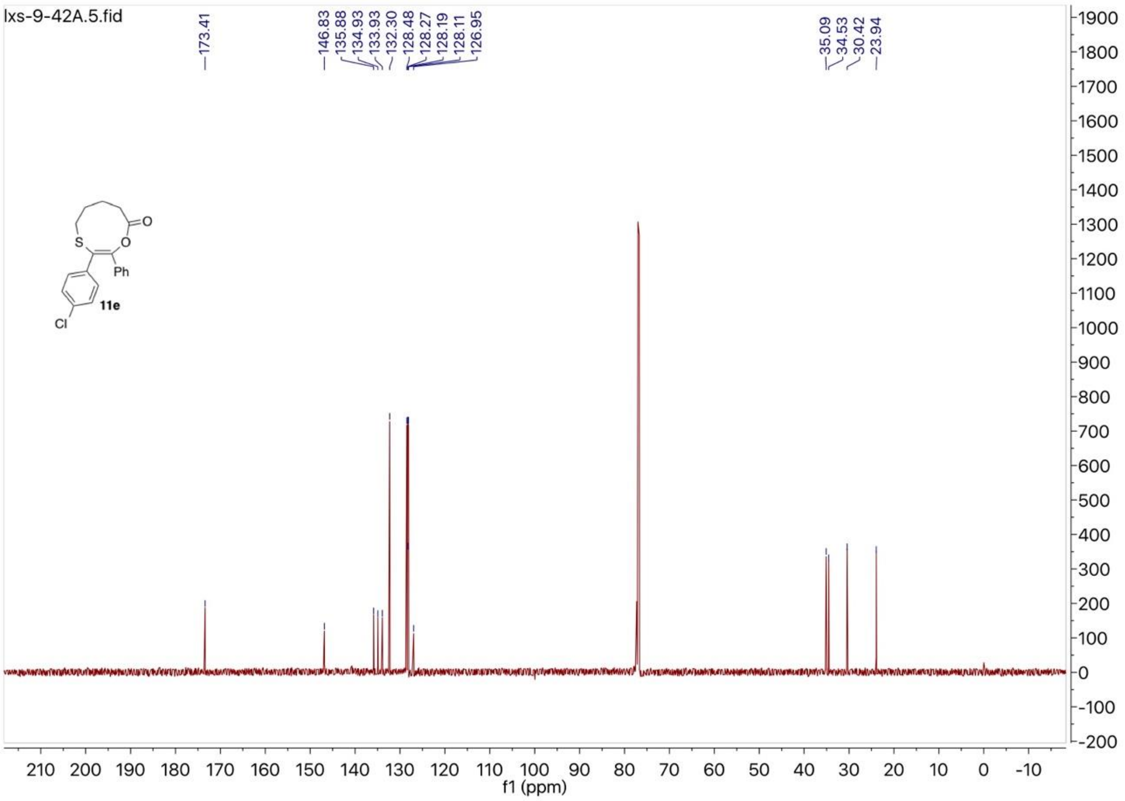

Supplementary Figure 240. ^{13}C NMR (125 MHz, $CDCl_3$) of compound **11e**

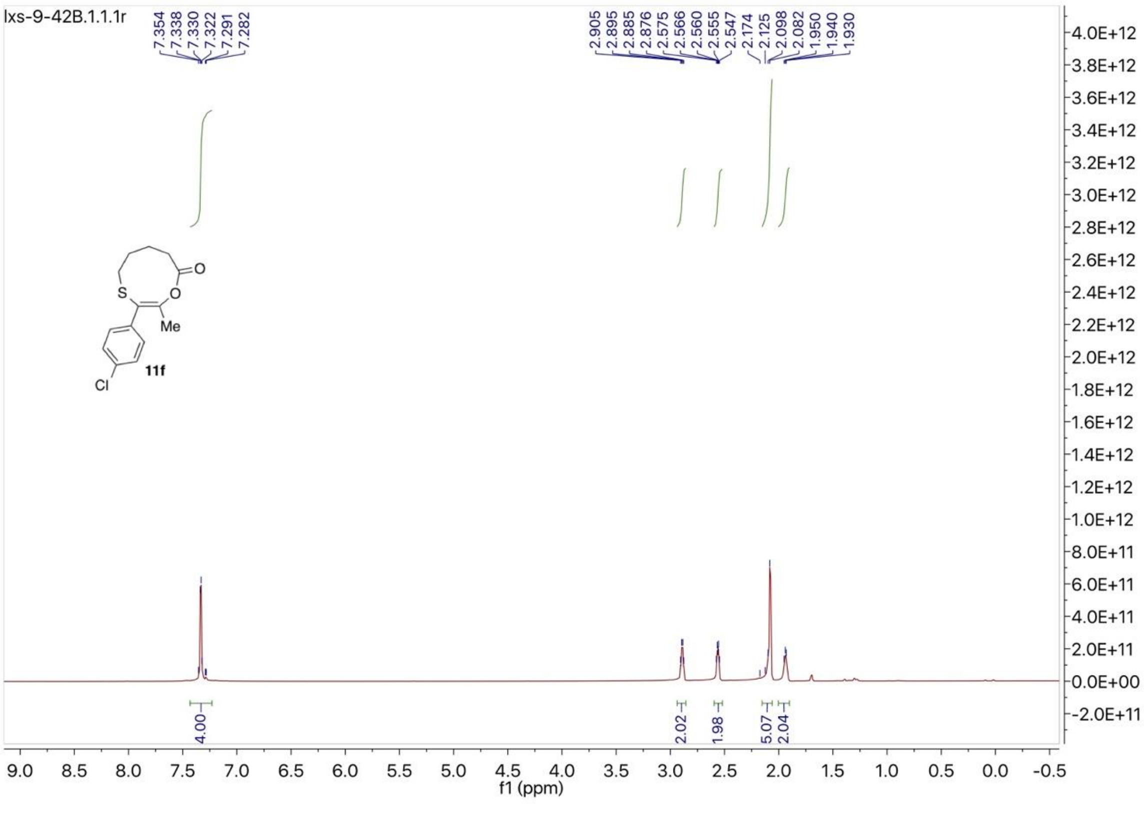

Supplementary Figure 241. ^{1}H NMR (600 MHz, $CDCl_3$) of compound **11f**

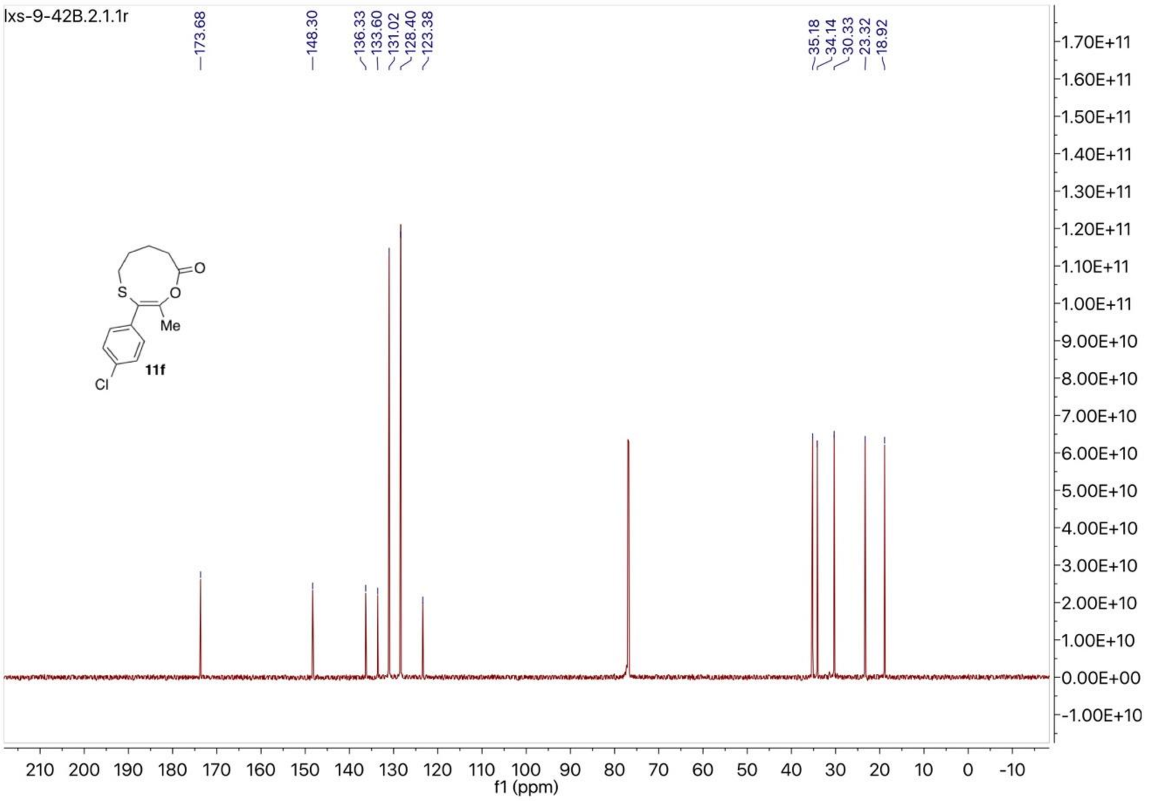

Supplementary Figure 242. ^{13}C NMR (150 MHz, $CDCl_3$) of compound **11f**

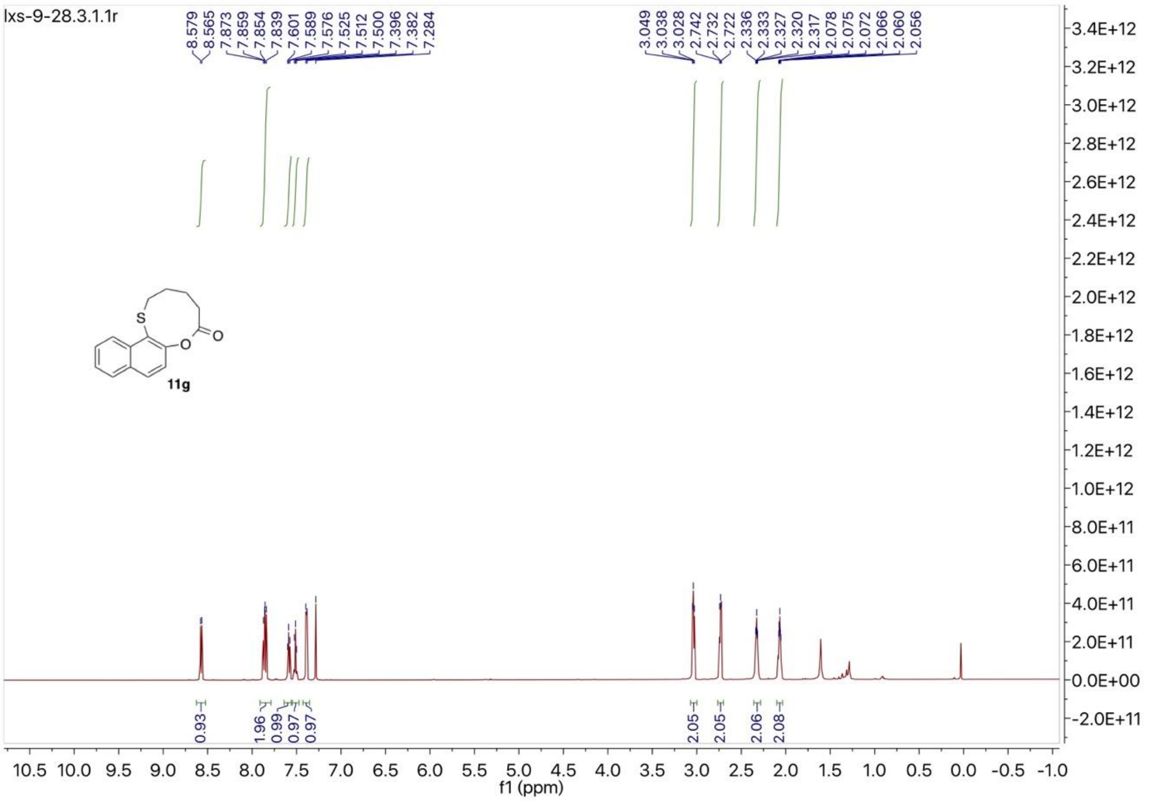

Supplementary Figure 243. ^{1}H NMR (600 MHz, $CDCl_3$) of compound **11g**

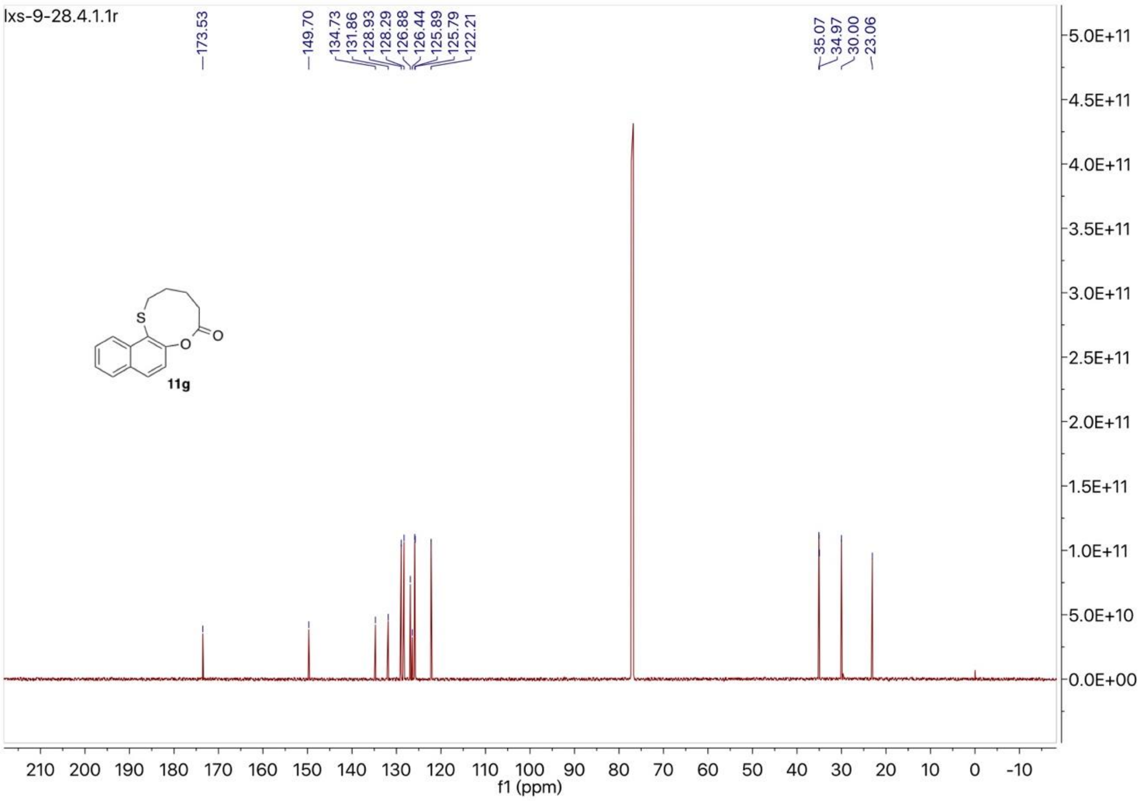

Supplementary Figure 244. ^{13}C NMR (150 MHz, $CDCl_3$) of compound **11g**

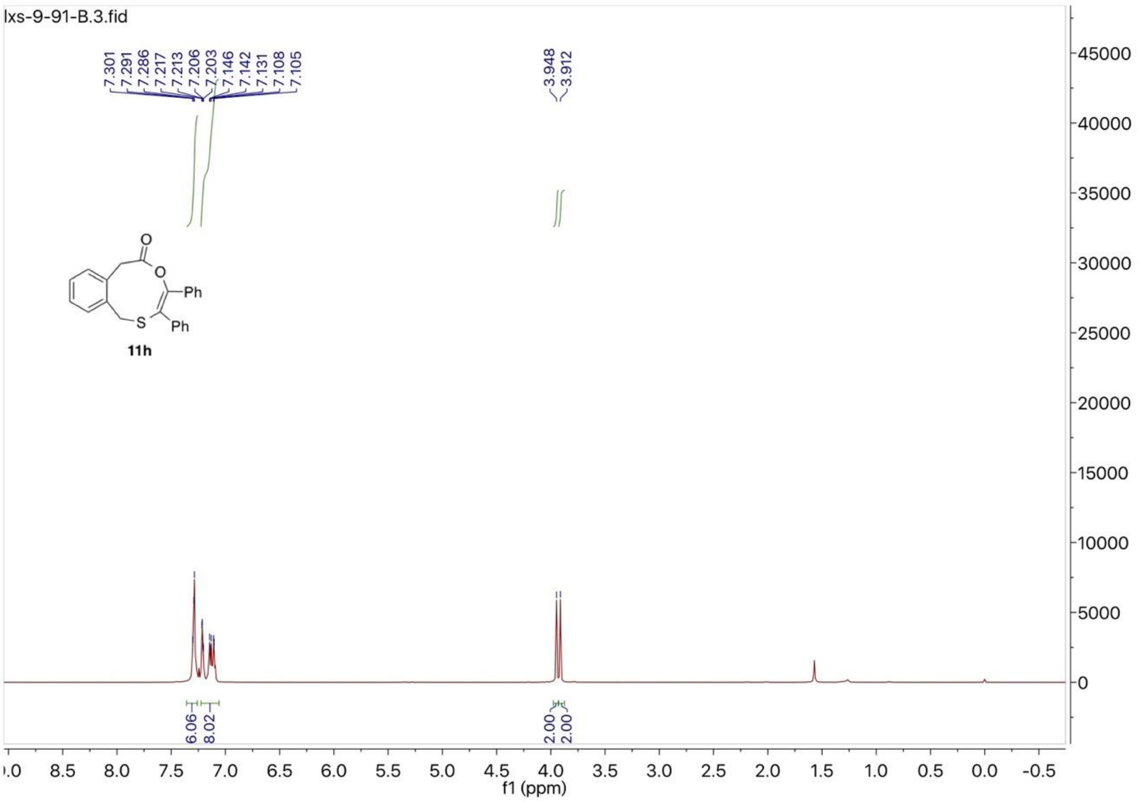

Supplementary Figure 245. ^{1}H NMR (500 MHz, $CDCl_3$) of compound **11h**

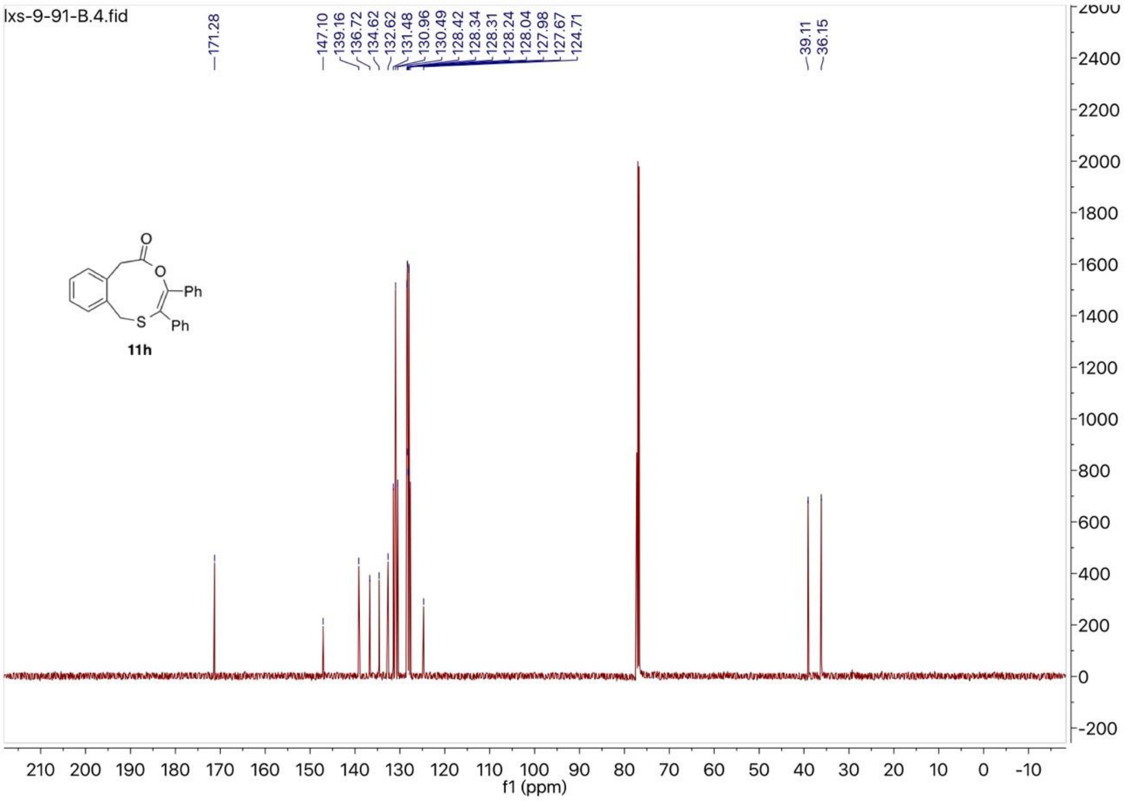

Supplementary Figure 246. ^{13}C NMR (125 MHz, $CDCl_3$) of compound **11h**

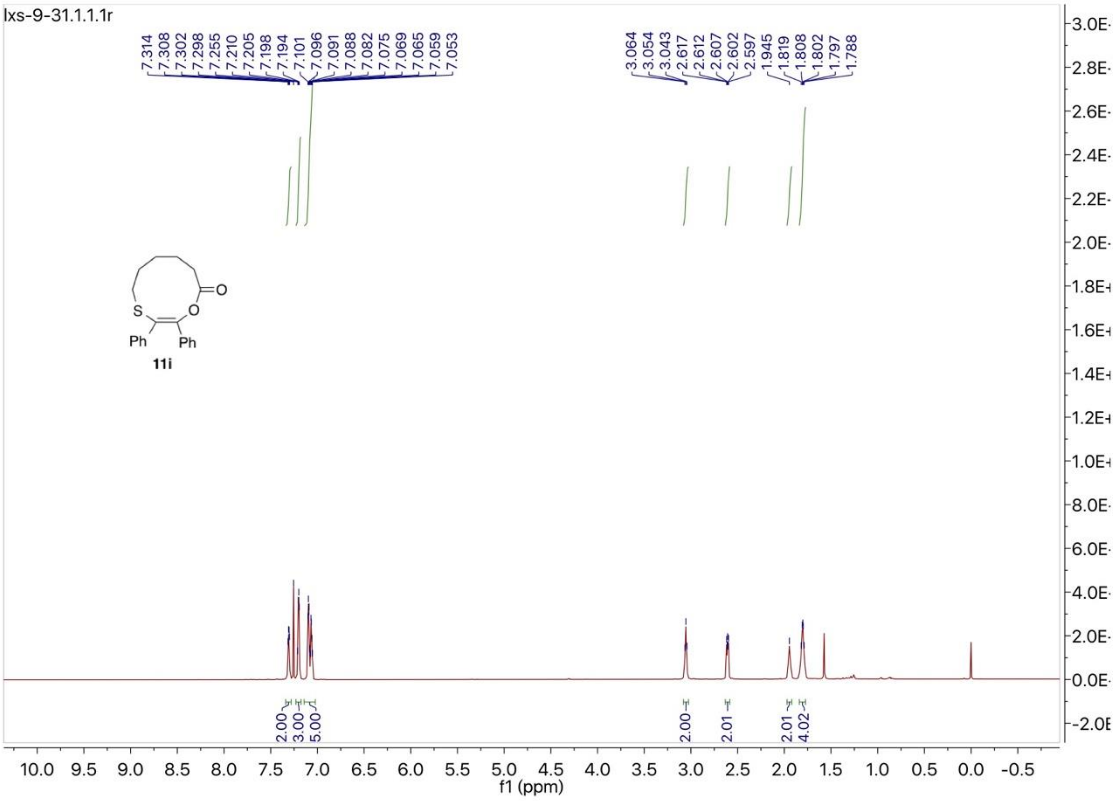

Supplementary Figure 247. ^{1}H NMR (600 MHz, $CDCl_3$) of compound **11i**

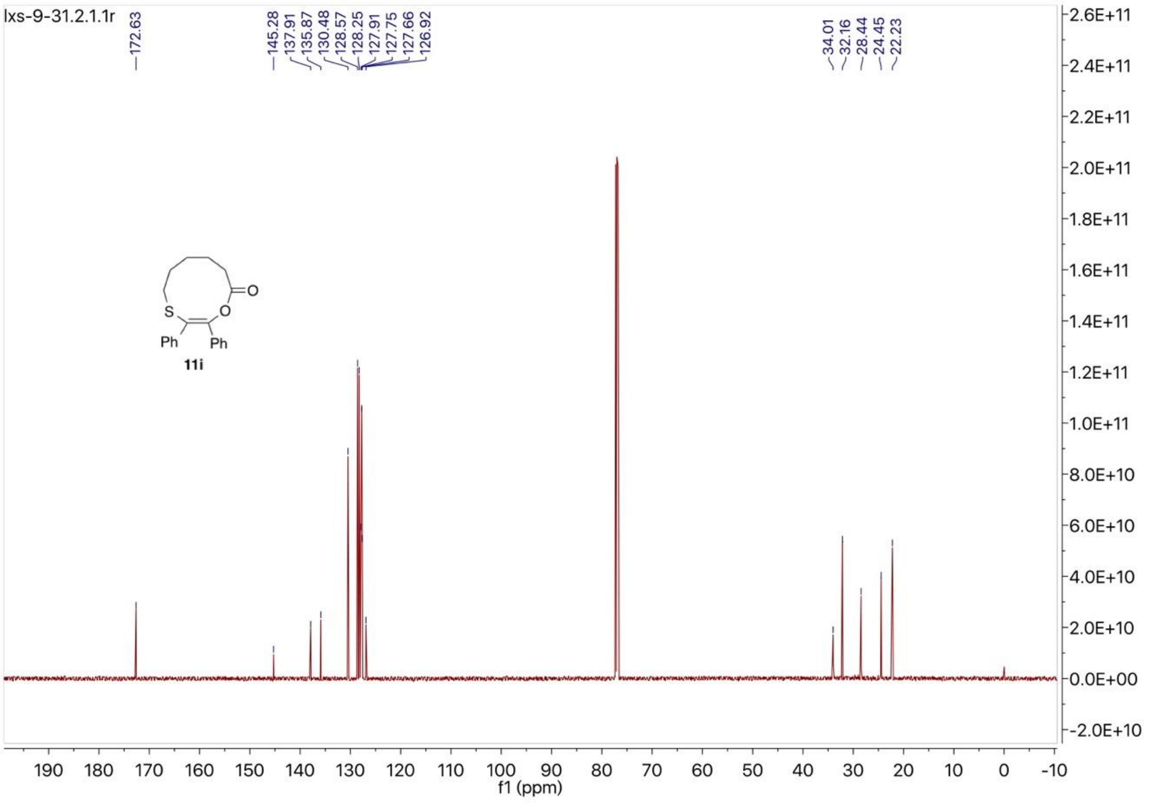

Supplementary Figure 248. ^{13}C NMR (150 MHz, $CDCl_3$) of compound **11i**

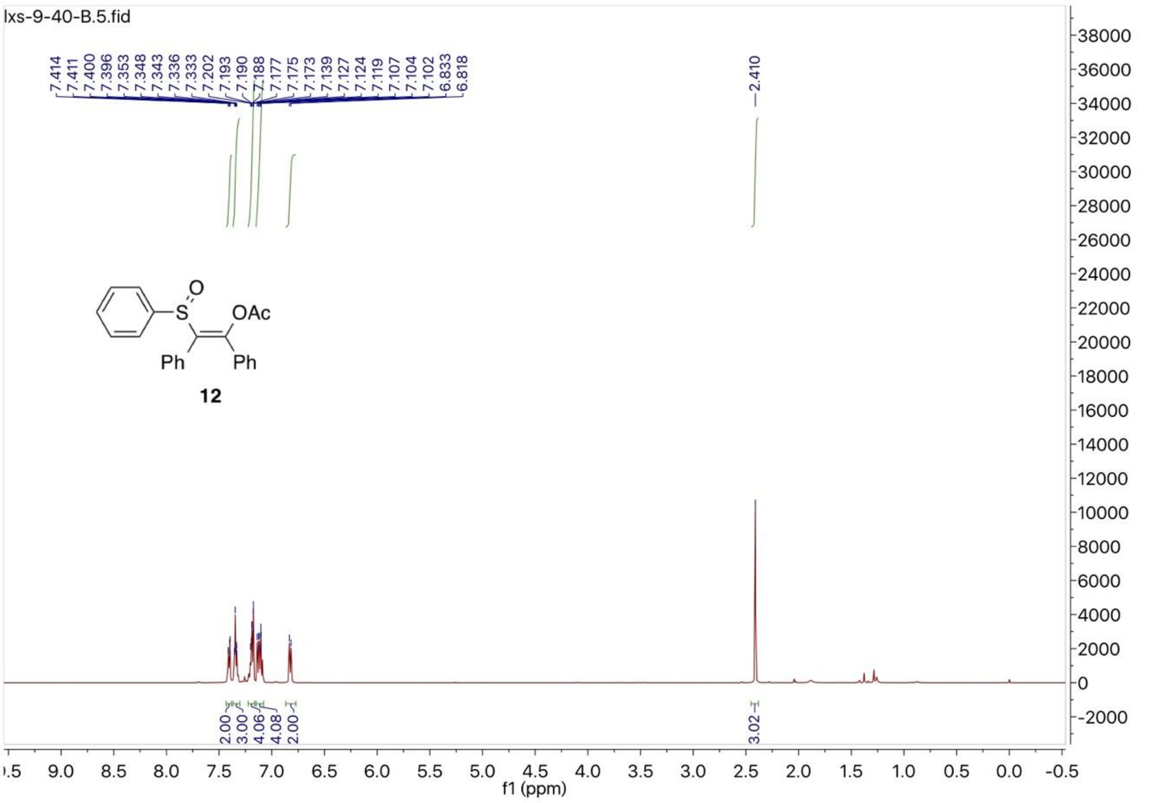

Supplementary Figure 249. ^{1}H NMR (500 MHz, $CDCl_3$) of compound **12**

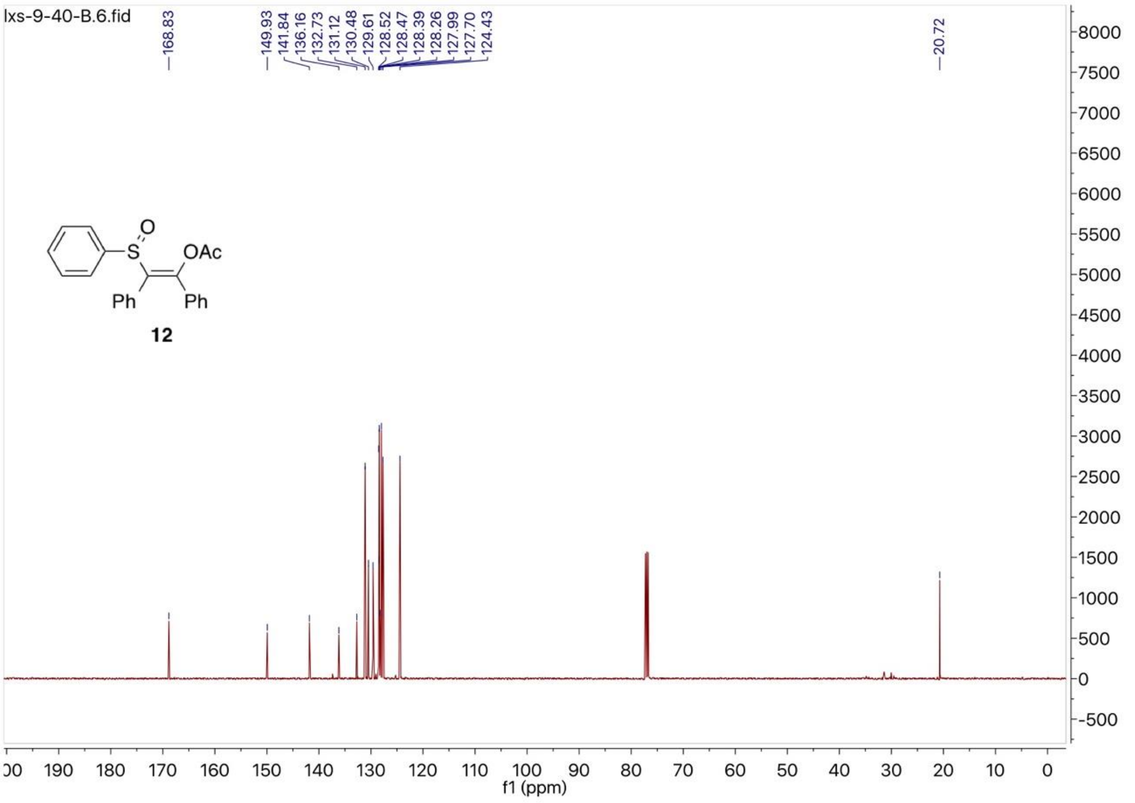

Supplementary Figure 250. ^{13}C NMR (125 MHz, $CDCl_3$) of compound **12**

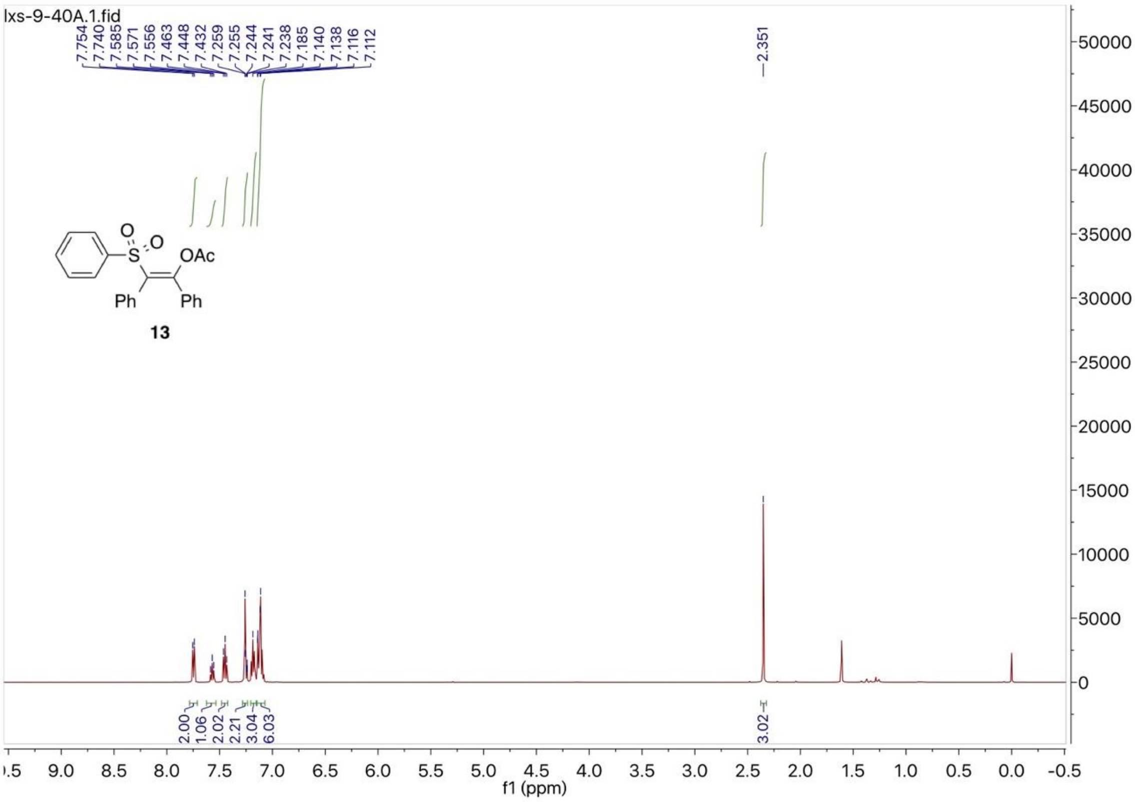

Supplementary Figure 251. ^{1}H NMR (500 MHz, $CDCl_3$) of compound **13**

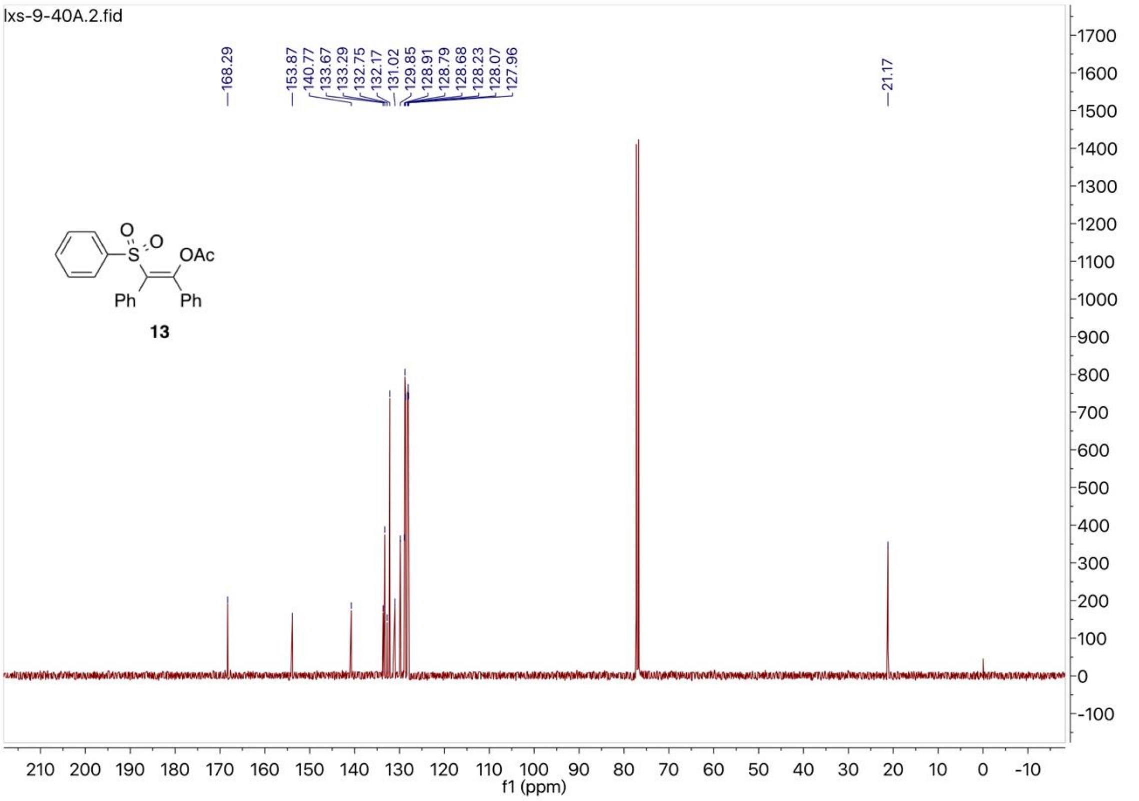

Supplementary Figure 252. ^{13}C NMR (125 MHz, $CDCl_3$) of compound **13**

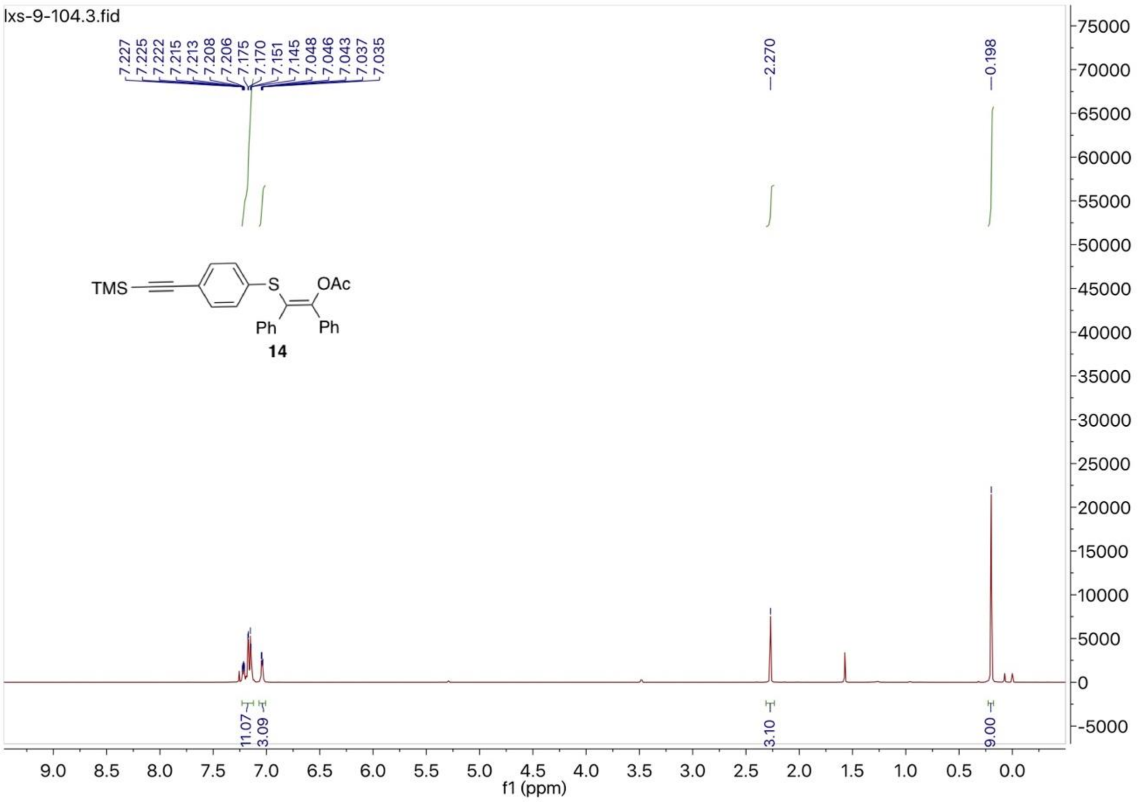

Supplementary Figure 253. ^{1}H NMR (500 MHz, $CDCl_3$) of compound **14**

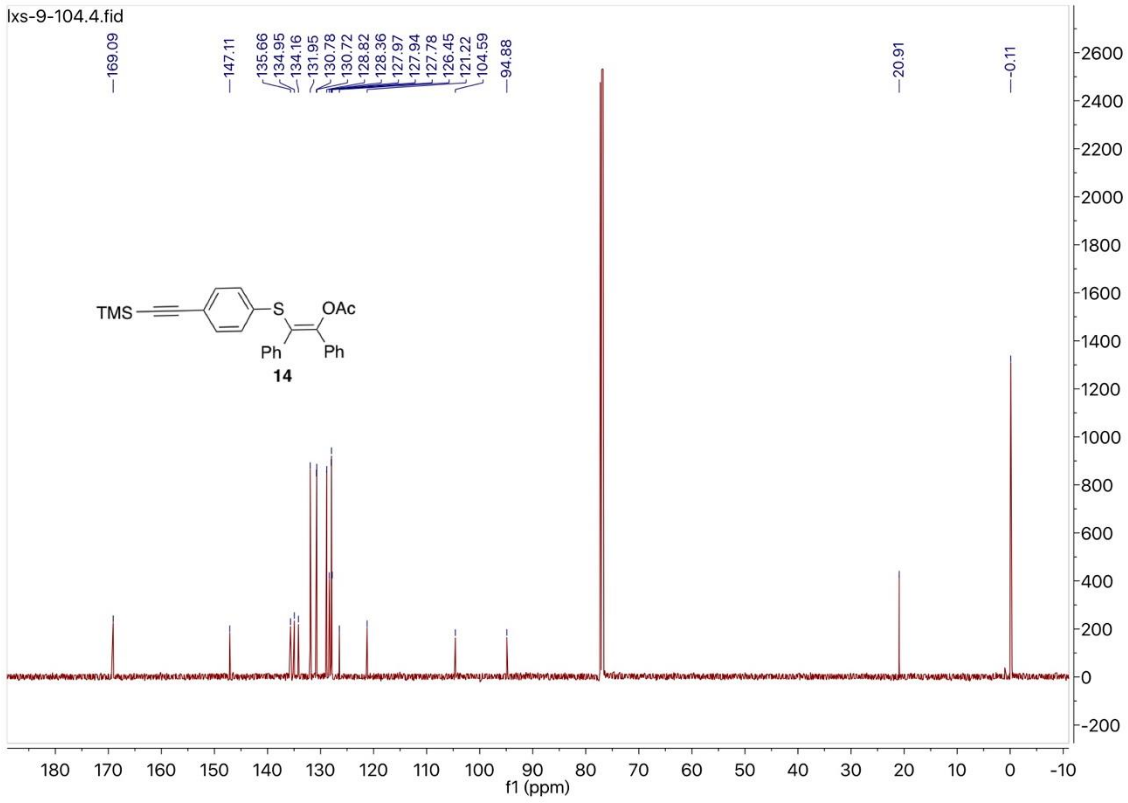

Supplementary Figure 254. ^{13}C NMR (125 MHz, $CDCl_3$) of compound **14**

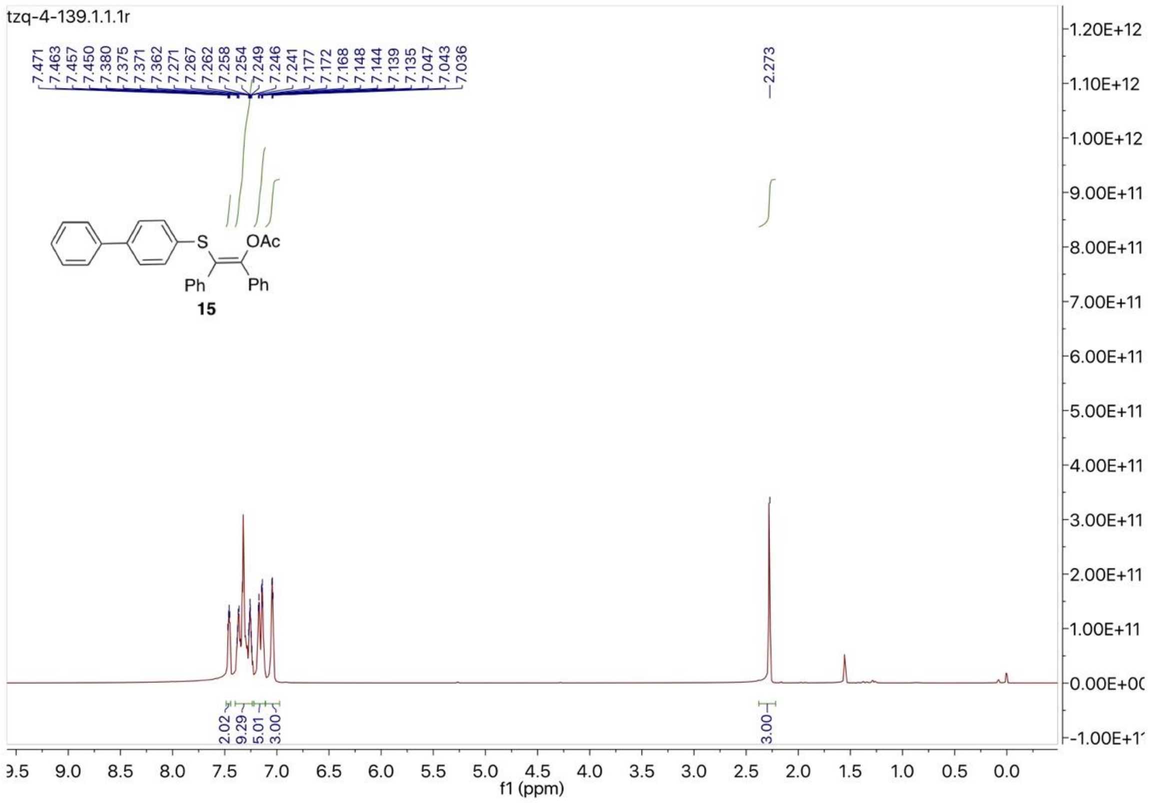

Supplementary Figure 255. ^{1}H NMR (600 MHz, $CDCl_3$) of compound **15**

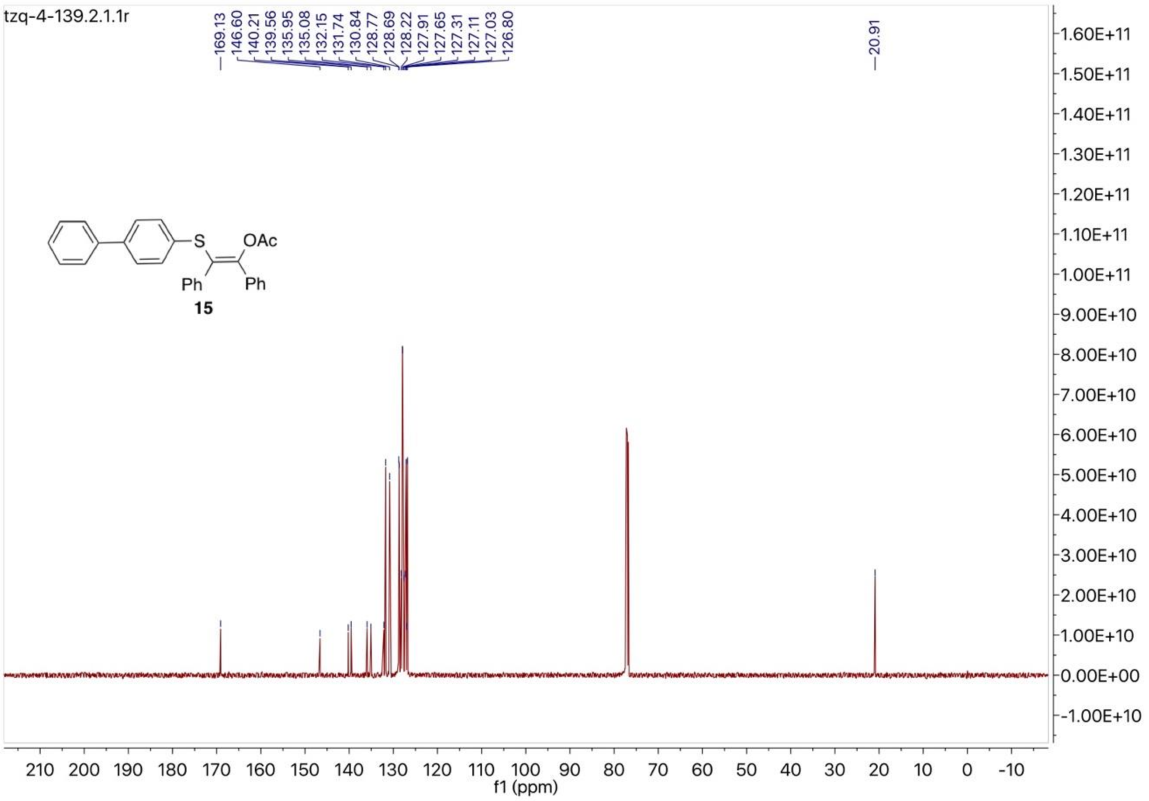

Supplementary Figure 256. ^{13}C NMR (150 MHz, $CDCl_3$) of compound **15**

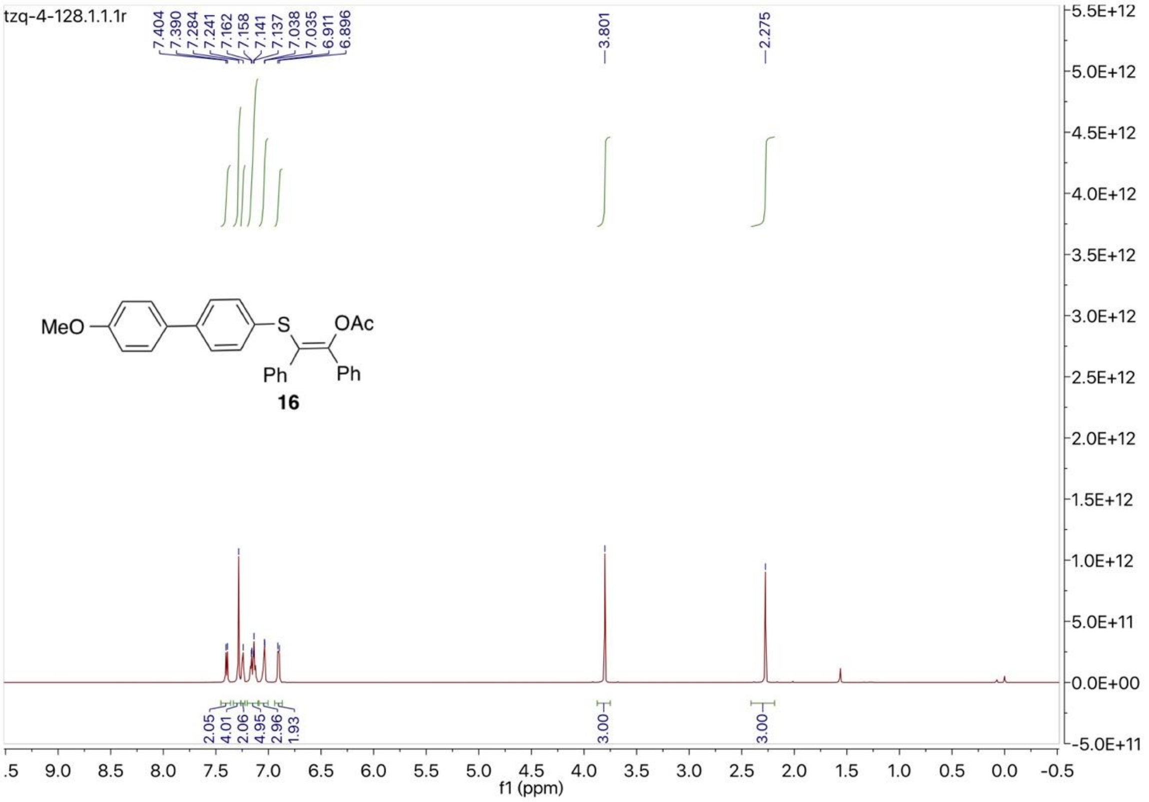

Supplementary Figure 257. ^{1}H NMR (600 MHz, $CDCl_3$) of compound **16**

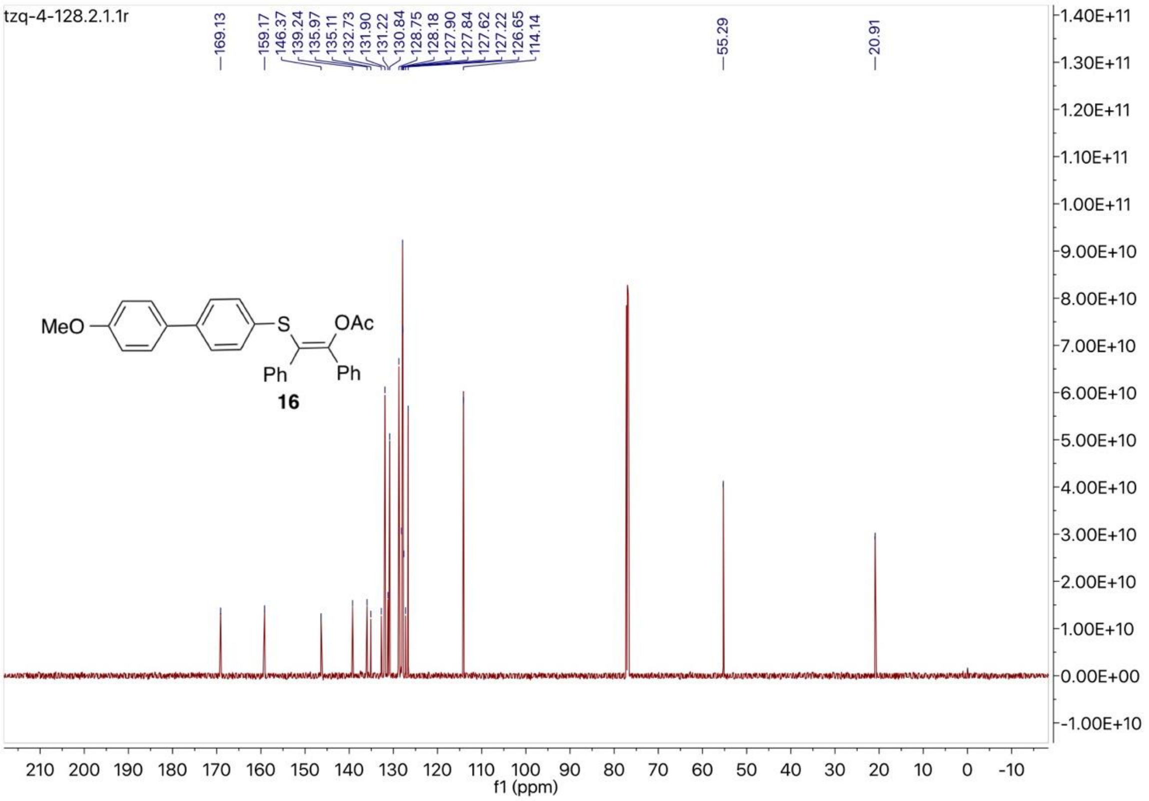

Supplementary Figure 258. ^{13}C NMR (150 MHz, $CDCl_3$) of compound **16**

Supplementary References

[1] Singh, P. & Peddinti, R. K. Harnessing the catalytic behaviour of 1,1,1,3,3,3-hexafluoro-2-propanol (HFIP): An expeditious synthesis of thioesters. *Tetrahedron Letters*, **58**, 1875 – 1878 (2017).

[2] Alieva, I. A., Belovezhetsb, L. A. & Oparina, L. A. Fungicidal Activity of *S*-Esters of Thiocarboxylic Acids as Antimicrobial Additives to Petroleum Products. *Pet. Chem.* **59**, 99 – 105 (2019).

[3] O'Driscoll, L. J. et. al. Carbazole‐Based Tetrapodal Anchor Groups for Gold Surfaces: Synthesis and Conductance Properties. *Angew. Chem. Int. Ed.* **59**, 882 – 889 (2020).

[4] Pijper, T. C., Robertus, J., Brownea, W. R. & Feringa, B. L. Mild Ti-mediated transformation of t-butyl thio-ethers into thio-acetates. *Org. Biomol. Chem.* **13**, 265 – 268 (2015).

[5] Xuan, M., Lu, C., Liu, M. & Lin, B.-L. Air-Tolerant Direct Thiol Esterification with Carboxylic Acids Using Hydrosilane via Simple Inorganic Base Catalysis. *J. Org. Chem.* **84**, 7694 – 7701 (2019).

[6] Chen, J. et. al. $AlCl_3$-promoted thiolation of acyl C–H bonds with arylsulfonyl hydrazides. Tetrahedron, **71**, 9496 – 9500 (2015).

[7] Lin, Y. A., Chalker, J. M., Floyd, N., Bernardes, G. J. L. & Davis, B. G. Allyl Sulfides Are Privileged Substrates in Aqueous Cross-Metathesis: Application to Site-Selective Protein Modification. *J. Am, Chem. Soc.* **130**, 9642 – 9643 (2008).

[8] Schuster, T., Schellenberger, S., Friedrich, R., Klapper, M. & Mullen, K. Branched fluorinated amphiphiles based on carbohydrates. *J. Fluorine. Chem.* **154**, 30 – 36 (2013).

[9] Crich, D. & Sana, K. S_N2-Type Nucleophilic Opening of *β*-Thiolactones (Thietan-2-ones) as a Source of Thioacids for Coupling Reactions. *J. Org. Chem.* **74**, 3389 – 3393 (2009).

[10] Lee, H. B., Park, H.-Y., Lee, B.-S. & Kim, Y. G. Downfield chemical shifts at *α*-protons and carbons of *β*-propiothiolactones. *Magn. Reson. Chem.* **38**, 468 – 471 (2000).

[11] McCourt, R. O. & Scanlan, E. M. A Sequential Acyl Thiol–Ene and Thiolactonization Approach for the Synthesis of *δ*-Thiolactones. *Org. Lett.* **21**, 3460 – 3464 (2019).

[12] Ogiwara, Y., Takano, K., Horikawa, S. & Sakai, N. Indium-Catalyzed Direct Conversion of Lactones into Thiolactones Using a Disilathiane as a Sulfur Source. *Molecules*, **23**, 1339 (2018).

[13] Bannin, T. J. & Kiesewetter, M. K. Poly(thioester) by Organocatalytic Ring-Opening Polymerization. *Macromolecules*, **48**, 5481 – 5486 (2015).

[14] Steliou, R., Salama, P. & Corriveau, J. Reagents for organic synthesis. 4. Group 14 metal assisted carbon-sulfur bond formation. *J. Org. Chem.* **50**, 4969 – 4971 (1985).

[15] Jiang, Y., Khong, V. Z. Y., Lourdusamy, E. & Park, C.-M. Synthesis of 2-aminofurans and 2-unsubstituted furansviacarbenoid-mediated [3 + 2] cycloaddition. *Chem. Commun.*, **48**, 3133 – 3135 (2012).

[16] Ye, F. et. al. Palladium-Catalyzed C–H Functionalization of Acyldiazomethane and Tandem Cross-Coupling Reactions. *J. Am. Chem. Soc.* **137**, 4435 – 4444 (2015).

[17] Kidonakis, M. & Stratakis, M. Au Nanoparticle-Catalyzed Insertion of Carbenes from *α*-Diazocarbonyl Compounds into Hydrosilanes. *Org. Lett.* **20**, 4086 – 4089 (2018).

[18] Zhang, J. et. al. Tandem Synthesis of α-Diazoketones from 1,3-Diketones. *J. Org. Chem.* **82**, 9171 – 9174 (2017).

[19] Thurow, S. et. al. Preparation of Organic Nitrates from Aryldiazoacetates and $Fe(NO_3)_3 \cdot 9H_2O$. *Org. Lett.* **21**, 6909 – 6913 (2019).

[20] Davies, H. M. L., Hansen, T. & Churchill, M. R. Catalytic Asymmetric C−H Activation of Alkanes and Tetrahydrofuran. *J. Am. Chem. Soc.* **122,** 3063 – 3070 (2000).

[21] Peng, C., Cheng, J. & Wang, J. Sequential Copper(I)-Catalyzed Reaction of Amines with *o*-Acetylenyl-Substituted Phenyldiazoacetates. *Adv. Synth. Catal.* **350**, 2359 – 2364 (2008).

[22] Kitamura, M., Tashiro, N., Sakata, R. & Okauchi, T. Synthesis of Diazonaphthoquinones from Naphthols by Diazo-Transfer Reaction with 2-Azido-1,3-dimethylimidazolinium Chloride. *Synlett*, **16**, 2503 – 2505 (2010).

[23] Becke, A. D. Density ‐ functional thermochemistry. III. The role of exact exchange. *J. Chem. Phys.* **98**, 5648 (1993).

[24] Stephens, P. J., Devlin, F. J., Chabalowski, C. F. & Frisch, M. J. Ab Initio Calculation of Vibrational Absorption and Circular Dichroism Spectra Using Density Functional Force Fields. *J. Phys. Chem*. ***98***, 11623 (1994).

[25] Lee, C., Yang, W. & Parr, R. G. Development of the Colle-Salvetti correlation-energy formula into a functional of the electron density. *Phys. Rev. B: Condens. Matter Mater. Phys.* **37**, 785 (1998).

[26] M. J. Frisch, G. W. Trucks, H. B. Schlegel, G. E. Scuseria, M. A. Robb, J. R. Cheeseman, G. Scalmani, V. Barone, B. Mennucci, G. A. Petersson, H. Nakatsuji, M. Caricato, X. Li, H. P. Hratchian, A. F. Izmaylov, J. Bloino, G. Zheng, J. L. Sonnenberg, M. Hada, M. Ehara, K. Toyota, R. Fukuda, J. Hasegawa, M. Ishida, T. Nakajima, Y. Honda, O. Kitao, H. Nakai, T. Vreven, J. A., Jr. Montgomery, J. E. Peralta, F. Ogliaro, M. Bearpark, J. J. Heyd, E. Brothers, K. N. Kudin, V. N. Staroverov, R. Kobayashi, J. Normand, K. Raghavachari, A. Rendell, J. C. Burant, S. S. Iyengar, J. Tomasi, M. Cossi, N. Rega, N. J. Millam, M. Klene, J. E. Knox, J. B. Cross, V. Bakken, C. Adamo, J. Jaramillo, R. Gomperts, R. E. Stratmann, O. Yazyev, A. J. Austin, R. Cammi, C. Pomelli, J. W. Ochterski, R. L. Martin, K. Morokuma, V. G. Zakrzewski, G. A. Voth, P. Salvador, J. J. Dannenberg, S. Dapprich, A. D. Daniels, O. Farkas, J. B. Foresman, J. V. Ortiz, J. Cioslowski, D. J. Fox, *Gaussian 09*, Revision D.01; Gaussian, Inc.: Wallingford, CT, 2009.

[27] Zhao, Y. & Truhlar, D. G. The Mo6 suite of density functionals for main group thermochemistry, thermochemical kinetics, noncovalent interactions, excited states, and transition elements: two new

functionals and systematic testing of four Mo6-class functionals and 12 other functionals. *Theor. Chem. Acc.* **120**, 215 – 241 (2008).

[28] T. H. Dunning Jr, P. J. Hay, In Modern Theoretical Chemistry, Ed. H. F. Schaefer III, Vol. 3, 1-28 (Plenum, New York, 1977)

[29] Fuentealba, P., Preuss, H., Stoll, H. & Szentpály, L. V. A proper account of core-polarization with pseudopotentials: single valence-electron alkali compounds. *Chem. Phys. Lett.* **89**, 418 (1982).

[30] Marenich, A. V., Cramer, C. J. & Truhlar, D. G. Universal Solvation Model Based on Solute Electron Density and on a Continuum Model of the Solvent Defined by the Bulk Dielectric Constant and Atomic Surface Tensions. *J. Phys. Chem. B* **113**, 6378 (2009).
